# Supplementary material for: Nickel-catalyzed direct stereoselective α-allylation of ketones with non-conjugated dienes
Source: Nat Commun. 2023 Nov 22;14:7640. doi: 10.1038/s41467-023-43197-z (PMC10665391; doi:10.1038/s41467-023-43197-z)
Supplement: Supplementary file 1 — Supplementary Information [file 41467_2023_43197_MOESM1_ESM.pdf]

Nickel-Catalyzed Direct Stereoselective α-Allylation of Ketones with Non-conjugated Dienes

Yi-Xuan Cao[1], Matthew D. Wodrich[2], Nicolai Cramer[1]*

1. Laboratory of Asymmetric Catalysis and Synthesis, Institute of Chemical Sciences and Engineering, Ecole Polytechnique Fédérale de Lausanne (EPFL), Lausanne, Switzerland.
2. Laboratory for Computational Molecular Design, Institute of Chemical Sciences and Engineering, Ecole Polytechnique Fédérale de Lausanne (EPFL), 1015 Lausanne, Switzerland.

*e-mail: nicolai.cramer@epfl.ch

Supplementary Information

Table of Contents

1. General information **3**

2. Ligand synthesis **4**

2.1 Achiral ligand synthesis 4

2.2 Chiral ligand synthesis 11

3. Starting material synthesis **20**

4. Nickel(0) NHC catalyzed hydro-alkylation reaction **23**

4.1 Optimization of diastereoselective hydro-alkylation of non-conjugated dienes 23

4.2 Control experiments 24

4.3 Scope of diastereoselective hydro-alkylation of nonconjugated dienes. 25

4.4 Optimization of enantioselective hydro-alkylation of non-conjugated dienes. 44

4.5 Scope of enantioselective hydro-alkylation of non-conjugated dienes. 46

5. Mechanistic studies **71**

5.1 Nickel complex synthesis. 71

5.2 Calculation of percent Buried Volume (%V_{bur}). 72

5.3 Reactivity investigation of nickel complex 76

5.4 NMR experiment1. 77

5.5 NMR experiment2. 78

5.6 NMR experiment3. 79

5.7 Deuterium labelling experiment. 80
5.8 Deuterium crossover experiment. 81
5.9 Deuterium scrambling experiment. 82
5.10 Kinetic isotope effect (KIE). 87
5.11 Kinetic studies. 90

6. Computational studies **92**
7. NMR spectra **98**
8. Reference **174**

1. General information

All reactions were carried out under an atmosphere of nitrogen in oven-dried glassware with magnetic stirring, unless otherwise indicated. Toluene, dichloromethane, tetrahydrofuran, acetonitrile and diethyl ether were purified by an Innovative Technology Solvent Delivery System. Chemicals were used as obtained from the suppliers. Flash chromatography was performed with Silicycle silica gel 60 (0.040-0.063 μm grade) or acidic alumina (C. Roth, Aluminium oxide 90 acidic). Analytical thin-layer chromatography was performed with commercial glass plates coated with 0.25 mm silica gel (E. Merck, Kieselgel 60 F254). Compounds were either visualised under UV-light at 254 nm or by dipping the plates in an aqueous potassium permanganate solution followed by heating. Proton nuclear magnetic resonance (^{1}H-NMR) data were acquired on a Bruker AV400 (400 MHz) or a Bruker DRX600 (600 MHz). Chemical shifts (δ) are reported in parts per million (ppm) relative to incompletely deuterated $CDCl_3$ (s, 7.26 ppm), C_6D_6 (s, 7.16 ppm), CD_3OD (p, 3.31 ppm). Splitting patterns are designated as s, singlet; d, doublet; t, triplet; q, quartet; p, pentet; dd, doublet of doublets; qd, quadruplet of dublets; m, multiplet; br, broad. Proton decoupled Carbon-13 nuclear magnetic resonance (^{13}C-NMR) data were acquired on a Bruker AV400 (100 MHz) and a Bruker DRX600 (150 MHz) spectrometer. Chemical shifts are reported in ppm relative to $CDCl_3$ (77.16 ppm), C_6D_6 (128.06 ppm), CD_3OD (49.00 ppm). Proton decoupled Fluorine-19 nuclear magnetic resonance (^{19}F-NMR) were acquired at 376 MHz on a Bruker AV400 spectrometer. Infrared (IR) data were recorded on an Alpha-P Bruker FT-IR Spectrometer. Absorbance frequencies are reported in reciprocal centimeters (cm^{-1}). HRMS measurements were performed by an Agilent LC-MS TOF. High resolution mass is given in m/z. Enantiomeric excesses were measured on an Agilent or Waters HPLC, or on a Thar SFC Investigator system using chiral stationary phase columns. Optical rotations were measured on a Polartronic M polarimeter using a 0.5 cm cell with a Na 589 nm filter. X-ray analysis was performed by Dr. R. Scopelliti and Dr. F. Fadaei Tirani at the EPF Lausanne.

2. Ligand synthesis.

1.1 Achiral ligand synthesis.

4-(*tert*-butyl)-2,6-diisopropylaniline (**a1**):

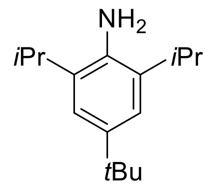

a1

The aniline was synthesized by a slightly modified procedure according to literature[1]. Under atmosphere of N_2 $AlCl_3$ (4.00 g, 30.0 mmol) was dissolved in 1,2-dichloroethane (25 mL) and cooled to 0 °C. Subsequently, 2,6-diisopropylaniline (4.00 g, 22.6 mmol) was added, *t*BuCl (7.88 g, 85.2 mmol) was added dropwise over a period of 10 min, during which the solution turned dark brown. Then the reaction was allowed to slowly warm up to room temperature. After having stirred the solution overnight, dichloromethane (50 mL) was added and the reaction mixture was poured on a mixture of ice (40 g) and aqueous ammonia solution (28 %, 12 mL). The white precipitate was filtered off. The organic phase was separated, washed with water (2 × 40 mL) and brine (2 ×40 mL) and dried with $MgSO_4$. The solvent was removed under reduced pressure giving a viscous yellow oil which was recrystallized from pentane (4 mL) at –30 °C. The crystals were further purified by sublimation (45 °C, 9.0 × 10^{-2} mbar) overnight to give the pure product **a1** as a white solid. Yield: 2.8 g, 12.0 mmol, 53%.

2,6-dibenzhydryl-4-(*tert*-butyl)aniline (**a2**):

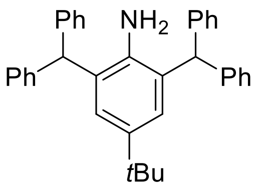

a2

The aniline **a2** was synthesized according to literature[2].

(1*E*,2*E*)-*N*[1],*N*[2]-bis(4-(tert-butyl)-2,6-diisopropylphenyl)ethane-1,2-diimine (**a3**):

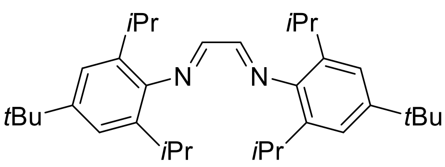

a3

Aniline **a1** (1930 mg, 8.27 mmol), and aqueous solution of glyoxal (8.8 M, 0.47 mL, 4.2 mmol) were dissolved in THF (40 mL) with several drops of formic acid and stirred for 24 h at 70 °C. To the resulting mixture, EtOAc and water were added. The organic layer was separated and washed with brine and water, and dried over $MgSO_4$. The organic layer was concentrated in vacuo to give a diimine as a yellow solid (1900 mg, ca. 93%). The diimine **a3** was used for the subsequent reaction without further purification.

^{1}H NMR (400 MHz, $CDCl_3$) δ 8.11 (s, 2H), 7.19 (s, 4H), 2.96 (hept, J = 6.9 Hz, 4H), 1.35 (s, 18H), 1.21 (d, J = 6.9 Hz, 24H). **^{13}C NMR** (101 MHz, $CDCl_3$) δ 163.4, 147.6, 145.7, 136.2, 120.2, 34.9, 31.7, 28.4, 23.7. **IR (ATR): $\tilde{\nu}$ (cm^{-1}) =** 2956, 2903, 2868, 1632, 1476, 1462, 1362, 1276, 1188, 875. **HRMS (ESI) (m/z) calcd. for $C_{34}H_{53}N_2^+$:** 489.4203; Found:489.4197. **m. p.:** decompose before reaching m.p.

(2*E*,3*E*)-*N*[2],*N*[3]-bis(4-(*tert*-butyl)-2,6-diisopropylphenyl)butane-2,3-diimine (**a4**):

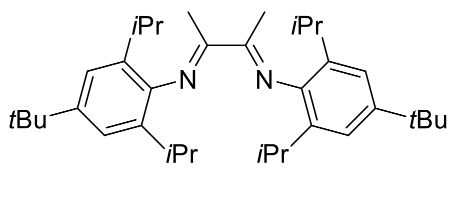

a4

A slightly modified literature procedure[3] was used to synthesize **a4**. To a round-bottom equipped with a stir-bar, EtOH (0.5 M), 2,3-butanedione (1 eq), substituted aniline **a1** (2 eq), and several drops of formic acid was added. This solution was allowed to heat at 70 ºC. A yellow precipitate is formed after a few hours. After stirring for 15 hours, the EtOH was evaporated and the product **a4** was purified by column chromatography on silica gel (pentane:DCM, 20:1) as a yellow solid. yield: 70%.

^{1}H NMR (400 MHz, $CDCl_3$) δ 7.16 (s, 4H), 2.71 (hept, *J* = 6.8 Hz, 4H), 2.06 (s, 6H), 1.35 (s, 18H), 1.35 (s, 18H), 1.19 (m, 24H). **^{13}C NMR** (101 MHz, $CDCl_3$) δ 168.4, 146.1, 143.8, 134.3, 119.9, 34.8, 31.8, 28.8, 23.2, 23.0, 16.8. **IR (ATR): $\tilde{\nu}$ (cm^{-1}) =** 2958, 2902, 2868, 1703, 1646, 1461, 1361, 1283, 1190, 1121, 876, 812. **HRMS (ESI) (m/z) calcd. for $C_{36}H_{57}N_2^+$:** 517.4516; Found: 517.4529. **m. p.:** 185-187 ºC. **R_f:** 0.8 (Pentane:DCM 10:1).

<u>(2*E*,3*E*)-*N*^{2},*N*^{3}-bis(2,6-dibenzhydryl-4-(tert-butyl)phenyl)butane-2,3-diimine (**a5**):</u>

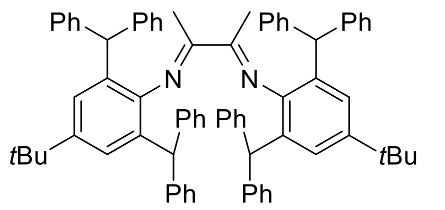

a5

A slightly modified literature procedure[4] was used to synthesize **a5**. To a mixture of 2,6-dibenzhydryl-4-(*tert*-butyl)aniline **a2** (6.4 mmol, 3.1 g) and 2,3-butadione (3.2 mmol, 276 mg) in toluene (20 mL), a catalytic amount of p-toluene sulfonic acid (0.15 g) was added and heated to 80 ºC for 9 h and then the reaction was refluxed using Dean-stark trap for another 72 h. After all the volatile was evaporated under reduced pressure and the crude product was purified by column chromatography using basic alumina with pentane/ethyl acetate (v/v = 500: 2) as eluent to afforded the **a5** as a yellow powder (2.2 g, 68%).

<u>*N*^{1},*N*^{2}-bis(4-(*tert*-butyl)-2,6-diisopropylphenyl)ethane-1,2-diamine (**a6**):</u>

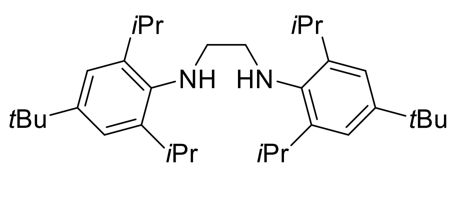

a6

Diamine **a6** was synthesized by the following method, $LiAlH_4$ (1.2 equiv) was suspended in THF (0.2 M) under nitrogen atmosphere, and diimine **a3** was added carefully. The mixture was stirred at ambient temperature for 5 h. Saturated NH_4Cl solution was added, and the mixture was extracted with Et_2O, washed with brine and dried over Na_2SO_4. The crude product obtained after filtration and concentration in vacuo was purified by column chromatography to afford the title product **a6** with almost quantitative yield.

^{1}H NMR (400 MHz, $CDCl_3$) δ 7.14 (s, 4H), 3.38 (hept, *J* = 6.8 Hz, 4H), 3.15 (s, 4H), 1.33 (s, 18H), 1.27 (d, *J* = 6.9 Hz, 24H). **^{13}C NMR** (101 MHz, $CDCl_3$) δ 146.10, 141.88, 140.75, 120.58, 52.43, 34.74, 31.69, 28.07, 24.52. **IR (ATR): $\tilde{\nu}$ (cm^{-1}) =** 2957, 2903, 2867, 1459, 1383, 1362, 1289, 1199, 1099, 878. **HRMS (ESI) (m/z) calcd. for $C_{34}H_{57}N_2^+$:** 493.4516; Found:493.4514. **m. p.:** 135-137 ºC. **R_f:** 0.7 (Pentane:EA 10:1).

<u>1,3-bis(2,6-diisopropylphenyl)-4,5-dihydro-1H-imidazol-3-ium tetrafluoroborate</u>

(**SIPr**·HBF_4):

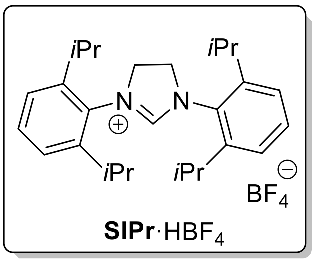

Imidazolium salt **SIPr**·HBF_4 was synthesized according to literature[5, 6].

1,3-bis(2,6-diisopropyl-4-methoxyphenyl)-4,5-dihydro-1H-imidazol-3-ium tetrafluoroborate (**SIPr^{OMe}**·HBF_4):

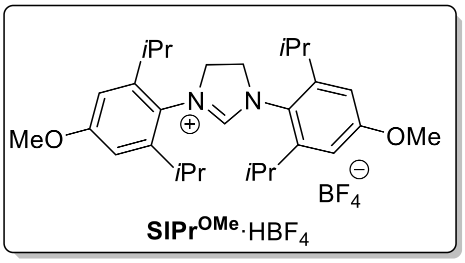

Imidazolium salt **SIPr^{OMe}**·HBF_4 was synthesized according to literature [7, 8].

1,3-bis(4-(*tert*-butyl)-2,6-diisopropylphenyl)-4,5-dimethyl-1H-imidazol-3-ium tetrafluoroborate (**^{Me}IPr^{tBu}**·HBF_4):

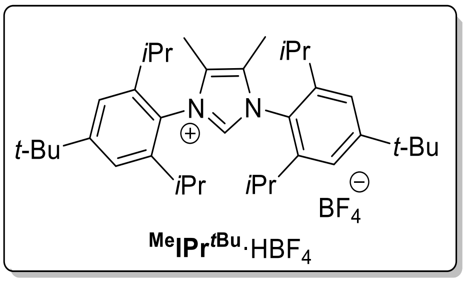

A slightly modified literature procedure[8,9] was used to synthesize **^{Me}IPr^{tBu}**·HBF_4. In a 100 mL three-necked, round bottom flask bisimine **a4** (1800 mg, 3.48 mmol, 1.0 eq) was dissolved in THF (35 mL) and the solution was heated to 70 °C. To this solution $ZnCl_2$ (498.4 mg, 3.66 mmol, 1.05 eq) was added and the mixture was stirred for 5 minutes. A 4M solution of HCl (1.1 mL, 4.35 mmol, 1.25 eq) and paraformaldehyde (115 mg, 3.83 mmol, 1.1 eq) were added successively and rapidly and stirred overnight at 70 °C. The mixture was cooled to ambient temperature and concentrated and the resultant residue was then dissolved in DCM (50mL) and washed with 2M HCl (3x50mL) and brine (1x50mL) and brine of pH = 8 (1 x 50ml), with 2 times back extracting with DCM. The organic solution was dried over $MgSO_4$ and concentrated. The black crude oil was suspended in 40 ml 100/1 v/v water/THF mixture and stirred for 15 mins. Then the mixture was treated with excess HBF_4 (48% w/w in water) at room temperature, resulting a black suspension. After stirring for 24 more hours, the mixture was extracted with DCM (3 x50mL), dried over anhydrous sodium sulfate, and filtered. The filtrate was concentrated before adding pentane, which caused precipitation of the pure imidazolium tetrafluoroborate salt; which was isolated by filtration and dried under vacuum as a pale-yellow solid, yield: 480 mg (22%).

^{1}H NMR (400 MHz, $CDCl_3$) δ 8.37 (s, 1H), 7.35 (s, 4H), 2.34 (hept, J = 6.5 Hz, 4H), 2.20 (s, 6H), 1.38 (s, 18H), 1.30 (d, J = 6.8 Hz, 12H), 1.17 (d, J = 6.9 Hz, 12H). **^{13}C NMR** (101 MHz, $CDCl_3$) δ 155.5, 144.8, 134.2, 130.5, 125.4, 122.0, 35.5, 31.4, 29.2, 25.4, 23.4, 9.3. **^{19}F NMR** (376 MHz, $CDCl_3$) δ -153.7, -153.7. **IR (ATR): $\tilde{\nu}$ (cm^{-1}) =** 2962, 2871, 1602, 1538, 1463,

1391, 1366, 1212, 1055, 915, 880, 732. **HRMS (ESI) (m/z) calcd. for $C_{37}H_{57}N_2^+$:** 529.4516; Found: 529.4521. **m. p.:** decompose before reaching m.p. Crystal structure was obtained by slow evaporation from 1:1 pentane and dichloromethane solution. CCDC: 2122971

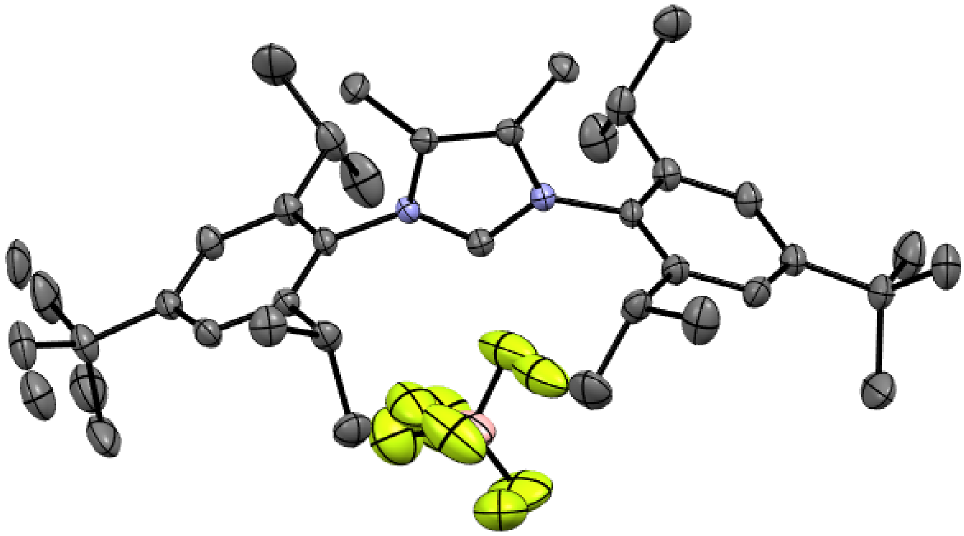

Supplementary Fig 1.1 | ORTEP diagram of $^{Me}IPr^{tBu}\cdot HBF_4$ (thermal ellipsoids are shown at the 50% probability level, for the reason of clarity all the hydrogen atoms are omitted). CCDC 2122971.

1,3-bis(4-(*tert*-butyl)-2,6-diisopropylphenyl)-4,5-dihydro-1H-imidazol-3-ium tetrafluoroborate (**SIPr*tBu***·HBF4):

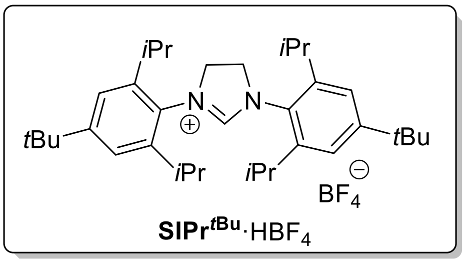

A slightly modified literature procedure[9,10] was used to synthesize **SIPr*tBu***·HBF4. Diamine **a6** (1.88g, 3.82 mmol, 1.0 equiv) and NH_4Cl (306.1 mg, 5.72 mmol, 1.5 equiv) were dissolved in $HC(OEt)_3$ (0.5 M) under nitrogen atmosphere. The mixture was stirred at 115 °C for 20 h and then cooled to room temperature. The crude product was filtered, washed with Et_2O (3 x 10 mL) and dried in vacuo to afford the saturated imidazolium chloride salt as a white solid. The white solid was suspended in 40 ml 100/1 v/v water/THF mixture and stirred for 15 min. Then treated with excess HBF_4 (48% w/ w in water) at room temperature. After the mixture was stirred for a further 24 hours, the mixture was extracted with DCM (3 x50mL), dried over anhydrous sodium sulfate, and filtered. The filtrate was concentrated before adding pentane, which caused precipitation of the pure imidazolium tetrafluoroborate salt; which was isolated by filtration and dried under vacuum as a white solid, yield: (1.20g, 2.02 mmol, 60%).

^{1}H NMR (400 MHz, $CDCl_3$) δ 7.48 (s, 1H), 7.28 (s, 4H), 4.66 (s, 4H), 3.02 (hept, *J* = 6.6 Hz, 4H), 1.42 (d, *J* = 6.7 Hz, 12H), 1.34 (s, 18H), 1.23 (d, *J* = 6.8 Hz, 12H). **^{13}C NMR** (101 MHz, $CDCl_3$) δ 157.6, 155.0, 145.6, 126.7, 122.1, 54.7, 35.4, 31.3, 29.4, 25.6, 24.0. **^{19}F NMR** (376 MHz, $CDCl_3$) δ -153.8, -153.8. **IR (ATR): $\tilde{\nu}$ (cm^{-1}) =** 2965, 1632, 1463, 1266, 1050, 907, 881, 732, 649. **HRMS (ESI) (m/z) calcd. for $C_{35}H_{55}N_2^+$:** 503.4360; Found: 503.4371. **m. p.:**

decompose before reaching m.p. Crystal structure was obtained by slow evaporation from 1:1 pentane and dichloromethane solution. CCDC: 2122972

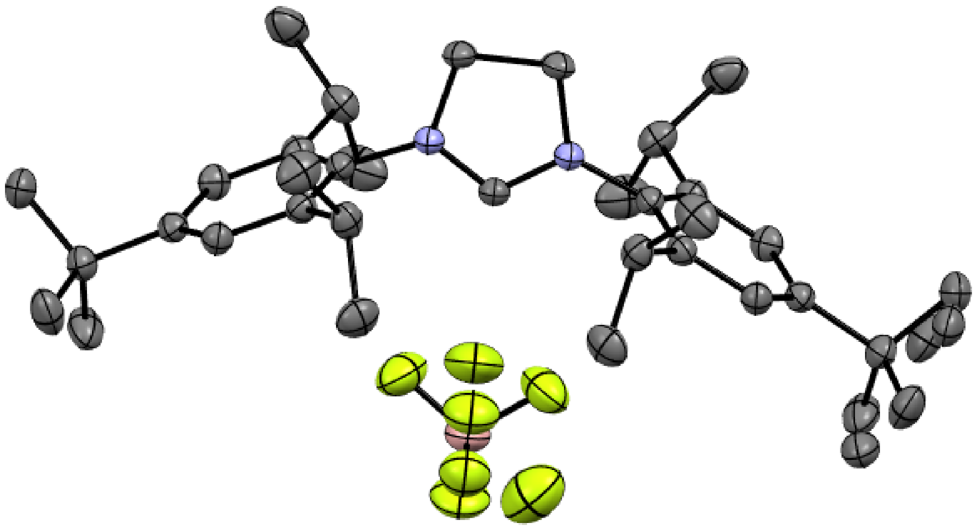

Supplementary Fig 1.2 | ORTEP diagram of $SIPr^{tBu}·HBF_4$ (thermal ellipsoids are shown at the 50% probability level, for the reason of clarity all the hydrogen atoms are omitted). CCDC 2122972.

<u>1,3-bis(2,6-dibenzhydryl-4-(tert-butyl)phenyl)-4,5-dimethyl-1H-imidazol-3-ium tetrafluoroborate (**^{Me}IPr*^{tBu}**·HBF_4):</u>

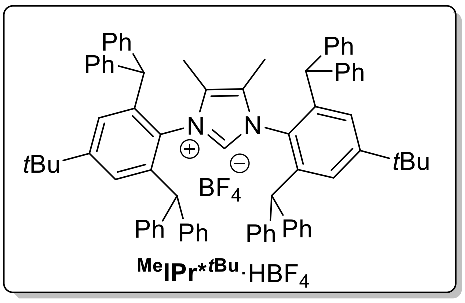

A slightly modified literature procedure [8, 9] was used to synthesize **^{Me}IPr*^{tBu}**·HBF_4. In a 100mL three-necked, roundbottom flask bisimine **a5** (2100 mg, 2.07 mmol, 1.0 eq) was dissolved in THF (20 mL) and the solution was heated to 70 °C. To this solution $ZnCl_2$ (296.6 mg, 2.18 mmol, 1.05 eq) was added and the mixture was stirred for 5 minutes. A 4 M solution of HCl (0.65 mL, 2.59 mmol, 1.25 eq) and paraformaldehyde (68.5 mg, 2.28 mmol, 1.1 eq) were added succesively and rapidly and stirred overnight at 70 °C. The mixture was cooled to ambient temperature and concentrated and the resultant residue was then dissolved in DCM (40 mL) and washed with 2M HCl (3x40mL) and brine (1x40 mL) and brine of pH=8 (1x40 ml), with 2 times back extracting with DCM. The organic solution was dried over $MgSO_4$ and concentrated. The black crude oil was suspended in 40 ml 100/1 v/v water/THF mixture and stirred for 15 min. Then treated with excess HBF_4 (48% w/ w in water) at room temperature, which resulted in the formation of a black suspension. After the mixture was stirred for a further 24 hours, the mixture was extracted with DCM (3 x50mL), dried over anhydrous sodium sulfate, and filtered. The filtrate was concentrated before adding pentane, which caused precipitation of the pure imidazolium tetrafluoroborate salt; which was isolated by filtration and dried under vacuum as a pale-yellow solid, yield: 1300 mg (57%).

^{1}H NMR (400 MHz, $CDCl_3$) δ 9.62 (s, 1H), 7.40 – 7.30 (m, 14H), 7.22 (m, 8H), 7.11 (m, 5H), 7.06 – 6.97 (m, 17H), 5.00 (s, 4H), 1.19 (s, 18H), 0.87 (s, 6H). **^{13}C NMR** (101 MHz, $CDCl_3$) δ 154.6, 141.8, 141.2, 140.6, 136.4, 130.6, 129.8, 129.3, 128.9, 128.8, 128.3, 127.8, 127.5, 127.2, 51.9, 35.2, 31.0, 7.8. **^{19}F NMR** (376 MHz, $CDCl_3$) δ -151.5, -151.5. **IR (ATR): $\tilde{\nu}$ (cm^{-1})** = 2964, 1599, 1494, 1478, 1447, 1196, 1056, 1030, 1002, 909, 763, 729, 699, 646, 621, 606, 587. **HRMS (ESI) (m/z) calcd. for $C_{77}H_{73}N_2^+$:** 1025.5768; Found: 1025.5726. **m. p.:** decompose before reaching m.p. Crystal structure was obtained by slow evaporation from 1:1 pentane and dichloromethane solution. CCDC: 2122973.

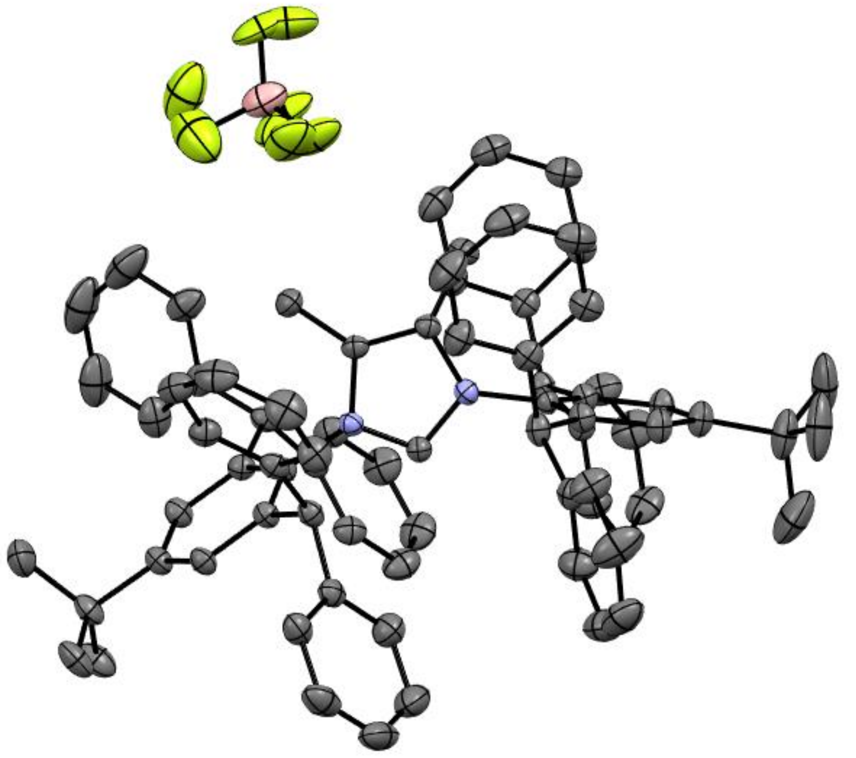

Supplementary Fig 1.3 | ORTEP diagram of ^{Me}IPr*^{tBu}·HBF_4 (thermal ellipsoids are shown at the 50% probability level, for the reason of clarity all the hydrogen atoms are omitted). CCDC 2122973.

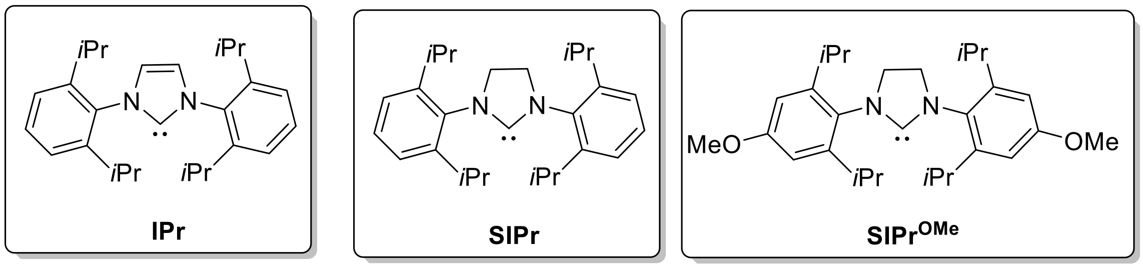

Free carbene **IPr**, **SIPr** and **SIPr^{OMe}** were synthesized according to the literature [9, 10].

General procedure 1 (GP1):

Inside the glovebox, the tetrafluoroborate salt was suspended in THF (0.05 M) and NaH (4 equiv) was added in plus catalytic amount (spatula tip) of KO*t*Bu in a Schlenk tube. The mixture was taken outside the glovebox and sonicated at room temperature for 4 hours, then the mixture was brought back inside the glovebox. After removal of the solid by-products by filtration through a pad of celite on a frit. The solution was concentrated directly afforded the desired carbenes.

<u>1,3-bis(4-(tert-butyl)-2,6-diisopropylphenyl)-4,5-dimethyl-1H-imidazol-3-ium-2-ide (**^{Me}IPr^{tBu}**):</u>

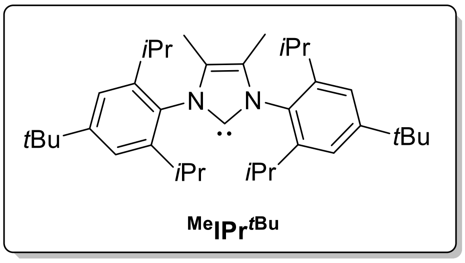

Follow the **<u>GP1</u>**, using **^{Me}IPr*^{tBu}**·HBF_4 (100 mg, 0.16 mmol) NaH (15.6 mg, 0.65 mmol,) giving free carbene **^{Me}IPr^{tBu}** (82 mg, 93% yield).
^{1}H NMR (400 MHz, C_6D_6) δ 7.47 (s, 4H), 3.00 (hept, *J* = 6.9 Hz, 4H), 1.68 (s, 6H), 1.40 (d, *J* = 6.8 Hz, 12H), 1.37 (s, 18H), 1.31 (d, *J* = 7.0 Hz, 12H). **^{13}C NMR** (101 MHz, C_6D_6) δ 217.4, 151.1, 146.1, 135.3, 124.0, 120.5, 35.1, 31.7, 29.1, 25.5, 23.2, 9.8.

<u>1,3-bis(4-(tert-butyl)-2,6-diisopropylphenyl)-4,5-dihydro-1H-imidazol-3-ium-2-ide (**SIPr^{tBu}**):</u>

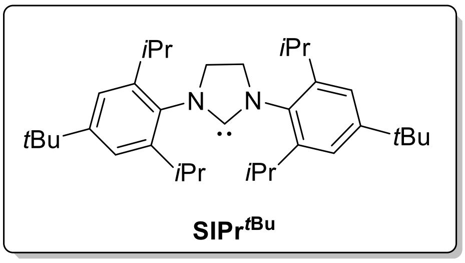

Inside the glovebox, the tetrafluoroborate salt **SIPr^{tBu}**·HBF_4 (100 mg, 0.17 mmol, 1 equiv) was suspended in THF (0.05 M) and NaH (16.2 mg, 4 equiv) was added in. The mixture was taken outside the glovebox and sonicated at room temperature for 4 hours, then the mixture was brought back inside the glovebox. After removal of the solid by-products by filtration through a pad of Celite on a frit. The solution was concentrated then the solid is washed with pentane to afford desired carbene **<u>SIPr^{tBu}</u>** (42.0 mg, 0.083 mmol, 49% yield).
^{1}H NMR (400 MHz, C_6D_6) δ 7.42 (s, 4H), 3.41 – 3.31 (m, 8H), 1.45 – 1.35 (m, 42H). **^{13}C NMR** (101 MHz, C_6D_6) δ 244.3, 150.4, 146.7, 137.2, 120.8, 67.8, 53.8, 35.0, 31.7, 29.2, 25.6, 23.8.

<u>1,3-bis(2,6-dibenzhydryl-4-(tert-butyl)phenyl)-4,5-dimethyl-1H-imidazol-3-ium-2-ide (**^{Me}IPr*^{tBu}**):</u>

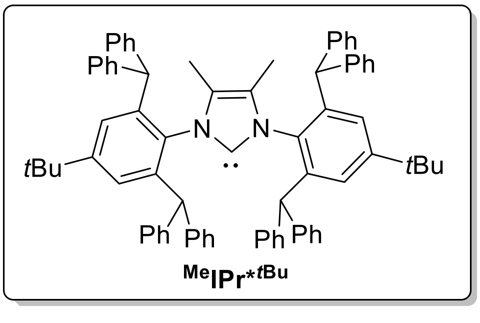

Follow the **<u>GP1</u>**, using **^{Me}IPr*^{tBu}**·HBF_4 (200 mg, 0.180 mmol) NaH (17.2 mg, 0.72 mmol,) giving free carbene **^{Me}IPr*^{tBu}** (150 mg 0.146 mmol, 81% yield).
^{1}H NMR (400 MHz, C_6D_6) δ 7.54 (s, 4H), 7.49 (d, *J* = 7.4 Hz, 8H), 7.32 (d, *J* = 7.2 Hz, 8H), 7.07 – 6.95 (m, 20H), 6.94 – 6.88 (m, 4H), 6.22 (s, 4H), 1.13 (s, 18H), 0.82 (s, 6H). **^{13}C NMR** (101 MHz, C_6D_6) δ 216.8, 150.7, 145.8, 142.9, 142.1, 137.2, 130.3, 130.0, 128.5, 127.1, 126.9, 126.6, 126.4, 51.6, 34.9, 31.2, 8.6.

2.2 Chiral ligand synthesis.

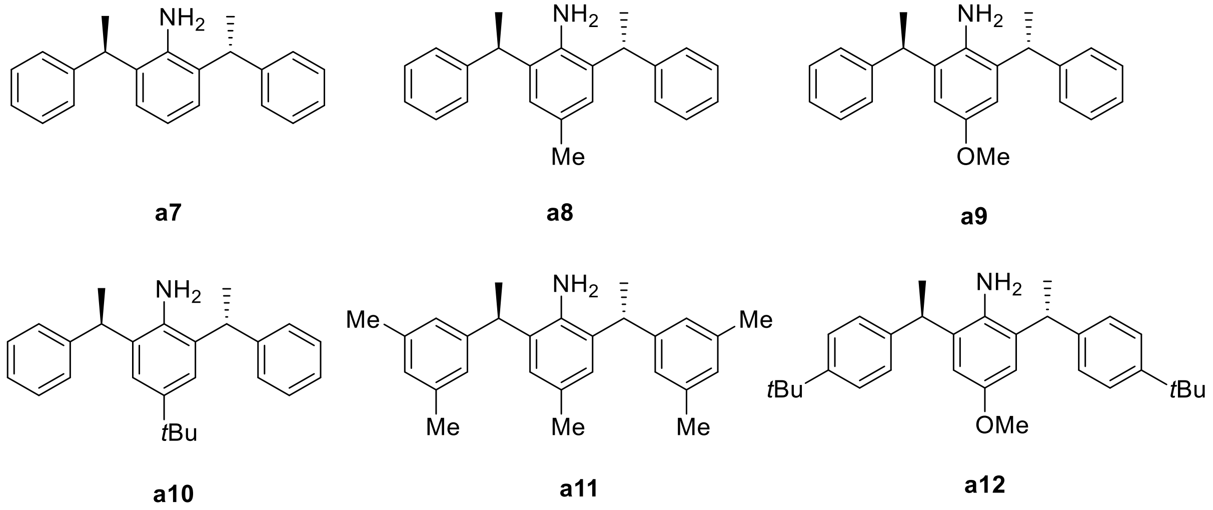

Supplementary Fig 2.1 | General overview of applied chiral anilines.

Chiral anilines **a7** to **a11** were synthesized according to literature[11, 12, 13]. Chiral aniline **a12** and diene precursor **a13** were synthesized using a slightly modified literature procedure[13].

<u>2,6-bis(1-(4-(tert-butyl)phenyl)vinyl)-4-methoxyaniline (**a13**):</u>

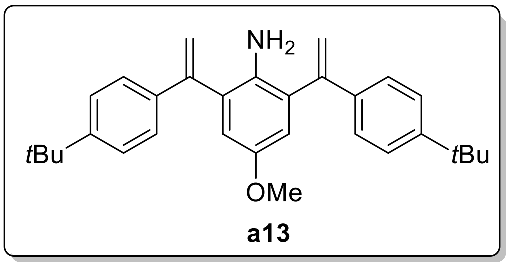

A 25 mL thick wall microwave tube equipped with a stirring bar was charged with p-anisidine (1.23 g, 10.0 mmol), KSF montmorillonite (1.0 g), and 4-*t*Bu-phenyl acetylene (3.8 mL, 21.0 mmol) under N_2 atmosphere. The tube was heated to 150 °C overnight with vigorous stirring. The reaction vessel was allowed to cool to room temperature before dilution with ethyl acetate and filtration. The solvent was removed from the mother liquor under reduced pressure, and the resultant green oil was purified via column chromatography with 50:50 dichloromethane and hexane, giving desired diene precursor **a13** as a pale-yellow solid (2.8137 g, 6.4 mmol, 64% yield).

^{1}H NMR (400 MHz, C_6D_6) δ 7.43 (d, *J* = 8.5 Hz, 4H), 7.21 (d, *J* = 8.5 Hz, 4H), 7.01 (s, 2H), 5.71 (d, *J* = 1.6 Hz, 2H), 5.34 (d, *J* = 1.6 Hz, 2H), 3.37 (s, 3H), 3.29 (s, 2H), 1.19 (s, 18H). **^{13}C NMR** (101 MHz, C_6D_6) δ 152.5, 151.1, 147.8, 137.2, 136.3, 126.9, 125.8, 116.4, 115.3, 55.3, 34.5, 31.4. **IR (ATR): $\tilde{\nu}$ (cm^{-1}) =** 3461, 3378, 3085, 3031, 2960, 2904, 2867, 2832, 1685, 1591, 1510, 1459, 1436, 1405, 1394, 1363, 1345, 1292, 1270, 1235, 1218, 1139, 1108, 1050, 1015, 940, 901, 877, 842, 811, 786, 756, 597, 566, 535, 499. **HRMS (ESI) (m/z) calcd. for $C_{31}H_{38}NO^+$:** 440.2948; Found: 440.2942. **m. p.:** 69-71 °C. **R_f:** 0.5 (Pentane:DCM 1:1).

<u>2,6-bis((*R*)-1-(4-(tert-butyl)phenyl)ethyl)-4-methoxyaniline (**a12**):</u>

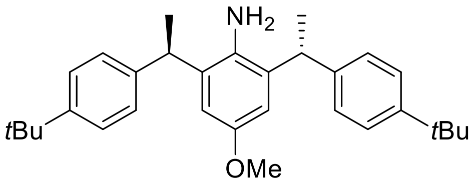

a12

In a microwave tube, $[Rh(nbd)_2]BF_4$ (2.5 mol%) and (1R,1'R,2S,2'S)-DuanPhos (3.5 mol%) were dissolved in DCM (1 ml) under a nitrogen atmosphere and stirred for 20 min. Subsequently a solution of alkenyl aniline **a13** (1 eq) in a minimal amount of DCM was added. Subsequently MeOH (ratio MeOH/DCM: >7/1, 0.2 M) was added. The microwave tube was transferred into the autoclave and the reactor was purged three times with H_2. The reactor was pressurized to 50 bar H_2 and the mixture was stirred at ambient temperature for 48h. The reaction mixture was concentrated and purified by silica gel column (DCM:pentane, 1:4). Chiral aniline **a12** was obtained in 90% yield as colourless oil.

^{1}H NMR (400 MHz, C_6D_6) δ 7.19 (d, J = 8.4 Hz, 4H), 7.11 – 7.04 (m, 6H), 3.85 (q, J = 7.1 Hz, 2H), 3.60 (s, 3H), 2.92 (s, 2H), 1.49 (d, J = 7.0 Hz, 6H), 1.19 (s, 18H). **^{13}C NMR** (101 MHz, C_6D_6) δ 153.3, 149.0, 143.3, 136.6, 131.9, 127.6, 125.8, 111.7, 55.4, 40.4, 34.4, 31.5, 22.6. **IR (ATR): $\tilde{\nu}$ (cm^{-1}) =** 3464, 3372, 3051, 2961, 2903, 2869, 2830, 2280, 1601, 1507, 1463, 1435, 1405, 1363, 1330, 1288, 1268, 1224, 1201, 1150, 1120, 1109, 1082, 1058, 1038, 1017, 997, 930, 869, 832, 813, 790, 759, 747, 667, 645, 618, 574, 516, 498. **HRMS (ESI) (m/z) calcd. for $C_{31}H_{42}NO^+$:** 444.3261; Found: 444.3252. **R_f:** 0.5 (Pentane:DCM 1:1). **$[\alpha]_D^{20}$:** 44° (c = 1, CH_2Cl_2).

Chiral HPLC: SFC, Chiralpak IB; CO_2/MeOH = 99:1, 2 mL/min, 250 nm; t_R (major) = 6.7 min, t_R (minor) = 10.2 min, >99:1 er.

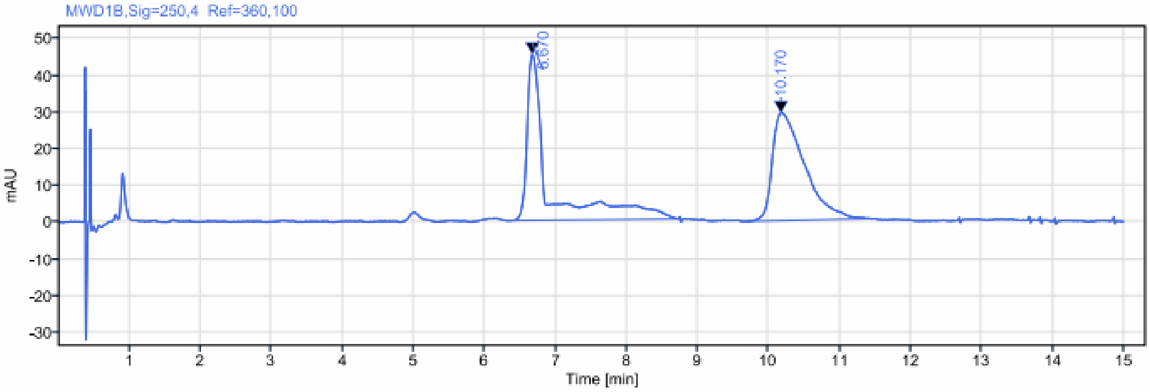

Signal: MWD1B,Sig=250,4 Ref=360,100

RT [min]	Type	Width [min]	Area	Height	Area%	Name
6.670	MM m	2.33	911.30	45.19	49.78	
10.170	MM m	1.74	919.51	29.29	50.22	
		**Sum**	**1830.81**			

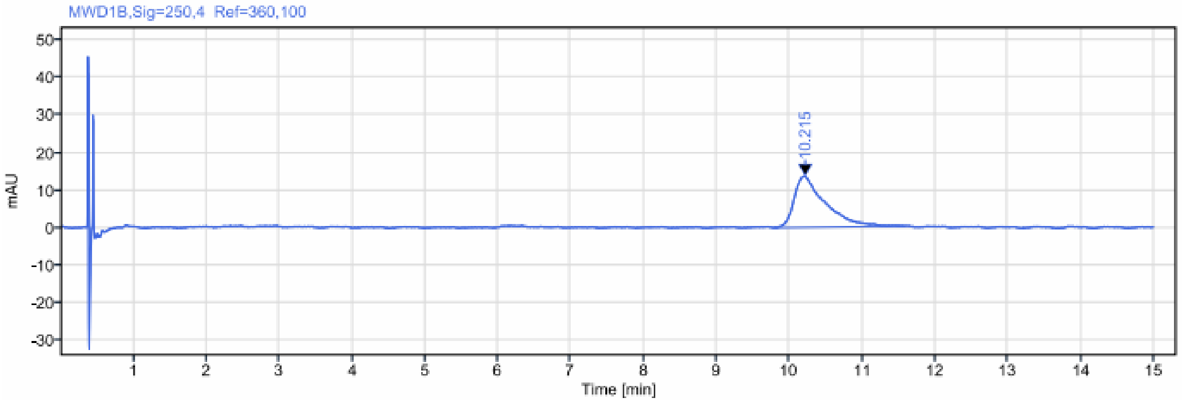

Signal: MWD1B,Sig=250,4 Ref=360,100

RT [min]	Type	Width [min]	Area	Height	Area%	Name
10.215	MM m	1.80	403.60	13.69	100.00	
		**Sum**	**403.60**			

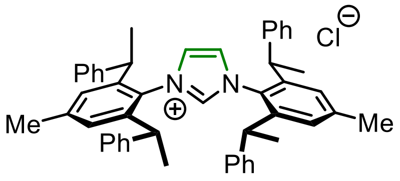

L1·HCl

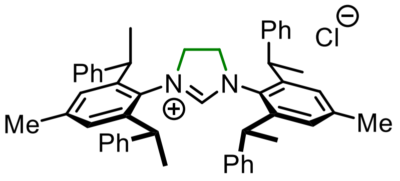

L2·HCl

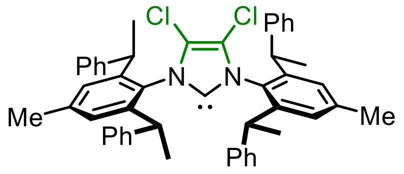

L3

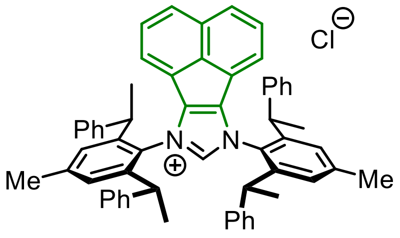

L4·HCl

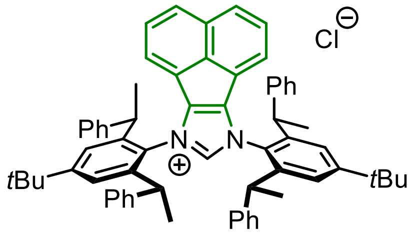

L5·HCl

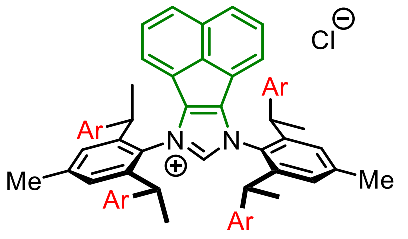

Ar = 3,5-Xyl

L6·HCl

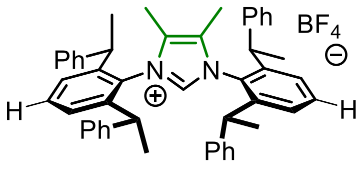

L7·HBF_4

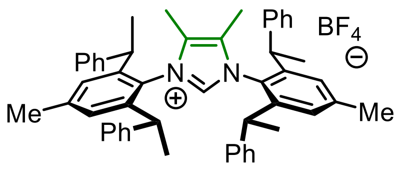

L8·HBF_4

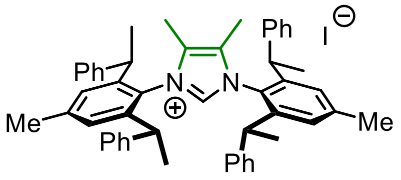

L8·HI

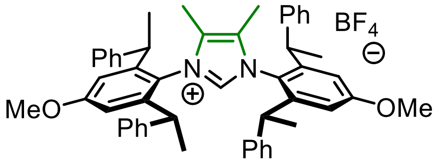

L9·HBF_4

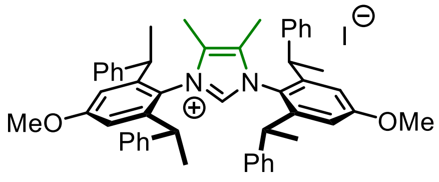

L9·HI

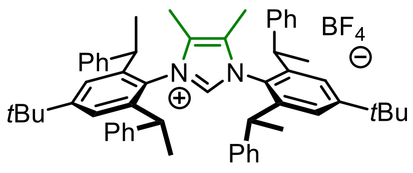

L10·HBF_4

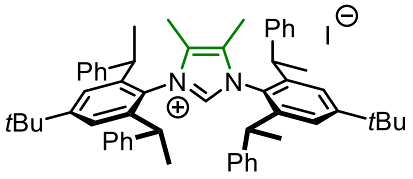

L10·HI

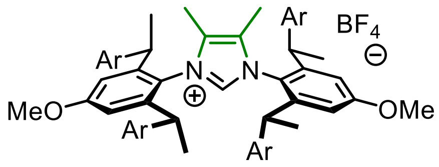

Ar = 4-*t*Bu-phenyl

L11·HBF_4

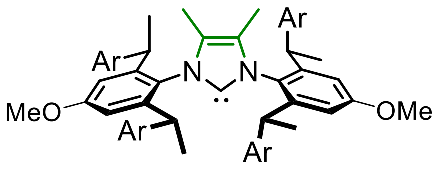

Ar = 4-*t*Bu-phenyl

L11

Supplementary Fig 2.2 | General overview of synthesized chiral carbene ligands.

Chiral carbene precursor and **L1**·HCl, **L2**·HCl, **L3**, **L4**·HCl, **L5**·HCl, **L6**·HCl and **L8**·HI were synthesized according to the literature[11, 12, 13]. Chiral carbene precursor **L7**·HBF_4, **L8**·HBF_4,

L9·HBF_4, **L9**·HI, **L10**·HBF_4, **L10**·HI, **L11**·HBF_4 and **L11** were synthesized using a slightly modified literature procedure[13].

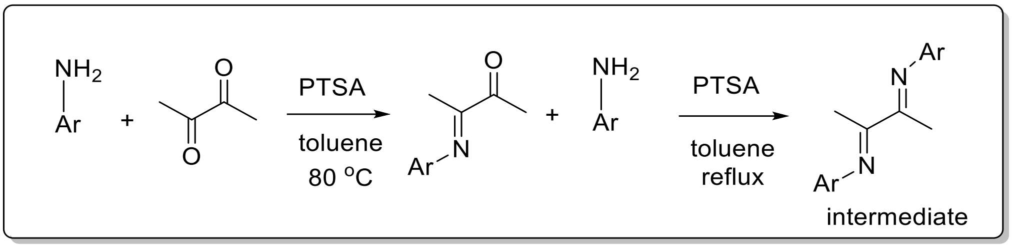

To a solution of 2,3-butanedione (1.00 equiv.) in toluene (0.20 M) were added aniline (2.10 equiv.) and p-toluenesulfonic acid monohydrate (5.00 mol%). The mixture was heated to 80 °C overnight, then refluxed for 48 hours (monitoring by TLC), then concentrated under reduced pressure to afford mixture of products. The mixture was washed with MeOH, obtaining the crude bisimine as a yellow solid after drying under vacuum. The crude bisimine can be directly submitted to next step without any purification.

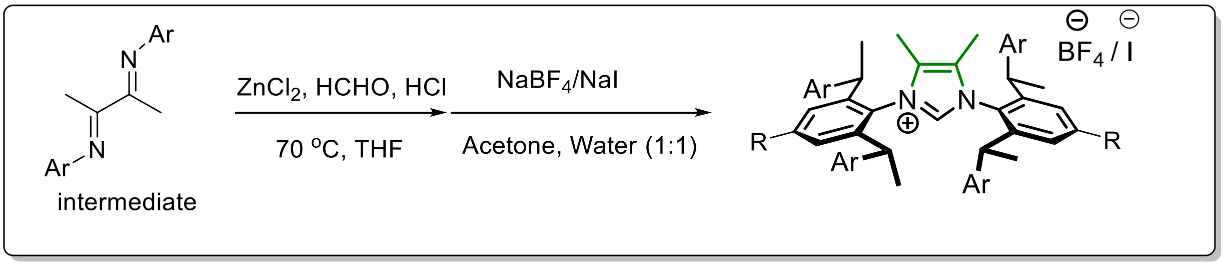

In a three-necked, round bottom flask bisimine (1.0 eq) was dissolved in THF (0.5 M) and the solution was heated to 70 °C. To this solution $ZnCl_2$ (1.05 eq) was added and the mixture was stirred for 5 minutes. 2 M solution of HCl in diethyl ether (1.25 eq) and paraformaldehyde (1.3 eq) were added succesively and rapidly and stirred over night at 70 °C. The mixture was cooled to ambient temperature and concentrated and the resultant residue was then dissolved in DCM (50 mL) and washed with 2 M HCl (3 x 50mL) and brine (1 x 50mL) and brine of pH = 8 (1 x 50ml), with 2 times back-extracting with DCM. The organic solution was dried over $MgSO_4$ and concentrated. The black crude oil was suspended in water and acetone (1:1) was added sodium iodide or sodium tetrafluoroborate (5 eq). The mixture was stirred for 4h at rt (sonicator to achieve homogenous suspension). The black mixture was extracted with DCM and dried over $MgSO_4$. The solution was then concentrated under reduced pressure, the mixture was then added dropwise to diethyl ether or pentane with stirring. The carbene salts formed as solid, were then collected by simple filtration.

1,3-bis(2,6-bis((*R*)-1-phenylethyl)phenyl)-4,5-dimethyl-1H-imidazol-3-ium tetrafluoroborate (**L7**·HBF_4):

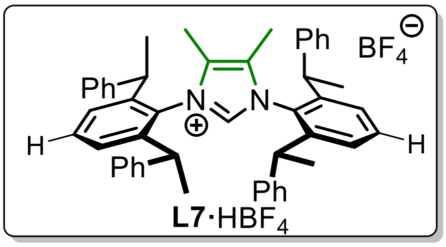

Carbene precursor **L7**·HBF4 was obtained in 32% yield over 2 steps as a grey solid. **^{1}H NMR** (400 MHz, $CDCl_3$) δ 9.19 (s, 1H), 7.68 – 7.55 (m, 4H), 7.43 – 7.36 (m, 4H), 7.31 (dd, *J* = 7.8, 1.5 Hz, 2H), 7.23 – 7.12 (m, 12H), 6.72 – 6.61 (m, 4H), 3.71 (q, *J* = 7.0 Hz, 2H), 3.25 (q, *J* = 7.0 Hz, 2H), 1.74 (d, *J* = 7.2 Hz, 6H), 1.31 – 1.11 (m, 12H). **^{13}C NMR** (101 MHz, $CDCl_3$) δ 144.6, 143.5, 142.7, 142.1, 136.4, 132.3, 130.0, 129.6, 129.5, 128.6, 127.7, 127.4, 127.2, 127.2, 127.1, 126.8, 40.4, 38.1, 22.9, 21.1, 7.9. **^{19}F NMR** (376 MHz, $CDCl_3$) δ -152.2. **IR (ATR): $\tilde{\nu}$ (cm^{-1}) =** 3027, 2973, 2934, 1599, 1529, 1494, 1448, 1378, 1330, 1283, 1229, 1205, 1056, 1027, 910, 880, 818, 761, 729, 700, 678, 647, 572, 520. **HRMS (ESI) (m/z) calcd. for $C_{49}H_{49}N_2^+$:** 665.3890; Found: 665.3887. **m. p.:** decompose before reaching m.p. $[\alpha]_D^{20}$**:** -200.0 ° (c = 0.1, $CHCl_3$).

<u>1,3-bis(4-methyl-2,6-bis((*R*)-1-phenylethyl)phenyl)-4,5-dimethyl-1H-imidazol-3-ium tetrafluoroborate (**L8**·HBF4):</u>

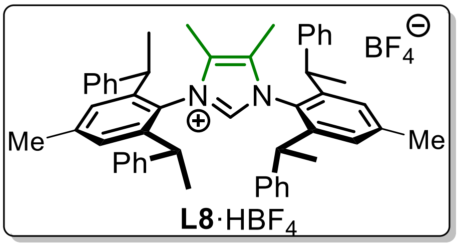

Carbene precursor **L8**·HBF4 was obtained in 45% yield over 2 steps as a grey solid. **^{1}H NMR** (400 MHz, $CDCl_3$) ^{1}H NMR (400 MHz, $CDCl_3$) δ 9.05 (s, 1H), 7.41 (dd, *J* = 8.7, 7.1 Hz, 4H), 7.34 (d, *J* = 2.1 Hz, 2H), 7.23 – 7.07 (m, 14H), δ 6.69 – 6.61 (m, 4H), 3.65 (q, *J* = 7.0 Hz, 2H), 3.20 (q, *J* = 7.1 Hz, 2H), 2.44 (s, 6H), 1.72 (d, *J* = 7.2 Hz, 6H), 1.26 – 1.12 (m, 12H). **^{13}C NMR** (101 MHz, $CDCl_3$) δ 144.7, 143.0, 142.9, 142.5, 141.7, 136.5, 129.6, 129.5, 128.6, 128.2, 128.0, 127.6, 127.4, 127.2, 127.1, 126.8, 40.2, 38.0, 22.9, 22.0, 21.1, 7.9. **^{19}F NMR** (376 MHz, $CDCl_3$) δ -152.5, -152.5. **IR (ATR): $\tilde{\nu}$ (cm^{-1})** = 3108, 3058, 3028, 2973, 2935, 2877, 1628, 1603, 1531, 1494, 1461, 1448, 1380, 1328, 1267, 1219, 1158, 1058, 1031, 912, 865, 798, 758, 732, 701, 684, 647, 605, 574, 552, 519. **HRMS (ESI) (m/z) calcd. for $C_{51}H_{53}N_2^+$**: 693.4203; Found: 693.4206. m. p.: decompose before reaching m.p. $[\alpha]_D^{20}$**:** 146.50° (c = 1, $CHCl_3$).

<u>1,3-bis(4-methoxy-2,6-bis((*R*)-1-phenylethyl)phenyl)-4,5-dimethyl-1H-imidazol-3-ium tetrafluoroborate (**L9**·HBF4):</u>

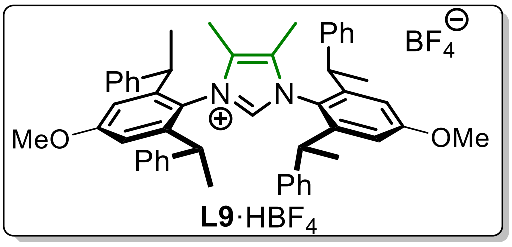

Carbene precursor **L9**·HBF4 was obtained in 42% yield over 2 steps as a brown solid. ^{1}H NMR (400 MHz, $CDCl_3$) δ 9.02 (s, 1H), 7.44 – 7.36 (m, 4H), 7.24 – 7.08 (m, 12H), 7.05 (d, *J* = 2.8 Hz, 2H), 6.75 (d, *J* = 2.9 Hz, 2H), 6.68 – 6.62 (m, 4H).3.83 (s, 6H), 3.65 (q, *J* = 6.9 Hz, 2H), 3.18 (q, *J* = 7.0 Hz, 2H), 1.72 (d, *J* = 7.0 Hz, 6H), 1.24 – 1.10 (m, 12H). ^{13}C NMR (101 MHz, $CDCl_3$) δ 161.9, 145.0, 144.4, 143.7, 142.7, 137.0, 129.8, 129.5, 128.6, 127.3, 127.1, 126.9, 122.8, 113.4, 112.0, 55.7, 40.4, 38.3, 22.8, 21.1, 7.8. ^{19}F NMR (376 MHz, $CDCl_3$) δ -152.2, -152.3. **IR (ATR): $\tilde{\nu}$ (cm^{-1}) =** 3107, 3060, 3027, 2972, 2936, 2842, 2262, 1599, 1531, 1494, 1465, 1449, 1390, 1379, 1341, 1326, 1266, 1216, 1158, 1055, 1026, 910, 872, 856, 796,

756, 728, 700, 647, 571, 547, 519. **HRMS (ESI) (m/z) calcd. for $C_{51}H_{53}N_2O_2^+$**: 725.4102; Found: 725.4104. **m. p.**: decompose before reaching m.p. $[\alpha]_D^{20}$**:** -20.22 ° (c = 1, $CHCl_3$).

1,3-bis(4-methoxy-2,6-bis((*R*)-1-phenylethyl)phenyl)-4,5-dimethyl-1H-imidazol-3-ium iodide (**L9**·HI):

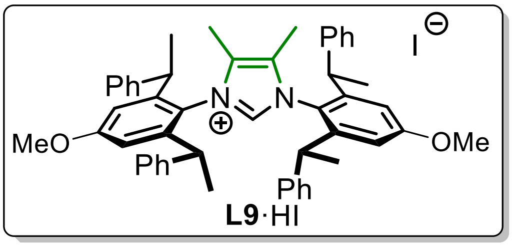

Carbene precursor **L9**·HI was obtained in 40% yield over 2 steps as a pale-yellow solid. **^{1}H NMR** (400 MHz, $CDCl_3$) δ 10.53 (s, 1H), 7.46 (t, *J* = 7.8 Hz, 4H), 7.37 (d, *J* = 7.8 Hz, 4H), 7.22 (t, *J* = 7.3 Hz, 2H), 7.13 (d, *J* = 7.5 Hz, 6H), 7.03 (d, *J* = 2.9 Hz, 2H), 6.73 (d, *J* = 2.9 Hz, 2H), 6.62 (d, *J* = 6.0 Hz, 4H), 3.83 (s, 6H), 3.67 (q, *J* = 7.0 Hz, 2H), 3.32 (q, *J* = 7.0 Hz, 2H), 1.72 (d, *J* = 7.2 Hz, 6H), 1.26 (d, *J* = 7.0 Hz, 6H), 1.17 (s, 6H). **^{13}C NMR** (101 MHz, $CDCl_3$) δ 161.9, 145.2, 144.5, 143.7, 142.4, 137.5, 129.7, 129.4, 128.6, 127.6, 127.4, 127.0, 126.8, 122.7, 113.6, 112.0, 55.7, 40.5, 38.2, 23.2, 21.0, 8.0. **IR (ATR): $\tilde{\nu}$ (cm^{-1})** = 3057, 3026, 2971, 2933, 1600, 1530, 1493, 1466, 1448, 1378, 1340 1327, 1266, 1218, 1159, 1089, 1038, 1027, 920, 872, 856, 755, 731, 701, 640, 571. **HRMS (ESI) (m/z) calcd. for $C_{51}H_{53}N_2O_2^+$:** 725.4102; Found: 725.4102. **m. p.:** decompose before reaching m.p. $[\alpha]_D^{20}$**:** 140.0 ° (c = 0.1, $CHCl_3$).

1,3-bis(4-(tert-butyl)-2,6-bis((*R*)-1-phenylethyl)phenyl)-4,5-dimethyl-1H-imidazol-3-ium tetrafluoroborate (**L10**·HBF_4):

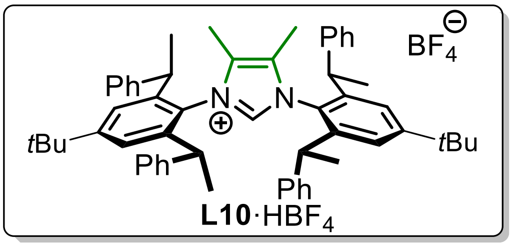

Carbene precursor **L10**·HBF_4 was obtained in 35% yield over 2 steps as a grey solid. ^{1}H NMR (400 MHz, $CDCl_3$) δ 8.60 (s, 1H), 7.57 (d, *J* = 2.3 Hz, 2H), 7.42 – 7.32 (m, 6H), 7.23 – 7.10 (m, 12H), 6.72 – 6.64 (m, 4H), 3.71 (q, *J* = 7.0 Hz, 2H), 3.14 (q, *J* = 7.0 Hz, 2H), 1.74 (d, *J* = 7.0 Hz, 6H), 1.36 (s, 18H), 1.23 (d, *J* = 7.0 Hz, 6H), 1.17 (s, 6H). ^{13}C NMR (101 MHz, $CDCl_3$) δ 155.4, 144.6, 143.2, 142.4, 141.3, 135.5, 130.0, 129.4, 128.8, 127.5, 127.3, 127.1, 127.1, 126.9, 124.5, 124.3, 40.5, 38.5, 35.5, 31.3, 23.2, 21.6, 7.9. ^{19}F NMR (376 MHz, $CDCl_3$) δ -152.7, -152.7. **IR (ATR): $\tilde{\nu}$ (cm^{-1}) =** 3108, 3059, 3028, 2966, 2906, 2874, 1628, 1601, 1532, 1494, 1477, 1448, 1419, 1393, 1365, 1329, 1281, 1227, 1204, 1054, 1028, 910, 882, 758, 728, 700, 647, 574, 553, 520. **HRMS (ESI) (m/z) calcd. for $C_{57}H_{65}N_2^+$:** 777.5142; Found: 777.5146. **m. p.:** decompose before reaching m.p. $[\alpha]_D^{20}$**:** -18.23 ° (c = 1, $CHCl_3$).

1,3-bis(4-(tert-butyl)-2,6-bis((*R*)-1-phenylethyl)phenyl)-4,5-dimethyl-1H-imidazol-3-ium iodide (**L10'**·HI):

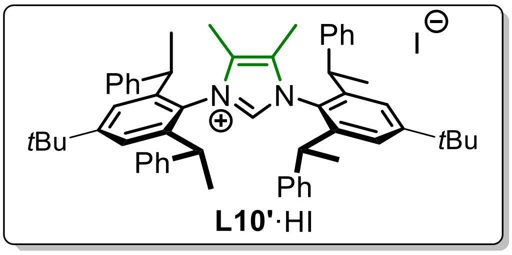

Carbene precursor **L10'**·HI was obtained in 31% yield over 2 steps as a pale-yellow solid. **^{1}H NMR** (600 MHz, CDCl$_3$) δ 9.51 (s, 1H), 7.56 (d, *J* = 2.2 Hz, 2H), 7.42 – 7.38 (m, 4H), 7.33 (d, *J* = 2.1 Hz, 2H), 7.26 – 7.17 (m, 10H), 7.14 (t, *J* = 7.3 Hz, 2H), 6.66 (d, *J* = 7.1 Hz, 4H), 3.72 (q, *J* = 7.0 Hz, 2H), 3.21 (q, *J* = 7.0 Hz, 2H), 1.75 (d, *J* = 7.2 Hz, 6H), 1.36 (s, 18H), 1.25 (d, *J* = 7.0 Hz, 6H), 1.17 (s, 6H). **^{13}C NMR** (151 MHz, CDCl$_3$) δ 155.4, 144.5, 143.0, 142.5, 141.3, 135.6, 130.0, 129.5, 128.8, 127.4, 127.4, 127.3, 127.0, 126.9, 124.5, 124.4, 40.6, 38.4, 35.5, 31.3, 23.4, 21.6, 8.2. **IR (ATR): $\tilde{\nu}$ (cm^{-1})** = 2966, 2926, 1600, 1529, 1494, 1476, 1449, 1365, 1204, 1030, 884, 789, 757, 736, 728, 700, 670, 660, 649, 594, 575, 526, 506, 492, 478, 459, 451, 414. **HRMS (ESI) (m/z) calcd. for $C_{57}H_{65}N_2^+$:** 777.5142; Found: 777.5154. **m. p.:** decompose before reaching m.p. $[\alpha]_D^{20}$**:** 133.3 ° (c = 0.1, CHCl$_3$).

<u>1,3-bis(2,6-bis((*R*)-1-(4-(tert-butyl)phenyl)ethyl)-4-methoxyphenyl)-4,5-dimethyl-1H-imidazol-3-ium tetrafluoroborate (**L11**·HBF$_4$):</u>

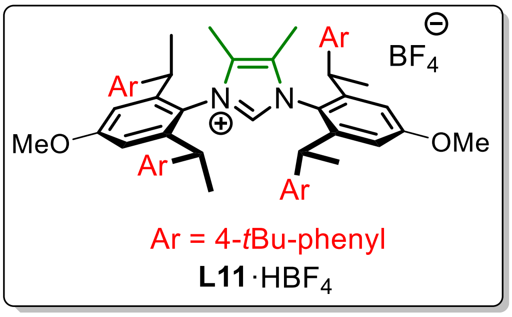

Carbene precursor **L11**·HBF$_4$ was obtained in 41% yield over 2 steps as a reddish brown solid. **^{1}H NMR** (400 MHz, CDCl$_3$) δ 8.82 (s, 1H), 7.41 (d, *J* = 8.3 Hz, 4H), 7.18 (d, *J* = 8.3 Hz, 4H), 7.10 (d, *J* = 8.2 Hz, 4H), 7.02 (d, *J* = 2.8 Hz, 2H), 6.78 (d, *J* = 2.8 Hz, 2H), 6.64 (d, *J* = 8.4 Hz, 4H), 3.84 (s, 6H), 3.64 (q, *J* = 7.1 Hz, 2H), 3.17 (q, *J* = 6.8 Hz, 2H), 1.72 (d, *J* = 7.2 Hz, 6H), 1.26 (d, *J* = 5.9 Hz, 36H), 1.18 – 1.12 (m, 12H). **^{13}C NMR** (101 MHz, CDCl$_3$) δ 161.9, 150.0, 149.9, 145.1, 144.1, 141.2, 139.7, 129.9, 127.2, 126.7, 126.4, 125.4, 122.7, 113.3, 112.0, 55.8, 40.0, 37.9, 34.5, 31.4, 22.8, 21.3, 7.7. **^{19}F NMR** (376 MHz, CDCl$_3$) δ -152.6. **IR (ATR): $\tilde{\nu}$ (cm^{-1})** = 2962, 2905, 2869, 1601, 1510, 1465, 1407, 1394, 1364, 1332, 1269, 1217, 1160, 1110, 1059, 1035, 912, 872, 834, 731, 574. **HRMS (ESI) (m/z) calcd. for $C_{57}H_{65}N_2^+$:** 949.6606; Found: 949.6616. **m. p.:** decompose before reaching m.p. $[\alpha]_D^{20}$**:** -171.0 ° (c = 0.1, CHCl$_3$).

<u>1,3-bis(2,6-bis((*R*)-1-(4-(tert-butyl)phenyl)ethyl)-4-methoxyphenyl)-4,5-dimethyl-1H-imidazol-3-ium-2-ide (**L11**):</u>

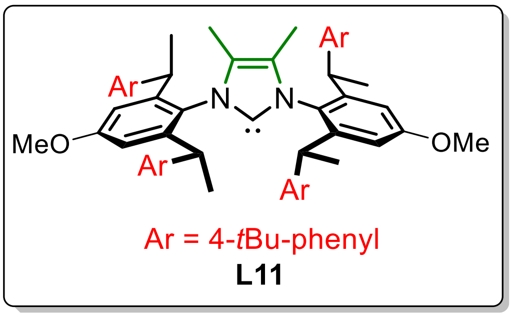

Inside the glovebox, the tetrafluoroborate salt **L11**·HBF$_4$ was suspended in THF/Toluene 1:1 (0.05 M) and NaH (4 equiv) was added in a, plus a catalytic amount (spatula tip) of KO*t*Bu in a Schlenk tube. The mixture was taken outside the glovebox and sonicated at room temperature for 4 hours, then the mixture was brought back inside the glovebox. After removal of the solid byproducts by filtration through a pad of celite on a frit, the solution was concentrated directly and afforded the desired carbene.

^{1}H NMR (400 MHz, C_6D_6) δ 7.80 (d, *J* = 8.8 Hz, 4H), 7.25 (d, *J* = 8.7 Hz, 4H), 7.17 – 7.13 (m, 4H), 7.08 (d, *J* = 2.9 Hz, 2H), 7.03 (d, *J* = 8.3 Hz, 4H), 6.86 (d, *J* = 2.9 Hz, 2H), 4.62 – 4.35 (m, 4H), 3.24 (s, 6H), 1.75 (d, *J* = 7.3 Hz, 6H), 1.48 – 1.35 (m, 12H), 1.19 (d, *J* = 6.3 Hz, 36H). **^{13}C NMR** (101 MHz, C_6D_6) δ 218.8, 160.1, 148.6, 147.5, 146.1, 143.6, 142.7, 131.6, 129.3, 125.7, 125.6, 125.5, 125.1, 112.2, 111.1, 54.7, 39.8, 37.4, 34.3, 34.3, 32.0, 31.5, 23.3, 23.1, 20.4, 14.4, 9.2.

3. Starting material synthesis

Phenyl benzyl ketones

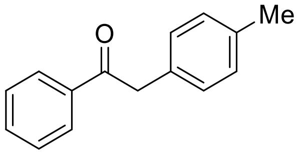

1a

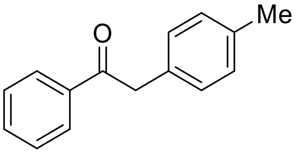

1b

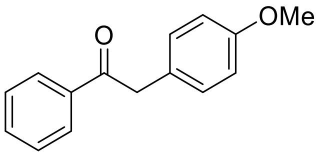

1c

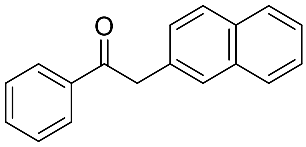

1d

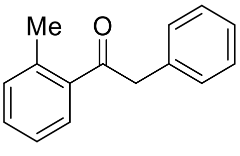

1e

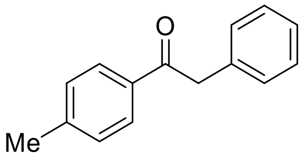

1f

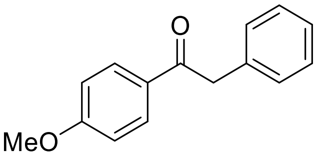

1g

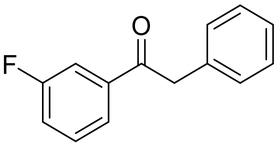

1h

Indanones

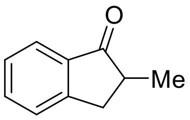

1i

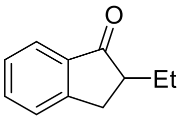

1j

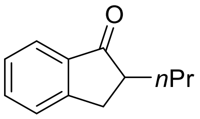

1k

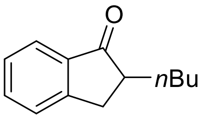

1l

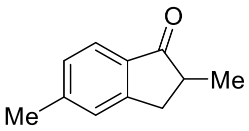

1m

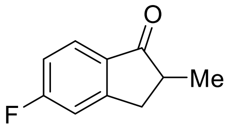

1n

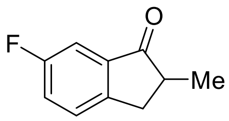

1o

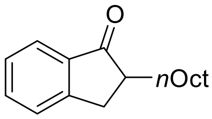

1p

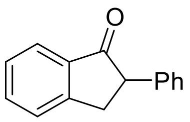

1q

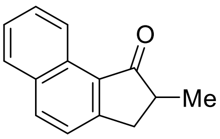

1r

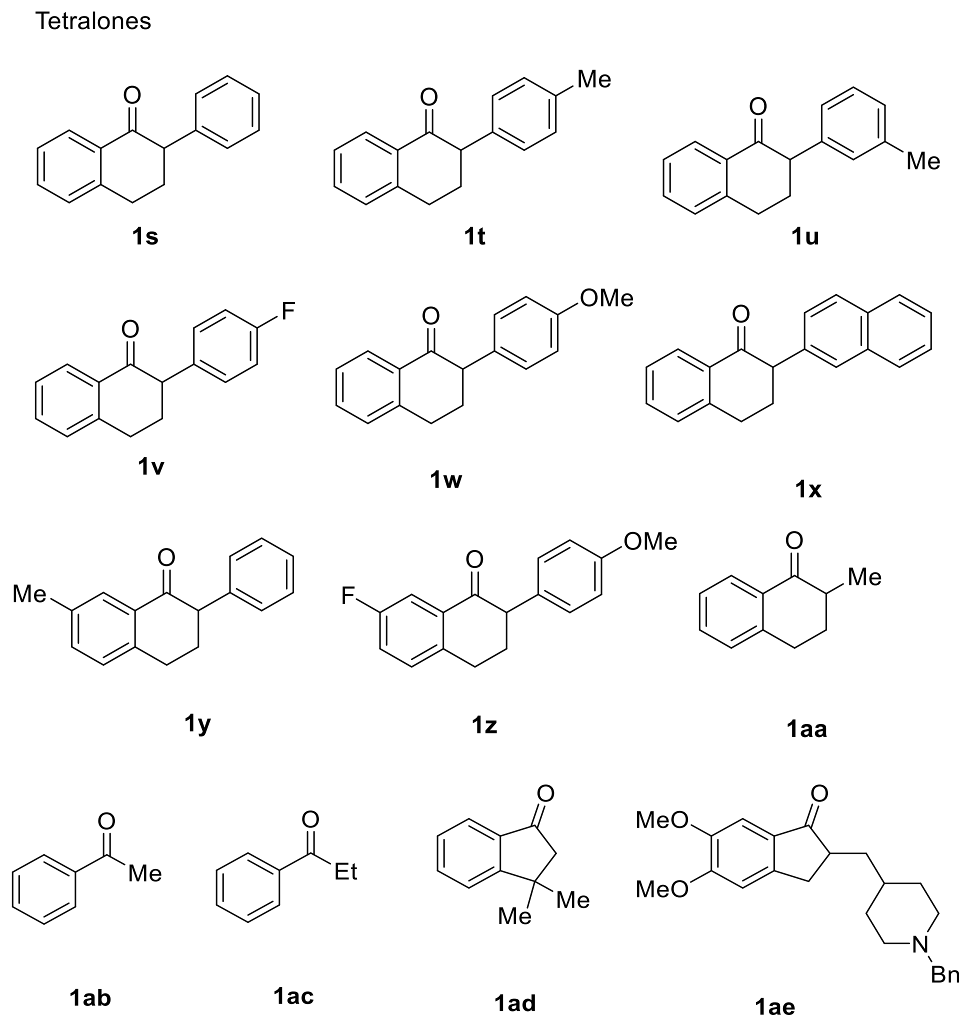

Supplementary Fig 3.1 | General overview of ketones as starting material.

Ketones **1a** to **1z** were synthesized according to the literature[14, 15, 16, 17, 18]. Ketones **1aa** to **1ae** were used as received.

<u>2-methyl-2,3-dihydro-1H-inden-1-one-2-d (**D-1i**):</u>

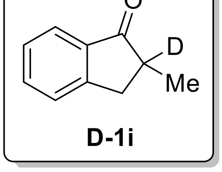

In a 10 mL round flask was added 2-methyl indanone **1i** (0.73 g, 5 mmol, and NaOD (40 wt% in D_2O, 0.4 mL) and CD_3OD (2 mL). The reaction mixture was allowed to stir at room temperature for 3 days. Then the reaction mixture was concentrated under vacuum. Then the desired product **D-1i** was afforded after silica gel chromatography as a colourless oil. (Pentane: EA 20:1. 0.70 g, 4.75 mmol, yield 95%). Deuterium degree >99%, substrate **1i** was not visible on the reported ^{1}H NMR and ^{13}C NMR spectra.

^{1}H NMR (400 MHz, $CDCl_3$) δ 7.74 (d, *J* = 7.7 Hz, 1H), 7.57 (td, *J* = 7.6, 1.1 Hz, 1H), 7.43 (d, *J* = 7.7 Hz, 1H), 7.35 (t, *J* = 7.4 Hz, 1H), 3.38 (d, *J* = 17.1 Hz, 1H), 2.71 (d, *J* = 17.1 Hz, 1H), 1.29 (s, 3H). ^{2}H NMR (61 MHz, $CHCl_3$) δ 2.40 (s, 1H).**^{13}C NMR** (101 MHz, $CDCl_3$) δ 209.6, 153.6, 136.4, 134.8, 127.4, 126.6, 124.0, 42.0 – 41.2 (m), 34.9, 16.3. **IR (ATR): $\tilde{\nu}$ (cm^{-1})** =

2962, 2929, 1705, 1608, 1587, 1464, 1435, 1372, 1325, 1281, 1209, 1194, 1165, 1149, 1089, 992, 952, 767, 735, 714, 674, 523, 468, 419. **HRMS (ESI) (m/z) calcd. for $C_{10}H_{10}DO^{+}$:** 148.0867; Found: 148.0872.

Olefins:

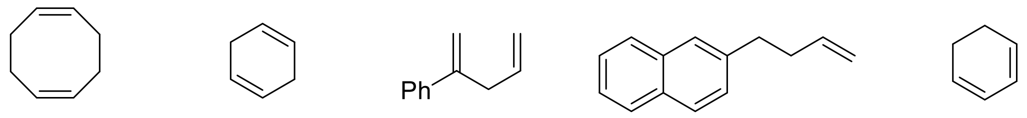

2a **2b** **2c** **2d** **2e**

Supplementary Fig 3.2 | General overview of dienes as starting material.

Olefins **2c** were synthesized according to the literature[19]. Olefins **2a**, **2b**, **2d** and **2e** were used as received.

4. Nickel(0) NHC catalyzed hydro-alkylation reaction.

4.1 Optimization of diastereoselective hydro-alkylation of non-conjugated diene.

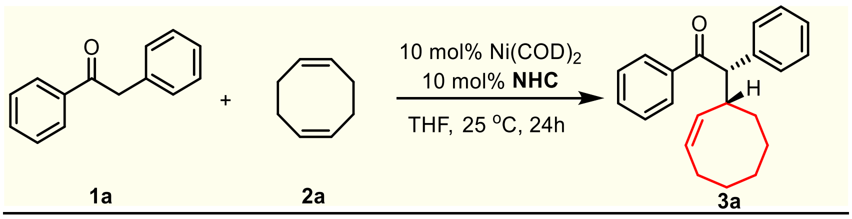

NHC ligands:

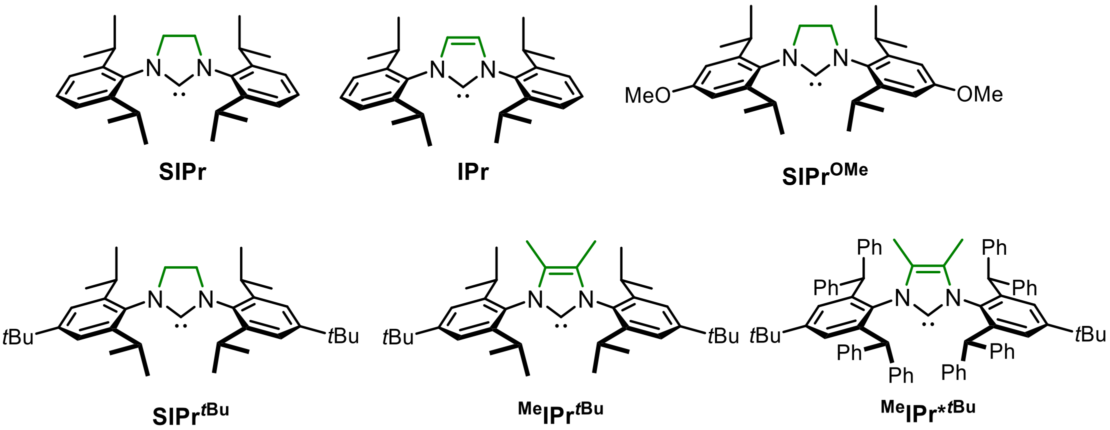

Entry	NHC	% Conv.[b]	% 3a[b]	dr of 3a[b]
1[c]	SIPr	74	70	1.2:1
2	SIPr	<5	traces	-
3	IPr	<5	traces	-
4	SIPr^{OMe}	43	43	7.0:1
5[d]	SIPr^{OMe}	43	42	6.7:1
6	SIPr^{tBu}	60	56	6.2:1
7	^{Me}IPr^{tBu}	85	85	4.2:1
8	^{Me}IPr*^{tBu}	<5	traces	-
9[e]	^{Me}IPr^{tBu}	0	0	-
10[f]	-	0	0	-

[a] 0.1 mmol **1a**, 0.15 mmol **2a**, 10 µmol Ni(COD)$_2$, 10 µmol **NHC** at 25 °C in THF (1 M) for 24 h; [b] determined by ^{1}H-NMR using 1, 3, 5,-trimethoxybenzene as internal standard; [c] 70 °C, 16 h; [d] 48 h; [e] without Ni(COD)$_2$; [f] without **NHC**.

Supplementary Fig 4.1 | Optimization of diastereoselective hydro-alkylation of non-conjugated dienes.

4.2 Control experiments.

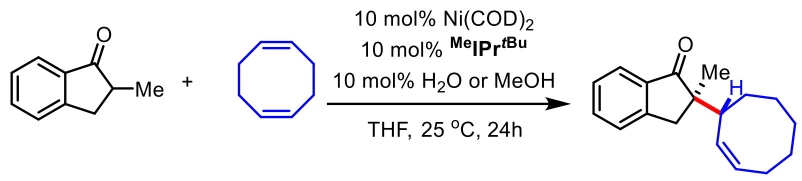

Supplementary Fig 4.2 | Influence of external proton source.

In a glovebox, an oven dried screw-capped 2 mL vial was charged with a magnetic stir bar, $Ni(COD)_2$ (10 µmol) , carbene ligand $^{Me}IPr^{tBu}$ (10 µmol), degassed (freeze pump thaw) THF (0.1 mL) was then added and the catalyst mixture was stirred at room temperature for 30 min, during which a red black solution is formed. Ketone (0.1 mmol degassed freeze pump thaw if the ketone is liquid) and COD (0.15 mmol degassed by freeze pump thaw) was then added successively. The vial was sealed with a Teflon-lined screw cap, and taking out of glovebox, then H_2O or MeOH (10 µmol)) was added under Schlenk line. After 24 h, the reaction mixture was diluted with dichloromethane and filtered through a plug of silica gel. After evaporated the solvent, the mixture was analysed by crude NMR.

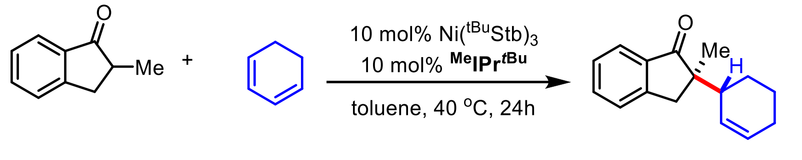

Supplementary Fig 4.3 | Investigation of conjugated diene.

In a glovebox, an oven dried screw-capped 2 mL vial was charged with a magnetic stir bar, Ni(^{tBu}stb)$_3$ (10 µmol), carbene ligand $^{Me}IPr^{tBu}$ (10 µmol), freshly distilled and degassed (freeze pump thaw) toluene (0.1 mL) was then added and the catalyst mixture was stirred at room temperature for 30 min, then 1,3-cyclohexadiene (0.2 mmol) was then added during which a green black solution is formed. The mixture was stirred at room temperature for 10 minutes, Ketone (0.1 mmol) was then added successively. The vial was sealed with a Teflon-lined screw cap, and running inside the glovebox at 40 °C After 24 h, the vial was shipped outside of the glovebox. The reaction mixture was diluted with dichloromethane and filtered through a plug of silica gel. The crude solution was concentrated in vacuum and subjected to column chromatography 100:1 PE/EA to isolate the products.

4.3 Scope of diastereoselective hydro-alkylation of nonconjugated dienes.

General procedure 2 (GP2)

In a glovebox, an oven dried screw-capped 2 mL vial was charged with a magnetic stir bar, $Ni(COD)_2$ (10 μmol) , carbene ligand $^{Me}IPr^{tBu}$ (10 μmol), degassed (freeze pump thaw) THF (0.1 mL) was then added and the catalyst mixture was stirred at room temperature for 30 min, during which a red black solution is formed. Ketone (0.1 mmol degassed freeze pump thaw if the ketone is liquid) and COD **2a** (0.15 mmol degassed by freeze pump thaw) was then added successively. The vial was sealed with a Teflon-lined screw cap, and running inside the glovebox at room temperature. After 24 h, the vial was shipped outside of the glovebox. The reaction mixture was diluted with dichloromethane and filtered through a plug of silica gel. The crude solution was concentrated in vacuum and subjected to column chromatography 100:1 PE/EA to isolate the products.

Note: Liquid reagents/reactants were degassed by freeze-pump-thaw (3×) before being stored in the glovebox at -30 °C. Solid reagents/reactants were dried under high vacuum for 16 h before being stored in the glovebox at -30 °C.

(*Z*)-2-(cyclooct-2-en-1-yl)-1,2-diphenylethan-1-one (**3a**):

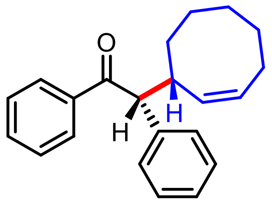

dr = 4.2:1
3a

According to **GP2**, using ketone **1a** and COD **2a** as starting materials, product **3a** was obtained as white solid in 82% yield (25.1 mg) dr = 4.2:1. **^{1}H NMR** (400 MHz, $CDCl_3$, the major isomer underlined) δ 7.99 – 7.82 (m, 2H), 7.47 – 7.04 (m, 8H),) δ 5.71 – 5.53 (m, 0.84H), 5.50 – 5.37 (m, 0.21H), 5.17 – 4.99 (m, 1H), 4.45 (m, 1H), 3.63 – 3.49 (m, 0.21H), 3.47 – 3.30 (m, 0.85H), 2.48 – 2.30 (m, 0.85H), 2.27 – 2.13 (m, 0.22H), 2.09 – 1.97 (m, 0.85H), 1.96 – 1.84 (m, 0.21H), 1.74 – 1.52 (m, 2H), 1.38 (m, 2H), 1.28 – 1.09 (m, 3H), 1.00 (m, 1H). **^{13}C NMR** (101 MHz, $CDCl_3$, the major isomer underlined) δ 200.7, 199.9, 138.4, 138.0, 137.8, 137.5, 133.1, 132.9, 132.8, 131.8, 131.0, 130.2, 129.1, 129.0, 128.9, 128.7, 128.7, 128.7, 128.6, 127.2, 127.1, 59.5, 58.9, 39.7, 39.5, 35.3, 33.3, 29.6, 27.2, 27.1, 26.9, 26.8, 25.9, 25.4. **IR (ATR): $\tilde{\nu}$ (cm^{-1})** = 2922, 1677, 1597, 1446, 1281, 1253, 1219, 1194, 1177, 973, 763, 744, 696, 671, 624. **HRMS (ESI) (m/z) calcd. for $C_{22}H_{25}O^+$:** 305.1900; Found: 305.1900. **m. p.:** 135-137 °C. **R_f:** 0.40 (Pentane:DCM 5:1).

(*Z*)-2-(cyclooct-2-en-1-yl)-1-phenyl-2-(p-tolyl)ethan-1-one (**3b**):

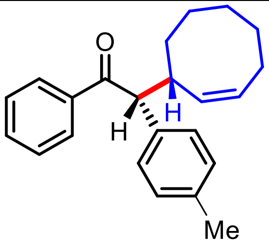

dr = 6.0:1
3b

According to **GP2**, using ketone **1b** and COD **2a** as starting materials, **3b** was obtained as white solid in 79% yield (25.0 mg) dr = 6.0:1. **^{1}H NMR** (400 MHz, $CDCl_3$, the major isomer underlined) δ 8.09 – 7.94 (m, 2H), 7.54 – 7.35 (m, 3H), 7.32 – 7.20 (m, 2H), 7.16 – 7.03 (m, 2H), 5.76 – 5.63 (m, 0.89H), 5.60 – 5.49 (m, 0.15H), 5.25 – 5.11 (m, 1H), 4.57 – 4.42 (m, 1H), 3.70 – 3.57 (m, 0.15H), 3.56 – 3.36 (m, 0.90H), 2.53 – 2.38 (m, 0.93H), 2.36 – 2.23 (m, 3.21H), 2.19 – 2.06 (m, 0.90H), 2.05 – 1.94 (m, 0.15H), 1.81 – 1.61 (m, 2H), 1.57 – 1.40 (m, 2H), 1.39 – 1.21 (m, 3H), 1.08 (tt, *J* = 13.1, 4.6 Hz, 1H). **^{13}C NMR** (101 MHz, $CDCl_3$, the major isomer underlined) δ 200.8, 199.9, 137.8, 137.5, 136.8,

136.7, 135.3, 134.9, 133.0, 132.8, 132.0, 130.9, 130.1, 129.6, 129.5, 129.0, 128.9, 128.7, 128.7, 128.6, 128.6, 59.1, 58.5, 39.6, 39.3, 35.3, 33.3, 29.6, 27.2, 27.1, 26.9, 26.8, 25.9, 25.5, 21.2. **IR (ATR): $\tilde{\nu}$ (cm^{-1})** = 2922, 1678, 1510, 1446, 1280, 1251, 1222, 1192, 1177, 799, 752, 732, 712, 689, 622. **HRMS (ESI) (m/z) calcd. for $C_{23}H_{27}O^+$:** 319.2056; Found: 319.2058. **m. p.:** 126-128 °C. **R_f:** 0.45 (Pentane:DCM 5:1).

(*Z*)-2-(cyclooct-2-en-1-yl)-2-(4-methoxyphenyl)-1-phenylethan-1-one (**3c**):

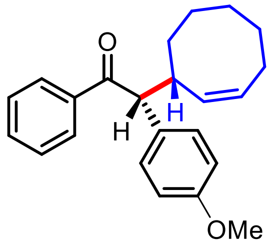
dr = 4.2:1
3c

According to **GP2**, using ketone **1c** and COD **2a** as starting materials, **3c** was obtained as white solid in 72% yield (24.0 mg) dr = 4.2:1. **^{1}H NMR** (400 MHz, $CDCl_3$, the major isomer underlined) δ 8.10 – 7.94 (m, 2H), 7.59 – 7.20 (m, 5H), 6.95 – 6.73 (m, 2H), 5.68 (q, *J* = 8.3 Hz, 0.87H), 5.54 (q, *J* = 9.1 Hz, 0.21H), 5.26 – 5.09 (m, 1H), 4.56 – 4.40 (m, 1H), 3.89 – 3.68 (m 3H), 3.66 – 3.54 (m, 0.23H), 3.44 (td, *J* = 12.9, 3.9 Hz, 0.84H), 2.44 (q, *J* = 10.0 Hz, 0.87H), 2.36 – 2.23 (m, 0.25H), 2.20 – 2.06 (m, 0.88H), 2.04 – 1.94 (m, 0.23H), 1.78 – 1.59 (m, 2H), 1.57 – 1.24 (m, 5H), 1.22 – 1.00 (m, 1H). **^{13}C NMR** (101 MHz, $CDCl_3$, the major isomer underlined) δ 200.8, 200.0, 158.8, 158.6, 137.8, 137.5, 133.0, 132.8, 132.0, 130.9, 130.4, 130.1, 130.0, 130.0, 128.7, 128.7, 128.6, 128.6, 114.3, 114.2, 58.6, 58.0, 55.3, 55.2, 39.6, 39.4, 35.3, 33.3, 29.6, 27.1, 27.1, 26.9, 26.8, 25.9, 25.5. **IR (ATR): $\tilde{\nu}$ (cm^{-1})** = 2925, 2850, 1679, 1608, 1596, 1580, 1509, 1446, 1302, 1282, 1250, 1224, 1177, 1035, 1004, 974, 808, 754, 712, 691, 622. **HRMS (ESI) (m/z) calcd. for $C_{23}H_{27}O_2^+$:** 335.2006; Found: 335.2016. **m. p.:** 146-148°C. **R_f:** 0.43 (Pentane:DCM 1:1).

(*Z*)-2-(cyclooct-2-en-1-yl)-2-(naphthalen-2-yl)-1-phenylethan-1-one (**3d**):

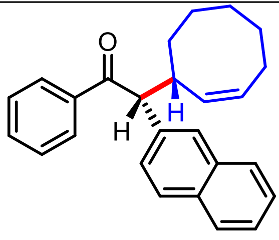
dr = 3.2:1
3d

According to **GP2**, using ketone **1d** and COD **2a** as starting materials, **3d** was obtained as white solid in 55% yield (19.6mg) dr = 3.2:1.**^{1}H NMR** (400 MHz, $CDCl_3$, the major isomer underlined) δ 8.10 – 7.99 (m, 2H), 7.91 – 7.72 (m, 4H), 7.62 – 7.34 (m, 6H), 5.79 – 5.67 (m, 0.77H), 5.53 – 5.43 (m, 0.25H), 5.29 – 5.17 (m, 1H), 4.77 – 4.65 (m, 1H), 3.76 (qd, *J* = 11.0, 3.6 Hz, 0.26H), 3.61 (ddt, *J* = 13.1, 9.4, 4.1 Hz, 0.79H), 2.60 – 2.43 (m, 0.80H), 2.40 – 2.24 (m, 0.29H), 2.22 – 2.08 (m, 0.80H), 2.05 – 1.91 (m, 0.27H), 1.88 – 1.07 (m, 8H). **^{13}C NMR** (101 MHz, $CDCl_3$, the major isomer underlined) δ 200.5, 199.7, 137.7, 137.4, 136.0, 135.6, 133.7, 133.6, 133.1, 132.9, 132.8, 132.7, 132.6, 131.8, 131.1, 130.3, 128.8, 128.7, 128.6, 128.5, 128.1, 128.0, 127.9, 127.8, 127.0, 126.8, 126.3, 126.1, 126.0, 125.8, 59.6, 59.1, 39.8, 39.5, 35.4, 33.4, 29.6, 27.2, 27.0, 26.9, 26.8, 25.9, 25.4. **IR (ATR): $\tilde{\nu}$ (cm^{-1})** = 2924, 2852, 1679, 1597, 1447, 1285, 1221, 1193, 813, 756, 712, 690, 478. **HRMS (ESI) (m/z) calcd. for $C_{26}H_{27}O^+$:** 355.2056; Found: 355.2057. **m. p.:** 196-198 °C. **R_f:** 0.60 (Pentane:DCM 3:1).

(Z)-2-(cyclooct-2-en-1-yl)-2-phenyl-1-(o-tolyl)ethan-1-one (**3e**):

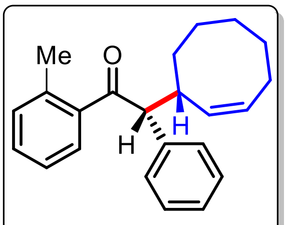

dr = 1.3:1
3e

According to **GP2**, using ketone **1e**, COD **2a** as starting materials and 40 °C was applied instead of RT, **3e** was obtained as colourless oil in 60% yield (19.2 mg) dr = 1.3:1. **^{1}H NMR** (400 MHz, $CDCl_3$, the major isomer underlined) δ 7.59 – 7.45 (m, 1H), 7.39 – 6.98 (m, 8H), 5.65 (q, *J* = 8.2 Hz, 0.56H), 5.45 (q, *J* = 8.7 Hz, 0.44H), 5.17 (t, *J* = 9.6 Hz, 0.59H), 5.01 (t, *J* = 9.8 Hz, 0.45H), 4.33 – 4.14 (m, 1H), 3.58 (qd, *J* = 12.1, 3.3 Hz, 0.47H), 3.48 – 3.32 (m, 0.57H), 2.46 – 2.31 (m, 0.58H), 2.29 – 2.09 (m, 3.36H), 2.09 – 1.99 (m, 0.64H), 1.92 (dq, *J* = 12.6, 4.0 Hz, 0.50H), 1.77 (dp, *J* = 13.4, 4.0 Hz, 0.49H), 1.71 – 1.11 (m, 7.33H), 0.95 (tt, *J* = 13.1, 4.5 Hz, 0.57H). **^{13}C NMR** (101 MHz, $CDCl_3$) δ 204.5, 204.2, 139.8, 139.7, 138.1, 138.0, 137.7, 137.7, 132.6, 131.7, 131.7, 131.6, 131.2, 130.9, 130.8, 130.2, 129.2, 129.2, 128.8, 128.7, 127.8, 127.7, 127.2, 127.2, 125.5, 125.4, 63.1, 62.9, 39.8, 38.7, 35.4, 33.2, 29.7, 27.2, 27.1, 27.0, 26.8, 26.0, 25.4, 20.7, 20.6. **IR (ATR): $\tilde{\nu}$ (cm^{-1})** = 2925, 2853, 1685, 1453, 759, 741, 729, 700, 627. **HRMS (ESI) (m/z) calcd. for $C_{23}H_{27}O^+$:** 319.2056; Found: 319.2056. **m. p.:** 102-104 °C. **R_f:** 0.47 (Pentane:DCM 5:1).

(*Z*)-2-(cyclooct-2-en-1-yl)-2-phenyl-1-(p-tolyl)ethan-1-one (**3f**):

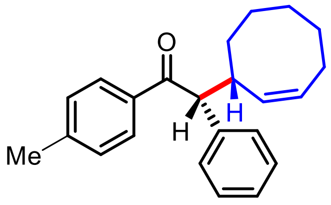

dr = 7.0:1
3f

According to **GP2**, using ketone **1f** and COD **2a** as starting materials, **3f** was obtained as white solid in 70% yield (22.3 mg) dr = 7.0:1.**^{1}H NMR** (400 MHz, $CDCl_3$, the major isomer underlined) δ 7.88 – 7.73 (m, 2H), 7.36 – 7.03 (m, 7H), 5.67 – 5.53 (m, 0.91H), 5.49 – 5.38 (m, 0.13H), 5.16 – 5.01 (m, 1H), 4.50 – 4.33 (m, 1H), 3.63 – 3.50 (m, 0.14H), 3.48 – 3.28 (m 0.92H), 2.44 – 2.31 (m, 0.93H), 2.29 (s, 0.34H), 2.27 (s, 2.65H), 2.23 – 2.16 (m, 0.14H) , 2.08 – 1.96 (m, 0.92H), 1.95 – 1.86 (m, 0.13H), 1.71 – 1.52 (m, 2H), 1.46 – 1.29 (m, 2H), 1.28 – 1.12 (m, 3H), 0.99 (tt, *J* = 13.1, 4.6 Hz, 1H). **^{13}C NMR** (101 MHz, $CDCl_3$, the major isomer underlined) δ 200.2, 199.4, 143.9, 143.7, 138.6, 138.3, 135.2, 134.9, 132.9, 132.0, 130.9, 130.1, 129.4, 129.3, 129.1, 129.0, 128.9, 128.8, 128.7, 127.1, 127.0, 59.3, 58.7, 39.6, 39.4, 35.3, 33.3, 29.6, 27.2, 27.1, 26.9, 26.8, 25.9, 25.5, 21.7. **IR (ATR): $\tilde{\nu}$ (cm^{-1})** = 2924, 2853, 1677, 1605, 1453, 1282, 1254, 1225, 1177, 808, 740, 701, 599. **HRMS (ESI) (m/z) calcd. for $C_{23}H_{27}O^+$:** 319.2056; Found: 319.2054. **m. p.:** 148-149 °C. **R_f:** 0.45 (Pentane:DCM 5:1).

(*Z*)-2-(cyclooct-2-en-1-yl)-1-(4-methoxyphenyl)-2-phenylethan-1-one (**3g**):

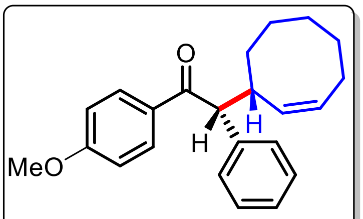

dr = 9.0:1
3g

According to **GP2**, using ketone **1g** and COD **2a** as starting materials, **3g** was obtained as white solid in 49% yield (16.5 mg) dr = 9.0:1.**^{1}H NMR** (400 MHz, $CDCl_3$, the major isomer underlined) δ 7.98 – 7.84 (m, 2H), 7.36 – 7.05 (m, 5H), 6.86 – 6.73 (m, 2H), 5.66 – 5.54 (m, 0.92H), 5.50 – 5.39 (m, 0.1H), 5.17 – 5.02 (m, 1H), 4.45 – 4.34 (m, 1H), 3.79 – 3.69 (m, 3H), 3.62 – 3.50 (m, 0.13H), 3.40 (ddt, *J* = 13.1, 9.6, 4.0 Hz, 0.93H), 2.37 (td, *J* = 13.3, 12.8, 5.9 Hz, 0.93H), 2.27 – 2.15 (m, 0.12H), 2.11 – 1.96 (m, 0.94H), 1.95 – 1.96 (m, 0.11H), 1.72 – 1.52 (m, 2H), 1.47 – 1.31 (m, 2H), 1.30 – 1.14 (m, 3H), 0.99 (tt, *J* = 13.1, 4.6 Hz, 1H). **^{13}C NMR** (101 MHz, $CDCl_3$, the major isomer

underlined) δ 199.1, 198.3, 163.5, 163.4, 138.8, 138.4, 132.9, 132.0, 131.0, 130.9, 130.7, 130.5, 130.1, 129.0, 128.9, 128.7, 127.1, 127.0, 113.9, 113.8, 59.1, 58.5, 55.6, 39.6, 39.4, 35.3, 33.3, 29.8, 29.6, 27.2, 27.1, 26.9, 26.8, 25.9, 25.5. **IR (ATR): $\tilde{\nu}$ (cm^{-1})** = 2926, 1671, 1599, 1574, 1509, 1454, 1260, 1225, 1168, 1031, 828, 741, 701, 606, 598. **HRMS (ESI) (m/z) calcd. for $C_{23}H_{27}O_2^+$:** 335.2006; Found: 335.1996. **m. p.:** 161-165°C. **R_f:** 0.42 (Pentane:DCM 1:1).

(*Z*)-2-(cyclooct-2-en-1-yl)-1-(3-fluorophenyl)-2-phenylethan-1-one (**3h**):

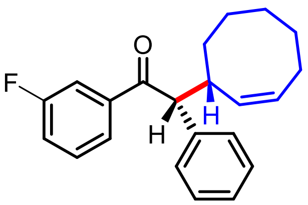

dr = 2:1
3h

According to **GP2**, using ketone **1h** and COD **2a** as starting materials, **3h** was obtained as white solid in 41% yield (13.2 mg) dr = 2.0:1. **^{1}H NMR** (400 MHz, $CDCl_3$, the major isomer underlined) δ 7.84 – 7.72 (m, 1H), 7.70 – 7.60 (m, 1H), 7.46 – 7.12 (m, 7H), 5.70 (q, *J* = 8.3 Hz, 0.68H), 5.52 (q, *J* = 8.6 Hz, 0.35H), 5.23 – 5.07 (m, 1H), 4.54 – 4.37 (m, 1H), 3.62 (qd, *J* = 11.4, 10.9, 3.3 Hz, 0.36H), 3.55 – 3.40 (m, 0.71H), 2.43 (q, *J* = 9.9 Hz, 0.71H), 2.33 – 2.20 (m, 0.36H), 2.18 – 2.06 (m, 0.69H), 2.03– 1.95 (m, 0.35H), 1.80 – 1.00 (m, 8H). **^{13}C NMR** (101 MHz, $CDCl_3$, the major isomer underlined) δ 199.4 (d, *J* = 1.7 Hz), 198.6 (d, *J* = 1.6 Hz), 162.9 (d, *J* = 247.7 Hz), 139.8 (d, *J* = 5.9 Hz), 139.6 (d, *J* = 6.0 Hz), 138.0, 137.6, 132.6, 131.5, 131.3, 130.5 – 130.1 (m), 129.1, 129.0, 128.9, 127.4, 127.3, 124.5 – 124.3 (m), 120.1 (d, *J* = 21.5 Hz), 119.9 (d, *J* = 21.5 Hz), 115.5 (d, *J* = 22.3 Hz), 115.4 (d, *J* = 22.3 Hz). 59.8, 59.3, 39.7, 39.4, 35.3, 33.2, 29.6, 27.2, 27.1, 26.9, 26.8, 25.9, 25.4. **^{19}F NMR** (376 MHz, $CDCl_3$, the major isomer underlined) δ -111.8, -112.0. **IR (ATR): $\tilde{\nu}$ (cm^{-1})** = 2926, 1683, 1587, 1453, 1440, 1272, 1255, 1150, 790, 741, 723, 700. **HRMS (ESI) (m/z) calcd. for $C_{22}H_{24}FO^+$:** 323.1806; Found: 323.1811. **m. p.:** 129-132 °C. **R_f:** 0.42 (Pentane:DCM 5:1).

(*Z*)-2-(cyclooct-2-en-1-yl)-2-methyl-2,3-dihydro-1H-inden-1-one (**3i**):

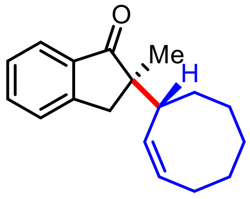

dr = 6.4:1
3i

According to **GP2**, using ketone **1i** and COD **2a** as starting materials, **3i** was obtained as colourless oil in 51% yield (13.0 mg) dr = 6.4:1. **^{1}H NMR** (400 MHz, $CDCl_3$, the major isomer underlined) δ 7.78 – 7.70 (m, 1H), 7.62 – 7.54 (m, 1H), 7.48 – 7.41 (m, 1H), 7.39 – 7.32 (m, 1H), 5.86 – 5.75 (m, 0.14H), 5.70 – 5.53 (m, 0.89H), 5.44 – 5.34 (m, 0.14H), 5.25 – 5.10 (m, 0.89H), 3.37 – 3.20 (m, 1H), 3.01 (ddd, *J* = 12.8, 9.5, 3.6 Hz, 0.14H), 2.94 – 2.74 (m, 1.90H), 2.42 – 2.17 (m, 1H), 2.12 – 1.93 (m, 1H), 1.86 – 1.73 (m, 1H), 1.73 – 1.62 (m, 2H), 1.58 – 1.32 (m, 3H), 1.31 – 1.12 (m, 5H). **^{13}C NMR** (101 MHz, $CDCl_3$, the major isomer underlined) δ 212.1, 211.5, 153.4, 153.1, 137.0, 136.8, 134.9, 134.8, 131.8, 131.2, 130.4, 129.5, 127.5, 127.5, 126.7, 126.5, 124.3, 124.1, 52.2, 51.4, 42.8, 42.2, 38.8, 37.4, 31.2, 30.5, 29.6, 29.6, 27.2, 27.0, 26.8, 26.7, 25.7, 25.6, 25.0, 22.8. **IR (ATR): $\tilde{\nu}$ (cm^{-1})** = 2926, 2860, 1710, 1608, 1490, 1464, 1454, 1283, 1227, 1019, 972, 952, 754, 740. **HRMS (ESI) (m/z) calcd. for $C_{18}H_{23}O^+$:** 255.1743; Found: 255.1733. **R_f:** 0.60 (Pentane:EA 10:1).

(*Z*)-2-(cyclooct-2-en-1-yl)-2-ethyl-2,3-dihydro-1H-inden-1-one (**3j**):

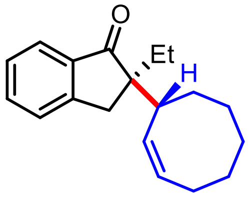

dr = 10.7:1
3j

According to **GP2**, using ketone **1j** and COD **2a** as starting materials, **3j** was obtained as colourless oil in 41% yield (11.1 mg) dr = 10.7:1. **^{1}H NMR** (400 MHz, $CDCl_3$, the major isomer underlined) δ 7.76 – 7.67 (m, 1H), 7.61 – 7.53 (m, 1H), 7.44 (d, J = 7.7 Hz, 1H), 7.34 (t, J = 7.4 Hz, 1H), 5.77 (q, J = 8.9 Hz, 0.09H), 5.70 – 5.54 (m, 0.96H), 5.42 – 5.26 (m, 1H), 3.26 – 3.09 (m, 1H), 3.03 – 2.85 (m, 2H), 2.41 – 2.17 (m, 1H), 2.06 – 1.96 (m, 1H), 1.75 (q, J = 7.4 Hz, 2H), 1.71 – 1.58 (m, 3H), 1.57 – 1.40 (m, 2H), 1.39 – 1.19 (m, 2H), 1.10 (tt, J = 12.8, 4.4 Hz, 1H), 0.75 (t, J = 7.4 Hz, 3H). **^{13}C NMR** (101 MHz, $CDCl_3$, the major isomer underlined) δ 211.5, 153.8, 138.3, 134.8, 134.7, 131.0, 130.5, 129.5, 127.4, 127.4, 126.4, 126.2, 123.7, 123.6, 55.3, 42.2, 41.6, 36.4, 30.4, 29.7, 29.6, 29.1, 27.2, 26.8, 25.6, 8.7, 8.5. **IR (ATR): $\tilde{\nu}$ (cm^{-1})** = 2924, 2854, 1706, 1607, 1463, 740. **HRMS (ESI) (m/z) calcd. for $C_{19}H_{25}O^+$:** 269.1900; Found: 269.1907. **R$_f$:** 0.62 (Pentane:EA 10:1).

(Z)-2-(cyclooct-2-en-1-yl)-2-propyl-2,3-dihydro-1H-inden-1-one (**3k**):

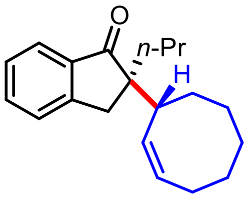

dr = 11.5:1
3k

According to **GP2**, using ketone **1k** and COD **2a** as starting materials, **3k** was obtained as colourless oil in 35% yield (10.0 mg) dr = 11.5:1. **^{1}H NMR** (400 MHz, $CDCl_3$, the major isomer underlined) δ 7.70 (d, J = 7.6 Hz, 1H), 7.56 (td, J = 7.5, 1.2 Hz, 1H), 7.48 – 7.40 (m, 1H), 7.38 – 7.30 (m, 1H), 5.83 – 5.73 (m, 0.08H), 5.69 – 5.54 (m, 0.88H), 5.39 – 5.24 (m, 1H), 3.28 – 3.12 (m, 1H), 3.06 (ddd, J = 13.2, 9.9, 4.2 Hz, 0.09H), 3.01 – 2.86 (m, 1.80H), 2.40 – 2.16 (m, 1H), 2.12 – 1.94 (m, 1H), 1.78 – 0.94 (m, 12H), 0.90 – 0.73 (m, 3H). **^{13}C NMR** (101 MHz, $CDCl_3$) δ 212.1, 211.6, 154.1, 153.7, 138.5, 138.2, 134.8, 134.7, 131.6, 131.0, 130.5, 129.4, 127.4, 126.4, 126.2, 123.8, 123.6, 56.0, 55.1, 42.6, 42.0, 41.1, 39.0, 36.8, 35.2, 30.7, 30.4, 29.6, 27.2, 27.1, 26.8, 25.6, 17.5, 17.4, 14.9, 14.8. **IR (ATR): $\tilde{\nu}$ (cm^{-1})** = 2955, 2925, 2852, 1704, 1608, 1463, 1295, 758, 739. **HRMS (ESI) (m/z) calcd. for $C_{20}H_{27}O^+$:** 283.2056; Found: 283.2051. **R$_f$:** 0.65 (Pentane:EA 10:1).

(Z)-2-butyl-2-(cyclooct-2-en-1-yl)-2,3-dihydro-1H-inden-1-one (**3l**):

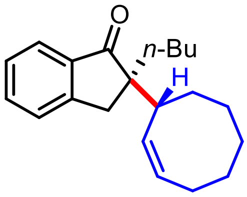

dr = 12.5:1
3l

According to **GP2**, using ketone **1l** and COD **2a** as starting materials, 40 °C was applied. **3l** was obtained as colourless oil in 62% yield (18.5 mg) dr = 12.5:1. **^{1}H NMR** (400 MHz, $CDCl_3$, the major isomer underlined) δ 7.70 (d, J = 7.7 Hz, 1H), 7.57 (td, J = 7.6, 1.0 Hz, 1H), 7.44 (d, J = 7.6 Hz, 1H), 7.34 (t, J = 7.4 Hz, 1H), 5.77 (dt, J = 10.0, 7.9 Hz, 0.08H), 5.61 (dddd, J = 10.1, 8.5, 7.3, 1.1 Hz, 0.94H), 5.30 (ddd, J = 10.8, 9.4, 1.5 Hz, 1H), 3.16 (d, J = 17.7 Hz, 1H), 3.02 – 2.86 (m, 2H), 2.24 (tdt, J = 12.9, 8.9, 1.9 Hz, 1H), 2.01 (dddd, J = 13.1, 8.0, 4.9, 3.3 Hz, 1H), 1.76 – 0.96 (m, 15H), 0.81 (t, J = 7.3 Hz, 3H). **^{13}C NMR** (101 MHz, $CDCl_3$) δ 212.2, 211.6, 154.1, 153.7, 138.5, 138.2, 134.8, 134.7, 131.6, 131.0, 130.5, 129.4, 127.4, 127.4, 126.4, 126.2, 123.8, 123.6, 55.8, 54.9, 42.7, 42.0, 39.0, 38.4, 36.8, 36.4, 30.7, 30.4, 29.6, 29.1, 27.2, 27.1, 26.8, 26.3, 26.2, 25.7, 25.6, 23.5, 23.4, 14.2, 14.1. **IR (ATR): $\tilde{\nu}$ (cm^{-1})** = 2925, 2856, 1705, 1607, 1463, 1275, 741, 712. **HRMS (ESI) (m/z) calcd. for $C_{21}H_{29}O^+$:** 297.2213; Found: 297.2218. **R$_f$:** 0.67 (Pentane:EA 10:1).

(Z)-2-(cyclooct-2-en-1-yl)-2,5-dimethyl-2,3-dihydro-1H-inden-1-one (**3m**):

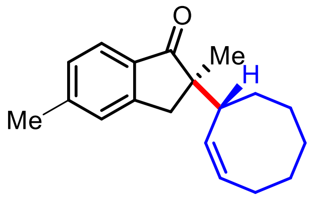

dr = 3.6:1
3m

According to **GP2**, using ketone **1m** and COD **2a** as starting materials, 40 °C was applied. **3m** was obtained as colourless oil in 58% yield (15.5 mg) dr = 3.6:1. **^{1}H NMR** (400 MHz, $CDCl_3$, the major isomer underlined) δ 7.63 (dd, *J* = 11.0, 7.9 Hz, 1H), 7.23 (d, *J* = 6.3 Hz, 1H), 7.16 (d, *J* = 7.8 Hz, 1H), 5.85 – 5.74 (m, 0.23H), 5.66 – 5.53 (m, 0.83H), 5.37 (t, *J* = 10.1 Hz, 0.23H), 5.17 (t, *J* = 10.0 Hz, 0.83H), 3.31 – 3.12 (m, 1H), 3.05 – 2.95 (m, 0.22H), 2.86 (ddd, *J* = 12.9, 9.6, 3.9 Hz, 0.83H), 2.81 – 2.71 (m, 1H), 2.43 (s, 3H), 2.26 (dt, *J* = 23.4, 13.2 Hz, 1H), 2.02 (tdt, *J* = 16.5, 7.4, 3.8 Hz, 1H), 1.77 (ddt, *J* = 14.3, 8.6, 4.2 Hz, 0.87H), 1.71 – 1.28 (m, 6.25H), 1.28 – 1.10 (m, 4.92H), 1.00 (ddt, *J* = 18.1, 8.7, 4.6 Hz, 0.29H). **^{13}C NMR** (101 MHz, $CDCl_3$, the major isomer underlined) δ 211.6, 211.0, 153.9, 153.6, 146.0, 145.9, 134.8, 134.5, 131.8, 131.1, 130.6, 129.6, 128.8, 128.8, 127.0, 126.8, 124.2, 124.0, 52.3, 51.5, 42.8, 42.2, 38.6, 37.2, 31.1, 30.5, 29.6, 29.6. 27.2, 27.0, 26.8, 26.7, 25.8, 25.6, 25.0, 22.8, 22.2. **IR (ATR): $\tilde{\nu}$ (cm^{-1})** = 2924, 2854, 1704, 1610, 1450, 1278, 830, 753. **HRMS (ESI) (m/z) calcd. for $C_{19}H_{25}O^{+}$:** 269.1900; Found: 269.1900. **R_f:** 0.62 (Pentane:EA 10:1).

(Z)-2-(cyclooct-2-en-1-yl)-5-fluoro-2-methyl-2,3-dihydro-1H-inden-1-one (**3n**):

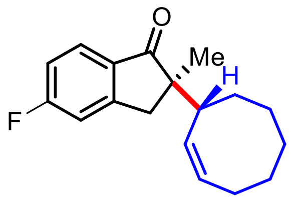

dr = 6.0:1
3n

According to **GP2**, using ketone **1n** and COD **2a** as starting materials, 40 °C was applied. **3n** was obtained as colourless oil in 33% yield (9.1 mg) dr = 6.0:1. **^{1}H NMR** (400 MHz, $CDCl_3$, the major isomer underlined) δ 7.80 – 7.66 (m, 1H), 7.17 – 6.99 (m, 2H), 5.85 – 5.76 (m, 0.15H), 5.69 – 5.55 (m, 0.92H), 5.40 – 5.31 (m, 0.15H), 5.21 – 5.09 (m, 0.91H), 3.23 (d, *J* = 17.7 Hz, 1H), 3.04 – 2.95 (m, 0.16H), 2.91 – 2.74 (m, 1.92H), 2.42 – 2.16 (m, 1H), 2.06 – 1.95 (m, 1H), 1.84 – 1.62 (m, 3H), 1.60 – 1.28 (m, 3.70H), 1.28 – 1.12 (m, 5.31H), 1.00 (ddt, *J* = 18.1, 9.1, 4.6 Hz, 0.20H). **^{13}C NMR** (101 MHz, $CDCl_3$, the major isomer underlined) δ 209.5, 168.7, 166.1, 156.0, 155.9, 133.1 (d, *J* = 1.7 Hz), 132.0, 131.4, 130.2, 129.3, 126.6 (d, *J* = 10.5 Hz), 115.8 (d, *J* = 23.8 Hz), 113.0 (d, *J* = 22.0 Hz), 52.6, 51.8, 42.8, 42.2, 38.7, 38.7, 31.1, 30.5, 29.6, 29.5, 27.2, 27.0, 26.8, 26.8, 25.7, 25.6, 24.9, 22.7. **^{19}F NMR** (376 MHz, $CDCl_3$, the major isomer underlined) δ -103.1, -103.2. **IR (ATR): $\tilde{\nu}$ (cm^{-1})** = 2926, 2853, 1709, 1616, 1593, 1483, 1453, 1331, 1292, 1254, 1085, 973, 859, 750. **HRMS (ESI) (m/z) calcd. for $C_{18}H_{22}FO^{+}$:** 273.1649; Found: 273.1648. **R_f:** 0.60 (Pentane:EA 10:1).

(Z)-2-(cyclooct-2-en-1-yl)-6-fluoro-2-methyl-2,3-dihydro-1H-inden-1-one (**3o**):

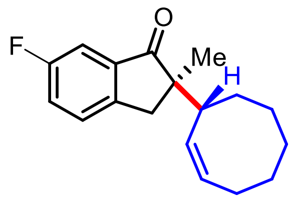

dr = 8.3:1
3o

According to **GP2**, using ketone **1o** and COD **2a** as starting materials, **3o** was obtained as colourless oil in 67% yield (18.2 mg) dr = 8.3:1.**^{1}H NMR** (400 MHz, $CDCl_3$, the major isomer underlined) δ 7.38 – 7.16 (m, 3H), 5.78 – 5.69 (m, 0.10H), 5.61 – 5.50 (m, 0.86H), 5.28 (t, *J* = 10.0 Hz, 0.1H), 5.08 (t, *J* = 10.0 Hz, 0.86H), 3.24 – 3.08 (m, 1H), 2.93 (ddd, *J* = 12.7, 9.8, 3.7 Hz, 0.12H), 2.86 – 2.64 (m, 1.85H), 2.33 – 2.09 (m, 1H), 2.05 – 1.87 (m, 1H), 1.79 – 1.03 (m, 11H). **^{13}C NMR** (101 MHz, $CDCl_3$, the major isomer underlined) δ 211.2 (d, *J* = 2.5 Hz), 210.5 (d, *J* = 2.5 Hz), 162.5 (d, *J* = 247.7 Hz), 148.8 – 148.7 (m), 148.4 (d, *J* = 1.7 Hz), 138.7 (d, *J* = 6.8 Hz), 138.6 – 138.4 (m), 132.0, 131.4, 130.1, 129.2, 128.0 (d, *J* = 7.8 Hz), 127.8 (d, *J* = 7.9 Hz), 122.5 (d, *J* = 23.7 Hz), 110.0 (d, *J* = 21.5 Hz), 109.7, 53.3, 52.6, 42.9, 42.3, 38.2, 36.8, 31.1, 30.5, 29.6, 29.5, 27.2, 27.0, 26.8, 26.8, 25.7, 25.6, 24.9, 22.7. ^{19}F NMR (376 MHz, CDCl3, the major isomer underlined) δ -114.6, -114.7. **IR (ATR): $\tilde{\nu}$ (cm^{-1})** = 2925, 2854, 1710, 1484, 1448, 1290, 1263, 882, 771, 749. **HRMS (ESI) (m/z) calcd. for $C_{18}H_{22}FO^{+}$:** 273.1649; Found: 273.1648. **R_f:** 0.60 (Pentane:EA 10:1).

General procedure 3 (GP3)

In a glovebox, an oven dried screw-capped 2 mL vial was charged with a magnetic stir bar, Ni(^{tBu}stb)$_3$ (10 μmol), carbene ligand ^{Me}IPr^{tBu} (10 μmol), freshly distilled and degassed (freeze pump thaw) toluene (0.1 mL) was then added and the catalyst mixture was stirred at room temperature for 30 min, 1,4-cyclohexadiene **2b** (0.2 mmol) was then added during which a dark solution is formed. The mixture was stirred at room temperature for 10 minutes, ketone (0.1 mmol) was then added successively. The vial was sealed with a Teflon-lined screw cap, and running inside the glovebox at RT (26-27 °C). After 24 h, the vial was shipped outside of the glovebox. The reaction mixture was diluted with dichloromethane and filtered through a plug of silica gel. The crude solution was concentrated in vacuum and subjected to column chromatography to isolate the products.

Note: Liquid reagents/reactants were degassed by freeze-pump-thaw (3×) before being stored in the glovebox at -30 °C. Solid reagents/reactants were dried under high vacuum for 16h before being stored in the glovebox at -30 °C.

2-(cyclohex-2-en-1-yl)-1,2-diphenylethan-1-one (**4a**):

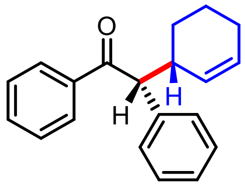

dr = >20:1
4a

According to **GP3**, using ketone **1a** and 1,4-cyclohexadiene **2b** as starting materials, **4a** was obtained as white solid in 85% yield (23.5 mg) dr = > 20:1.**^{1}H NMR** (400 MHz, $CDCl_3$) δ 7.96 – 7.85 (m, 2H), 7.45 – 7.37 (m, 1H), 7.36 – 7.17 (m, 6H), 7.16 – 7.08 (m, 1H), 5.63 (m, 1H), 5.57 – 5.50 (m, 1H), 4.33 (d, *J* = 10.9 Hz, 1H), 3.06 (m, 1H), 1.92 (m, 2H), 1.63 (m, 1H), 1.43 (m, 1H), 1.30 (m, 1H), 1.09 (m, 1H). **^{13}C NMR** (101 MHz, $CDCl_3$) δ 200.2, 137.9, 137.6, 133.0, 130.1, 128.9, 128.9, 128.8, 128.7, 128.6, 127.3, 59.1, 38.8, 26.9, 25.5, 21.2. **IR (ATR): $\tilde{\nu}$ (cm^{-1})** = 3060, 3023, 2922, 2859, 2835, 1961, 1811, 1746, 1677, 1597,

1580, 1492, 1447, 1394, 1340, 1274, 1210, 1176, 1159, 1071, 1002, 954, 932, 908, 894, 867, 838, 815, 765, 749, 723, 697, 685, 656, 619, 586, 549, 518, 420, 403. **HRMS (ESI) (m/z) calcd. for $C_{20}H_{21}O^+$:** 277.1587; Found: 277.1587. **m. p.:** 102-104 °C. **R_f:** 0.40 (Pentane:DCM 10:1).

2-(cyclohex-2-en-1-yl)-1-phenyl-2-(p-tolyl)ethan-1-one (**4b**):

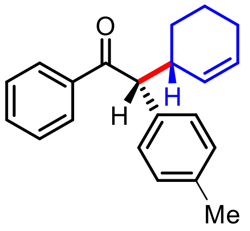

dr = 11.5:1
4b

According to **GP3**, using ketone **1b** and 1,4-cyclohexadiene **2b** as starting materials, **4b** was obtained as thick oil in 75% yield (21.6 mg) dr = 11.5:1.**^{1}H NMR** (400 MHz, $CDCl_3$, the major isomer underlined) δ 8.02 – 7.93 (m, 2H), 7.47 (t, J = 7.3 Hz, 1H), 7.44 – 7.39 (m, 2H), 7.23 (d, J = 8.0 Hz, 2H), 7.09 (d, J = 7.9 Hz, 2H), 5.74 – 5.55 (m, 3.4 Hz, 1H), 5.64 – 5.56 (m, 0.94H), 5.26 – 5.19 (m, 0.08H), 4.42 – 4.32 (m, 1H), 3.11 (dddt, J = 10.7, 8.0, 5.2, 2.5 Hz, 1H), 2.28 (s, 3H), 2.08 – 1.93 (m, 2H), 1.78 – 1.64 (m, 1H), 1.57 – 1.44 (m, 1H), 1.44 – 1.33 (m, 1H), 1.23 – 1.10 (m, 1H). **^{13}C NMR** (101 MHz, $CDCl_3$, the major isomer underlined) δ 200.3, 137.7, 136.9, 134.8, 133.0, 132.9, 130.2, 129.6, 129.1, 128.7, 128.7, 128.6, 58.8, 58.5, 38.8, 38.7, 28.4, 26.9, 25.6, 25.4, 21.4, 21.2, 21.2. **IR (ATR): $\tilde{\nu}$ (cm^{-1}) =** 2923, 1678, 1511, 1447, 1274, 1211, 1181, 1002, 798, 787, 753, 691, 657. **HRMS (ESI) (m/z) calcd. for $C_{21}H_{23}O^+$:** 291.1743; Found: 291.1753. **R_f:** 0.45 (Pentane:DCM 10:1).

2-(cyclohex-2-en-1-yl)-1-phenyl-2-(p-tolyl)ethan-1-one (**4c**):

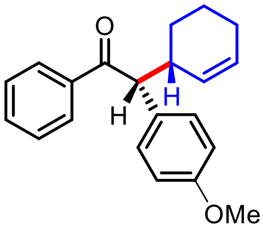

dr = 12.5:1
4c

According to **GP3**, using ketone **1c** and 1,4-cyclohexadiene **2b** as starting materials, **4c** was obtained as thick oil in 70% yield (21.6 mg) dr = 12.5:1. **^{1}H NMR** (400 MHz, $CDCl_3$, the major isomer underlined) δ 8.02 – 7.90 (m, 2H), 7.52 – 7.44 (m, 1H), 7.43 – 7.34 (m, 2H), 7.31 – 7.20 (m, 2H), 6.86 – 6.75 (m, 2H), 5.73 – 5.50 (m, 2H), 5.28 – 5.16 (m, 0.08H), 4.43 – 4.26 (m, 1H), 3.75 (s, 3H), 3.08 (dddt, J = 10.8, 8.2, 5.3, 2.5 Hz, 1H), 2.08 – 1.90 (m, 2H), 1.78 – 1.62 (m, 1H), 1.58 – 1.32 (m, 2H), 1.25 – 1.08 (m, 1H). **^{13}C NMR** (101 MHz, $CDCl_3$) δ 200.4, 158.9, 137.7, 132.9, 130.2, 129.9, 128.7, 128.7, 128.6, 114.4, 58.2, 55.3, 38.7, 26.9, 25.6, 21.2. **IR (ATR): $\tilde{\nu}$ (cm^{-1}) =** 2931, 2835, 1677, 1608, 1597, 1581, 1509, 1447, 1302, 1275, 1250, 1211, 1177, 1034, 1002, 831, 804, 753, 690, 657, 564. **HRMS (ESI) (m/z) calcd. for $C_{21}H_{23}O_2^+$:** 307.1693; Found: 307.1693. **R_f:** 0.45 (Pentane:DCM 2:1).

2-(cyclohex-2-en-1-yl)-2-(naphthalen-2-yl)-1-phenylethan-1-one (**4d**):

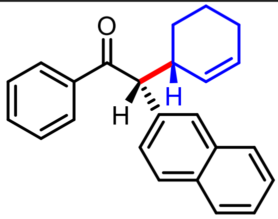

dr = 11.3:1
4d

According to **GP3**, using ketone **1d** and 1,4-cyclohexadiene **2b** as starting materials, **4d** was obtained as thick oil in 51% yield (16.8 mg) dr = 11.3:1. **^{1}H NMR** (400 MHz, $CDCl_3$, the major isomer underlined) δ 8.06 – 7.98 (m, 2H), 7.84 – 7.73 (m, 4H), 7.58 – 7.35 (m, 6H), 5.80 – 5.61 (m, 1.9H), 5.24 – 5.17 (m, 0.1H), 4.67 – 4.51 (m, 1H), 3.24 (dddq, J = 10.8, 8.0, 5.1, 2.6 Hz, 1H), 2.07 – 1.94 (m, 2H), 1.77 – 1.65 (m, 1H), 1.57 – 1.45 (m, 1H), 1.43 – 1.32 (m, 1H), 1.28 – 1.16 (m, 1H). **^{13}C NMR** (101 MHz,

CDCl$_3$, the major isomer underlined) δ 200.13, 200.00, 137.6, 135.4, 133.7, 133.0, 132.7, 130.1, 128.9, 128.8, 128.7, 128.7, 127.9, 127.8, 127.8, 126.7, 126.3, 126.0, 59.2, 59.0, 38.8, 27.0, 25.6, 25.4, 21.4, 21.2. **IR (ATR): $\tilde{\nu}$ (cm^{-1}) =** 2924, 1677, 1596, 1447, 1347, 1279, 1227, 1211, 1181, 1002, 909, 812, 793, 745, 689, 662, 478. **HRMS (ESI) (m/z) calcd. for $C_{24}H_{23}O^+$:** 291.1743; Found: 291.1753. **R$_f$:** 0.50 (Pentane:DCM 10:1).

General procedure 4 (GP4)

In a glovebox, an oven dried screw-capped 2 mL vial was charged with a magnetic stir bar, Ni(^{tBu}stb)$_3$ (10 μmol), carbene ligand ^{Me}IPr^{tBu} (10 μmol), freshly distilled and degassed (freeze pump thaw) toluene (0.1 mL) was then added and the catalyst mixture was stirred at room temperature for 30 min, then 1,4-cyclohexadiene (0.2 mmol) **2b** was then added during which a dark solution is formed. The mixture was stirred at room temperature for 10 minutes, ketone (0.1 mmol) was then added successively. The vial was sealed with a Teflon-lined screw cap, and running inside the glovebox at 40 ^{o}C After 24 h, the vial was shipped outside of the glovebox. The reaction mixture was diluted with dichloromethane and filtered through a plug of silica gel. The crude solution was concentrated in vacuum and subjected to column chromatography 100:1 PE/EA to isolate the products.

Note: Liquid reagents/reactants were degassed by freeze-pump-thaw (3×) before being stored in the glovebox at -30 °C. Solid reagents/reactants were dried under high vacuum for 16h before being stored in the glovebox at -30 °C.

2-(cyclohex-2-en-1-yl)-2-methyl-2,3-dihydro-1H-inden-1-one (**4i**):

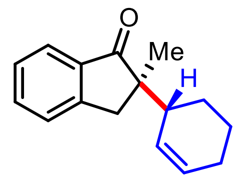

dr = 8.3:1

4i

According to **GP4**, using ketone **1i** and 1,4-cyclohexadiene **2b** as starting materials, **4i** was obtained as colourless oil in 85% yield (19.3 mg) dr = 8.3:1.**^{1}H NMR** (400 MHz, CDCl$_3$, the major isomer underlined) δ 7.76 (d, *J* = 7.7 Hz, 1H), 7.57 (t, *J* = 7.8 Hz, 1H), 7.42 (d, *J* = 7.7 Hz, 1H), 7.36 (t, *J* = 7.4 Hz, 1H), 5.89 – 5.81 (m, 0.11H), 5.74 (d, *J* = 10.4 Hz, 0.11H), 5.71 – 5.59 (m, 0.92H), 5.07 (d, *J* = 10.2 Hz, 0.90H), 3.15 (d, *J* = 17.2 Hz, 1H), 2.78 – 2.63 (m, 1.92H), 2.60 – 2.48 (m, 0.12H), 2.07 – 1.78 (m, 4H), 1.61 – 1.39 (m, 1H), 1.33 – 1.14 (m, 4H), 0.90 – 0.76 (m, 0.15H). **^{13}C NMR** (101 MHz, CDCl$_3$, the major isomer underlined) δ 212.1, 211.5, 153.7, 153.6, 136.9, 136.5, 134.9, 134.9, 130.3, 130.2, 128.1, 127.5, 127.4, 126.8, 126.5, 124.2, 124.2, 52.6, 51.7, 42.6, 42.1, 37.4, 37.2, 25.3, 25.3, 25.2, 25.1, 23.0, 22.6, 22.4, 22.2. **IR (ATR): $\tilde{\nu}$ (cm^{-1}) =** 2926, 2859, 1710, 1608, 1464, 1433, 1281, 1208, 975, 744, 727, 677. **HRMS (ESI) (m/z) calcd. for $C_{16}H_{19}O^+$:** 227.1430; Found: 227.1434. **R$_f$:** 0.60 (Pentane:EA 10:1).

5 mmol scale synthesis:

In a glovebox, an oven dried screw-capped 2 mL vial was charged with a magnetic stir bar, Ni(^{tBu}stb)$_3$ (0.25 mmol), carbene ligand ^{Me}IPr^{tBu} (0.25 mmol), freshly distilled and degassed (freeze pump thaw) toluene (2.5 mL) was then added and the catalyst mixture was stirred at room temperature for 30 min, then 1,4-cyclohexadiene (10 mmol) **2b** was then added during which a dark solution is formed. The mixture was stirred at room temperature for 10 minutes,

ketone (5 mmol) was then added successively. The vial was sealed with a Teflon-lined screw cap, and running inside the glovebox at 40 °C After 48 h, the vial was shipped outside of the glovebox. The reaction mixture was diluted with dichloromethane and filtered through a plug of silica gel. The crude solution was concentrated in vacuum and subjected to column chromatography 100:1 PE/EA to isolate the products 74% yield dr = 8.0:1.

2-(cyclohex-2-en-1-yl)-2-ethyl-2,3-dihydro-1H-inden-1-one (**4j**):

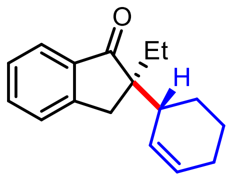

dr = 18.7:1
4j

According to **GP4**, using ketone **1j** and 1,4-cyclohexadiene **2b** as starting materials, **4j** was obtained as colourless oil in 90% yield (21.6 mg) dr = 18.7:1. ^{1}**H NMR** (400 MHz, $CDCl_3$, the major isomer underlined) δ 7.73 (d, *J* = 7.6 Hz, 1H), 7.56 (td, *J* = 7.5, 1.2 Hz, 1H), 7.42 (dt, *J* = 7.7, 0.8 Hz, 1H), 7.38 – 7.31 (m, 1H), 5.86 – 5.78 (m, 0.05H), 5.69 – 5.56 (m, 1H), 5.04 (dp, *J* = 10.1, 2.0 Hz, 1H), 3.09 (d, *J* = 17.3 Hz, 1H), 2.84 – 2.68 (m, 2H), 2.02 – 1.90 (m, 3H), 1.87 – 1.72 (m, 2H), 1.69 – 1.47 (m, 2H), 1.29 – 1.14 (m, 1H), 0.67 (t, *J* = 7.5 Hz, 3H). ^{13}C NMR (101 MHz, $CDCl_3$, the major isomer underlined) δ 212.1, 211.6, 154.4, 138.4, 138.1, 134.8, 130.4, 130.2, 128.1, 127.6, 127.3, 127.2, 126.4, 126.3, 123.5, 56.4, 55.8, 42.2, 41.6, 35.2, 34.7, 29.1, 25.3, 25.1, 24.9, 22.4, 22.2, 8.6. **IR (ATR):** $\tilde{\nu}$ **(cm^{-1}) =** 2962, 2921, 2857, 1703, 1606, 1463, 1433, 1295, 1280, 1263, 777, 746, 720, 678. **HRMS (ESI) (m/z) calcd. for $C_{17}H_{21}O^+$:** 241.1587; Found: 241.1588. **R_f:** 0.62 (Pentane:EA 10:1).

2-(cyclohex-2-en-1-yl)-2-propyl-2,3-dihydro-1H-inden-1-one (**4k**):

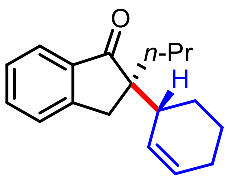

dr = >20:1
4k

According to **GP4**, using ketone **1k** and 1,4-cyclohexadiene **2b** as starting materials, **4k** was obtained as colourless oil in 92% yield (23.5 mg) dr = >20:1. ^{1}**H NMR** (400 MHz, $CDCl_3$, the major isomer underlined) δ 7.73 (d, *J* = 7.6 Hz, 1H), 7.56 (td, *J* = 7.5, 1.2 Hz, 1H), 7.42 (dt, *J* = 7.7, 0.8 Hz, 1H), 7.38 – 7.31 (m, 1H), 5.86 – 5.79 (m, 0.04H), 5.69 – 5.57 (m, 1H), 5.04 (dt, *J* = 10.3, 1.8 Hz, 1H), 3.10 (d, *J* = 17.3 Hz, 1H), 2.85 – 2.67 (m, 2H), 2.05 – 1.91 (m, 3H), 1.87 – 1.77 (m, 1H), 1.69 (ddd, *J* = 13.2, 11.5, 5.7 Hz, 1H), 1.55 (dddd, *J* = 17.4, 10.1, 6.3, 2.5 Hz, 2H), 1.22 (tdd, *J* = 13.2, 11.0, 2.9 Hz, 1H), 1.12 – 0.93 (m, 2H), 0.79 (t, *J* = 7.2 Hz, 3H), 0.63 (t, *J* = 7.2 Hz, 0.02H). ^{13}**C NMR** (101 MHz, $CDCl_3$, the major isomer underlined) δ 212.2, 154.3, 138.0, 134.8, 130.4, 130.2, 128.0, 127.6, 127.3, 126.4, 126.3, 123.5, 56.2, 55.5, 42.5, 42.0, 39.1, 38.9, 35.5, 35.1, 25.4, 25.1, 24.8, 22.4, 22.2, 17.4, 14.8. **IR (ATR):** $\tilde{\nu}$ **(cm^{-1}) =** 2955, 2928, 2870, 1706, 1607, 1463, 1294, 923, 751, 740, 723. **HRMS (ESI) (m/z) calcd. for $C_{18}H_{23}O^+$:** 255.1743; Found: 255.1744. **R_f:** 0.65 (Pentane:EA 10:1).

2-butyl-2-(cyclohex-2-en-1-yl)-2,3-dihydro-1H-inden-1-one (**4i**):

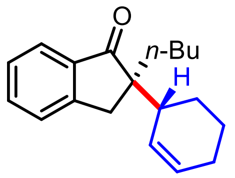

dr = >20:1
4i

According to **GP4**, using ketone **1i** and 1,4-cyclohexadiene **2b** as starting materials, **4i** was obtained as colourless oil in 95% yield (25.6 mg) dr = >20:1. **^{1}H NMR** (400 MHz, $CDCl_3$, the major isomer underlined) δ 7.73 (d, *J* = 7.6 Hz, 1H), 7.56 (td, *J* = 7.5, 1.2 Hz, 1H), 7.42 (d, *J* = 7.7 Hz, 1H), 7.38 – 7.31 (m, 1H), 5.86 – 5.79 (m, 0.04H), 5.70 (dt, *J* = 10.3, 1.7 Hz, 0.05H), 5.67 – 5.57 (m, 1H), 5.04 (dt, *J* = 10.3, 1.8 Hz, 1H), 3.10 (d, *J* = 17.3 Hz, 1H), 2.83 – 2.69 (m, 2H), 2.04 – 1.89 (m, 3H), 1.82 (dp, *J* = 11.6, 4.3, 3.7 Hz, 1H), 1.71 (ddd, *J* = 13.2, 11.2, 6.2 Hz, 1H), 1.62 – 1.47 (m, 2H), 1.30 – 1.12 (m, 3H), 0.99 (tdd, *J* = 13.7, 7.2, 3.9 Hz, 2H), 0.78 (t, *J* = 7.3 Hz, 3H). **^{13}C NMR** (101 MHz, $CDCl_3$) δ 212.2, 154.3, 138.1, 134.8, 130.2, 128.0, 127.3, 126.5, 123.6, 56.0, 42.0, 36.3, 35.5, 26.1, 25.4, 25.1, 23.5, 22.4, 14.0. **IR (ATR): $\tilde{\nu}$ (cm^{-1})** = 2928, 2857, 1705, 1607, 1464, 1275, 934, 747, 723, 678. **HRMS (ESI) (m/z) calcd. for $C_{19}H_{25}O^+$:** 269.1900; Found: 269.1903. **R_f:** 0.65 (Pentane:EA 10:1).

2-(cyclohex-2-en-1-yl)-2,5-dimethyl-2,3-dihydro-1H-inden-1-one (**4m**):

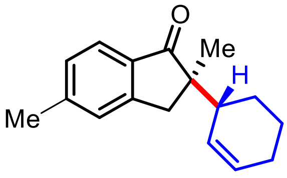

dr = 7.7:1
4m

According to **GP4**, using ketone **1m** and 1,4-cyclohexadiene **2b** as starting materials, **4m** was obtained as colourless oil in 89% yield (21.4 mg) dr =7.7:1. **^{1}H NMR** (400 MHz, $CDCl_3$, the major isomer underlined) δ 7.65 (d, *J* = 7.8 Hz, 1H), 7.22 (s, 1H), 7.17 (d, *J* = 7.3 Hz, 1H), 5.84 (dddd, *J* = 9.9, 4.6, 3.3, 1.7 Hz, 0.12H), 5.74 (dp, *J* = 10.3, 1.8 Hz, 0.12H), 5.69 – 5.60 (m, 0.90H), 5.07 (dp, *J* = 10.2, 1.9 Hz, 0.90H), 3.09 (d, *J* = 17.2 Hz, 1H), 2.77 – 2.57 (m, 1H), 2.43 (s, 3H), 2.07 – 1.90 (m, 3H), 1.89 – 1.77 (m, 1H), 1.60 – 1.47 (m, 1H), 1.29 – 1.27 (m, 0.27H), 1.24 (ddd, *J* = 13.0, 2.9, 2.0 Hz, 0.99H), 1.17 (s, 2.74H), 0.91 – 0.75 (m, 0.21H). **^{13}C NMR** (101 MHz, $CDCl_3$, the major isomer underlined) δ 211.62, 211.00, 154.16, 154.04, 146.08, 146.03, 134.22, 130.26, 130.14, 128.77, 128.70, 128.20, 127.53, 127.12, 126.86, 124.04, 123.99, 52.63, 51.78, 42.55, 42.06, 37.17, 37.02, 25.35, 25.29, 25.19, 25.08, 23.12, 22.64, 22.40, 22.24, 22.20. **IR (ATR): $\tilde{\nu}$ (cm^{-1})** = 2923, 2858, 1703, 1609, 1447, 1432, 1324, 1278, 974, 831, 744, 722, 659. **HRMS (ESI) (m/z) calcd. for $C_{17}H_{21}O^+$:** 241.1587; Found: 241.1589. **R_f:** 0.60 (Pentane:EA 10:1).

2-(cyclohex-2-en-1-yl)-5-fluoro-2-methyl-2,3-dihydro-1H-inden-1-one (**4n**):

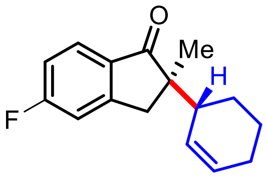

dr = 7.7:1
4n

According to **GP4**, using ketone **1n** and 1,4-cyclohexadiene **2b** as starting materials, **4n** was obtained as colourless oil in 88% yield (21.4 mg) dr = 6.3:1. **^{1}H NMR** (400 MHz, $CDCl_3$, the major isomer underlined) δ 7.76 (dd, *J* = 8.3, 5.4 Hz, 1H), 7.13 – 7.01 (m, 2H), 5.85 (s, 0.13H), 5.75 – 5.61 (m, 1H), 5.05 (dp, *J* = 10.2, 1.9 Hz, 0.92H), 3.13 (d, *J* = 17.4 Hz, 1H), 2.75 – 2.48 (m, 2H), 2.11 – 1.74 (m, 4H), 1.59 – 1.49 (m, 1H), 1.29 (s, 0.26H), 1.27 – 1.20 (m, 1.34H), 1.18 (s, 2.71H), 0.88 – 0.78 (m, 0.25H). **^{13}C NMR** (101 MHz, $CDCl_3$, the major isomer underlined) δ 210.16, 208.72, 168.74, 166.20, 156.53 (d, *J* = 9.9 Hz), 132.87, 130.54, 127.85, 126.46 (d, *J* = 10.6 Hz), 115.77 (d, *J* = 23.8 Hz), 113.35 (d, *J* = 22.1 Hz), 52.96, 42.07, 37.17, 37.15, 25.33, 25.15, 22.57, 22.34. **^{19}F NMR** (376 MHz, $CDCl_3$, the major isomer underlined) δ -103.04, -103.11. **IR (ATR): $\tilde{\nu}$ (cm^{-1})** = 2927, 1710, 1615,

1592, 1483, 1432, 1329, 1290, 1254, 1233, 1085, 976, 859, 660. **HRMS (ESI) (m/z) calcd. for $C_{16}H_{18}FO^+$:** 245.1336; Found: 245.1335. **R_f:** 0.60 (Pentane:EA 10:1).

2-(cyclohex-2-en-1-yl)-6-fluoro-2-methyl-2,3-dihydro-1H-inden-1-one (**4o**):

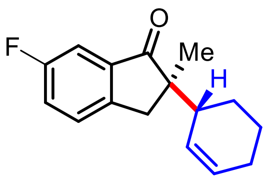

dr = 7.4:1
4o

According to **GP4**, using ketone **1o** and 1,4-cyclohexadiene **2b** as starting materials, **4o** was obtained as colourless oil in 84% yield (20.4 mg) dr =7.4:1. **^{1}H NMR** (400 MHz, $CDCl_3$, the major isomer underlined) δ 7.45 – 7.34 (m, 2H), 7.32 – 7.27 (m, 1H), 5.89 – 5.81 (m, 0.12H), 5.75 – 5.59 (m, 1.02H), 5.05 (dp, J = 10.3, 1.9 Hz, 0.87H), 3.16 – 3.01 (m, 1H), 2.77 – 2.48 (m, 2H), 2.07 – 1.77 (m, 3.85H), 1.65 (dq, J = 9.4, 3.1 Hz, 0.27H), 1.61 – 1.47 (m, 1.08H), 1.28 (s, 0.38H), 1.28 – 1.20 (m, 0.79H), 1.18 (s, 2.79H), 0.88 – 0.76 (m, 0.15H). **^{13}C NMR** (101 MHz, $CDCl_3$, the major isomer underlined) δ 211.18 (d, J = 2.7 Hz), 209.62, 163.68, 163.65, 161.22, 161.19, 149.00 (d, J = 2.1 Hz), 148.88 (d, J = 2.2 Hz), 138.54 (d, J = 7.0 Hz), 138.14 (d, J = 7.0 Hz), 130.50, 128.12 (d, J = 7.9 Hz), 127.92, 127.84, 127.17, 122.59 (d, J = 23.7 Hz), 122.57 (d, J = 23.7 Hz), 109.85 (d, J = 21.6 Hz), 109.80 (d, J = 21.5 Hz). 53.67, 52.82, 42.62, 42.15, 36.85, 36.67, 25.31, 25.24, 25.16, 25.08, 23.01, 22.51, 22.33, 22.15. **^{19}F NMR** (376 MHz, $CDCl_3$, the major isomer underlined) δ -114.68, -114.79. . **IR (ATR): $\tilde{\nu}$ (cm^{-1}) =** 2928, 2860, 1712, 1612, 1484, 1442, 1291, 1264, 1227, 1180, 890, 868, 820, 770, 729. **HRMS (ESI) (m/z) calcd. for $C_{16}H_{18}FO^+$:** 245.1336; Found: 245.1334. **R_f:** 0.60 (Pentane:EA 10:1).

2-octyl-2-(cyclohex-2-en-1-yl)-2,3-dihydro-1H-inden-1-one (**4p**):

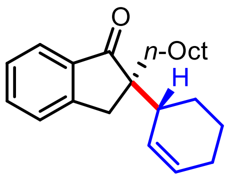

dr = >20:1
4p

According to **GP4**, using ketone **1p** and 1,4-cyclohexadiene **2b** as starting materials, **4p** was obtained as colourless oil in 90% yield (29.3 mg) dr = >20:1. **^{1}H NMR** (400 MHz, $CDCl_3$, the major isomer underlined) δ 7.73 (d, J = 7.6 Hz, 1H), 7.56 (td, J = 7.5, 1.1 Hz, 1H), 7.42 (d, J = 7.7 Hz, 1H), 7.34 (t, J = 7.4 Hz, 1H), 5.85 – 5.79 (m, 0.04H), 5.73 – 5.67 (m, 0.04H), 5.67 – 5.56 (m, 1H), 5.04 (dt, J = 10.3, 1.7 Hz, 1H), 3.09 (d, J = 17.3 Hz, 1H), 2.82 – 2.66 (m, 2H), 1.96 (td, J = 6.5, 5.3, 2.9 Hz, 3H), 1.82 (dt, J = 12.6, 3.6 Hz, 1H), 1.70 (ddd, J = 13.2, 11.2, 5.9 Hz, 1H), 1.61 – 1.46 (m, 2H), 1.30 – 1.10 (m, 11H), 1.07 – 0.92 (m, 2H), 0.83 (t, J = 7.0 Hz, 3H). **^{13}C NMR** (101 MHz, $CDCl_3$) δ 212.2, 154.3, 138.0, 134.8, 130.2, 128.0, 127.3, 126.5, 123.6, 56.1, 42.0, 36.6, 35.6, 31.9, 30.5, 29.5, 29.4, 25.4, 25.1, 24.0, 22.7, 22.4, 14.2. **IR (ATR): $\tilde{\nu}$ (cm^{-1}) =** 2922, 2853, 1706, 1607, 1463, 1433, 1293, 1269, 911, 786, 744, 721, 677, 470. **HRMS (ESI) (m/z) calcd. for $C_{23}H_{33}O^+$:** 325.2526; Found: 325.2528. **R_f:** 0.69 (Pentane:EA 10:1).

2-(cyclohex-2-en-1-yl)-2-phenyl-2,3-dihydro-1H-inden-1-one (**4q**):

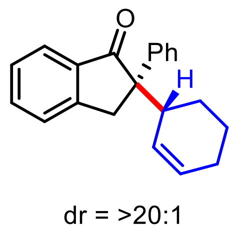

4q

According to **GP4**, using ketone **1q** and 1,4-cyclohexadiene **2b** as starting materials, **4q** was obtained as a pale yellow thick oil in 35% yield (10.1 mg) dr = >20:1.**^{1}H NMR** (400 MHz, $CDCl_3$) δ 7.69 (d, J = 7.7 Hz, 1H), 7.58 (td, J = 7.4, 1.3 Hz, 1H), 7.55 – 7.47 (m, 3H), 7.38 – 7.25 (m, 3H), 7.23 – 7.16 (m, 1H), 5.82 – 5.72 (m, 1H), 5.35 – 5.29 (m, 1H), 3.61 (d, J = 17.4 Hz, 1H), 3.45 – 3.33 (m, 2H), 2.05 – 1.92 (m, 2H), 1.78 – 1.69 (m, 1H), 1.63 – 1.56 (m, 1H), 1.54 – 1.45 (m, 1H), 1.17 – 1.06 (m, 1H). **^{13}C NMR** (101 MHz, $CDCl_3$) δ 207.6, 153.4, 139.5, 136.4, 135.0, 130.8, 128.6, 128.1, 127.6, 127.1, 126.9, 126.2, 124.7, 61.3, 43.1, 34.9, 25.6, 25.5, 22.4. **IR (ATR): ṽ (cm^{-1})** = 3018, 2924, 2858, 1706, 1608, 1495, 1463, 1445, 1327, 1297, 1270, 1222, 1185, 1085, 1034, 922, 783, 762, 739, 721, 698, 648. **HRMS (ESI) (m/z) calcd. for $C_{21}H_{21}O^+$:** 289.1587; Found: 289.1588. **Rf:** 0.50 (Pentane:EA 10:1).

2-(cyclohex-2-en-1-yl)-2-methyl-2,3-dihydro-1H-cyclopenta[a]naphthalen-1-one (**4r**):

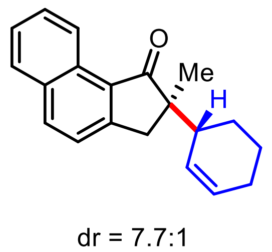

4r

According to **GP4**, using ketone **1r** and 1,4-cyclohexadiene **2b** as starting materials, **4r** was obtained as a colorless thick oil in 54% yield (14.9 mg) dr = 7.7:1. **^{1}H NMR** (400 MHz, $CDCl_3$) δ 9.19 (d, J = 8.3 Hz, 1H), 8.04 (d, J = 8.4 Hz, 1H), 7.89 (d, J = 8.0 Hz, 1H), 7.71 – 7.64 (m, 1H), 7.59 – 7.53 (m, 1H), 7.49 (d, J = 8.4 Hz, 1H), 5.69 – 5.57 (m, 1H), 5.14 (dt, J = 10.2, 2.0 Hz, 1H), 3.23 (d, J = 17.6 Hz, 1H), 2.88 – 2.69 (m, 2H), 2.10 – 1.79 (m, 4H), 1.67 – 1.55 (m, 1H), 1.39 – 1.31 (m, 1H), 1.26 (s, 3H). **^{13}C NMR** (101 MHz, $CDCl_3$) δ 212.6, 157.0, 135.9, 132.9, 130.3, 130.2, 129.7, 129.0, 128.2, 128.1, 126.6, 124.3, 124.3, 52.8, 42.2, 37.7, 25.4, 25.3, 22.7, 22.4. **IR (ATR): ṽ (cm^{-1})** = 3017 2924, 2857, 1693, 1628, 1593, 1573, 1517, 1439, 1370, 1302, 1216, 1197, 1172, 1151, 1090, 1070, 1028, 900, 820, 794, 748, 722, 676, 627, 514, 496. **HRMS (ESI) (m/z) calcd. for $C_{20}H_{21}O^+$:** 277.1587; Found: 277.1587. **R_f:** 0.55 (Pentane:EA 10:1).

General procedure 5 (GP5)

In a glovebox, an oven dried screw-capped 2 mL vial was charged with a magnetic stir bar, Ni(^{tBu}stb)$_3$ (10 μmol), carbene ligand ^{Me}IPr^{tBu} (10 μmol), freshly distilled and degassed (freeze pump thaw) toluene (0.1 mL) was then added and the catalyst mixture was stirred at room temperature for 30 min, then 1,4-cyclohexadiene (0.2 mmol) was then added during which a dark solution is formed. The mixture was stirred at room temperature for 10 minutes, ketone (0.1 mmol) was then added successively. The vial was sealed with a Teflon-lined screw cap, and running inside the glovebox at 40 ^{o}C After 72 h, the vial was shipped outside of the glovebox. The reaction mixture was diluted with dichloromethane and filtered through a plug of silica gel. The crude solution was concentrated in vacuum and subjected to column chromatography to isolate the products.

Note: Liquid reagents/reactants were degassed by freeze-pump-thaw (3×) before being stored in the glovebox at -30 °C. Solid reagents/reactants were dried under high vacuum for 16h before being stored in the glovebox at -30 °C.

2-(cyclohex-2-en-1-yl)-2-phenyl-3,4-dihydronaphthalen-1(2H)-one (**4s**):

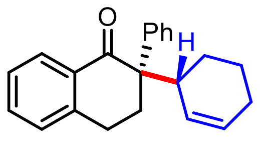

dr = >20:1
4s

According to **GP5**, using ketone **1s** and 1,4-cyclohexadiene **2b** as starting materials, **4s** was obtained as a white solid in 64% yield (19.5 mg) dr = >20:1. ^{1}**H NMR** (400 MHz, $CDCl_3$) δ 8.07 (dd, J = 7.9, 1.6 Hz, 1H), 7.34 – 7.08 (m, 7H), 7.01 (d, J = 7.7 Hz, 1H), 5.68 – 5.60 (m, 1H), 5.40 (dt, J = 10.3, 1.9 Hz, 1H), 3.16 (ddt, J = 13.8, 5.3, 2.7 Hz, 1H), 3.04 (ddd, J = 17.6, 13.2, 4.5 Hz, 1H), 2.80 (ddd, J = 17.2, 4.7, 2.5 Hz, 1H), 2.55 (ddd, J = 14.6, 4.5, 2.6 Hz, 1H), 2.29 (ddd, J = 14.6, 13.2, 4.6 Hz, 1H), 1.94 – 1.81 (m, 2H), 1.69 – 1.57 (m, 1H), 1.44 – 1.30 (m, 1H), 1.21 – 1.10 (m, 1H), 1.05 – 0.93 (m, 1H). ^{13}**C NMR** (101 MHz, $CDCl_3$) δ 199.2, 143.3, 137.0, 133.2, 130.0, 128.7, 128.6, 128.5, 128.4, 127.8, 127.0, 126.5, 56.4, 44.0, 25.8, 25.5, 23.9, 22.9. **IR (ATR): $\tilde{\nu}$ (cm^{-1})** = 3022, 2924, 2858, 2836, 1673, 1600, 1494, 1454, 1446, 1431, 1292, 1224, 1156, 1123, 927, 910, 894, 782, 771, 759, 741, 716, 701, 667, 637, 618, 547. **HRMS (ESI) (m/z) calcd. for $C_{22}H_{23}O^+$:** 303.1743; Found: 303.1746. **m. p.:** 121-122 °C. **R_f:** 0.65 (Pentane:EA 10:1).

2-cyclohex-2-en-1-yl)-2-(p-tolyl)-3,4-dihydronaphthalen-1(2H)-one (**4t**):

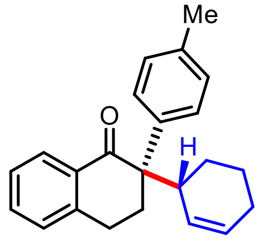

dr = >20:1
4t

According to **GP5**, using ketone **1t** and 1,4-cyclohexadiene **2b** as starting materials, **4t** was obtained as a colourless thick oil in 53% yield (16.8 mg) dr = >20:1.^{1}**H NMR** (400 MHz, $CDCl_3$) δ 8.14 (dd, J = 7.9, 1.6 Hz, 1H), 7.36 (td, J = 7.4, 1.5 Hz, 1H), 7.27 – 7.18 (m, 3H), 7.09 (t, J = 7.7 Hz, 3H), 5.71 (dt, J = 10.2, 3.8 Hz, 1H), 5.47 (dt, J = 10.3, 1.9 Hz, 1H), 3.20 (dq, J = 8.4, 2.6 Hz, 1H), 3.11 (ddd, J = 17.4, 13.2, 4.5 Hz, 1H), 2.86 (ddd, J = 17.3, 4.9, 2.6 Hz, 1H), 2.64 – 2.55 (m, 1H), 2.34 (ddd, J = 14.6, 13.1, 4.7 Hz, 1H), 2.28 (s, 3H), 2.00 – 1.89 (m, 2H), 1.70 (dt, J = 12.9, 3.5 Hz, 1H), 1.51 – 1.40 (m, 1H), 1.28 – 1.18 (m, 1H), 1.13 – 1.03 (m, 1H). ^{13}**C NMR** (101 MHz, $CDCl_3$) δ 199.3, 143.3, 136.7, 133.9, 133.2, 133.1, 130.2, 129.2, 128.6, 128.4, 127.7, 126.4, 56.1, 44.0, 25.8, 25.7, 25.5, 23.9, 22.9, 21.0. **IR (ATR): $\tilde{\nu}$ (cm^{-1})** = 3021, 2926, 2860, 2836, 1675, 1600, 1512, 1486, 1453, 1431, 1356, 1338, 1302, 1292, 1261, 1223, 1194, 1156, 1144, 1124, 1104, 1091, 1060, 1018, 962, 926, 894, 883, 844, 833, 802, 780, 759, 741, 722, 681, 654, 610, 560, 547, 526, 517, 490, 474, 456, 437, 418. **HRMS (ESI) (m/z) calcd. for $C_{23}H_{25}O^+$:** 317.1900; Found: 317.1901. **R_f:** 0.67 (Pentane:EA 10:1).

(2-cyclohex-2-en-1-yl)-2-(m-tolyl)-3,4-dihydronaphthalen-1(2H)-one (**4u**):

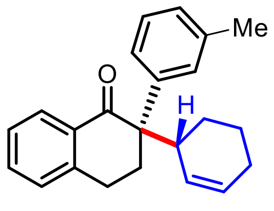

According to **GP5**, using ketone **1u** and 1,4-cyclohexadiene **2b** as starting materials, **4u** was obtained as a white solid in 58% yield (18.3 mg) dr = >20:1. **^{1}H NMR** (400 MHz, $CDCl_3$) δ 8.15 (d, J = 9.3 Hz, 1H), 7.37 (td, J = 7.5, 1.5 Hz, 1H), 7.29 – 7.05 (m, 5H), 7.01 (d, J = 7.0 Hz, 1H), 5.76 – 5.65 (m, 1H), 5.51 – 5.41 (m, 1H), 3.25 – 3.06 (m, 2H), 2.92 – 2.81 (m, 1H), 2.66 – 2.56 (m, 1H), 2.40 – 2.27 (m, 4H), 1.99 – 1.91 (m, 2H), 1.76 – 1.66 (m, 1H), 1.51 – 1.39 (m, 1H), 1.27 – 1.17 (m, 1H), 1.11 – 1.03 (m, 1H). **^{13}C NMR** (101 MHz, $CDCl_3$) δ 199.2, 143.4, 138.1, 136.9, 133.2, 133.2, 130.2, 128.6, 128.4, 128.2, 127.8, 126.4, 124.9, 56.4, 44.0, 25.8, 25.8, 25.5, 23.9, 22.9, 21.8. **IR (ATR): ṽ (cm^{-1})** = 3022, 2924, 2859, 2835, 1673, 1600, 1487, 1453, 1432, 1301, 1228, 1219, 1155, 1122, 928, 907, 893, 870, 817, 784, 756, 740, 729, 701, 686, 640, 619, 500. **HRMS (ESI) (m/z) calcd. for $C_{23}H_{25}O^+$:** 317.1900; Found: 317.1905. **m. p.:** 134-135 °C. **R_f:** 0.67 (Pentane:EA 10:1).

(2-cyclohex-2-en-1-yl)-2-(4-fluorophenyl)-3,4-dihydronaphthalen-1(2H)-one (**4v**):

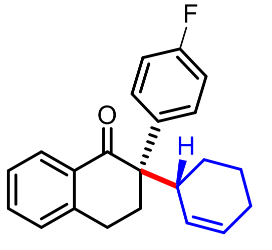

According to **GP5**, using ketone **1v** and 1,4-cyclohexadiene **2b** as starting materials, **4v** was obtained as a white solid in 68% yield (21.9 mg) dr = >20:1. **^{1}H NMR** (400 MHz, $CDCl_3$) δ 8.14 (dd, J = 7.9, 1.6 Hz, 1H), 7.39 (td, J = 7.4, 1.6 Hz, 1H), 7.35 – 7.22 (m, 3H), 7.10 (d, J = 7.7 Hz, 1H), 7.03 – 6.93 (m, 2H), 5.78 – 5.64 (m, 1H), 5.52 – 5.40 (m, 1H), 3.28 – 3.15 (m, 1H), 3.12 – 2.99 (m, 1H), 2.93 – 2.83 (m, 1H), 2.64 – 2.52 (m, 1H), 2.43 – 2.31 (m, 1H), 2.01 – 1.89 (m, 2H), 1.77 – 1.66 (m, 1H), 1.52 – 1.37 (m, 1H), 1.27 – 1.15 (m, 1H), 1.10 – 1.00 (m, 1H). **^{13}C NMR** (101 MHz, $CDCl_3$) δ 199.1, 162.0 (d, J = 246.1 Hz), 143.1, 133.4, 133.0, 132.7 (d, J = 3.3 Hz), 129.8, 129.4 (d, J = 7.7 Hz), 128.9, 128.6, 128.4, 126.6, 115.3 (d, J = 20.9 Hz), 55.9, 44.0, 25.8, 25.6, 25.4, 23.8, 22.8. ^{19}F NMR (376 MHz, $CDCl_3$) δ -115.9. **IR (ATR): ṽ (cm^{-1})** = 3023, 2931, 2859, 2836, 1673, 1600, 1508, 1454, 1432, 1305, 1292, 1225, 1167, 1144, 1120, 1014, 927, 894, 884, 842, 811, 780, 761, 739, 722, 653, 609, 549, 468. **HRMS (ESI) (m/z) calcd. for $C_{22}H_{22}FO^+$:** 321.1649; Found: 321.1653. **m. p.:** 127-128 °C. **R_f:** 0.70 (Pentane:EA 10:1).

(2-cyclohex-2-en-1-yl)-2-(4-methoxyphenyl)-3,4-dihydronaphthalen-1(2H)-one (**4w**):

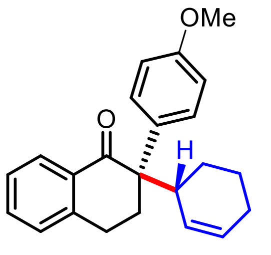

According to **GP5**, using ketone **1w** and 1,4-cyclohexadiene **2b** as starting materials, **4w** was obtained as a white solid in 50% yield (16.7 mg) dr = >20:1. **^{1}H NMR** (400 MHz, $CDCl_3$) δ 8.14 (d, J = 7.9 Hz, 1H), 7.40 – 7.33 (m, 1H), 7.28 – 7.20 (m, 3H), 7.08 (d, J = 7.7 Hz, 1H), 6.86 – 6.78 (m, 2H), 5.75 – 5.65 (m, 1H), 5.47 (d, J = 10.4 Hz, 1H), 3.75 (s, 3H), 3.26 – 3.15 (m, 1H), 3.09 (ddd, J = 17.4, 13.2, 4.5 Hz, 1H), 2.91 – 2.81 (m, 1H), 2.57 (ddd, J = 14.6, 4.5, 2.5 Hz, 1H), 2.41 – 2.26 (m, 1H), 2.00 – 1.89 (m, 2H), 1.75 – 1.66 (m, 1H), 1.51 – 1.39 (m, 1H), 1.25 – 1.15 (m, 1H), 1.13 – 1.04 (m, 1H). **^{13}C NMR** (101 MHz, $CDCl_3$) δ 199.3, 158.6, 143.3, 133.2, 130.1, 128.9, 128.8, 128.6, 128.4, 126.4, 113.8, 55.8, 55.3, 44.0, 25.8, 25.8, 25.5, 23.9, 22.9. **IR (ATR): ṽ (cm^{-1})** = 3021, 2929, 2859, 2835, 1672, 1600, 1579, 1510, 1486, 1454, 1307, 1287, 1252, 1224, 1185, 1156, 1144,

1123, 1090, 1061, 1038, 926, 910, 894, 837, 810, 779, 759, 738, 654, 612, 552. **HRMS (ESI) (m/z) calcd. for $C_{23}H_{25}O_2^+$:** 333.1849; Found: 333.1849. **m. p.:** 144-145 °C. **R_f:** 0.40 (Pentane:EA 10:1).

(2-cyclohex-2-en-1-yl)-3,4-dihydro-[2,2'-binaphthalen]-1(2H)-one (**4x**):

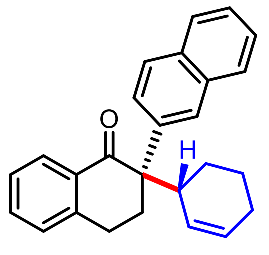

dr = >20:1
4x

According to **GP5**, using ketone **1x** and 1,4-cyclohexadiene **2b** as starting materials, **4x** was obtained as a colourless thick oil in 24% yield (8.6 mg) dr = >20:1. **^{1}H NMR** (400 MHz, $CDCl_3$) δ 8.19 (dd, J = 7.9, 1.6 Hz, 1H), 7.83 – 7.69 (m, 4H), 7.54 (dd, J = 8.8, 2.0 Hz, 1H), 7.47 – 7.39 (m, 2H), 7.35 (td, J = 7.4, 1.6 Hz, 1H), 7.28 – 7.22 (m, 1H), 7.07 (d, J = 7.7 Hz, 1H), 5.80 – 5.68 (m, 1H), 5.53 (dt, J = 10.3, 1.9 Hz, 1H), 3.41 – 3.27 (m, 1H), 3.23 – 3.10 (m, 1H), 2.97 – 2.87 (m, 1H), 2.82 – 2.70 (m, 1H), 2.46 (ddd, J = 14.6, 13.2, 4.7 Hz, 1H), 2.03 – 1.89 (m, 2H), 1.73 – 1.63 (m, 1H), 1.50 – 1.37 (m, 1H), 1.35 – 1.24 (m, 1H), 1.15 – 1.03 (m, 1H). **^{13}C NMR** (101 MHz, $CDCl_3$) δ 199.2, 143.2, 134.7, 133.4, 133.3, 133.2, 132.4, 130.0, 128.8, 128.6, 128.5, 128.3, 127.9, 127.4, 127.0, 126.5, 126.1, 126.1, 125.7, 56.6, 44.0, 26.0, 25.9, 25.5, 24.0, 22.9. **IR (ATR): $\tilde{\nu}$ (cm^{-1})** = 3022, 2928, 2875, 2859, 2835, 1673, 1599, 1505, 1454, 1432, 1296, 1276, 1223, 1155, 1122, 926, 907, 862, 810, 739, 664, 478. **HRMS (ESI) (m/z) calcd. for $C_{26}H_{25}O^+$:** 353.1900; Found: 353.1890. **R_f:** 0.45 (Pentane:EA 10:1).

(2-cyclohex-2-en-1-yl)-7-methyl-2-phenyl-3,4-dihydronaphthalen-1(2H)-one (**4y**):

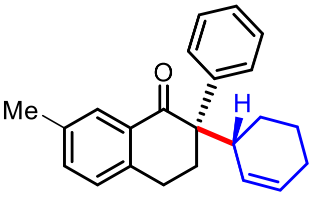

dr = >20:1
4y

According to **GP5**, using ketone **1y** and 1,4-cyclohexadiene **2b** as starting materials, **4y** was obtained as a white solid in 71% yield (22.5 mg) dr = >20:1. **^{1}H NMR** (400 MHz, $CDCl_3$) δ 7.96 (s, 1H), 7.37 – 7.25 (m, 4H), 7.23 – 7.16 (m, 2H), 6.98 (d, J = 7.8 Hz, 1H), 5.77 – 5.66 (m, 1H), 5.52 – 5.43 (m, 1H), 3.23 (dt, J = 5.9, 2.9 Hz, 1H), 3.06 (ddd, J = 17.4, 13.1, 4.5 Hz, 1H), 2.83 (ddd, J = 17.1, 4.8, 2.6 Hz, 1H), 2.61 (ddd, J = 14.6, 4.5, 2.5 Hz, 1H), 2.40 – 2.28 (m, 4H), 2.00 – 1.90 (m, 2H), 1.74 – 1.65 (m, 1H), 1.51 – 1.38 (m, 1H), 1.28 – 1.17 (m, 1H), 1.11 – 1.00 (m, 1H). **^{13}C NMR** (101 MHz, $CDCl_3$) δ 199.4, 140.4, 137.1, 136.1, 134.3, 133.0, 130.1, 128.7, 128.6, 128.4, 128.4, 127.8, 127.0, 56.4, 44.0, 25.8, 25.5, 25.3, 23.9, 22.9, 21.1. **IR (ATR): $\tilde{\nu}$ (cm^{-1})** = 3021, 2923, 2859, 2835, 2249, 1672, 1611, 1578, 1496, 1445, 1432, 1409, 1380, 1355, 1306, 1283, 1241, 1197, 1168, 1142, 1120, 1090, 1079, 1062, 1039, 1024, 1001, 948, 907, 881, 814, 781, 771, 759, 726, 710, 700, 667, 644, 615, 566, 531, 491, 469, 453, 433, 417. **HRMS (ESI) (m/z) calcd. for $C_{23}H_{25}O^+$:** 317.1900; Found: 317.1894. **m. p.:** 168-171 °C. **R_f:** 0.67 (Pentane:EA 10:1).

(2-cyclohex-2-en-1-yl)-7-fluoro-2-(4-methoxyphenyl)-3,4-dihydronaphthalen-1(2H)-one(**4z**):

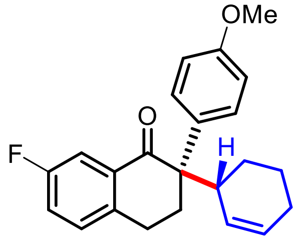

dr = >20:1
4z

According to **GP5**, using ketone **1z** and 1,4-cyclohexadiene **2b** as starting materials, **4z** was obtained as a white solid in 82% yield (28.9 mg) dr = >20:1. **^{1}H NMR** (400 MHz, $CDCl_3$) δ 7.83 – 7.75 (m, 1H), 7.24 – 7.19 (m, 2H), 7.10 – 7.02 (m, 2H), 6.86 – 6.80 (m, 2H), 5.75 – 5.67 (m, 1H), 5.43 (dt, J = 10.3, 1.9 Hz, 1H), 3.75 (s, 3H), 3.24 – 3.14 (m, 1H), 3.09 – 2.98 (m, 1H), 2.88 – 2.79 (m, 1H), 2.61 – 2.53 (m, 1H), 2.37 – 2.27 (m, 1H), 2.00 – 1.90 (m, 2H), 1.75 – 1.67 (m, 1H), 1.50 – 1.38 (m, 1H), 1.23 – 1.16 (m, 1H), 1.13 – 1.05 (m, 1H). **^{13}C NMR** (101 MHz, $CDCl_3$) δ 198.3 (d, J = 1.8 Hz), 161.5 (d, J = 245.4 Hz), 158.7, 138.8, 134.7 (d, J = 5.9 Hz), 130.4 (d, J = 7.0 Hz), 129.8, 128.8, 128.8, 128.3, 120.6 (d, J = 22.4 Hz), 114.2, 113.9, 113.9, 55.5, 55.3, 43.9, 25.8, 25.5, 25.0, 23.9, 22.9. **^{19}F NMR** (376 MHz, $CDCl_3$) δ -115.7. **IR (ATR): $\tilde{\nu}$ (cm^{-1})** = 3021, 2931, 2860, 2836, 1677, 1607, 1589, 1579, 1510, 1491, 1460, 1432, 1420, 1355, 1306, 1286, 1270, 1244, 1186, 1168, 1154, 1125, 1061, 1037, 904, 887, 824, 799, 766, 731, 723, 707, 655, 612, 561, 543. **HRMS (ESI) (m/z) calcd. for $C_{23}H_{24}FO_2^+$:** 351.1755; Found: 351.1756. **m. p.:** 157-158 °C. **R$_f$:** 0.45 (Pentane:EA 10:1).

2-(cyclohex-2-en-1-yl)-2-methyl-3,4-dihydronaphthalen-1(2H)-one (**4aa**):

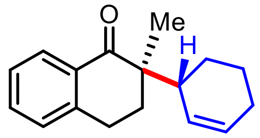

dr = 5.0:1
4aa

Following a slight modification of **GP5**, using ketone **1aa** and 1,4-cyclohexadiene **2b** as starting materials at 80 °C. After 24 hours, **4aa** was obtained as a colourless oil in 62% yield (14.8 mg) dr = 5.0:1. **^{1}H NMR** (400 MHz, $CDCl_3$, the major isomer underlined) δ 8.08 – 7.98 (m, 1H), 7.50 – 7.41 (m, 1H), 7.30 (t, *J* = 7.6 Hz, 1H), 7.21 (d, *J* = 7.8 Hz, 1H), 5.82 – 5.68 (m, 1H), 5.53 – 5.46 (m, 0.17H), 5.46 – 5.36 (m, 0.87H), 3.12 – 2.78 (m, 3H), 2.70 – 2.60 (m, 0.17H), 2.27 – 2.09 (m, 1H), 2.03 – 1.72 (m, 4H), 1.60 – 1.43 (m, 2H), 1.42 – 1.23 (m, 1H), 1.17 – 1.06 (m, 3H). **^{13}C NMR** (101 MHz, $CDCl_3$, , the major isomer underlined) δ 202.9, 143.4, 133.1, 132.4, 129.4, 128.7, 128.2, 128.1, 126.8, 47.6, 47.4, 39.2, 31.5, 30.4, 25.3, 25.3, 23.6, 22.7, 19.4. **IR (ATR): $\tilde{\nu}$ (cm^{-1})** = 3021, 2930, 2860, 2836, 1677, 1600, 1454, 1434, 1374, 1353, 1306, 1291, 1260, 1220, 1153, 1095, 1047, 1023, 1004, 980, 954, 915, 900, 797, 772, 742, 723, 656, 619, 608, 492. **HRMS (ESI) (m/z) calcd. for $C_{17}H_{21}O^+$:** 241.1587; Found: 241.1590. **R$_f$:** 0.50 (Pentane:EA 10:1).

2-(cyclohex-2-en-1-yl)-1-phenylethan-1-one (**4ab**):

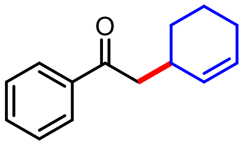

4ab

Following a slight modification of **GP5**, using ketone **1ab** and 1,4-cyclohexadiene **2b** as starting materials at 40 °C. After 24 hours, **4ab** was obtained as a colourless oil in 20% yield (4.0 mg) **^{1}H NMR** (400 MHz, $CDCl_3$) δ 7.99 – 7.93 (m, 2H), 7.59 – 7.53 (m, 1H), 7.49 – 7.42 (m, 2H), 5.77 – 5.68 (m, 1H), 5.64 – 5.53 (m, 1H), 3.02 – 2.89 (m, 2H), 2.88 – 2.74 (m, 1H), 2.04 – 1.96 (m, 2H), 1.91 – 1.83 (m, 1H), 1.76 – 1.67 (m, 1H), 1.64 – 1.57 (m, 1H), 1.36 – 1.29 (m, 1H). **^{13}C NMR** (101 MHz, $CDCl_3$) δ 199.8, 137.5, 133.1, 130.9, 128.7, 128.3, 128.1, 45.0, 31.8, 29.2, 25.3, 21.2. data matches with the literature[19].

2-(cyclohex-2-en-1-yl)-1-phenylpropan-1-one (**4ac**):

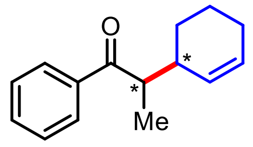

4ac dr = 1:1

Following a slight modification of **GP5**, using ketone **1ac** and 1,4-cyclohexadiene **2b** as starting materials at 80 °C. After 24 hours, **4ac** was obtained as a colourless oil in 44% yield (9.4 mg) dr = 1:1. **^{1}H NMR** (400 MHz, $CDCl_3$) δ 7.96 – 7.79 (m, 2H), 7.54 – 7.45 (m, 1H), 7.44 – 7.34 (m, 2H), 5.77 – 5.67 (m, 0.5H), 5.66 – 5.55 (m, 1H), 5.45 – 5.37 (m, 0.5H), 3.44 – 3.30 (m, 1H), 2.62 – 2.45 (m, 1H), 1.95 – 1.84 (m, 2H), 1.76 – 1.58 (m, 2H), 1.47 – 1.38 (m, 1H), 1.33 – 1.21 (m, 1H), 1.12 (d, *J* = 6.9 Hz, 1.5H), 1.08 (d, *J* = 6.9 Hz, 1.5H). **^{13}C NMR** (101 MHz, $CDCl_3$) δ 204.5, 137.3, 137.3, 133.0, 133.0, 130.2, 129.2, 128.8, 128.8, 128.6, 128.4, 128.4, 128.2, 45.5, 45.0, 38.4, 38.1, 28.1, 25.3, 25.2, 21.8, 21.8, 14.2, 13.8. **IR (ATR): $\tilde{\nu}$ (cm^{-1})** = 3022, 2930, 2859, 2837, 1680, 1597, 1579, 1447, 1359, 1286, 1253, 1212, 1183, 1077, 992, 970, 881, 793, 762, 711, 689, 670, 654, 420. **HRMS (ESI) (m/z) calcd. for $C_{15}H_{19}O^+$:** 215.1430; Found: 215.1428. **R_f:** 0.60 (Pentane:EA 10:1).

2-(cyclohex-2-en-1-yl)-3,3-dimethyl-2,3-dihydro-1H-inden-1-one (**4ad**):

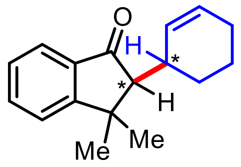

dr = 1.2:1
4ad

Following a slight modification of **GP5**, using ketone **1ad** and 1,4-cyclohexadiene **2b** as starting materials at 80 °C. After 24 hours, **4ad** was obtained as a colourless oil in 86% yield (20.6 mg) dr = 1.2:1. **^{1}H NMR** (400 MHz, $CDCl_3$) δ 7.73 – 7.64 (m, 1H), 7.63 – 7.55 (m, 1H), 7.49 – 7.45 (m, 1H), 7.39 – 7.30 (m, 1H), 5.74 – 5.56 (m, 1.54H), 5.44 – 5.35 (m, 0.54H), 3.01 – 2.91 (m, 0.49H), 2.79 – 2.68 (m, 0.56H), 2.49 (d, *J* = 4.6 Hz, 0.48H), 2.42 (d, *J* = 3.9 Hz, 0.56H), 2.11 – 1.91 (m, 2H), 1.89 – 1.72 (m, 2H), 1.61 – 1.49 (m, 2H), 1.46 (s, 1.5H), 1.41 (s, 1.5H), 1.40 – 1.37 (m, 3H). **^{13}C NMR** (101 MHz, $CDCl_3$) δ 207.6, 207.2, 163.4, 162.9, 135.6, 135.2, 134.9, 134.8, 130.8, 128.8, 128.7, 127.7, 127.5, 127.4, 123.3, 123.0, 63.8, 63.4, 42.9, 42.4, 36.1, 36.0, 32.7, 31.9, 28.7, 27.4, 26.0, 25.2, 24.9, 22.8, 22.8. **IR (ATR): $\tilde{\nu}$ (cm^{-1})** = 3023, 2957, 2928, 2861, 2836, 1709, 1604, 1460, 1366, 1324, 1287, 1269, 1220, 947, 765, 740, 719, 663. **HRMS (ESI) (m/z) calcd. for $C_{17}H_{21}O^+$:** 241.1587; Found: 241.1590. **R_f:** 0.65 (Pentane:EA 10:1).

2-((1-benzylpiperidin-4-yl)methyl)-2-(cyclohex-2-en-1-yl)-5,6-dimethoxy-2,3-dihydro-1H-inden-1-one (**4ae**):

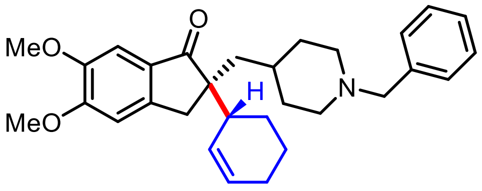

dr = 3:1
4ae

Following a slight modification of **GP5**, using ketone **1aa** and 1,4-cyclohexadiene **2b** as starting materials at 100 °C. After 24 hours, **4ae** was obtained as a colourless oil in 33% yield (15.3 mg) dr = 3:1. **^{1}H NMR** (400 MHz, CD_2Cl_2) δ 7.46 – 7.34 (m, 1H), 7.32 – 7.17 (m, 4H), 7.14 – 7.04 (m, 1H), 6.84 (s, 1H), 5.71 – 5.57 (m, 1H), 5.07 – 4.94 (m, 1H), 3.91 (s, 3H), 3.86 (s, 3H), 3.34 (d, *J* = 2.9 Hz, 1H), 2.94 (d, *J* = 17.2 Hz, 1H), 2.76 – 2.53 (m, 5H), 2.02 – 1.89 (m, 4H), 1.87 – 1.59 (m, 5H), 1.56 – 1.32 (m, 3H), 1.23 – 1.05 (m, 4H). **^{13}C NMR** (101 MHz, CD_2Cl_2, major isomer underlined) δ 210.2, 209.9, 156.1, 149.9, 149.6, 139.2, 133.2, 131.2, 131.0, 130.6, 130.1, 129.5,

128.9, 128.4, 128.0, 127.6, 127.2, 107.9, 107.7, 104.2, 63.5, 63.0, 56.4, 56.3, 56.2, 43.7, 43.5, 43.1, 42.4, 35.5, 34.6, 34.4, 32.6, 30.6, 30.1, 28.5, 28.0, 25.6, 25.6, 25.5, 25.4, 22.8, 22.7. **IR (ATR): ṽ (cm^{-1})** = 2922, 2855, 1692, 1605, 1590, 1500, 1455, 1440, 1421, 1365, 1342, 1306, 1266, 1219, 1190, 1112, 1050, 1030, 979, 864, 782, 740, 721, 700. **HRMS (ESI/QTOF) (m/z): calcd for $C_{30}H_{38}NO_3^+$**: 460.2846; Found: 460.2856. **R$_f$:** 0.30 (Pentane:EA 1:1).

(*E*)-2-methyl-2-(2-phenylpent-2-en-1-yl)-2,3-dihydro-1H-inden-1-one (**4af**):

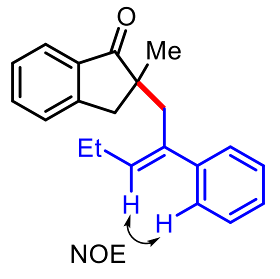

E/Z = 9:1
4af

Following a slight modification of **GP5**, using ketone **1ac** and olefin **2c** as starting materials at 80 °C. After 24 hours, **4ae** was obtained as a colourless oil in 36% yield (10.5 mg) E/Z = 9:1. **^{1}H NMR** (400 MHz, $CDCl_3$, major isomer underlined) δ 7.55 (d, *J* = 7.5 Hz, 1H), 7.49 (td, *J* = 7.5, 1.4 Hz, 1H), 7.30 – 7.24 (m, 1H), 7.23 – 7.19 (m, 1H), 7.19 – 7.12 (m, 3H), 7.09 – 7.02 (m, 1.86H), 6.94 – 6.80 (m, 0.17H), 5.53 (t, *J* = 7.3 Hz, 1H), 2.96 – 2.87 (m, 2H), 2.82 (d, *J* = 13.9 Hz, 1H), 2.53 (d, *J* = 17.2 Hz, 1H), 2.21 (pd, *J* = 7.4, 2.4 Hz, 2H), 1.93 – 1.80 (m, 0.12H), 1.14 (s, 3H), 1.03 (t, *J* = 7.5 Hz, 2.82H), 0.85 (t, *J* = 7.5 Hz, 0.32H). **^{13}C NMR** (101 MHz, $CDCl_3$) δ 211.0, 152.8, 144.6, 136.8, 135.9, 135.6, 134.7, 128.1, 127.2, 127.1, 126.7, 126.4, 124.2, 50.0, 40.0, 37.5, 24.6, 22.6, 14.4. **IR (ATR): ṽ (cm^{-1})** = 3027, 2962, 2929, 2871, 1713, 1608, 1492, 1464, 1372, 1326, 1297, 1206, 1185, 1153, 1090, 1032, 978, 869, 799, 774, 736, 700, 545, 474, 428, 400. **HRMS (ESI/QTOF) (m/z): calcd for $C_{21}H_{23}O^+$**: 291.1743; Found: 291.1742.

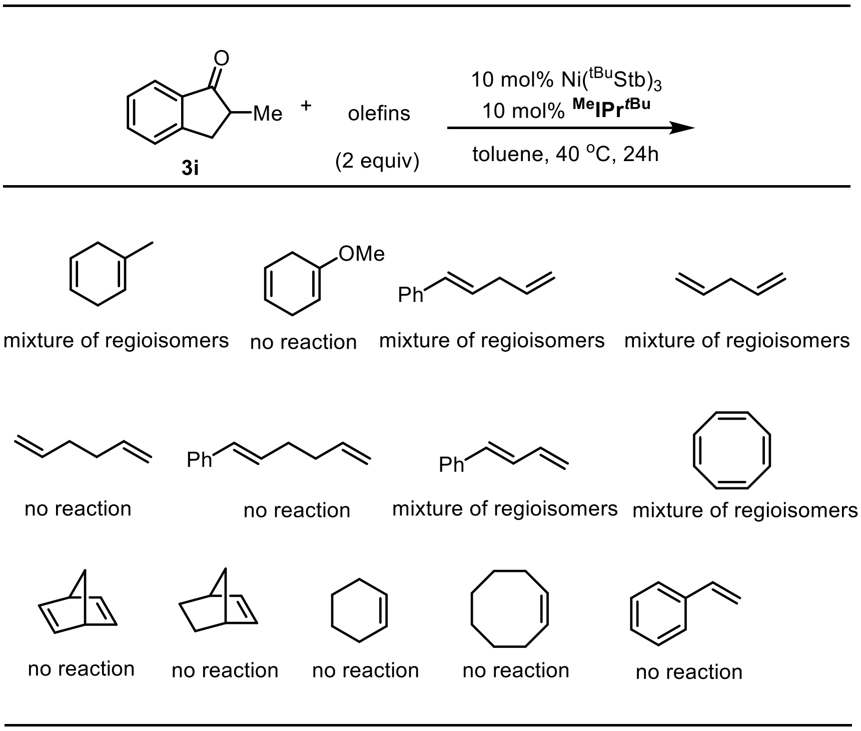

Supplementary Fig 4.4 | Unsuccessful dienes.

4.4 Optimization of enantioselective hydro-alkylation of non-conjugated dienes[a].

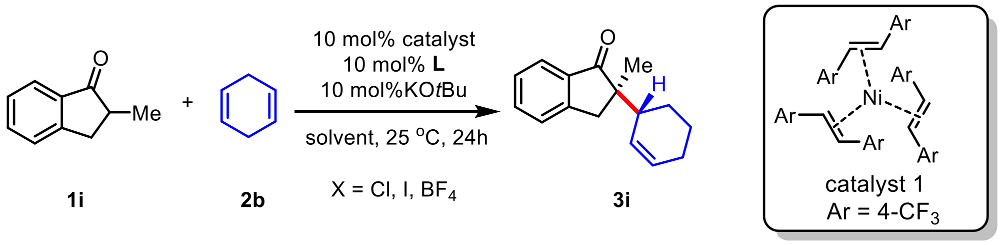

Chiral NHC ligands:

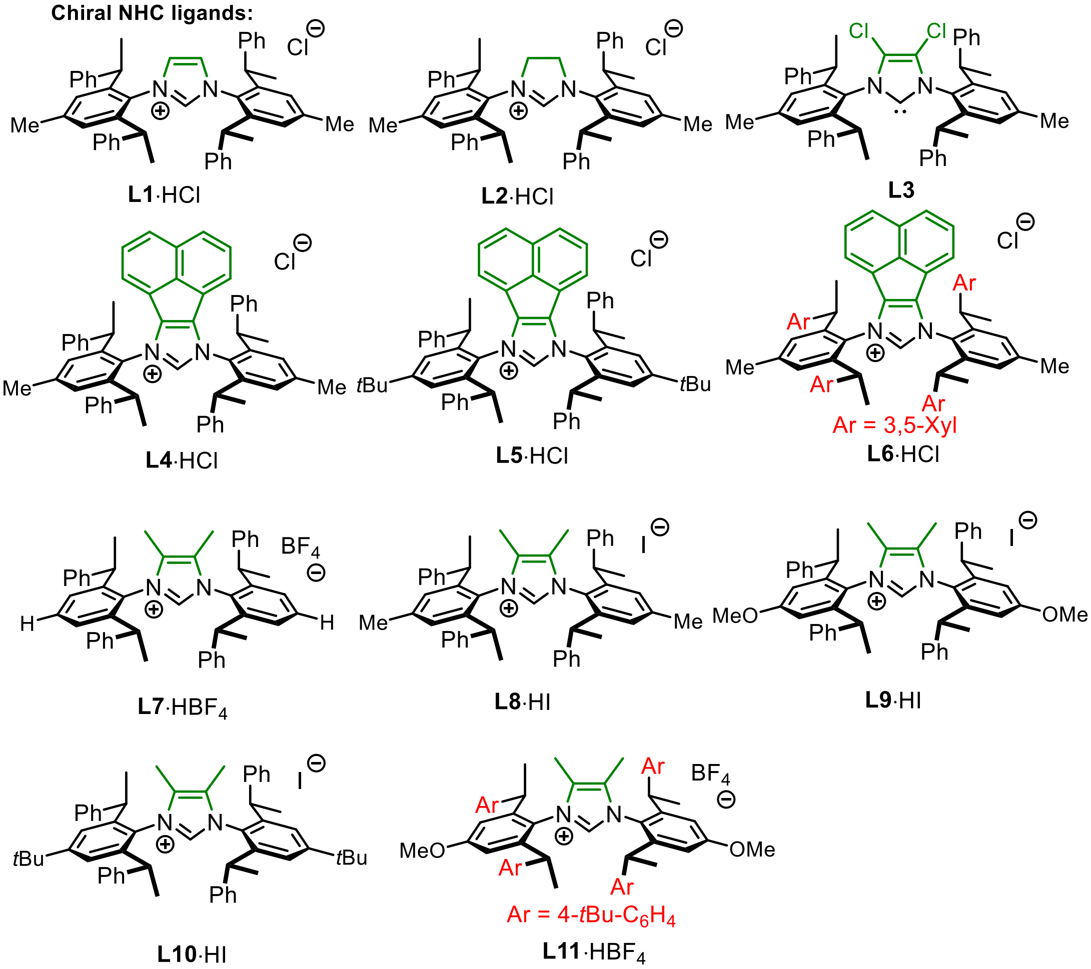

Entry	solvent	L	% Conv.[b]	% 3i[b]	dr of 3i[b]	er of 3i[c]
1	toluene	L1·HCl	40	40	10:1	69:31
2	toluene	L2·HCl	57	52	9:1	60:40
3	toluene	L3	0	0	-	-
4	toluene	L4·HCl	40	38	10:1	85:15
5	toluene	L5·HCl	55	55	11:1	75:25
6	toluene	L6·HCl	0	0	-	-
7	CPME	L7·HBF$_4$	30	29	15:1	88:12
8	CPME	L8·HI	32	31	16:1	91:9
9	CPME	L9·HI	35	35	10:1	90:10

**10**	**CPME**	**L10**·HI	**30**	**30**	**15:1**	**87:13**
**11**	**CPME**	**L11**·HBF_4	**48**	**48**	**16:1**	**92:8**

[a] 0.1 mmol **1i**, 0.2 mmol **2b**, 10 μmol Ni catalyst, 10 μmol ligand and 10 μmol KO*t*Bu at 25 °C in solvent (1 M) for 24 h; [b] determined by ^{1}H NMR using dibromomethane as internal standard.[c] determined by HPLC.

Supplementary Fig 4.5 | Optimization of nickel NHC* catalyzed hydro-alkylation of 1,4-cyclohexadiene.

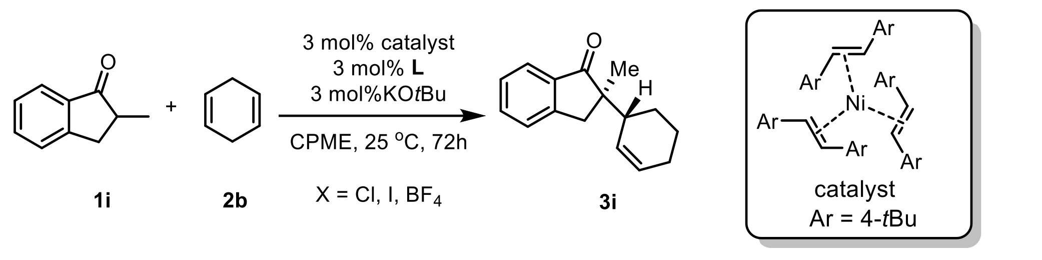

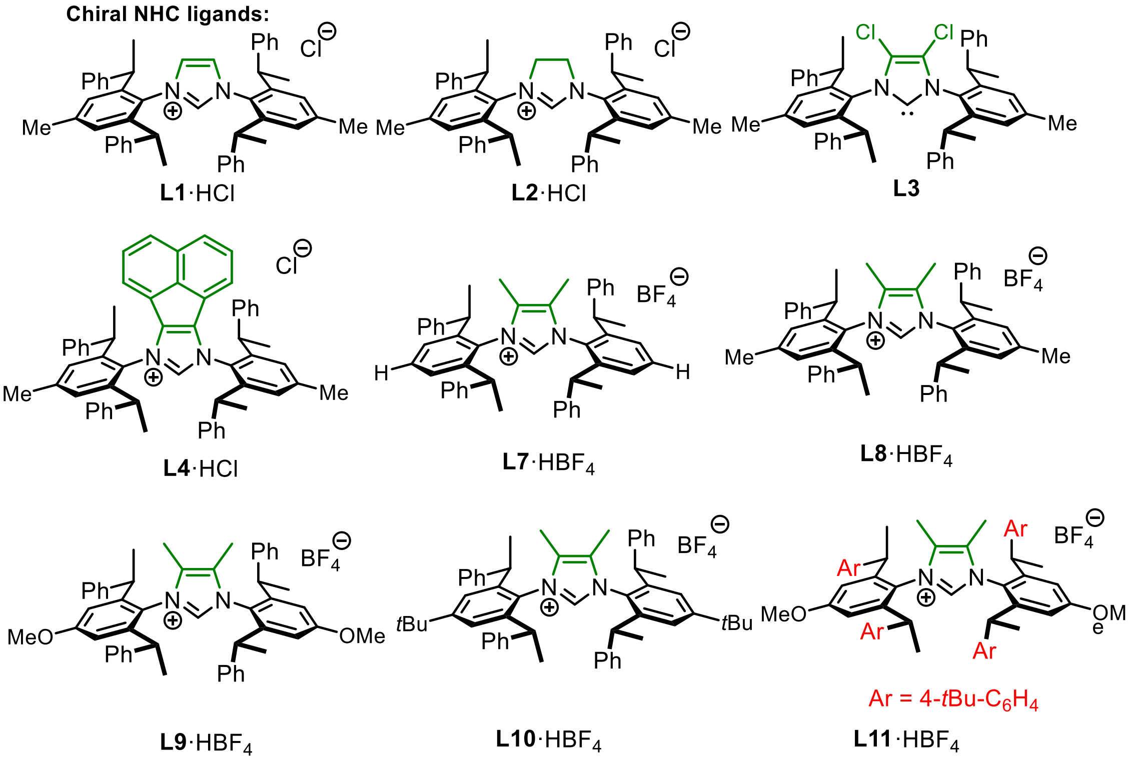

Entry	L	% Conv.[b]	% 3i[b]	dr of 3i[b]	er of 3i[b]
1[c]	**L1**·HCl	<5	-	-	-
2	**L2**·HCl	<5	-	-	-
3	**L3**	<5	-	-	-
4	**L4**·HCl	<5	-	-	-
5	**L7**·HBF_4	<5	-	-	-
6	**L8**·HBF_4	<5	-	-	-
7	**L9**·HBF_4	30	29	10:1	91:9
8	**L10**·HBF_4	<5	-	-	-
9	**L11**·HBF_4	85	85	16:1	92:8

[a] 0.1 mmol **1i**, 0.2 mmol **2b**, 3 μmol Ni catalyst, 3 μmol ligand and 3 μmol KO*t*Bu at 25 °C in solvent (1 M) for 24 h; [b] determined by ^{1}H NMR using dibromomethane as internal standard.[c] determined by HPLC.

Supplementary Fig 4.6 | Optimization of nickel NHC catalyzed hydroalkylation of cyclohexadiene with different nickel catalyst precursor.

4.5 Scope of enantioselective hydro-alkylation of nonconjugated dienes.

General procedure 6 (GP6)

In a glovebox, an oven dried screw-capped 2 mL vial was charged with a magnetic stir bar, Ni($^{t\mathrm{Bu}}$stb)$_3$ (3 μmol), carbene ligand precursor **L11**·HBF_4 (3 μmol), KO*t*Bu (3 μmol) freshly distilled and degassed (freeze pump thaw) CPME (Cyclopentyl methyl ether 0.1 mL) was then added and the catalyst mixture was stirred at room temperature for 30 min, then 1,4-cyclohexadiene (0.2 mmol) was then added during which a red black solution is formed. The mixture was stirred at room temperature for 10 minutes, ketone (0.1 mmol) was then added successively. The vial was sealed with a Teflon-lined screw cap, and running inside the glovebox at room temperature After 72 h, the vial was shipped outside of the glovebox. The reaction mixture was diluted with dichloromethane and filtered through a plug of silica gel. The crude solution was concentrated in vacuum and subjected to column chromatography to isolate the products.

Note: Liquid reagents/reactants were degassed by freeze-pump-thaw (3×) before being stored in the glovebox at -30 °C. Solid reagents/reactants were dried under high vacuum for 16h before being stored in the glovebox at -30 °C.

(*R*)-2-((*R*)-cyclohex-2-en-1-yl)-2-methyl-2,3-dihydro-1H-inden-1-one (**5i**):

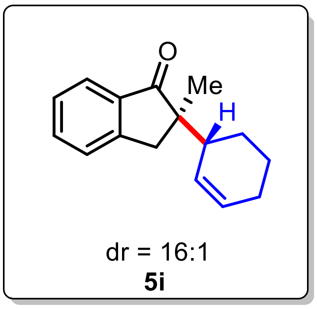

Following **GP6**, using ketone **1i** and 1,4-cyclohexadiene **2b**, **5i** was obtained as colourless oil in 87% yield (19.8 mg) dr = 16:1.**^{1}H NMR** (400 MHz, $CDCl_3$) δ 7.76 (d, *J* = 7.7 Hz, 1H), 7.57 (t, *J* = 7.8 Hz, 1H), 7.42 (d, *J* = 7.7 Hz, 1H), 7.36 (t, *J* = 7.4 Hz, 1H), 5.71 – 5.59 (m, 1H), 5.07 (d, *J* = 10.2 Hz, 1H), 3.15 (d, *J* = 17.2 Hz, 1H), 2.78 – 2.63 (m, 1.92H), 2.07 – 1.78 (m, 4H), 1.61 – 1.39 (m, 1H), 1.33 – 1.14 (m, 4H). **$[\alpha]_D^{20}$:** 105.8 ° (c = 1.0, $CHCl_3$).

Chiral HPLC: Chiralpak IC 4.6 x 250 mm; hexane:*i*-PrOH 99:1, 1.0 mL/min, 230 nm; t_R (major) = 10.7 min, t_R (minor) = 13.9 min, 92:8 er.

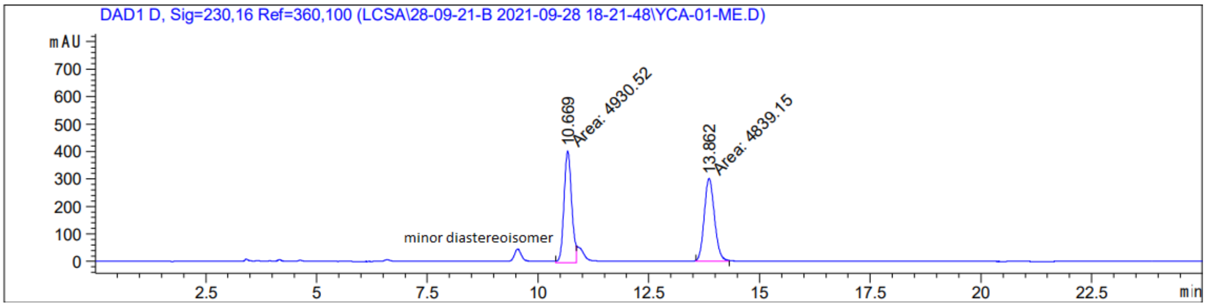

Signal 3: DAD1 D, Sig=230,16 Ref=360,100

Peak #	RetTime [min]	Type	Width [min]	Area [mAU*s]	Height [mAU]	Area %
1	10.669	MM	0.2014	4930.52393	408.12210	50.4677
2	13.862	MM	0.2679	4839.14795	301.10806	49.5323

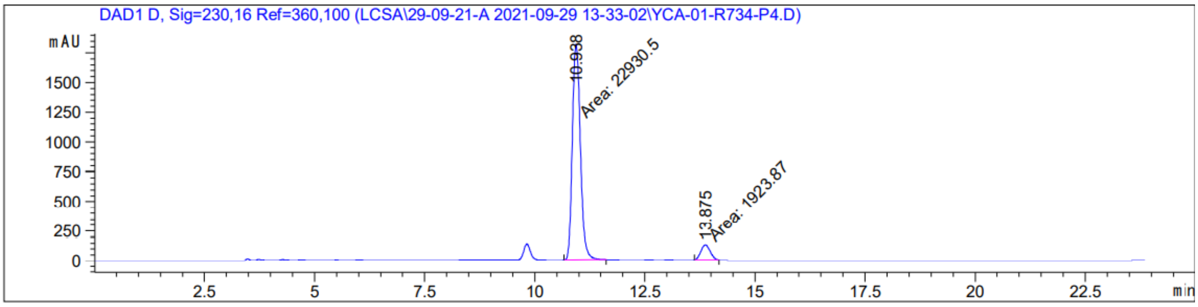

Signal 3: DAD1 D, Sig=230,16 Ref=360,100

Peak #	RetTime [min]	Type	Width [min]	Area [mAU*s]	Height [mAU]	Area %
1	10.938	MM	0.2105	2.29305e4	1815.20435	92.2594
2	13.875	MM	0.2493	1923.87170	128.62234	7.7406

(*R*)-2-((*R*)-cyclohex-2-en-1-yl)-2-ethyl-2,3-dihydro-1H-inden-1-one (**5j**):

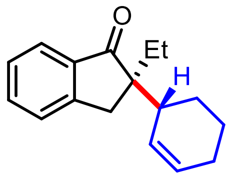

dr = >20:1
5j

Following **GP6**, using ketone **1j** and 1,4-cyclohexadiene **2b**, **5j** was obtained as colourless oil in 86% yield (20.6 mg) dr = >20:1. **^{1}H NMR** (400 MHz, $CDCl_3$,) δ 7.73 (d, *J* = 7.6 Hz, 1H), 7.56 (td, *J* = 7.5, 1.2 Hz, 1H), 7.42 (dt, *J* = 7.7, 0.8 Hz, 1H), 7.38 – 7.31 (m, 1H), 5.69 – 5.56 (m, 1H), 5.04 (dp, *J* = 10.1, 2.0 Hz, 1H), 3.09 (d, *J* = 17.3 Hz, 1H), 2.84 – 2.68 (m, 2H), 2.02 – 1.90 (m, 3H), 1.87 – 1.72 (m, 2H), 1.69 – 1.47 (m, 2H), 1.29 – 1.14 (m, 1H), 0.67 (t, *J* = 7.5 Hz, 3H). $[\alpha]_D^{20}$: 92.5 ° (c = 1.0, $CHCl_3$).

Chiral HPLC: Chiralpak IC 4.6 x 250 mm; hexane:*i*-PrOH 99:1, 1.0 mL/min, 230 nm; t_R (major) = 9.7 min, t_R (minor) = 12.4 min, 93:7 er.

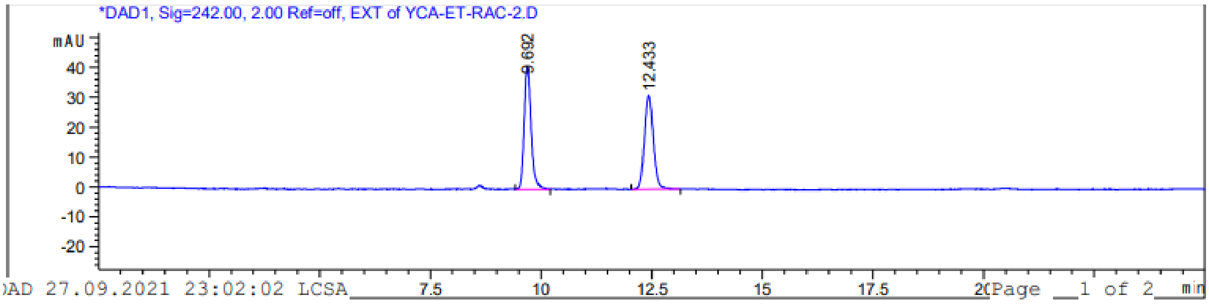

Peak #	RetTime [min]	Type	Width [min]	Area [mAU*s]	Height [mAU]	Area %
1	9.692	BB	0.1655	444.65799	40.67656	50.1471
2	12.433	BB	0.2162	442.04987	31.41203	49.8529

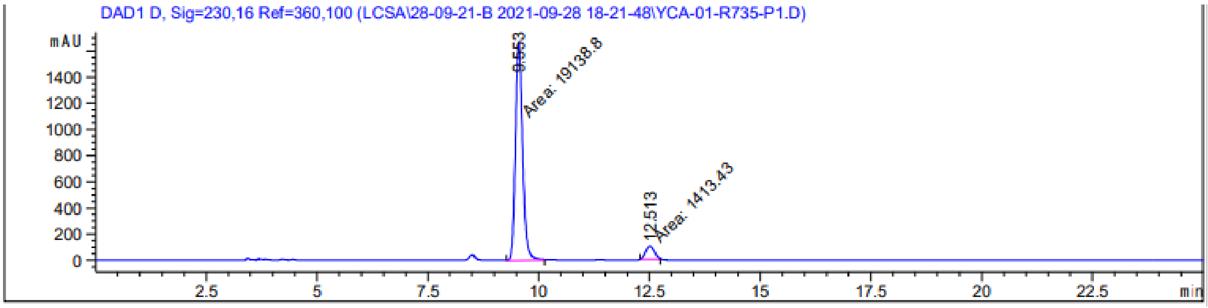

Peak #	RetTime [min]	Type	Width [min]	Area [mAU*s]	Height [mAU]	Area %
1	9.553	MM	0.1915	1.91388e4	1665.35571	93.1228
2	12.513	MM	0.2292	1413.42651	102.76643	6.8772

(*R*)-2-((*R*)-cyclohex-2-en-1-yl)-2-propyl-2,3-dihydro-1H-inden-1-one (**5k**):

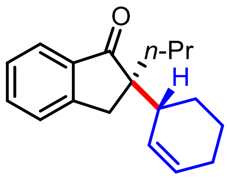

dr = >20:1
5k

Following **GP6**, using ketone **1k** and 1,4-cyclohexadiene **2b**, **5k** was obtained as colourless oil in 85% yield (21.6 mg) dr = >20:1. **^{1}H NMR** (400 MHz, $CDCl_3$) δ 7.73 (d, *J* = 7.6 Hz, 1H), 7.56 (td, *J* = 7.5, 1.2 Hz, 1H), 7.42 (dt, *J* = 7.7, 0.8 Hz, 1H), 7.38 – 7.31 (m, 1H), 5.69 – 5.57 (m, 1H), 5.04 (dt, *J* = 10.3, 1.8 Hz, 1H), 3.10 (d, *J* = 17.3 Hz, 1H), 2.85 – 2.67 (m, 2H), 2.05 – 1.91 (m, 3H), 1.87 – 1.77 (m, 1H), 1.69 (ddd, *J* = 13.2, 11.5, 5.7 Hz, 1H), 1.55 (dddd, *J* = 17.4, 10.1, 6.3, 2.5 Hz, 2H), 1.22 (tdd, *J* = 13.2, 11.0, 2.9 Hz, 1H), 1.12 – 0.93 (m, 2H), 0.79 (t, *J* = 7.2 Hz, 3H). $[\alpha]_D^{20}$**:** 58.0 ° (c = 1.0, $CHCl_3$).
Chiral HPLC: Chiralpak IC 4.6 x 250 mm; hexane:*i*-PrOH 99:1, 1.0 mL/min, 230 nm; t_R (major) = 8.0 min, t_R (minor) = 9.1 min, 95:5 er.

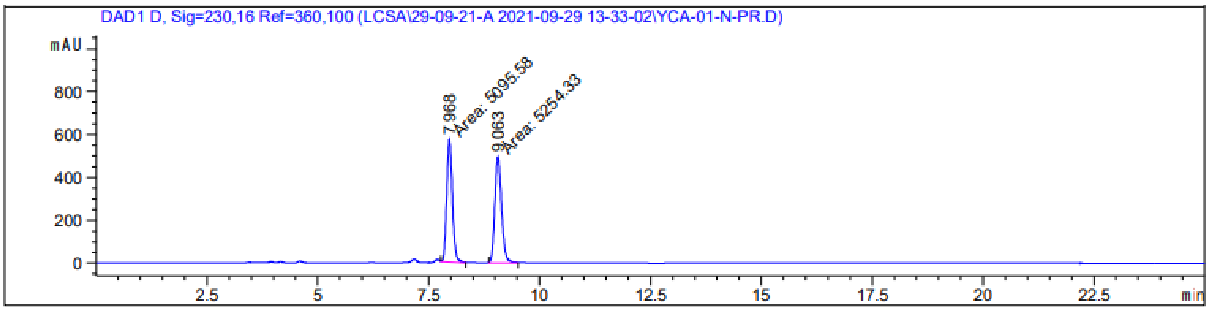

Peak #	RetTime [min]	Type	Width [min]	Area [mAU*s]	Height [mAU]	Area %
1	7.968	MM	0.1488	5095.57568	570.62500	49.2331
2	9.063	MM	0.1780	5254.32959	492.05777	50.7669

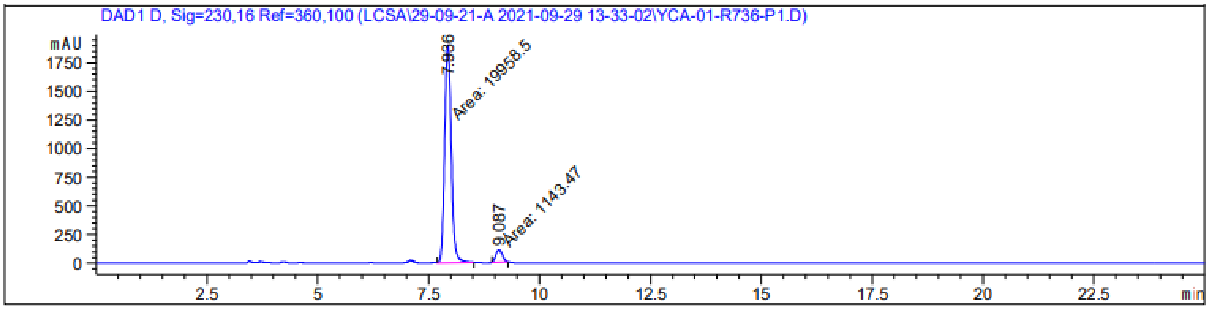

Peak #	RetTime [min]	Type	Width [min]	Area [mAU*s]	Height [mAU]	Area %
1	7.936	MM	0.1749	1.99585e4	1901.74158	94.5812
2	9.087	MM	0.1734	1143.46948	109.93412	5.4188

(*R*)-2-butyl-2-((*R*)-cyclohex-2-en-1-yl)-2,3-dihydro-1H-inden-1-one (**5l**):

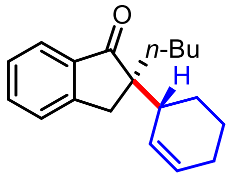

dr = >20:1

5l

Following **GP6**, using ketone **1l** and 1,4-cyclohexadiene **2b**, **5l** was obtained as colourless oil in 75% yield (20.1 mg) dr = >20:1.**^{1}H NMR** (400 MHz, $CDCl_3$) δ 7.73 (d, *J* = 7.6 Hz, 1H), 7.56 (td, *J* = 7.5, 1.2 Hz, 1H), 7.42 (d, *J* = 7.7 Hz, 1H), 7.38 – 7.31 (m, 1H), 5.67 – 5.57 (m, 1H), 5.04 (dt, *J* = 10.3, 1.8 Hz, 1H), 3.10 (d, *J* = 17.3 Hz, 1H), 2.83 – 2.69 (m, 2H), 2.04 – 1.89 (m, 3H), 1.82 (dp, *J* = 11.6, 4.3, 3.7 Hz, 1H), 1.71 (ddd, *J* = 13.2, 11.2, 6.2 Hz, 1H), 1.62 – 1.47 (m, 2H), 1.30 – 1.12 (m, 3H), 0.99 (tdd, *J* = 13.7, 7.2, 3.9 Hz, 2H), 0.78 (t, *J* = 7.3 Hz, 3H). $[\alpha]_D^{20}$**:** 92.3 ° (c = 0.5, $CHCl_3$).

Chiral HPLC: Chiralpak IC 4.6 x 250 mm; hexane:*i*-PrOH 99:1, 1.0 mL/min, 230 nm; t_R (major) = 10.8 min, t_R (minor) = 12.2 min, 94:6 er.

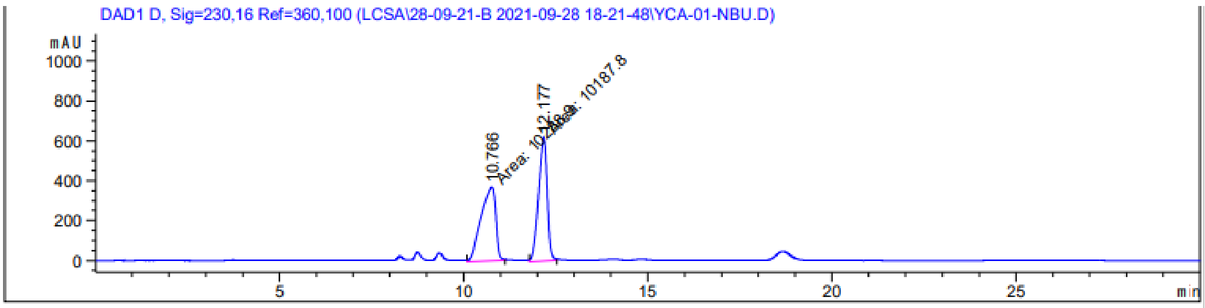

Peak #	RetTime [min]	Type	Width [min]	Area [mAU*s]	Height [mAU]	Area %
1	10.766	MM	0.4662	1.02889e4	367.84625	50.2469
2	12.177	MM	0.2745	1.01878e4	618.48175	49.7531

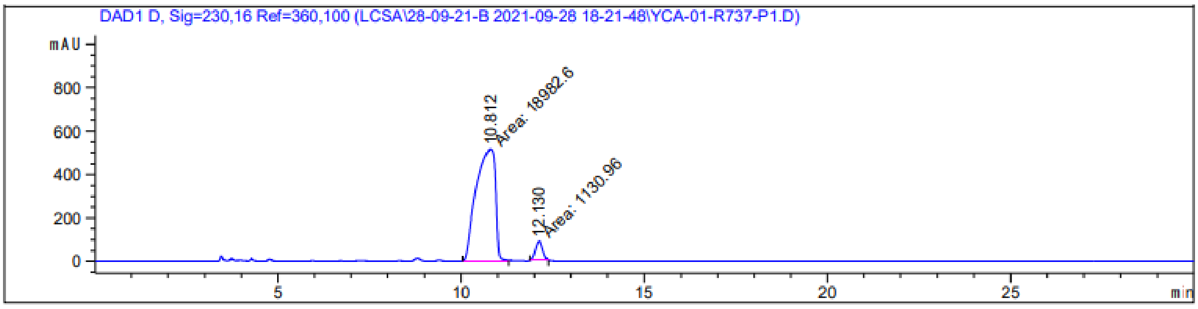

Peak #	RetTime [min]	Type	Width [min]	Area [mAU*s]	Height [mAU]	Area %
1	10.812	MM	0.6128	1.89826e4	516.24554	94.3771
2	12.130	MM	0.2188	1130.96399	86.16316	5.6229

(*R*)-2-((*R*)-cyclohex-2-en-1-yl)-2,5-dimethyl-2,3-dihydro-1H-inden-1-one (**5m**):

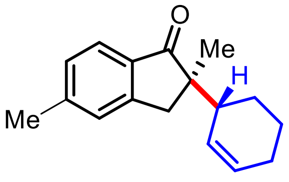

dr = 15:1
5m

Following **GP6**, using ketone **1m** and 1,4-cyclohexadiene **2b**, **5m** was obtained as colourless oil in 72% yield (17.2 mg) dr =15:1. **^{1}H NMR** (400 MHz, $CDCl_3$) δ 7.65 (d, *J* = 7.8 Hz, 1H), 7.22 (s, 1H), 7.17 (d, *J* = 7.3 Hz, 1H), 5.69 – 5.60 (m, 1H), 5.07 (dp, *J* = 10.2, 1.9 Hz, 1H), 3.09 (d, *J* = 17.2 Hz, 1H), 2.77 – 2.57 (m, 1H), 2.43 (s, 3H), 2.07 – 1.90 (m, 3H), 1.89 – 1.77 (m, 1H), 1.60 – 1.47 (m, 1H), 1.24 (ddd, *J* = 13.0, 2.9, 2.0 Hz, 1H), 1.17 (s, 3H). $[\alpha]_D^{20}$: 67.3 ° (c = 0.5, $CHCl_3$).

Chiral HPLC: Chiralpak IG 4.6 x 250 mm; hexane:*i*-PrOH 99:1, 1.0 mL/min, 230 nm; t_R (major) = 13.3 min, t_R (minor) = 25.3 min, 93:7 er.

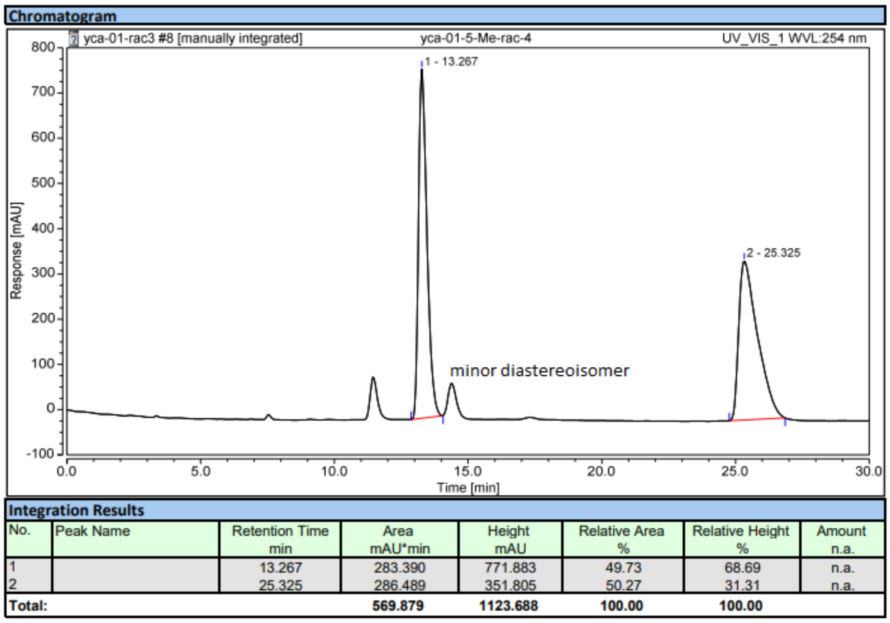

Integration Results

No.	Peak Name	Retention Time min	Area mAU*min	Height mAU	Relative Area %	Relative Height %	Amount n.a.
1		13.267	283.390	771.883	49.73	68.69	n.a.
2		25.325	286.489	351.805	50.27	31.31	n.a.
**Total:**			**569.879**	**1123.688**	**100.00**	**100.00**	

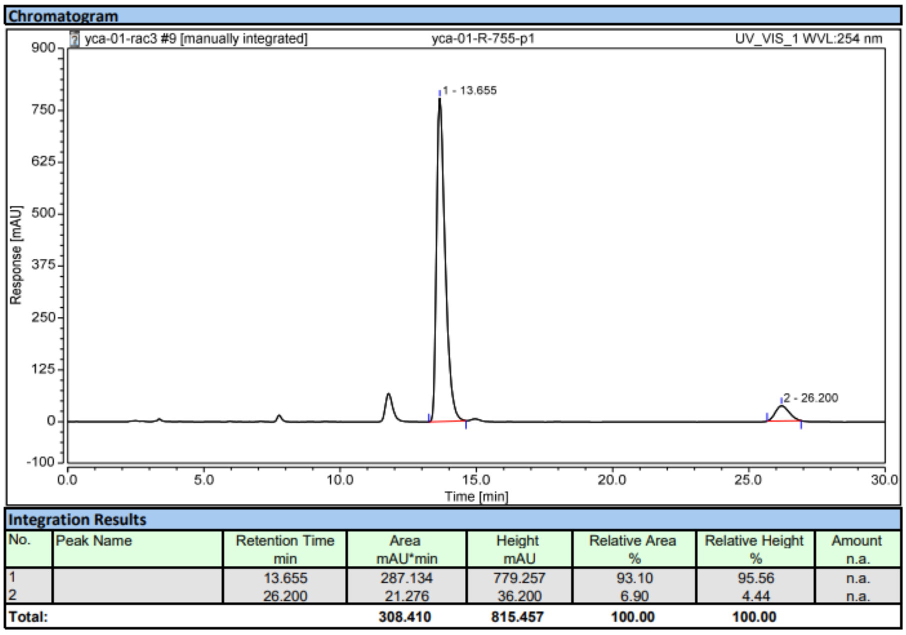

Integration Results

No.	Peak Name	Retention Time min	Area mAU*min	Height mAU	Relative Area %	Relative Height %	Amount n.a.
1		13.655	287.134	779.257	93.10	95.56	n.a.
2		26.200	21.276	36.200	6.90	4.44	n.a.
**Total:**			**308.410**	**815.457**	**100.00**	**100.00**	

(*R*)-2-((*R*)-cyclohex-2-en-1-yl)-6-fluoro-2-methyl-2,3-dihydro-1H-inden-1-one (**5o**):

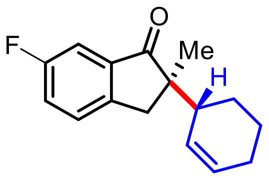

dr = 15:1
5o

Following **GP6**, using ketone **1o** and 1,4-cyclohexadiene **2b**, **5o** was obtained as colourless oil in 64% yield (15.7 mg) dr =15:1. **^{1}H NMR** (400 MHz, $CDCl_3$) δ 7.45 – 7.34 (m, 2H), 7.32 – 7.27 (m, 1H), 5.75 – 5.59 (m, 1.H), 5.05 (dp, *J* = 10.3, 1.9 Hz, 1H), 3.16 – 3.01 (m, 1H), 2.77 – 2.48 (m, 2H), 2.07 – 1.77 (m, 4H), 1.61 – 1.47 (m, 1.H), 1.28 – 1.20 (m, 1H), 1.18 (s, 3H).). $[\alpha]_D^{20}$: 98.3 ° (c = 0.5, $CHCl_3$). **Chiral HPLC:** Chiralpak IG 4.6 x 250 mm; hexane:*i*-PrOH 99:1, 1.0 mL/min, 230 nm; t_R (major) = 7.3 min, t_R (minor) = 12.3 min, 93:7 er.

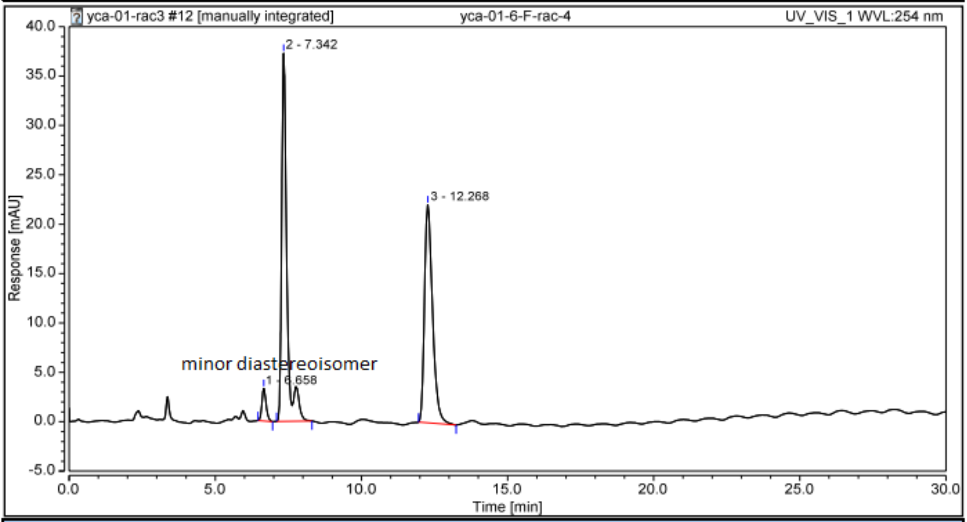

Integration Results

No.	Peak Name	Retention Time min	Area mAU*min	Height mAU	Relative Area %	Relative Height %	Amount n.a.
1		6.658	0.551	3.288	3.60	5.25	n.a.
2		7.342	7.698	37.273	50.25	59.51	n.a.
3		12.268	7.070	22.073	46.15	35.24	n.a.
**Total:**			**15.319**	**62.633**	**100.00**	**100.00**	

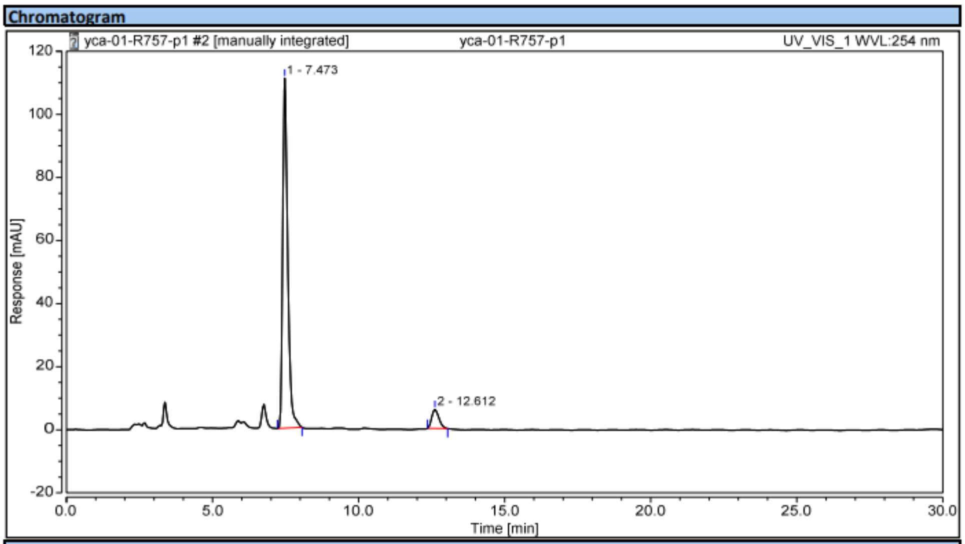

Integration Results

No.	Peak Name	Retention Time min	Area mAU*min	Height mAU	Relative Area %	Relative Height %	Amount n.a.
1		7.473	22.103	110.974	92.80	94.87	n.a.
2		12.612	1.714	6.007	7.20	5.13	n.a.
**Total:**			**23.817**	**116.981**	**100.00**	**100.00**	

(*R*)-2-((*R*)-cyclohex-2-en-1-yl)-2-methyl-2,3-dihydro-1H-cyclopenta[a]naphthalen-1-one (**5r**):

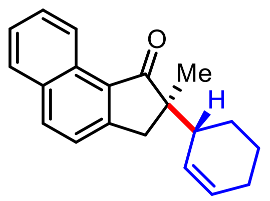

dr = 9.5:1
5r

Following a slight modification of **GP6**, using ketone **1r**, 1,4-cyclohexadiene **2b**, 10 mol% Ni catalyst 10 mol% ligand precursor 10 mol% KO*t*Bu. **5r** was obtained as a colorless thick oil in 31% yield (8.6 mg) dr = 9.5:1. **^{1}H NMR** (400 MHz, $CDCl_3$) δ 9.19 (d, J = 8.3 Hz, 1H), 8.04 (d, J = 8.4 Hz, 1H), 7.89 (d, J = 8.0 Hz, 1H), 7.71 – 7.64 (m, 1H), 7.59 – 7.53 (m, 1H), 7.49 (d, J = 8.4 Hz, 1H), 5.69 – 5.57 (m, 1H), 5.14 (dt, J = 10.2, 2.0 Hz, 1H), 3.23 (d, J = 17.6 Hz, 1H), 2.88 – 2.69 (m, 2H), 2.10 – 1.79 (m, 4H), 1.67 – 1.55 (m, 1H), 1.39 – 1.31 (m, 1H), 1.26 (s, 3H). $[\alpha]_D^{20}$**:** 40.0 ° (c = 0.5, $CHCl_3$).

Chiral HPLC: Chiralpak IC 4.6 x 250 mm; hexane:*i*-PrOH 99:1, 0.7 mL/min, 230 nm; t_R (major) = 10.0 min, t_R (minor) = 13.2 min, 64:36 er.

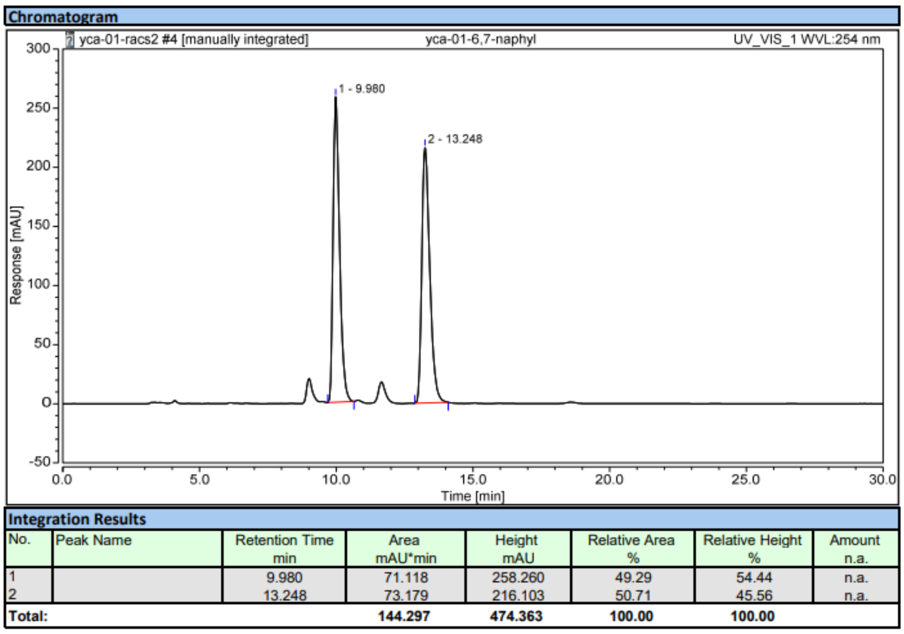

Integration Results

No.	Peak Name	Retention Time min	Area mAU*min	Height mAU	Relative Area %	Relative Height %	Amount n.a.
1		9.980	71.118	258.260	49.29	54.44	n.a.
2		13.248	73.179	216.103	50.71	45.56	n.a.
**Total:**			**144.297**	**474.363**	**100.00**	**100.00**	

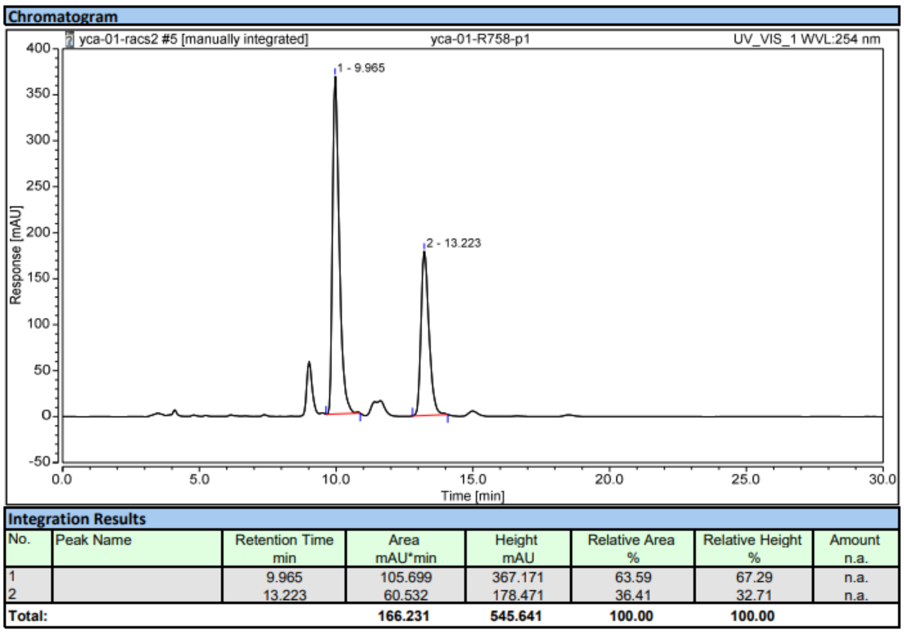

Integration Results

No.	Peak Name	Retention Time min	Area mAU*min	Height mAU	Relative Area %	Relative Height %	Amount n.a.
1		9.965	105.699	367.171	63.59	67.29	n.a.
2		13.223	60.532	178.471	36.41	32.71	n.a.
**Total:**			**166.231**	**545.641**	**100.00**	**100.00**	

In a glovebox, an oven dried screw-capped 2 mL vial was charged with a magnetic stir bar, Ni(^{tBu}stb)$_3$ (10 μmol), free carbene ligand **L8** (10 μmol), freshly distilled and degassed (freeze pump thaw) CPME (Cyclopentyl methyl ether 0.1 mL) was then added and the catalyst mixture was stirred at room temperature for 10 min, then 1,4-cyclohexadiene (0.2 mmol) was then added during which a dark solution is formed. The mixture was stirred at room temperature for 1 minute, ketone (0.1 mmol) was then added successively. The vial was sealed with a Teflon-lined screw cap, and running inside the glovebox at room temperature After 72 h, the vial was shipped outside of the glovebox. The reaction mixture was diluted with dichloromethane and filtered through a plug of silica gel. The crude solution was concentrated in vacuum and subjected to column chromatography 100:1 PE/EA to isolate the products.

(*R*)-2-((*R*)-cyclohex-2-en-1-yl)-1,2-diphenylethan-1-one (**6a**):

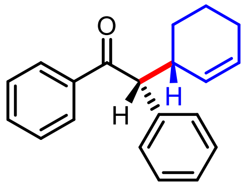

dr = >20:1

6a

6a was obtained as white solid in 38% yield (10.5 mg) dr = > 20:1.**^{1}H NMR** (400 MHz, CDCl$_3$) δ 7.96 – 7.85 (m, 2H), 7.45 – 7.37 (m, 1H), 7.36 – 7.17 (m, 6H), 7.16 – 7.08 (m, 1H), 5.63 (m, 1H), 5.57 – 5.50 (m, 1H), 4.33 (d, *J* = 10.9 Hz, 1H), 3.06 (m, 1H), 1.92 (m, 2H), 1.63 (m, 1H), 1.43 (m, 1H), 1.30 (m, 1H), 1.09 (m, 1H).$[\alpha]_D^{20}$**:** -4.3 ° (c = 1.0, CHCl$_3$).

Chiral HPLC: Chiralpak IC 4.6 x 250 mm; hexane:*i*-PrOH 99:1, 0.7 mL/min, 230 nm; t_R (major) = 5.7 min, t_R (minor) = 8.1 min, 72:28 er.

Chromatogram

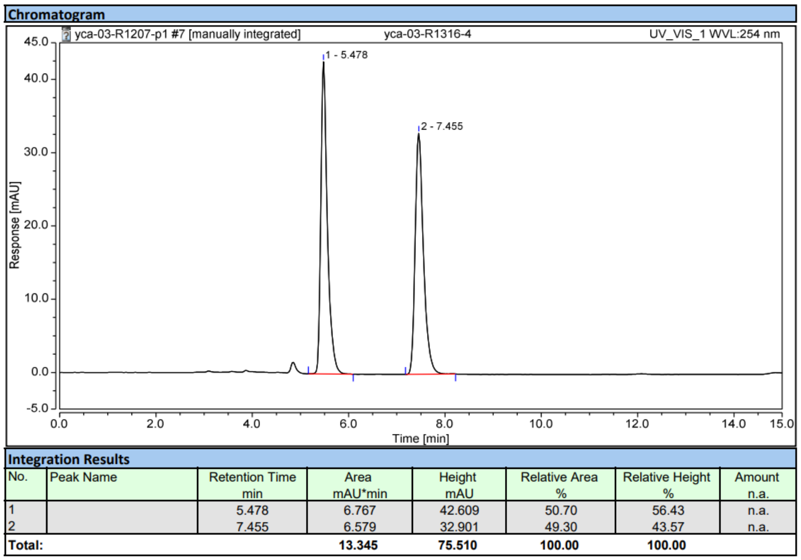

Integration Results

No.	Peak Name	Retention Time min	Area mAU*min	Height mAU	Relative Area %	Relative Height %	Amount n.a.
1		5.478	6.767	42.609	50.70	56.43	n.a.
2		7.455	6.579	32.901	49.30	43.57	n.a.
**Total:**			**13.345**	**75.510**	**100.00**	**100.00**	

Chromatogram and Results

Injection Details

Injection Name:	**yca-01-R671-p1**	*Run Time (min):*	**30.00**
Vial Number:	**1:92**	*Injection Volume:*	**10.00**
Injection Type:	**Unknown**	*Channel:*	**UV_VIS_1**
Calibration Level:		*Wavelength:*	**254**
Instrument Method:	**IC 99_1 HEX_IPA 30 min 0-7ml_min**	*Bandwidth:*	**n.a.**
Processing Method:	**Basic_quant**	*Dilution Factor:*	**1.0000**
Injection Date/Time:	**29.août.21 16:52**	*Sample Weight:*	**1.0000**

Chromatogram

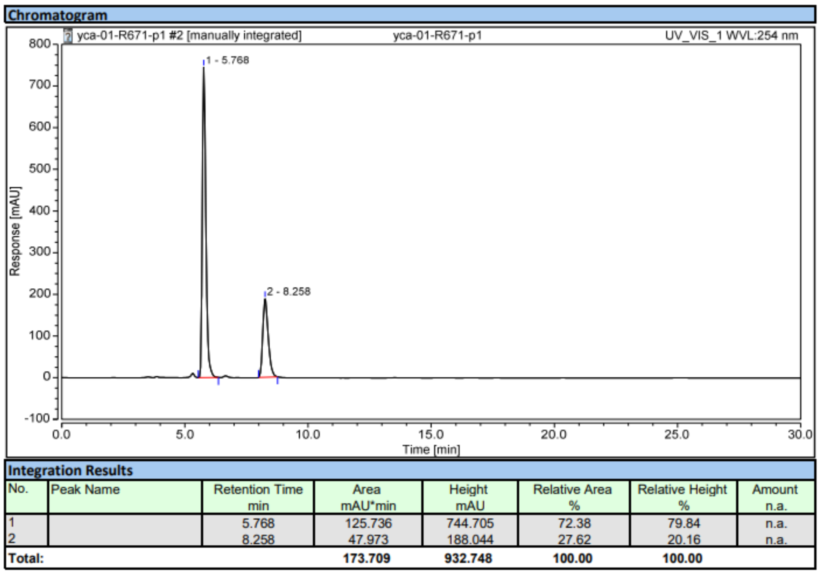

Integration Results

No.	Peak Name	Retention Time min	Area mAU*min	Height mAU	Relative Area %	Relative Height %	Amount n.a.
1		5.768	125.736	744.705	72.38	79.84	n.a.
2		8.258	47.973	188.044	27.62	20.16	n.a.
**Total:**			**173.709**	**932.748**	**100.00**	**100.00**	

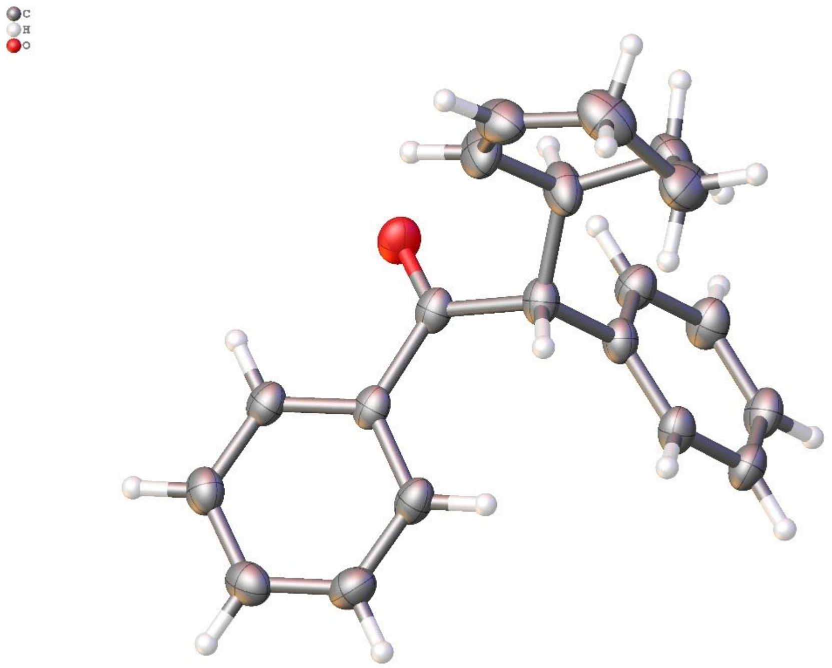

Supplementary Fig 4.7 | ORTEP diagram of **6a** (thermal ellipsoids are shown at the 50% probability level, for the reason of clarity all the hydrogen atoms are omitted). CCDC 2122925.

In a glovebox, an oven dried screw-capped 2 mL vial was charged with a magnetic stir bar, Ni(^{tBu}stb)$_3$ (10 μmol), carbene ligand precursor **L8**·HBF_4 (10 μmol), KO*t*Bu (10 μmol) freshly distilled and degassed (freeze pump thaw) CPME (Cyclopentyl methyl ether 0.1 mL) was then added and the catalyst mixture was stirred at room temperature for 30 min, then 1,5-cycloctadiene (0.2 mmol) was then added during which a dark solution is formed. The mixture was stirred at room temperature for 10 minutes, ketone (0.1 mmol) was then added successively. The vial was sealed with a Teflon-lined screw cap, and running inside the glovebox at room temperature After 72 h, the vial was shipped outside of the glovebox. The reaction mixture was diluted with dichloromethane and filtered through a plug of silica gel. The crude solution was concentrated in vacuum and subjected to column chromatography to isolate the products.

<u>(*R*)-2-((*R*,*Z*)-cyclooct-2-en-1-yl)-2-methyl-2,3-dihydro-1H-inden-1-one (**7i**):</u>

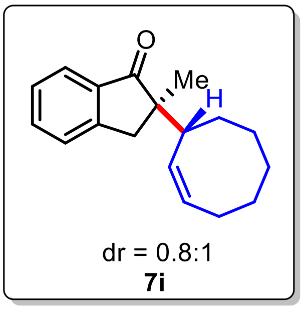

7i was obtained as colourless oil in 31% yield (7.9 mg) dr = 1.2:1. $[\alpha]_D^{20}$**:** -3.0 ° (c = 1.0, $CHCl_3$).

Chiral HPLC: Chiralpak IC 4.6 x 250 mm; hexane:*i*-PrOH 99:1, 1.0 mL/min, 230 nm; t_R (minor) = 8.6 min, t_R (minor) = 10.4 min, t_R (major) = 12.1 min, t_R (major) = 13.2 min er = 87:13 er' =97:3

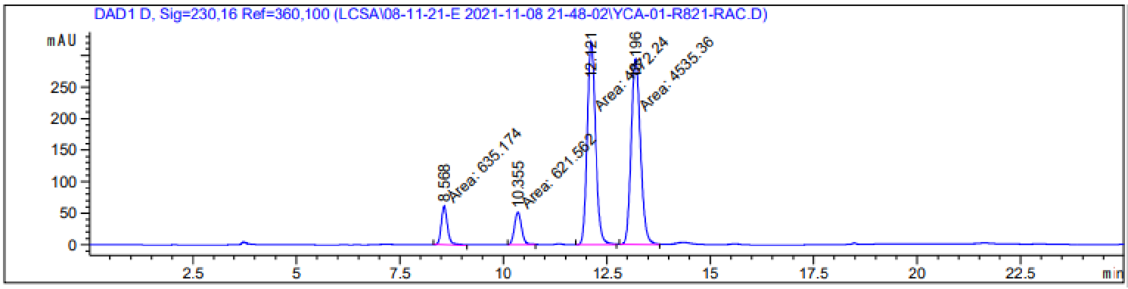

Peak #	RetTime [min]	Type	Width [min]	Area [mAU*s]	Height [mAU]	Area %
1	8.568	MM	0.1741	635.17365	60.81872	6.1285
2	10.355	MM	0.1996	621.56238	51.88994	5.9971
3	12.121	MM	0.2369	4572.23535	321.63141	44.1151
4	13.196	MM	0.2573	4535.36426	293.76538	43.7593

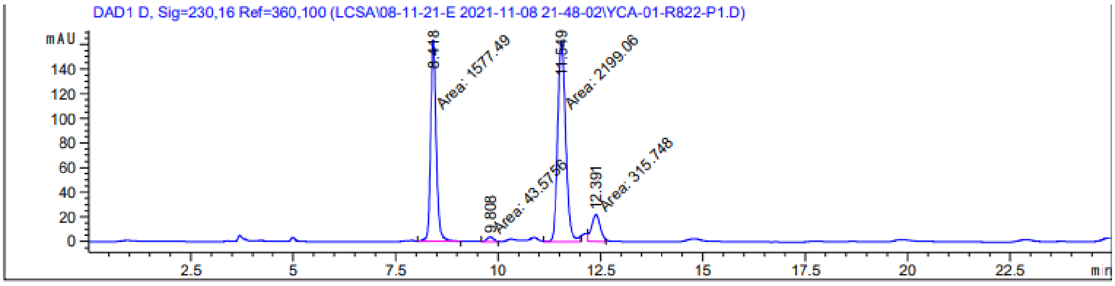

Peak #	RetTime [min]	Type	Width [min]	Area [mAU*s]	Height [mAU]	Area %
1	8.418	MM	0.1607	1577.49121	163.61981	38.1416
2	9.808	MM	0.1897	43.57558	3.82777	1.0536
3	11.549	MM	0.2255	2199.06372	162.51646	53.1704
4	12.391	MM	0.2418	315.74829	21.75967	7.6344

General procedure 7 (GP7)

In a glovebox, an oven dried screw-capped 2 mL vial was charged with a magnetic stir bar, Ni(^{tBu}stb)$_3$ (10 μmol), carbene ligand precursor **L11**·HBF_4 (10 μmol), KO*t*Bu (10 μmol) freshly distilled and degassed (freeze pump thaw) CPME (Cyclopentyl methyl ether 0.1 mL) was then added and the catalyst mixture was stirred at room temperature for 30 min, then 1,4-cyclohexadiene (0.2 mmol) was then added during which a red black solution is formed. The mixture was stirred at room temperature for 10 minutes, ketone (0.1 mmol) was then added successively. The vial was sealed with a Teflon-lined screw cap, and running inside the glovebox at 40 ^{o}C. After 72 h, the vial was shipped outside of the glovebox. The reaction mixture was diluted with dichloromethane and filtered through a plug of silica gel. The crude solution was concentrated in vacuum and subjected to column chromatography to isolate the products.

Note: Liquid reagents/reactants were degassed by freeze-pump-thaw (3×) before being stored in the glovebox at -30 °C. Solid reagents/reactants were dried under high vacuum for 16h before being stored in the glovebox at -30 °C.

(*R*)-2-((*R*)-cyclohex-2-en-1-yl)-2-phenyl-3,4-dihydronaphthalen-1(2H)-one (**8s**):

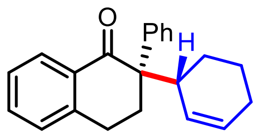

dr = >20:1
8s

Following **GP7**, using ketone **1s** and 1,4-cyclohexadiene **2b**, **8s** was obtained as a white solid in 80% yield (24.1 mg) dr = >20:1. **^{1}H NMR** (400 MHz, $CDCl_3$) δ 8.07 (dd, J = 7.9, 1.6 Hz, 1H), 7.34 – 7.08 (m, 7H), 7.01 (d, J = 7.7 Hz, 1H), 5.68 – 5.60 (m, 1H), 5.40 (dt, J = 10.3, 1.9 Hz, 1H), 3.16 (ddt, J = 13.8, 5.3, 2.7 Hz, 1H), 3.04 (ddd, J = 17.6, 13.2, 4.5 Hz, 1H), 2.80 (ddd, J = 17.2, 4.7, 2.5 Hz, 1H), 2.55 (ddd, J = 14.6, 4.5, 2.6 Hz, 1H), 2.29 (ddd, J = 14.6, 13.2, 4.6 Hz, 1H), 1.94 – 1.81 (m, 2H), 1.69 – 1.57 (m, 1H), 1.44 – 1.30 (m, 1H), 1.21 – 1.10 (m, 1H), 1.05 – 0.93 (m, 1H). $[\alpha]_D^{20}$: -158 ° (c = 1.0, $CHCl_3$).

Chiral HPLC: Chiralpak IC 4.6 x 250 mm; hexane:*i*-PrOH 99:1, 0.7 mL/min, 230 nm; t_R (major) = 7.3 min, t_R (minor) = 11.8 min, 93:7 er.

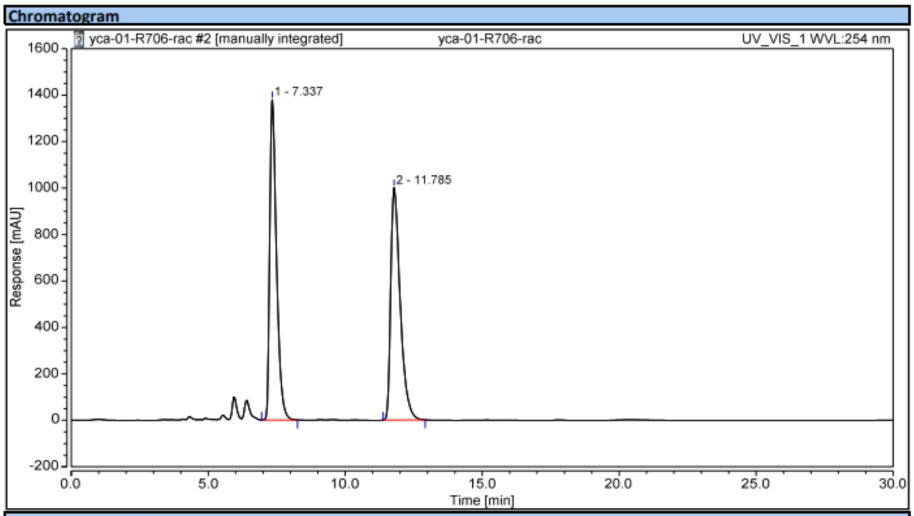

Integration Results

No.	Peak Name	Retention Time min	Area mAU*min	Height mAU	Relative Area %	Relative Height %	Amount n.a.
1		7.337	396.609	1378.911	49.73	57.96	n.a.
2		11.785	400.972	1000.075	50.27	42.04	n.a.
**Total:**			**797.581**	**2378.985**	**100.00**	**100.00**	

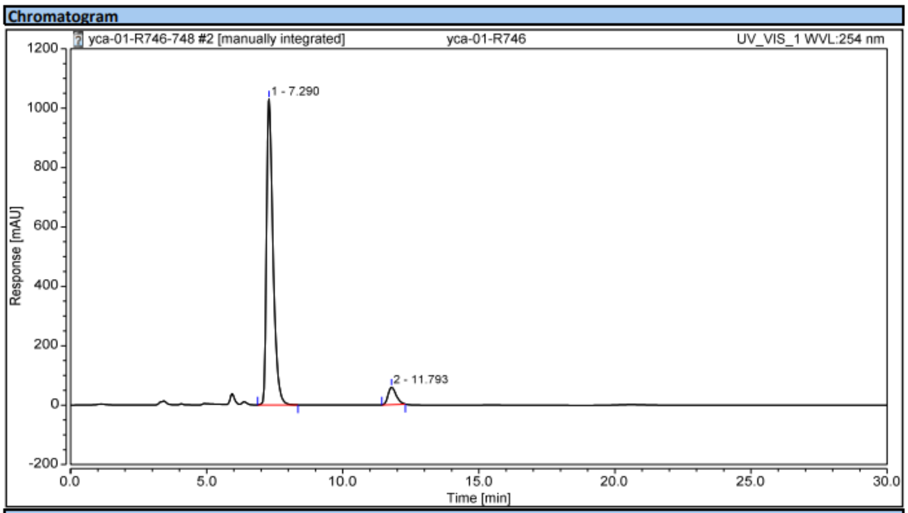

Integration Results

No.	Peak Name	Retention Time min	Area mAU*min	Height mAU	Relative Area %	Relative Height %	Amount n.a.
1		7.290	286.620	1030.255	93.40	94.61	n.a.
2		11.793	20.260	58.736	6.60	5.39	n.a.
**Total:**			**306.880**	**1088.992**	**100.00**	**100.00**	

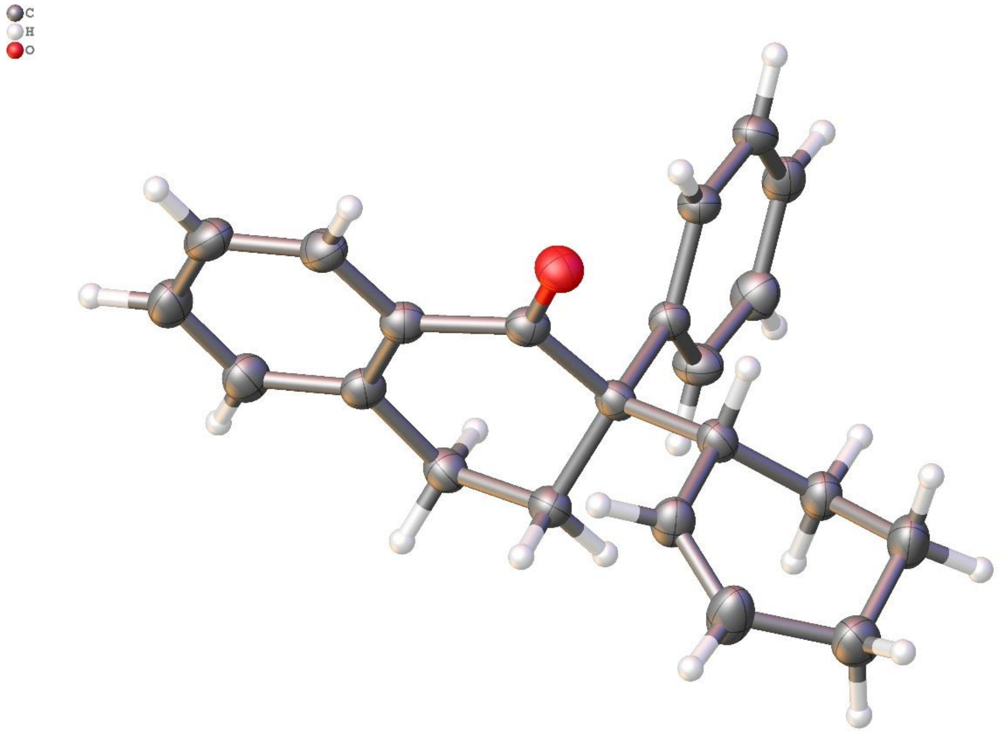

Supplementary Fig 4.8 | ORTEP diagram of **8s** (thermal ellipsoids are shown at the 50% probability level, for the reason of clarity all the hydrogen atoms are omitted). CCDC 2122926.

<u>(*R*)-2-((*R*)-cyclohex-2-en-1-yl)-2-(p-tolyl)-3,4-dihydronaphthalen-1(2H)-one (**8t**):</u>

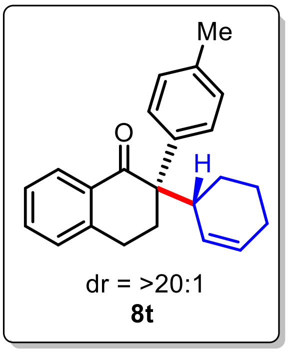

Following **GP7**, using ketone **1t** and 1,4-cyclohexadiene **2b**, **8t** was obtained as a colourless thick oil in 74% yield (23.5 mg) dr = >20:1.^{1}**H NMR** (400 MHz, $CDCl_3$) δ 8.14 (dd, J = 7.9, 1.6 Hz, 1H), 7.36 (td, J = 7.4, 1.5 Hz, 1H), 7.27 – 7.18 (m, 3H), 7.09 (t, J = 7.7 Hz, 3H), 5.71 (dt, J = 10.2, 3.8 Hz, 1H), 5.47 (dt, J = 10.3, 1.9 Hz, 1H), 3.20 (dq, J = 8.4, 2.6 Hz, 1H), 3.11 (ddd, J = 17.4, 13.2, 4.5 Hz, 1H), 2.86 (ddd, J = 17.3, 4.9, 2.6 Hz, 1H), 2.64 – 2.55 (m, 1H), 2.34 (ddd, J = 14.6, 13.1, 4.7 Hz, 1H), 2.28 (s, 3H), 2.00 – 1.89 (m, 2H), 1.70 (dt, J = 12.9, 3.5 Hz, 1H), 1.51 – 1.40 (m, 1H), 1.28 – 1.18 (m, 1H), 1.13 – 1.03 (m, 1H). $[\boldsymbol{\alpha}]_{\boldsymbol{D}}^{\boldsymbol{20}}$**:** -132 ° (c = 1.0, $CHCl_3$).

Chiral HPLC: SFC, Chiralpak IJ; MeOH:CO_2 = 1%, 2 mL/min, 230 nm; t_R (major) = 4.4 min, t_R (minor) = 4.0 min, 93:7 er.

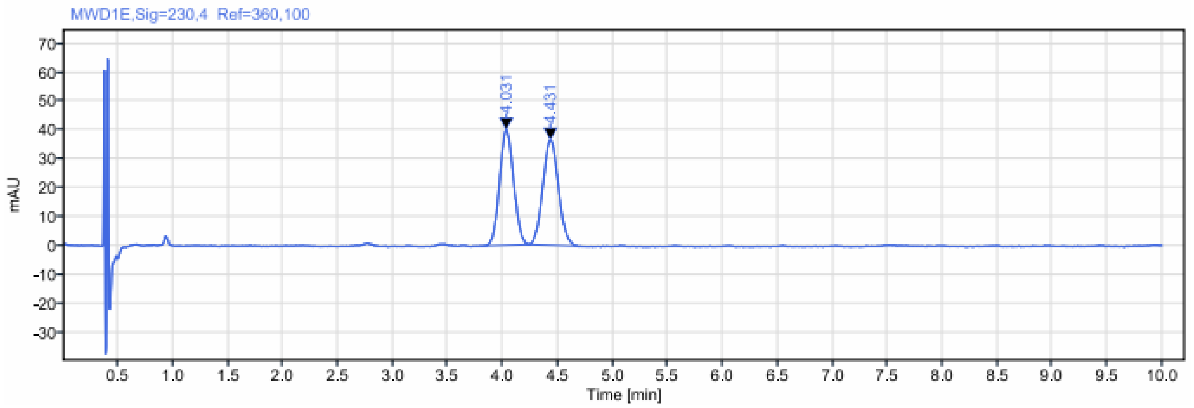

Signal: MWD1E,Sig=230,4 Ref=360,100

RT [min]	Type	Width [min]	Area	Height	Area%	Name
4.031	MM m	0.44	353.38	39.66	49.63	
4.431	MM m	0.49	358.69	36.25	50.37	

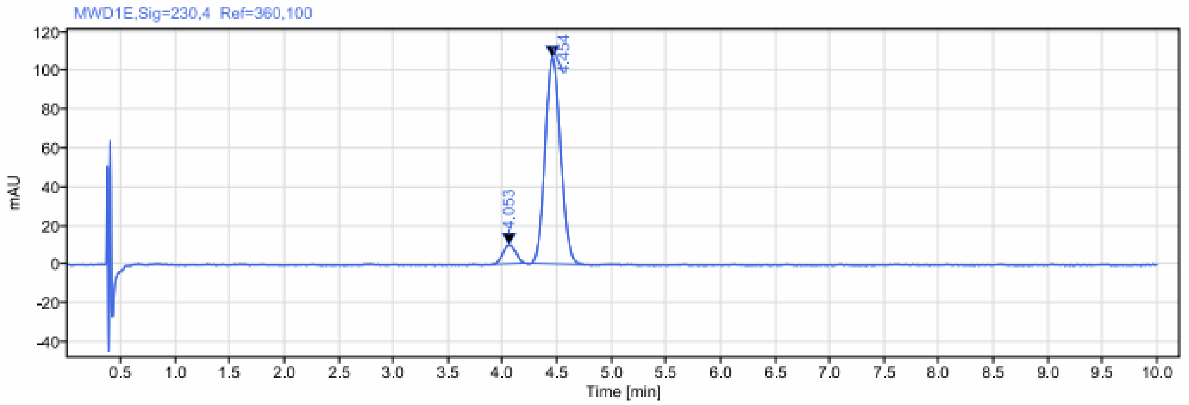

Signal: MWD1E,Sig=230,4 Ref=360,100

RT [min]	Type	Width [min]	Area	Height	Area%	Name
4.053	MM m	0.32	79.30	9.58	7.02	
4.454	MM m	0.61	1050.52	106.21	92.98	

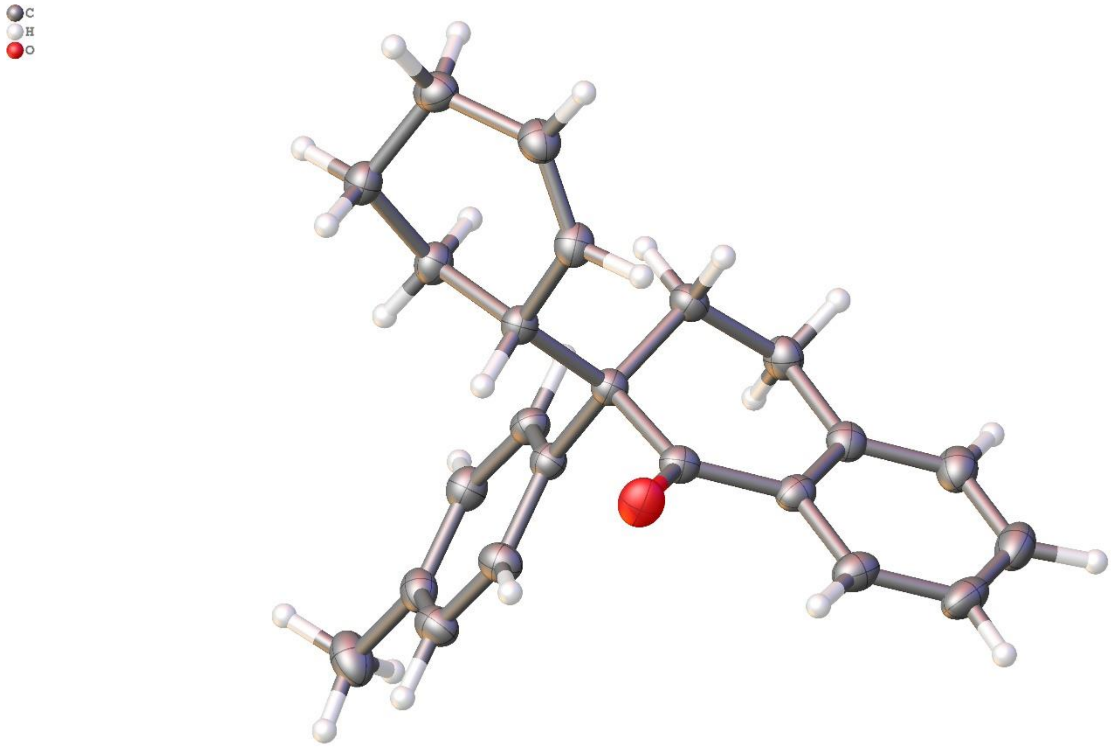

Supplementary Fig 4.9 | ORTEP diagram of **8t** (thermal ellipsoids are shown at the 50% probability level, for the reason of clarity all the hydrogen atoms are omitted). CCDC 2122924.

(*R*)-2-((*R*)-cyclohex-2-en-1-yl)-2-(m-tolyl)-3,4-dihydronaphthalen-1(2H)-one (**8u**):

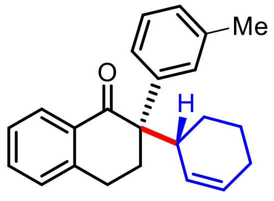

dr = >20:1
8u

Following **GP7**, using ketone **1u** and 1,4-cyclohexadiene **2b**, **8u** was obtained as a white solid in 60% yield (18.9 mg) dr = >20:1. **^{1}H NMR** (400 MHz, $CDCl_3$) δ 8.15 (d, J = 9.3 Hz, 1H), 7.37 (td, J = 7.5, 1.5 Hz, 1H), 7.29 – 7.05 (m, 5H), 7.01 (d, J = 7.0 Hz, 1H), 5.76 – 5.65 (m, 1H), 5.51 – 5.41 (m, 1H), 3.25 – 3.06 (m, 2H), 2.92 – 2.81 (m, 1H), 2.66 – 2.56 (m, 1H), 2.40 – 2.27 (m, 4H), 1.99 – 1.91 (m, 2H), 1.76 – 1.66 (m, 1H), 1.51 – 1.39 (m, 1H), 1.27 – 1.17 (m, 1H), 1.11 – 1.03 (m, 1H).$[\alpha]_D^{20}$: -130.8 ° (c = 1.0, $CHCl_3$).

Chiral HPLC: Chiralpak IC 4.6 x 250 mm; hexane:*i*-PrOH 99:1, 0.7 mL/min, 230 nm; t_R (major) = 7.2 min, t_R (minor) = 12.7 min, 91:9 er.

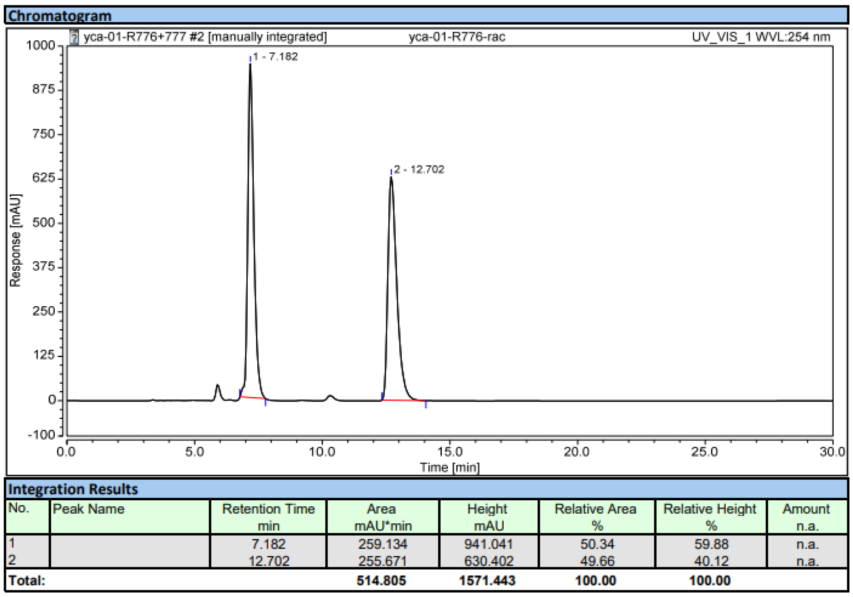

Integration Results

No.	Peak Name	Retention Time min	Area mAU*min	Height mAU	Relative Area %	Relative Height %	Amount n.a.
1		7.182	259.134	941.041	50.34	59.88	n.a.
2		12.702	255.671	630.402	49.66	40.12	n.a.
**Total:**			**514.805**	**1571.443**	**100.00**	**100.00**	

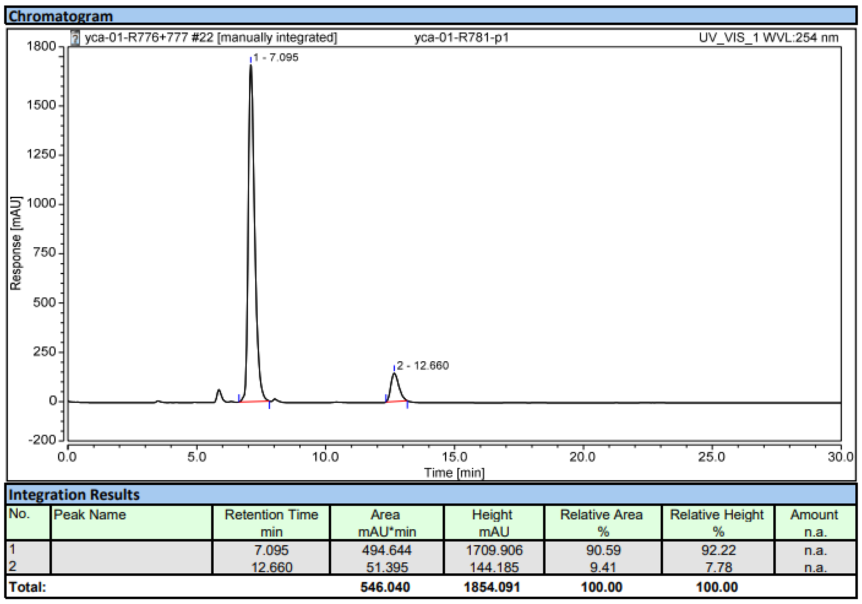

Integration Results

No.	Peak Name	Retention Time min	Area mAU*min	Height mAU	Relative Area %	Relative Height %	Amount n.a.
1		7.095	494.644	1709.906	90.59	92.22	n.a.
2		12.660	51.395	144.185	9.41	7.78	n.a.
**Total:**			**546.040**	**1854.091**	**100.00**	**100.00**	

(*R*)-2-((*R*)-cyclohex-2-en-1-yl)-2-(4-fluorophenyl)-3,4-dihydronaphthalen-1(2H)-one (**8v**):

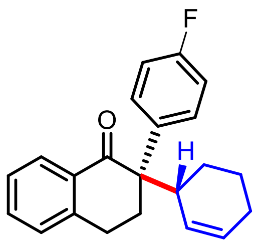

dr = >20:1

8v

Following **GP7**, using ketone **1v** and 1,4-cyclohexadiene **2b**, **8v** was obtained as a white solid in 58% yield (18.6 mg) dr = >20:1. **^{1}H NMR** (400 MHz, $CDCl_3$) δ 8.14 (dd, J = 7.9, 1.6 Hz, 1H), 7.39 (td, J = 7.4, 1.6 Hz, 1H), 7.35 – 7.22 (m, 3H), 7.10 (d, J = 7.7 Hz, 1H), 7.03 – 6.93 (m, 2H), 5.78 – 5.64 (m, 1H), 5.52 – 5.40 (m, 1H), 3.28 – 3.15 (m, 1H), 3.12 – 2.99 (m, 1H), 2.93 – 2.83 (m, 1H), 2.64 – 2.52 (m, 1H), 2.43 – 2.31 (m, 1H), 2.01 – 1.89 (m, 2H), 1.77 – 1.66 (m, 1H), 1.52 – 1.37 (m, 1H), 1.27 – 1.15 (m, 1H), 1.10 – 1.00 (m, 1H). $[\alpha]_D^{20}$: -160.8 (c = 1.0, $CHCl_3$).

Chiral HPLC: SFC, Chiralpak IC; MeOH:CO_2 = 3%, 2 mL/min, 230 nm; t_R (major) = 2.4 min, t_R (minor) = 2.9 min, 93:7 er.

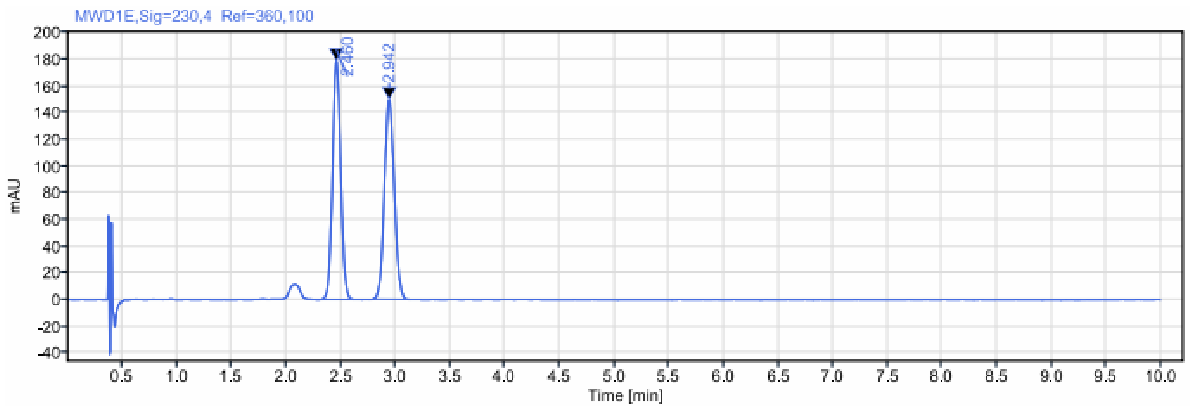

Signal: MWD1E,Sig=230,4 Ref=360,100

RT [min]	Type	Width [min]	Area	Height	Area%	Name
2.460	MM m	0.29	938.93	178.87	50.02	
2.942	MM m	0.35	938.24	149.95	49.98	

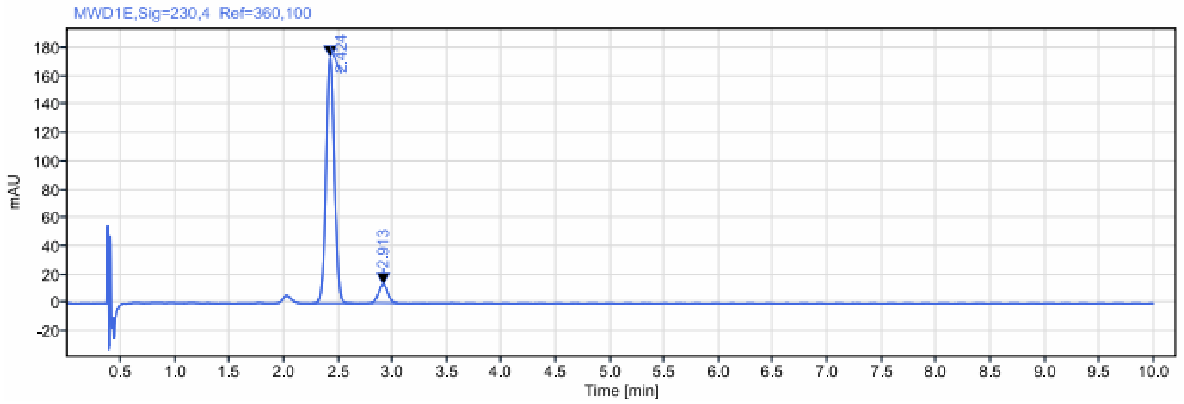

Signal: MWD1E,Sig=230,4 Ref=360,100

RT [min]	Type	Width [min]	Area	Height	Area%	Name
2.424	MM m	0.38	919.84	173.32	92.29	
2.913	MM m	0.24	76.87	12.79	7.71	

(*R*)-2-((*R*)-cyclohex-2-en-1-yl)-2-(4-methoxyphenyl)-3,4-dihydronaphthalen-1(2H)-one (**8w**):

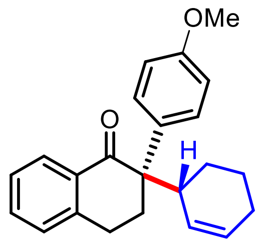

dr = >20:1
8w

Following **GP7**, using ketone **1w** and 1,4-cyclohexadiene **2b**, **8w** was obtained as a white solid in 57% yield (18.9 mg) dr = >20:1. **^{1}H NMR** (400 MHz, $CDCl_3$) δ 8.14 (d, J = 7.9 Hz, 1H), 7.40 – 7.33 (m, 1H), 7.28 – 7.20 (m, 3H), 7.08 (d, J = 7.7 Hz, 1H), 6.86 – 6.78 (m, 2H), 5.75 – 5.65 (m, 1H), 5.47 (d, J = 10.4 Hz, 1H), 3.75 (s, 3H), 3.26 – 3.15 (m, 1H), 3.09 (ddd, J = 17.4, 13.2, 4.5 Hz, 1H), 2.91 – 2.81 (m, 1H), 2.57 (ddd, J = 14.6, 4.5, 2.5 Hz, 1H), 2.41 – 2.26 (m, 1H), 2.00 – 1.89 (m, 2H), 1.75 – 1.66 (m, 1H), 1.51 – 1.39 (m, 1H),

1.25 – 1.15 (m, 1H), 1.13 – 1.04 (m, 1H). $[\alpha]_D^{20}$: -143.2 ° (c = 1.0, $CHCl_3$).

Chiral HPLC: SFC, Chiralpak IJ; $MeOH:CO_2$ = 0.5%, 2 mL/min, 230 nm; t_R (major) = 8.2 min, t_R (minor) = 7.6 min, 99:1 er.

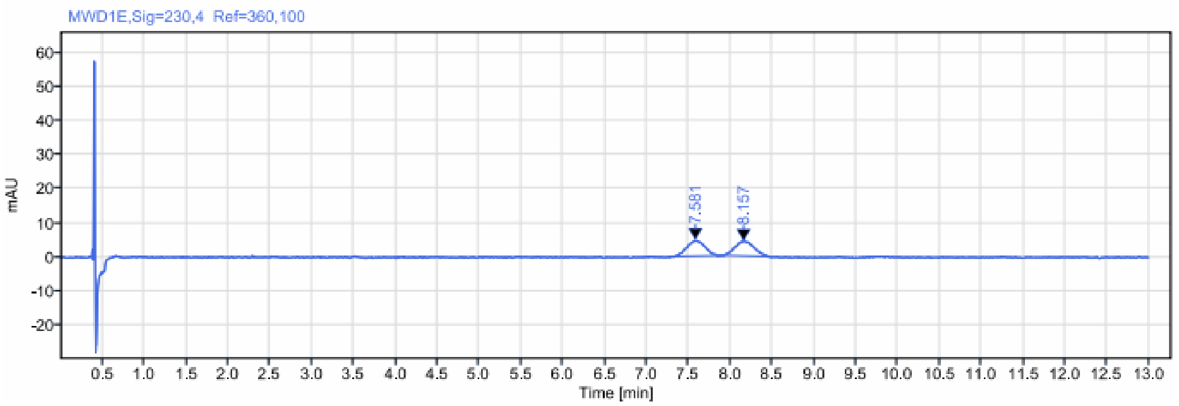

Signal: MWD1E,Sig=230,4 Ref=360,100

RT [min]	Type	Width [min]	Area	Height	Area%	Name
7.581	MM m	0.54	67.39	4.61	49.67	
8.157	MM m	0.54	68.28	4.29	50.33	

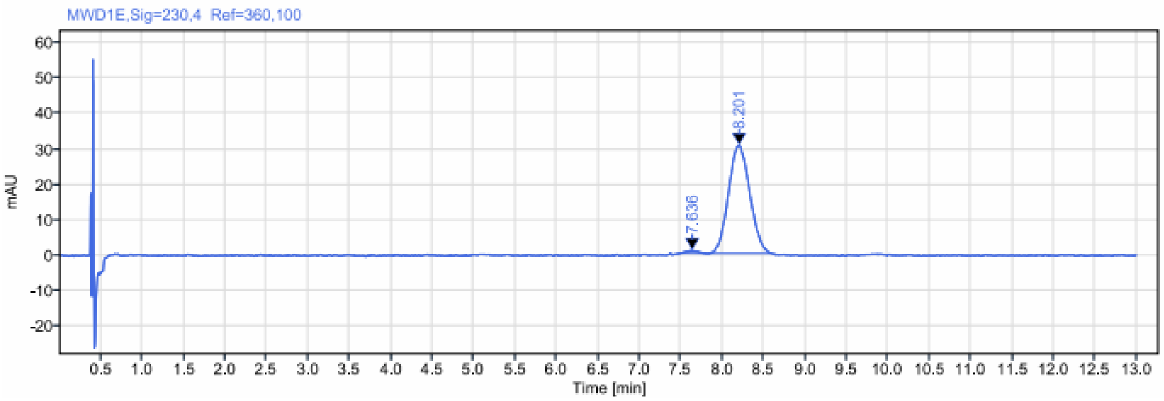

Signal: MWD1E,Sig=230,4 Ref=360,100

RT [min]	Type	Width [min]	Area	Height	Area%	Name
7.636	MM m	0.27	6.85	0.85	1.24	
8.201	MM m	0.82	544.31	30.55	98.76	

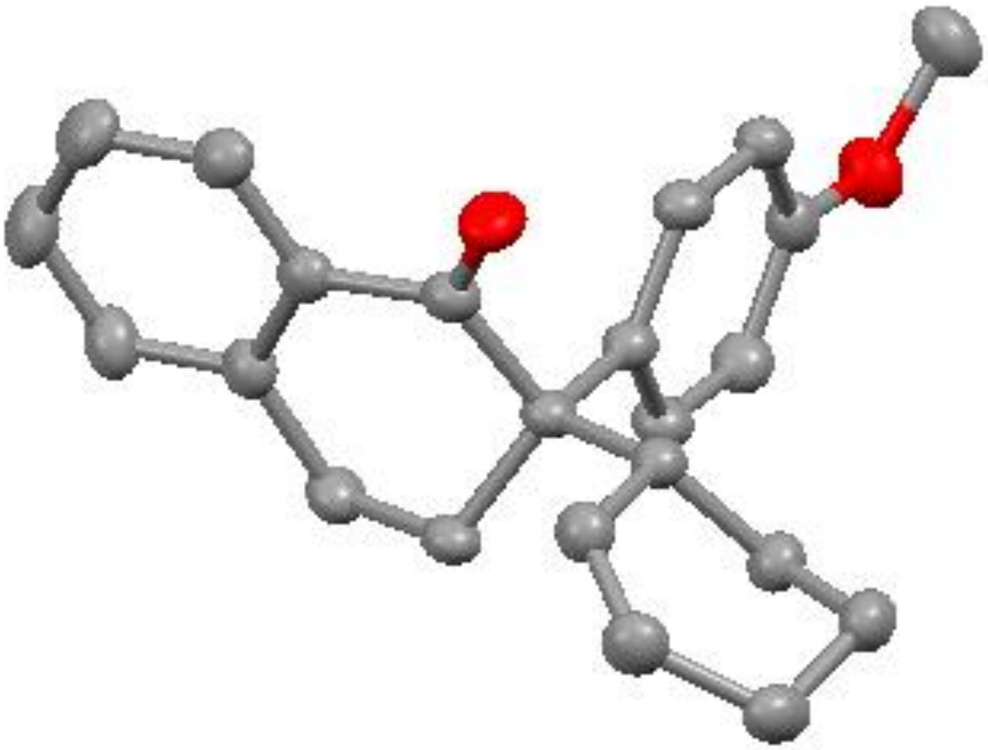

Supplementary Fig 4.9 | ORTEP diagram of **8w** (thermal ellipsoids are shown at the 50% probability level, for the reason of clarity all the hydrogen atoms are omitted). CCDC 2116594.

(*R*)-2-((*R*)-cyclohex-2-en-1-yl)-3,4-dihydro-[2,2'-binaphthalen]-1(2H)-one (**8x**):

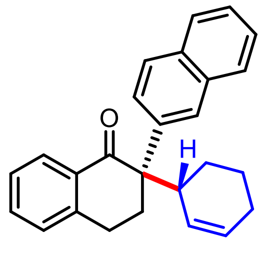

dr = >20:1
8x

Following **GP7**, using ketone **1x** and 1,4-cyclohexadiene **2b**, **8x** was obtained as a colourless thick oil in 50% yield (17.5 mg) dr = >20:1. **^{1}H NMR** (400 MHz, $CDCl_3$) δ 8.19 (dd, J = 7.9, 1.6 Hz, 1H), 7.83 – 7.69 (m, 4H), 7.54 (dd, J = 8.8, 2.0 Hz, 1H), 7.47 – 7.39 (m, 2H), 7.35 (td, J = 7.4, 1.6 Hz, 1H), 7.28 – 7.22 (m, 1H), 7.07 (d, J = 7.7 Hz, 1H), 5.80 – 5.68 (m, 1H), 5.53 (dt, J = 10.3, 1.9 Hz, 1H), 3.41 – 3.27 (m, 1H), 3.23 – 3.10 (m, 1H), 2.97 – 2.87 (m, 1H), 2.82 – 2.70 (m, 1H), 2.46 (ddd, J = 14.6, 13.2, 4.7 Hz, 1H), 2.03 – 1.89 (m, 2H), 1.73 – 1.63 (m, 1H), 1.50 – 1.37 (m, 1H), 1.35 – 1.24 (m, 1H), 1.15 – 1.03 (m, 1H). $[\alpha]_D^{20}$**:** -129.5 ° (c = 1.0, $CHCl_3$).

Chiral HPLC: SFC, Chiralpak IC; MeOH:CO_2 = 5%, 2 mL/min, 230 nm; t_R (major) = 5.2 min, t_R (minor) = 5.8 min, 91:9 er.

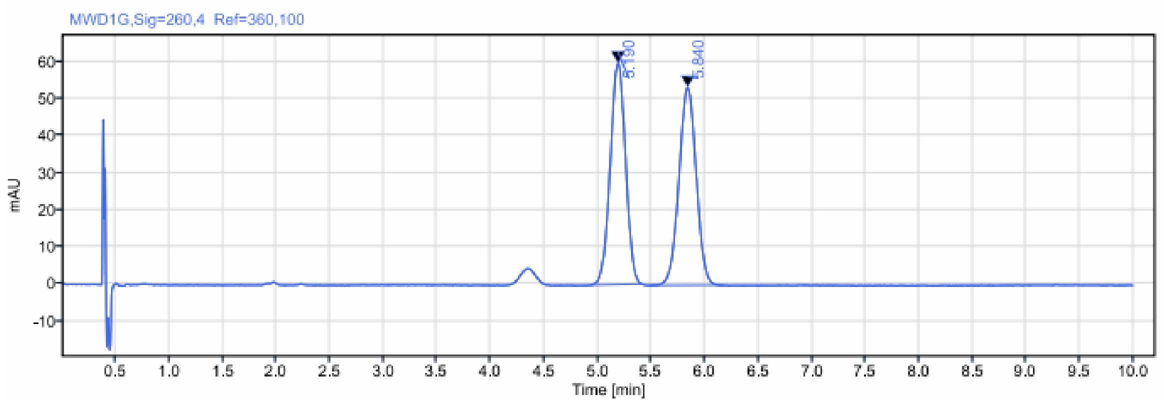

Signal: MWD1G,Sig=260,4 Ref=360,100

RT [min]	Type	Width [min]	Area	Height	Area%	Name
5.190	MM m	0.48	593.42	59.69	49.74	
5.840	MM m	0.68	599.68	53.37	50.26	

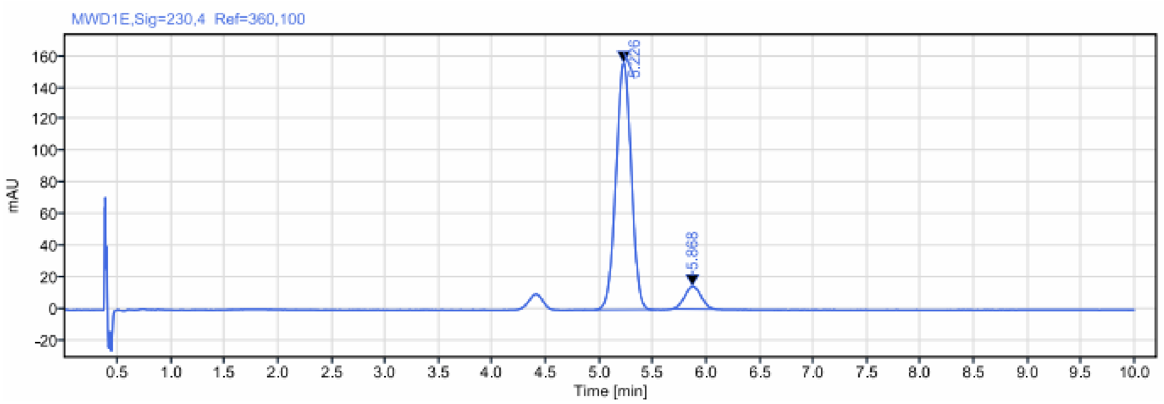

Signal: MWD1E,Sig=230,4 Ref=360,100

RT [min]	Type	Width [min]	Area	Height	Area%	Name
5.226	MM m	0.55	1555.54	155.50	91.17	
5.868	MM m	0.40	150.66	14.19	8.83	

(*R*)-2-((*R*)-cyclohex-2-en-1-yl)-7-methyl-2-phenyl-3,4-dihydronaphthalen-1(2H)-one (**8y**):

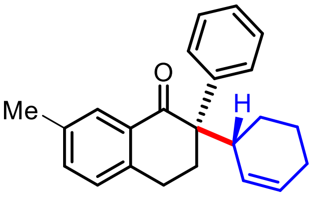

dr = >20:1
8y

Following **GP7**, using ketone **1y** and 1,4-cyclohexadiene **2b**, **8y** was obtained as a white solid in 82% yield (25.8 mg) dr = >20:1. **^{1}H NMR** (400 MHz, $CDCl_3$) δ 7.96 (s, 1H), 7.37 – 7.25 (m, 4H), 7.23 – 7.16 (m, 2H), 6.98 (d, J = 7.8 Hz, 1H), 5.77 – 5.66 (m, 1H), 5.52 – 5.43 (m, 1H), 3.23 (dt, J = 5.9, 2.9 Hz, 1H), 3.06 (ddd, J = 17.4, 13.1, 4.5 Hz, 1H), 2.83 (ddd, J = 17.1, 4.8, 2.6 Hz, 1H), 2.61 (ddd, J = 14.6, 4.5, 2.5 Hz, 1H), 2.40 – 2.28 (m, 4H), 2.00 – 1.90 (m, 2H), 1.74 – 1.65 (m, 1H), 1.51 – 1.38 (m, 1H), 1.28 – 1.17 (m, 1H), 1.11 – 1.00 (m, 1H). $[\boldsymbol{\alpha}]_D^{20}$**:** -138.2 ° (c = 1.0, $CHCl_3$).

Chiral HPLC: Chiralpak IC 4.6 x 250 mm; hexane:*i*-PrOH 99:1, 0.7 mL/min, 230 nm; t_R (major) = 7.5 min, t_R (minor) = 20.0 min, 92:8 er.

Chromatogram

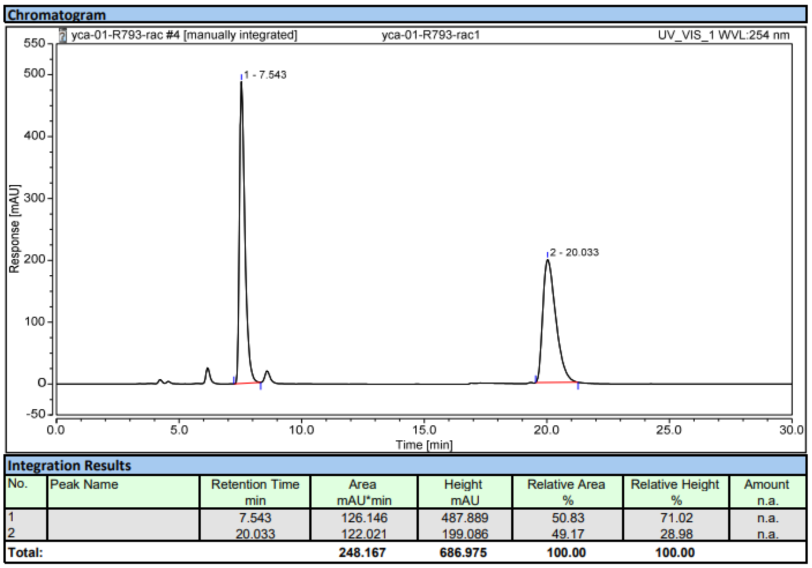

Integration Results

No.	Peak Name	Retention Time min	Area mAU*min	Height mAU	Relative Area %	Relative Height %	Amount n.a.
1		7.543	126.146	487.889	50.83	71.02	n.a.
2		20.033	122.021	199.086	49.17	28.98	n.a.
Total:			248.167	686.975	100.00	100.00	

Chromatogram

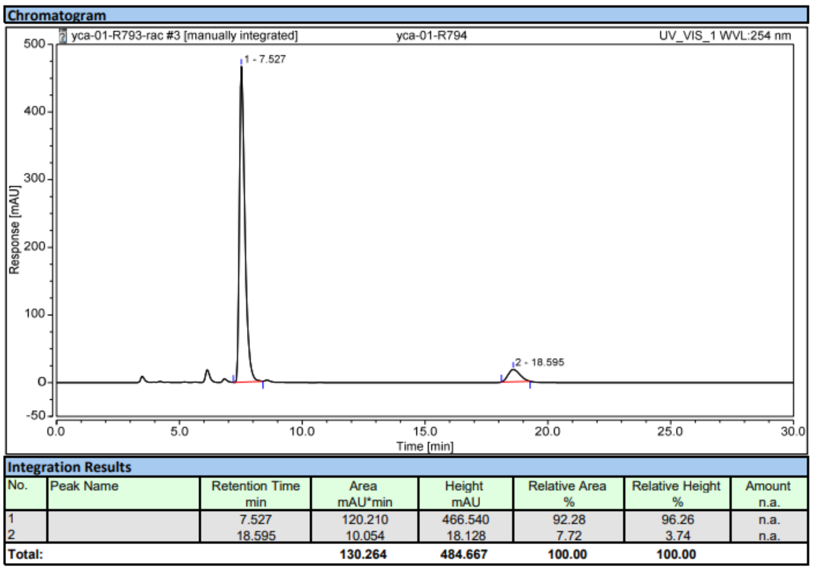

Integration Results

No.	Peak Name	Retention Time min	Area mAU*min	Height mAU	Relative Area %	Relative Height %	Amount n.a.
1		7.527	120.210	466.540	92.28	96.26	n.a.
2		18.595	10.054	18.128	7.72	3.74	n.a.
Total:			130.264	484.667	100.00	100.00	

(*R*)-2-((*R*)-cyclohex-2-en-1-yl)-7-fluoro-2-(4-methoxyphenyl)-3,4-dihydronaphthalen-1(2H)-one (**8z**):

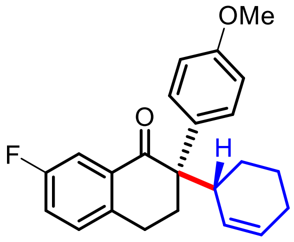

dr = >20:1
8z

Following **GP7**, using ketone **1z** and 1,4-cyclohexadiene **2b**, **8z** was obtained as a white solid in 45% yield (15.6 mg) dr = >20:1. ^{1}**H NMR** (400 MHz, $CDCl_3$) δ 7.83 – 7.75 (m, 1H), 7.24 – 7.19 (m, 2H), 7.10 – 7.02 (m, 2H), 6.86 – 6.80 (m, 2H), 5.75 – 5.67 (m, 1H), 5.43 (dt, J = 10.3, 1.9 Hz, 1H), 3.75 (s, 3H), 3.24 – 3.14 (m, 1H), 3.09 – 2.98 (m, 1H), 2.88 – 2.79 (m, 1H), 2.61 – 2.53 (m, 1H), 2.37 – 2.27 (m, 1H), 2.00 – 1.90 (m, 2H), 1.75 – 1.67 (m, 1H), 1.50 – 1.38 (m, 1H), 1.23 – 1.16 (m, 1H), 1.13 – 1.05 (m, 1H). $[\alpha]_D^{20}$: -137.5 ° (c = 1.0, $CHCl_3$).

Chiral HPLC: SFC, Chiralpak IJ; MeOH:CO_2 = 0.5%, 2 mL/min, 230 nm; t_R (major) = 6.3 min, t_R (minor) = 5.1 min, 95:5 er.

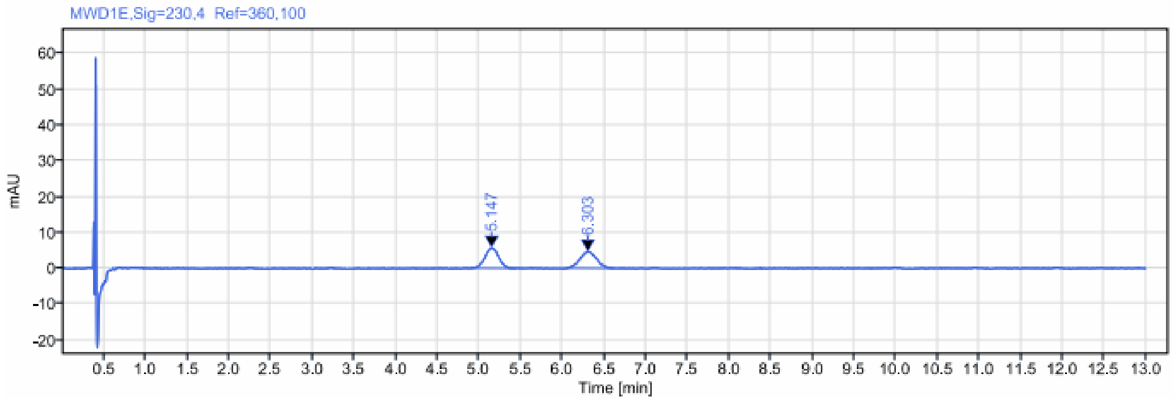

Signal: MWD1E,Sig=230,4 Ref=360,100

RT [min]	Type	Width [min]	Area	Height	Area%	Name
5.147	MM m	0.54	64.39	5.73	50.11	
6.303	MM m	0.56	64.11	4.63	49.89	

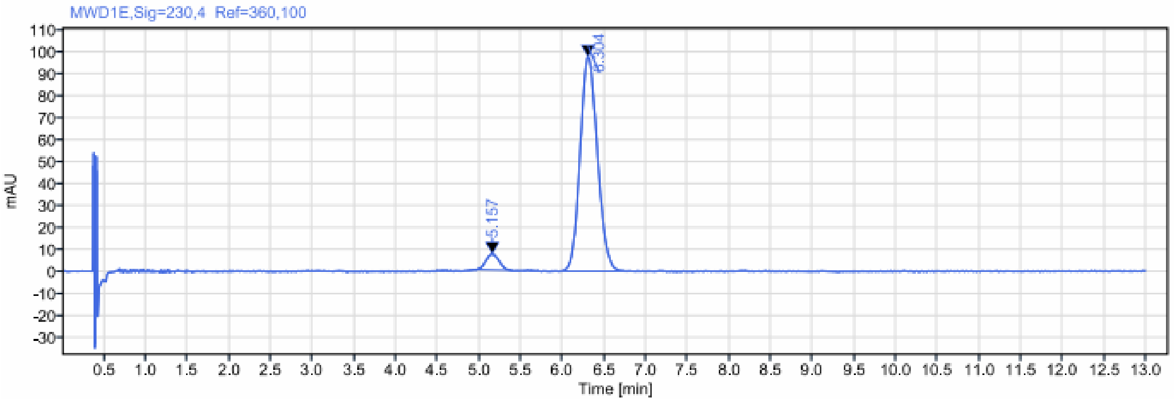

Signal: MWD1E,Sig=230,4 Ref=360,100

RT [min]	Type	Width [min]	Area	Height	Area%	Name
5.157	MM m	0.45	78.51	7.13	5.28	
6.304	MM m	0.88	1409.28	97.40	94.72	

5. Mechanistic studies.

5.1 Nickel complex synthesis.

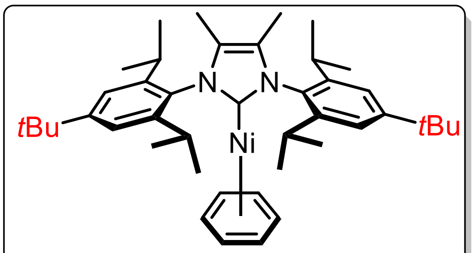

[^{Me}IPr^{tBu}-Ni(η^6-C_6H_6)]

9

A slight modification of a literature procedure[21, 22, 23] was used to synthesize **9**. A reaction tube was charged with Ni(cod)$_2$ (55.0 mg, 0.2 mmol), ^{Me}IPr^{tBu} (105.8 mg, 0.2 mmol), and benzene (2 mL). The resulting solution was transferred into an autoclave reactor, and then H_2 was pressurized quickly (recommended within 5 min) into the reactor at 50 atm. After the reaction mixture was stirred at room temperature overnight (~16 h), the resultant red solution was filtered, and all volatiles were removed under reduced pressure to give [^{Me}IPr^{tBu}-Ni(η^6-C_6H_6)] as a reddish brown solid (126.0 mg, 0.189 mmol, 90% yield). **^{1}H NMR** (400 MHz, Benzene-*d*$_6$) δ 7.44 (s, 4H), 2.97 – 2.82 (m, 4H), 1.63 (s, 6H), 1.56 (d, *J* = 6.5 Hz, 12H), 1.41 (s, 18H). 1.22 (d, *J* = 6.6 Hz, 12H). **^{13}C NMR** (101 MHz, Benzene-*d*$_6$) δ 193.2, 150.9, 146.3, 135.4, 122.2, 120.2, 88.0 – 87.4 (m), 35.2, 31.7, 29.0, 27.0, 24.5, 24.1, 10.2.

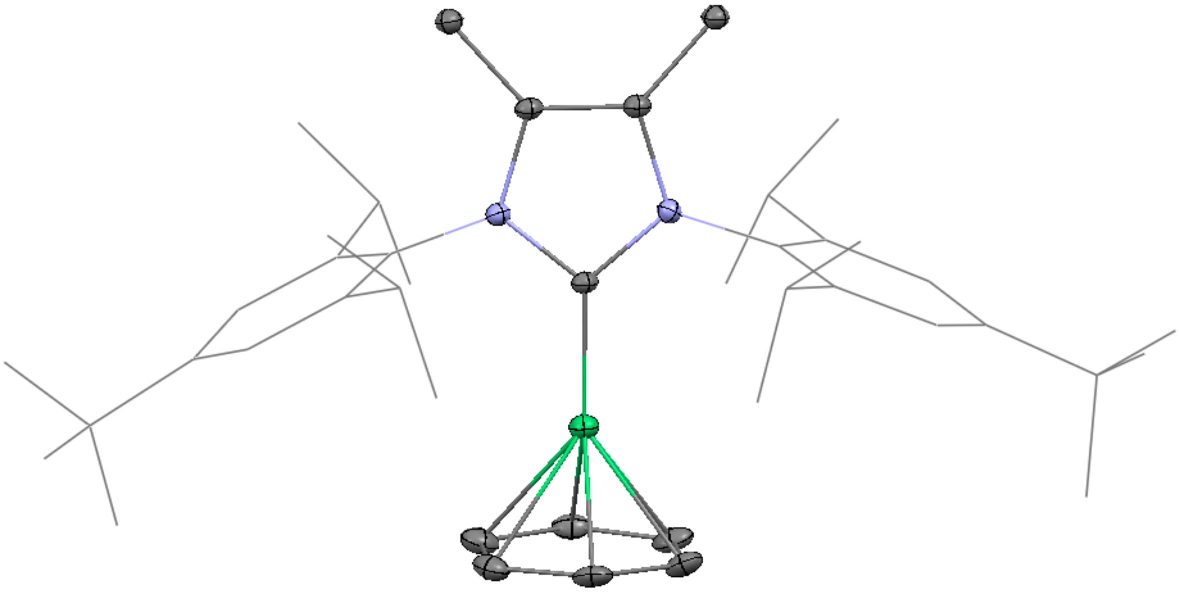

Supplementary Fig 5.1 | ORTEP diagram of **9** (thermal ellipsoids are shown at the 50% probability level, for the reason of clarity all the hydrogen atoms are omitted). CCDC 2218583.

5.2 Calculation of percent Buried Volume (%V_{bur}).

Calculations of percent buried volume[26] were performed with the SambVca 2.1[27] web tool. The following parameters (set with respect to the parameters suggested by Nolan) were employed for the calculations:

- Center of sphere: NHC carbon
- Atoms for Z-axis definition: nitrogen atoms in NHC (Z-negative)
- Atoms for XZ plane definition: one of the nitrogen atoms in NHC
- All non-NHC atoms were deleted
- Bondii radii scaled by 1.17
- Sphere radius: either 3.5 Å (suggested parameter) or 5.5 Å (expanded radius parameter)
- Distance of coordination point from center of sphere: 2.0 Å
- Mesh spacing for numerical integration: 0.1
- no H atoms included in calculation

A representative calculation for [^{Me}IPr^{tBu}-Ni(η^6-C_6H_6)] (radius of 3.5 Å) is shown below:

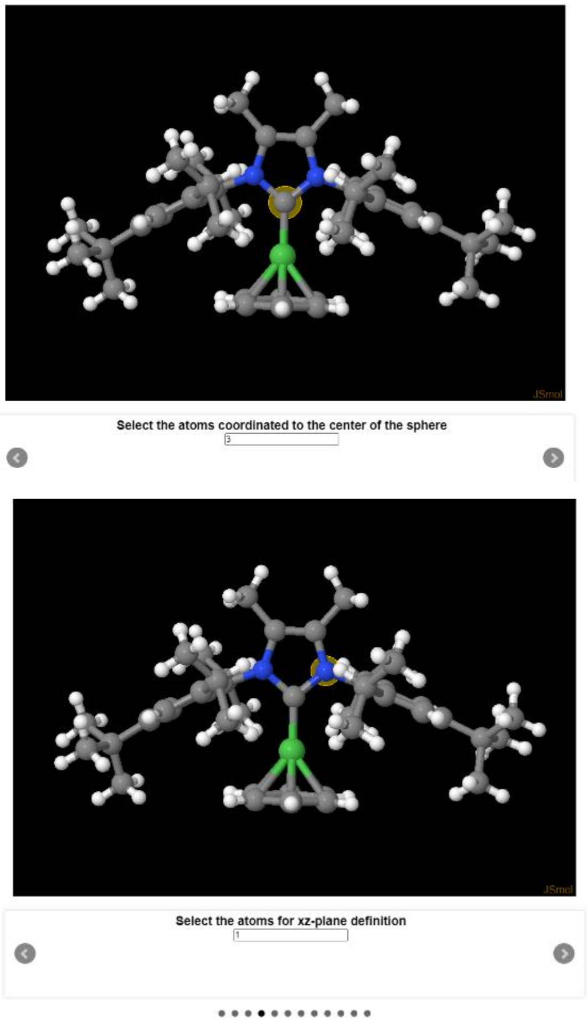

Supplementary Fig 5.2 | Comprehensive procedures for Buried Volume calculation 1.

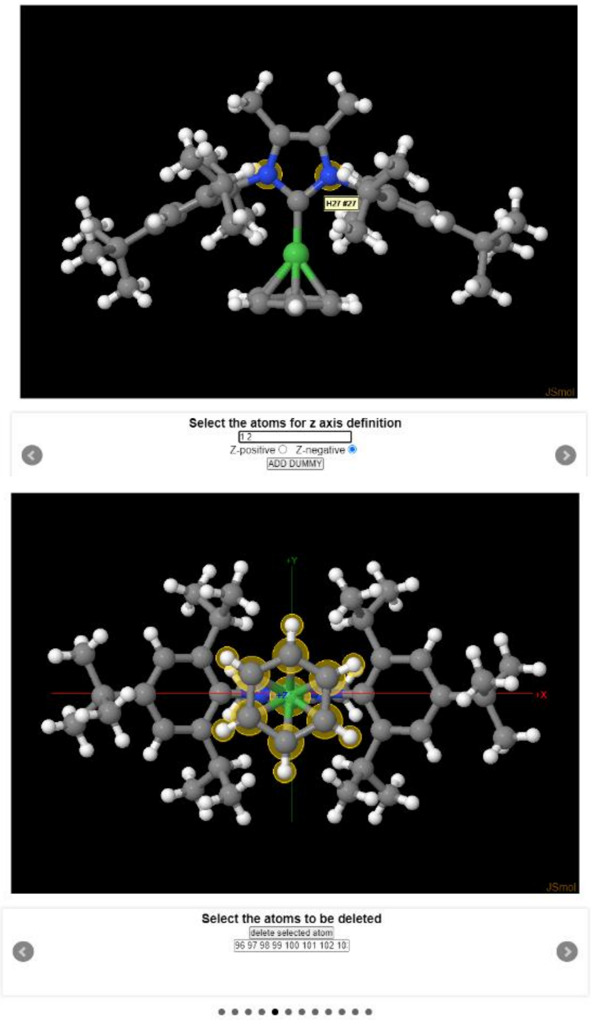

Supplementary Fig 5.2 | Comprehensive procedures for Buried Volume calculation 2.

SambVca 2.1 - Results

HOME HELP LIBRARY INFO DOWNLOAD

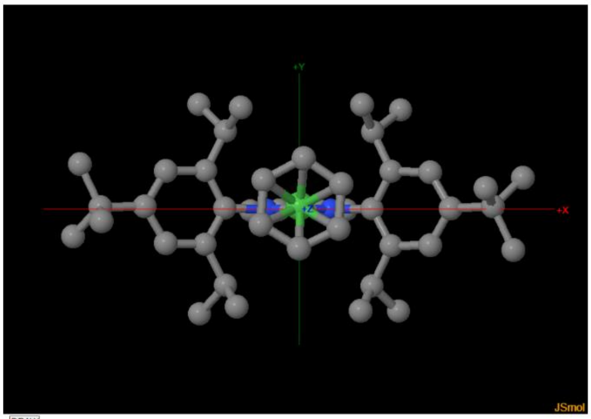

DRAW

%V Free	%V Buried	% V Tot/V Ex
56.1	43.9	99.9

Quadrant	V f	V b	V t	%V f	%V b
SW	23.8	21.1	44.9	53.1	46.9
NW	25.5	19.3	44.9	56.9	43.1
NE	26.4	18.5	44.9	58.8	41.2
SE	24.9	19.9	44.9	55.5	44.5

Steric Map

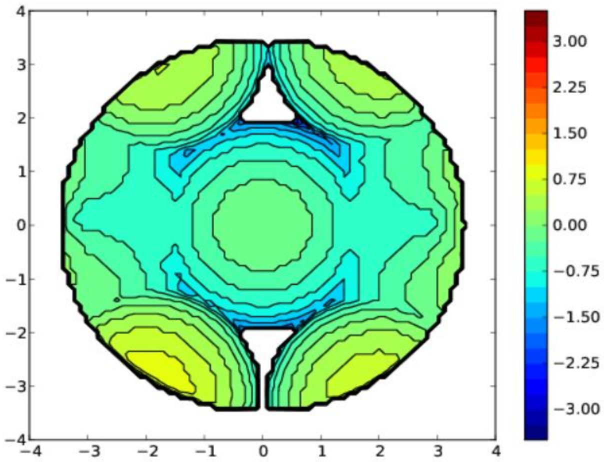

Supplementary Fig 5.3 | Result for Buried Volume (%V_{bur}) of [^{Me}IPr^{tBu}-Ni(η^6-C_6H_6)] (radius of 3.5 Å):

SambVca 2.1 - Results

HOME HELP LIBRARY INFO DOWNLOAD

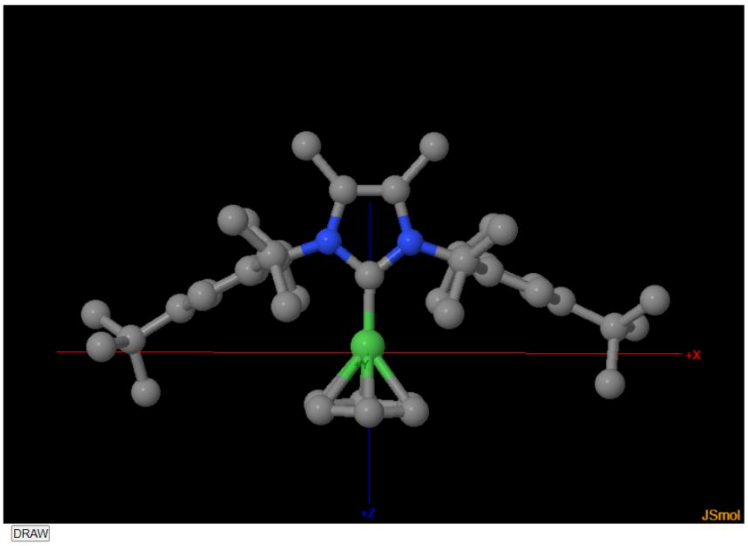

%V Free	%V Buried	% V Tot/V Ex
53.2	46.8	99.9

Quadrant	V f	V b	V t	%V f	%V b
SW	92.0	82.0	174.1	52.9	47.1
NW	92.2	81.9	174.1	52.9	47.1
NE	91.4	82.6	174.1	52.5	47.5
SE	94.6	79.5	174.1	54.4	45.6

Steric Map

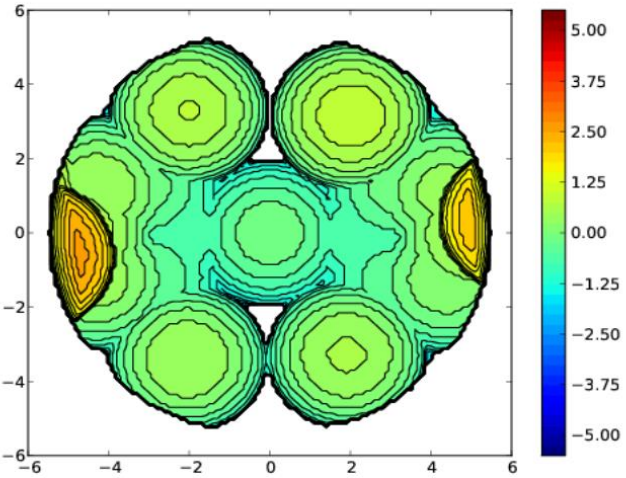

Supplementary Fig 5.4 | Result for Buried Volume (%V_{bur}) of [^{Me}IPr^{tBu}-Ni(η^6-C_6H_6)] (radius of 3.5 Å):

5.3 *Reactivity investigation of nickel complex.*

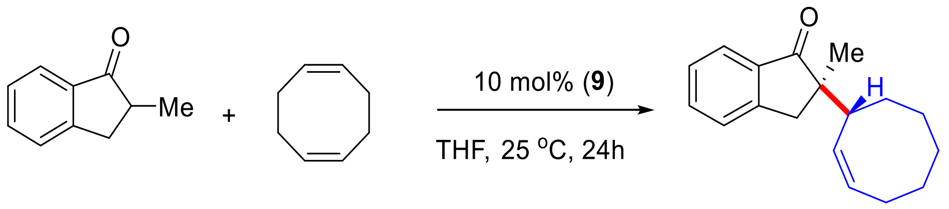

1i **2a** **3i**

65%, 6.3:1 dr

In a glovebox, an oven dried screw-capped 2 mL vial was charged with a magnetic stir bar, [^{Me}IPr^{tBu}-Ni(η^6-C_6H_6)] **9** (10 μmol), degassed (freeze pump thaw) THF (0.1 mL) was then added ketone **1i** (0.1 mmol degassed freeze pump thaw if the ketone is liquid) and COD **2a** (0.2 mmol degassed by freeze pump thaw) was then added successively. The vial was sealed with a Teflon-lined screw cap, and running inside the glovebox at room temperature. After 24 h, the vial was shipped outside of the glovebox. The reaction mixture was diluted with dichloromethane and filtered through a plug of silica gel. The crude solution was concentrated in vacuum and subjected to column chromatography 100:1 PE/EA to isolate the products **3i** with 65% yield and 6.3:1 dr.

5.4 NMR experiment 1.

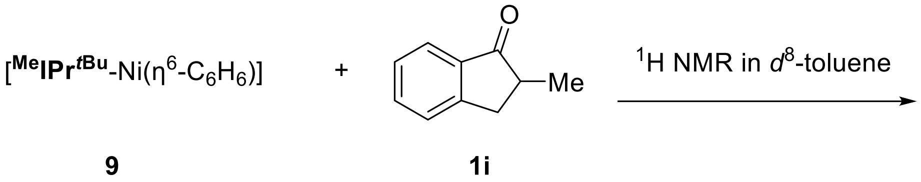

In a glovebox, an oven dried screw-capped 2 mL vial was charged with [^{Me}IPr^{tBu}-Ni(η^6-C_6H_6)] **9** (25 μmol), degassed (freeze pump thaw) toluene-d_8 (0.25 mL) was then added. Another oven dried screw-capped 2 mL vial was charged with ketone **1i** (25 μmol), degassed (freeze pump thaw) toluene-d_8 (0.25 mL) was then added. Both vials were pre-cooled to -78 ^{o}C. Then mix both solution and added in to J-Young NMR tube. The tube was immediately taken out of glovebox and insert into dry ice acetone bath. The tube was shaken and quickly placed into the probe of the NMR spectrometer pre-cooled to −80 °C. The probe temperature was increased gradually and tested by ^{1}H NMR every 20 ^{o}C gradient (the temperature was stable for 10 min before starting scanning).

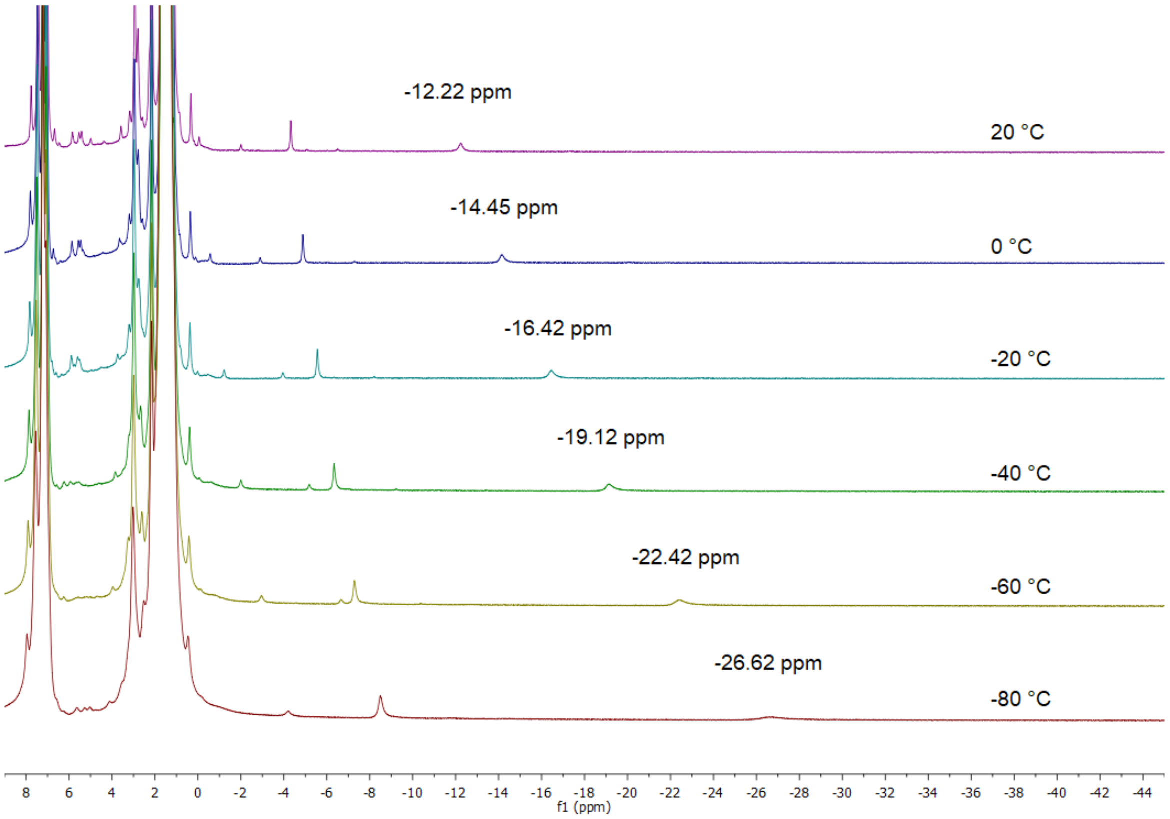

Supplementary Fig 5.5 | Observation of hydride signal on ^{1}H NMR.

5.5 NMR experiment 2:

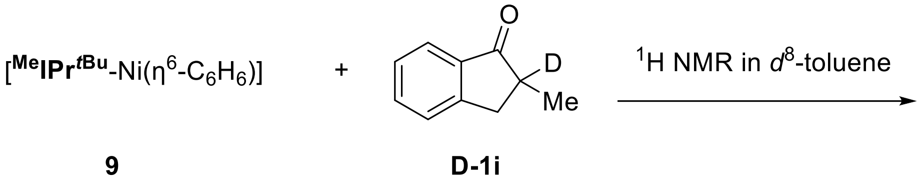

In a glovebox, an oven dried screw-capped 2 mL vial was charged with [${}^{Me}IPr^{tBu}$-Ni(η^6-C_6H_6)] **9** (25 µmol), degassed (freeze pump thaw) toluene-d_8 (0.25 mL) was then added. Another oven dried screw-capped 2 mL vial was charged with ketone **D-1i** (25 µmol), degassed (freeze pump thaw) toluene-d_8 (0.25 mL) was then added. Both vials were pre-cooled to -78 °C. Then mix both solution and added in to J-Young NMR tube. The tube was immediately taking out of glovebox and insert into dry ice acetone bath. The tube was shaken and quickly placed into the probe of the NMR spectrometer pre-cooled to −80 °C. The probe temperature was increased gradually and tested by ^{1}H NMR every 20 °C gradient (the temperature was stable for 10 min before starting scanning).

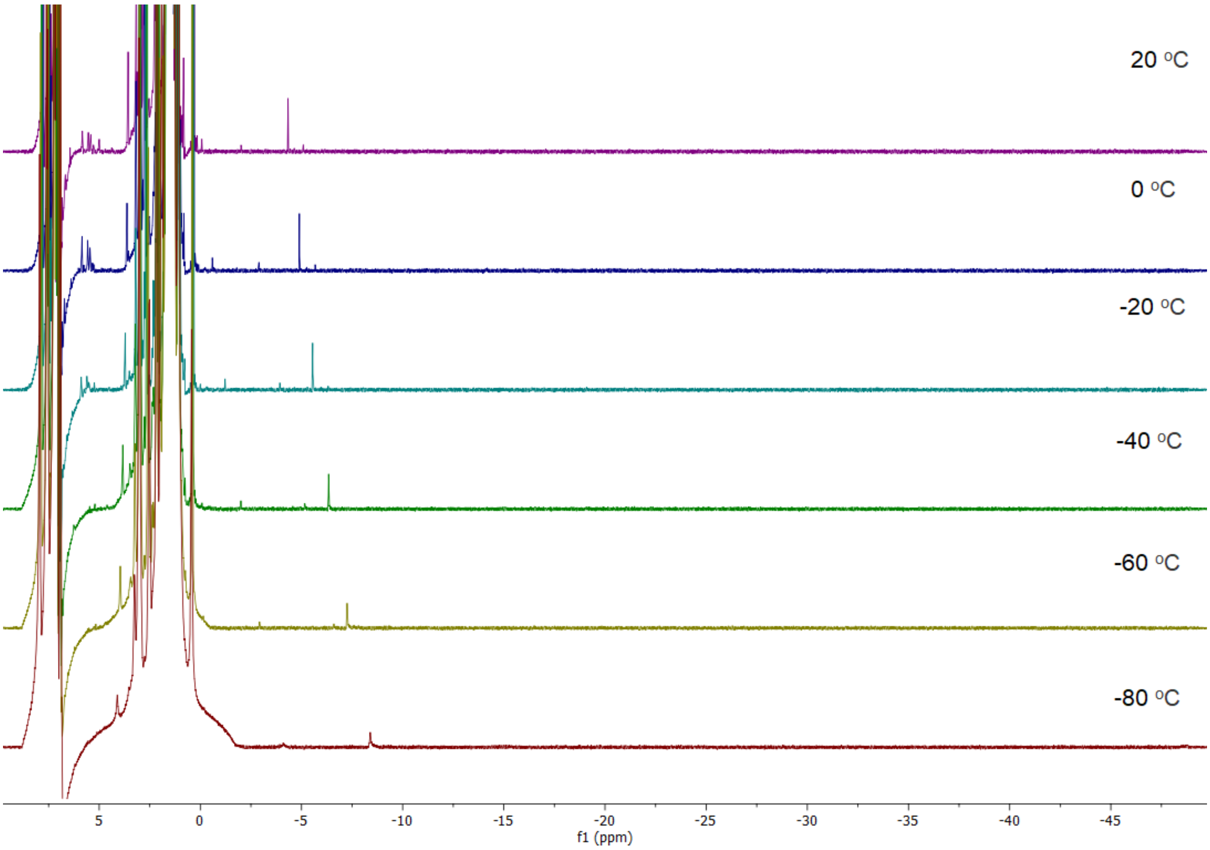

Supplementary Fig 5.6 | Vanishing of hydride signal on ^{1}H NMR.

5.6 *NMR experiment 3:*

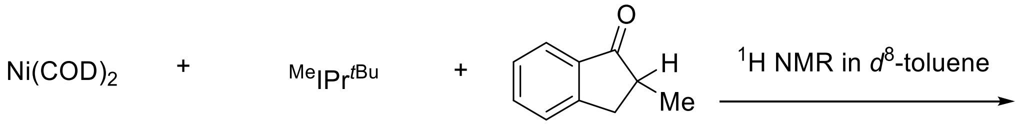

1i

In a glovebox, an oven dried screw-capped 2 mL vial with stirring bar was charged $Ni(COD)_2$ (25 μmol), $^{Me}IPr^{tBu}$ (25 μmol), degassed (freeze pump thaw) toluene-d_8 (0.25 mL) was then added , mixture was stirred at room temperature for 30 min. Another oven dried screw-capped 2 mL vial was charged with ketone **1i** (25 μmol), degassed (freeze pump thaw) toluene-d_8 (0.25 mL) was then added. Both vials were pre-cooled to -78 ^{o}C. Then mix both solution and added in to J-Young NMR tube. The tube was immediately taken out of glovebox and insert into dry ice acetone bath. The tube was shaken and quickly placed into the probe of the NMR spectrometer pre-cooled to −80 °C. The probe temperature was increased gradually and tested by ^{1}H NMR every 20 ^{o}C gradient (the temperature was stable for 10 min before starting scanning).

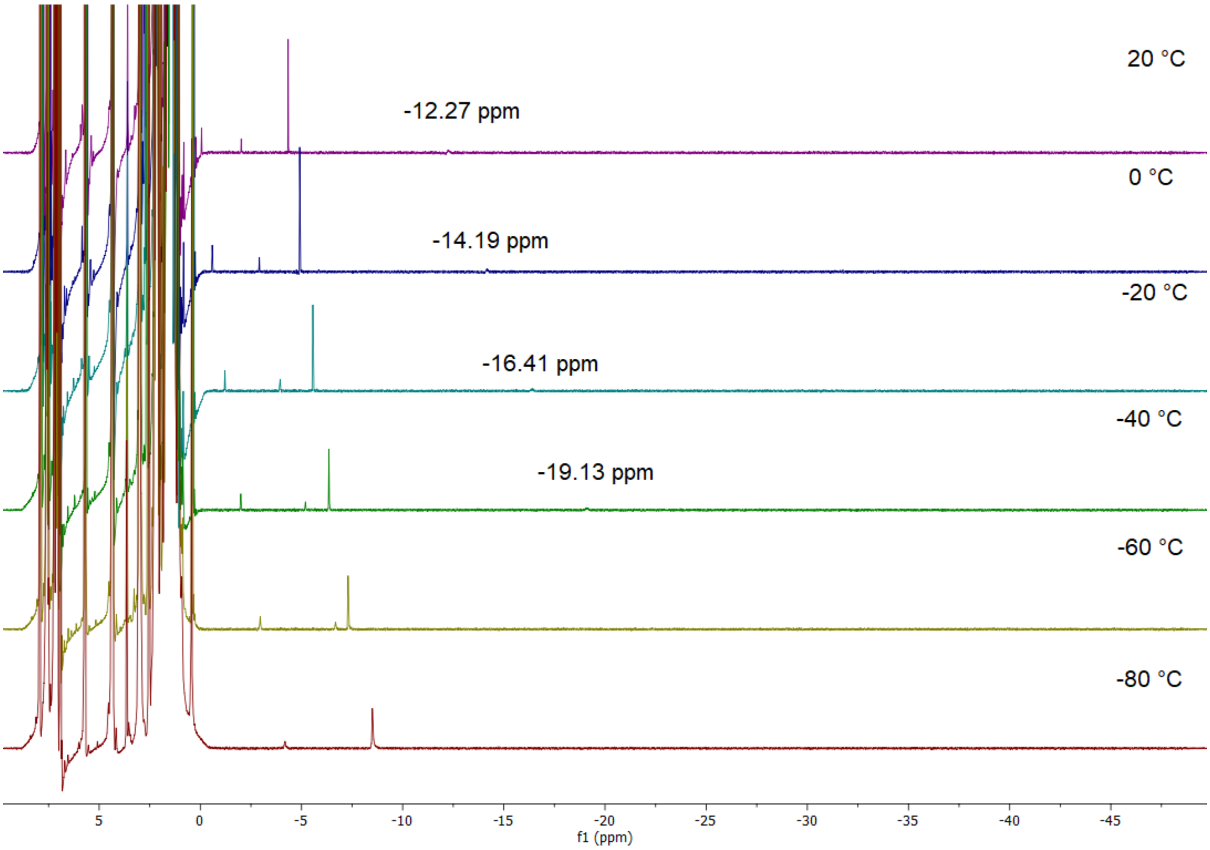

Supplementary Fig 5.7 | Suppression of hydride signal on ^{1}H NMR.

5.7 *Deuterium labelling experiment.*

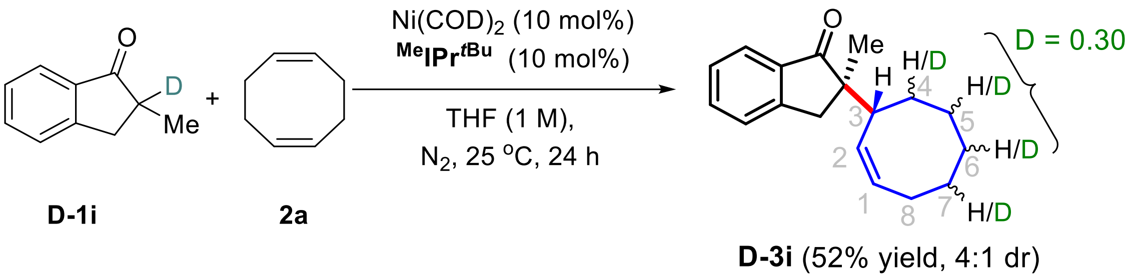

In a glovebox, an oven dried screw-capped 2 mL vial was charged with a magnetic stir bar, $Ni(COD)_2$ (10 μmol) , carbene ligand $^{Me}IPr^{tBu}$ (10 μmol), degassed (freeze pump thaw) THF (0.1 mL) was then added and the catalyst mixture was stirred at room temperature for 30 min, during which a dark red solution was formed. Deuterated ketone **D-1i** (0.1 mmol degassed freeze pump thaw) and COD **2a** (0.15 mmol degassed by freeze pump thaw) was then added successively. The vial was sealed with a Teflon-lined screw cap, and running inside the glovebox at room temperature. After 24 h, the vial was shipped outside of the glovebox. The reaction mixture was diluted with dichloromethane and filtered through a plug of silica gel. The crude solution was concentrated in vacuum and subjected to column chromatography 100:1 PE/EA to isolate the product **D-3i** yield 52% dr 4.0:1.

Deuterated (*Z*)-2-(cyclooct-2-en-1-yl)-2-methyl-2,3-dihydro-1H-inden-1-one (**D-3i**):

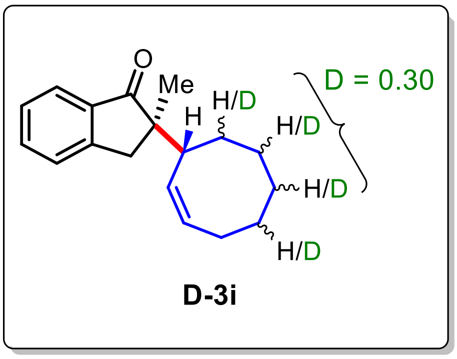

^{1}H NMR (400 MHz, $CDCl_3$) δ 7.71 – 7.63 (m, 1H), 7.55 – 7.47 (m, 1H), 7.42 – 7.34 (m, 1H), 7.33 – 7.25 (m, 1H), 5.73 (dddd, *J* = 10.2, 8.6, 7.3, 1.2 Hz, 0.20H), 5.54 (dddd, *J* = 10.7, 8.6, 7.3, 1.1 Hz, 0.85H), 5.31 (ddd, *J* = 10.7, 9.4, 1.5 Hz, 0.20H), 5.10 (ddd, *J* = 10.8, 9.3, 1.5 Hz, 0.85H), 3.31 – 3.11 (m, 1H), 2.99 – 2.90 (m, 0.20H). 2.88 – 2.62 (m, 2H), 2.33 – 2.11 (m, 1H), 2.04 – 1.87 (m, 1H), 1.77 – 1.66 (m, 0.89H), 1.65 – 1.55 (m, 1.63H), 1.50 – 1.25 (m, 3H), 1.21 – 1.06 (m, 5H), 0.99 – 0.88 (m, 0.20H). **^{2}H NMR** (61 MHz, $CHCl_3$) δ 2.22 – 1.13 (m)**.** **^{13}C NMR** (101 MHz, $CDCl_3$) δ 212.1, 211.5, 153.4, 153.1, 137.0, 136.8, 134.9, 134.8, 131.8, 131.2, 130.4, 129.5, 127.5, 127.5, 126.7, 126.5, 124.3, 124.1, 52.2, 51.4, 42.8, 42.3, 38.8, 37.4, 31.2, 30.8 – 30.4 (m), 29.7 – 29.3 (m), 27.5 – 26.4 (m), 26.0 – 25.4 (m), 25.0, 22.8. **IR (ATR): $\tilde{\nu}$ (cm^{-1}) =** 2920, 2852, 1705, 1607, 1463, 1450, 1324, 1296, 1281, 1206, 1187, 970, 798, 737, 712. **HRMS (ESI) (m/z) calcd. for $C_{18}H_{22}DO^+$:** 256.1806; Found: 256.1802. **R_f:** 0.60 (Pentane:EA 10:1).

5.8 *Deuterium crossover experiment.*

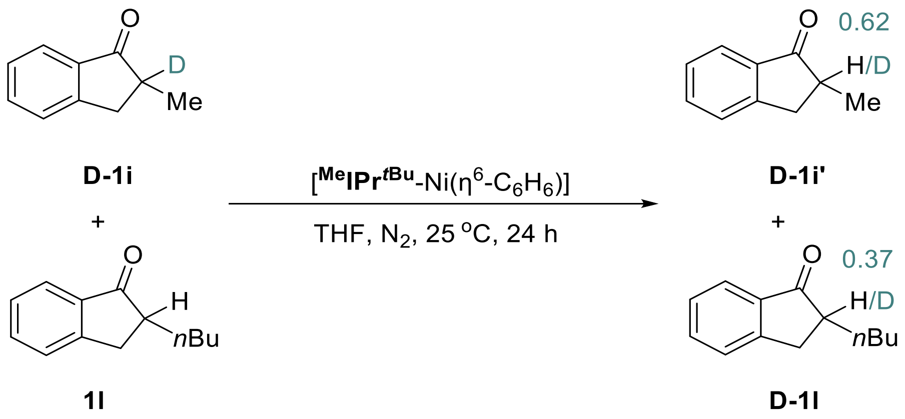

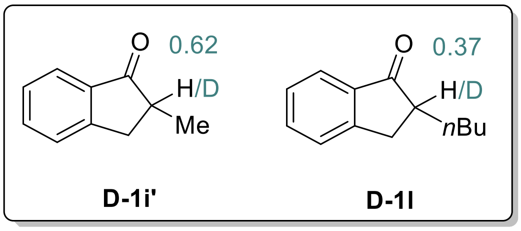

In a glovebox, an oven dried screw-capped 2 mL vial was charged with a magnetic stir bar, [(η6 -arene)Ni(^{Me}IPr^{tBu})] (10 μmol), degassed (freeze pump thaw) THF (0.1 mL) was then added ketone **D-1i** (0.05 mmol degassed by freeze pump thaw) and ketone **1l** (0.05 mmol degassed by freeze pump thaw) was then added successively. The vial was sealed with a Teflon-lined screw cap, and running inside the glovebox at room temperature. After 24 h, the vial was shipped outside of the glovebox. The reaction mixture was diluted with dichloromethane and filtered through a plug of silica gel. The crude solution was concentrated in vacuum and subjected to column chromatography 100:1 PE/EA.

D-1i' ^{1}H NMR (400 MHz, $CDCl_3$) δ 7.76 (d, *J* = 7.6 Hz, 1H), 7.59 (t, *J* = 7.4 Hz, 1H), 7.45 (d, *J* = 7.7 Hz, 1H), 7.37 (t, *J* = 7.7 Hz, 1H), 3.41 (dd, *J* = 17.4, 7.6 Hz, 1H), 2.82 – 2.63 (m, 1.62H), 1.32 (d, *J* = 6.6 Hz, 3H).

D-1l ^{1}H NMR (400 MHz, $CDCl_3$) δ 7.75 (d, *J* = 7.7 Hz, 1H), 7.58 (t, *J* = 7.4 Hz, 1H), 7.45 (d, *J* = 7.7 Hz, 1H), 7.36 (t, *J* = 7.4 Hz, 1H), 3.32 (dd, *J* = 17.2, 7.5 Hz, 1H), 2.82 (dd, *J* = 17.2, 3.9 Hz, 1H), 2.65 (tt, *J* = 8.3, 4.1 Hz, 0.61H), 2.02 – 1.90 (m, 1H), 1.41 (dddd, *J* = 28.7, 17.7, 9.0, 5.0 Hz, 6H), 1.21 (dd, *J* = 21.4, 6.9 Hz, 0.63H), 0.91 (t, *J* = 7.1 Hz, 3H).

5.9 *Deuterium scrambling experiment.*

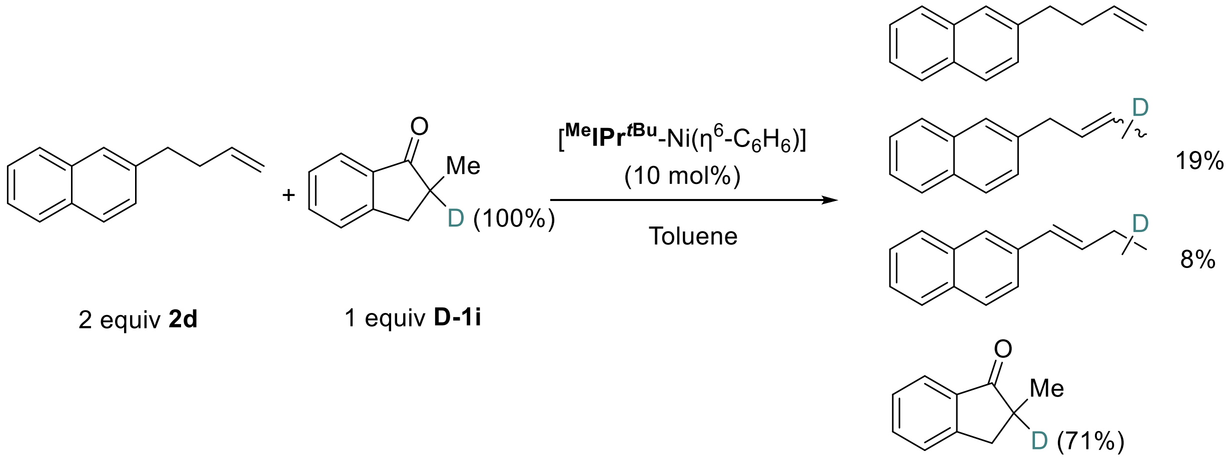

In a glovebox, an oven dried screw-capped 2 mL vial was charged with [(η6 – C_6H_6)Ni(^{Me}IPr^{tBu})] (10 μmol), degassed (freeze pump thaw) toluene (0.1 mL) was then added. ketone **D-1i** (0.1 mmol), and olefin **2d** (0.2 mmol degassed by freeze pump thaw) was added successively. The vial was sealed with a Teflon-lined screw cap, and running inside the glovebox at 40 °C. After 72 h, the vial was shipped outside of the glovebox. The reaction mixture was diluted with dichloromethane and filtered through a plug of silica gel. The crude solution was concentrated in vacuum and subjected to column chromatography 100:1 PE/EA to isolate the products, the isolated olefin isomers shown below matched the reported structure[24,25].

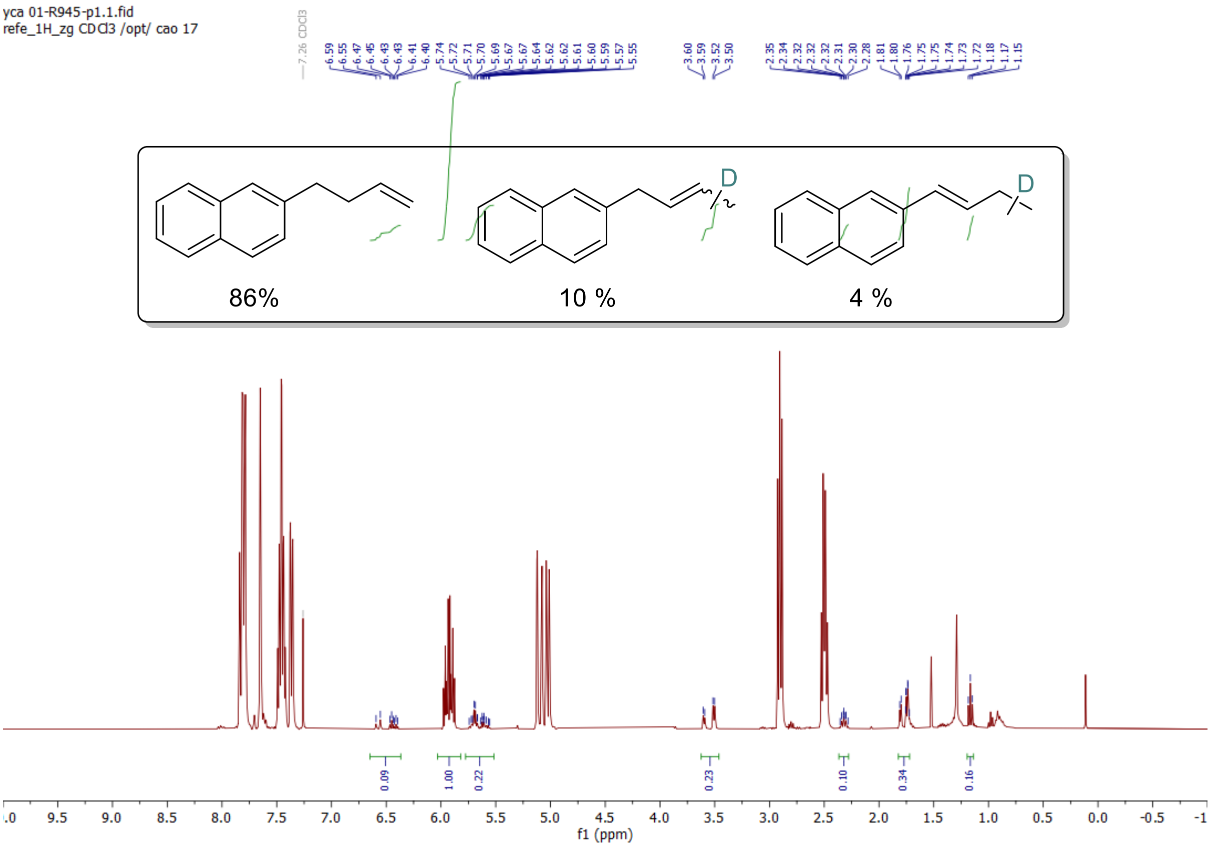

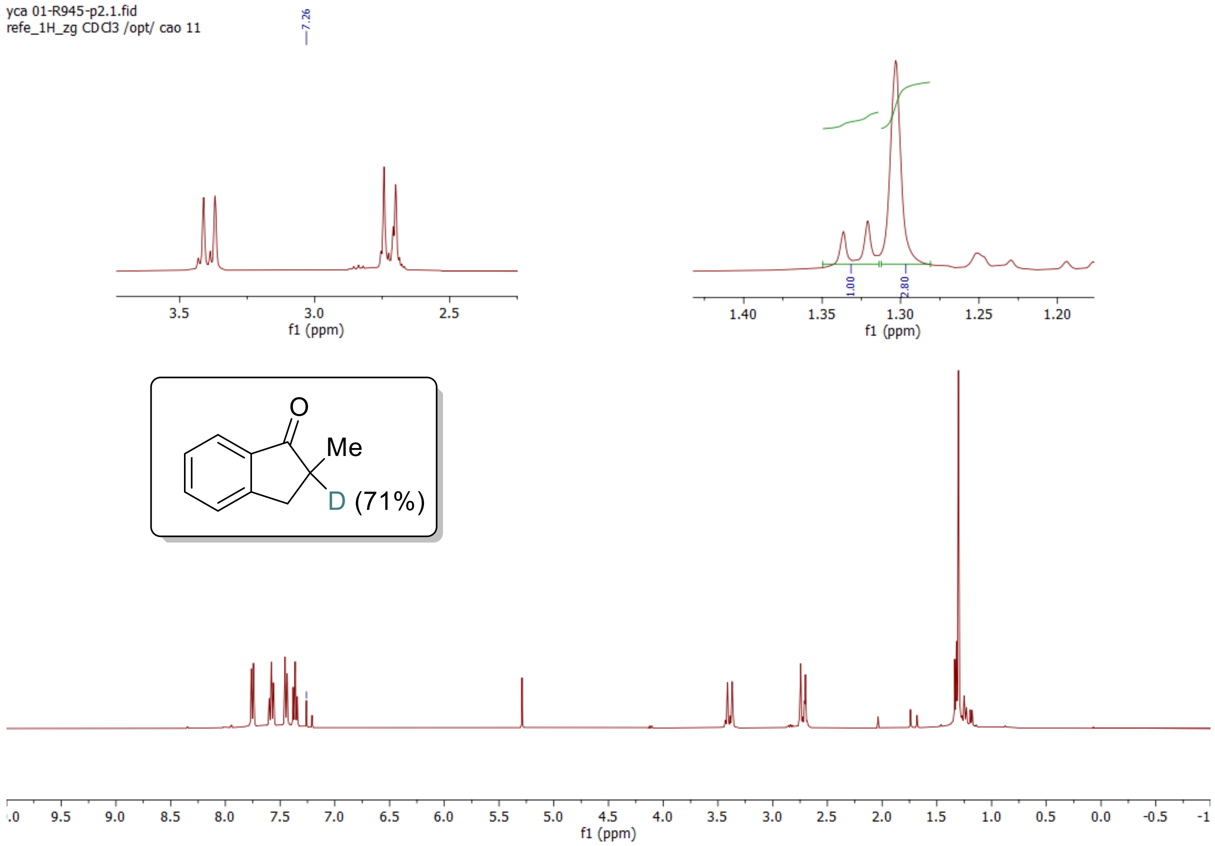

GC-MS

Theorical MS of deuterated targeted molecules.

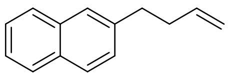

m/z: 182.1096 (100.0%), 183.1129 (15.1%), 184.1163 (1.1%)

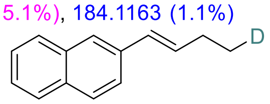

m/z: 183.1158 (100.0%), 184.1192 (15.1%), 185.1225 (1.1%)

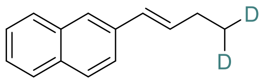

m/z: 184.1221 (100.0%), 185.1255 (15.1%), 186.1288 (1.1%)

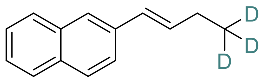

m/z: 185.1284 (100.0%), 186.1317 (15.1%), 187.1351 (1.1%)

Control experiment
GC-MS of starting material xx

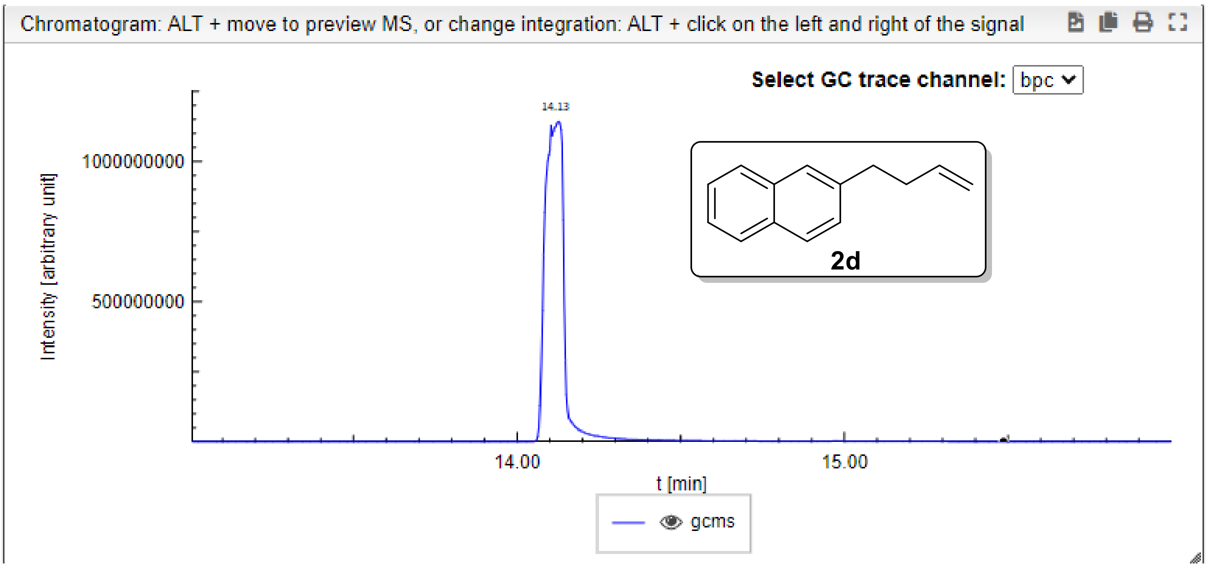

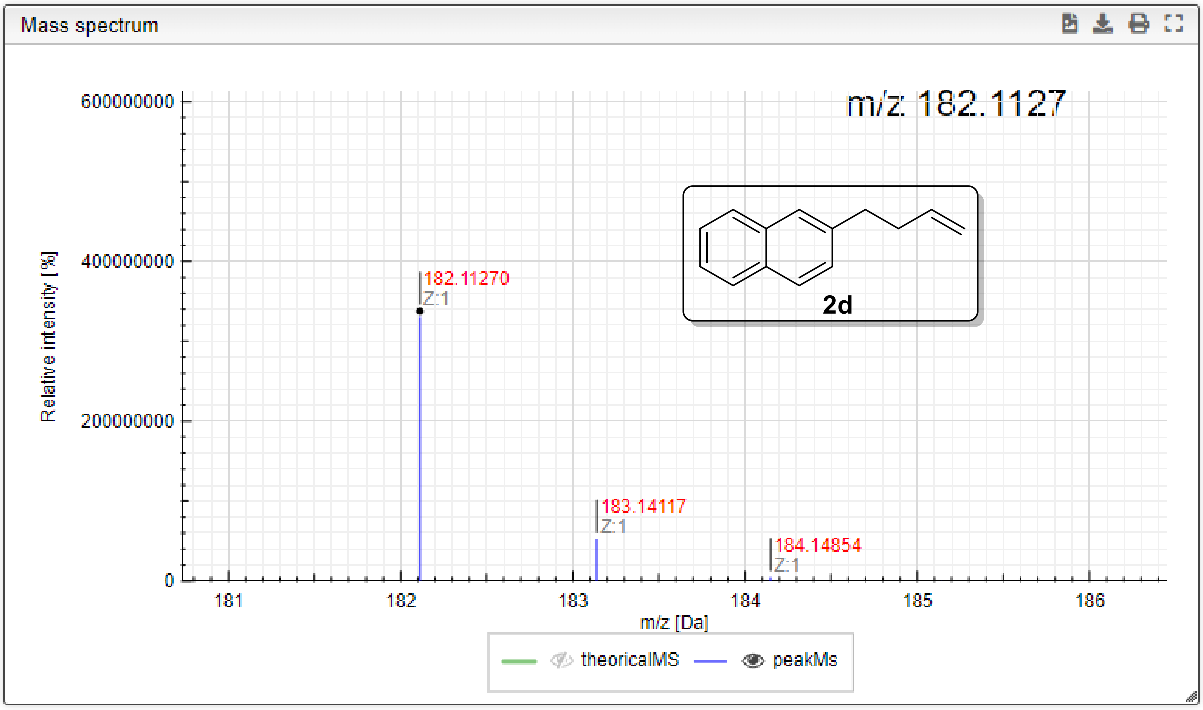

GC-MS spectrum of the target mixture.

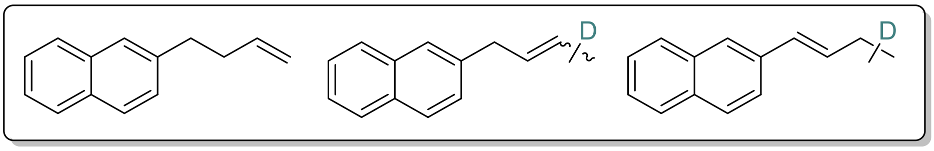

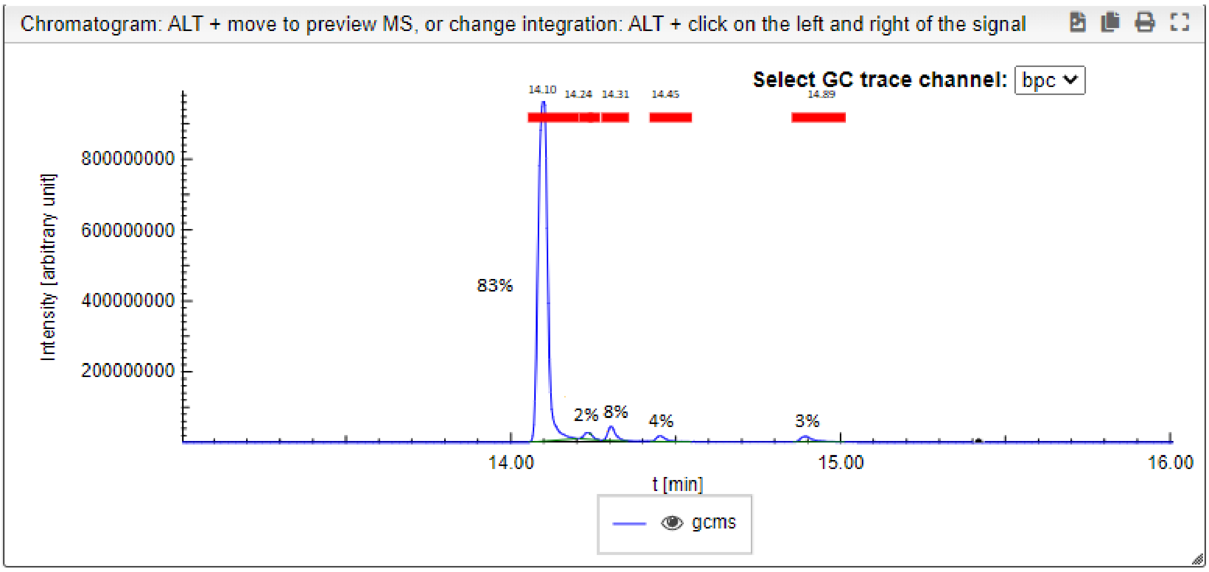

PEAK
LIST
220314_lcsa_cao_yca-01-R945_R-9451_571c0cdb87.raw
RT: 0.00 - 20.43
Number of detected peaks: 5

Apex RT	Start RT	End RT	Area	%Area	Height	%Height
14.10	14.06	14.20	4877477545	83	2794645992	40.03
14.24	14.20	14.27	111573541	2	67933541.4	0.97
14.31	14.27	14.42	440281647	8	220942568	3.16
14.45	14.42	14.55	216210291	4	97273934.7	1.39
14.89	14.86	15.00	200946017	3	75602135.1	1.08

MS spectrum of retention time **14.10 min**

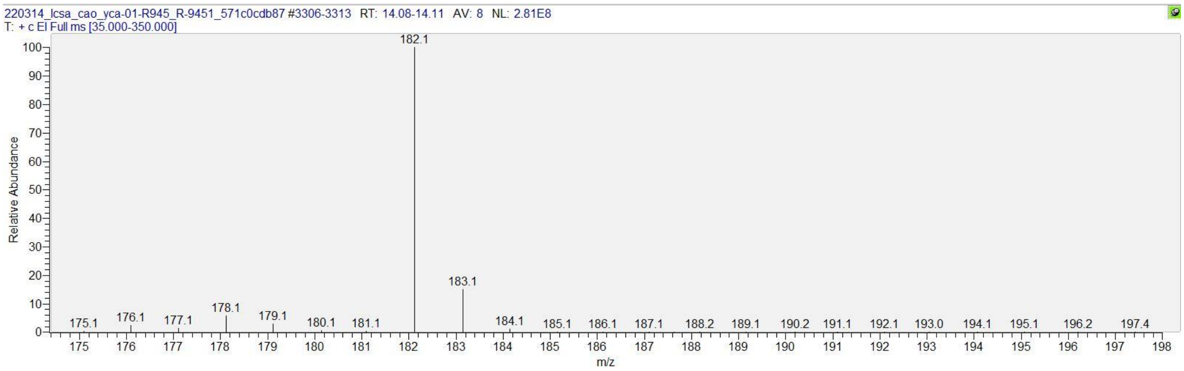

MS spectrum of retention time **14.24 min.**

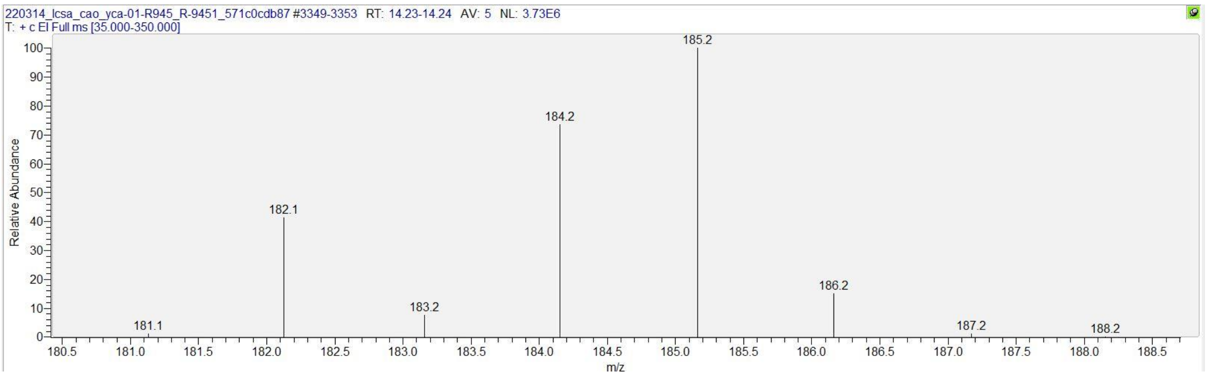

MS spectrum of retention time **14.31 min**, **14.45 min**, **14.89 min.**

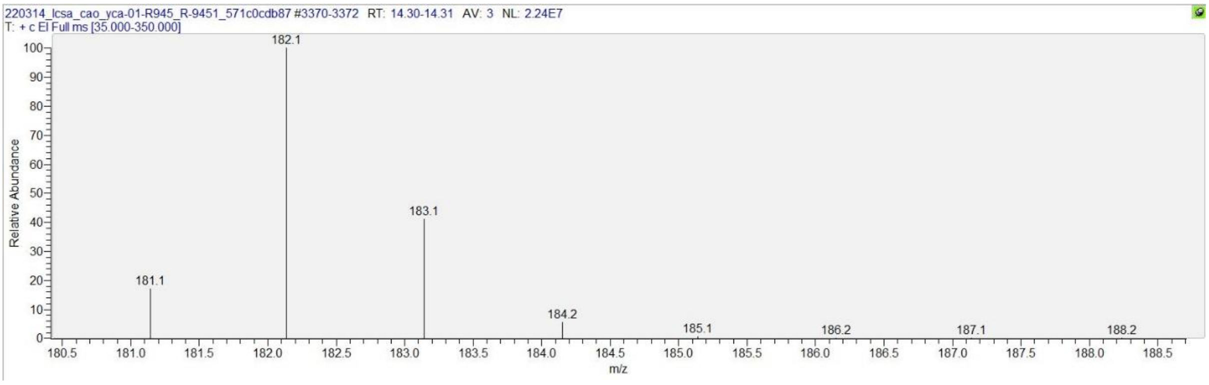

5.10 *Kinetic isotope effect (KIE).*

In a glovebox, an oven dried screw-capped 2 mL vial was charged with a magnetic stir bar, $Ni(COD)_2$ (10 μmol), carbene ligand $^{Me}IPr^{tBu}$ (10 μmol), degassed (freeze pump thaw) THF (0.1 mL) was then added and the catalyst mixture was stirred at room temperature for 30 min, during which a dark solution is formed. Deuterated ketone **D-1i** (0.1 mmol degassed freeze pump thaw) and COD **2a** (0.15 mmol degassed by freeze pump thaw) was then added successively. The vial was sealed with a Teflon-lined screw cap, and running inside the glovebox at room temperature. After 1, 6, 12, 16, 20, 24 h, the vial was shipped outside of the glovebox. The reaction mixture was diluted with dichloromethane and filtered through a plug of silica gel. The crude solution was concentrated in vacuum and subjected to column chromatography 100:1 PE/EA to isolate the products

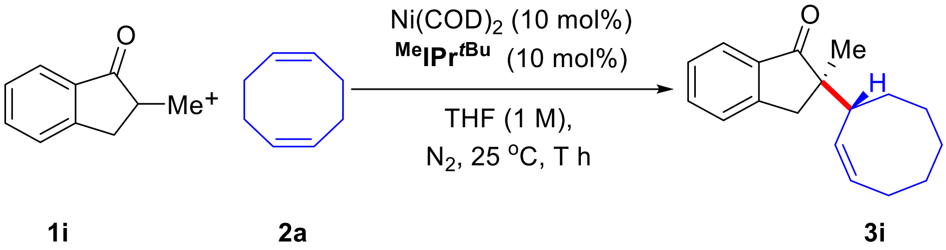

entry	T(hours)	yield	dr
1	1	10%	6.4:1
2	6	29%	6.4:1
3	12	36%	6.4:1
4	16	41%	6.4:1
5	20	46%	6.4:1
6	24	51%	6.4:1

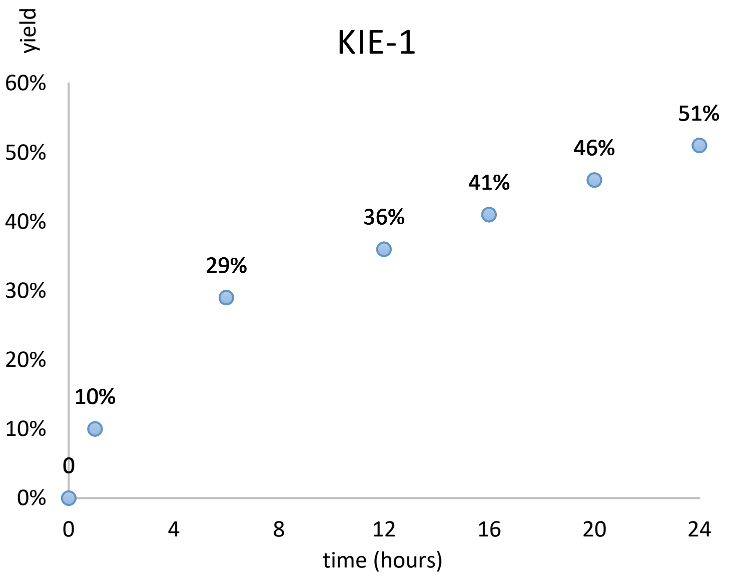

Supplementary Fig 5.8 | Plot of the yield of **3i** vs reaction time under standard conditions.

In a glovebox, an oven dried screw-capped 2 mL vial was charged with a magnetic stir bar, $Ni(COD)_2$ (10 μmol), carbene ligand $^{Me}IPr^{tBu}$ (10 μmol), degassed (freeze pump thaw) THF (0.1 mL) was then added and the catalyst mixture was stirred at room temperature for 30 min, during which a red black solution is formed. Deuterated ketone **D-1i** (0.1 mmol degassed freeze pump thaw) and COD **2a** (0.15 mmol degassed by freeze pump thaw) was then added successively. The vial was sealed with a Teflon-lined screw cap, and running inside the glovebox at room temperature. After 1, 6, 12, 16, 20, 24 h, the vial was shipped outside of the glovebox. The reaction mixture was diluted with dichloromethane and filtered through a plug of silica gel. The crude solution was concentrated in vacuum and subjected to column chromatography 100:1 PE/EA to isolate the products

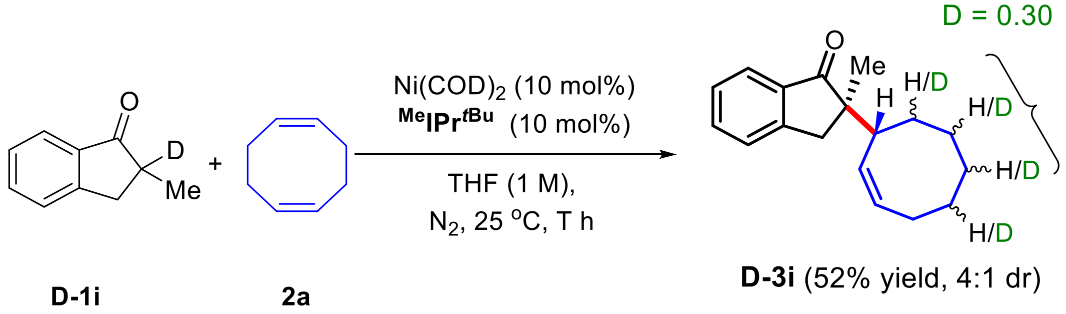

entry	T(hours)	yield	dr
1	1	11%	4.0:1
2	6	27%	4.0:1
3	12	35%	4.0:1
4	16	39%	4.0:1
5	20	46%	4.0:1
6	24	50%	4.0:1

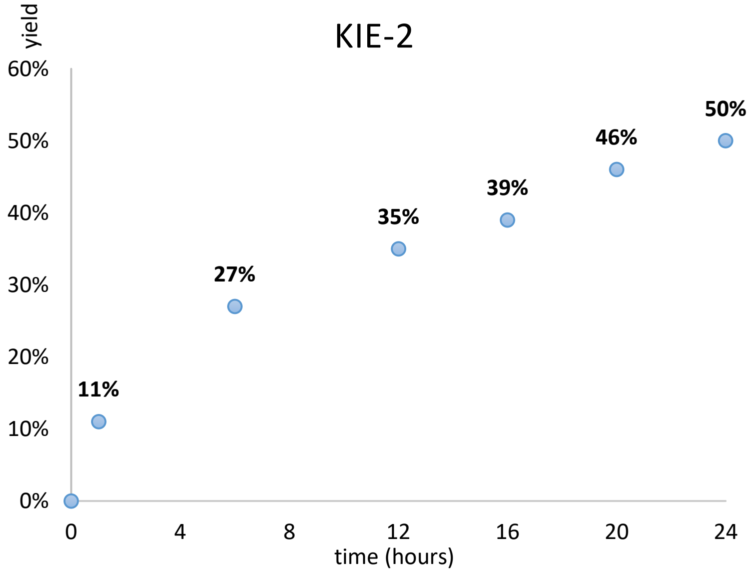

Supplementary Fig 5.9 | Plot of the yield of **D-3i** vs reaction time under standard conditions.

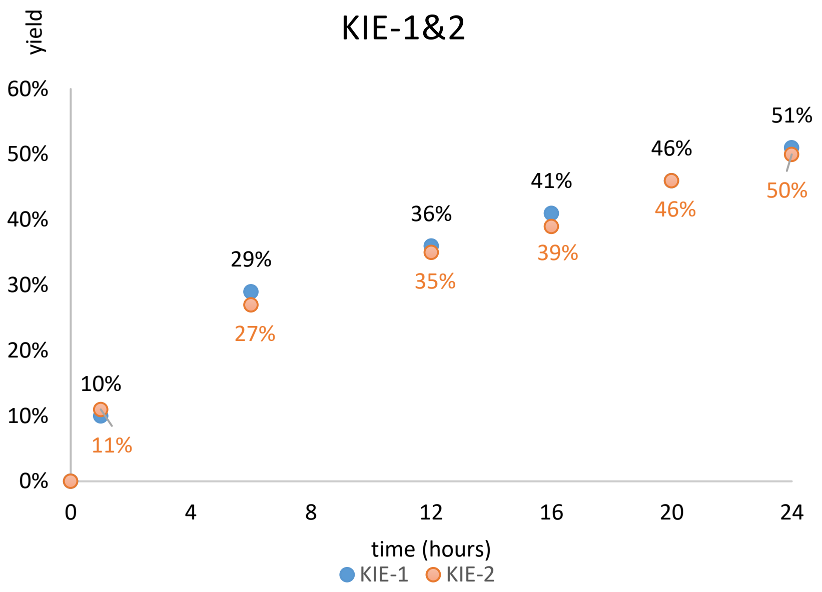

Supplementary Fig 5.10 | Plot of the yield of **3i** and **D-3i** vs reaction time under standard conditions.

The KIE value was calculated by dividing initial reaction rate of **3i** and **D-3i**. (0.10/0.11 = 0.91).

5.11 *Kinetic studies.*

In a glovebox, an oven dried screw-capped 2 mL vial was charged with a magnetic stir bar, $Ni(COD)_2$ (10 μmol), carbene ligand $^{Me}IPr^{tBu}$ (10 μmol), degassed (freeze pump thaw) THF (0.1 mL) was then added and the catalyst mixture was stirred at room temperature for 30 min, during which a dark solution is formed. Deuterated ketone **D-1i** (0.1 mmol degassed freeze pump thaw) and COD **2a** (0.15 mmol degassed by freeze pump thaw) was then added successively. The vial was sealed with a Teflon-lined screw cap, and running inside the glovebox at room temperature. After 1, 6, 12, 16, 20, 24 h, the vial was shipped outside of the glovebox. The reaction mixture was diluted with dichloromethane and filtered through a plug of silica gel. The crude solution was concentrated in vacuum and subjected to column chromatography 100:1 PE/EA to isolate the products.

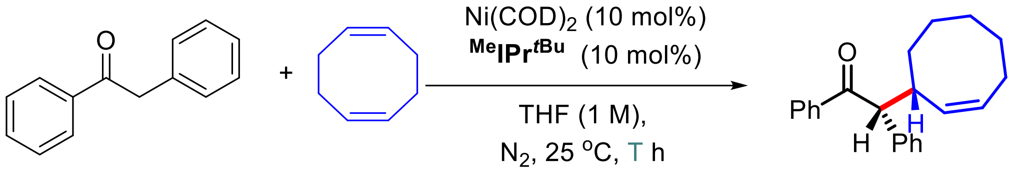

1a **2a** **3a**

entry	T(hours)	yield	dr
1	1	10%	8.0:1
2	6	25%	7.2:1
3	12	51%	6.5:1
4	16	64%	6.2:1
5	20	75%	5.3:1
6	24	85%	4.2:1

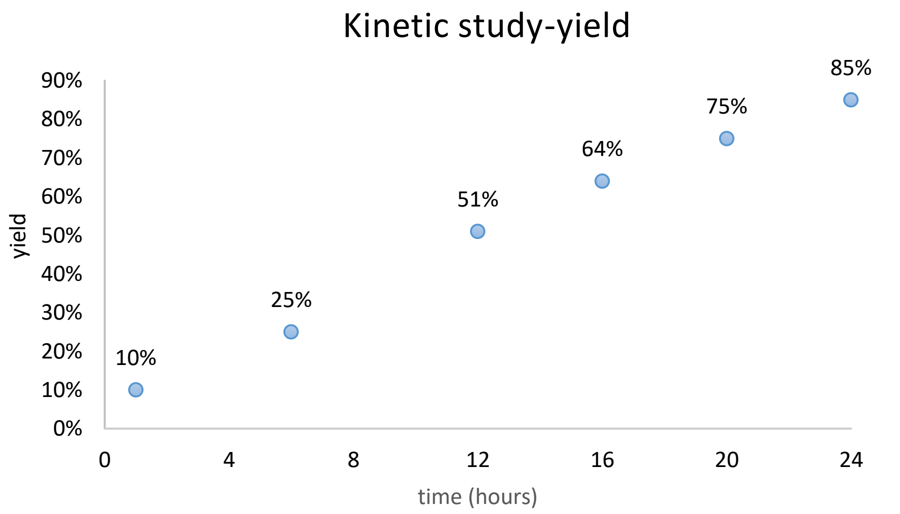

Supplementary Fig 5.11 | Plot of the yield of **3a** vs reaction time under standard conditions.

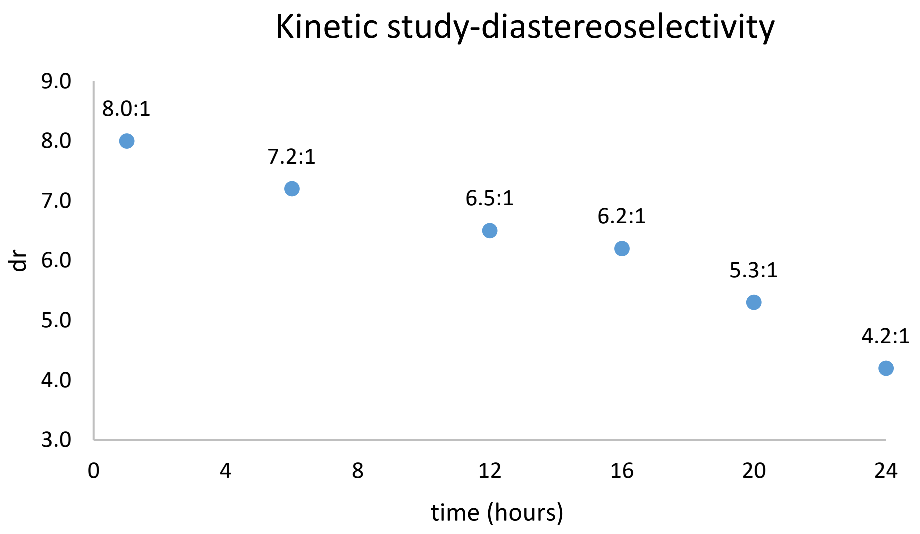

Supplementary Fig 5.12 | Plot of the diastereoselectivity of **3a** vs reaction time under standard conditions.

6. Computational studies.

Computational Details.

As numerous conformers of these catalysts exist and the obtaining an accurate picture of energetics is critical to understanding and reproducing selectivity, we employed a computational pipeline[28,29] using the graph-based Molassembler library that involved determining the energies of the presumed rate-determining transition state [TS(1,2)]. Here, approximately 30 structures of TS1,2 (for both the major and minor pathways) were analyzed using the computational protocol discussed below. The lowest energy transition state for both species was then used as a starting point and the complete catalytic cycle propagated from this structure to determine the relative free energies seen in Scheme X.

The geometries of all species were optimized in the gas phase using the ωB97X-D[30] functional along with the def-SVP (H,C,O,N) and def2-TZVP (Ni) basis sets[31] for Ni using Gaussian16.[32] This combination, henceforth referred to as ωB97X-D/BS1, was chosen to assure accurate energetics and geometries of bond formation/breaking process occurring at the Ni center. Single point energies using the SMD solvation model[33] for THF were determined at the ωB97X-D/def2-TZVP level on the optimized ωB97X-D/BS1 geometries. Species were characterized as either minima (zero imaginary frequencies) or transition states (one imaginary frequency) by examining vibrational frequencies on the optimized structures. Free energy corrections were determined using the quasi rigid-rotor harmonic oscillator model[34] and a pressure correction (13.1 mol/L for THF) to treat the translational entropy in solution following the approach of Martin, Hay, and Pratt[35] using Goodvibes.[36,37] Final free energies for each species/conformer were obtained by summing the ωB97X-D/def2-TZVP electronic energies and the enthalpy/entropy corrections from the ωB97X-D/BS1 frequency analysis (see SI for a breakdown on the free energy of each species by component).

Supplementary Table 6.1 | Relative free energies (relative to the separated catalyst and ketone) of catalytic cycle species computed at the ωB97X-D/def2-TZVP/ ωB97X-D/BS1 level level (see Computational Details in main text). Values in kcal/mol.

Species	Major Pathway	Minor Pathway
Int0	0.24	-0.84
Int1	0.71	4.60
TS(1,2)	23.60	29.26
Int2	1.61	2.43
Int3	0.02	3.33
TS(3,4)	21.04	18.60
Int4	20.74	16.96
TS(4,5)	20.89	18.68
Int5	2.57	3.58
Int6	0.05	6.61
TS(6,7)	20.02	24.51
Int7	17.65	22.38
TS(7,8)	21.44	22.67
Int8	1.99	4.19
Int9	-13.21	-12.16
TS(9,10)	9.27	9.27
Int10	-13.26	1.45
Product	-4.88	-7.39

Supplementary Table 6.2 | Computed energies of relevant compounds. Values in Hartree.

Reference	ωB97X-D/BS1 Electronic Energy	ωB97X-D/BS1 Free Energy Correction	ωB97X-D/def2-TZVP Single Point Energy	ωB97X-D Total Free Energy
Ketone	-461.843037	0.146344	-462.350576	-462.204232
Cyclooctadiene	-311.715653	0.156199	-312.052087	-311.895888
Catalyst	-3371.833996	0.961014	-3373.798117	-3372.837103
**Major Product Pathway**				
Major-Int0	-3833.711708	1.129848	-3836.170800	-3835.040952
Major-Int1	-3833.706484	1.127672	-3836.167871	-3835.040199
Major-TS(1,2)	-3833.701954	1.121387	-3836.125119	-3835.003732
Major-Int2	-3833.706415	1.128698	-3836.167462	-3835.038764
Major-Int3	-3833.704736	1.127458	-3836.168763	-3835.041305
Major-TS(3,4)	-3833.665911	1.123316	-3836.131123	-3835.007807
Major-Int4	-3833.671022	1.125140	-3836.133431	-3835.008291
Major-TS(4,5)	-3833.670479	1.124789	-3836.132841	-3835.008052
Major-Int5	-3833.704807	1.125468	-3836.162705	-3835.037237
Major-Int6	-3833.708873	1.127329	-3836.168585	-3835.041256

Major-TS(6,7)	-3833.675431	1.125011	-3836.134443	-3835.009432
Major-Int7	-3833.680068	1.125747	-3836.138958	-3835.013211
Major-TS(7,8)	-3833.675639	1.126209	-3836.133383	-3835.007174
Major-Int8	-3833.706955	1.127365	-3836.165536	-3835.038171
Major-Int9	-3833.735778	1.130906	-3836.193296	-3835.062390
Major-TS(9,10)	-3833.703992	1.130812	-3836.157378	-3835.026566
Major-Int10	-3833.741973	1.133292	-3836.195755	-3835.062463
Major-Product	-773.600616	0.324065	-774.431957	-774.107892
**Minor Product Pathway**				
Minor-Int0	-3833.713367	1.129760	-3836.172434	-3833.713367
Minor-Int1	-3833.700161	1.126877	-3836.160887	-3833.700161
Minor-TS(1,2)	-3833.660433	1.122773	-3836.117473	-3833.660433
Minor-Int2	-3833.705188	1.129082	-3836.166540	-3833.705188
Minor-Int3	-3833.702553	1.128744	-3836.164776	-3833.702553
Minor-TS(3,4)	-3833.673370	1.124040	-3836.135740	-3833.673370
Minor-Int4	-3833.673968	1.122607	-3836.136907	-3833.673968
Minor-TS(4,5)	-3833.673461	1.124405	-3836.135980	-3833.673461
Minor-Int5	-3833.706123	1.128733	-3836.164362	-3833.706123
Minor-Int6	-3833.696945	1.127034	-3836.157831	-3833.696945
Minor-TS(6,7)	-3833.661930	1.123089	-3836.125370	-3833.661930
Minor-Int7	-3833.667499	1.124543	-3836.130218	-3833.667499
Minor-TS(7,8)	-3833.667137	1.125084	-3836.130286	-3833.667137
Minor-Int8	-3833.703766	1.128518	-3836.163172	-3833.703766
Minor-Int9	-3833.736615	1.130709	-3836.191420	-3833.736615
Minor-TS(9,10)	-3833.703992	1.130812	-3836.157378	-3833.703992
Minor-Int10	-3833.723673	1.137694	-3836.176724	-3833.723673
Minor-Product	-773.604051	0.323204	-774.435100	-773.604051

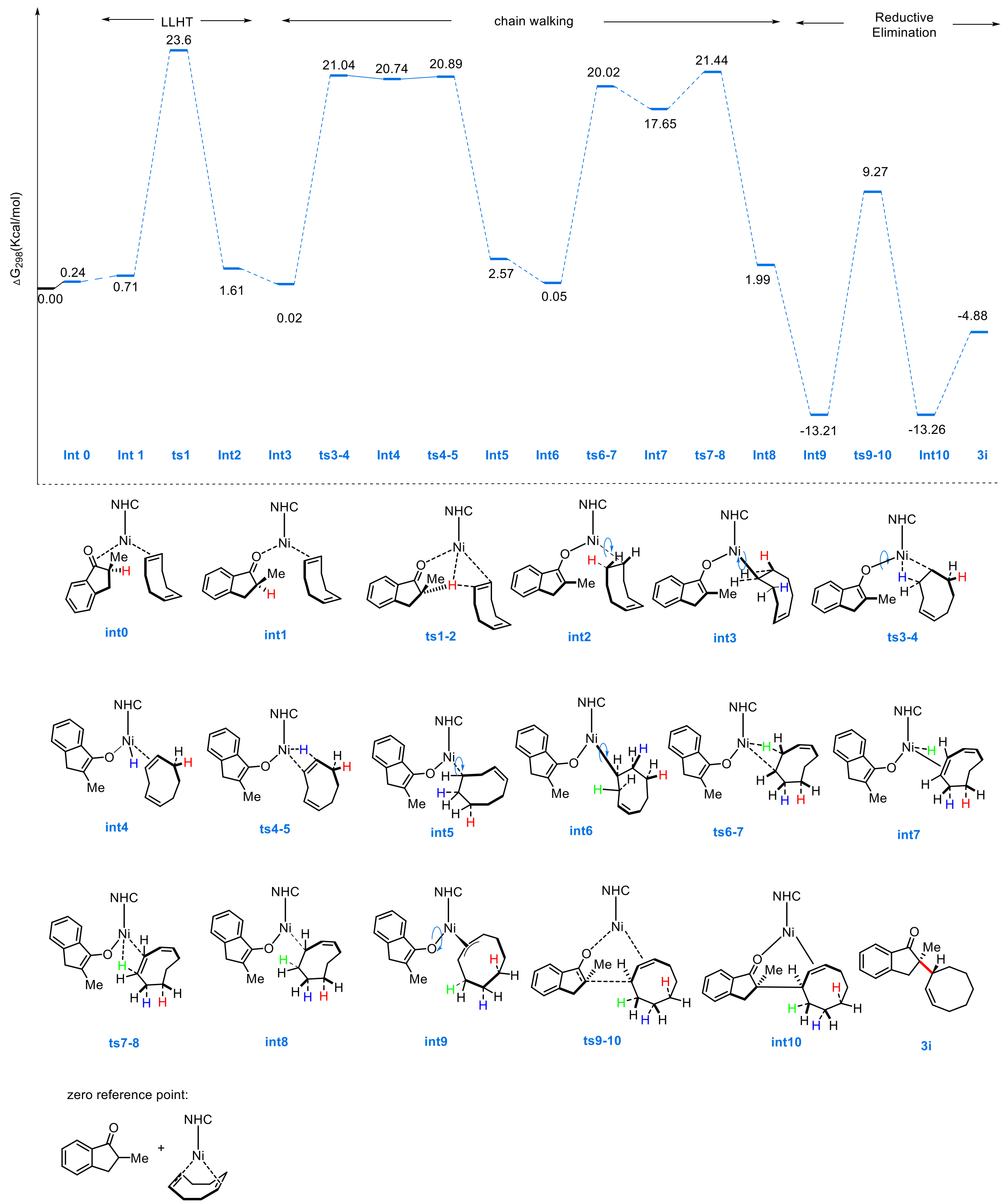

Supplementary Fig 6.1 | Computed energetics for the hydro-alkylation of diene **1a** with ketone **3i**. (formation of major diastereoisomer **3i**).

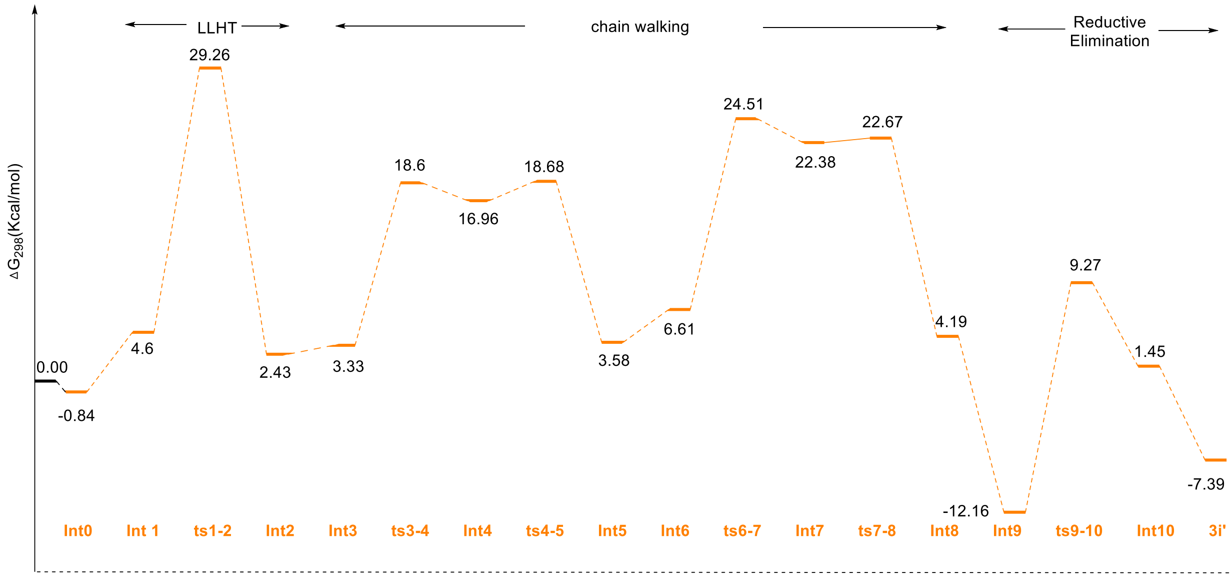

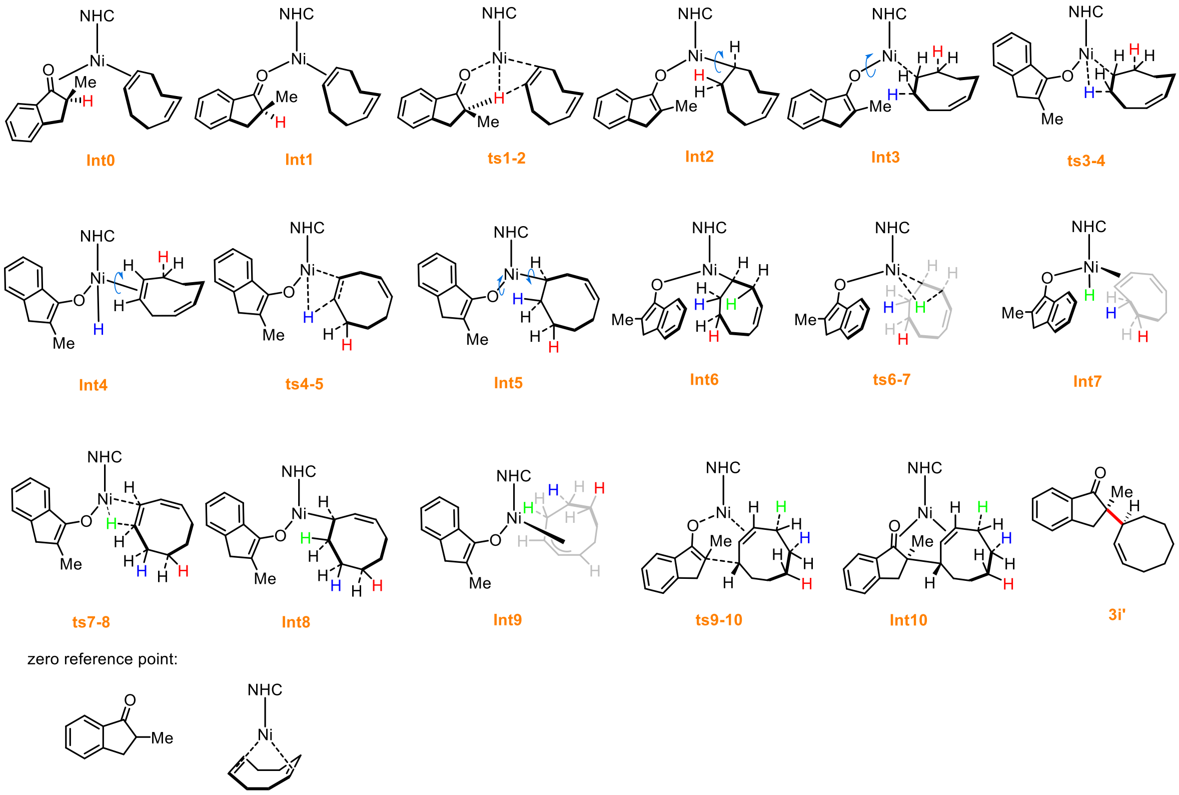

Supplementary Fig 6.2 | Computed energetics for the hydro-alkylation of diene **1a** with ketone **3i**. (formation of minor diastereoisomer **3i'**).

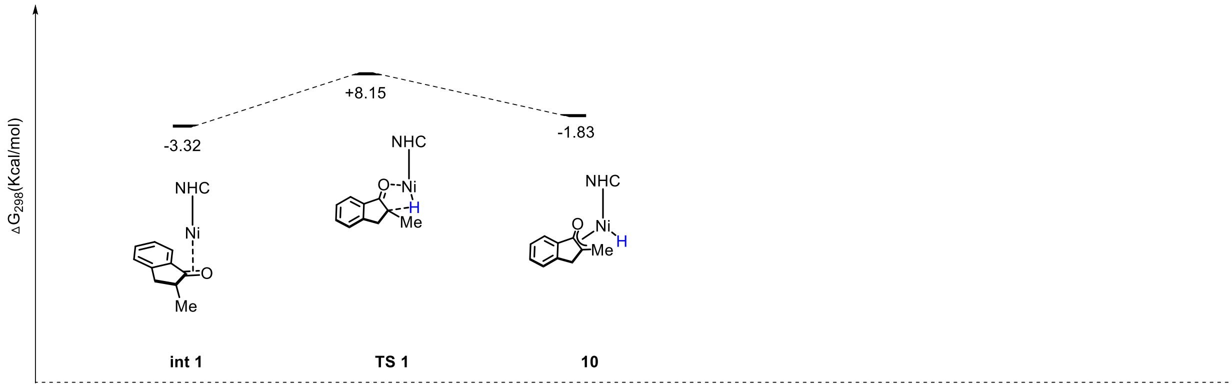

Supplementary Fig 6.3 | Computed energetics for complex **10**.

7. NMR spectra.

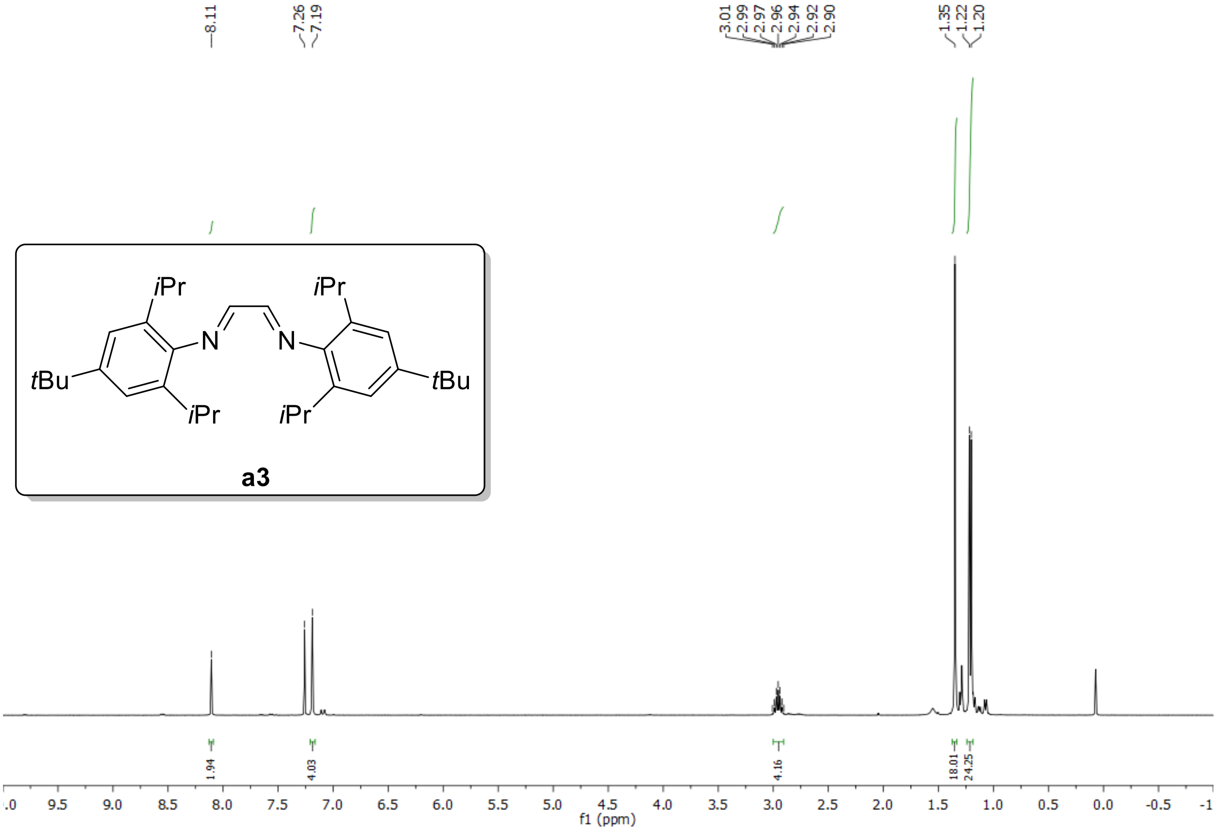

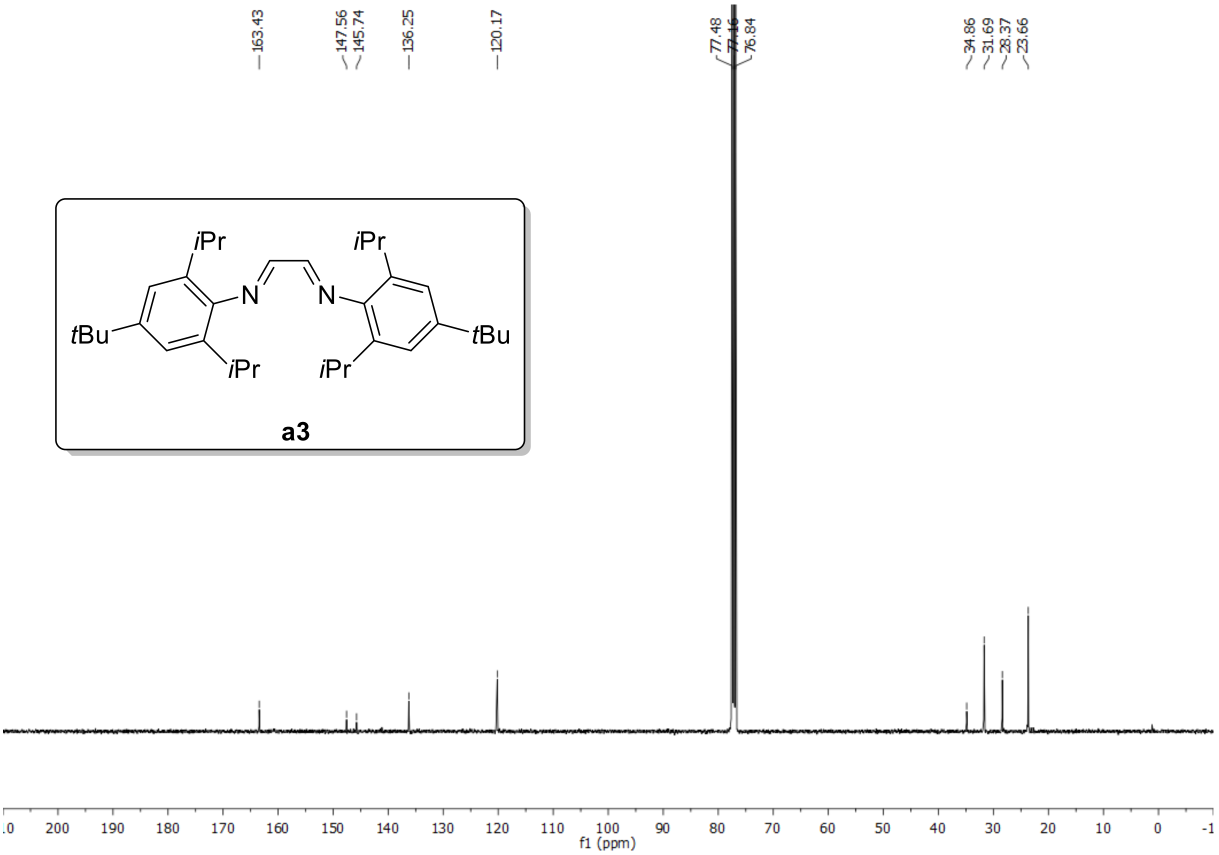

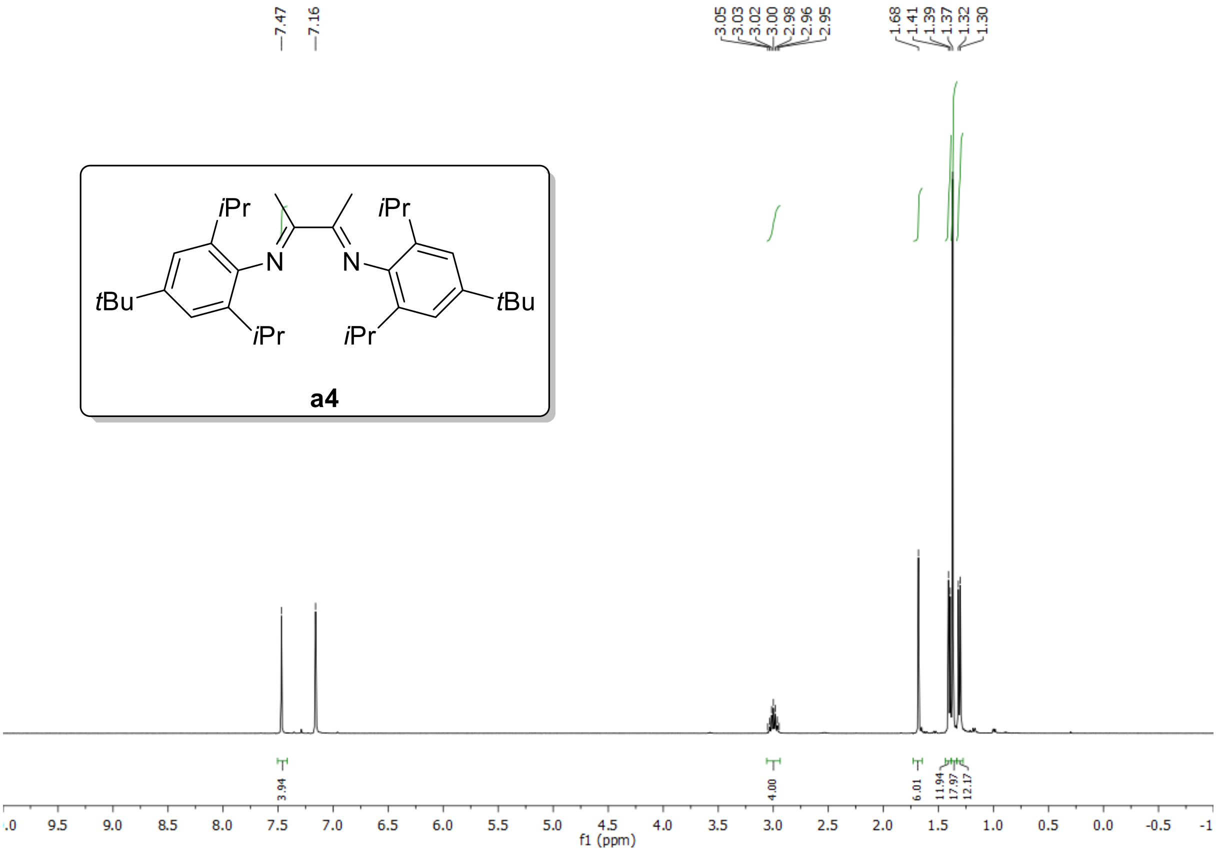
7.47
7.16
3.05
3.03
3.02
3.00
2.98
2.96
2.95
1.68
1.41
1.39
1.37
1.32
1.30
iPr
iPr
N
N
tBu
tBu
iPr
iPr
a4
3.94
4.00
6.01
11.94
17.97
12.17
f1 (ppm)

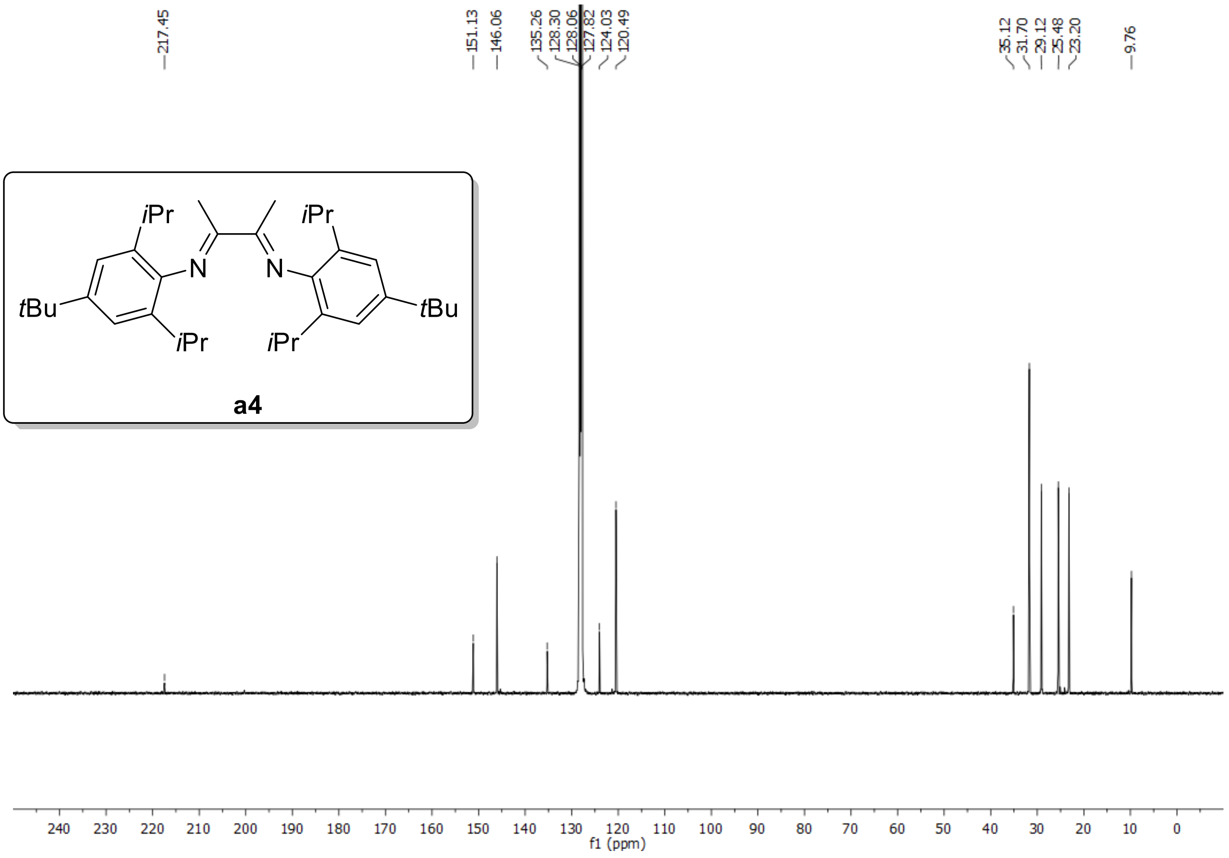
217.45
151.13
146.06
135.26
128.30
128.06
127.82
124.03
120.49
35.12
31.70
29.12
25.48
23.20
9.76
iPr
iPr
N
N
tBu
tBu
iPr
iPr
a4
f1 (ppm)

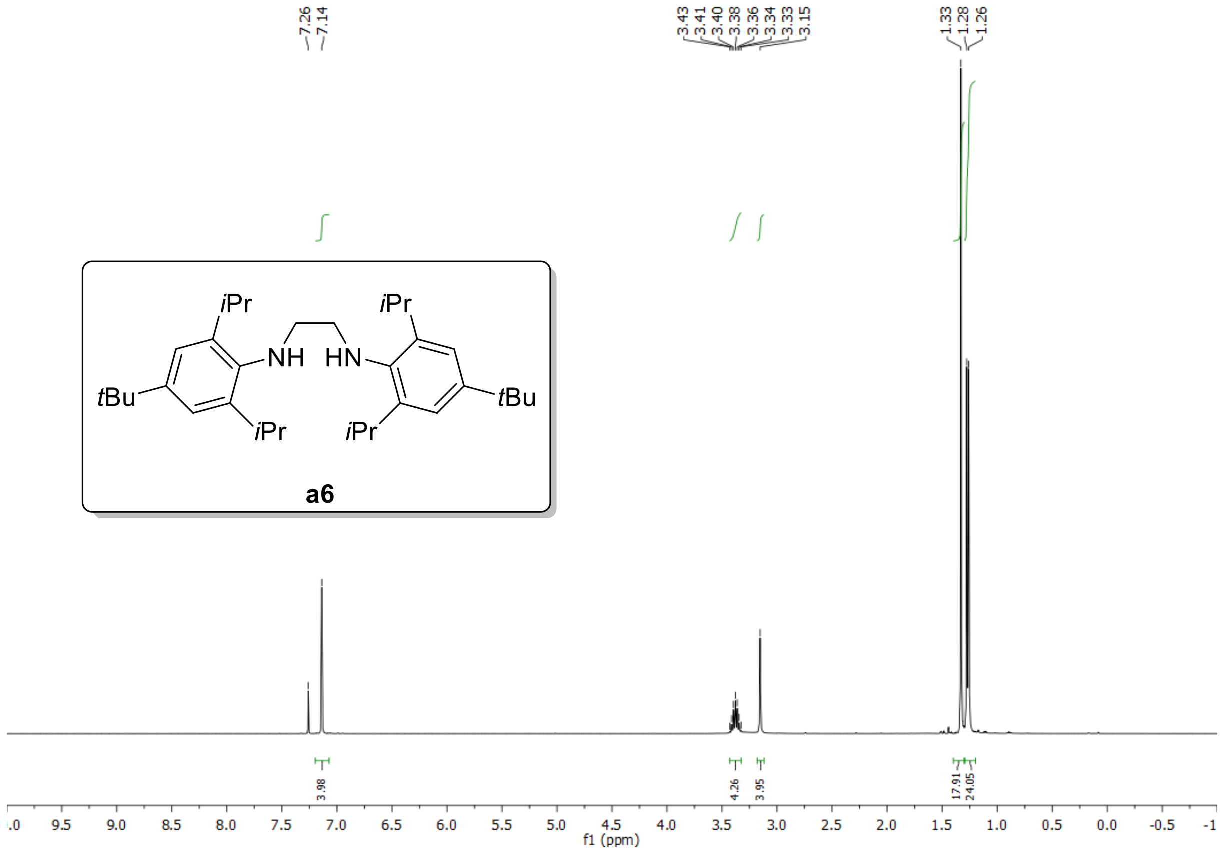
7.26
7.14
3.43
3.41
3.40
3.38
3.36
3.34
3.33
3.15
1.33
1.28
1.26
iPr
iPr
NH
HN
tBu
tBu
iPr
iPr
a6
3.98
4.26
3.95
17.91
24.05
f1 (ppm)

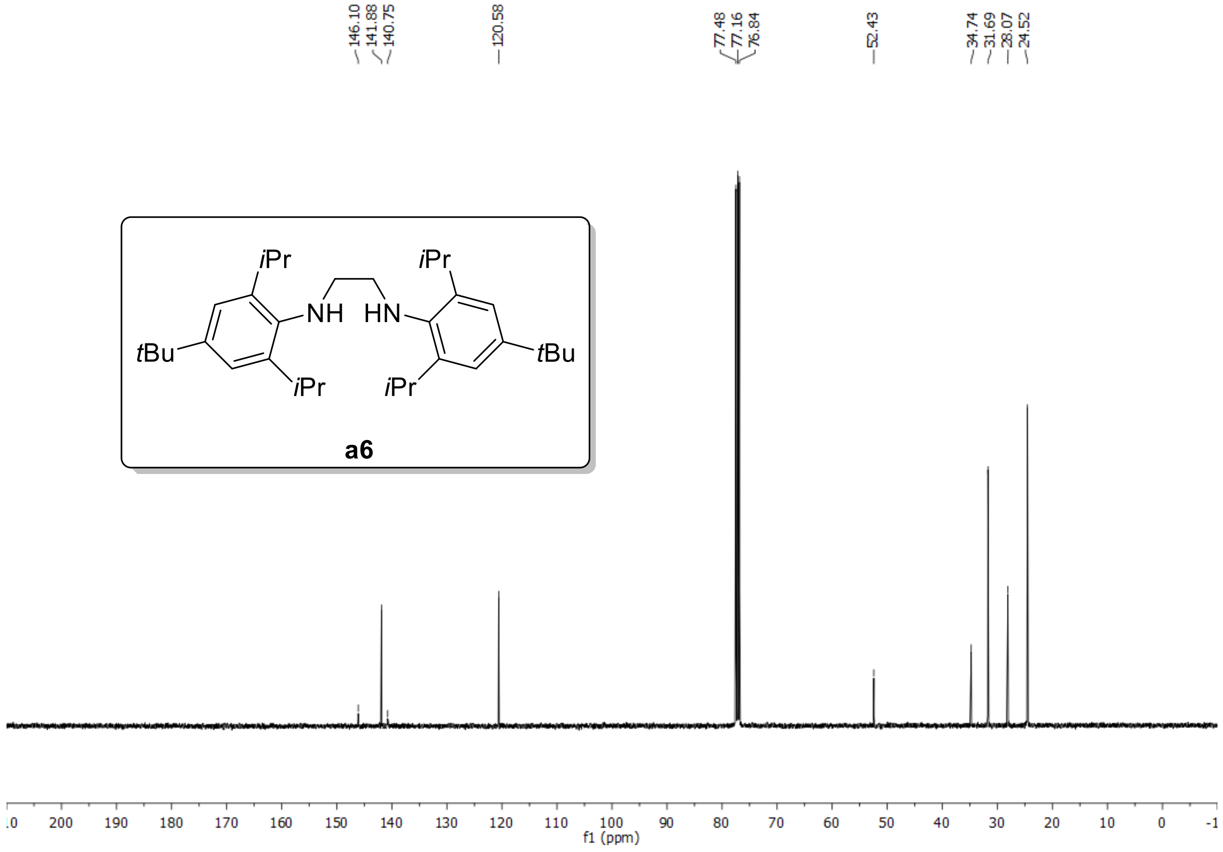
146.10
141.88
140.75
120.58
77.48
77.16
76.84
52.43
34.74
31.69
28.07
24.52
iPr
iPr
NH
HN
tBu
tBu
iPr
iPr
a6
f1 (ppm)

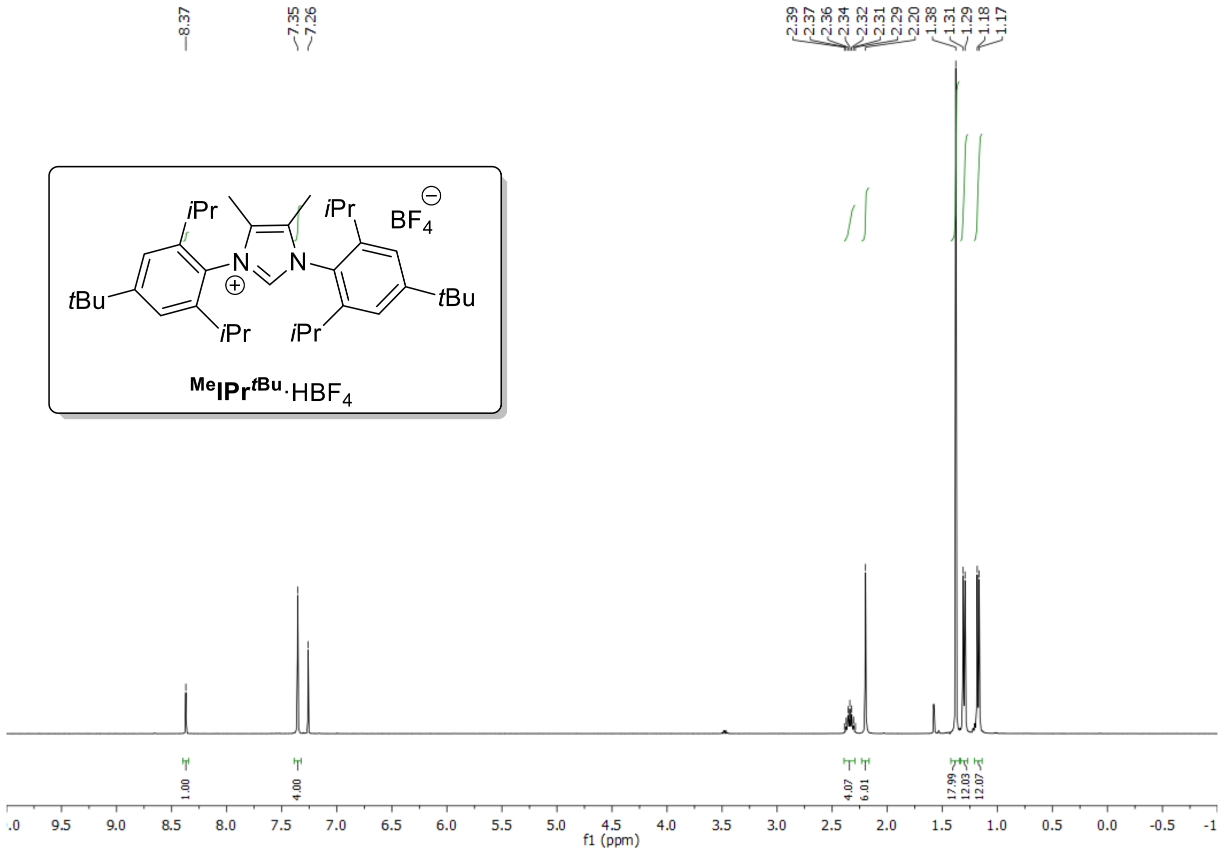
8.37
7.35
7.26
2.39
2.37
2.36
2.34
2.32
2.31
2.29
2.20
1.38
1.31
1.29
1.18
1.17
iPr
iPr
BF4
N
N
tBu
tBu
iPr
iPr
MeIPrtBu·HBF4
1.00
4.00
4.07
6.01
17.99
12.03
12.07
f1 (ppm)

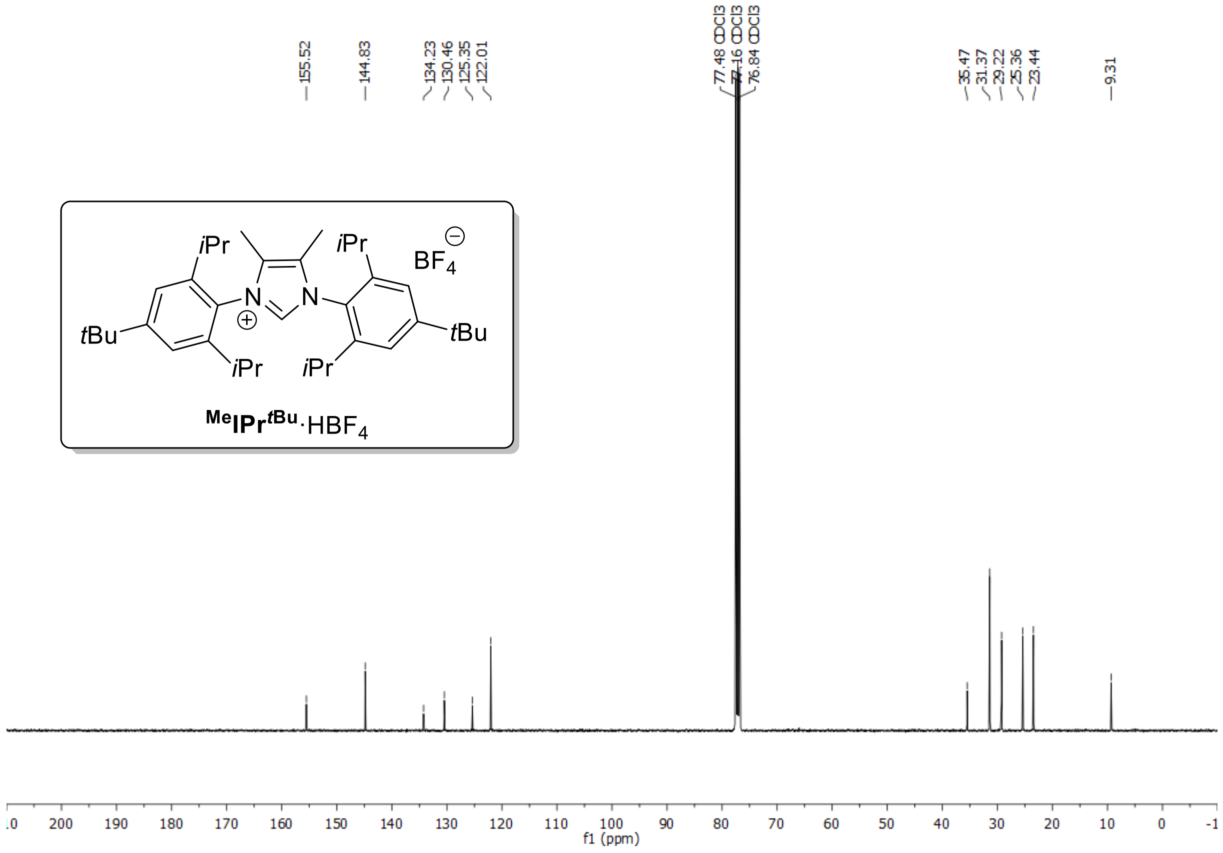
155.52
144.83
134.23
130.46
125.35
122.01
77.48 CDCl3
77.16 CDCl3
76.84 CDCl3
35.47
31.37
29.22
25.36
23.44
9.31
iPr
iPr
BF4
N
N
tBu
tBu
iPr
iPr
MeIPrtBu·HBF4
f1 (ppm)

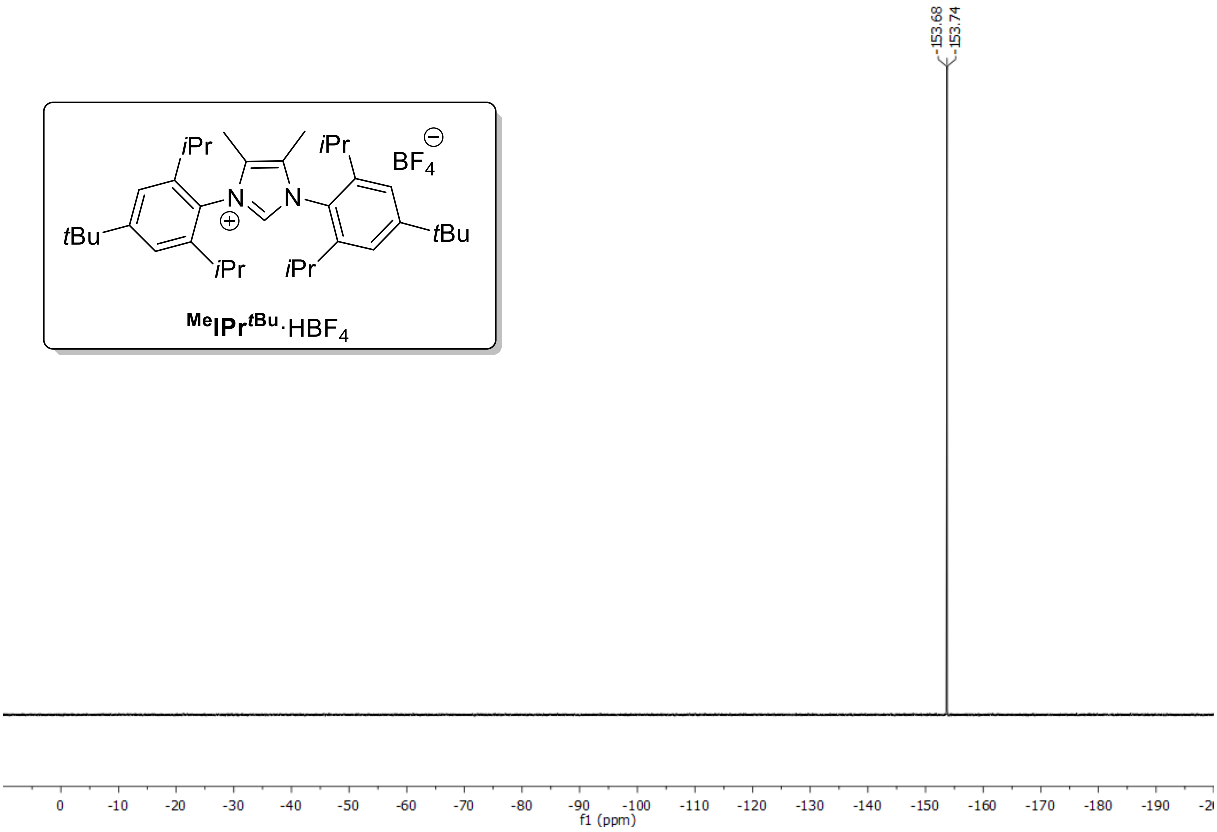
-153.68
-153.74
iPr
iPr
BF4
tBu
tBu
iPr
iPr
MeIPrtBu·HBF4
f1 (ppm)

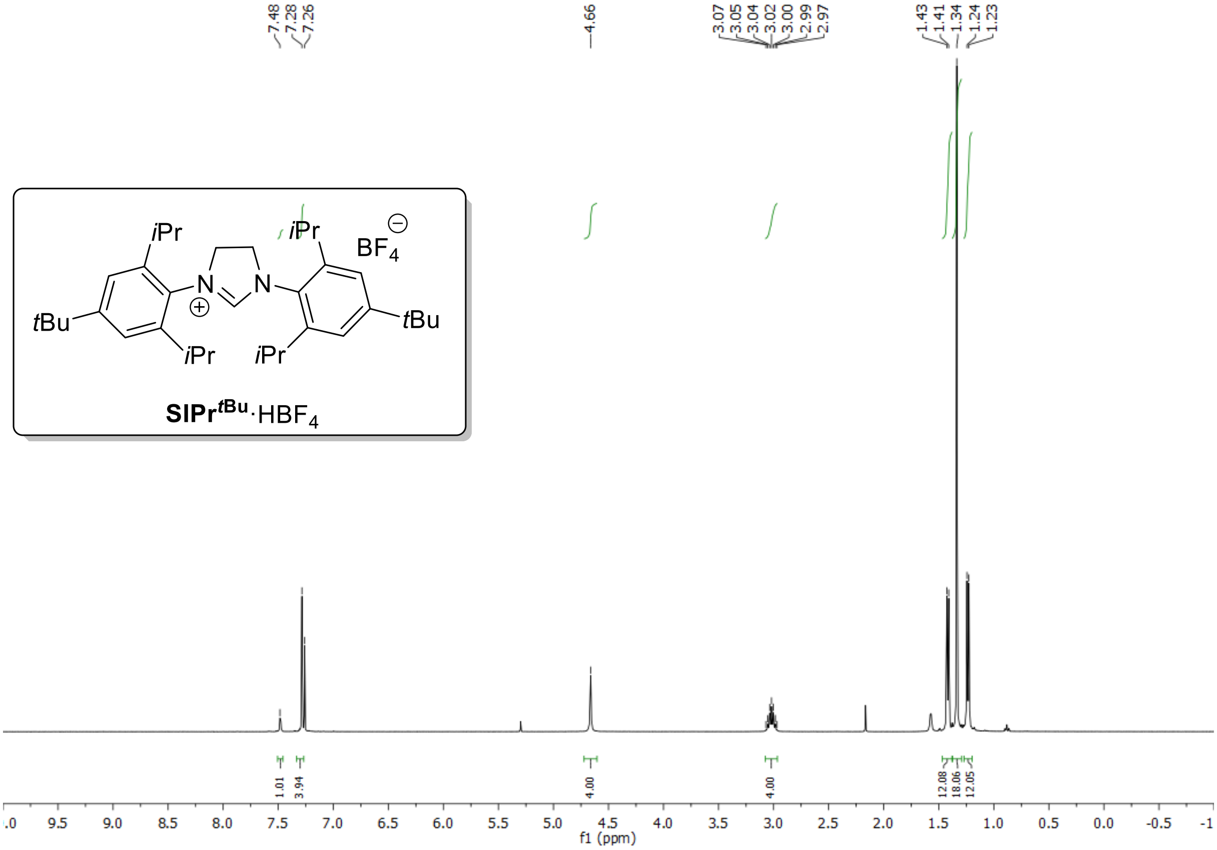
7.48
7.28
7.26
4.66
3.07
3.05
3.04
3.02
3.00
2.99
2.97
1.43
1.41
1.34
1.24
1.23
iPr
iPr
BF4
tBu
tBu
iPr
iPr
SIPrtBu·HBF4
1.01
3.94
4.00
4.00
12.08
18.06
12.05
f1 (ppm)

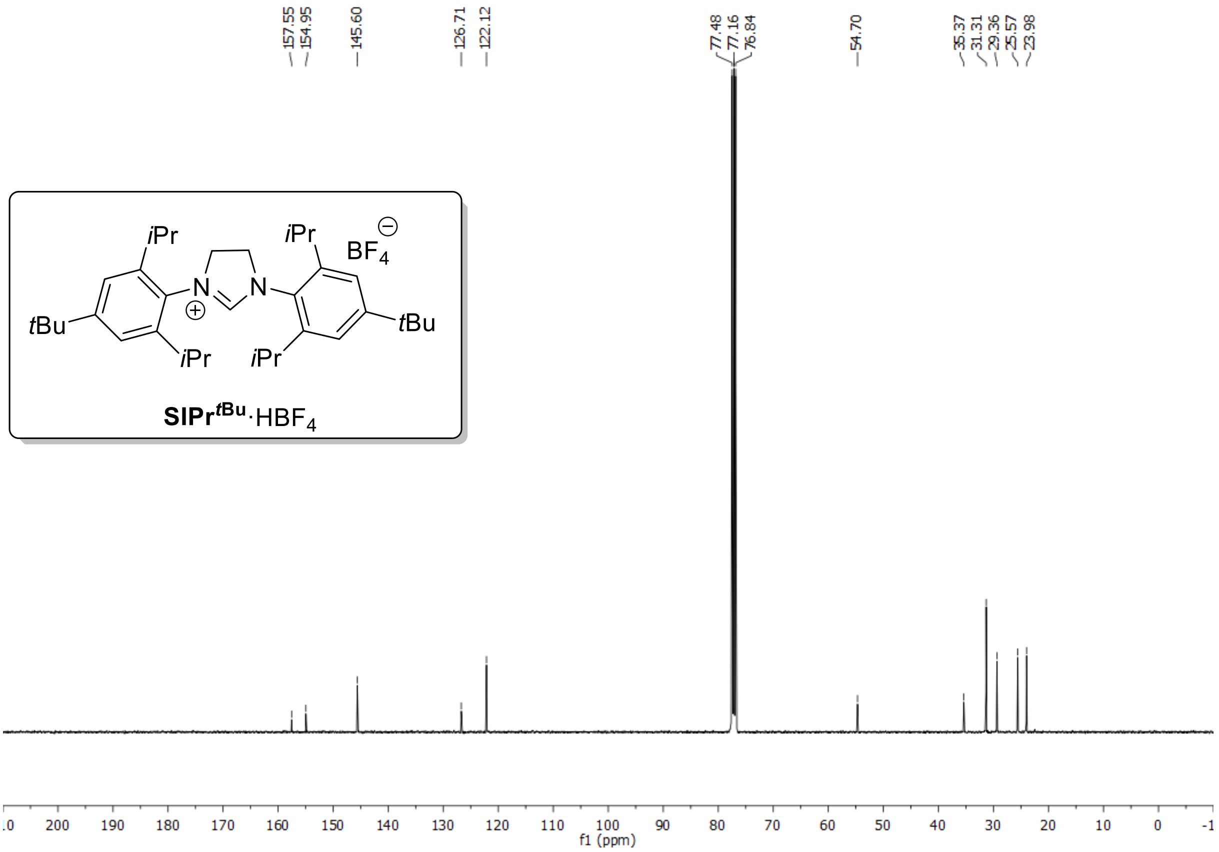
157.55
154.95
145.60
126.71
122.12
77.48
77.16
76.84
54.70
35.37
31.31
29.36
25.57
23.98
iPr
iPr
BF4
N
N
tBu
tBu
iPr
iPr
SIPr^tBu·HBF4
200 190 180 170 160 150 140 130 120 110 100 90 80 70 60 50 40 30 20 10 0
f1 (ppm)

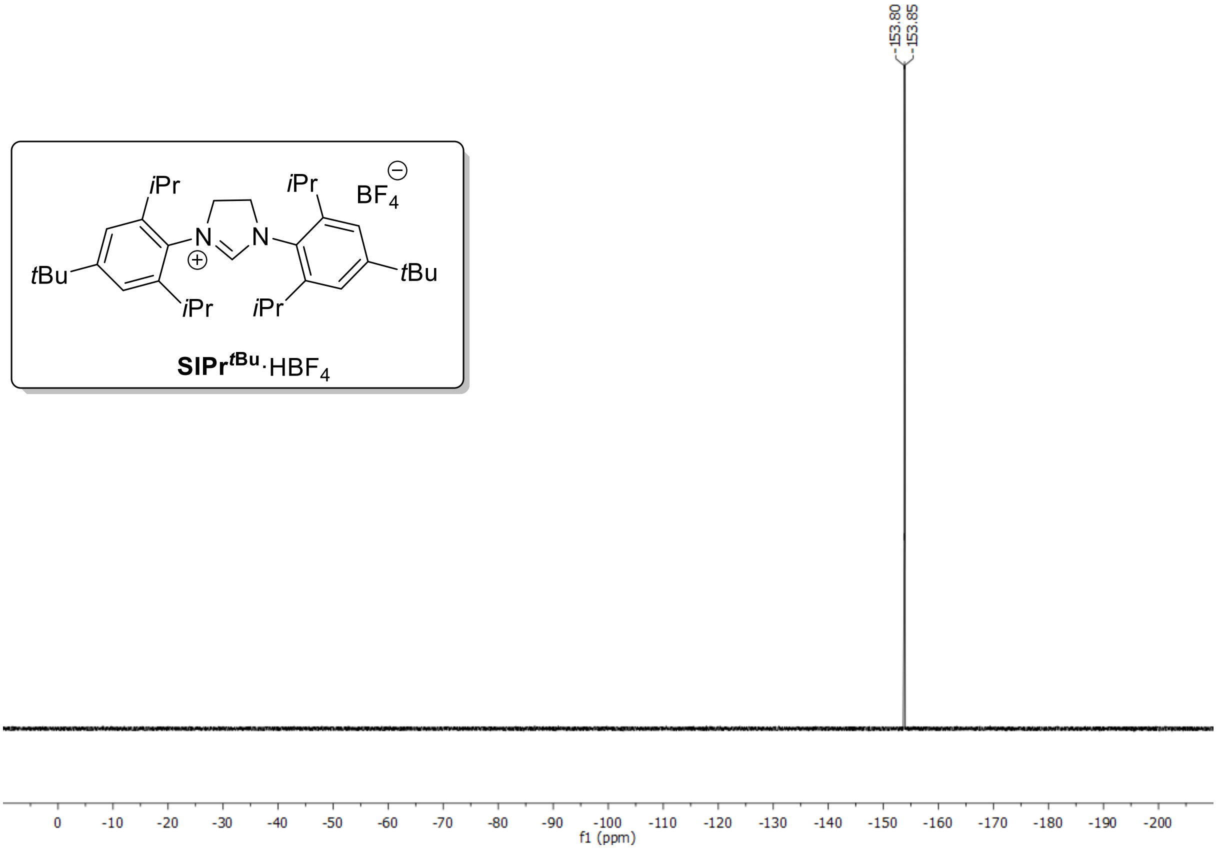
153.80
153.85
iPr
iPr
BF4
N
N
tBu
tBu
iPr
iPr
SIPr^tBu·HBF4
0 -10 -20 -30 -40 -50 -60 -70 -80 -90 -100 -110 -120 -130 -140 -150 -160 -170 -180 -190 -200
f1 (ppm)

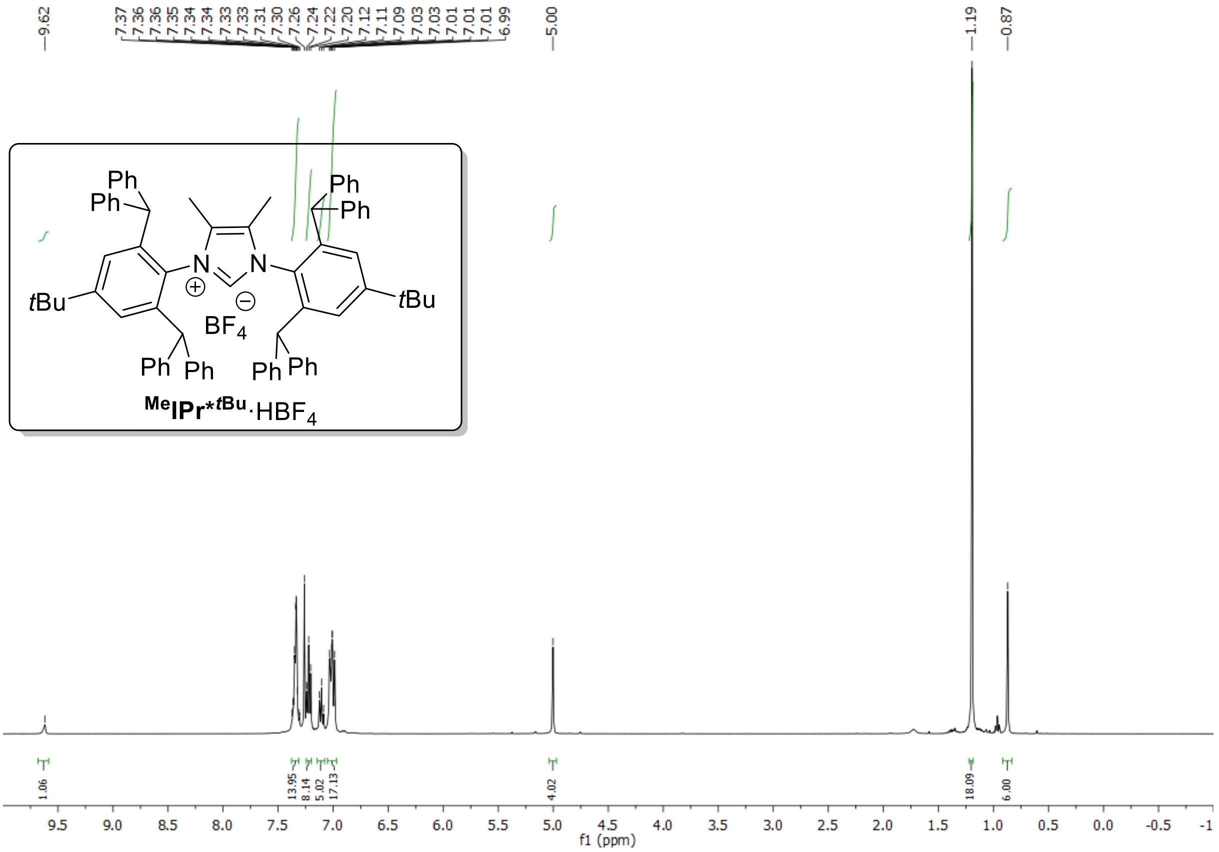
9.62
7.37 7.36 7.36 7.35 7.34 7.34 7.33 7.33 7.31 7.30 7.26 7.24 7.22 7.20 7.12 7.11 7.09 7.03 7.03 7.01 7.01 7.01 6.99
5.00
1.19
0.87
Ph
Ph
Ph
Ph
N
N
tBu
tBu
BF4
Ph
Ph
Ph
Ph
MeIPr*tBu·HBF4
1.06
13.95
8.14
5.02
17.13
4.02
18.09
6.00
f1 (ppm)

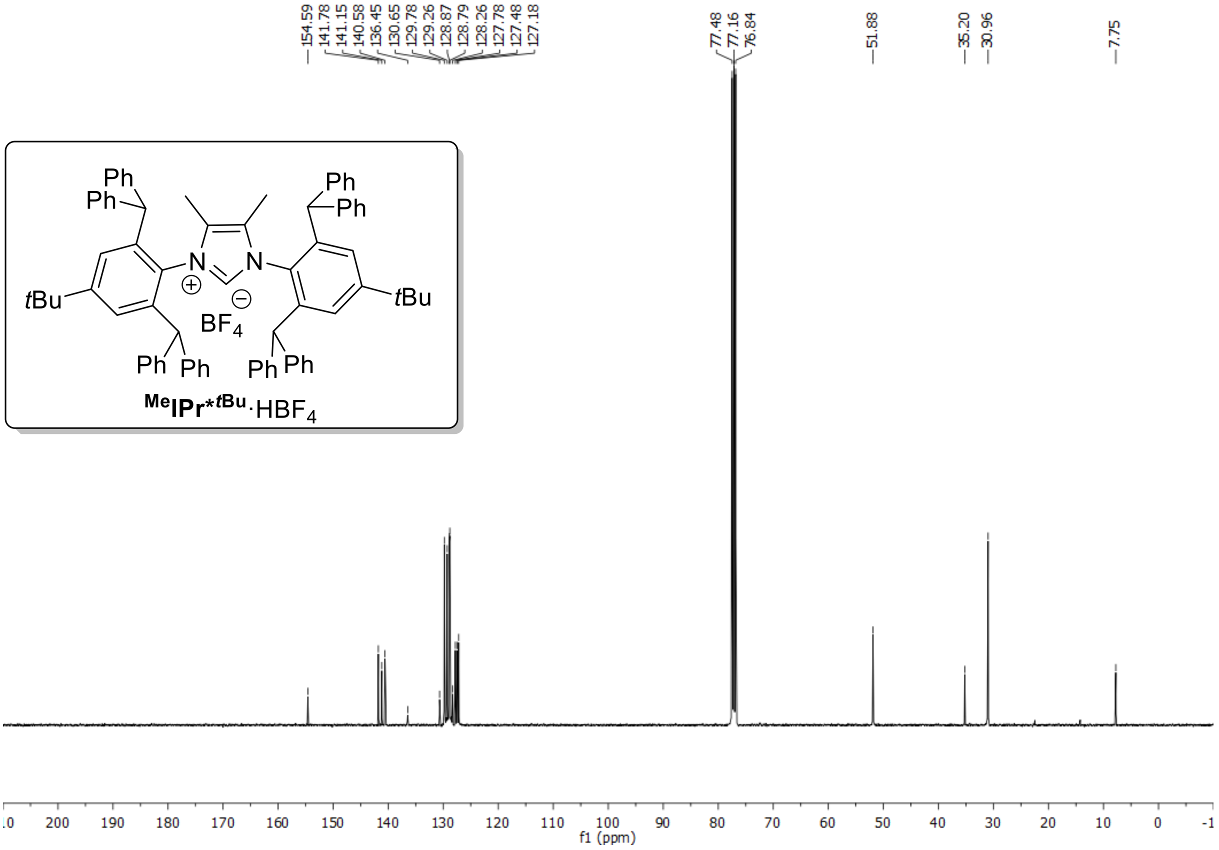
154.59 141.78 141.15 140.58 136.45 130.65 129.78 129.26 128.87 128.79 128.26 127.78 127.48 127.18
77.48 77.16 76.84
51.88
35.20
30.96
7.75
Ph
Ph
Ph
Ph
N
N
tBu
tBu
BF4
Ph
Ph
Ph
Ph
MeIPr*tBu·HBF4
f1 (ppm)

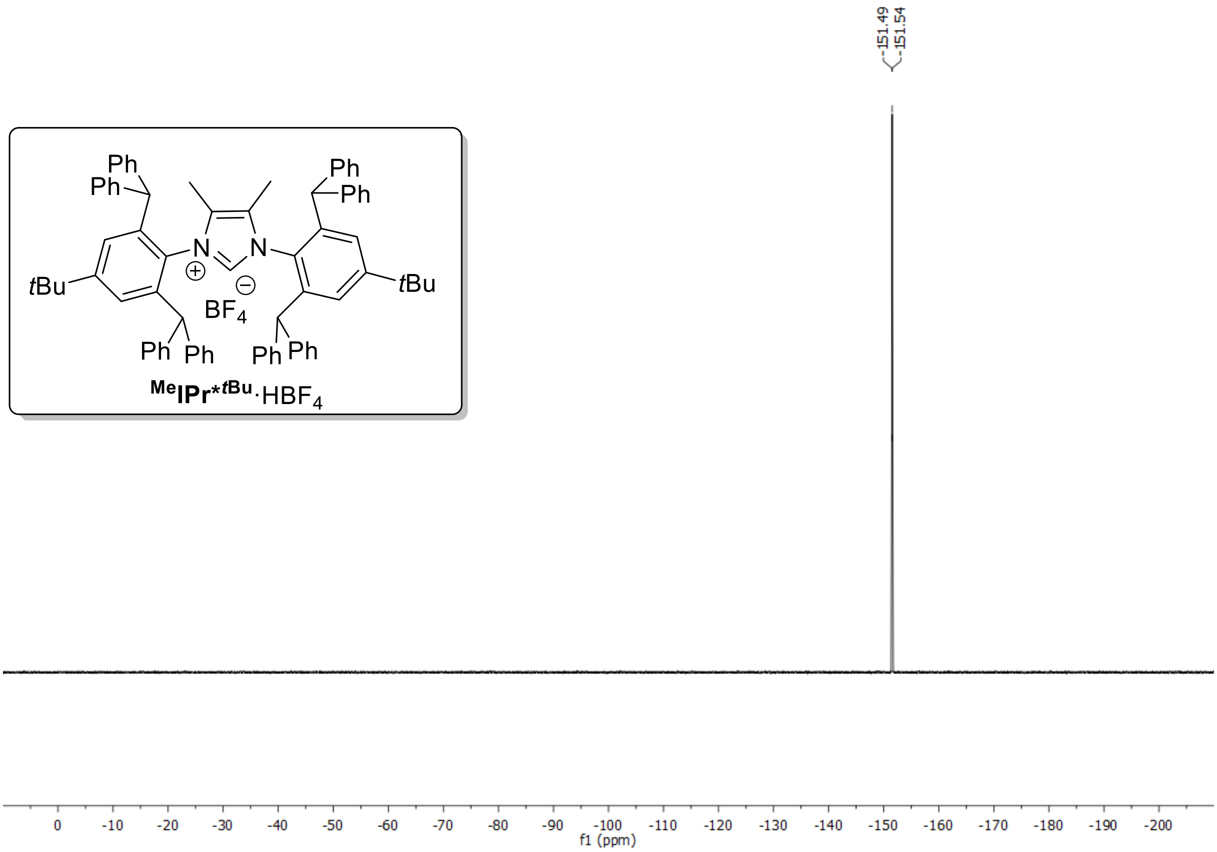
-151.49
-151.54
Ph
Ph
Ph
Ph
tBu
tBu
BF4
Ph
Ph
Ph
Ph
MeIPr*tBu·HBF4
f1 (ppm)

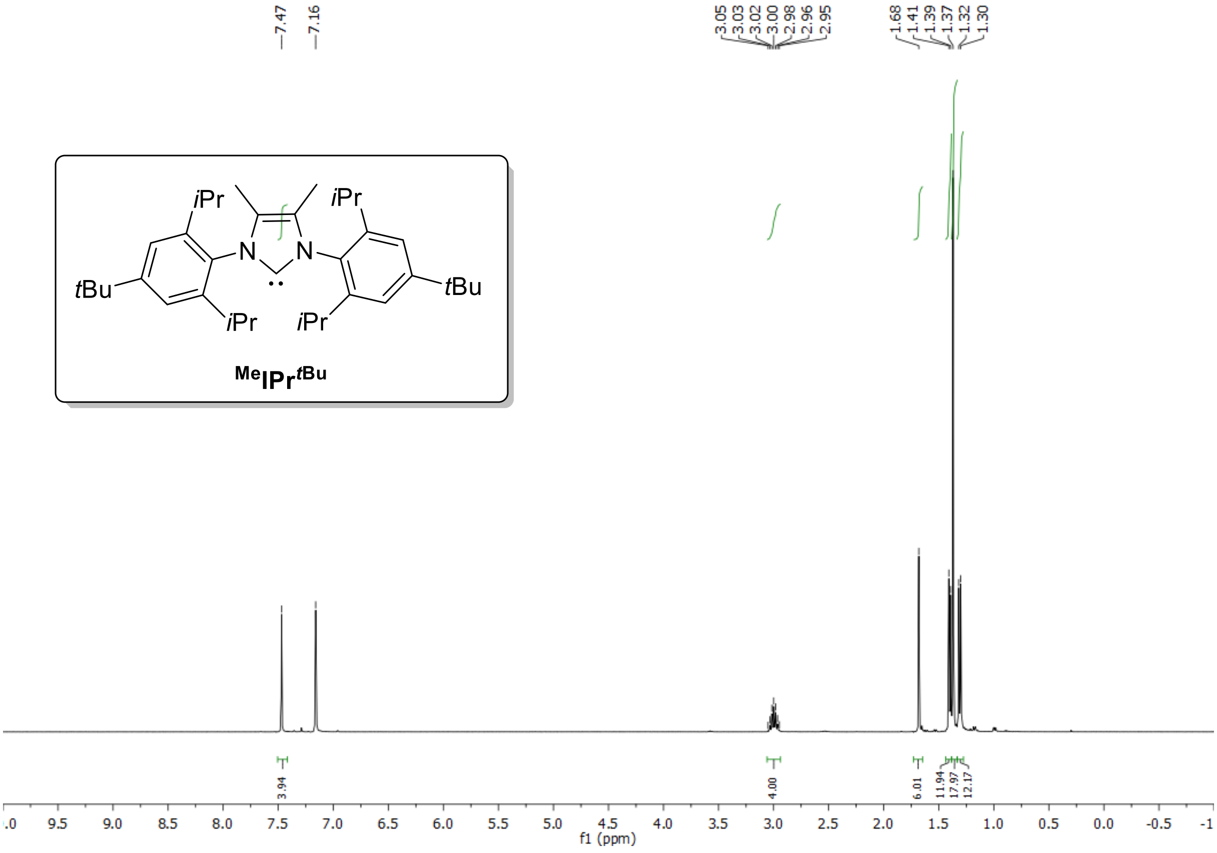
7.47
7.16
3.05
3.03
3.02
3.00
2.98
2.96
2.95
1.68
1.41
1.39
1.37
1.32
1.30
iPr
iPr
tBu
tBu
iPr
iPr
MeIPrtBu
3.94
4.00
6.01
11.94
17.97
12.17
f1 (ppm)

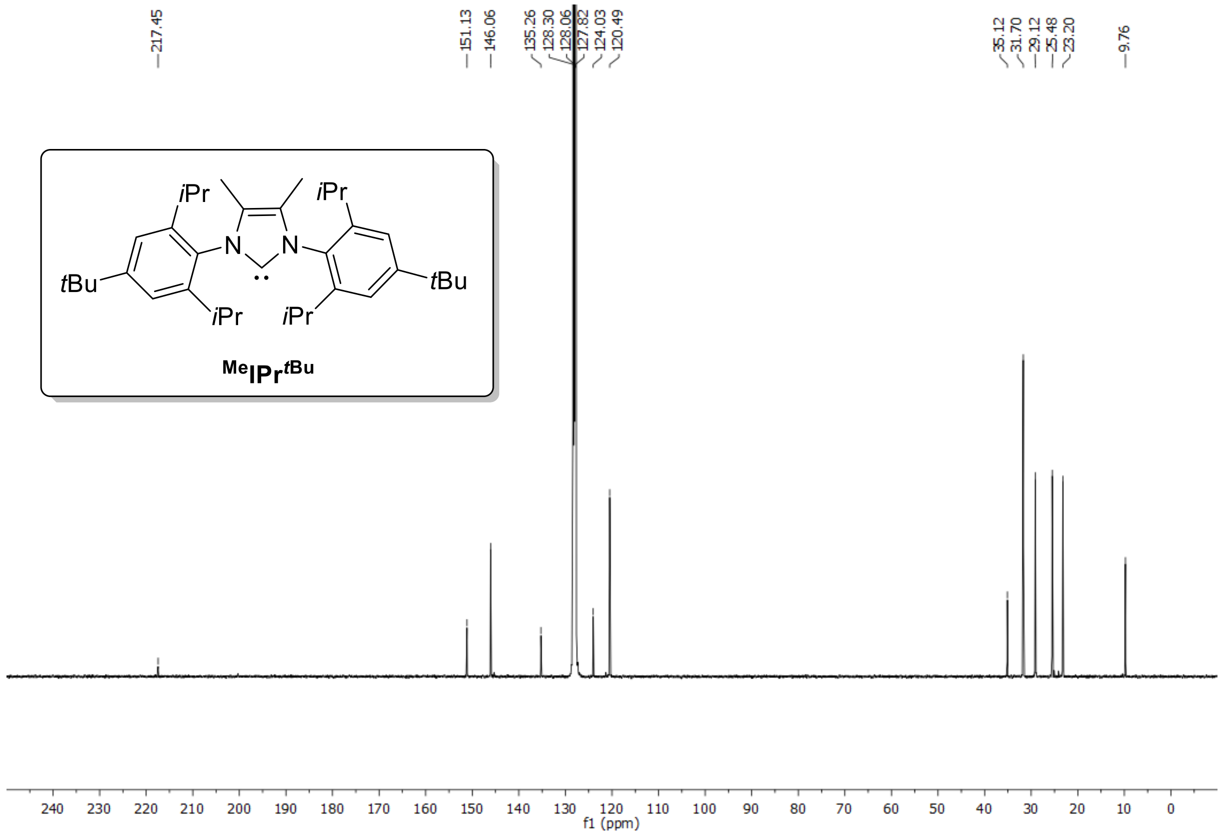
217.45
151.13
146.06
135.26
128.30
128.06
127.82
124.03
120.49
35.12
31.70
29.12
25.48
23.20
9.76
iPr
iPr
tBu
N
N
tBu
iPr
iPr
MeIPrtBu
f1 (ppm)

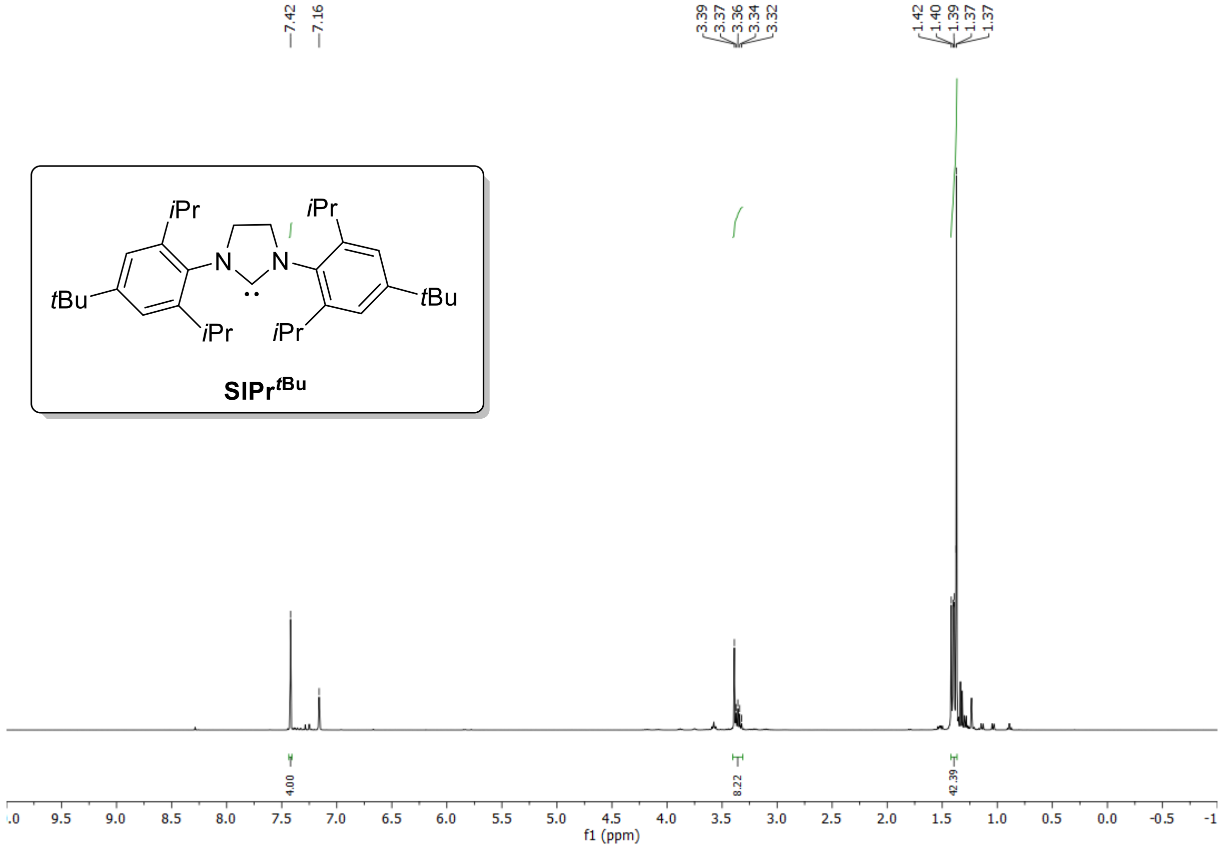
7.42
7.16
3.39
3.37
3.36
3.34
3.32
1.42
1.40
1.39
1.37
1.37
iPr
iPr
tBu
N
N
tBu
iPr
iPr
SIPrtBu
4.00
8.22
42.39
f1 (ppm)

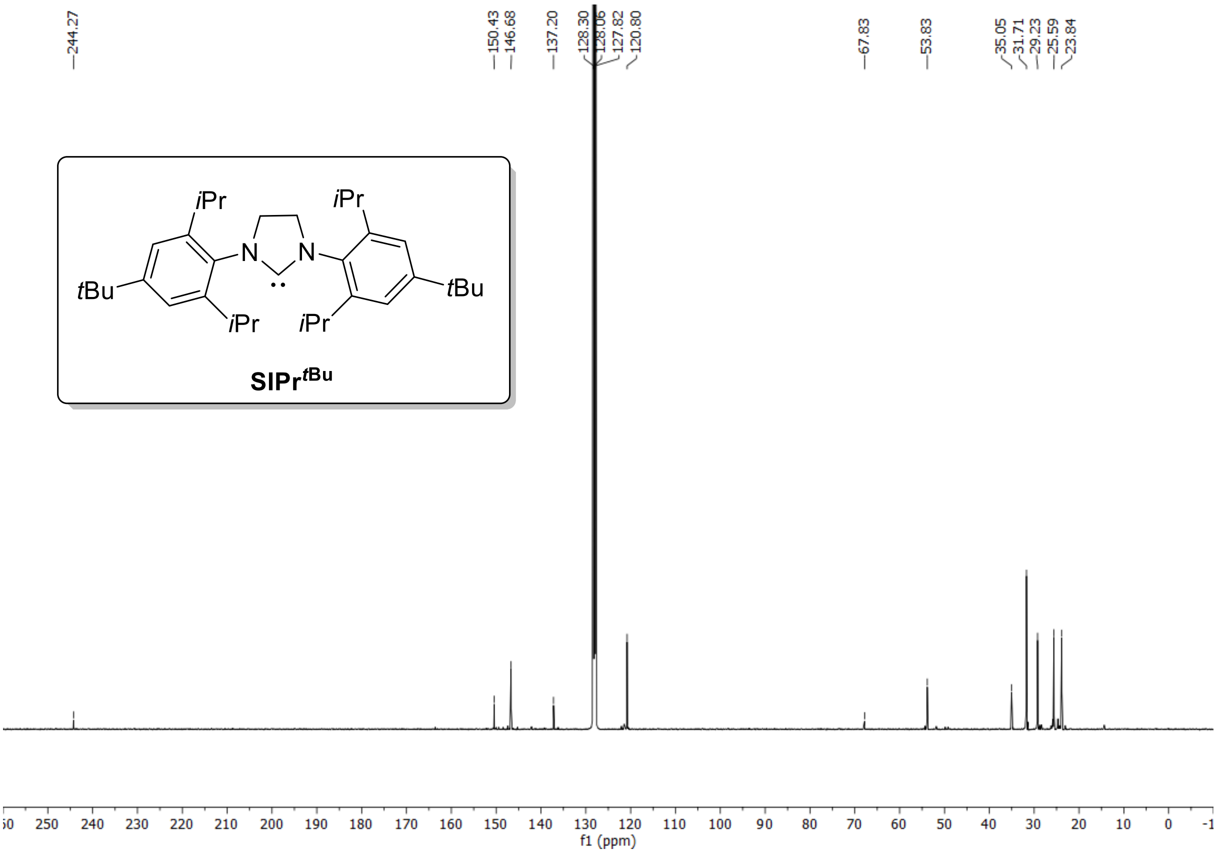

244.27
150.43
146.68
137.20
128.30
128.06
127.82
120.80
67.83
53.83
35.05
31.71
29.23
25.59
23.84
SIPr^{tBu}
f1 (ppm)

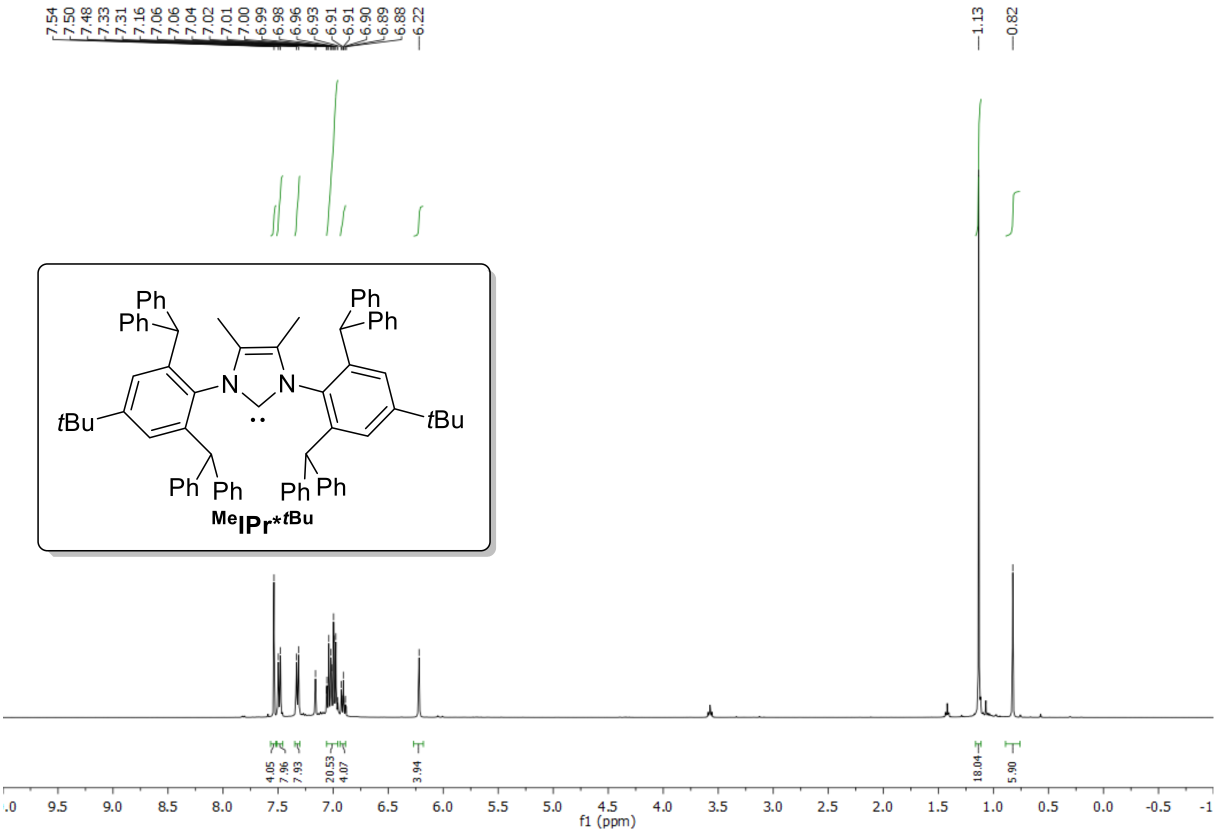

7.54
7.50
7.48
7.33
7.31
7.16
7.06
7.06
7.04
7.02
7.01
7.00
6.99
6.98
6.96
6.93
6.91
6.91
6.90
6.89
6.88
6.22
1.13
0.82
4.05
7.96
7.93
20.53
4.07
3.94
18.04
5.90
^{Me}IPr*^{tBu}
f1 (ppm)

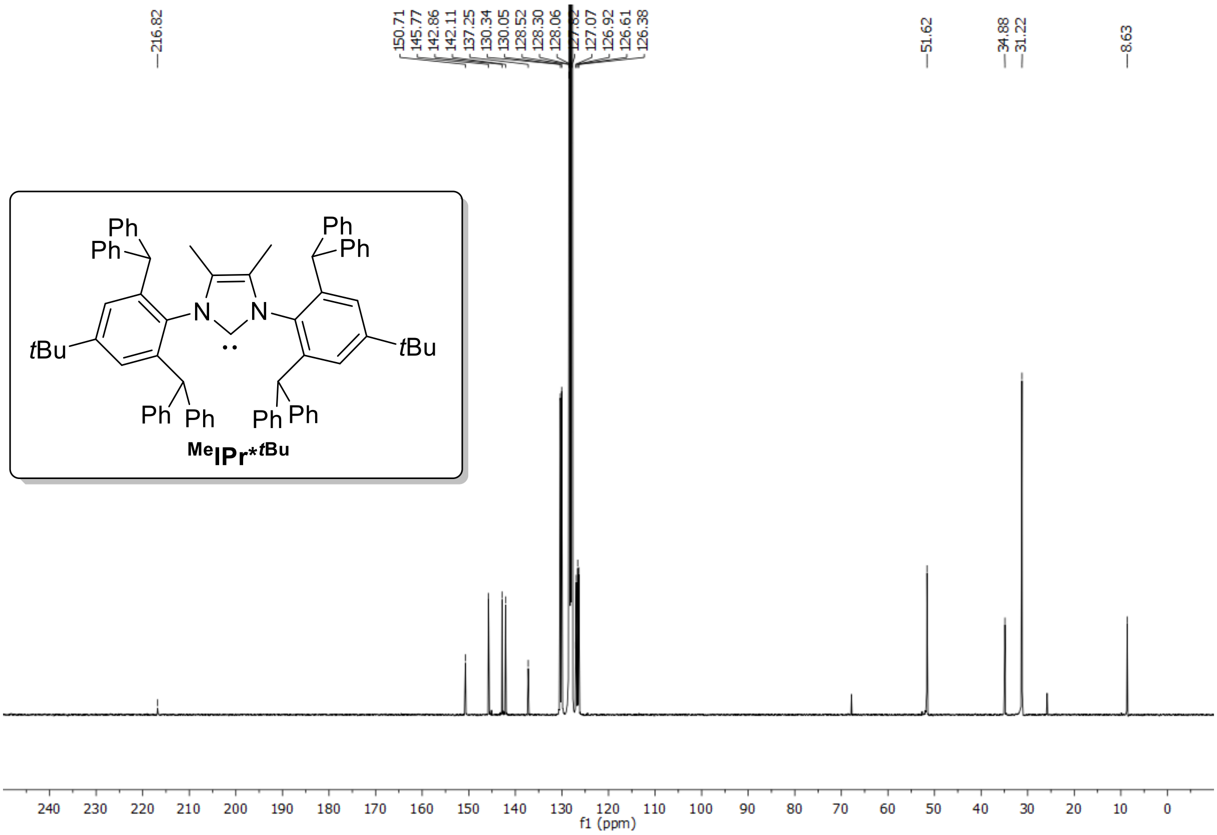
216.82
150.71
145.77
142.86
142.11
137.25
130.34
130.05
128.52
128.30
128.06
127.82
127.07
126.92
126.61
126.38
51.62
34.88
31.22
8.63
Ph
Ph
Ph
Ph
tBu
N
N
tBu
Ph
Ph
Ph
Ph
MeIPr*tBu
f1 (ppm)

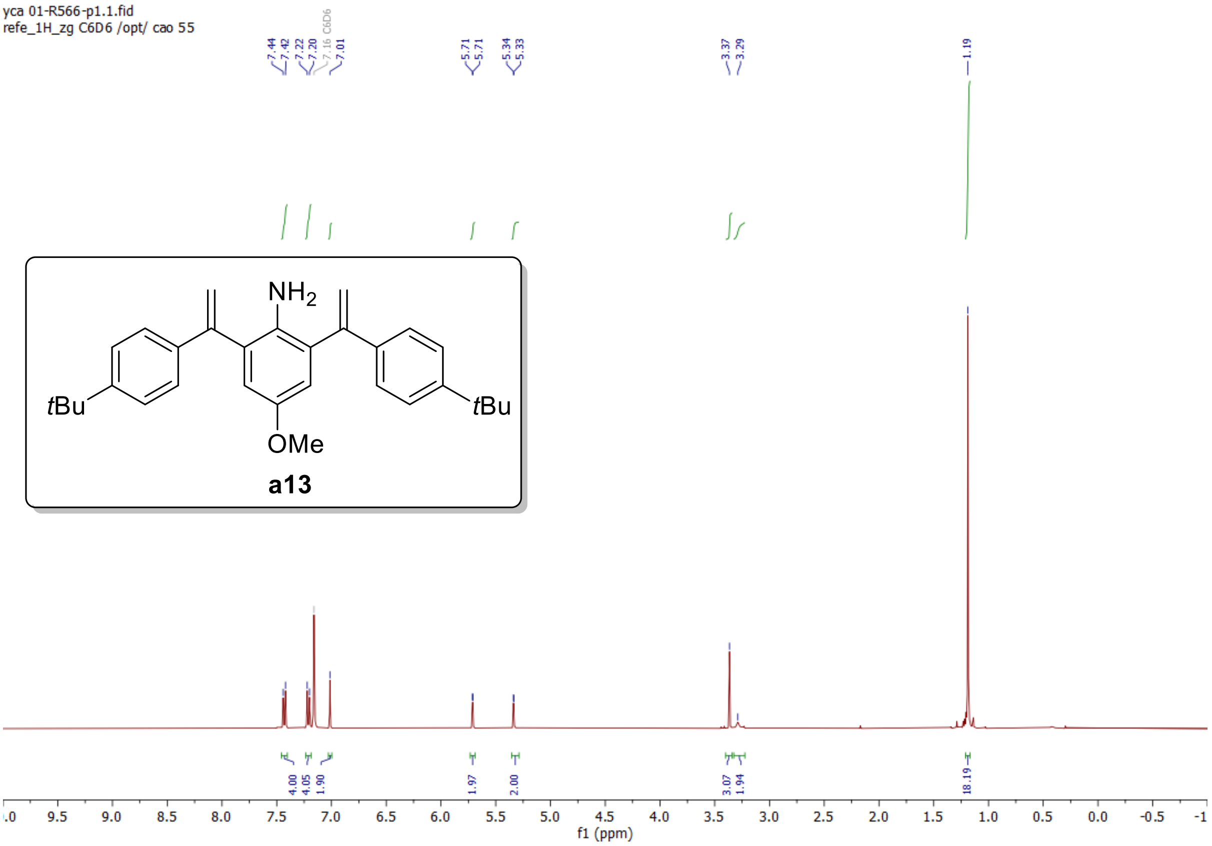
yca 01-R566-p1.1.fid
refe_1H_zg C6D6 /opt/ cao 55
7.44
7.42
7.22
7.20
7.16 C6D6
7.01
5.71
5.71
5.34
5.33
3.37
3.29
1.19
NH2
tBu
tBu
OMe
a13
4.00
4.05
1.90
1.97
2.00
3.07
1.94
18.19
f1 (ppm)

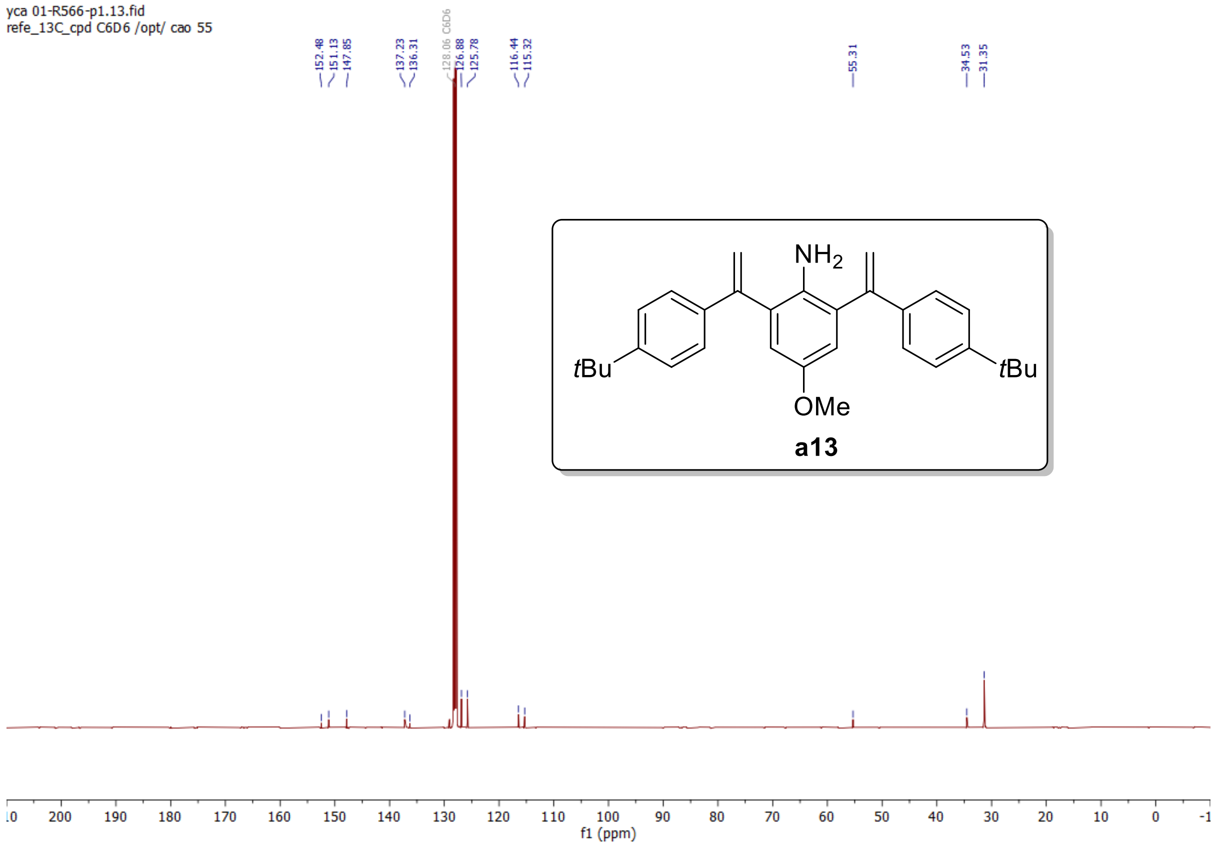
yca 01-R566-p1.13.fid
refe_13C_cpd C6D6 /opt/ cao 55
152.48
151.13
147.85
137.23
136.31
128.06 C6D6
126.88
125.78
116.44
115.32
55.31
34.53
31.35
NH2
tBu
tBu
OMe
a13
f1 (ppm)

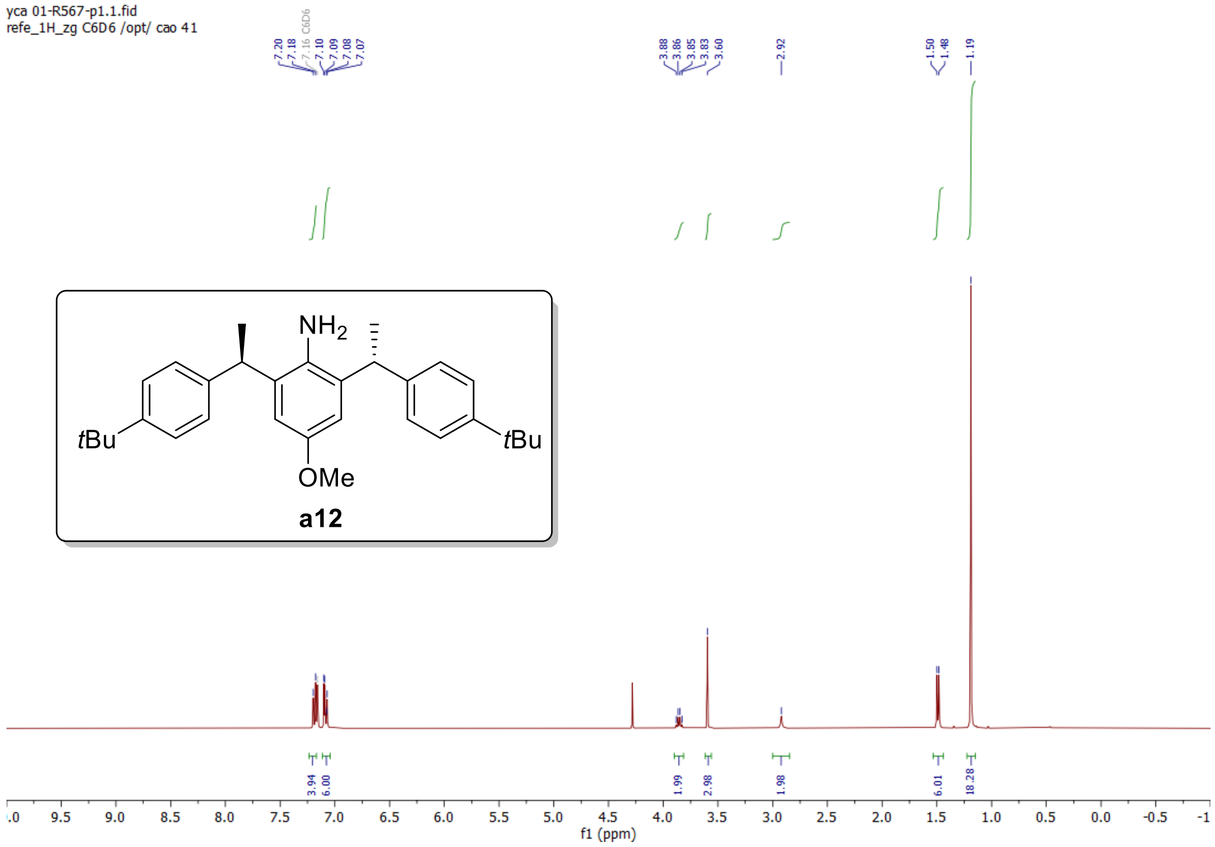
yca 01-R567-p1.1.fid
refe_1H_zg C6D6 /opt/ cao 41
7.20
7.18
7.16 C6D6
7.10
7.09
7.08
7.07
3.88
3.86
3.85
3.83
3.60
2.92
1.50
1.48
1.19
NH2
tBu
tBu
OMe
a12
3.94
6.00
1.99
2.98
1.98
6.01
18.28
f1 (ppm)

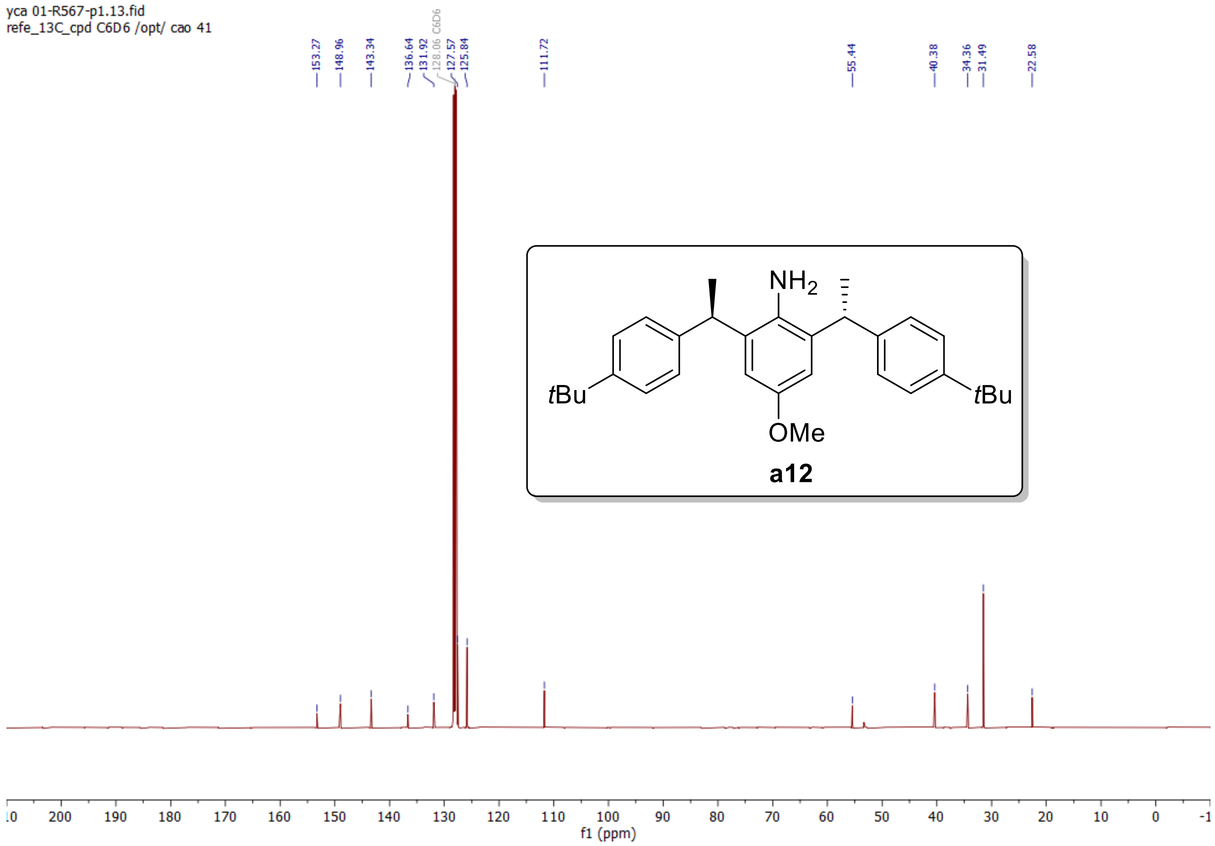
yca 01-R567-p1.13.fid
refe_13C_cpd C6D6 /opt/ cao 41
153.27
148.96
143.34
136.64
131.92
128.06 C6D6
127.57
125.84
111.72
55.44
40.38
34.36
31.49
22.58
NH2
tBu
tBu
OMe
a12
f1 (ppm)

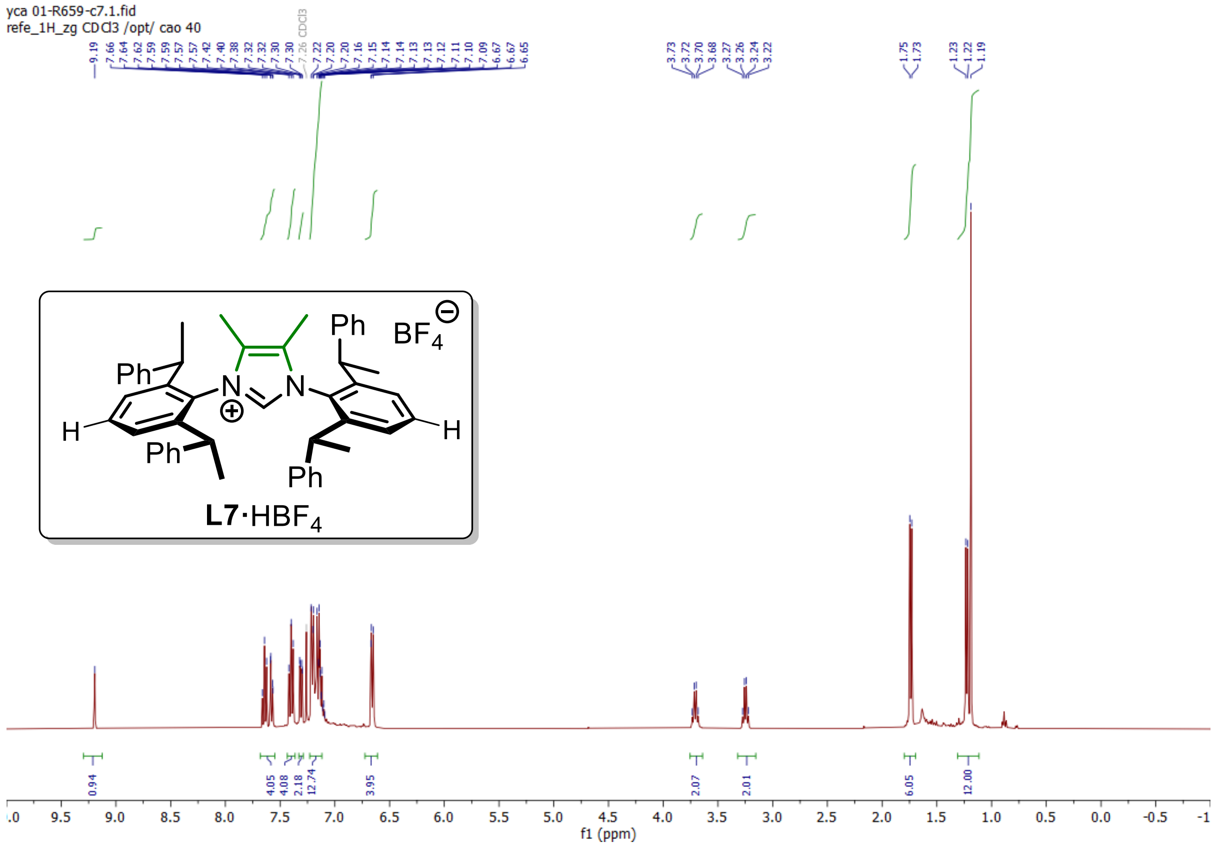
yca 01-R659-c7.1.fid
refe_1H_zg CDCl3 /opt/ cao 40
9.19
7.66
7.64
7.62
7.59
7.57
7.57
7.42
7.40
7.38
7.32
7.30
7.30
7.26 CDCl3
7.22
7.20
7.20
7.16
7.15
7.14
7.14
7.13
7.13
7.12
7.11
7.10
7.09
6.67
6.67
6.65
3.73
3.72
3.70
3.68
3.27
3.26
3.24
3.22
1.75
1.73
1.23
1.22
1.19
Ph
BF4
Ph
N
N
H
H
Ph
Ph
L7·HBF4
0.94
4.05
4.08
2.18
12.74
3.95
2.07
2.01
6.05
12.00
f1 (ppm)

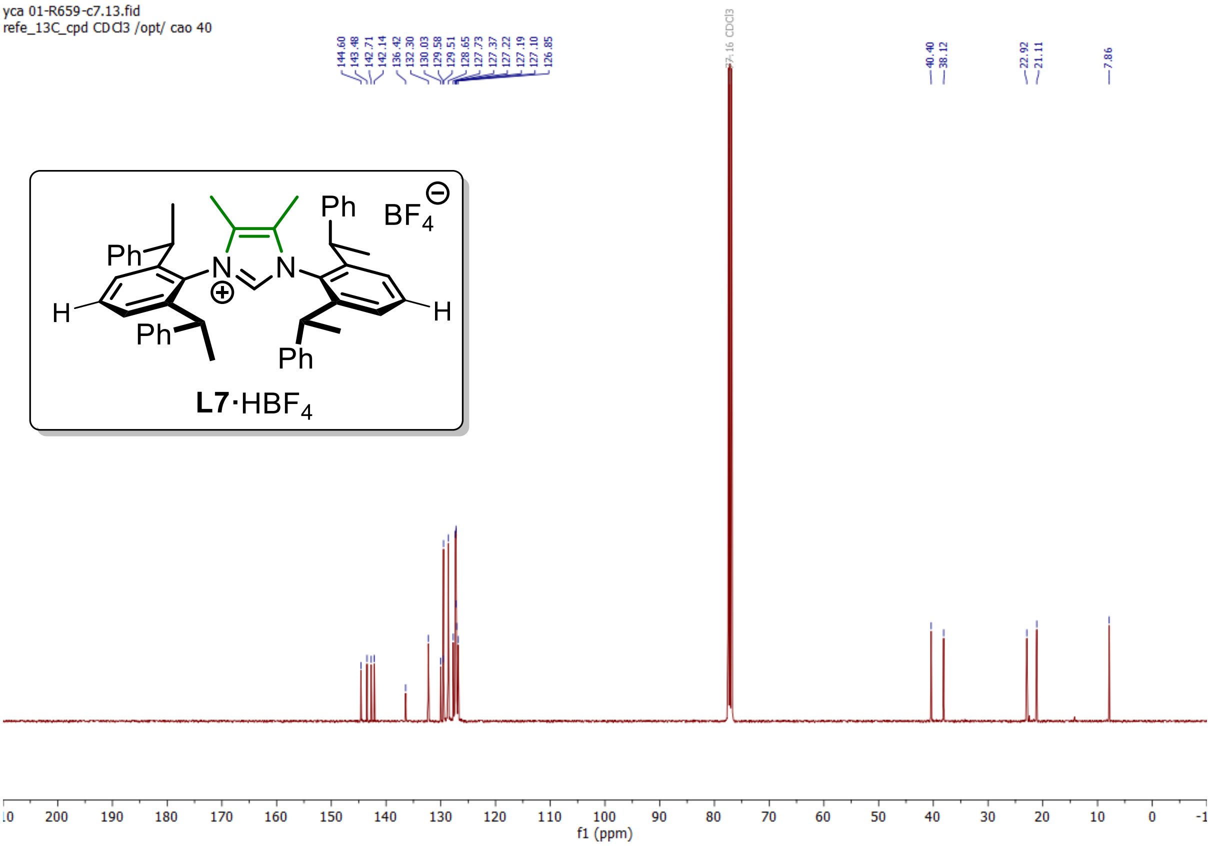
yca 01-R659-c7.13.fid
refe_13C_cpd CDCl3 /opt/ cao 40
144.60
143.48
142.71
142.14
136.42
132.30
130.03
129.58
129.51
128.65
127.73
127.37
127.22
127.19
127.10
126.85
77.16 CDCl3
40.40
38.12
22.92
21.11
7.86
L7·HBF4
f1 (ppm)

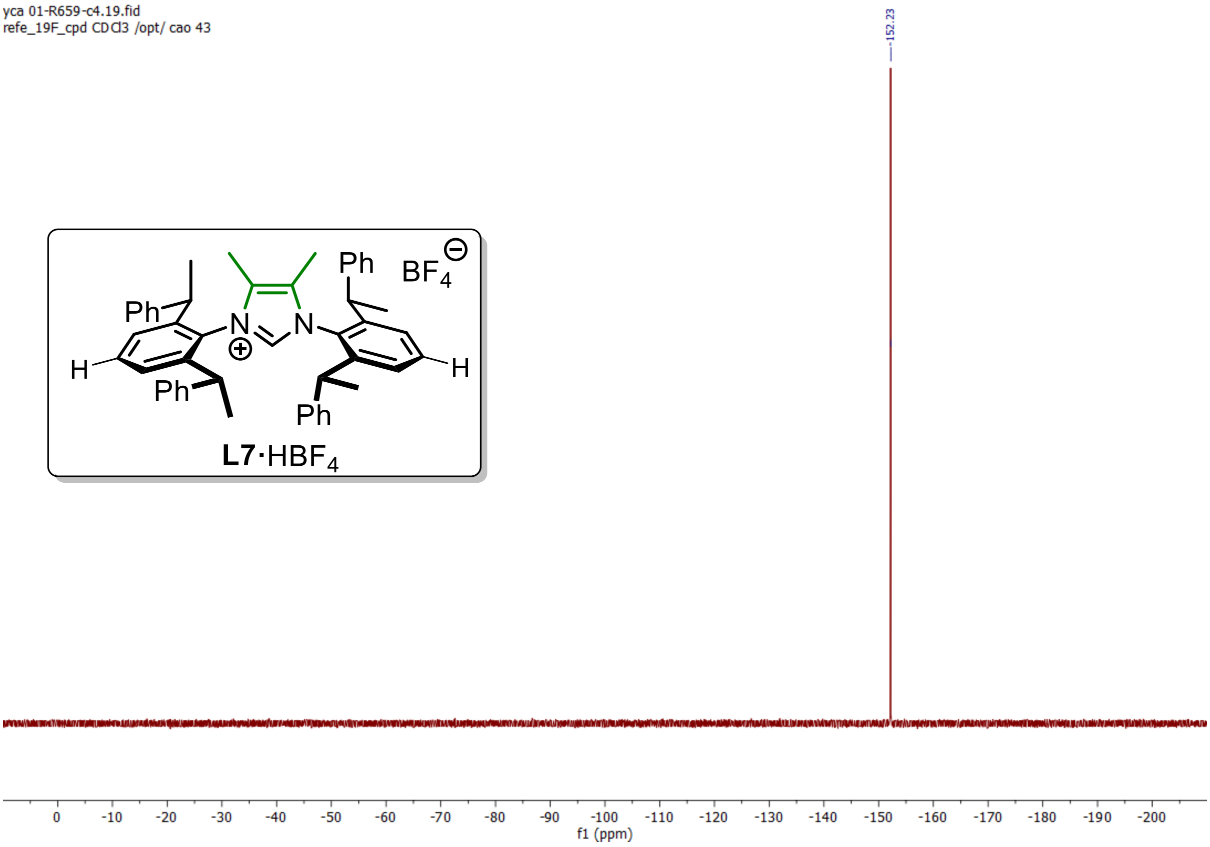
yca 01-R659-c4.19.fid
refe_19F_cpd CDCl3 /opt/ cao 43
-152.23
L7·HBF4
f1 (ppm)

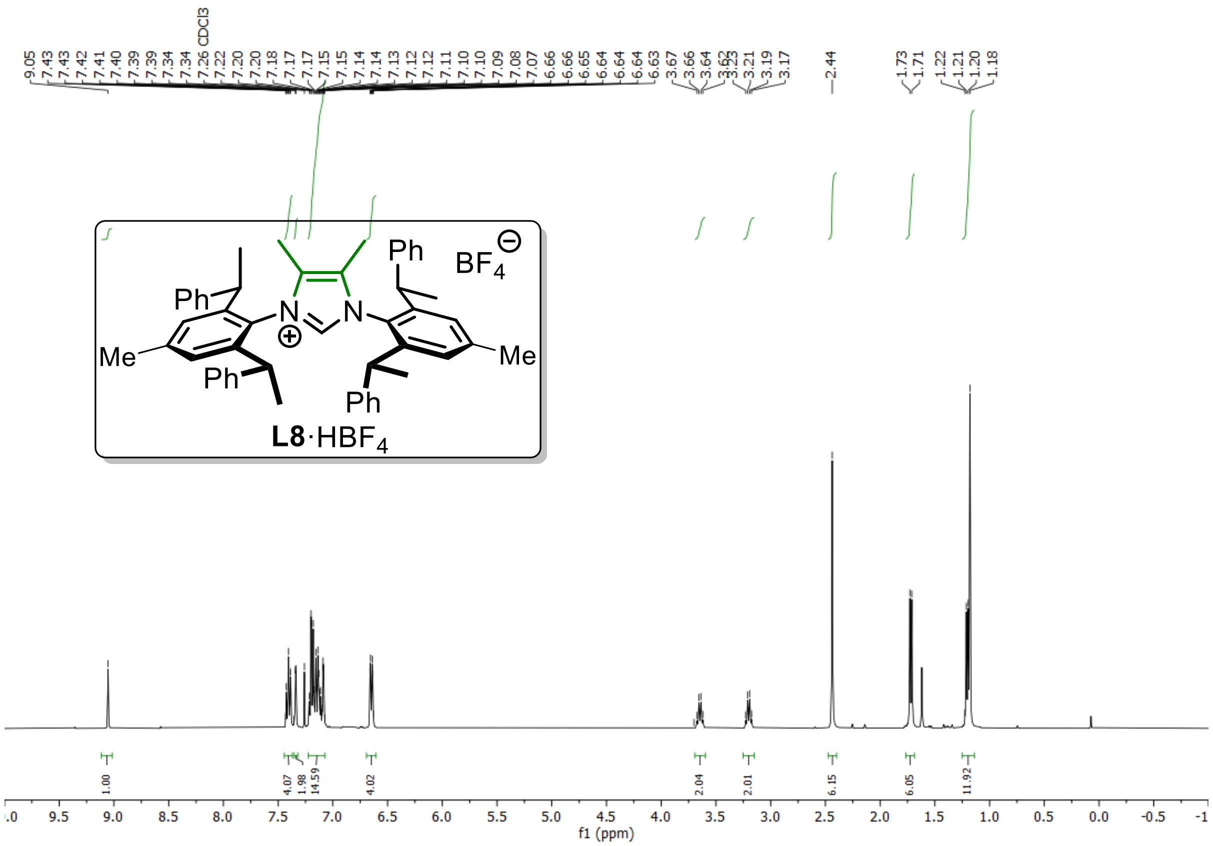

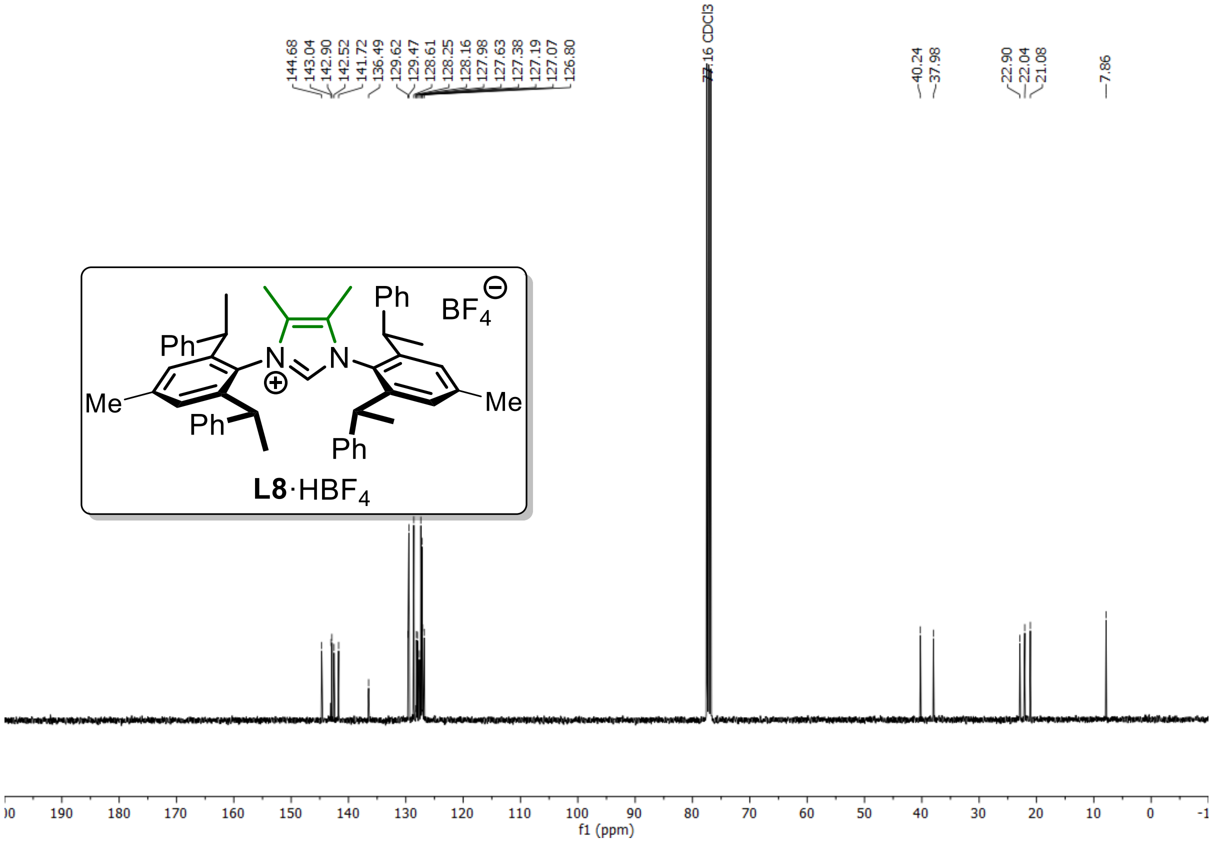

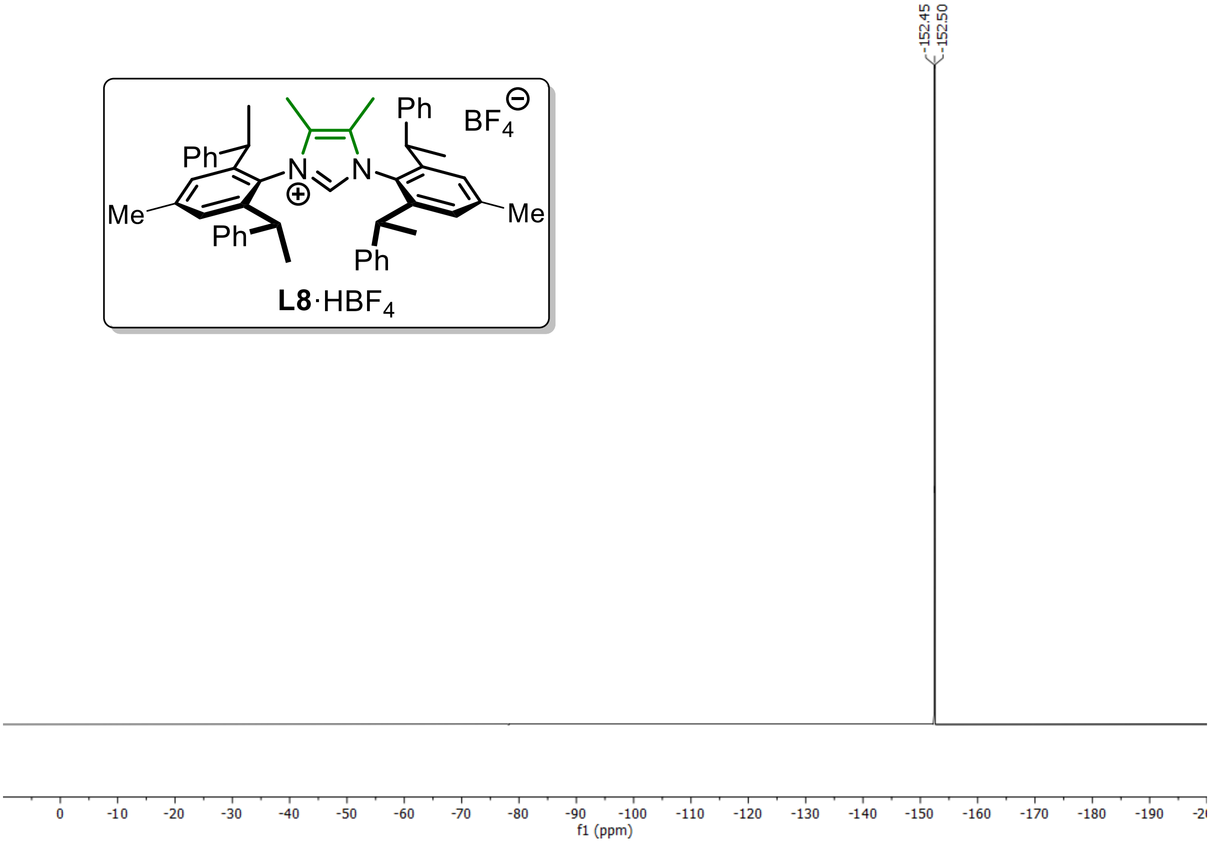
-152.45
-152.50
Ph
BF4
Me
N
N
L8·HBF4
f1 (ppm)

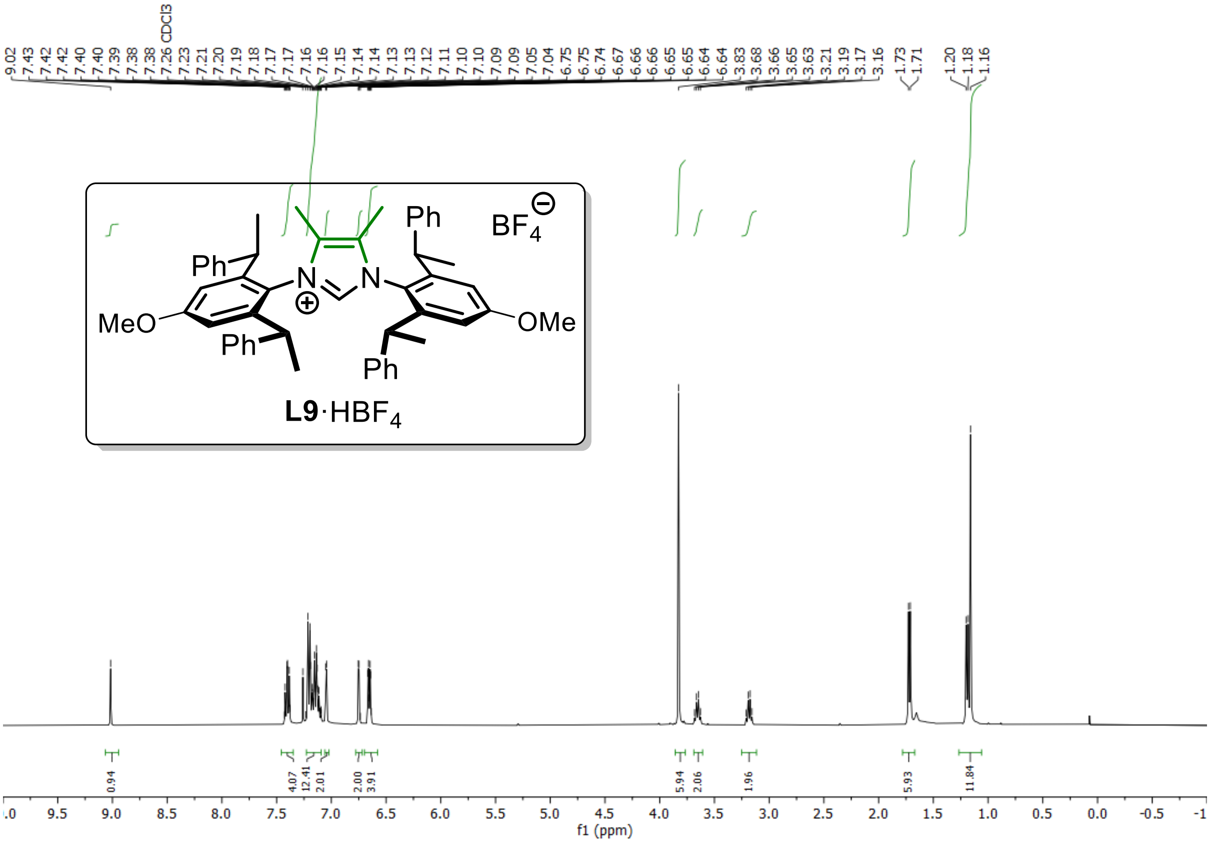
CDCl3
Ph
BF4
MeO
OMe
N
N
L9·HBF4
f1 (ppm)

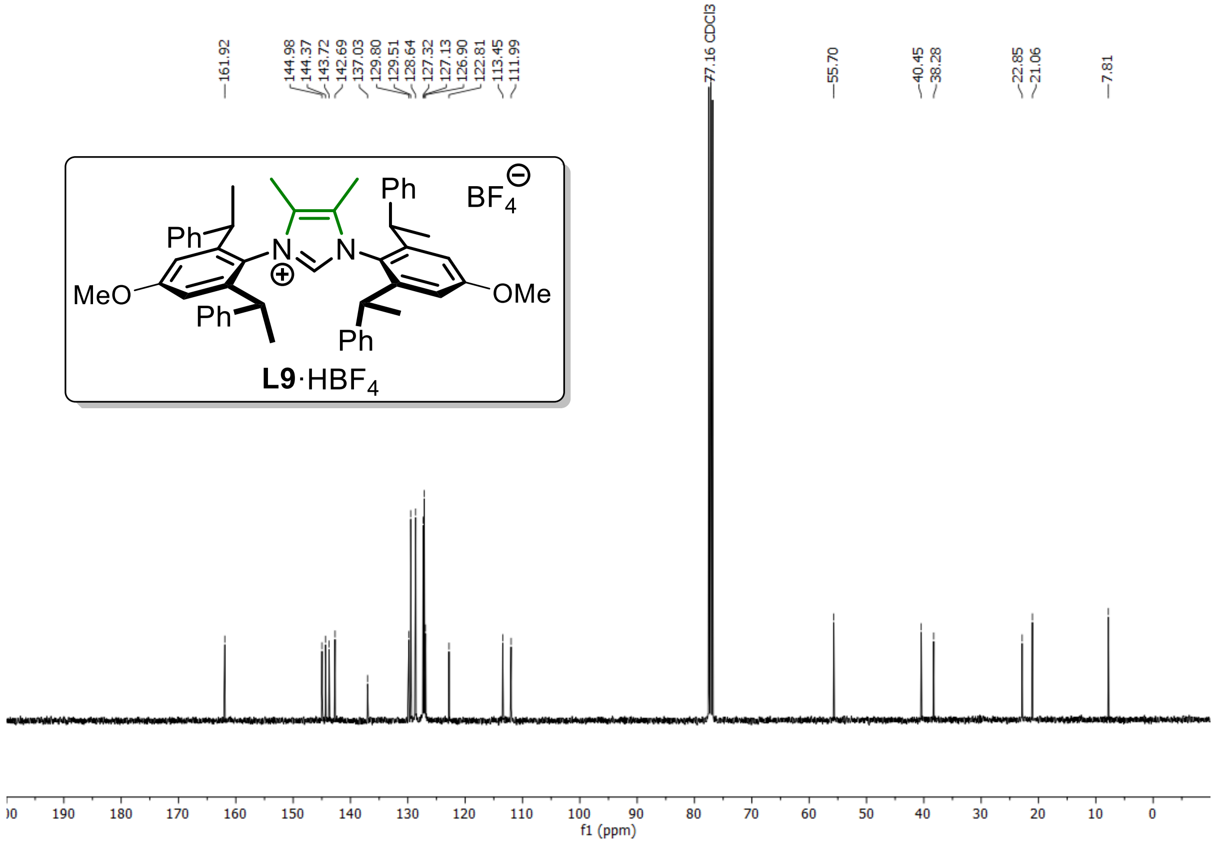
161.92
144.98
144.37
143.72
142.69
137.03
129.80
129.51
128.64
127.32
127.13
126.90
122.81
113.45
111.99
77.16 CDCl3
55.70
40.45
38.28
22.85
21.06
7.81
MeO
OMe
Ph
BF4
L9·HBF4
f1 (ppm)

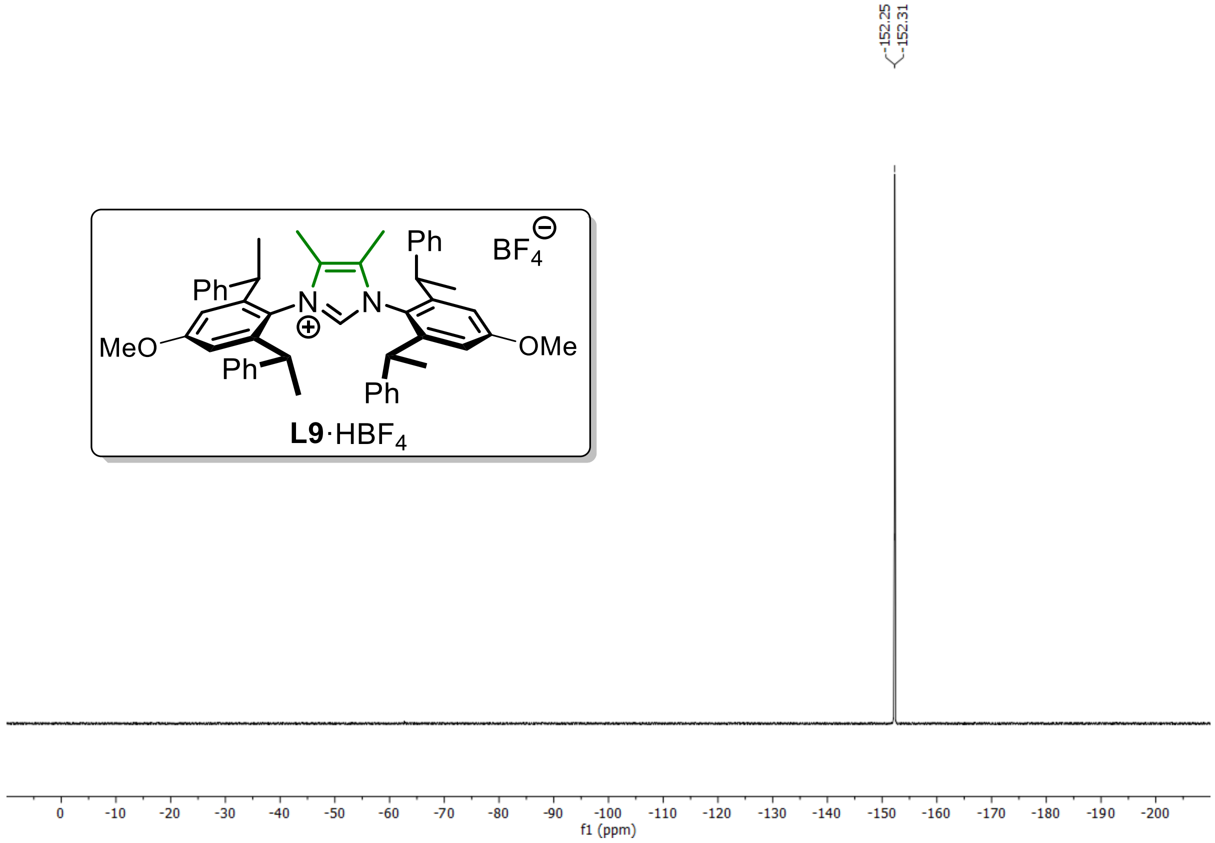
-152.25
-152.31
MeO
OMe
Ph
BF4
L9·HBF4
f1 (ppm)

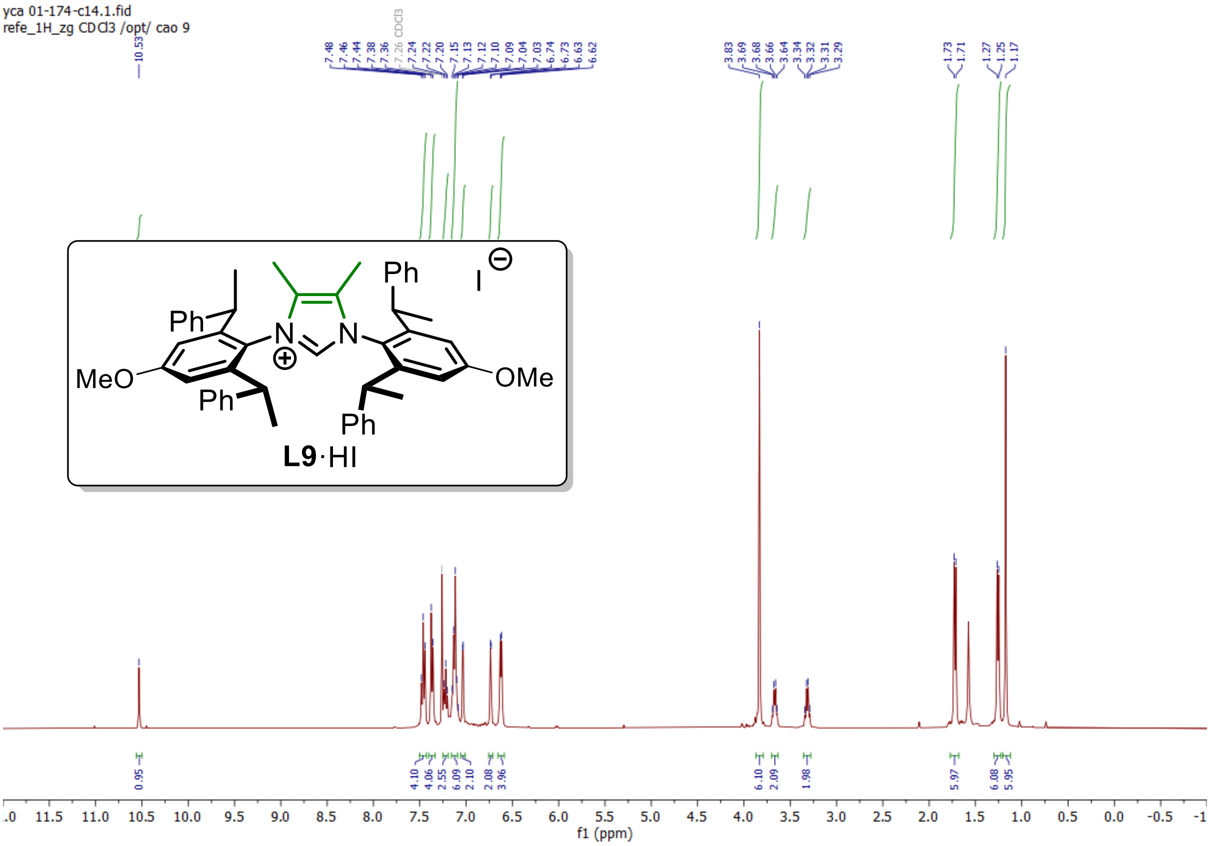

yca 01-174-c14.1.fid
refe_1H_zg CDCl3 /opt/ cao 9
10.53
7.48 7.46 7.44 7.38 7.36 7.26 CDCl3 7.24 7.22 7.20 7.15 7.13 7.12 7.10 7.09 7.04 7.03 6.74 6.73 6.63 6.62
3.83 3.69 3.68 3.66 3.64 3.34 3.32 3.31 3.29
1.73 1.71 1.27 1.25 1.17
0.95 4.10 4.06 2.55 6.09 2.10 2.08 3.96 6.10 2.09 1.98 5.97 6.08 5.95
L9·HI
MeO
OMe
Ph
I
f1 (ppm)

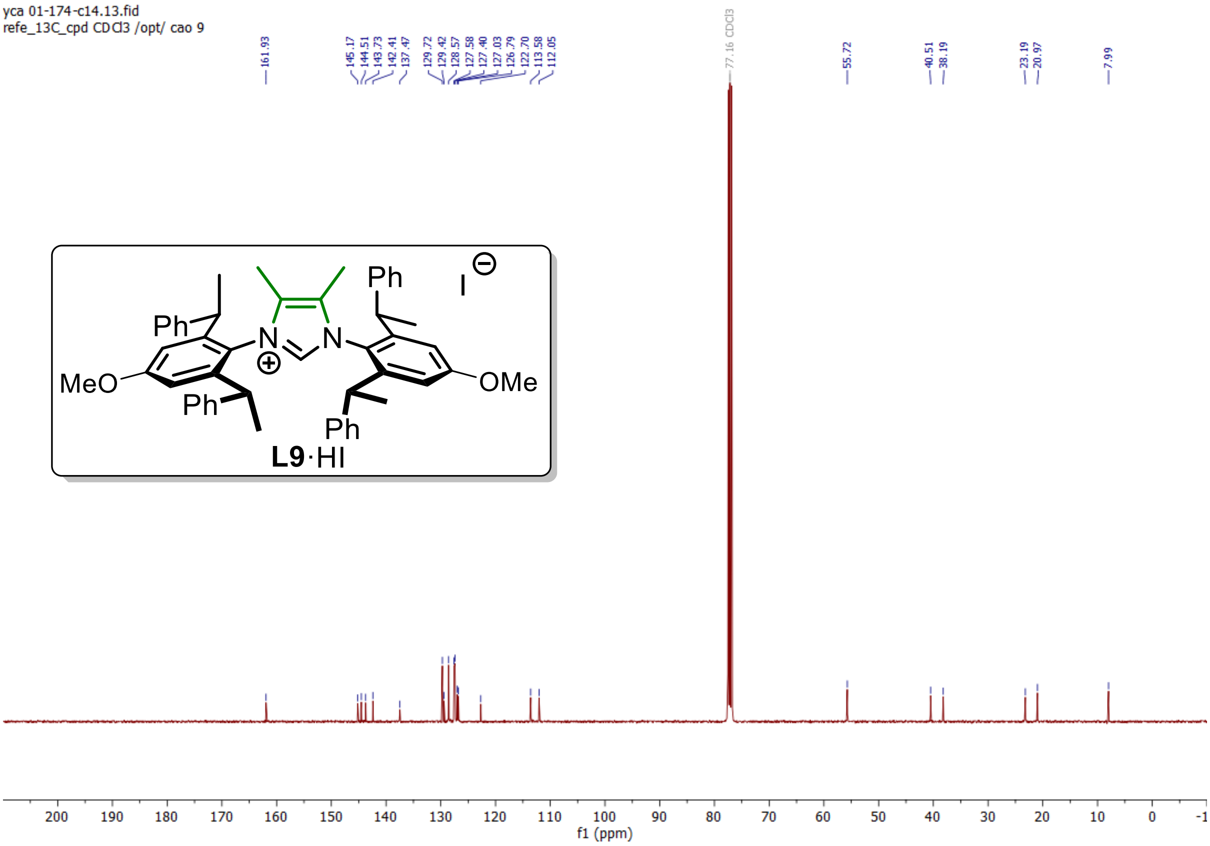

yca 01-174-c14.13.fid
refe_13C_cpd CDCl3 /opt/ cao 9
161.93
145.17 144.51 143.73 142.41 137.47 129.72 129.42 128.57 127.58 127.40 127.03 126.79 122.70 113.58 112.05
77.16 CDCl3
55.72
40.51 38.19
23.19 20.97
7.99
L9·HI
MeO
OMe
Ph
I
f1 (ppm)

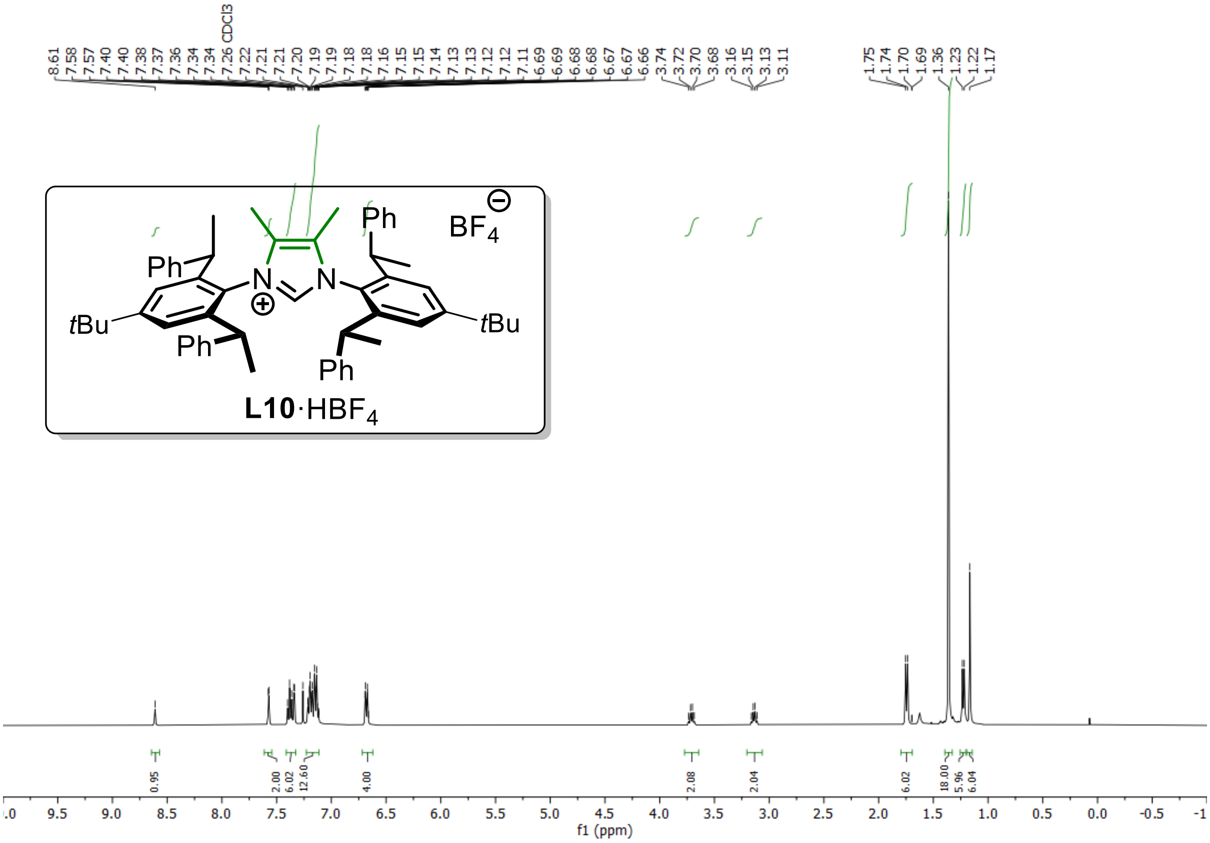
8.61
7.58
7.57
7.40
7.40
7.38
7.37
7.36
7.34
7.34
7.26 CDCl3
7.22
7.21
7.21
7.20
7.19
7.19
7.18
7.16
7.15
7.15
7.14
7.13
7.13
7.12
7.12
7.11
6.69
6.69
6.68
6.68
6.67
6.67
6.66
3.74
3.72
3.70
3.68
3.16
3.15
3.13
3.11
1.75
1.74
1.70
1.69
1.36
1.23
1.22
1.17
Ph
BF4
Ph
N
N
tBu
tBu
Ph
Ph
L10·HBF4
0.95
2.00
6.02
12.60
4.00
2.08
2.04
6.02
18.00
5.96
6.04
f1 (ppm)

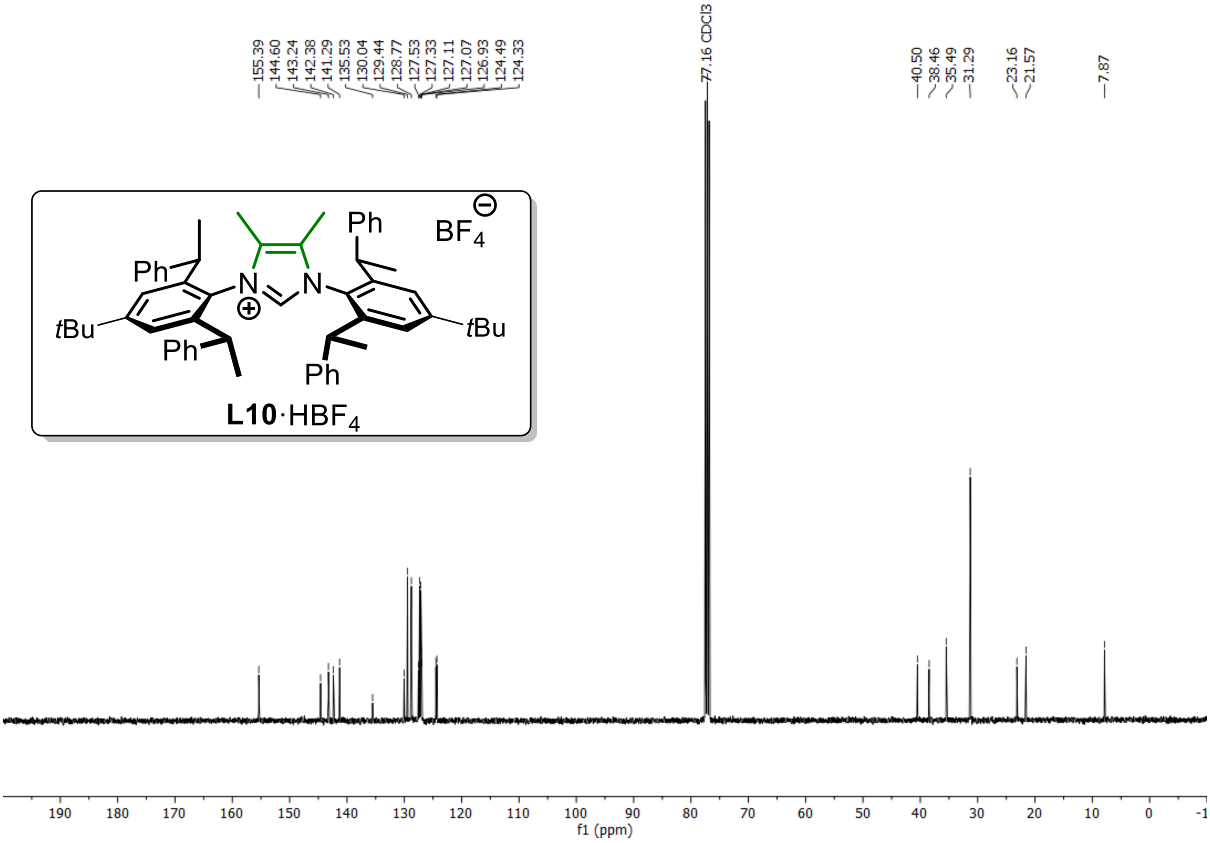
155.39
144.60
143.24
142.38
141.29
135.53
130.04
129.44
128.77
127.53
127.33
127.11
127.07
126.93
124.49
124.33
77.16 CDCl3
40.50
38.46
35.49
31.29
23.16
21.57
7.87
Ph
BF4
Ph
N
N
tBu
tBu
Ph
Ph
L10·HBF4
f1 (ppm)

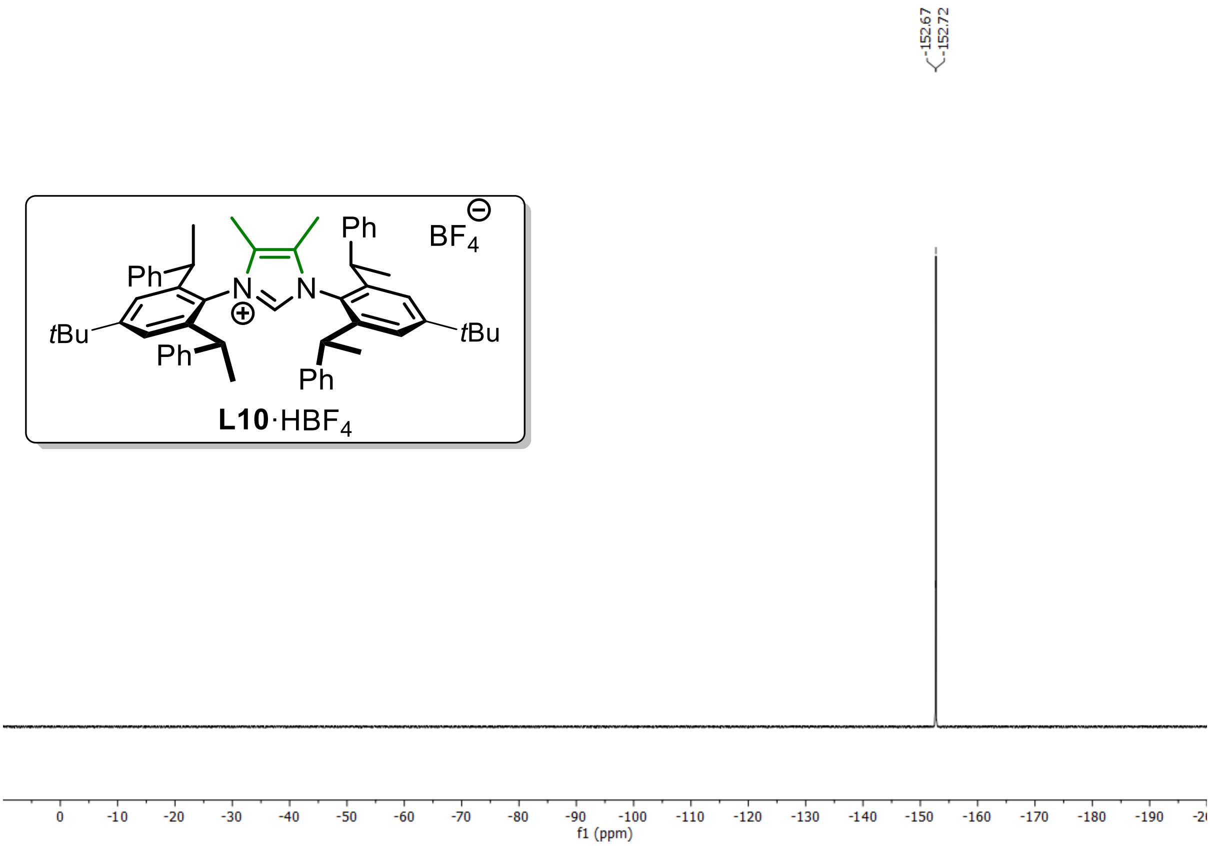

-152.67
-152.72
Ph
BF4
tBu
Ph
tBu
Ph
Ph
L10·HBF4
0 -10 -20 -30 -40 -50 -60 -70 -80 -90 -100 -110 -120 -130 -140 -150 -160 -170 -180 -190
f1 (ppm)

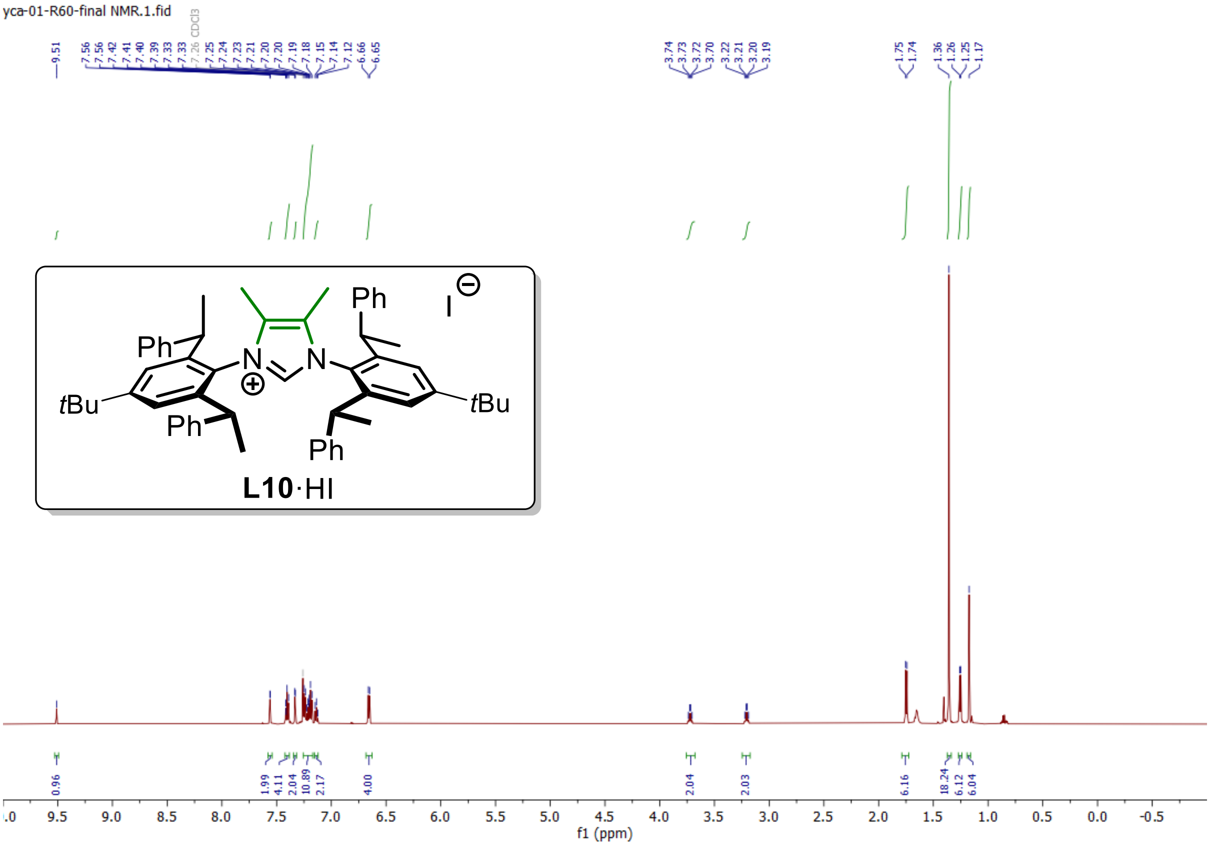

yca-01-R60-final NMR.1.fid
9.51
7.56 7.56 7.42 7.41 7.40 7.39 7.33 7.33 7.26 CDCl3 7.25 7.24 7.23 7.21 7.20 7.20 7.19 7.18 7.15 7.14 7.12 6.66 6.65
3.74 3.73 3.72 3.70 3.22 3.21 3.20 3.19
1.75 1.74 1.36 1.26 1.25 1.17
Ph
I
tBu
Ph
tBu
Ph
Ph
L10·HI
0.96 1.99 4.11 2.04 10.89 2.17 4.00 2.04 2.03 6.16 18.24 6.12 6.04
9.5 9.0 8.5 8.0 7.5 7.0 6.5 6.0 5.5 5.0 4.5 4.0 3.5 3.0 2.5 2.0 1.5 1.0 0.5 0.0 -0.5
f1 (ppm)

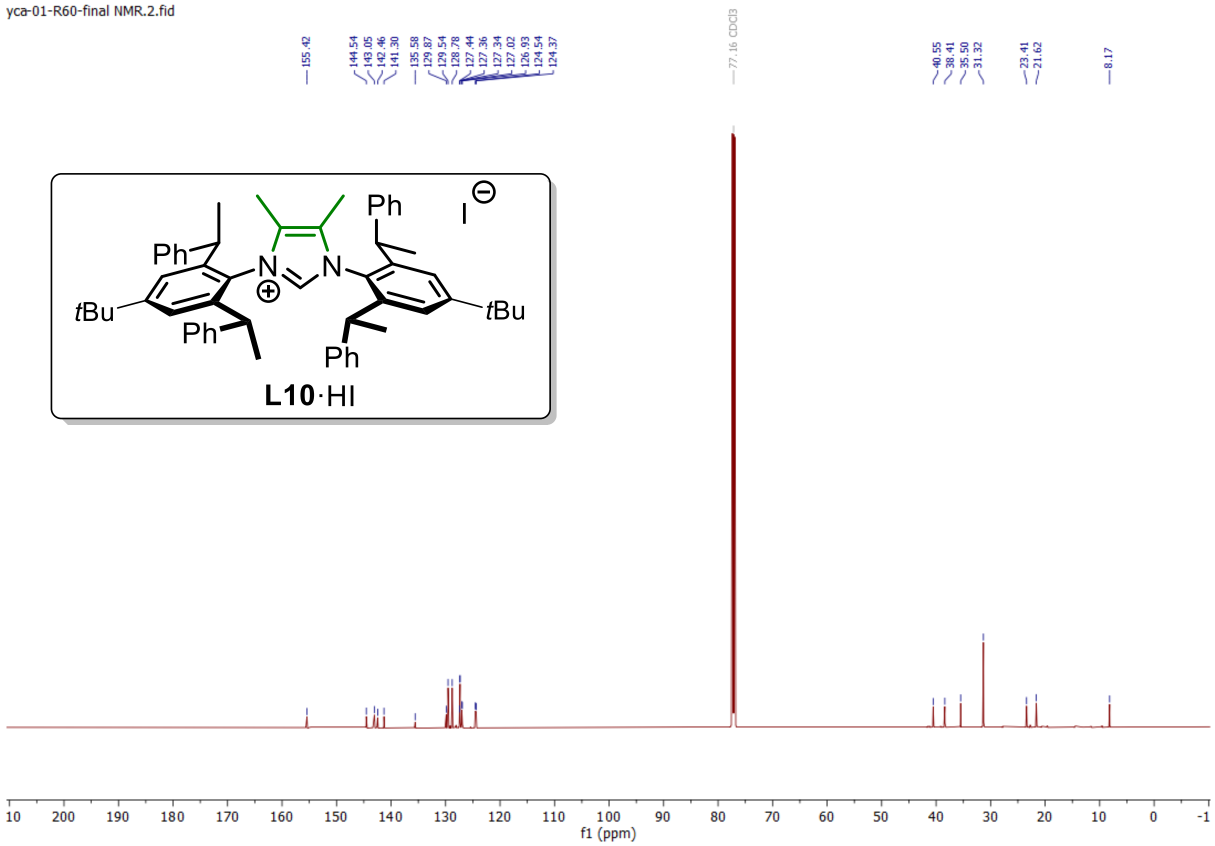
yca-01-R60-final NMR.2.fid
L10·HI
f1 (ppm)

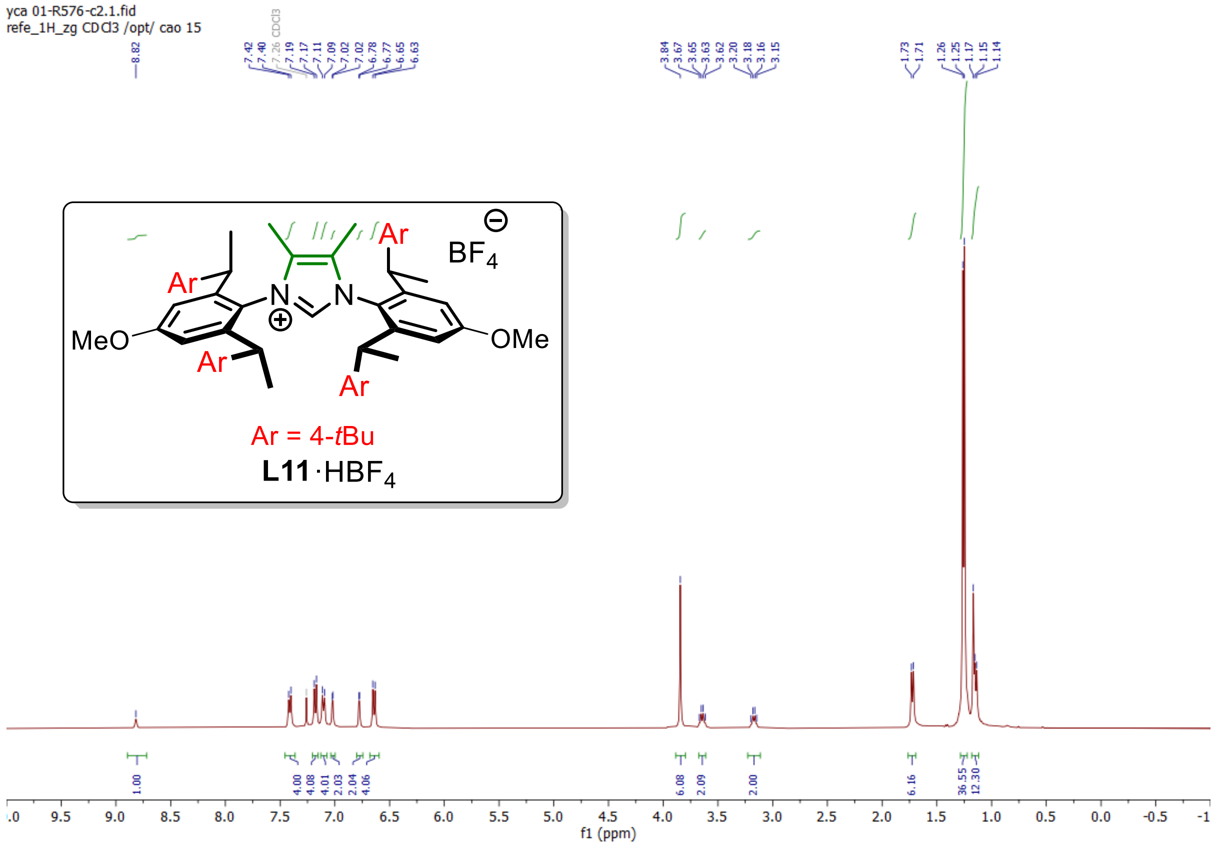
yca 01-R576-c2.1.fid
refe_1H_zg CDCl3 /opt/ cao 15
Ar = 4-tBu
L11·HBF4
f1 (ppm)

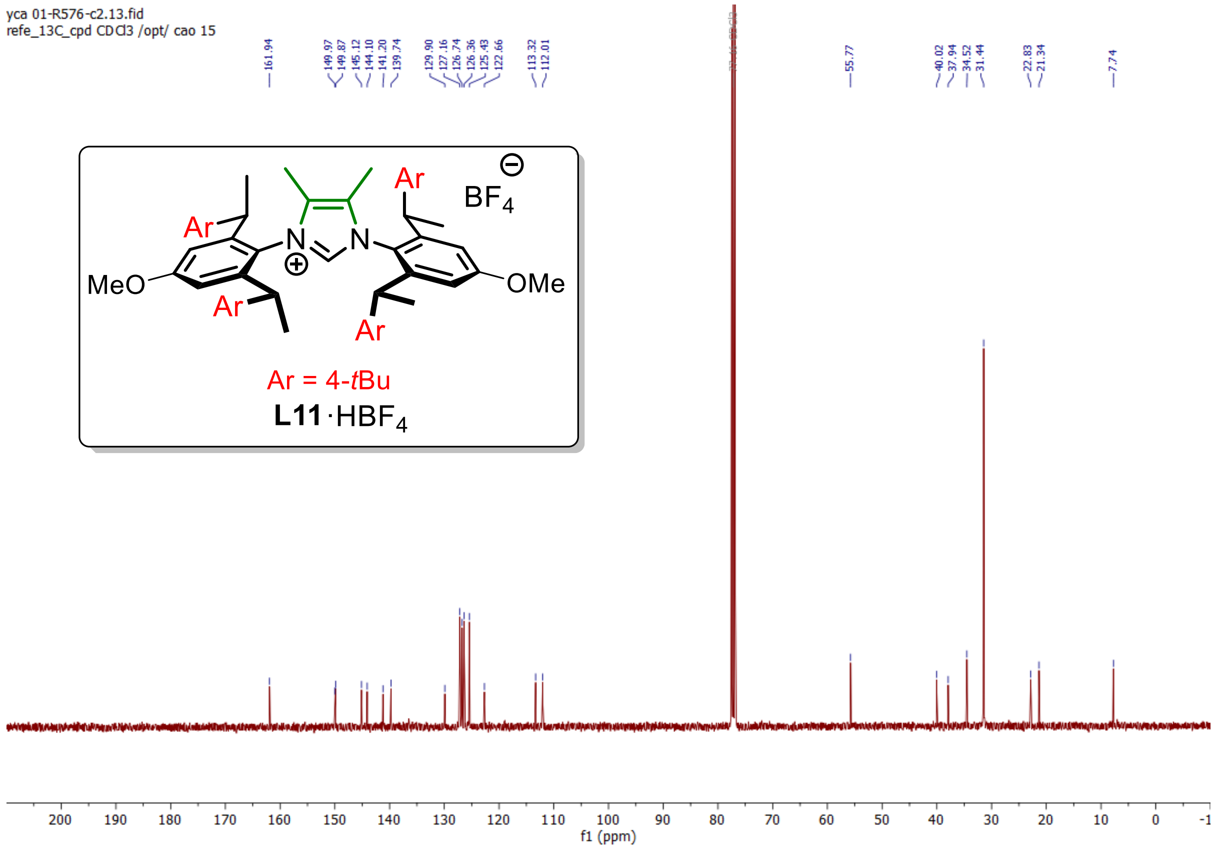
yca 01-R576-c2.13.fid
refe_13C_cpd CDCl3 /opt/ cao 15
161.94
149.97
149.87
145.12
144.10
141.20
139.74
129.90
127.16
126.74
126.36
125.43
122.66
113.32
112.01
55.77
40.02
37.94
34.52
31.44
22.83
21.34
7.74
Ar
BF4
MeO
OMe
N
N
Ar = 4-tBu
L11·HBF4
f1 (ppm)

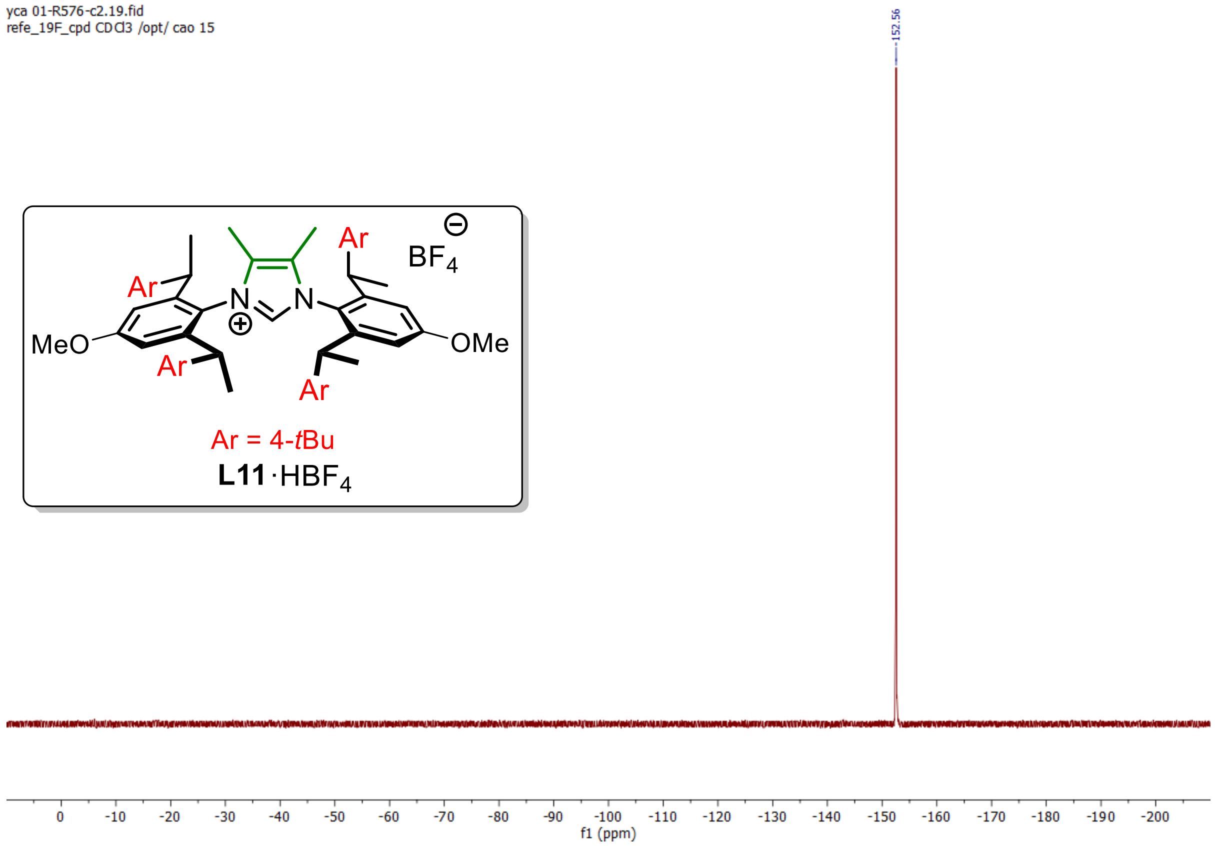
yca 01-R576-c2.19.fid
refe_19F_cpd CDCl3 /opt/ cao 15
-152.56
Ar
BF4
MeO
OMe
N
N
Ar = 4-tBu
L11·HBF4
f1 (ppm)

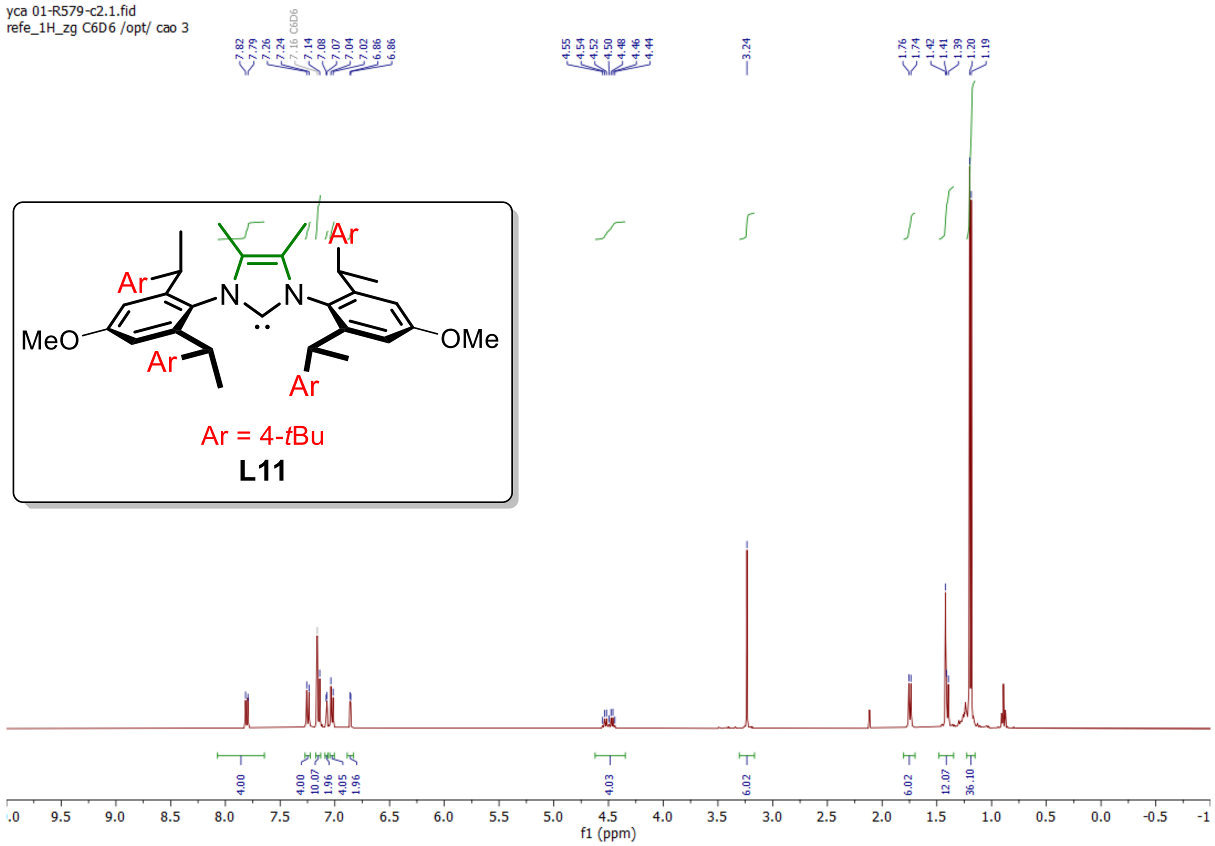
yca 01-R579-c2.1.fid
refe_1H_zg C6D6 /opt/ cao 3
MeO
OMe
Ar
Ar = 4-tBu
L11
f1 (ppm)

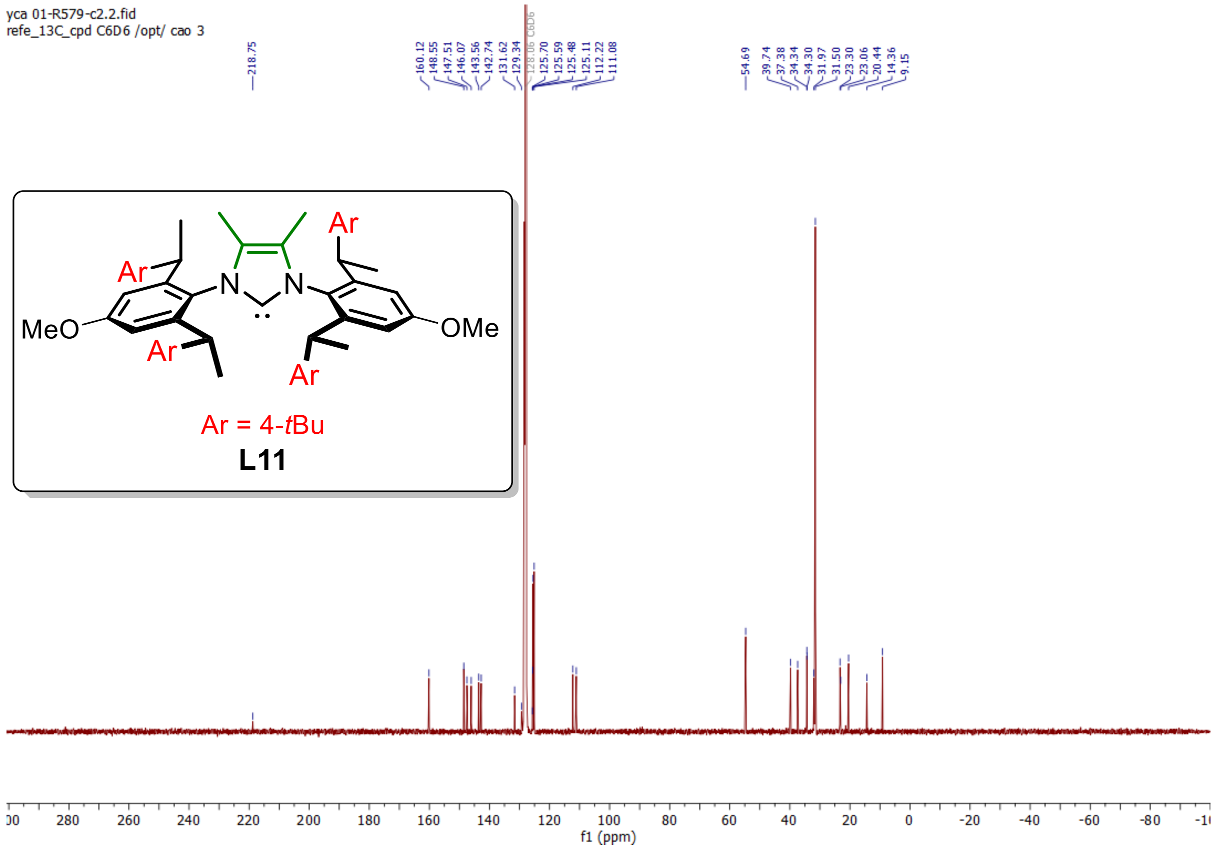
yca 01-R579-c2.2.fid
refe_13C_cpd C6D6 /opt/ cao 3
MeO
OMe
Ar
Ar = 4-tBu
L11
f1 (ppm)

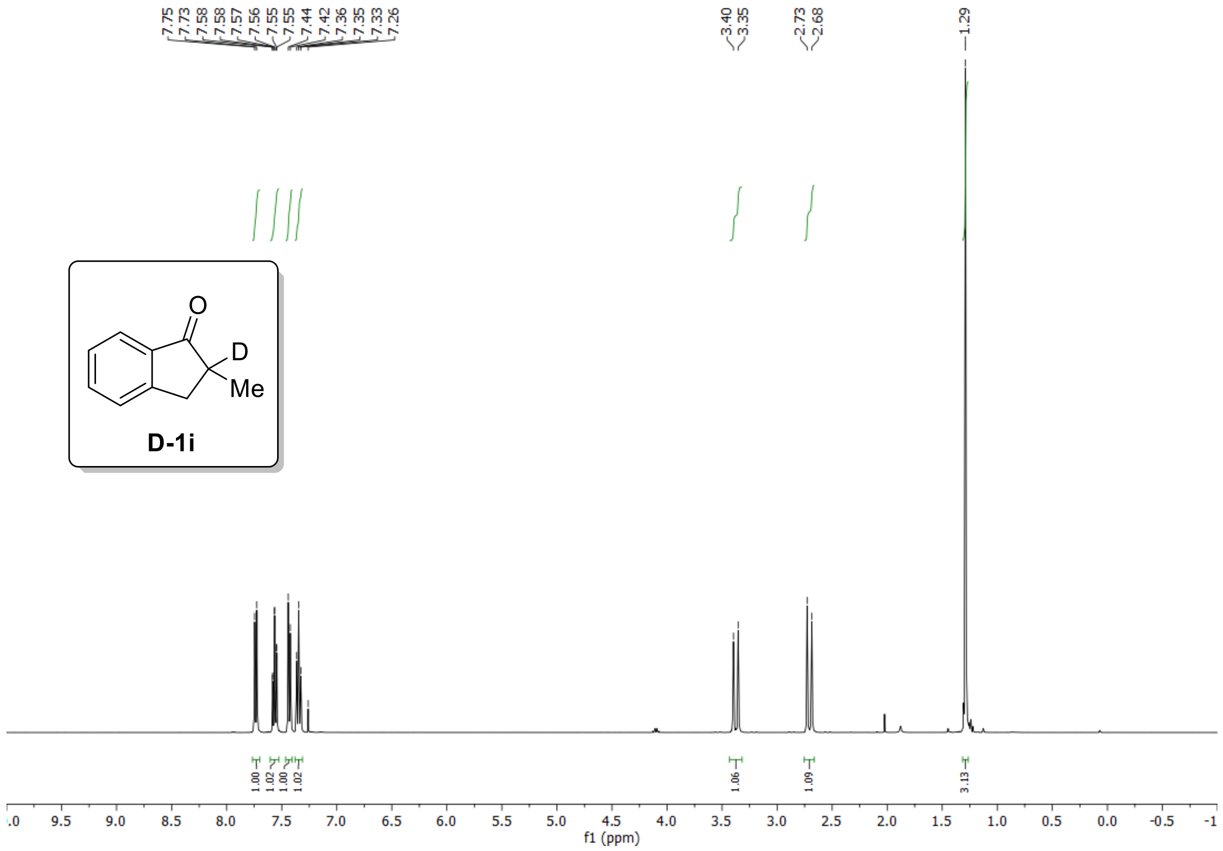
7.75
7.73
7.58
7.58
7.57
7.56
7.55
7.55
7.44
7.42
7.36
7.35
7.33
7.26
3.40
3.35
2.73
2.68
1.29
O
D
Me
D-1i
1.00
1.02
1.00
1.02
1.06
1.09
3.13
f1 (ppm)

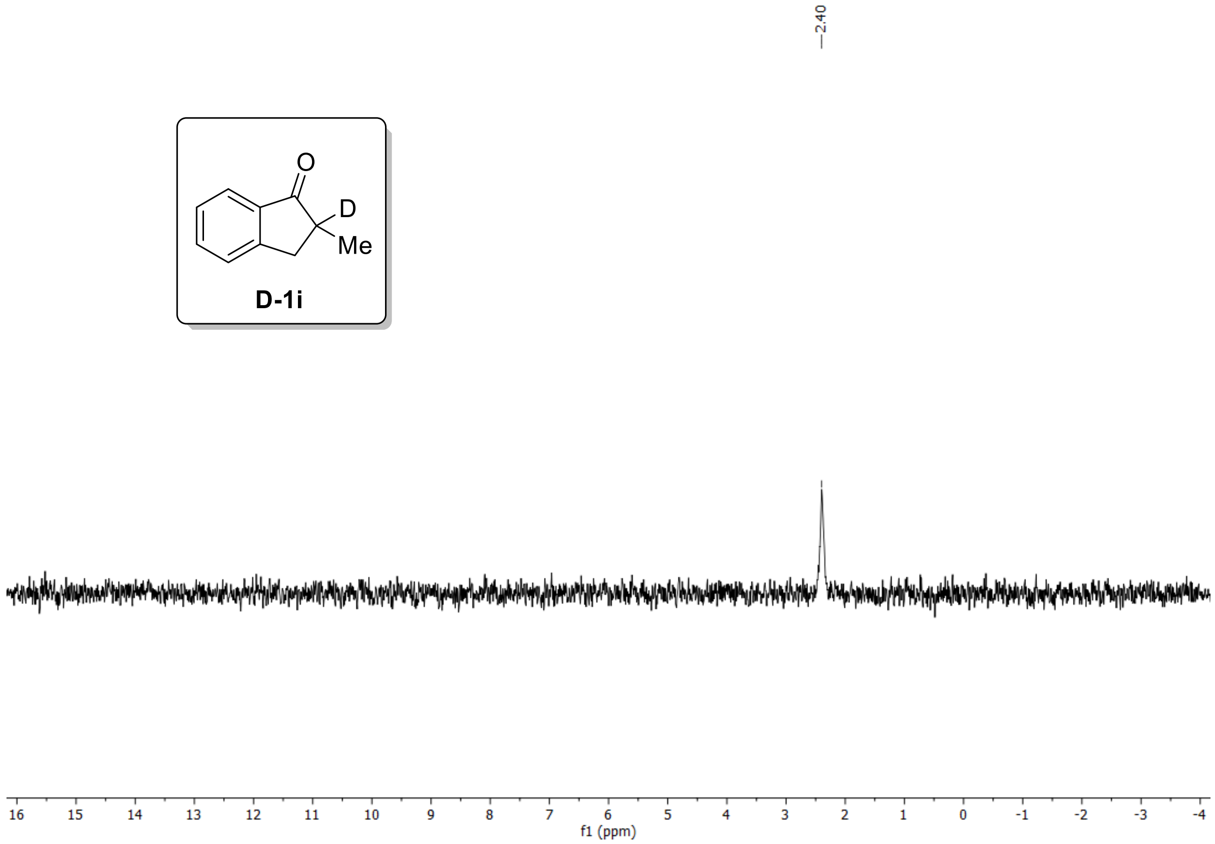
2.40
O
D
Me
D-1i
f1 (ppm)

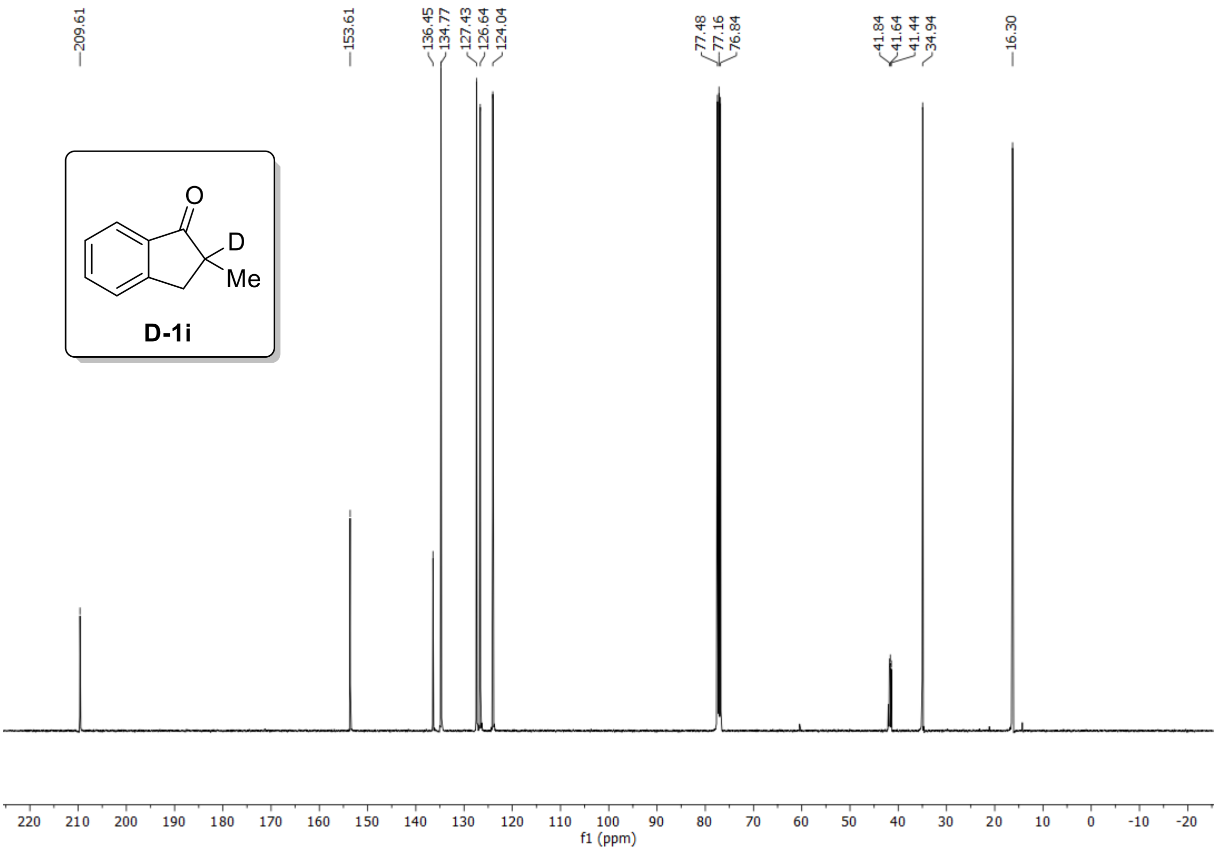
D
Me
O
D-1i
f1 (ppm)

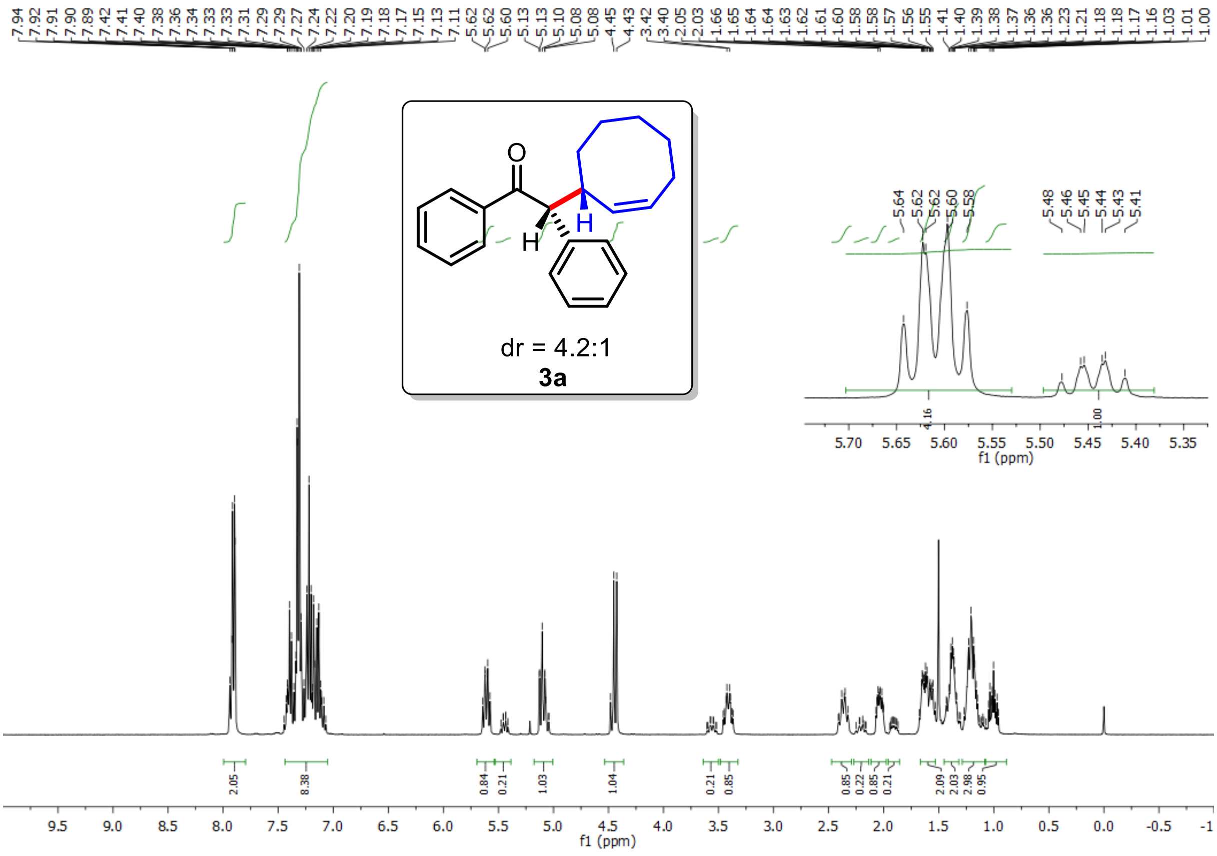
O
H
H
dr = 4.2:1
3a
f1 (ppm)

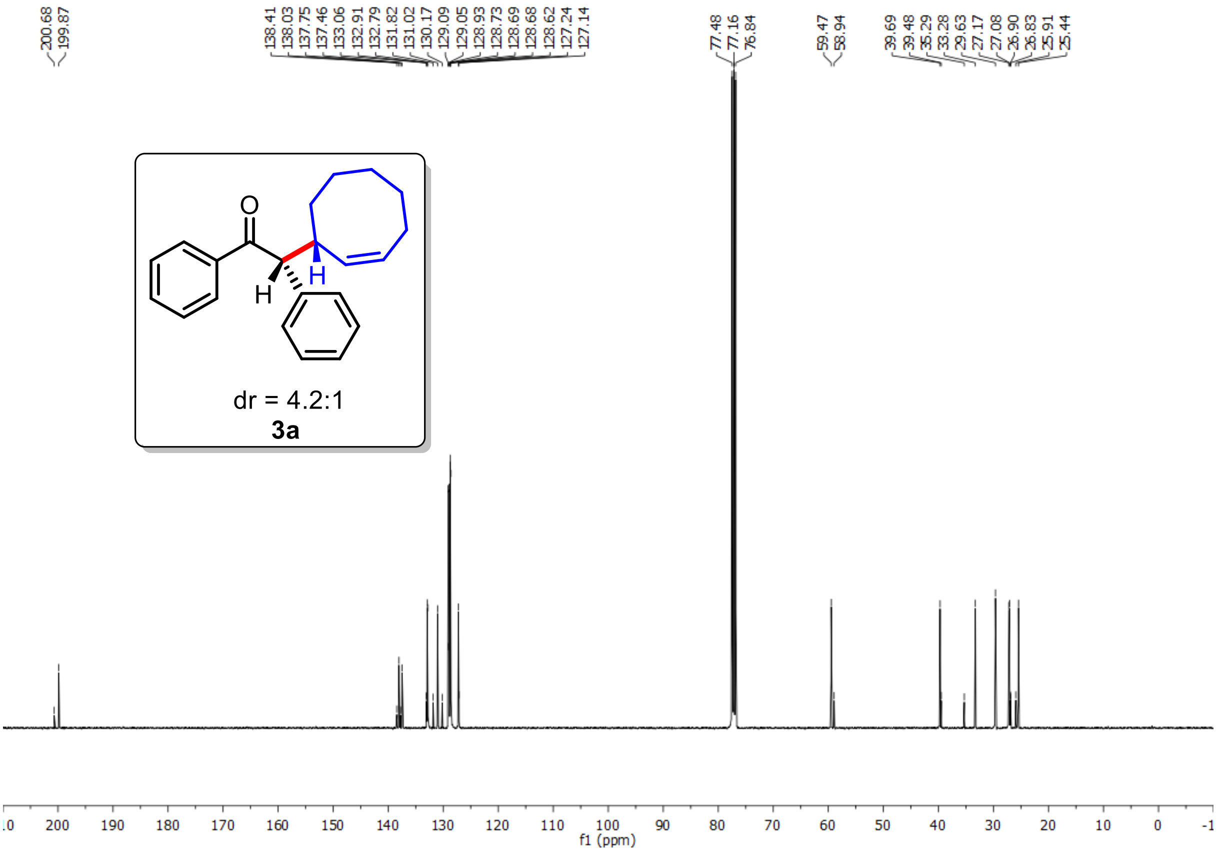
200.68
199.87
138.41
138.03
137.75
137.46
133.06
132.91
132.79
131.82
131.02
130.17
129.09
129.05
128.93
128.73
128.69
128.68
128.62
127.24
127.14
77.48
77.16
76.84
59.47
58.94
39.69
39.48
35.29
33.28
29.63
27.17
27.08
26.90
26.83
25.91
25.44
O
H
H
dr = 4.2:1
3a
f1 (ppm)

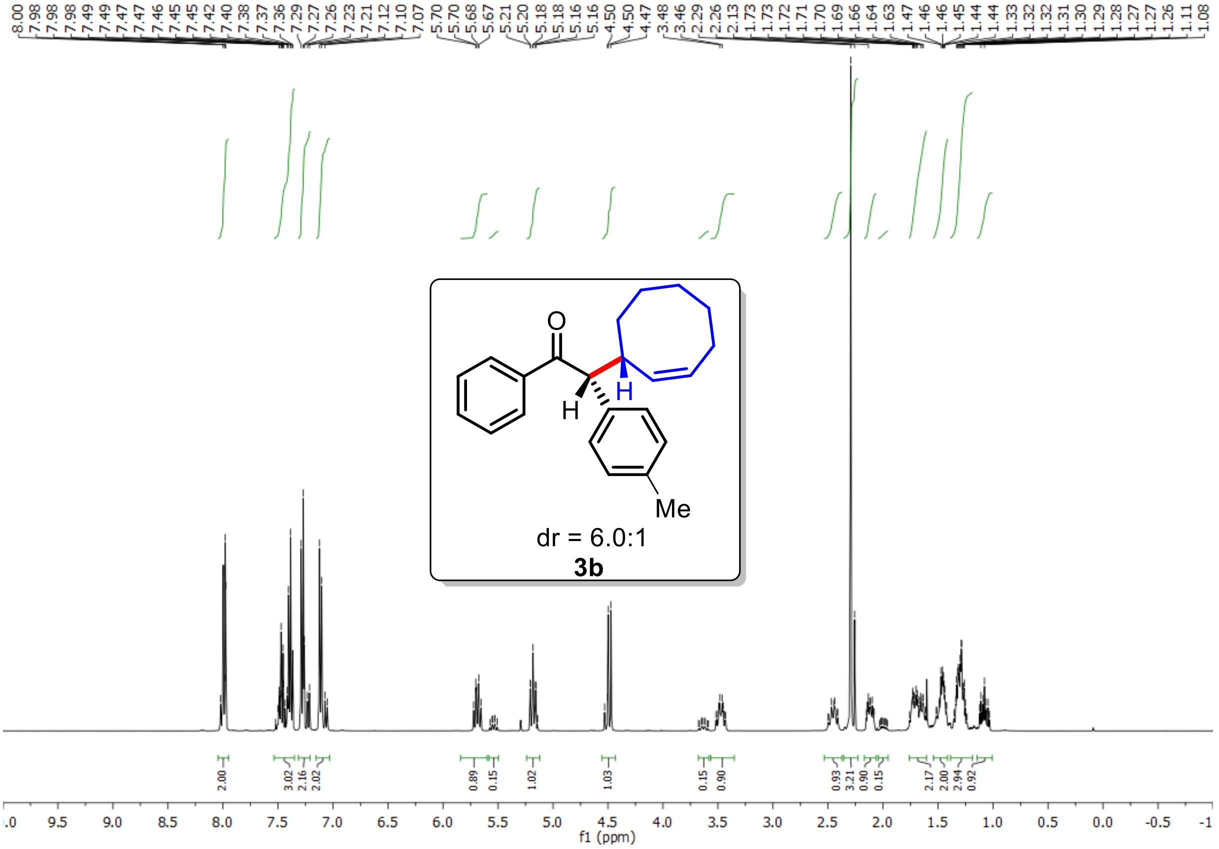
8.00
7.98
7.98
7.49
7.49
7.47
7.47
7.46
7.45
7.42
7.40
7.38
7.37
7.36
7.29
7.27
7.26
7.23
7.21
7.12
7.10
7.07
5.70
5.70
5.68
5.67
5.21
5.20
5.18
5.18
5.16
5.16
4.50
4.50
4.47
3.48
3.46
2.29
2.26
2.13
1.73
1.73
1.72
1.71
1.70
1.69
1.66
1.64
1.63
1.47
1.46
1.46
1.45
1.44
1.44
1.33
1.32
1.32
1.31
1.30
1.29
1.28
1.27
1.27
1.26
1.11
1.08
O
H
H
Me
dr = 6.0:1
3b
2.00
3.02
2.16
2.02
0.89
0.15
1.02
1.03
0.15
0.90
0.93
3.21
0.90
0.15
2.17
2.00
2.94
0.92
f1 (ppm)

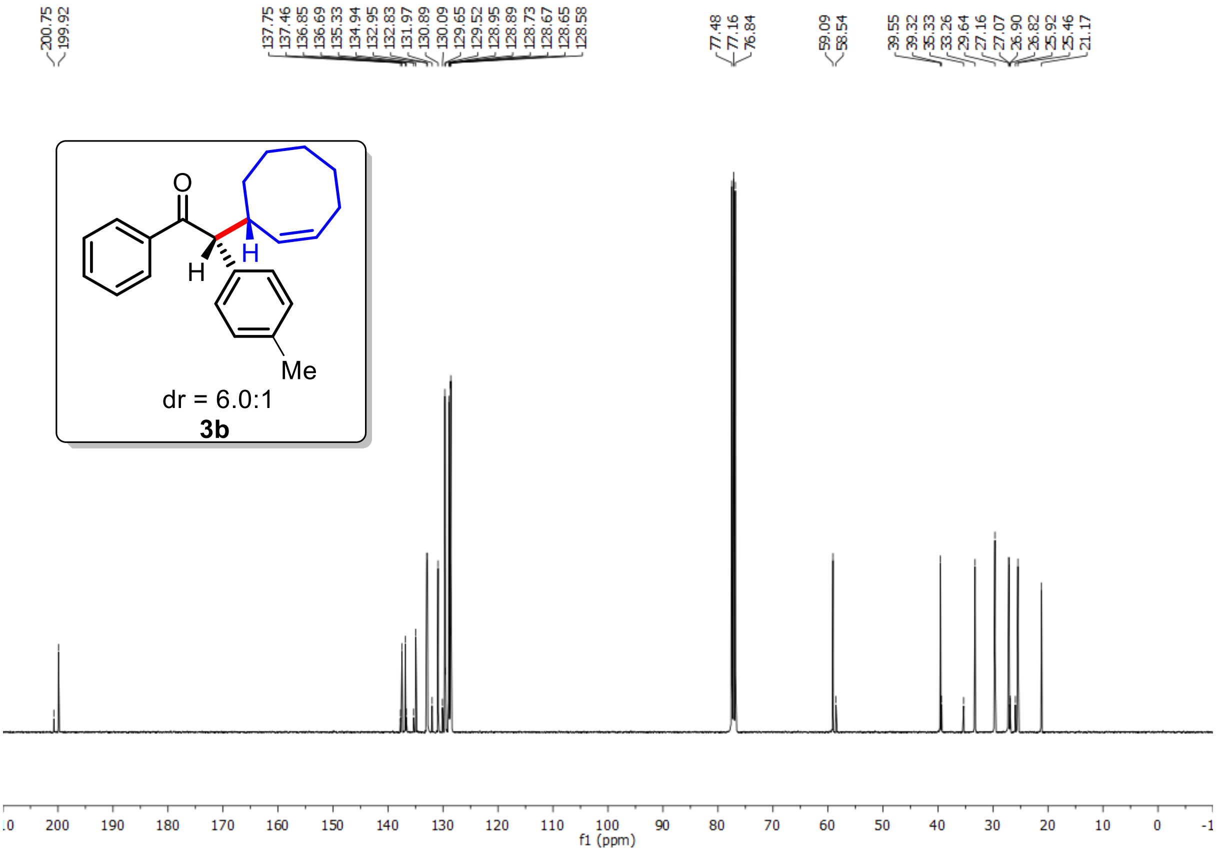
200.75
199.92
137.75
137.46
136.85
136.69
135.33
134.94
132.95
132.83
131.97
130.89
130.09
129.65
129.52
128.95
128.89
128.73
128.67
128.65
128.58
77.48
77.16
76.84
59.09
58.54
39.55
39.32
35.33
33.26
29.64
27.16
27.07
26.90
26.82
25.92
25.46
21.17
O
H
H
Me
dr = 6.0:1
3b
f1 (ppm)

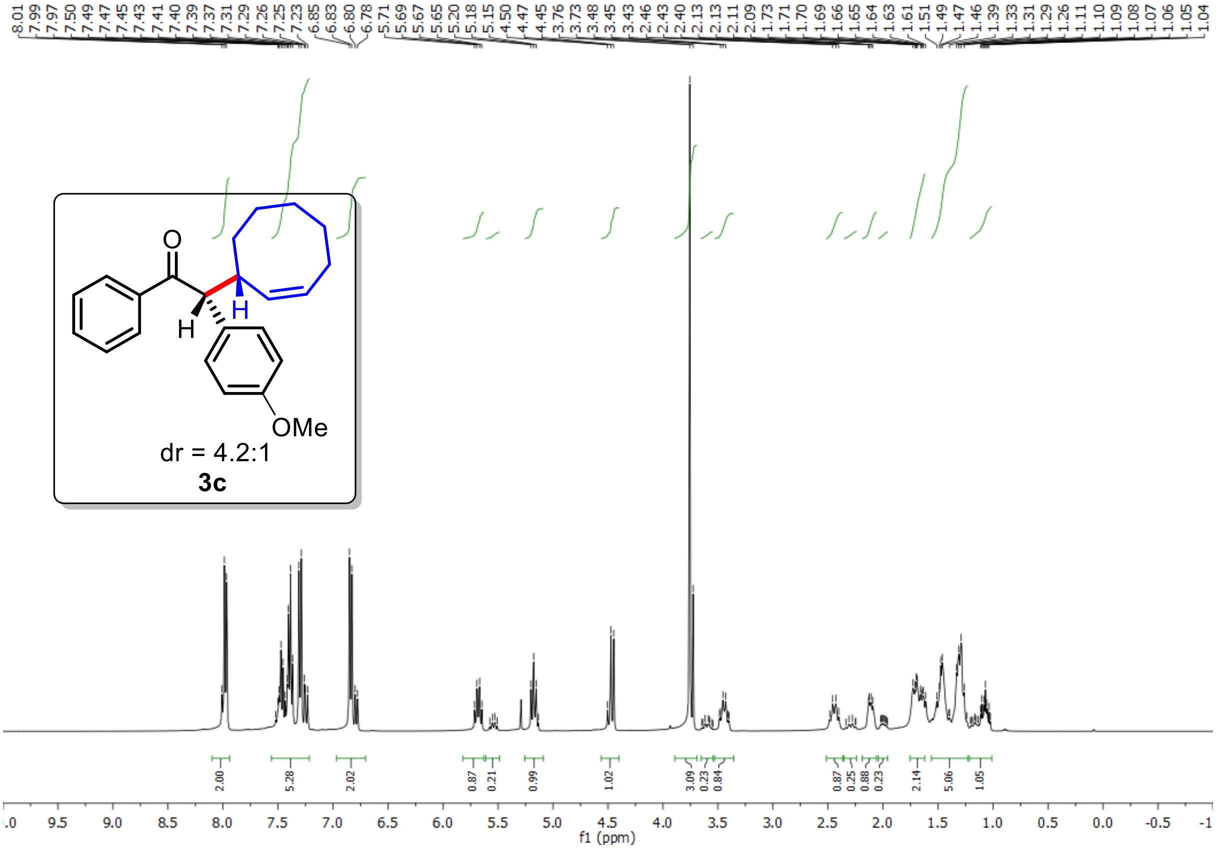
8.01
7.99
7.97
7.50
7.49
7.47
7.45
7.43
7.41
7.40
7.39
7.37
7.31
7.29
7.26
7.25
7.23
6.85
6.83
6.80
6.78
5.71
5.69
5.67
5.65
5.20
5.18
5.15
4.50
4.47
4.45
3.76
3.73
3.48
3.45
3.43
2.46
2.43
2.40
2.13
2.13
2.11
2.09
1.73
1.71
1.70
1.69
1.66
1.65
1.64
1.63
1.61
1.51
1.49
1.47
1.46
1.39
1.33
1.31
1.29
1.26
1.11
1.10
1.09
1.08
1.07
1.06
1.05
1.04
O
H
H
OMe
dr = 4.2:1
3c
2.00
5.28
2.02
0.87
0.21
0.99
1.02
3.09
0.23
0.84
0.87
0.25
0.88
0.23
2.14
5.06
1.05
f1 (ppm)

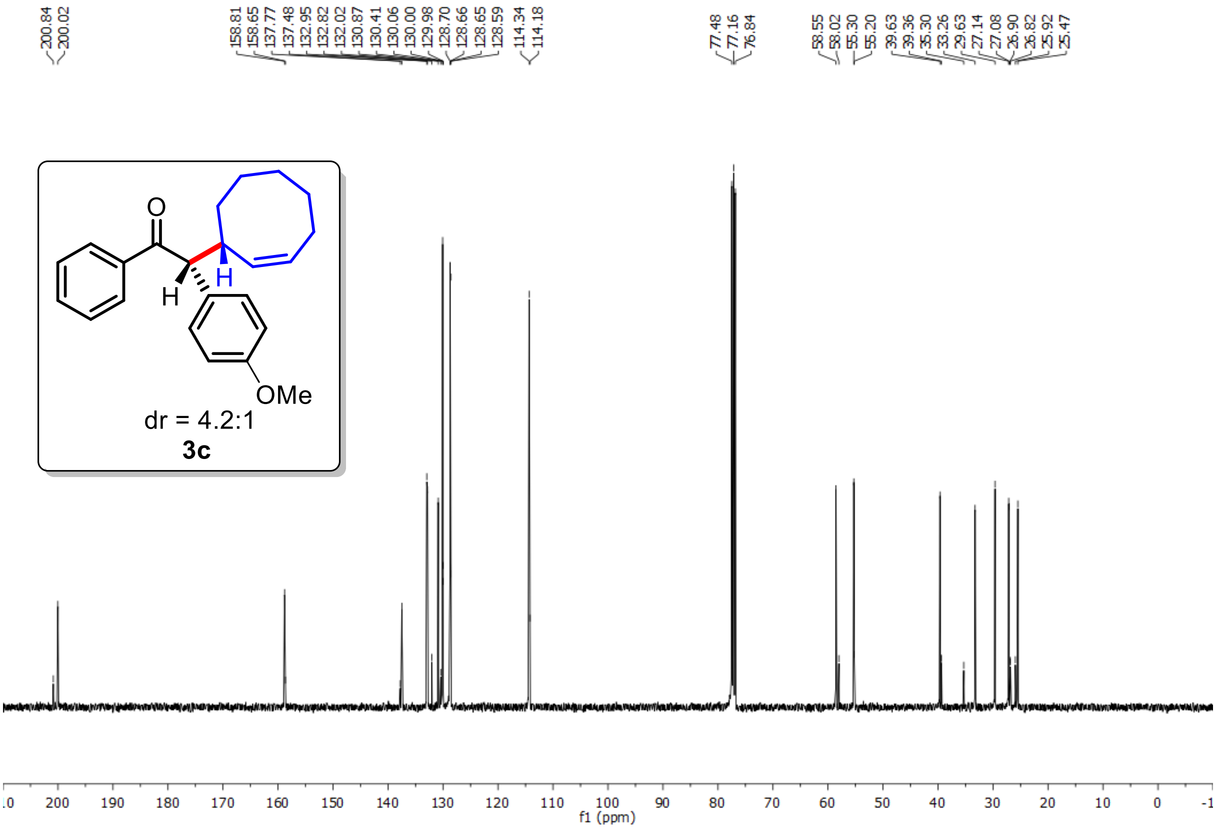

O
H
H
OMe
dr = 4.2:1
3c
f1 (ppm)

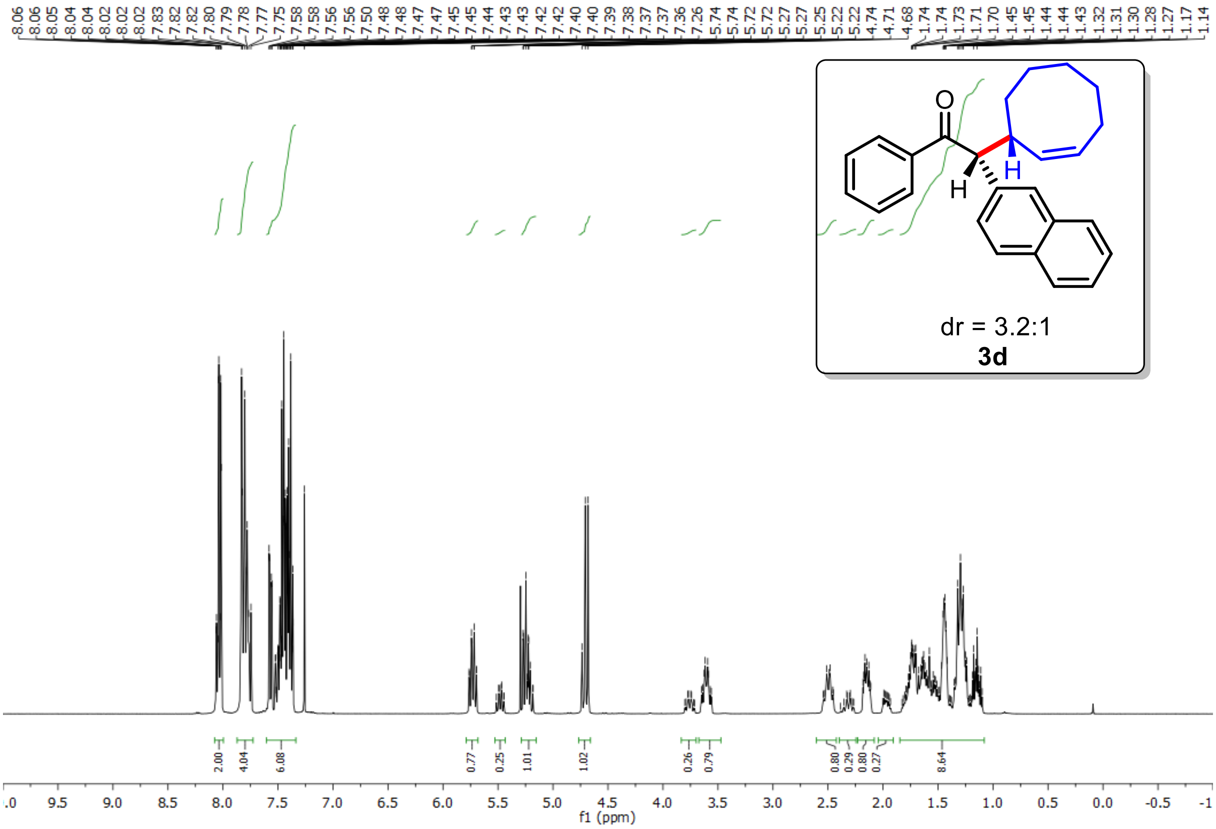

O
H
H
dr = 3.2:1
3d
f1 (ppm)

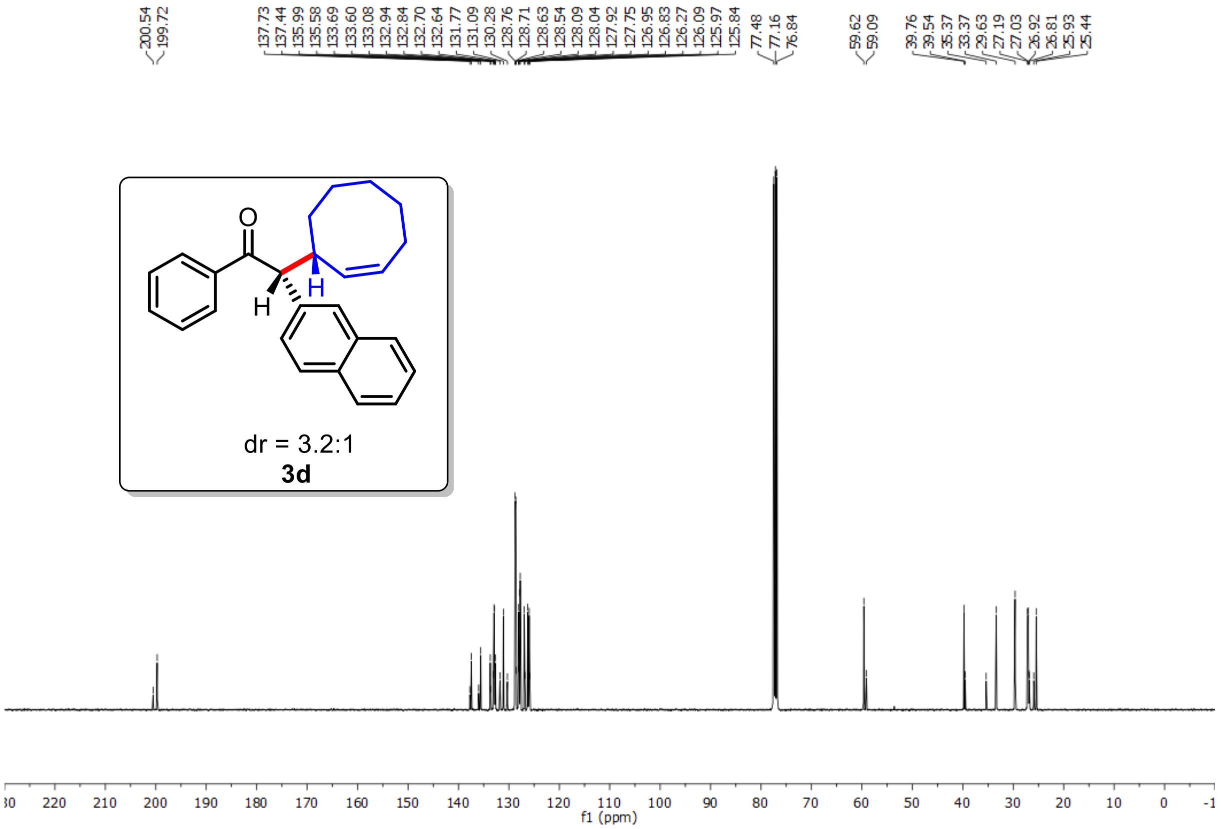
200.54
199.72
137.73
137.44
135.99
135.58
133.69
133.60
133.08
132.94
132.84
132.70
132.64
131.77
131.09
130.28
128.76
128.71
128.63
128.54
128.09
128.04
127.92
127.75
126.95
126.83
126.27
126.09
125.97
125.84
77.48
77.16
76.84
59.62
59.09
39.76
39.54
35.37
33.37
29.63
27.19
27.03
26.92
26.81
25.93
25.44
O
H
H
dr = 3.2:1
3d
f1 (ppm)

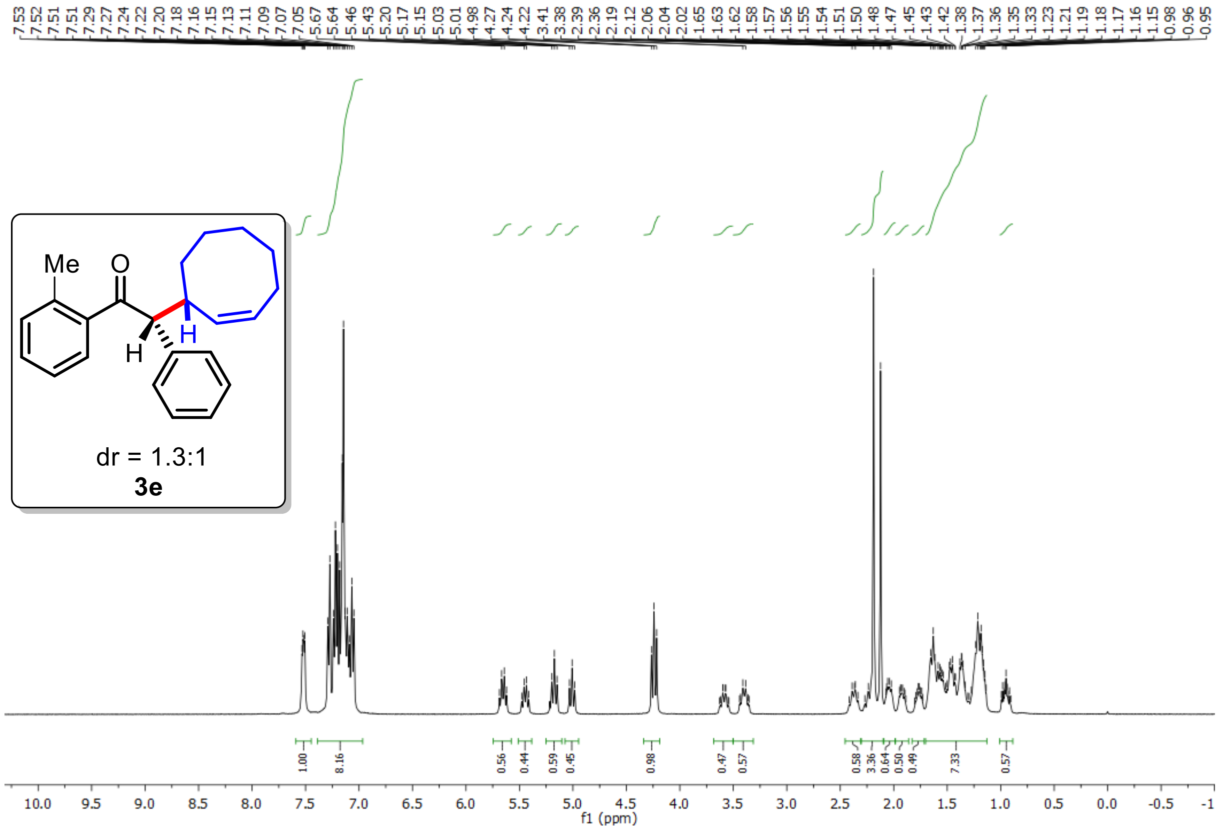
7.53
7.52
7.51
7.51
7.29
7.27
7.24
7.22
7.20
7.18
7.16
7.15
7.13
7.11
7.09
7.07
7.05
5.67
5.64
5.46
5.43
5.20
5.17
5.15
5.03
5.01
4.98
4.27
4.24
4.22
3.41
3.38
2.39
2.36
2.19
2.12
2.06
2.04
2.02
1.65
1.63
1.62
1.58
1.57
1.56
1.55
1.54
1.51
1.50
1.48
1.47
1.45
1.43
1.42
1.38
1.37
1.36
1.35
1.33
1.23
1.21
1.19
1.18
1.17
1.16
1.15
0.98
0.96
0.95
Me
O
H
H
dr = 1.3:1
3e
1.00
8.16
0.56
0.44
0.59
0.45
0.98
0.47
0.57
0.58
3.36
0.64
0.50
0.49
7.33
0.57
f1 (ppm)

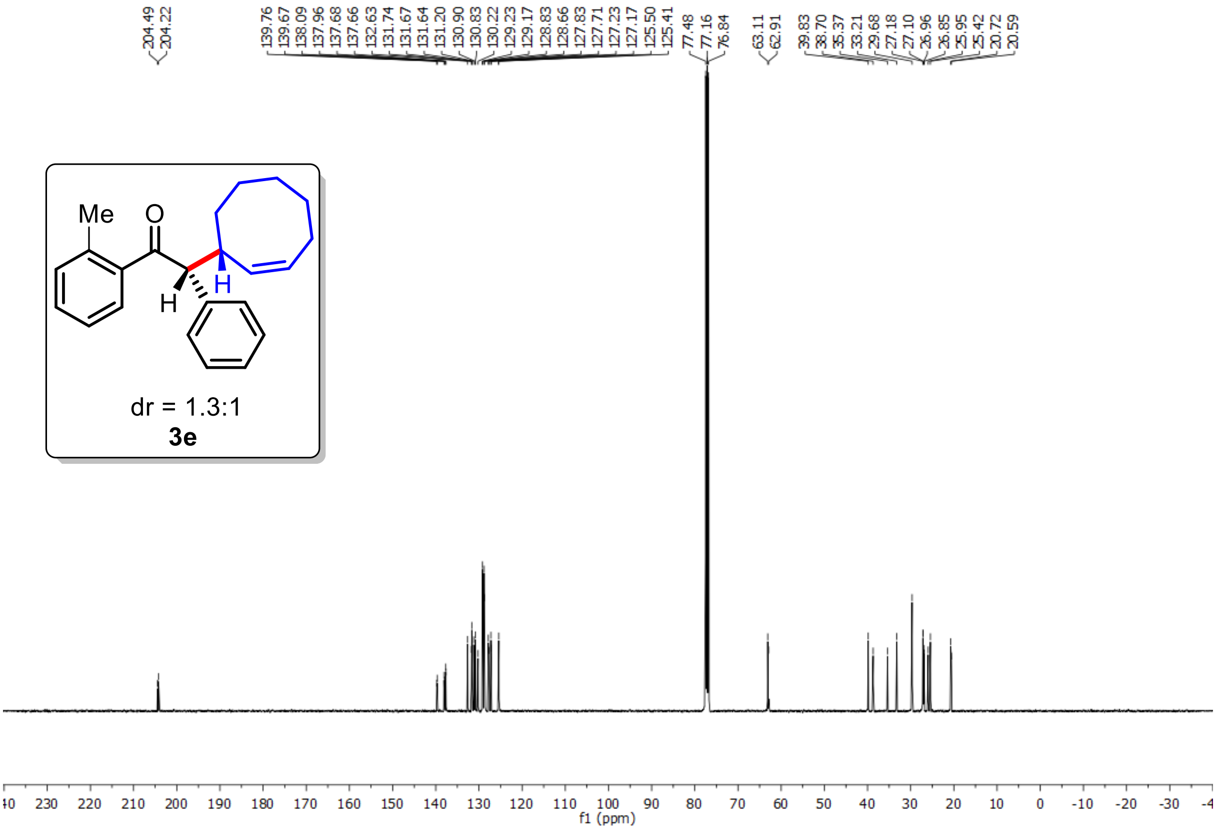
Me
O
H
H
dr = 1.3:1
3e
f1 (ppm)

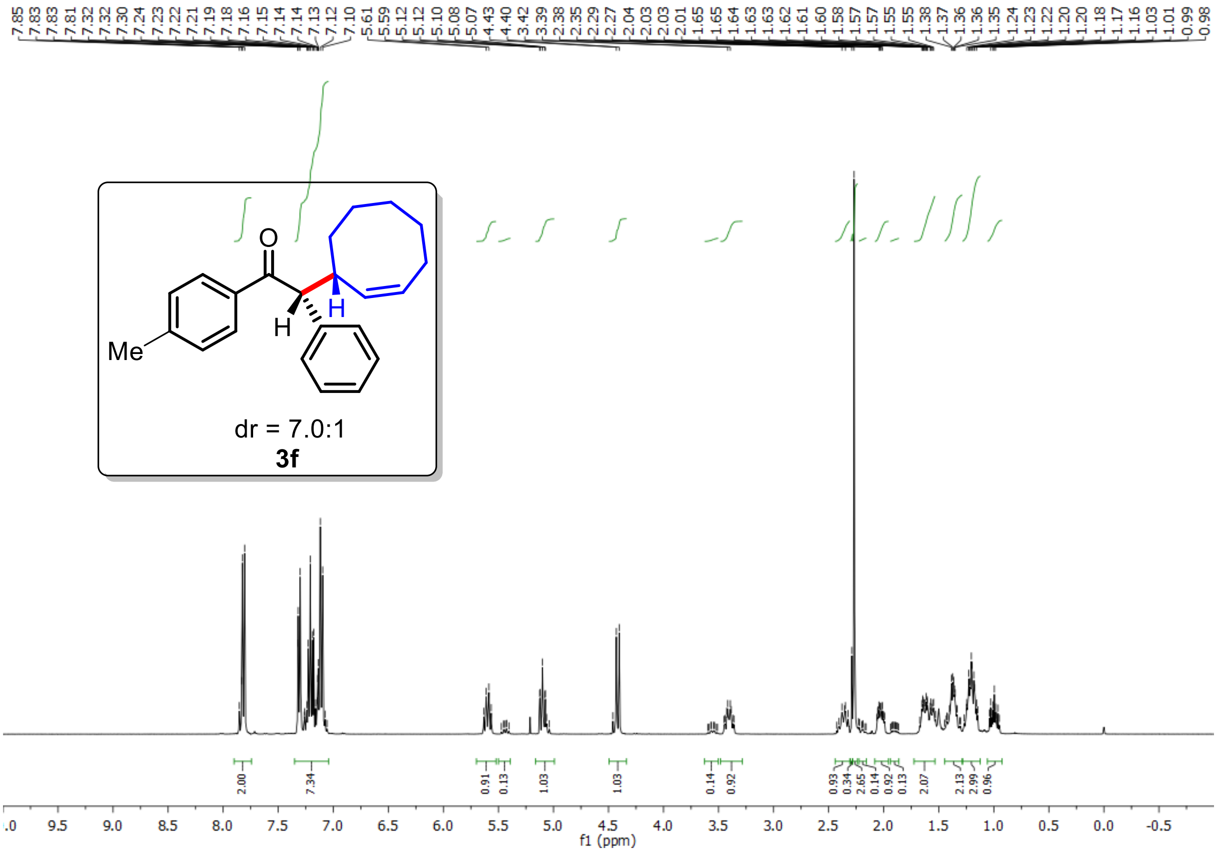
O
H
H
Me
dr = 7.0:1
3f
f1 (ppm)

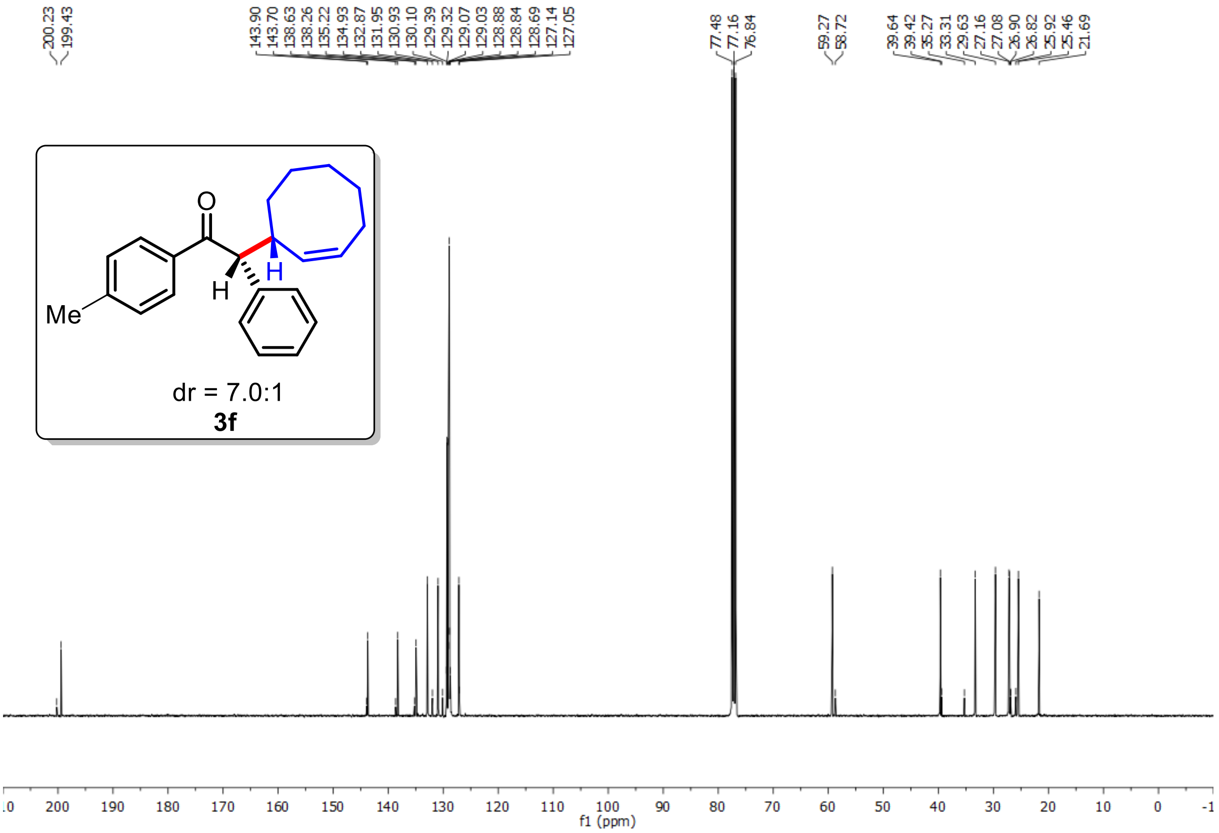
200.23
199.43
143.90
143.70
138.63
138.26
135.22
134.93
132.87
131.95
130.93
130.10
129.39
129.32
129.07
129.03
128.88
128.84
128.69
127.14
127.05
77.48
77.16
76.84
59.27
58.72
39.64
39.42
35.27
33.31
29.63
27.16
27.08
26.90
26.82
25.92
25.46
21.69
O
H
H
Me
dr = 7.0:1
3f
f1 (ppm)

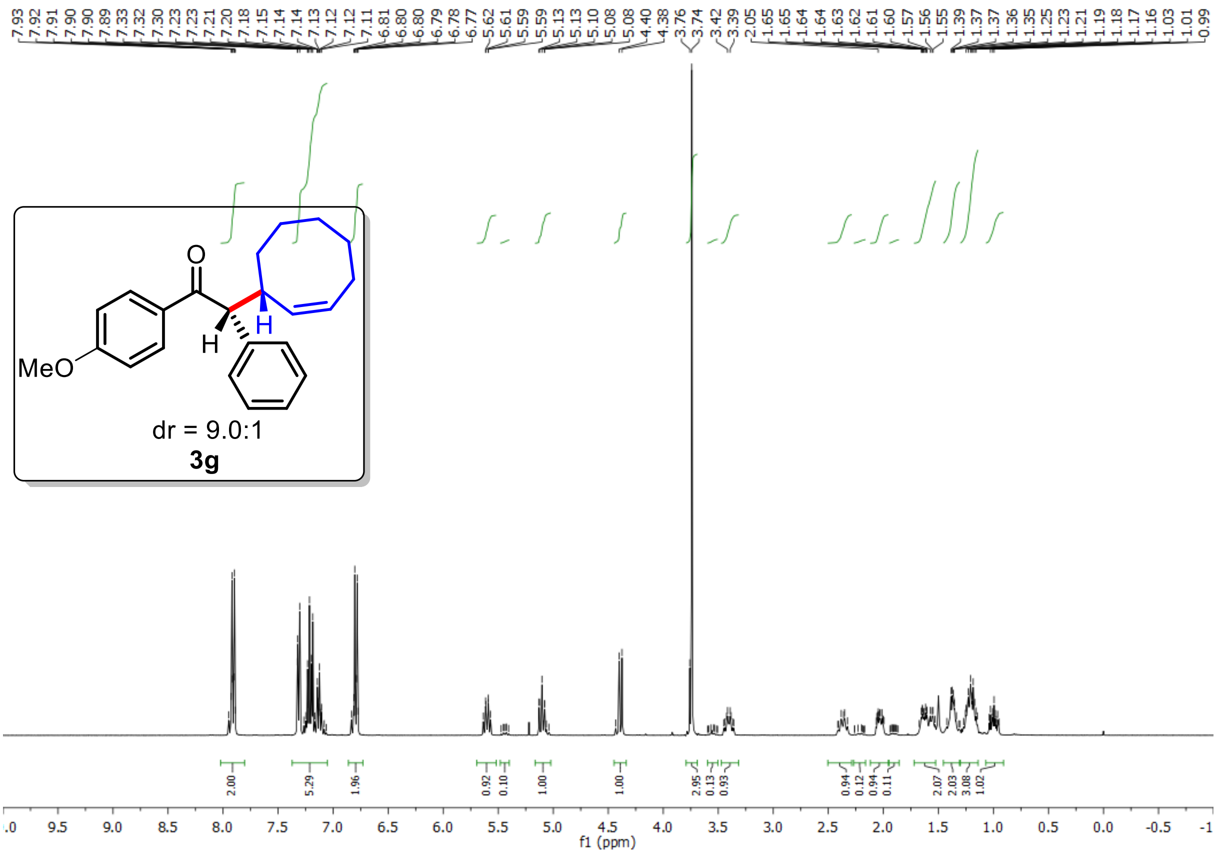
7.93
7.92
7.91
7.90
7.90
7.89
7.33
7.32
7.30
7.23
7.23
7.21
7.20
7.18
7.15
7.14
7.13
7.12
7.11
6.81
6.80
6.80
6.79
6.78
6.77
5.62
5.61
5.59
5.59
5.13
5.13
5.10
5.08
4.40
4.38
3.76
3.74
3.42
3.39
2.05
1.65
1.65
1.64
1.64
1.63
1.62
1.61
1.60
1.57
1.56
1.55
1.39
1.37
1.37
1.36
1.35
1.25
1.23
1.21
1.19
1.18
1.17
1.16
1.03
1.01
0.99
O
H
H
MeO
dr = 9.0:1
3g
2.00
5.29
1.96
0.92
0.10
1.00
1.00
2.95
0.13
0.93
0.94
0.12
0.94
0.11
2.07
2.03
3.08
1.02
f1 (ppm)

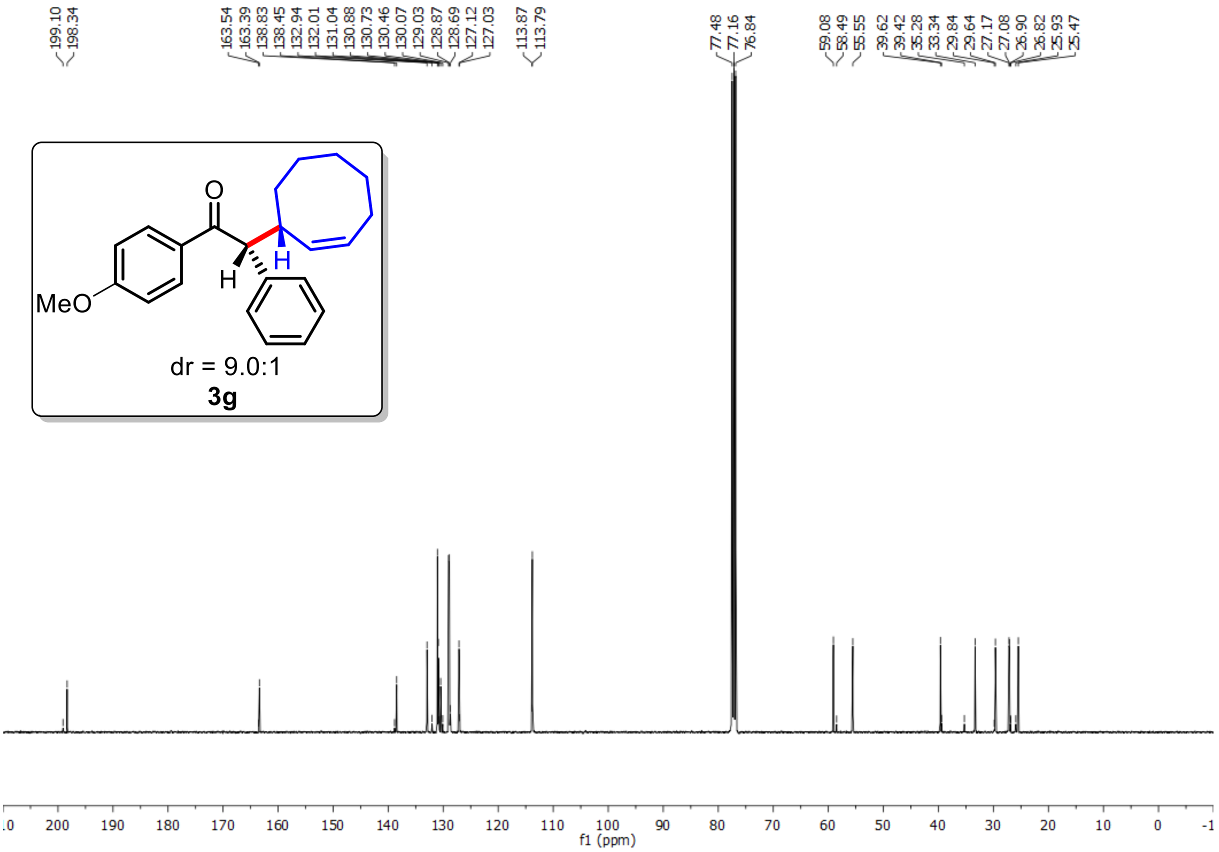
199.10
198.34
163.54
163.39
138.83
138.45
132.94
132.01
131.04
130.88
130.73
130.46
130.07
129.03
128.87
128.69
127.12
127.03
113.87
113.79
77.48
77.16
76.84
59.08
58.49
55.55
39.62
39.42
35.28
33.34
29.84
29.64
27.17
27.08
26.90
26.82
25.93
25.47
O
H
H
MeO
dr = 9.0:1
3g
f1 (ppm)

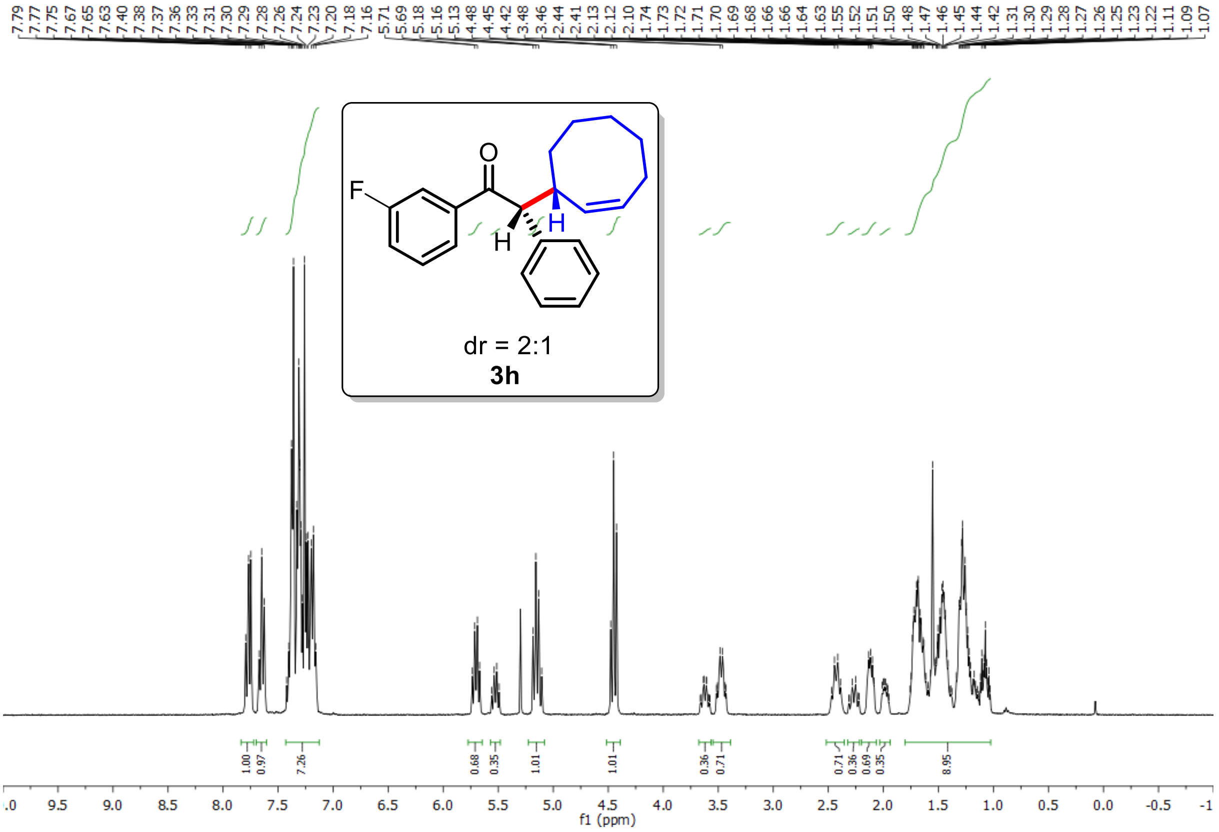
O
F
H
H
dr = 2:1
3h
f1 (ppm)

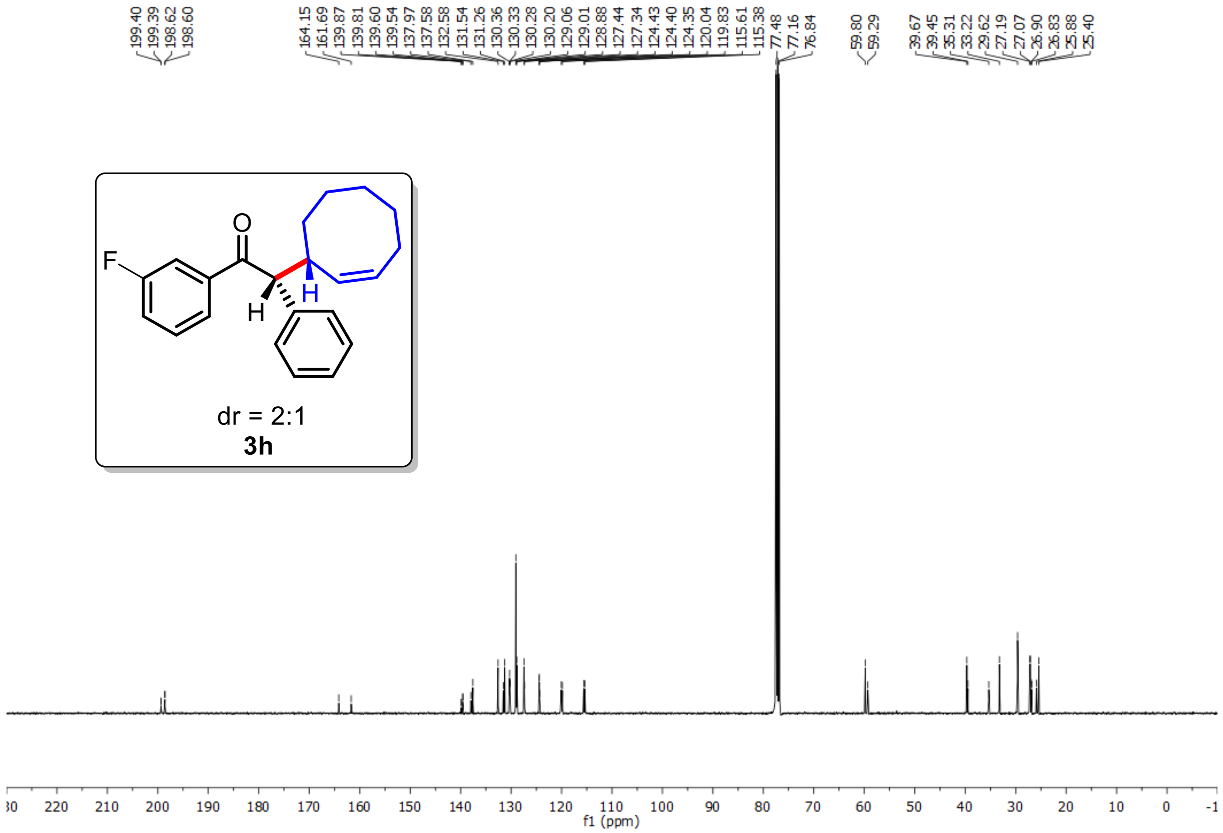
199.40
199.39
198.62
198.60
164.15
161.69
139.87
139.81
139.60
139.54
137.97
137.58
132.58
131.54
131.26
130.36
130.33
130.28
130.20
129.06
129.01
128.88
127.44
127.34
124.43
124.40
124.35
120.04
119.83
115.61
115.38
77.48
77.16
76.84
59.80
59.29
39.67
39.45
35.31
33.22
29.62
27.19
27.07
26.90
26.83
25.88
25.40
F
O
H
H
dr = 2:1
3h
f1 (ppm)

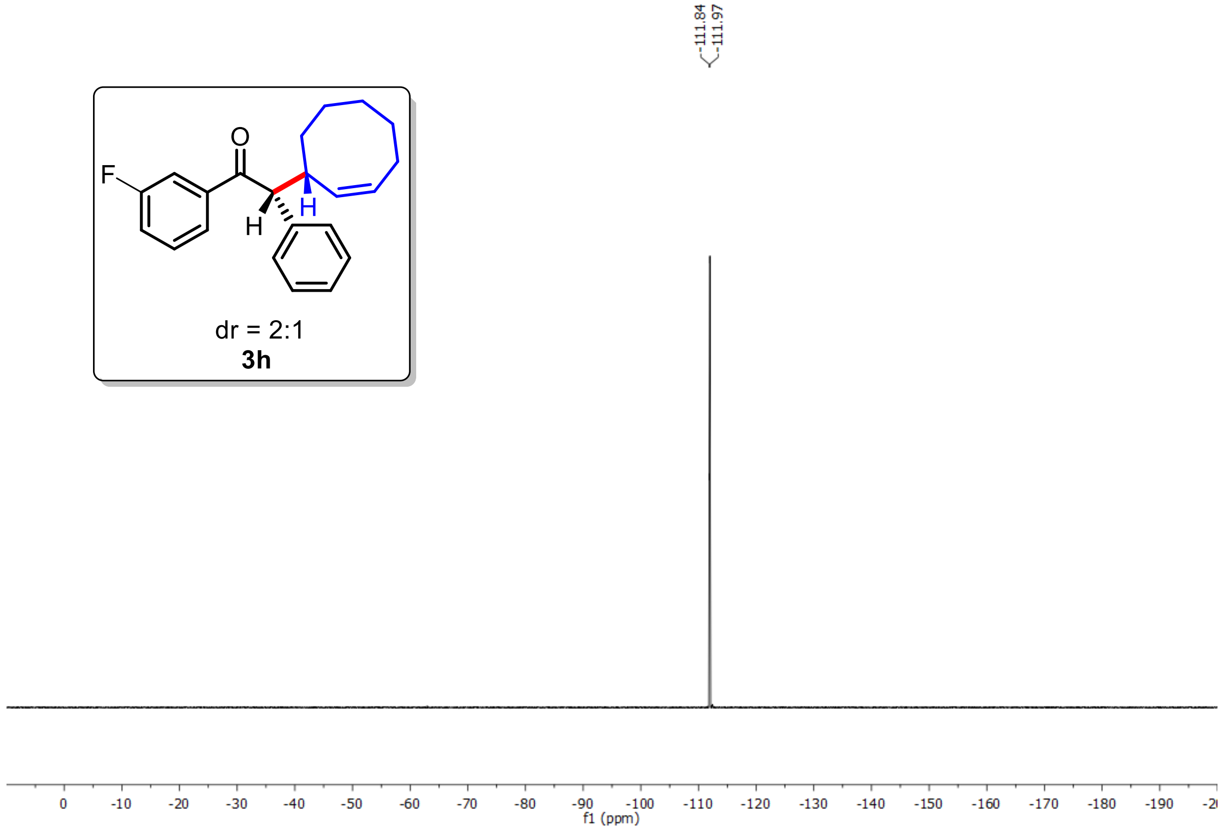
-111.84
-111.97
F
O
H
H
dr = 2:1
3h
f1 (ppm)

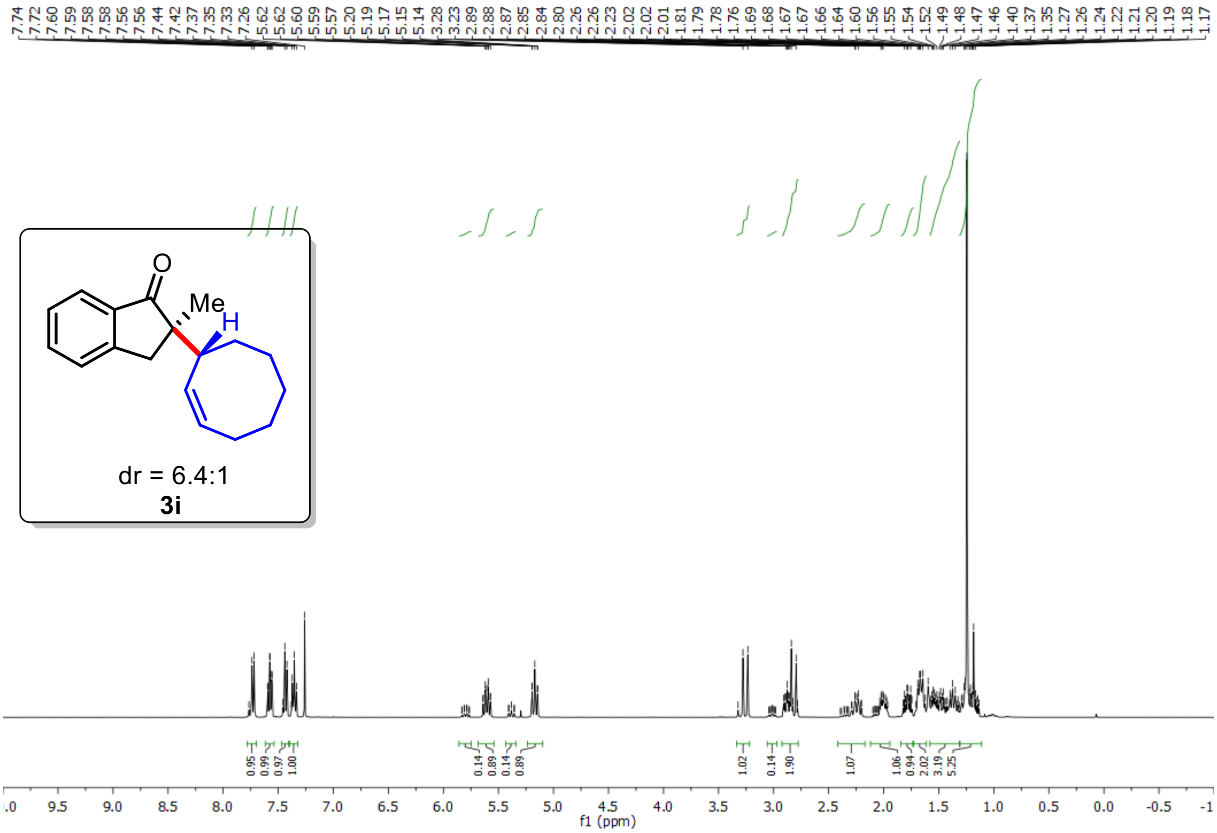
Me
H
dr = 6.4:1
3i
f1 (ppm)

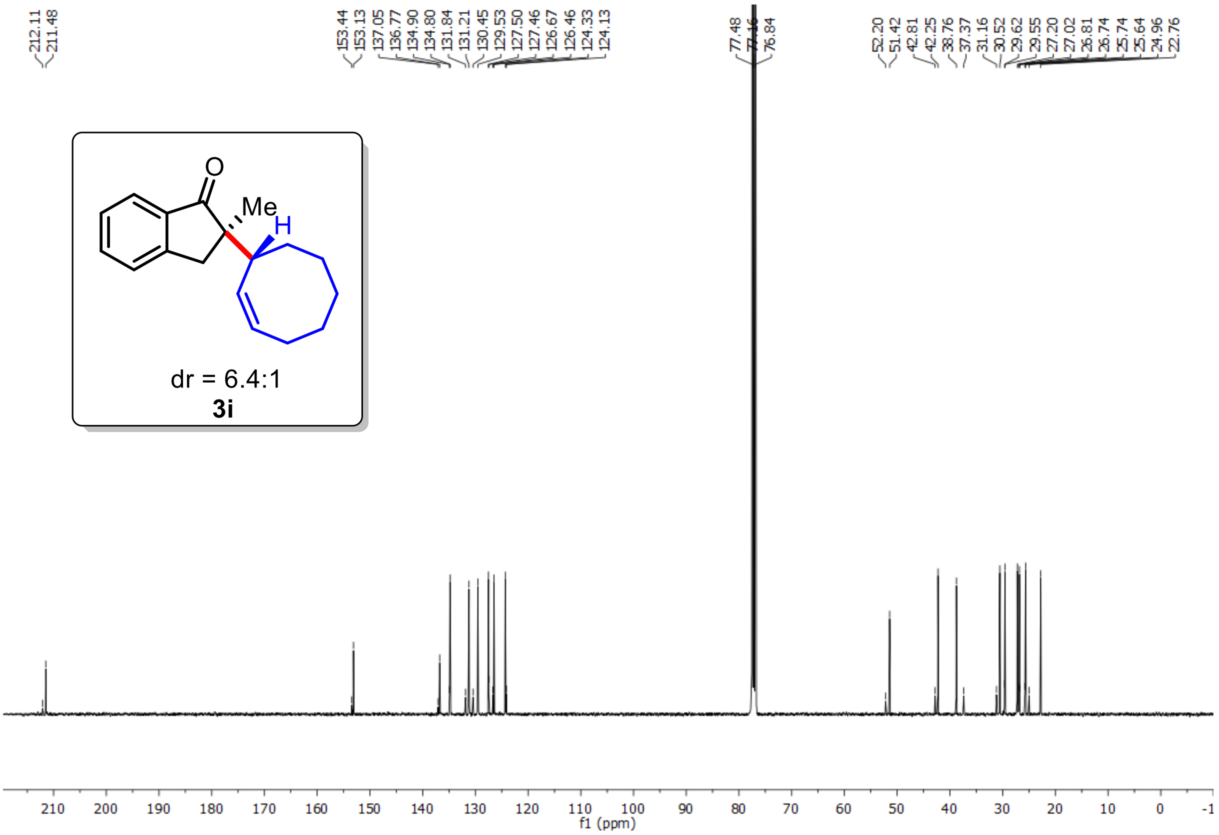
212.11
211.48
153.44
153.13
137.05
136.77
134.90
134.80
131.84
131.21
130.45
129.53
127.50
127.46
126.67
126.46
124.33
124.13
77.48
[illegible]
76.84
52.20
51.42
42.81
42.25
38.76
37.37
31.16
30.52
29.62
29.55
27.20
27.02
26.81
26.74
25.74
25.64
24.96
22.76
O
Me
H
dr = 6.4:1
3i
f1 (ppm)

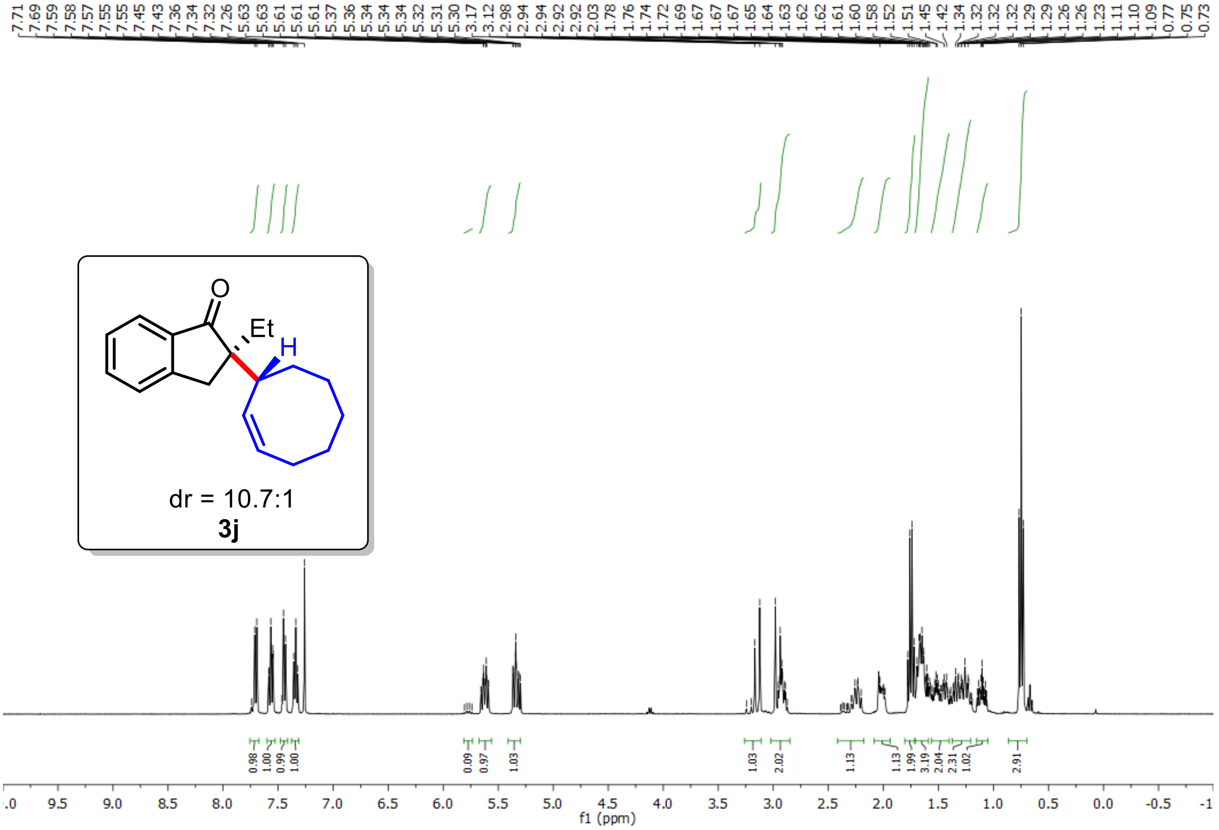
7.71 7.69 7.59 7.58 7.57 7.55 7.55 7.45 7.43 7.36 7.34 7.32 7.26 5.63 5.63 5.61 5.61 5.61 5.37 5.36 5.34 5.34 5.34 5.32 5.31 5.30 3.17 3.12 2.98 2.94 2.94 2.92 2.92 2.03 1.78 1.76 1.74 1.72 1.69 1.67 1.67 1.65 1.64 1.63 1.62 1.61 1.60 1.58 1.52 1.51 1.45 1.42 1.34 1.32 1.32 1.32 1.29 1.29 1.26 1.23 1.11 1.10 1.09 0.77 0.75 0.73
O
Et
H
dr = 10.7:1
3j
0.98 1.00 0.99 1.00 0.09 0.97 1.03 1.03 2.02 1.13 1.13 1.99 3.19 2.04 2.31 1.02 2.91
f1 (ppm)

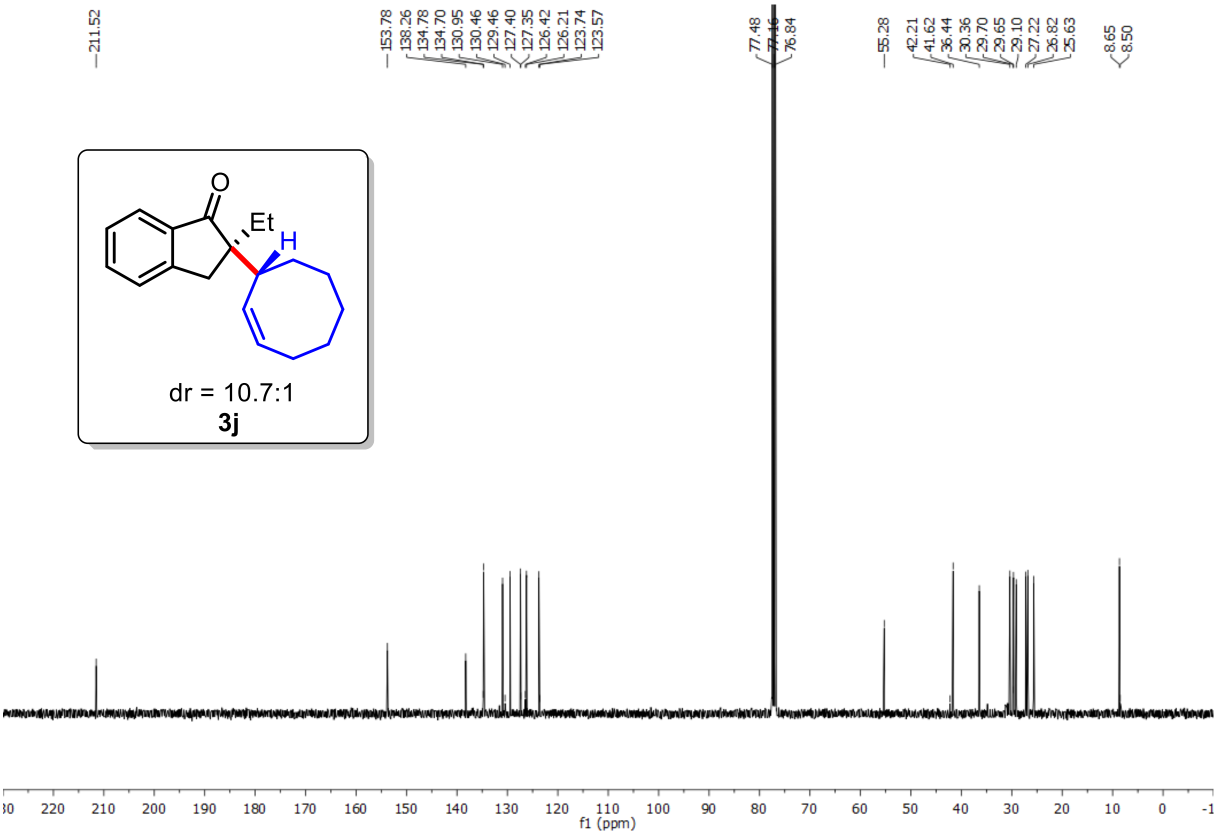
211.52
153.78
138.26
134.78
134.70
130.95
130.46
129.46
127.40
127.35
126.42
126.21
123.74
123.57
77.48
77.16
76.84
55.28
42.21
41.62
36.44
30.36
29.70
29.65
29.10
27.22
26.82
25.63
8.65
8.50
O
Et
H
dr = 10.7:1
3j
f1 (ppm)

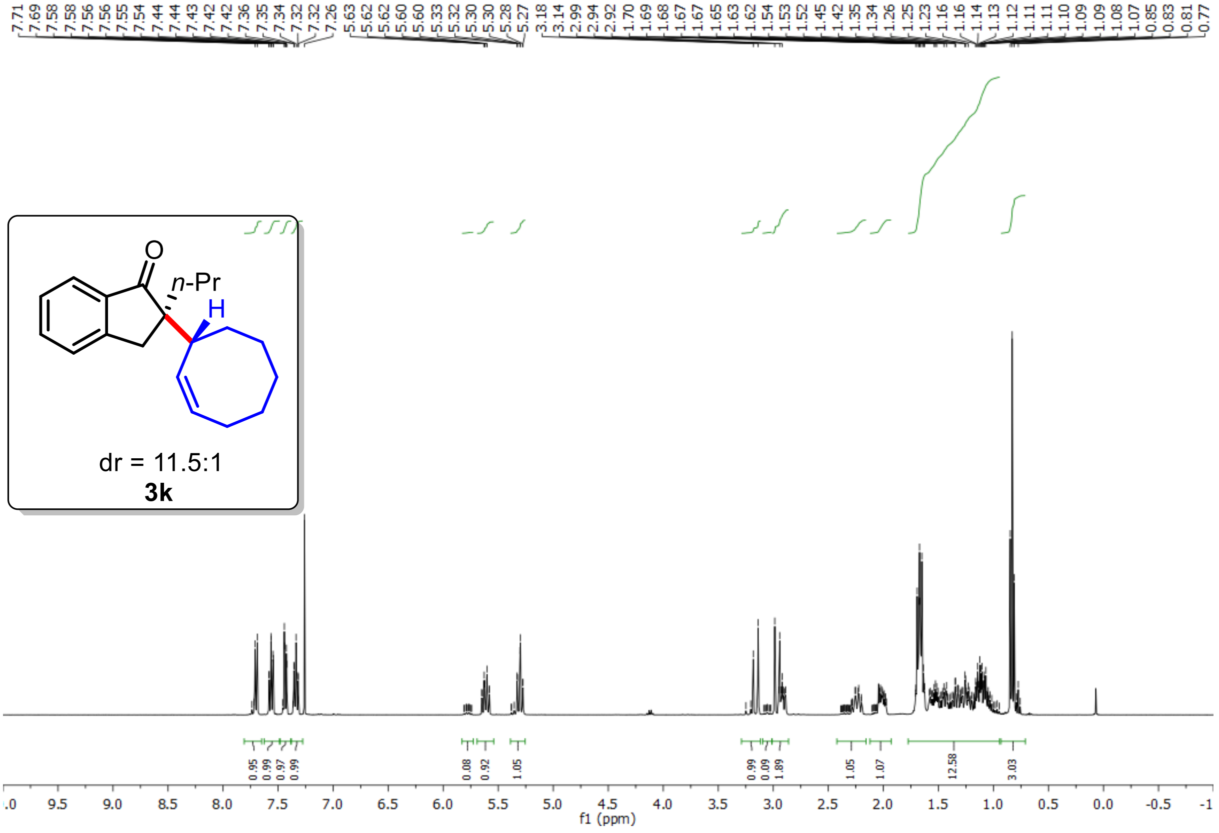
O
n-Pr
H
dr = 11.5:1
3k
0.95
0.99
0.97
0.99
0.08
0.92
1.05
0.99
0.09
1.89
1.05
1.07
12.58
3.03
f1 (ppm)

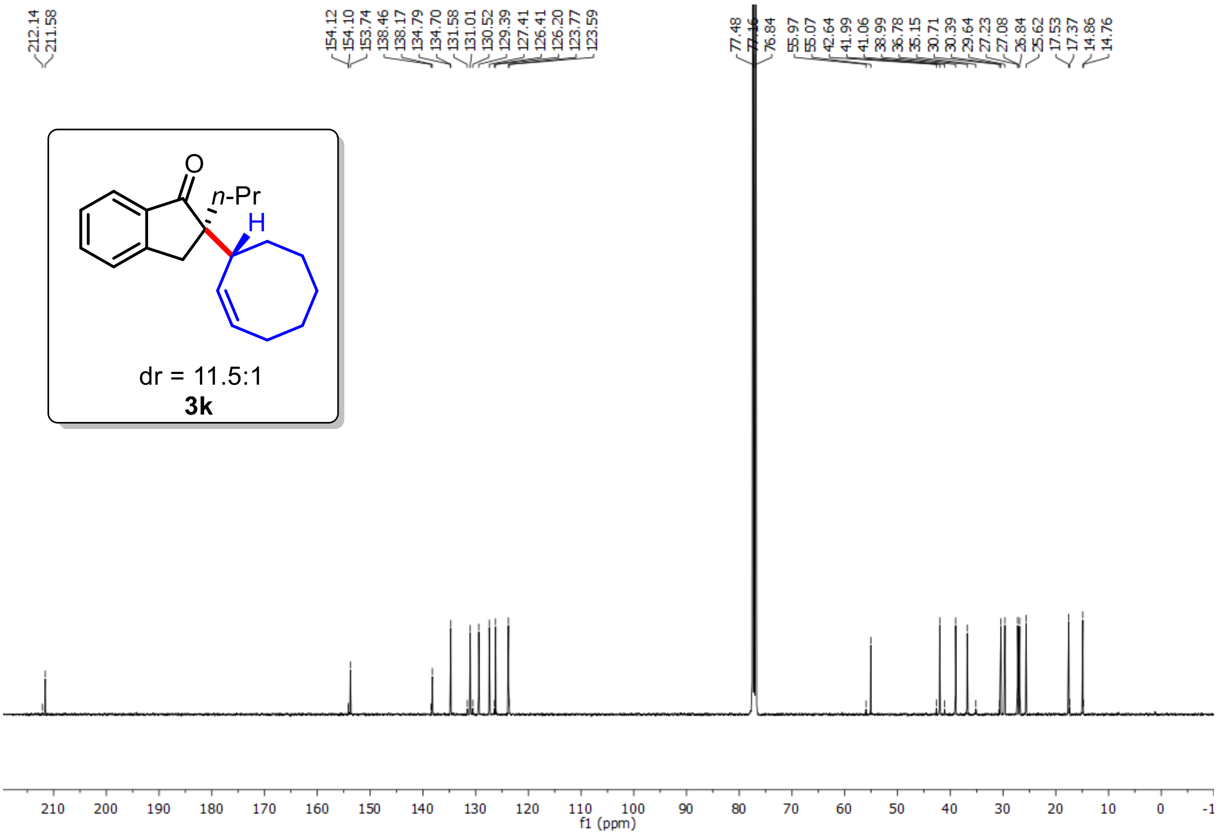
212.14
211.58
154.12
154.10
153.74
138.46
138.17
134.79
134.70
131.58
131.01
130.52
129.39
127.41
126.41
126.20
123.77
123.59
77.48
77.16
76.84
55.97
55.07
42.64
41.99
41.06
38.99
36.78
35.15
30.71
30.39
29.64
27.23
27.08
26.84
25.62
17.53
17.37
14.86
14.76
O
n-Pr
H
dr = 11.5:1
3k
f1 (ppm)

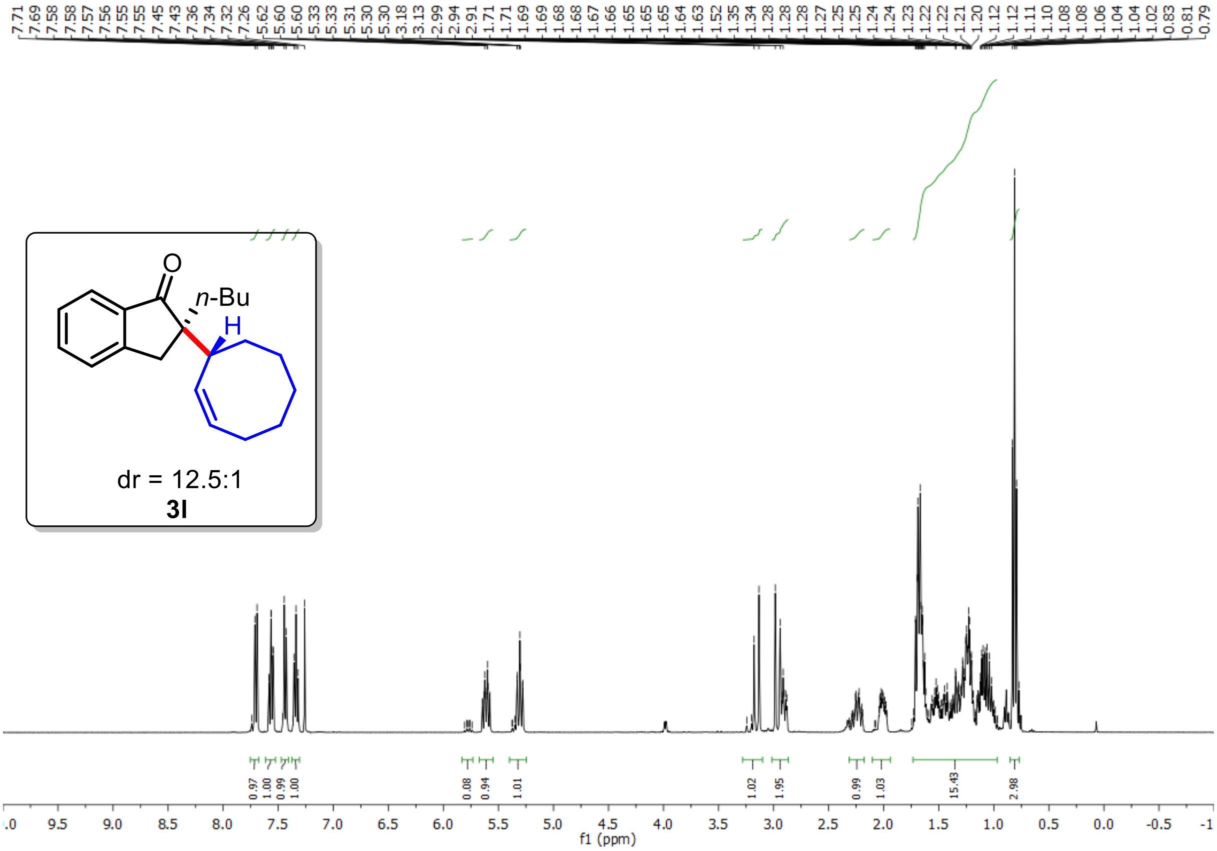
7.71
7.69
7.58
7.58
7.57
7.56
7.55
7.55
7.45
7.43
7.36
7.34
7.32
7.26
5.62
5.60
5.60
5.33
5.33
5.31
5.30
5.30
3.18
3.13
2.99
2.94
2.91
1.71
1.71
1.69
1.69
1.68
1.67
1.66
1.65
1.65
1.64
1.63
1.52
1.35
1.34
1.28
1.28
1.27
1.25
1.25
1.24
1.24
1.23
1.22
1.22
1.21
1.20
1.12
1.12
1.11
1.10
1.08
1.06
1.04
1.02
0.83
0.81
0.79
O
n-Bu
H
dr = 12.5:1
3l
0.97
1.00
0.99
1.00
0.08
0.94
1.01
1.02
1.95
0.99
1.03
15.43
2.98
f1 (ppm)

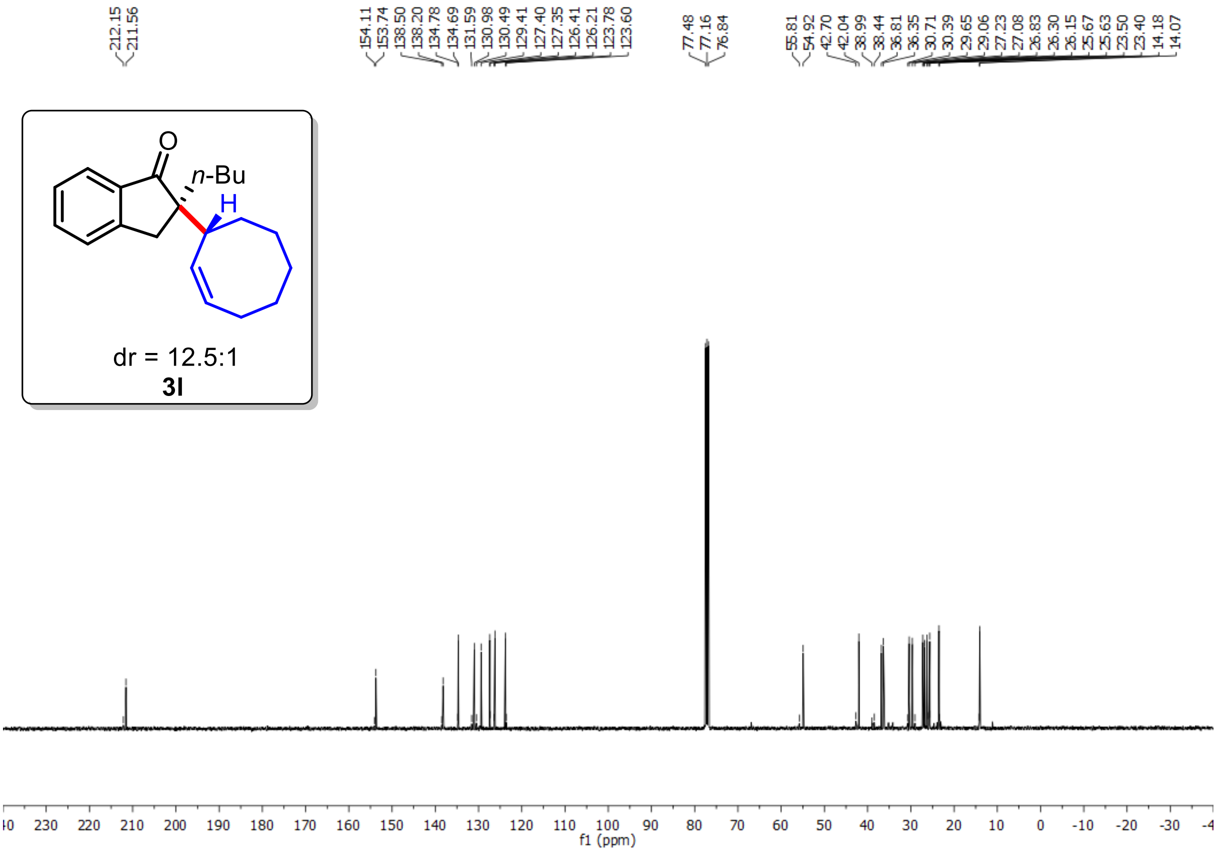
212.15
211.56
154.11
153.74
138.50
138.20
134.78
134.69
131.59
130.98
130.49
129.41
127.40
127.35
126.41
126.21
123.78
123.60
77.48
77.16
76.84
55.81
54.92
42.70
42.04
38.99
38.44
36.81
36.35
30.71
30.39
29.65
29.06
27.23
27.08
26.83
26.30
26.15
25.67
25.63
23.50
23.40
14.18
14.07
O
n-Bu
H
dr = 12.5:1
3l
f1 (ppm)

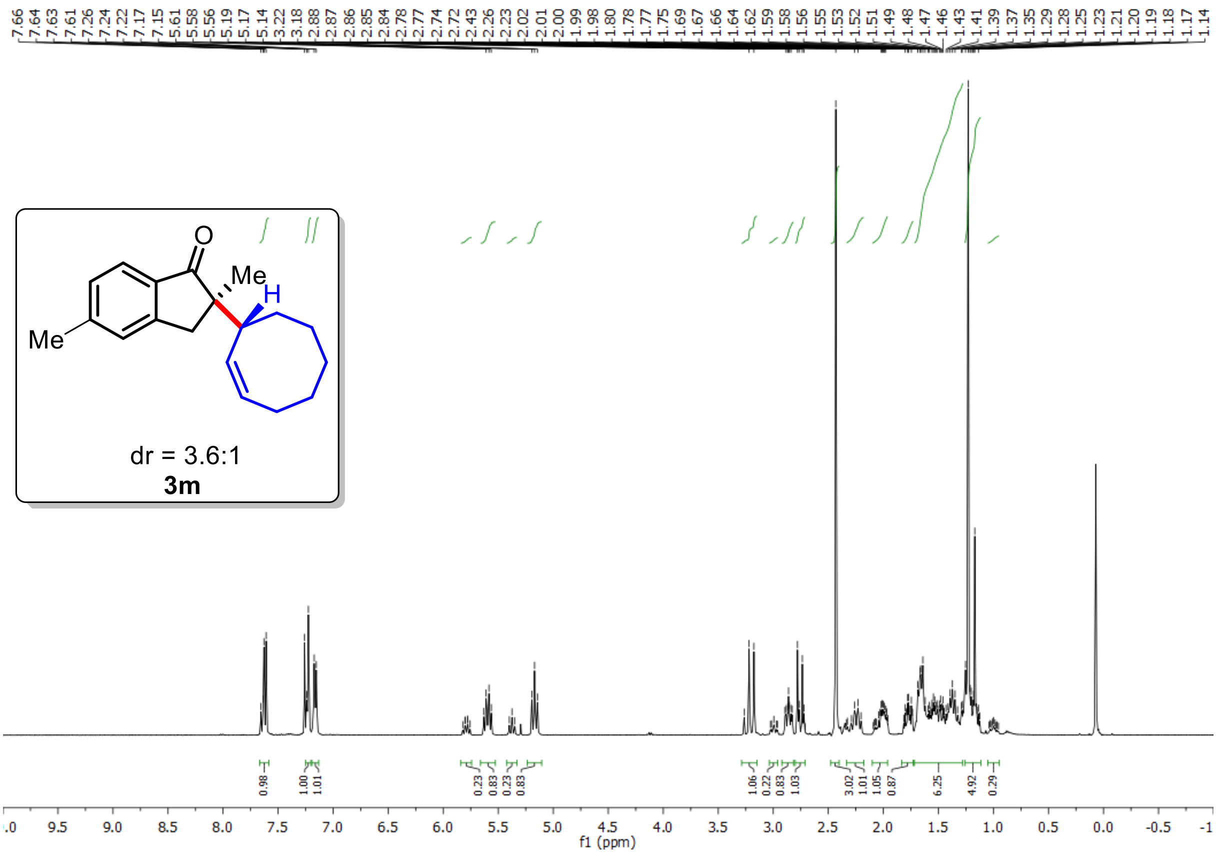
7.66
7.64
7.63
7.61
7.26
7.24
7.22
7.17
7.15
5.61
5.58
5.56
5.19
5.17
5.14
3.22
3.18
2.88
2.87
2.86
2.85
2.84
2.78
2.77
2.74
2.72
2.43
2.26
2.23
2.02
2.01
2.00
1.99
1.98
1.80
1.78
1.77
1.75
1.69
1.67
1.66
1.64
1.62
1.59
1.58
1.56
1.55
1.53
1.52
1.51
1.49
1.48
1.47
1.46
1.43
1.41
1.39
1.37
1.35
1.29
1.28
1.25
1.23
1.21
1.20
1.19
1.18
1.17
1.14
O
Me
H
Me
dr = 3.6:1
3m
0.98
1.00
1.01
0.23
0.83
0.23
0.83
1.06
0.22
0.83
1.03
3.02
1.01
1.05
0.87
6.25
4.92
0.29
f1 (ppm)

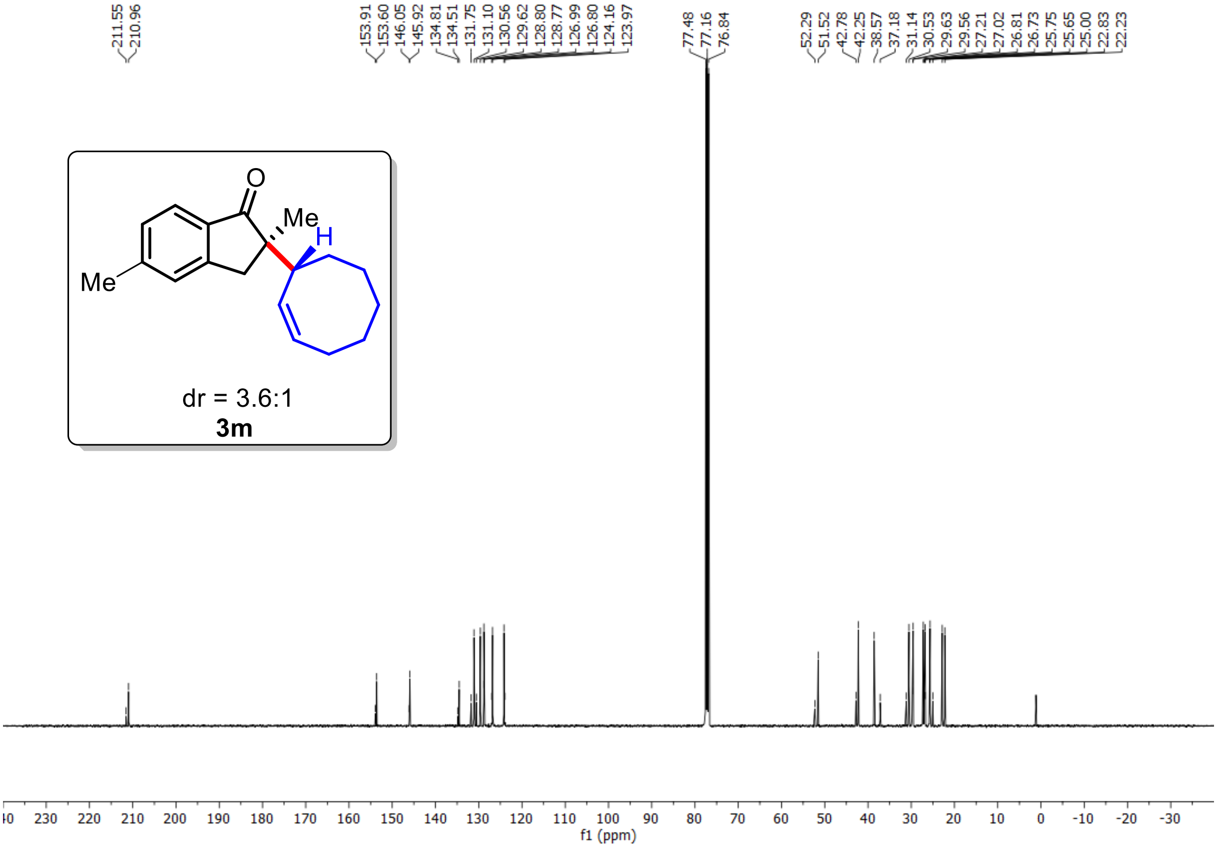
211.55
210.96
153.91
153.60
146.05
145.92
134.81
134.51
131.75
131.10
130.56
129.62
128.80
128.77
126.99
126.80
124.16
123.97
77.48
77.16
76.84
52.29
51.52
42.78
42.25
38.57
37.18
31.14
30.53
29.63
29.56
27.21
27.02
26.81
26.73
25.75
25.65
25.00
22.83
22.23
O
Me
H
Me
dr = 3.6:1
3m
f1 (ppm)

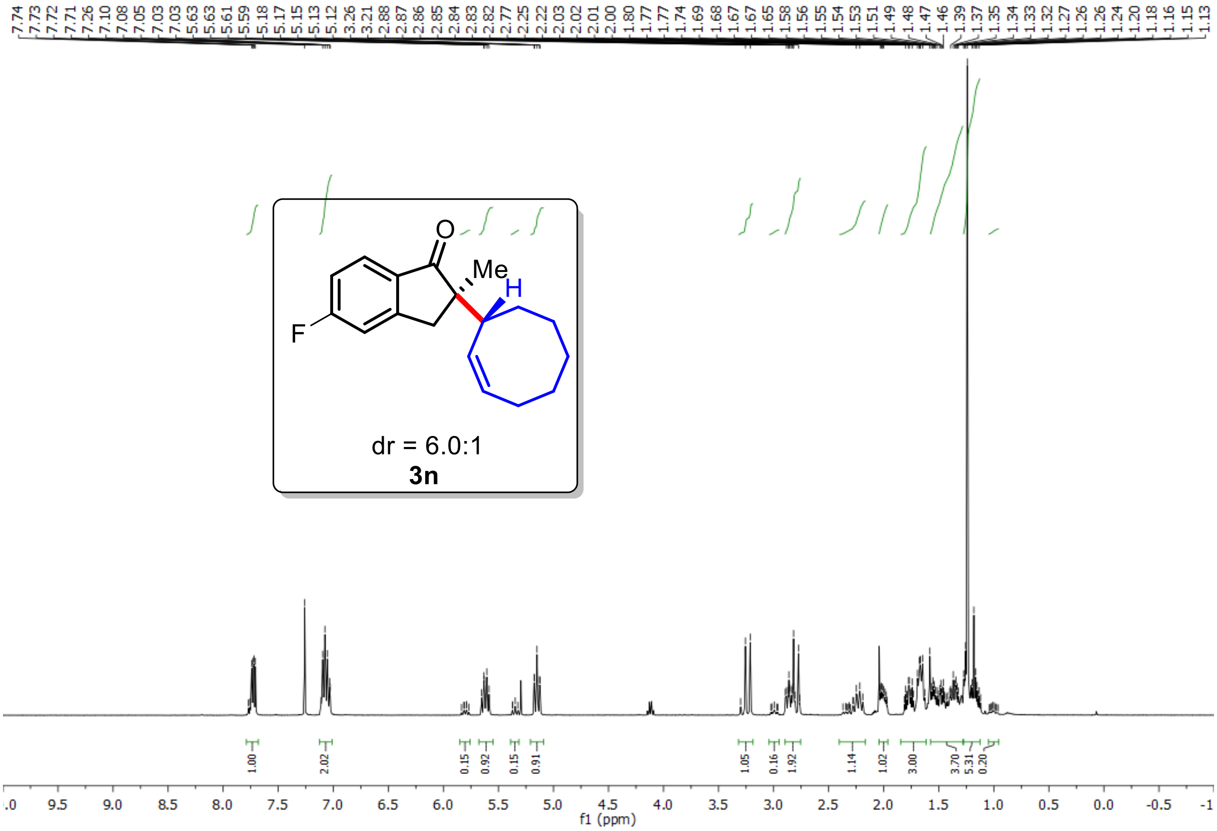
7.74
7.73
7.72
7.71
7.26
7.10
7.08
7.05
7.03
7.03
5.63
5.63
5.61
5.59
5.18
5.17
5.15
5.13
5.12
3.26
3.21
2.88
2.87
2.86
2.85
2.84
2.83
2.82
2.77
2.25
2.22
2.03
2.02
2.01
2.00
1.80
1.77
1.77
1.74
1.69
1.68
1.67
1.65
1.58
1.56
1.55
1.54
1.53
1.51
1.49
1.48
1.47
1.46
1.39
1.37
1.35
1.34
1.33
1.32
1.27
1.26
1.26
1.24
1.20
1.18
1.16
1.15
1.13
O
Me
H
F
dr = 6.0:1
3n
1.00
2.02
0.15
0.92
0.15
0.91
1.05
0.16
1.92
1.14
1.02
3.00
3.70
5.31
0.20
f1 (ppm)

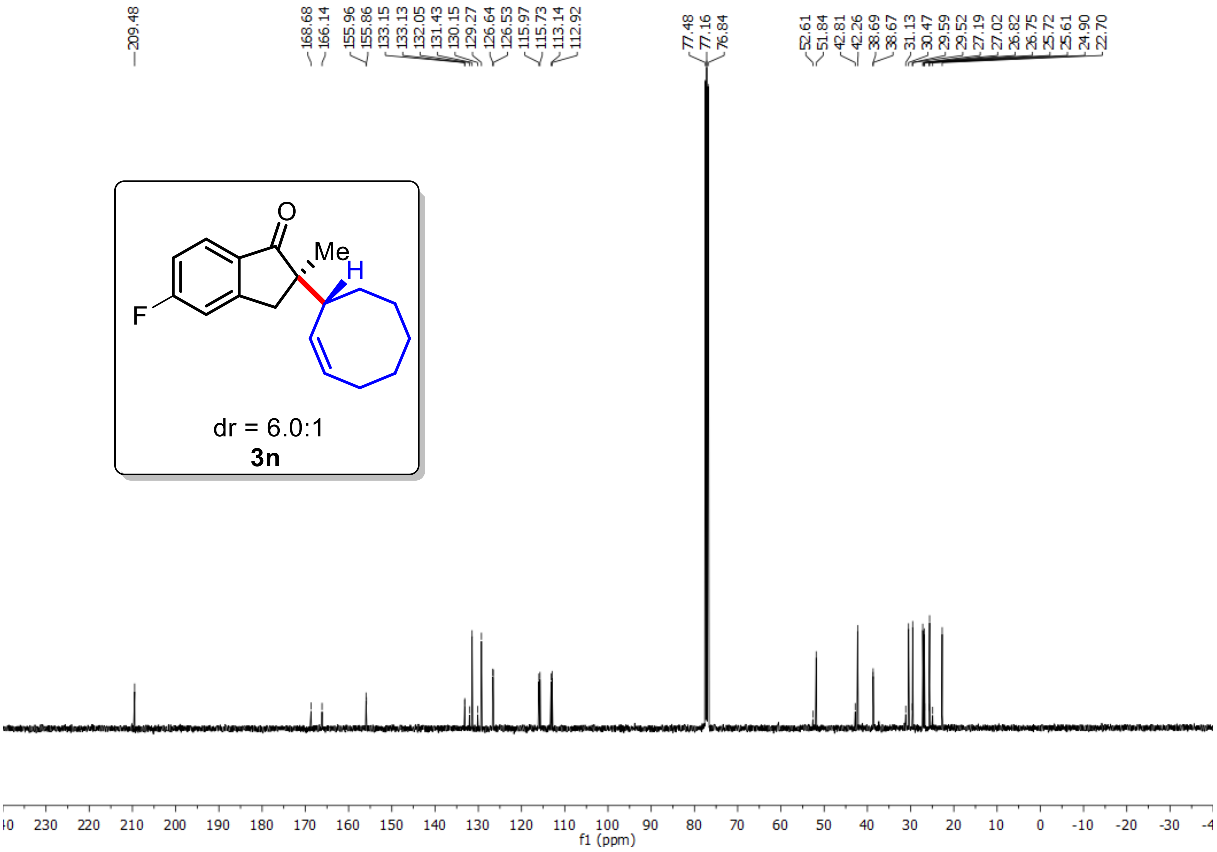
209.48
168.68
166.14
155.96
155.86
133.15
133.13
132.05
131.43
130.15
129.27
126.64
126.53
115.97
115.73
113.14
112.92
77.48
77.16
76.84
52.61
51.84
42.81
42.26
38.69
38.67
31.13
30.47
29.59
29.52
27.19
27.02
26.82
26.75
25.72
25.61
24.90
22.70
O
Me
H
F
dr = 6.0:1
3n
f1 (ppm)

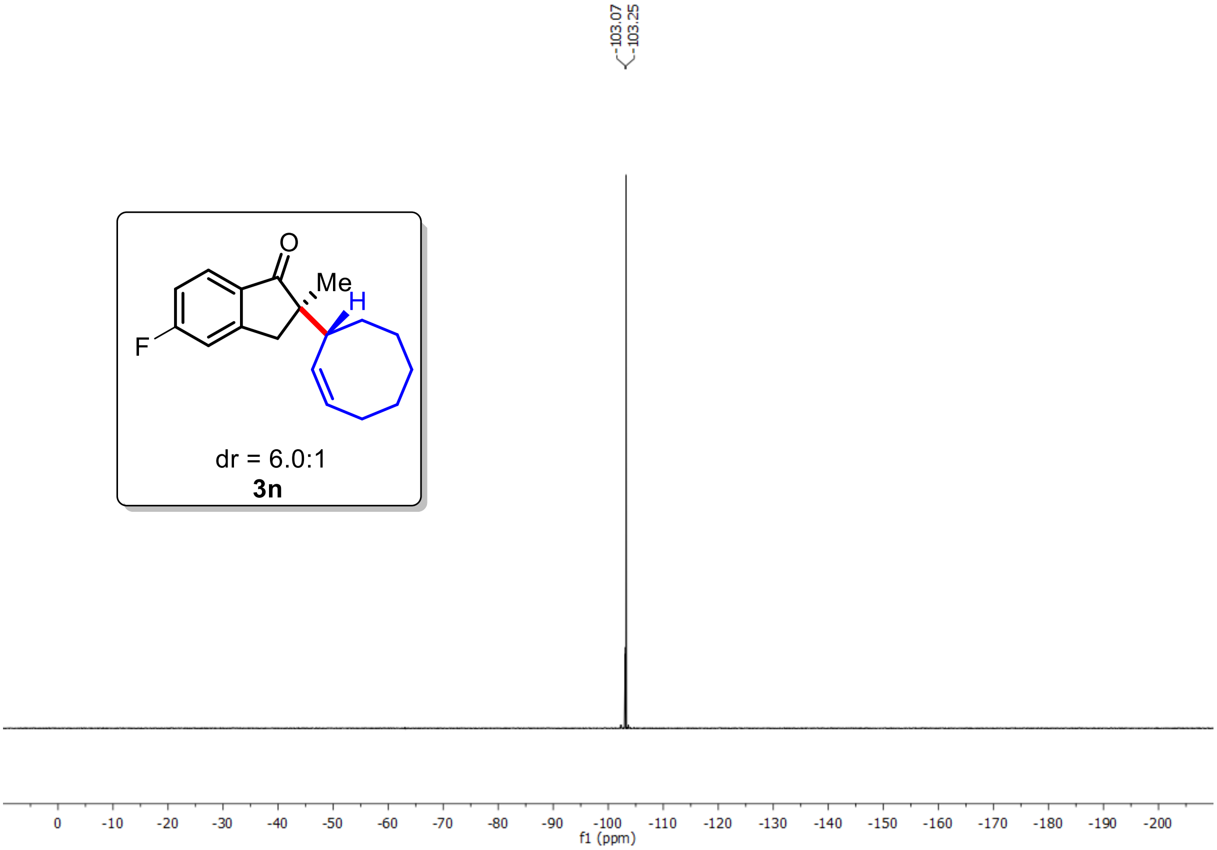
-103.07
-103.25
O
Me
H
F
dr = 6.0:1
3n
f1 (ppm)

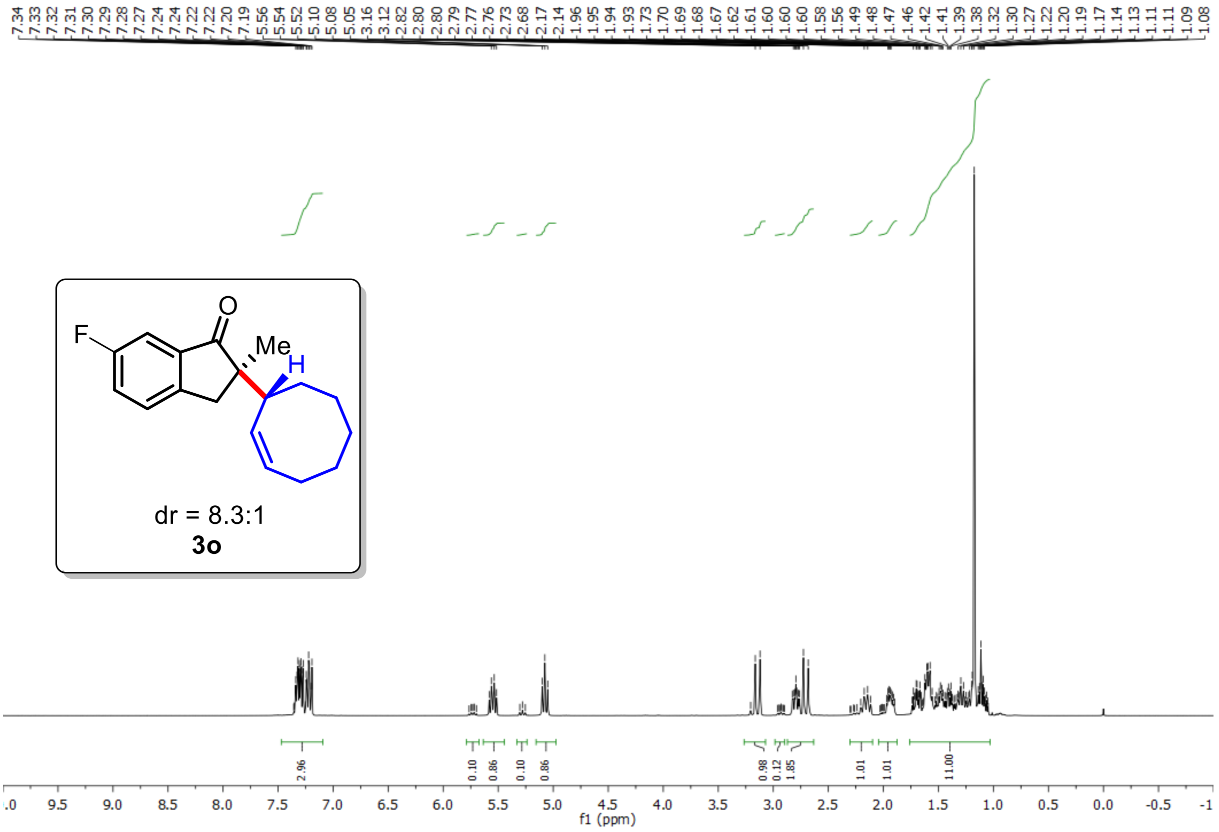

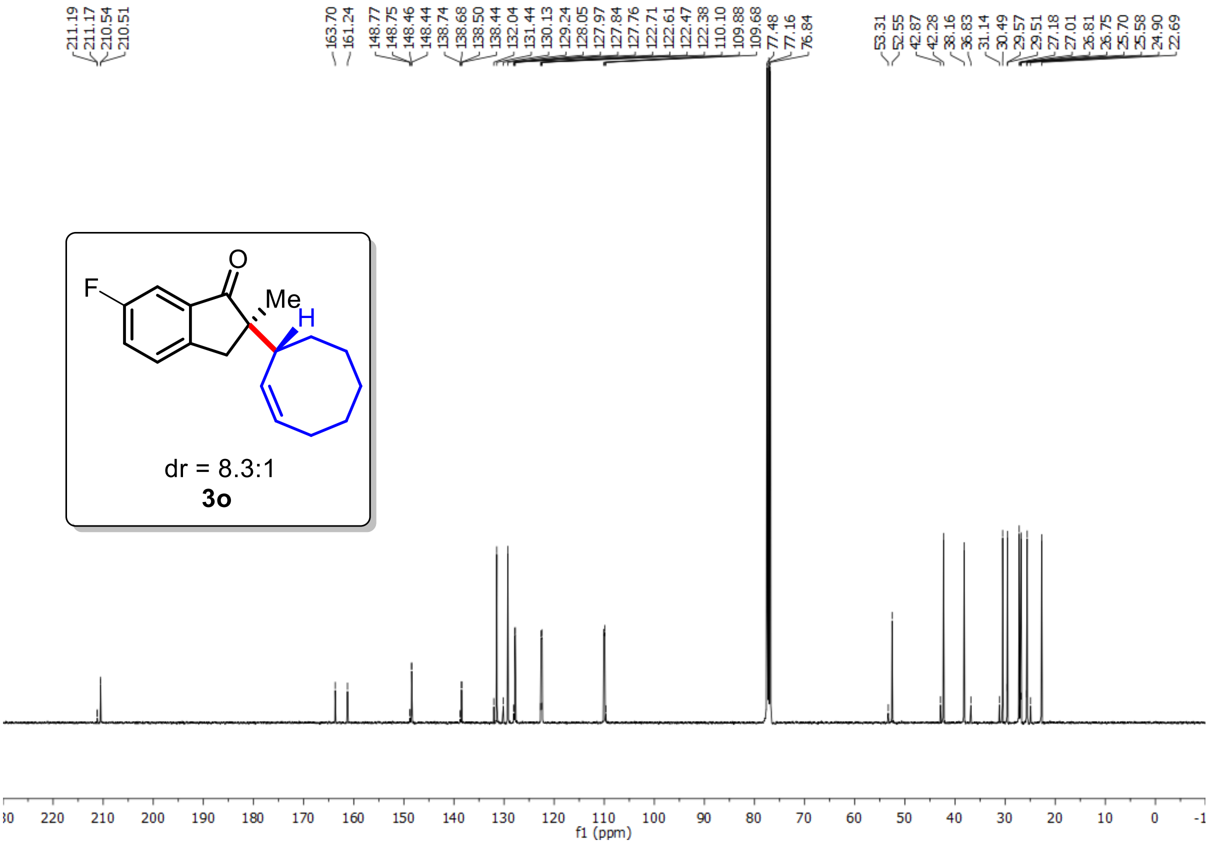

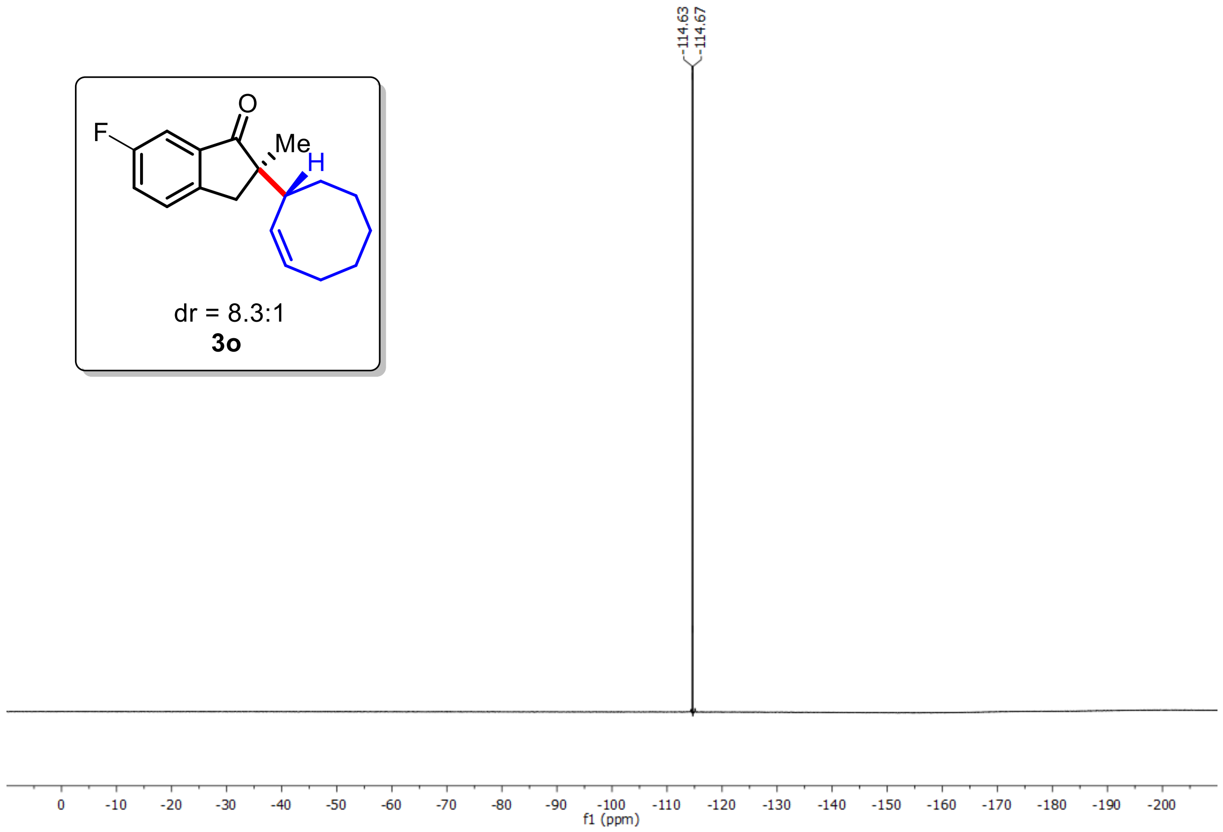
-114.63
-114.67
dr = 8.3:1
3o
f1 (ppm)

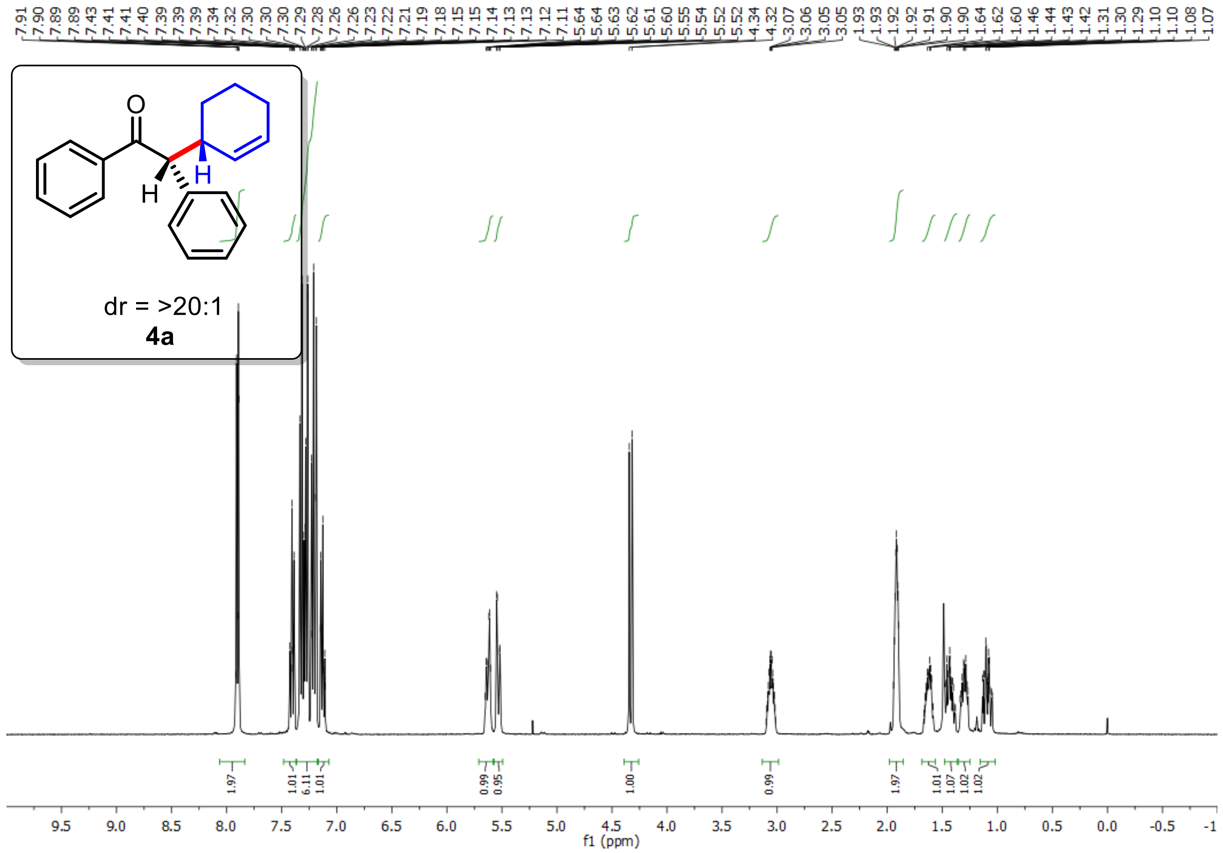
dr = >20:1
4a
f1 (ppm)

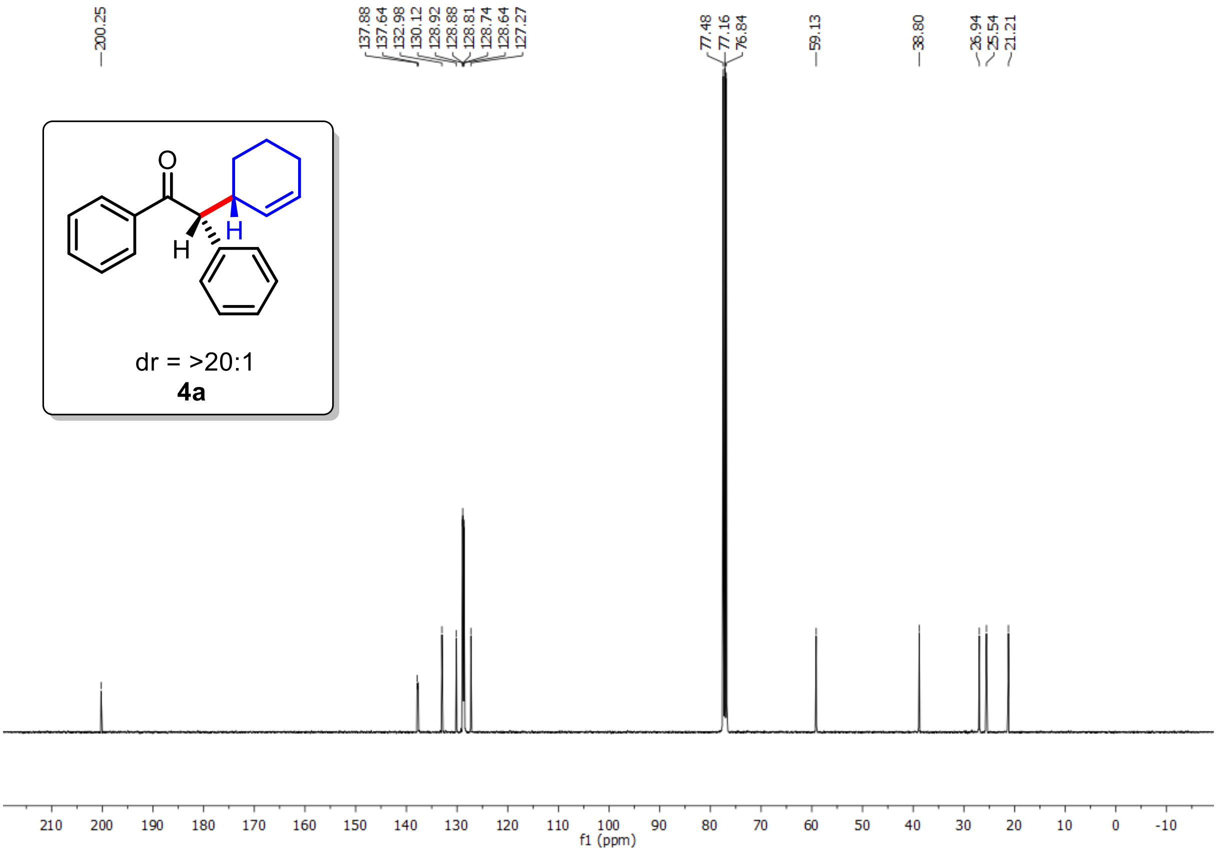
200.25
137.88
137.64
132.98
130.12
128.92
128.88
128.81
128.74
128.64
127.27
77.48
77.16
76.84
59.13
38.80
26.94
25.54
21.21
O
H
H
dr = >20:1
4a
210 200 190 180 170 160 150 140 130 120 110 100 90 80 70 60 50 40 30 20 10 0 -10
f1 (ppm)

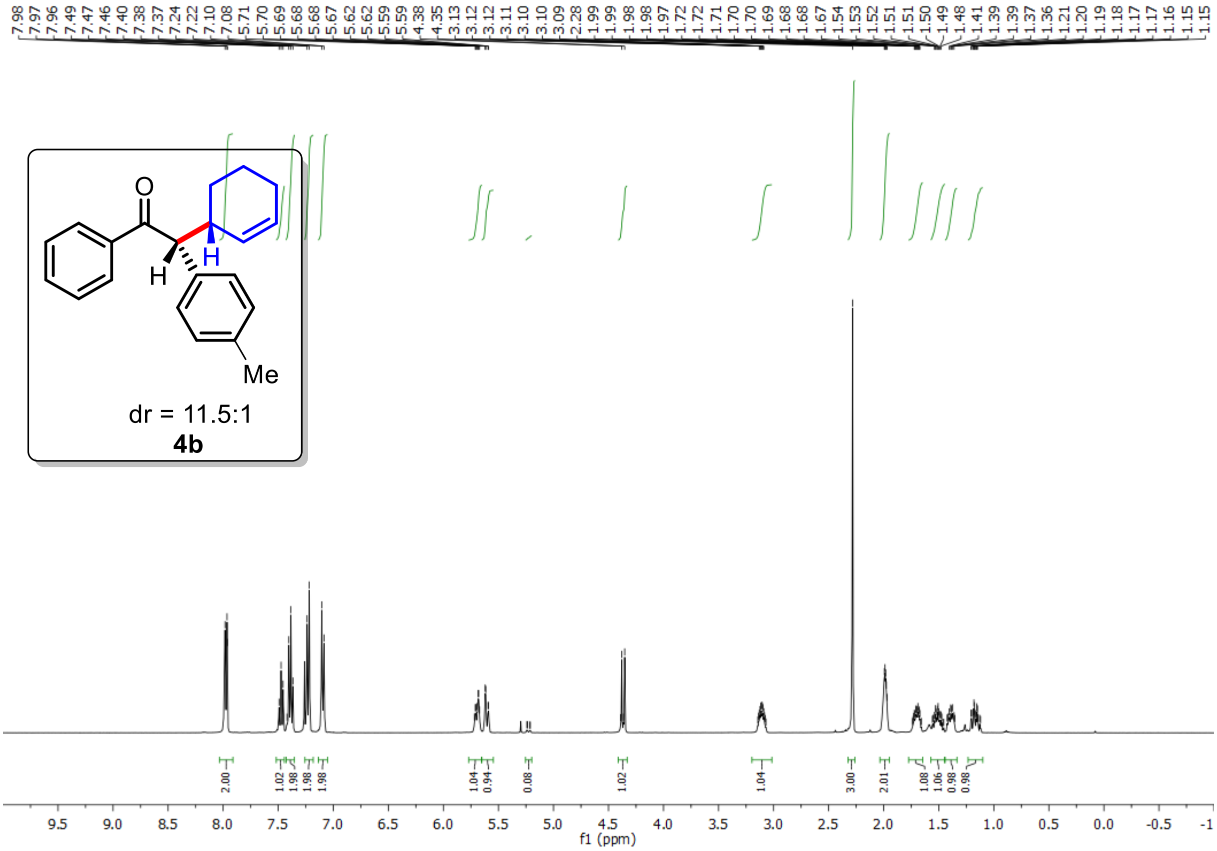
7.98 7.97 7.96 7.49 7.47 7.46 7.40 7.38 7.37 7.24 7.22 7.10 7.08 5.71 5.70 5.69 5.68 5.68 5.67 5.62 5.62 5.59 5.59 4.38 4.35 3.13 3.12 3.12 3.11 3.11 3.10 3.10 3.09 2.28 1.99 1.99 1.98 1.98 1.97 1.72 1.72 1.71 1.70 1.70 1.69 1.68 1.68 1.67 1.54 1.53 1.52 1.51 1.51 1.50 1.49 1.48 1.41 1.39 1.37 1.36 1.21 1.20 1.19 1.18 1.17 1.17 1.16 1.15 1.15
O
H
H
Me
dr = 11.5:1
4b
2.00 1.02 1.98 1.98 1.98 1.04 0.94 0.08 1.02 1.04 3.00 2.01 1.08 1.06 0.98 0.98
9.5 9.0 8.5 8.0 7.5 7.0 6.5 6.0 5.5 5.0 4.5 4.0 3.5 3.0 2.5 2.0 1.5 1.0 0.5 0.0 -0.5 -1
f1 (ppm)

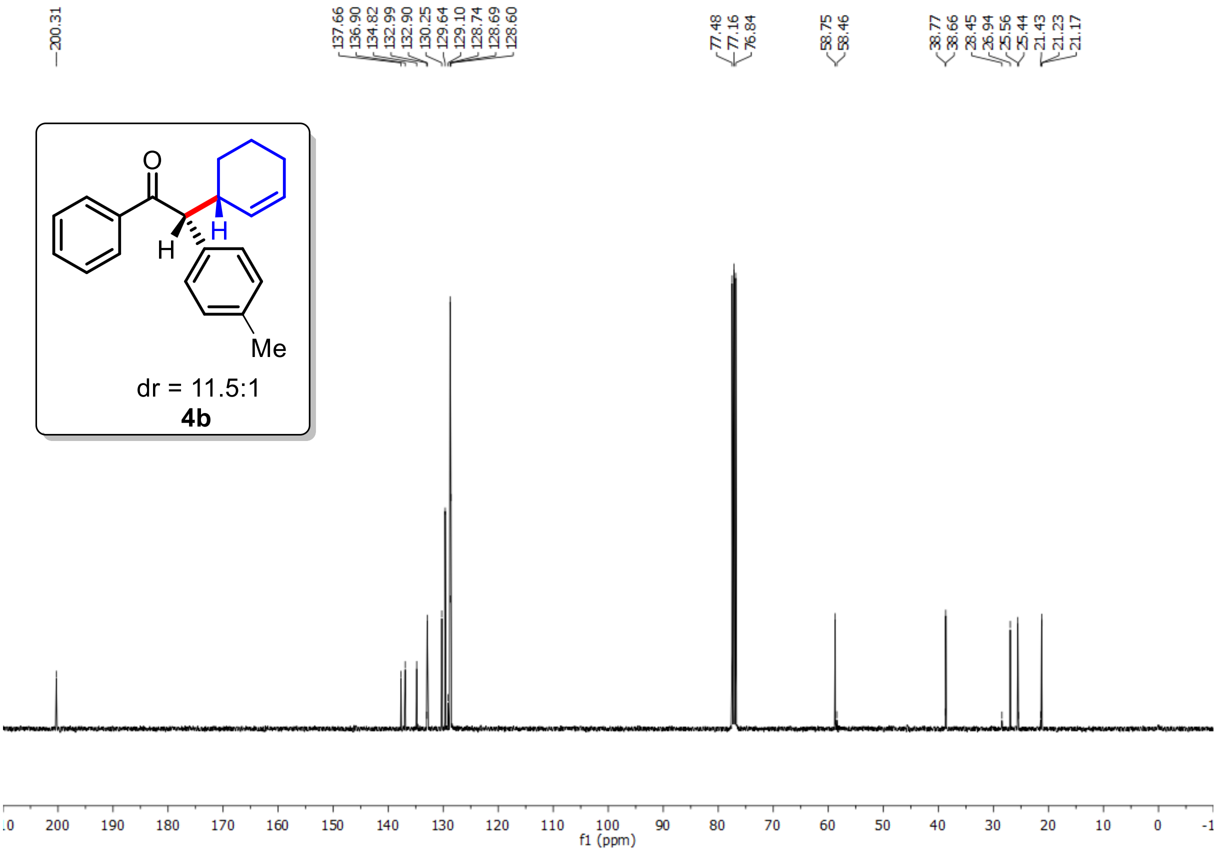
200.31
137.66 136.90 134.82 132.99 132.90 130.25 129.64 129.10 128.74 128.69 128.60
77.48 77.16 76.84
58.75 58.46
38.77 38.66 28.45 26.94 25.56 25.44 21.43 21.23 21.17
O
H
H
Me
dr = 11.5:1
4b
f1 (ppm)

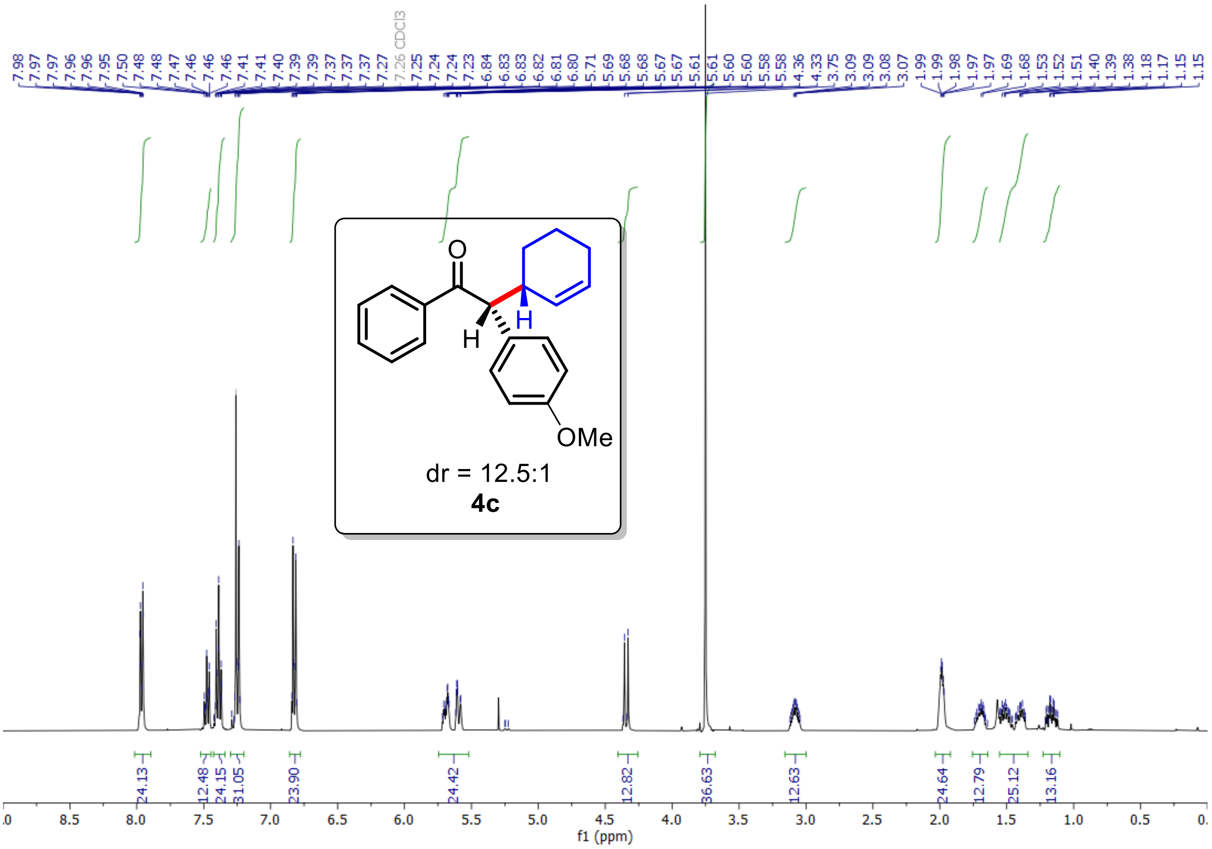
7.98 7.97 7.97 7.96 7.96 7.95 7.50 7.48 7.48 7.47 7.46 7.46 7.46 7.41 7.41 7.40 7.39 7.39 7.37 7.37 7.37 7.27 7.26 CDCl3 7.25 7.24 7.24 7.23 6.84 6.83 6.83 6.82 6.81 6.80 5.71 5.69 5.68 5.68 5.67 5.67 5.61 5.61 5.60 5.60 5.58 5.58 4.36 4.33 3.75 3.09 3.08 3.07 1.99 1.99 1.98 1.97 1.97 1.69 1.68 1.53 1.52 1.51 1.40 1.39 1.38 1.18 1.17 1.15 1.15
O
H
H
OMe
dr = 12.5:1
4c
24.13 12.48 24.15 31.05 23.90 24.42 12.82 36.63 12.63 24.64 12.79 25.12 13.16
f1 (ppm)

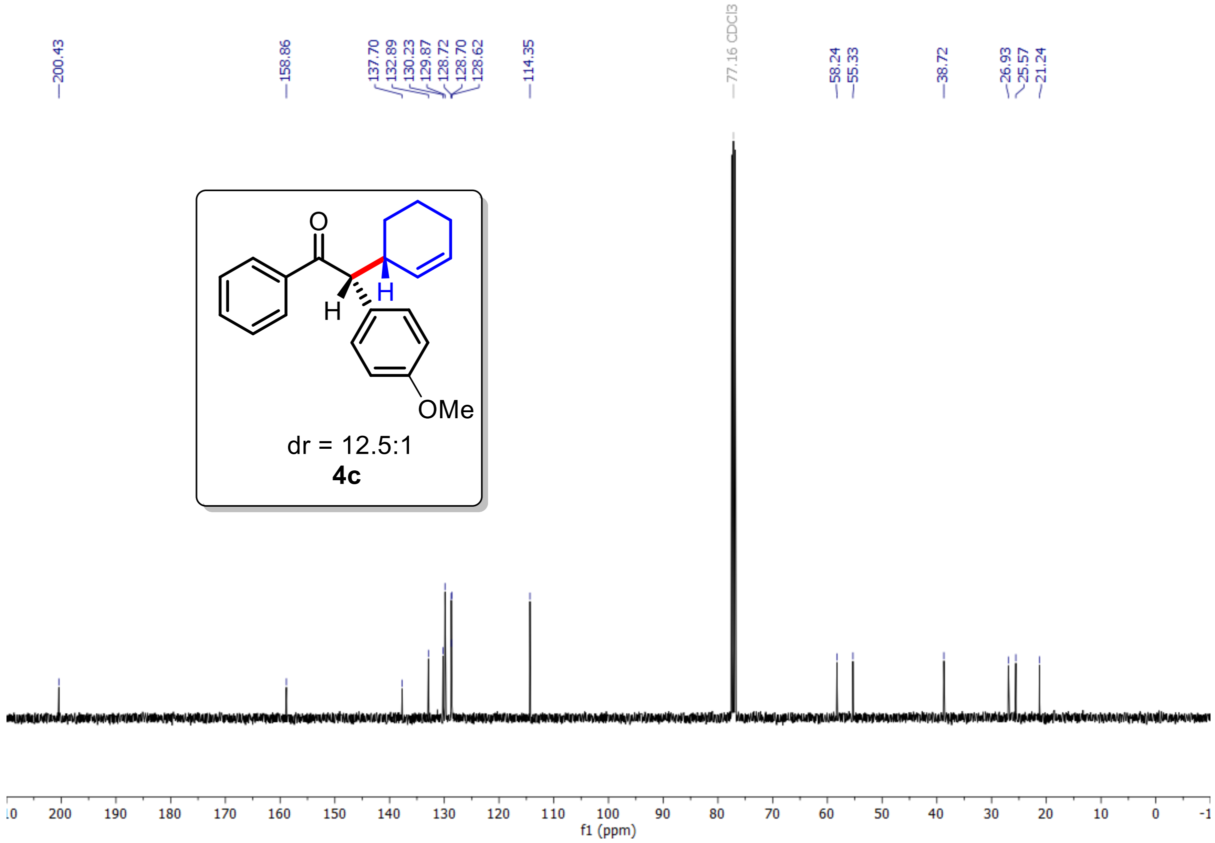
dr = 12.5:1
4c
f1 (ppm)

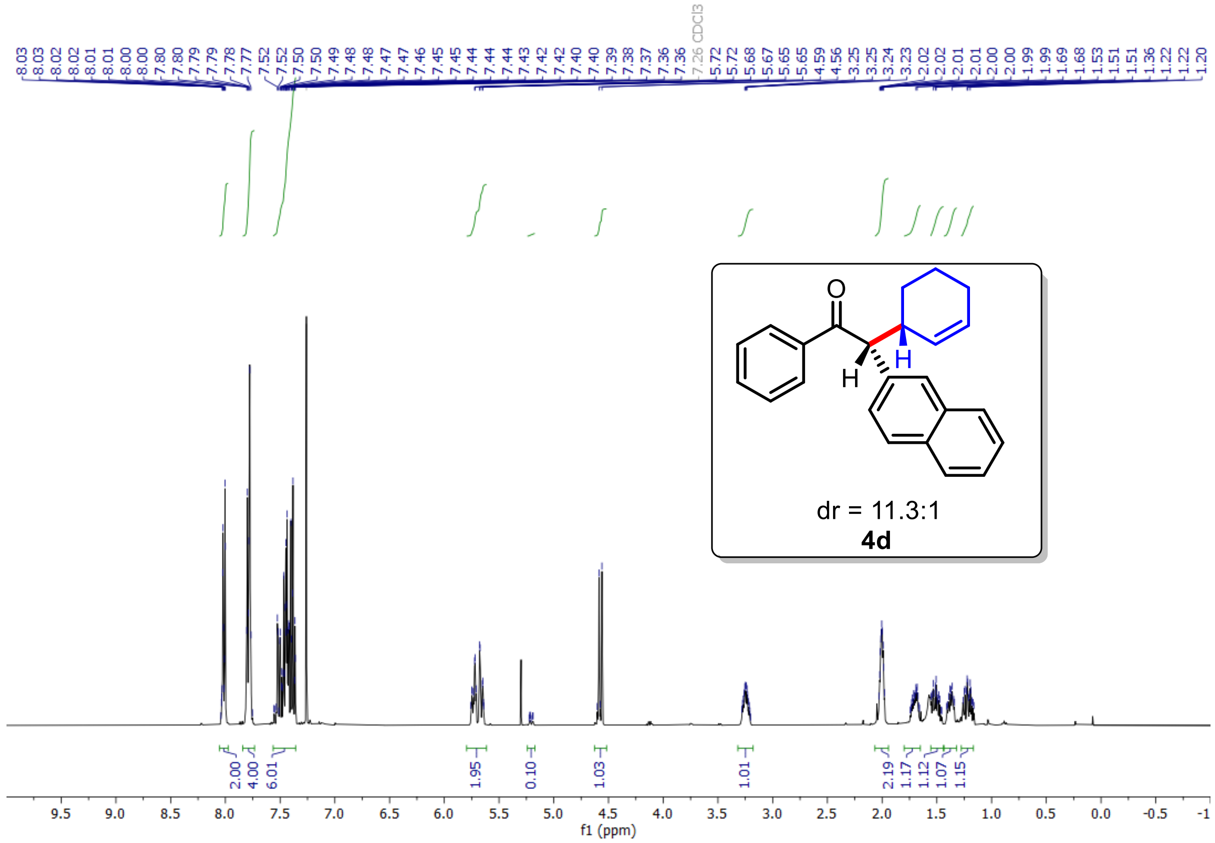
dr = 11.3:1
4d
f1 (ppm)

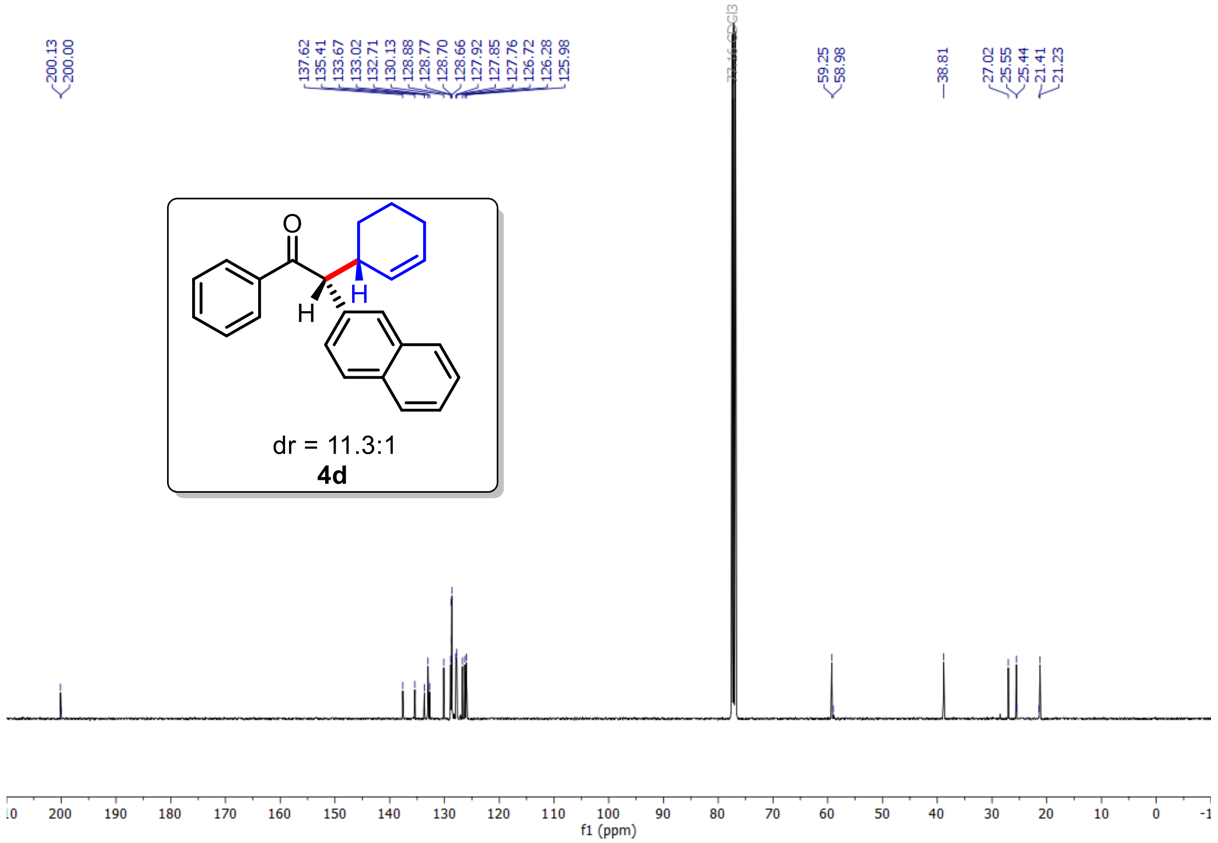

dr = 11.3:1
4d
f1 (ppm)

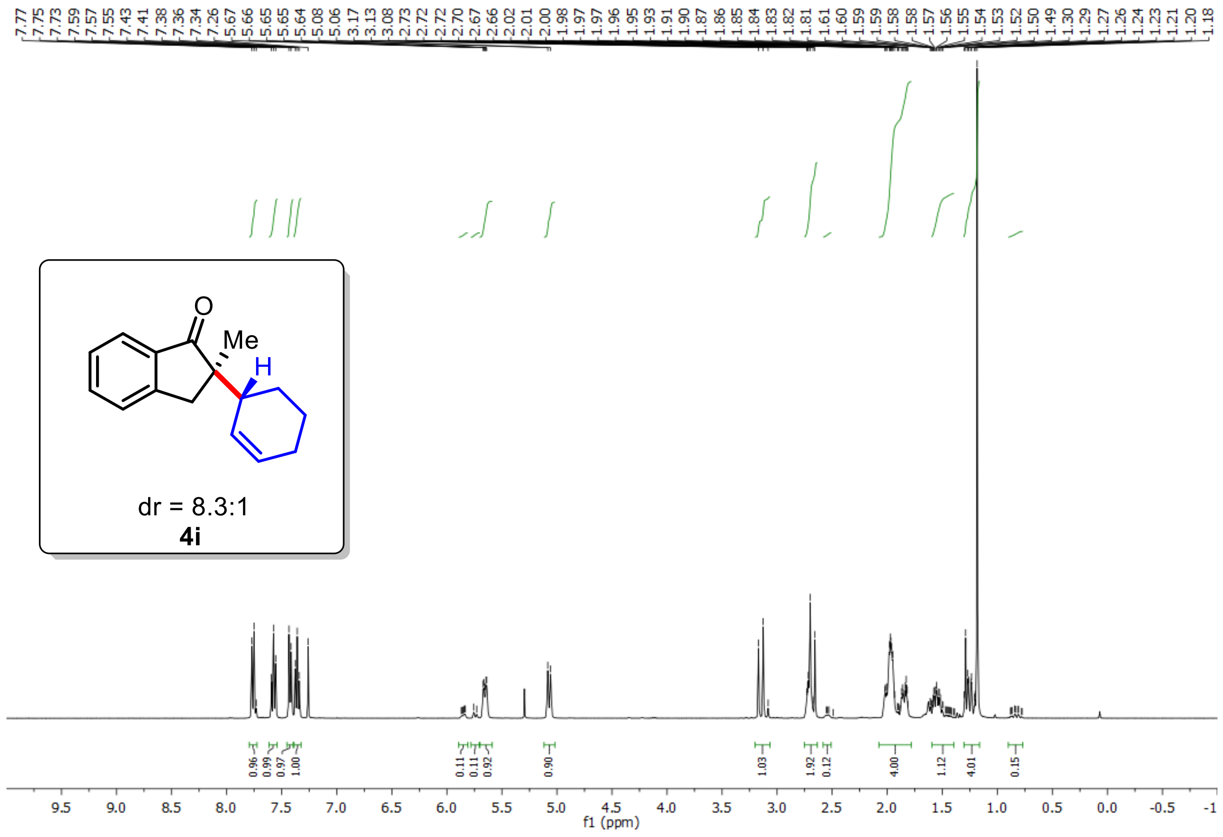

dr = 8.3:1
4i
f1 (ppm)

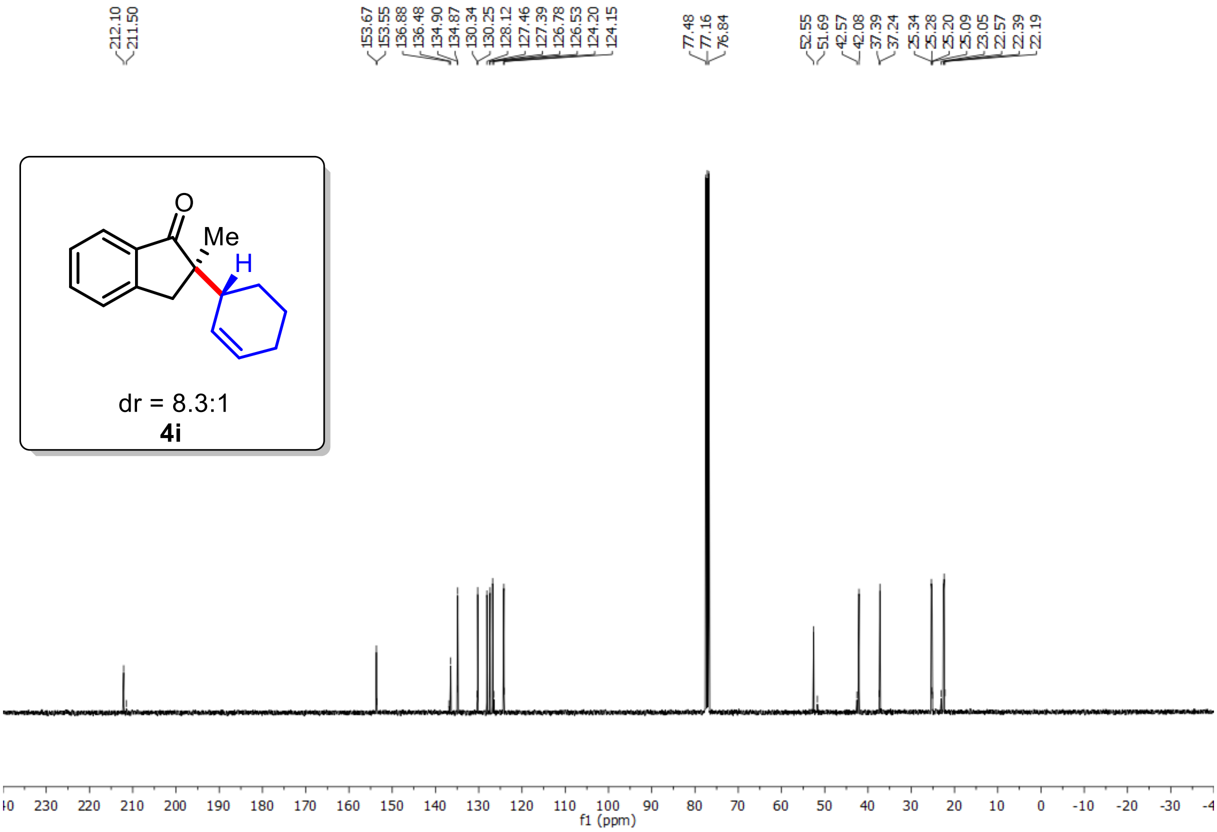
212.10
211.50
153.67
153.55
136.88
136.48
134.90
134.87
130.34
130.25
128.12
127.46
127.39
126.78
126.53
124.20
124.15
77.48
77.16
76.84
52.55
51.69
42.57
42.08
37.39
37.24
25.34
25.28
25.20
25.09
23.05
22.57
22.39
22.19
Me
H
dr = 8.3:1
4i
f1 (ppm)

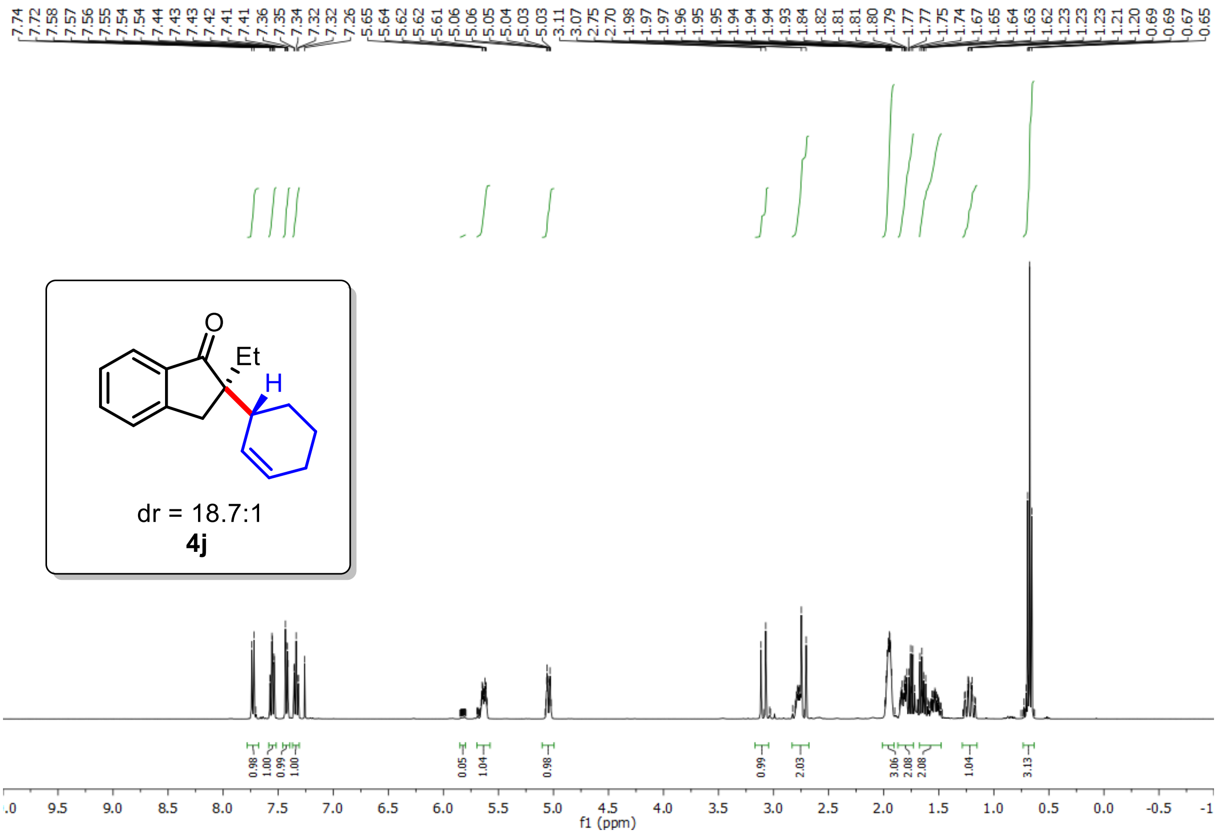
Et
H
dr = 18.7:1
4j
f1 (ppm)

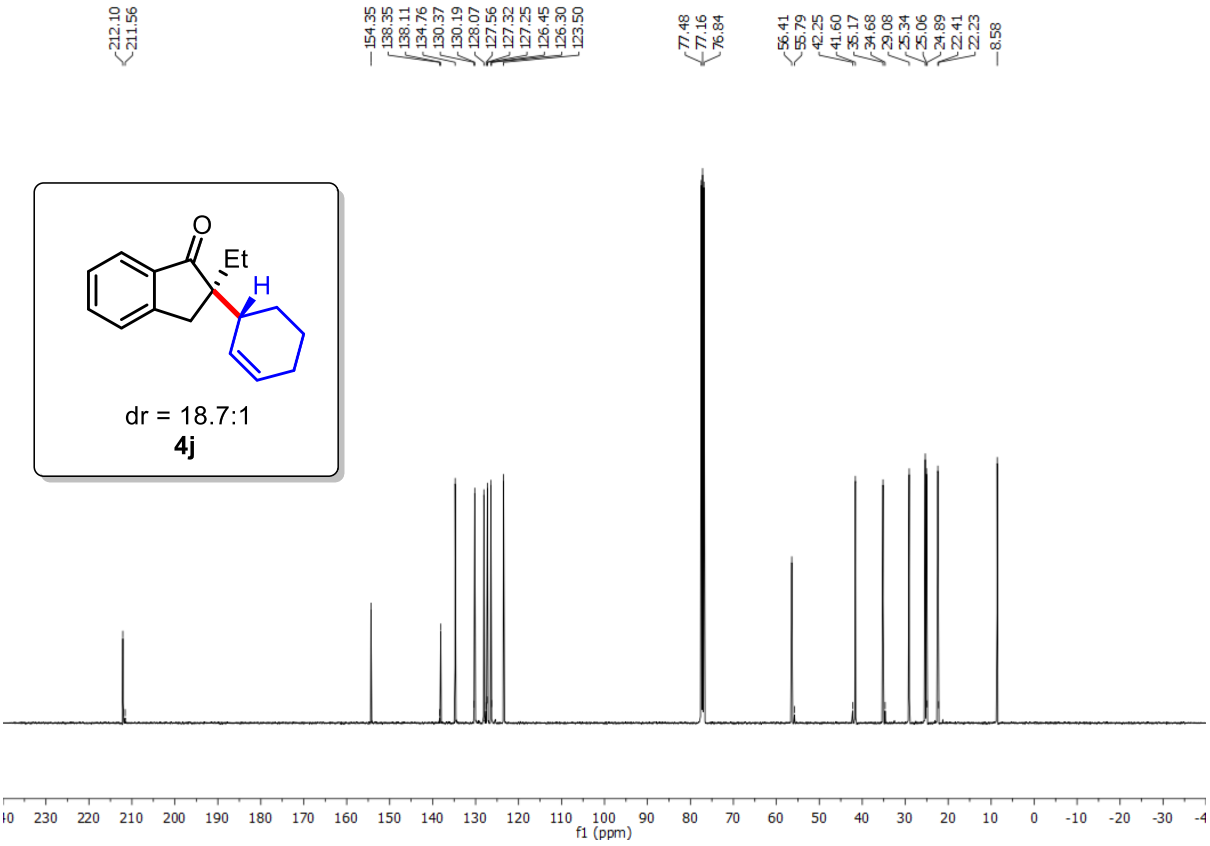

dr = 18.7:1
4j
f1 (ppm)

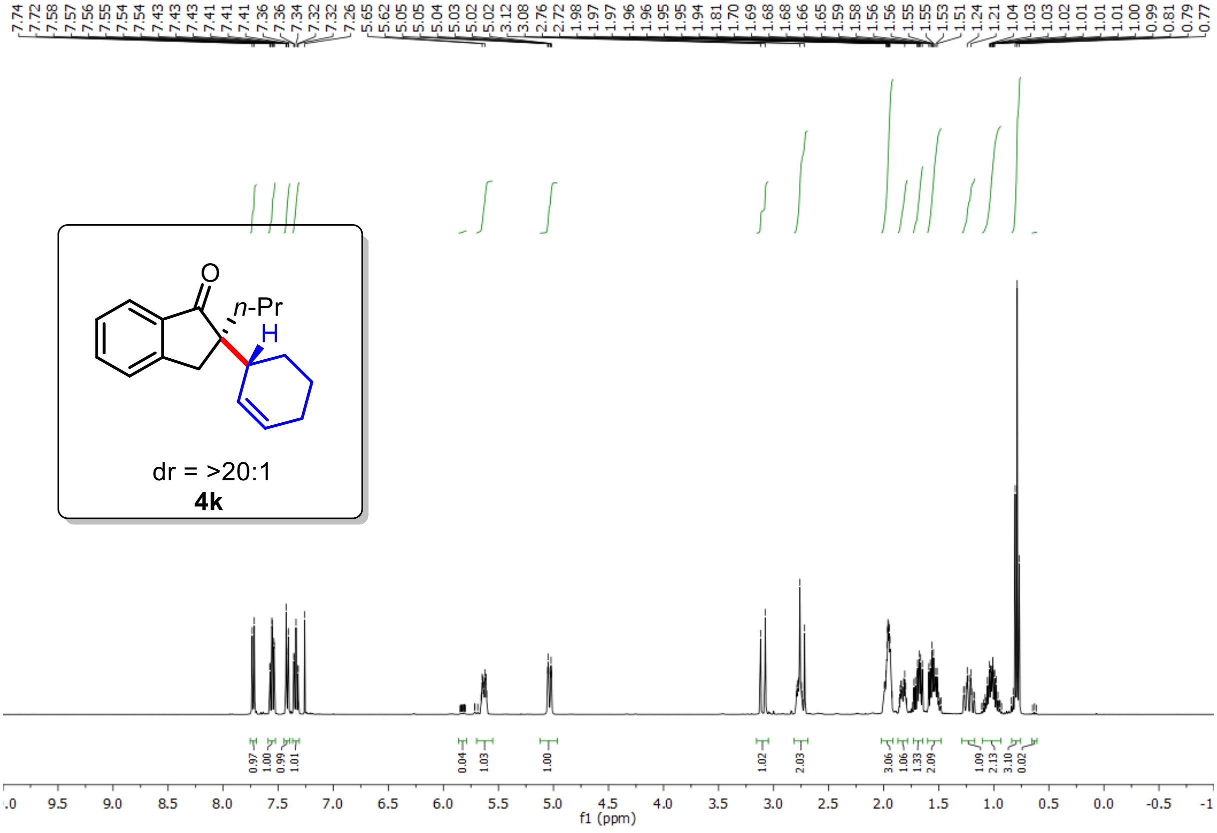

dr = >20:1
4k
f1 (ppm)

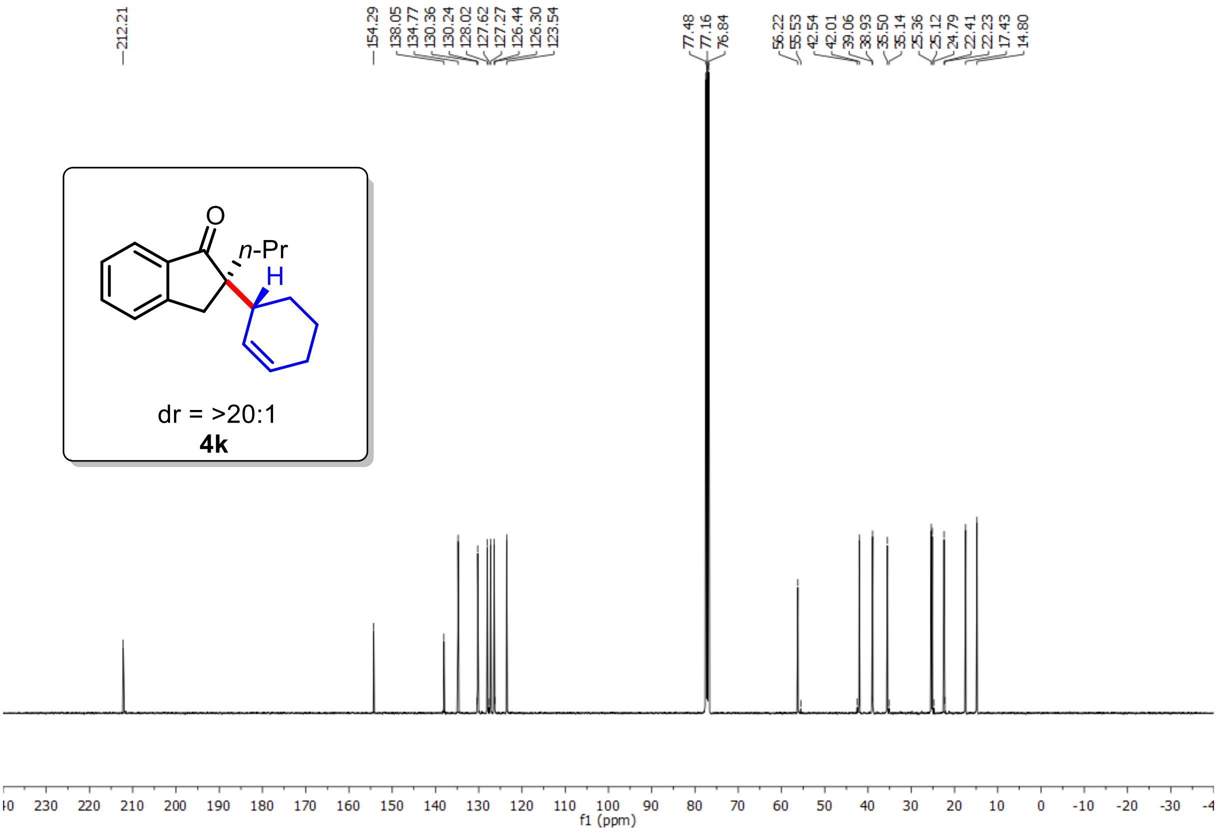
212.21
154.29
138.05
134.77
130.36
130.24
128.02
127.62
127.27
126.44
126.30
123.54
77.48
77.16
76.84
56.22
55.53
42.54
42.01
39.06
38.93
35.50
35.14
25.36
25.12
24.79
22.41
22.23
17.43
14.80
O
n-Pr
H
dr = >20:1
4k
f1 (ppm)

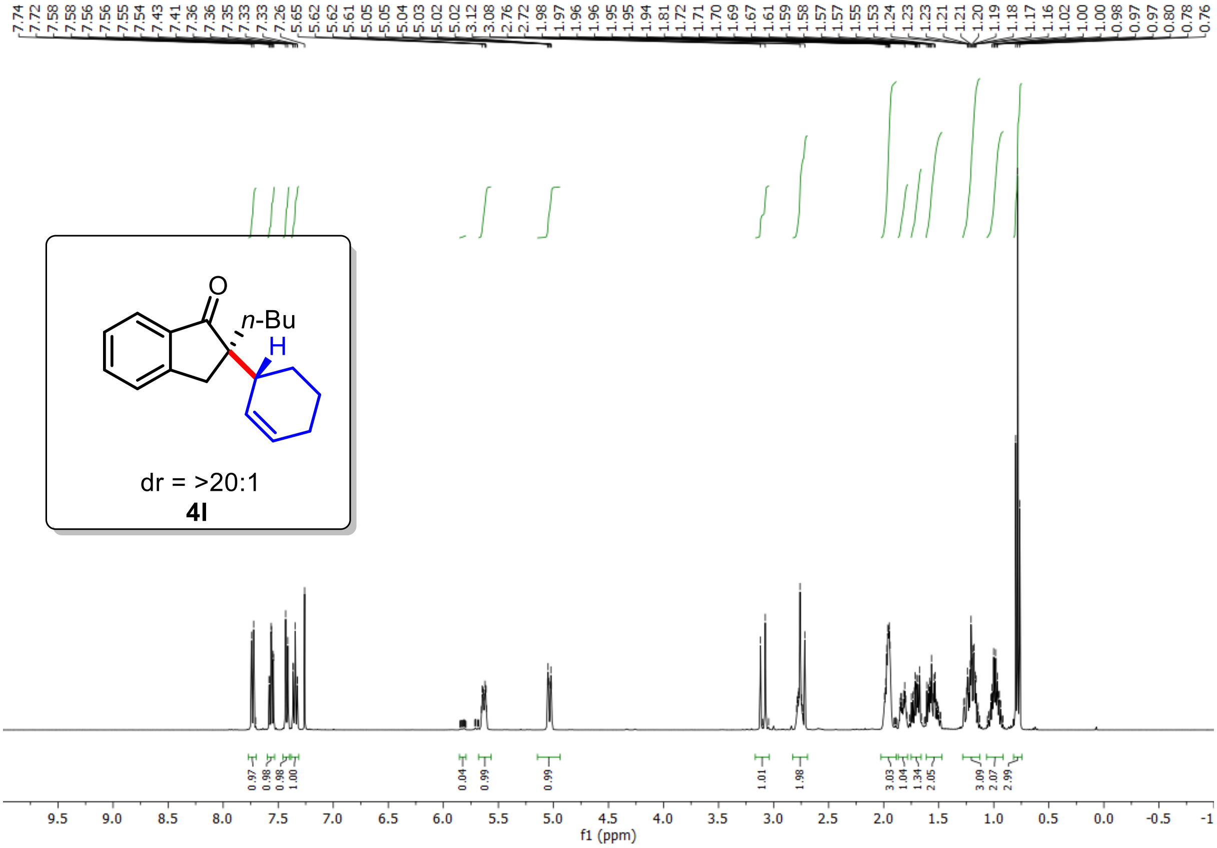
7.74
7.72
7.58
7.58
7.56
7.56
7.55
7.54
7.43
7.41
7.36
7.36
7.35
7.33
7.33
7.26
5.65
5.62
5.62
5.61
5.05
5.05
5.04
5.03
5.02
5.02
3.12
3.08
2.76
2.72
1.98
1.97
1.96
1.96
1.95
1.95
1.94
1.81
1.72
1.71
1.70
1.69
1.67
1.61
1.59
1.58
1.57
1.57
1.55
1.53
1.24
1.23
1.23
1.23
1.21
1.21
1.20
1.19
1.18
1.17
1.16
1.02
1.00
1.00
0.98
0.97
0.97
0.80
0.78
0.76
O
n-Bu
H
dr = >20:1
4l
0.97
0.98
0.98
1.00
0.04
0.99
0.99
1.01
1.98
3.03
1.04
1.34
2.05
3.09
2.07
2.99
f1 (ppm)

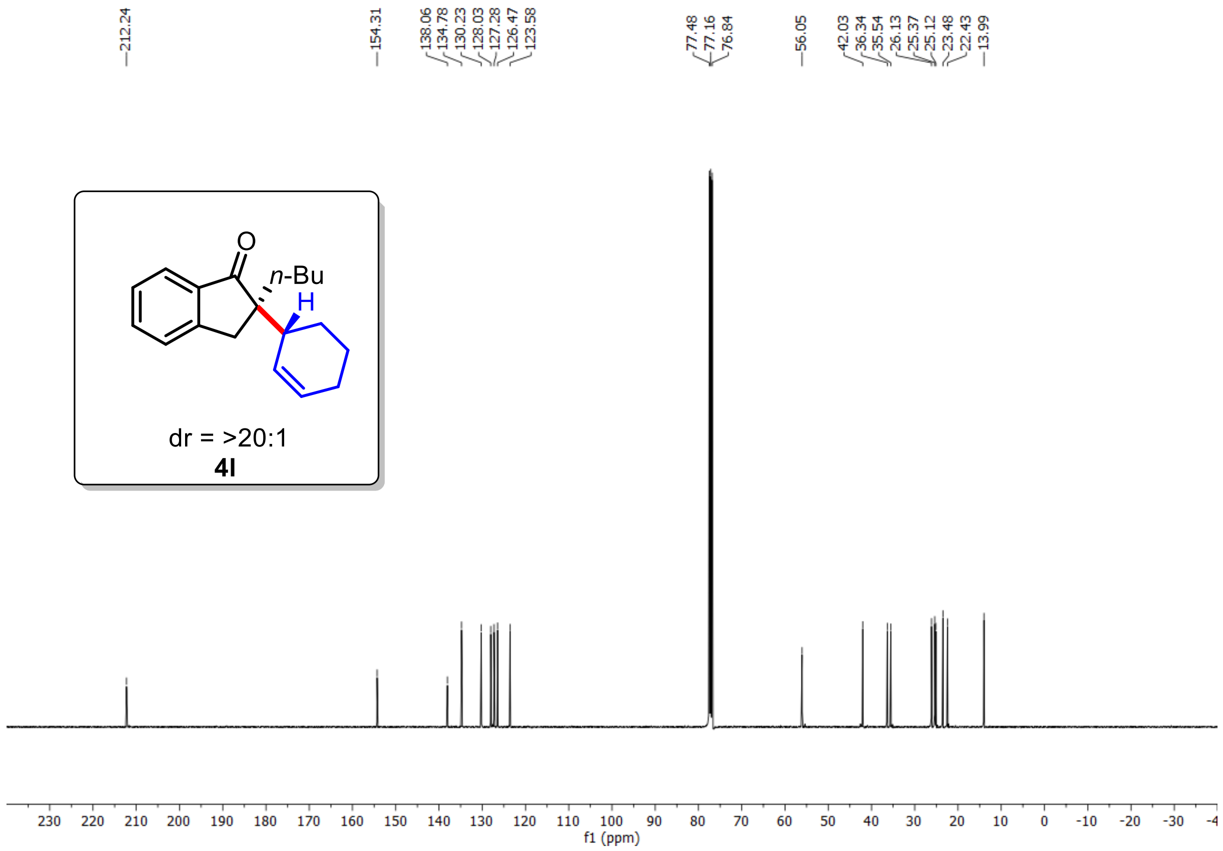
212.24
154.31
138.06
134.78
130.23
128.03
127.28
126.47
123.58
77.48
77.16
76.84
56.05
42.03
36.34
35.54
26.13
25.37
25.12
23.48
22.43
13.99
O
n-Bu
H
dr = >20:1
4l
f1 (ppm)

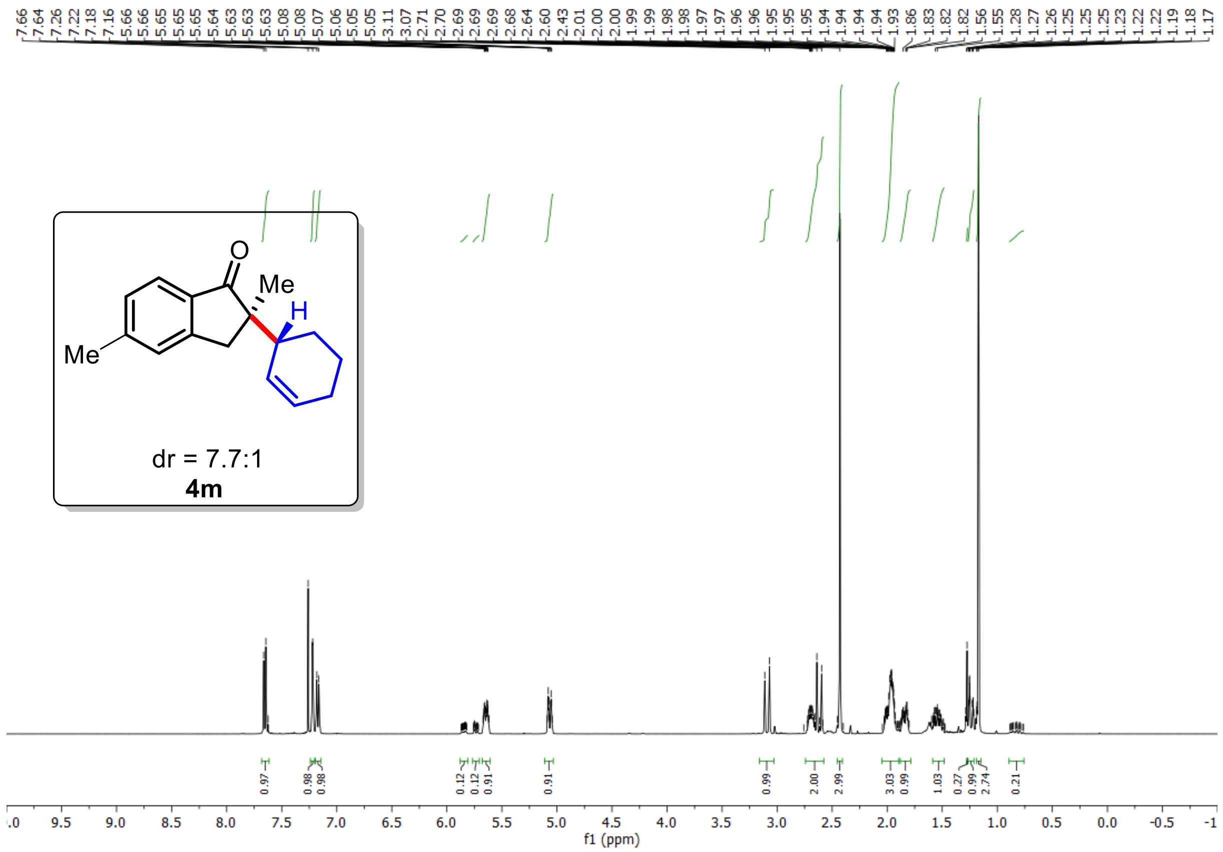
7.66
7.64
7.26
7.22
7.18
7.16
5.66
5.66
5.65
5.65
5.64
5.63
5.63
5.08
5.08
5.07
5.06
5.05
5.05
3.11
3.07
2.71
2.70
2.69
2.69
2.69
2.68
2.64
2.60
2.43
2.01
2.00
2.00
1.99
1.99
1.98
1.98
1.97
1.97
1.96
1.96
1.95
1.95
1.95
1.94
1.94
1.94
1.94
1.93
1.86
1.83
1.82
1.82
1.56
1.55
1.28
1.27
1.26
1.25
1.25
1.25
1.23
1.23
1.22
1.22
1.19
1.18
1.17
O
Me
H
Me
dr = 7.7:1
4m
0.97
0.98
0.98
0.12
0.12
0.91
0.91
0.99
2.00
2.99
3.03
0.99
1.03
0.27
0.99
2.74
0.21
f1 (ppm)

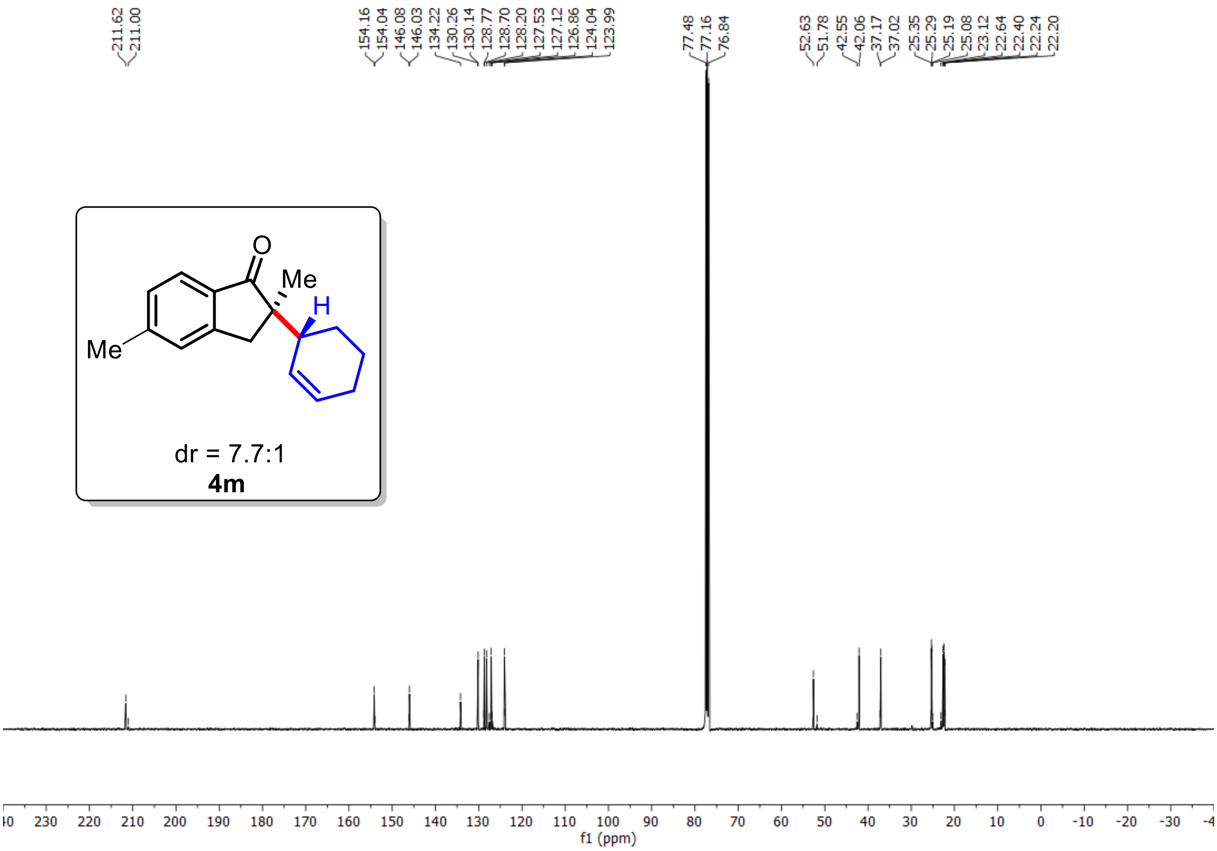
211.62
211.00
154.16
154.04
146.08
146.03
134.22
130.26
130.14
128.77
128.70
128.20
127.53
127.12
126.86
124.04
123.99
77.48
77.16
76.84
52.63
51.78
42.55
42.06
37.17
37.02
25.35
25.29
25.19
25.08
23.12
22.64
22.40
22.24
22.20
O
Me
H
Me
dr = 7.7:1
4m
f1 (ppm)

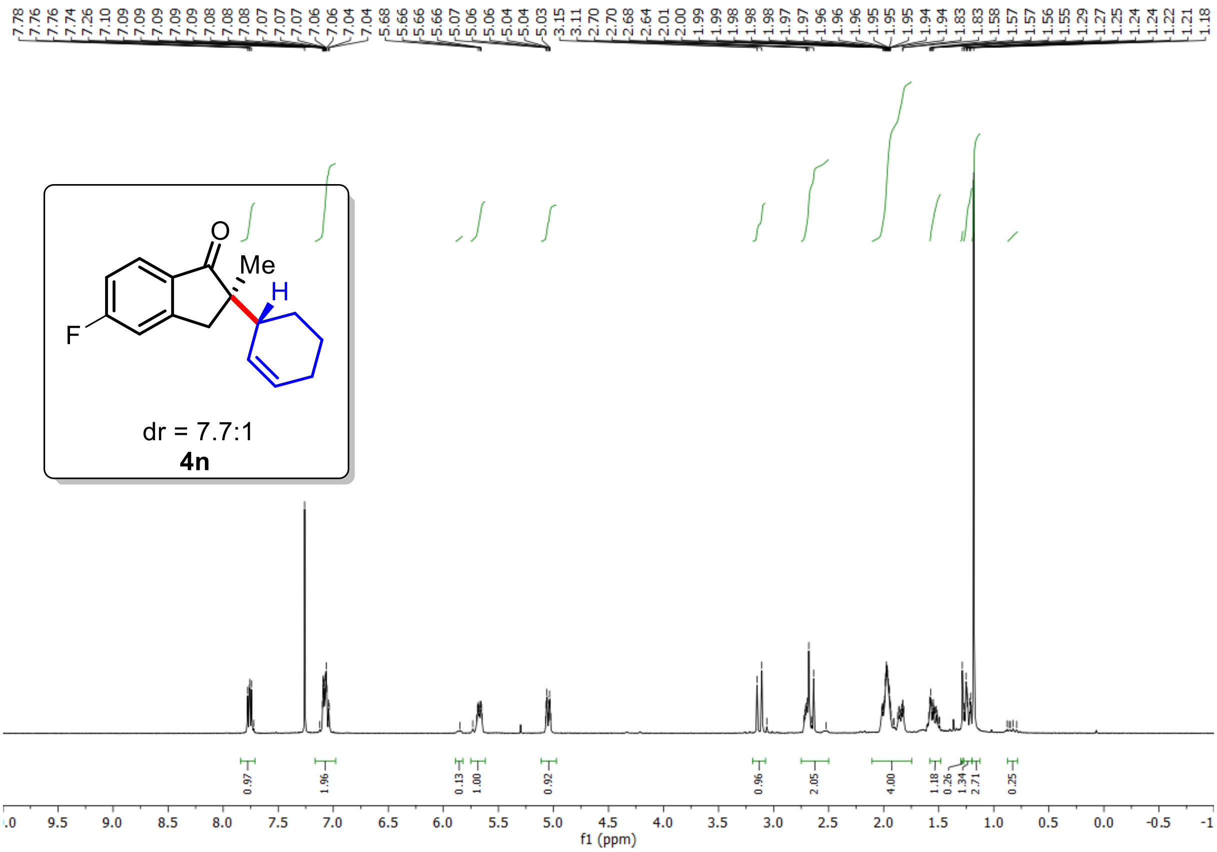
7.78
7.76
7.74
7.26
7.10
7.09
7.09
7.09
7.09
7.09
7.08
7.08
7.07
7.07
7.07
7.06
7.06
7.04
7.04
5.68
5.66
5.66
5.07
5.06
5.06
5.04
5.04
5.03
3.15
3.11
2.70
2.70
2.68
2.64
2.01
2.00
1.99
1.99
1.98
1.98
1.97
1.97
1.96
1.96
1.96
1.95
1.95
1.95
1.94
1.94
1.83
1.83
1.58
1.57
1.57
1.56
1.55
1.29
1.27
1.25
1.24
1.24
1.22
1.21
1.18
O
Me
H
F
dr = 7.7:1
4n
0.97
1.96
0.13
1.00
0.92
0.96
2.05
4.00
1.18
0.26
1.34
2.71
0.25
f1 (ppm)

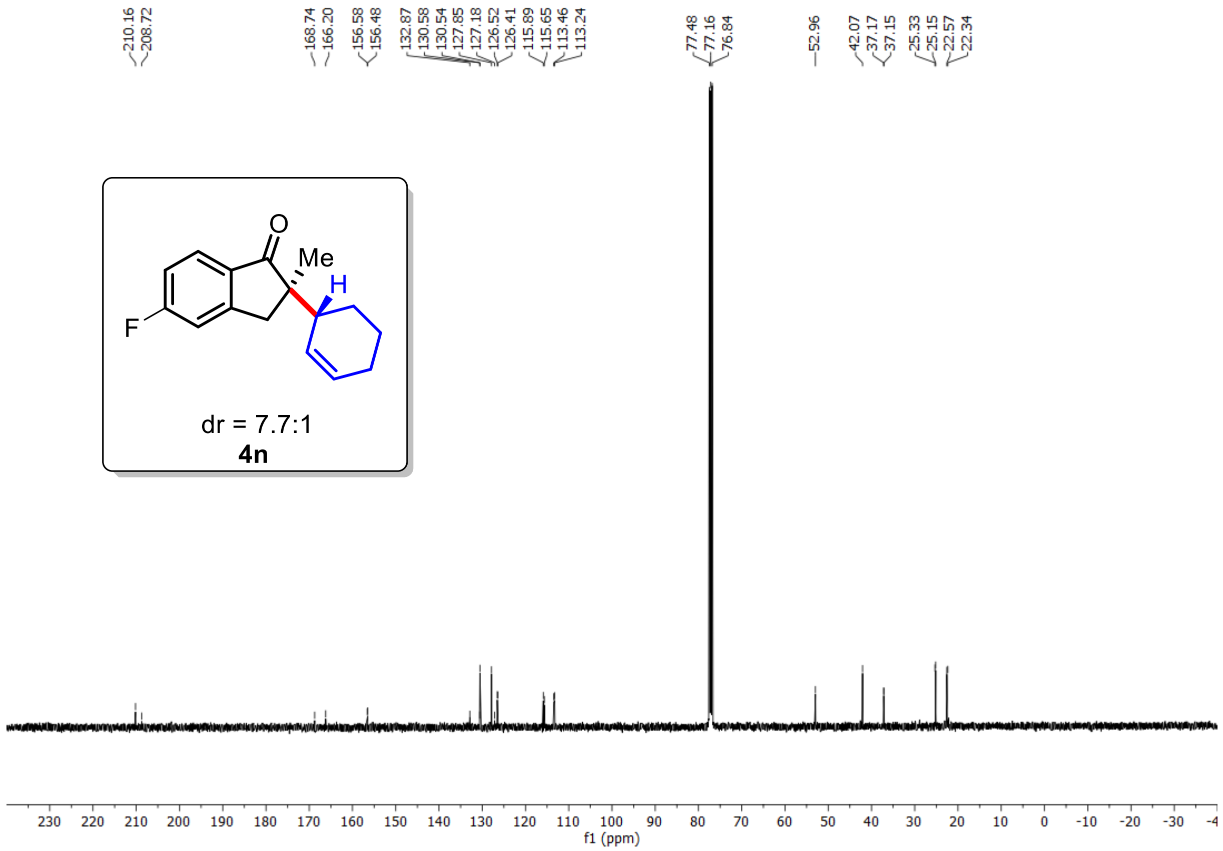

210.16
208.72
168.74
166.20
156.58
156.48
132.87
130.58
130.54
127.85
127.18
126.52
126.41
115.89
115.65
113.46
113.24
77.48
77.16
76.84
52.96
42.07
37.17
37.15
25.33
25.15
22.57
22.34
Me
H
O
F
dr = 7.7:1
4n
f1 (ppm)

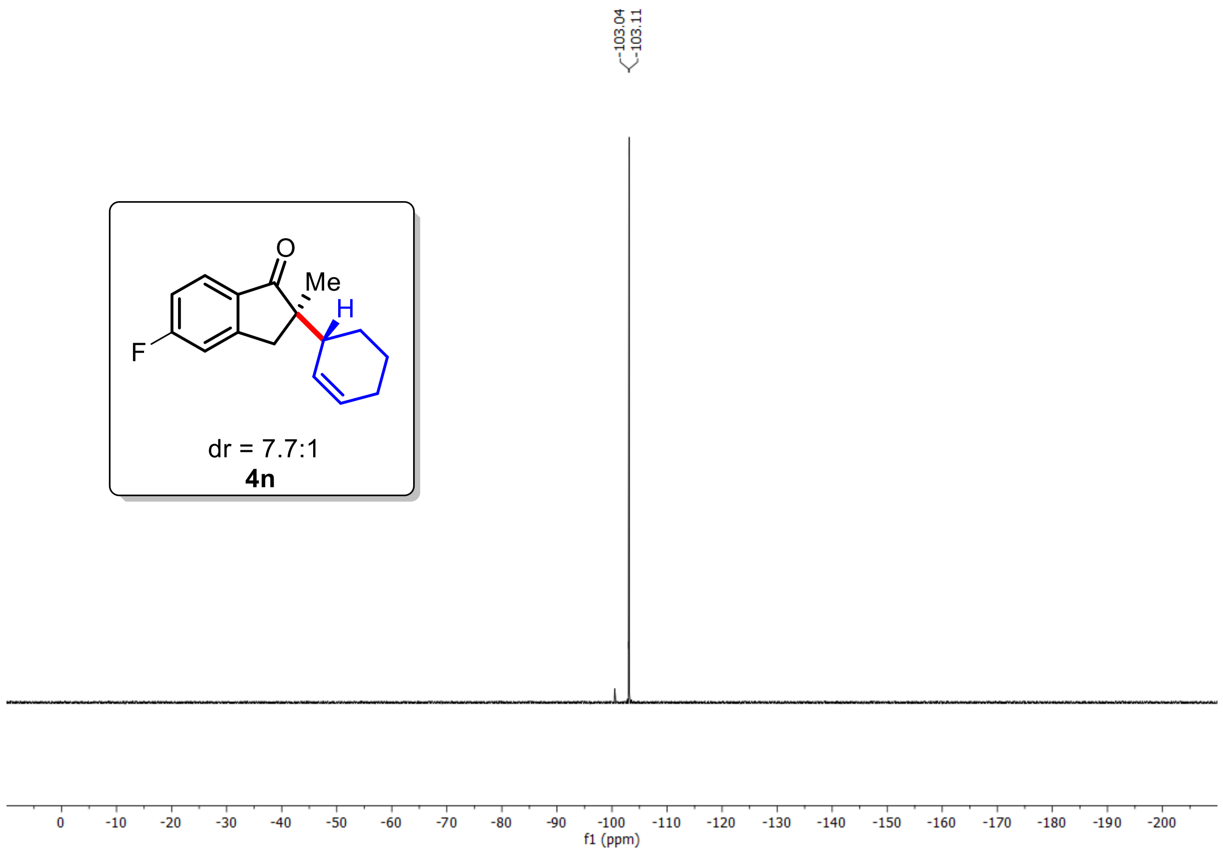

-103.04
-103.11
Me
H
O
F
dr = 7.7:1
4n
f1 (ppm)

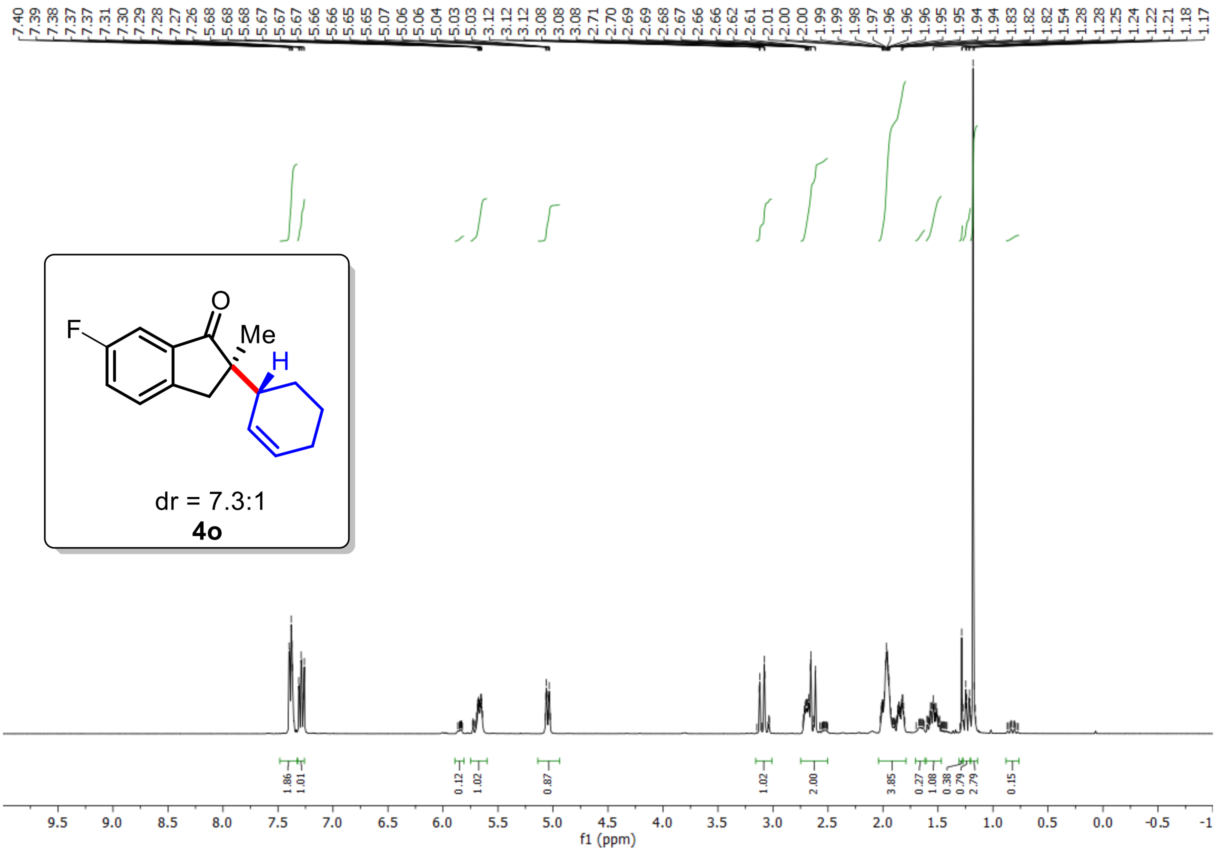
7.40 7.39 7.38 7.37 7.37 7.31 7.30 7.29 7.28 7.27 7.26 5.68 5.68 5.68 5.67 5.67 5.67 5.66 5.66 5.65 5.65 5.07 5.06 5.06 5.04 5.03 5.03 3.12 3.12 3.12 3.08 3.08 3.08 2.71 2.70 2.69 2.69 2.68 2.67 2.66 2.66 2.62 2.62 2.61 2.01 2.00 2.00 1.99 1.99 1.98 1.97 1.96 1.96 1.96 1.95 1.95 1.94 1.94 1.83 1.82 1.82 1.54 1.28 1.28 1.25 1.24 1.22 1.21 1.18 1.17
F
O
Me
H
dr = 7.3:1
4o
1.86 1.01 0.12 1.02 0.87 1.02 2.00 3.85 0.27 1.08 0.38 0.79 2.79 0.15
9.5 9.0 8.5 8.0 7.5 7.0 6.5 6.0 5.5 5.0 4.5 4.0 3.5 3.0 2.5 2.0 1.5 1.0 0.5 0.0 -0.5 -1
f1 (ppm)

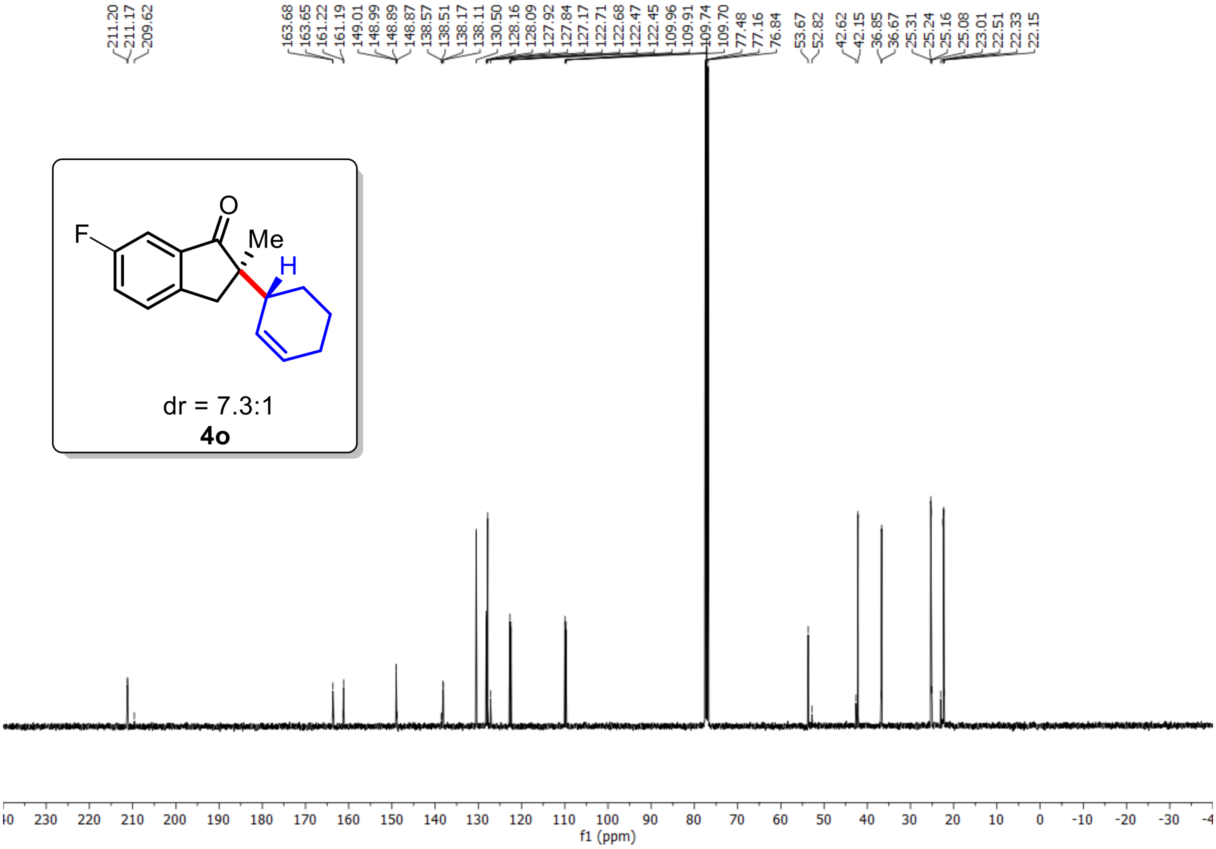
211.20 211.17 209.62 163.68 163.65 161.22 161.19 149.01 148.99 148.89 148.87 138.57 138.51 138.17 138.11 130.50 128.16 128.09 127.92 127.84 127.17 122.71 122.68 122.47 122.45 109.96 109.91 109.74 109.70 77.48 77.16 76.84 53.67 52.82 42.62 42.15 36.85 36.67 25.31 25.24 25.16 25.08 23.01 22.51 22.33 22.15
F
O
Me
H
dr = 7.3:1
4o
230 220 210 200 190 180 170 160 150 140 130 120 110 100 90 80 70 60 50 40 30 20 10 0 -10 -20 -30
f1 (ppm)

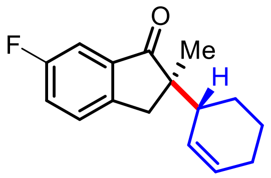

dr = 7.3:1
4o

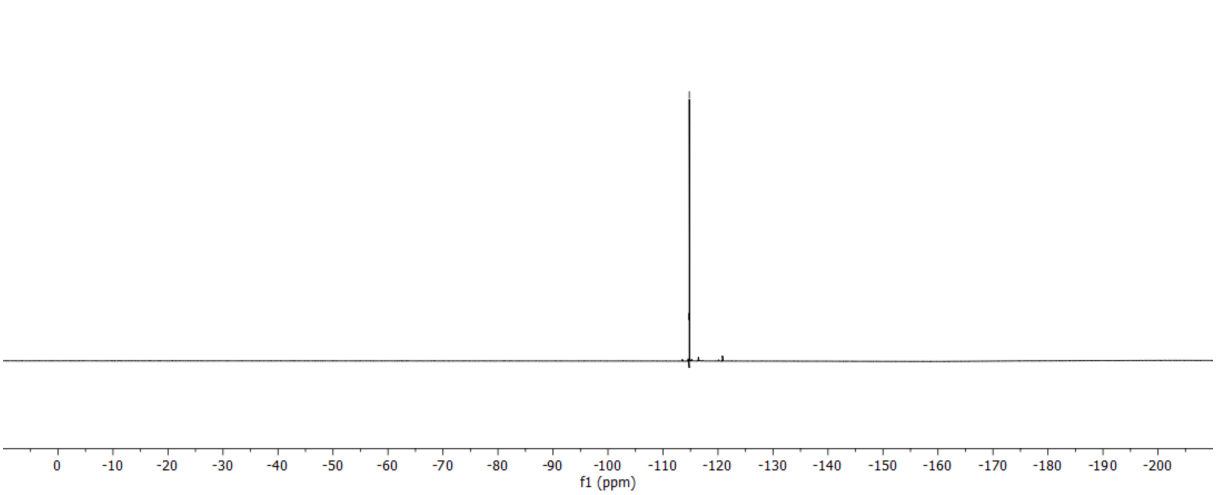

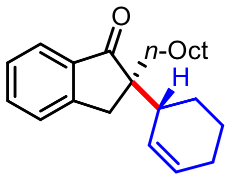

dr = >20:1
4p

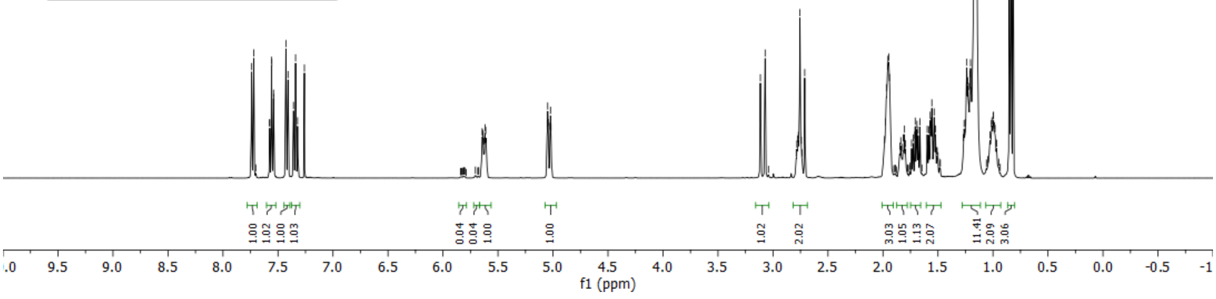

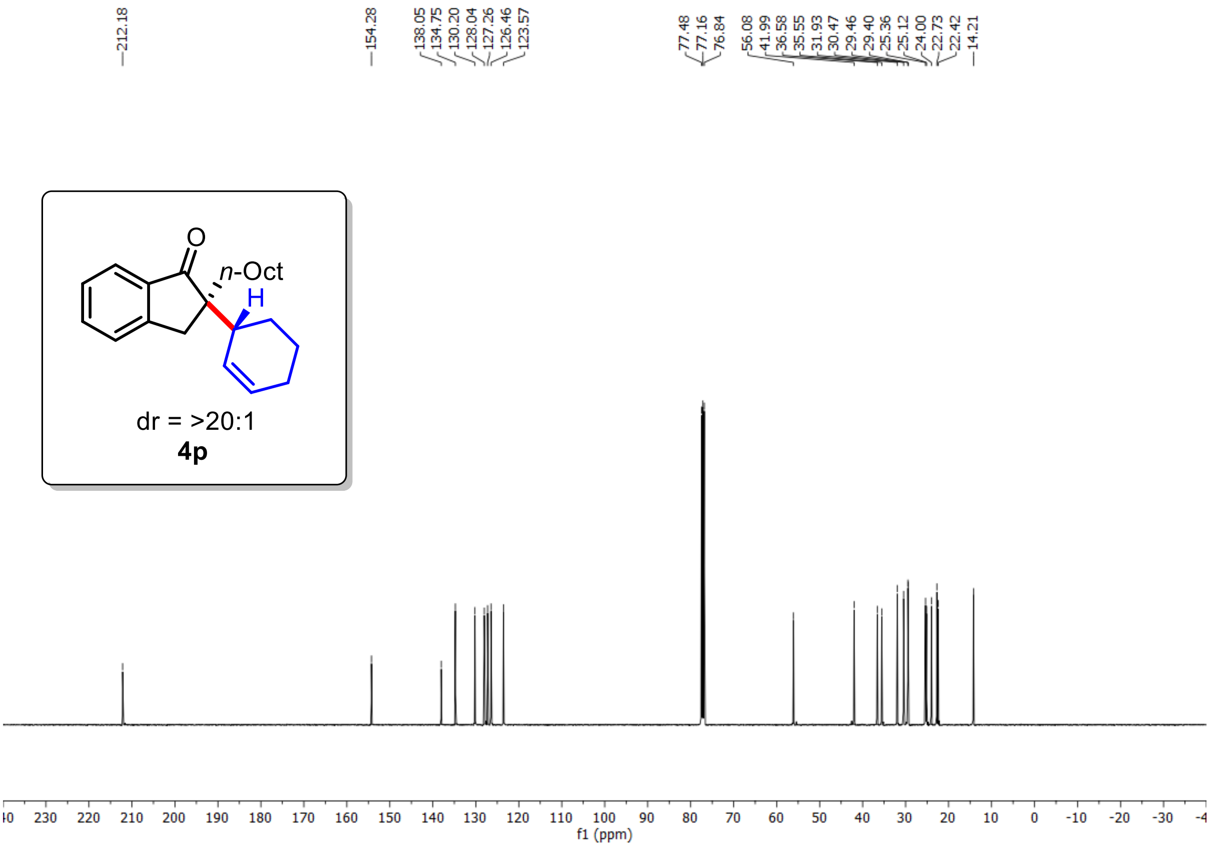
212.18
154.28
138.05
134.75
130.20
128.04
127.26
126.46
123.57
77.48
77.16
76.84
56.08
41.99
36.58
35.55
31.93
30.47
29.46
29.40
25.36
25.12
24.00
22.73
22.42
14.21
O
n-Oct
H
dr = >20:1
4p
f1 (ppm)

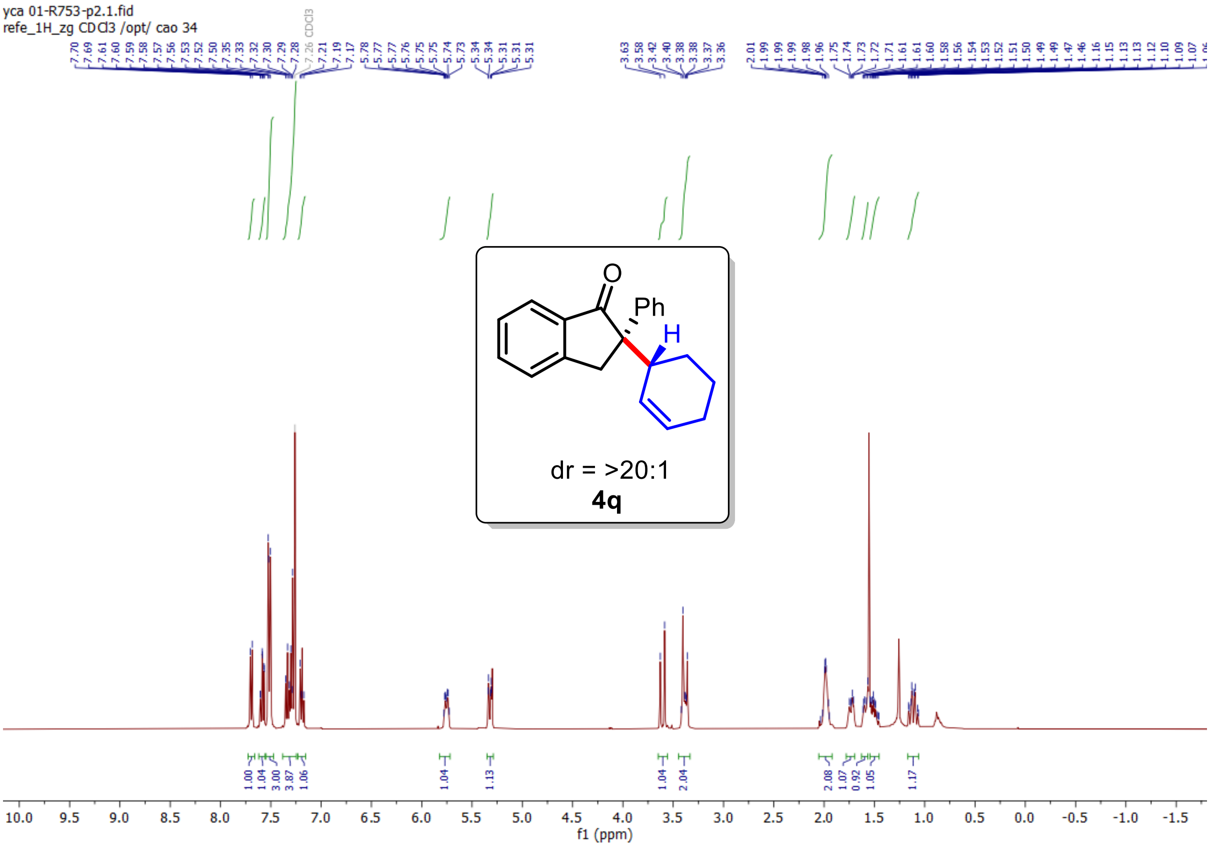
yca 01-R753-p2.1.fid
refe_1H_zg CDCl3 /opt/ cao 34
7.70
7.69
7.61
7.60
7.59
7.58
7.57
7.56
7.53
7.52
7.50
7.35
7.33
7.32
7.30
7.29
7.28
7.26 CDCl3
7.21
7.19
7.17
5.78
5.77
5.76
5.75
5.75
5.74
5.73
5.34
5.34
5.31
5.31
3.63
3.58
3.42
3.40
3.38
3.38
3.37
3.36
2.01
1.99
1.99
1.98
1.96
1.75
1.74
1.73
1.72
1.71
1.61
1.60
1.58
1.56
1.54
1.53
1.52
1.51
1.50
1.49
1.47
1.46
1.16
1.15
1.13
1.13
1.12
1.10
1.09
1.07
O
Ph
H
dr = >20:1
4q
1.00
1.04
3.00
3.87
1.06
1.04
1.13
1.04
2.04
2.08
1.07
0.92
1.05
1.17
f1 (ppm)

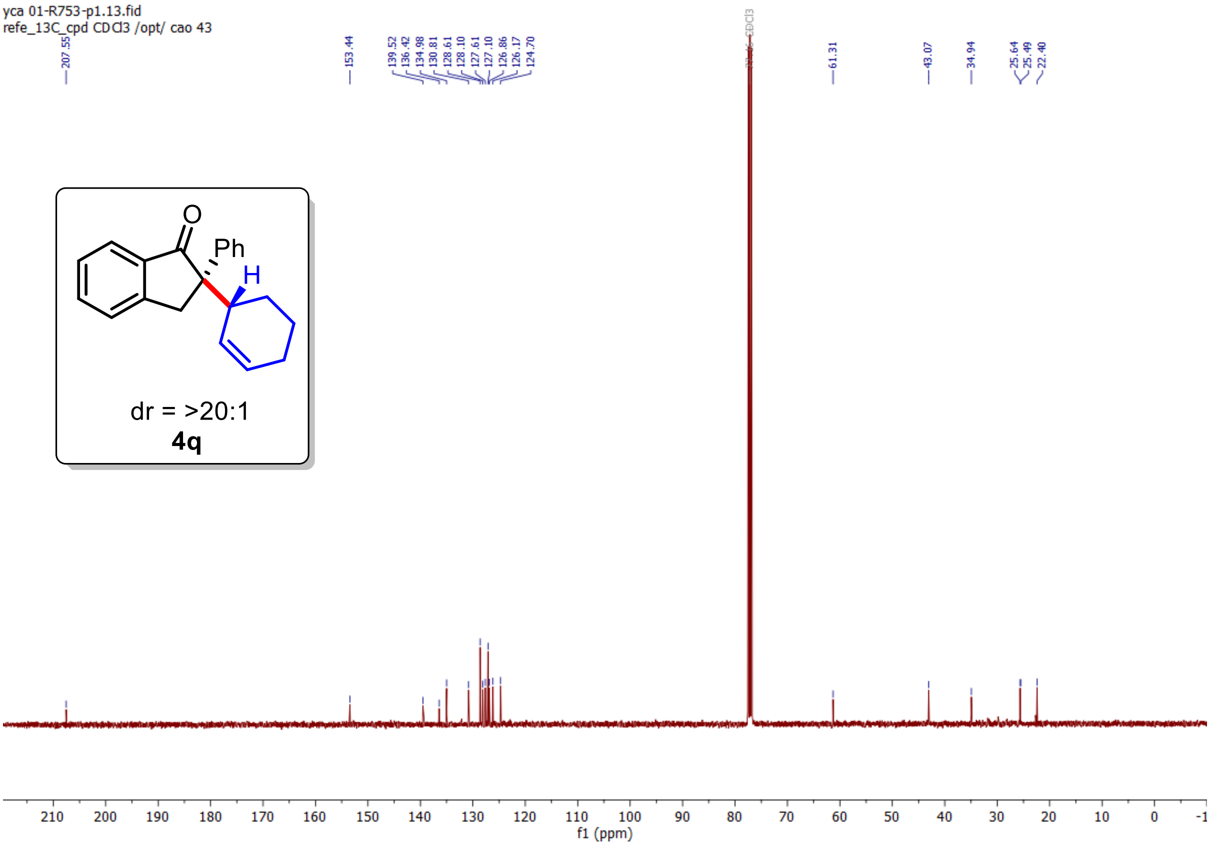
yca 01-R753-p1.13.fid
refe_13C_cpd CDCl3 /opt/ cao 43
207.55
153.44
139.52
136.42
134.98
130.81
128.61
128.10
127.61
127.10
126.86
126.17
124.70
61.31
43.07
34.94
25.64
25.49
22.40
Ph
H
O
dr = >20:1
4q
f1 (ppm)

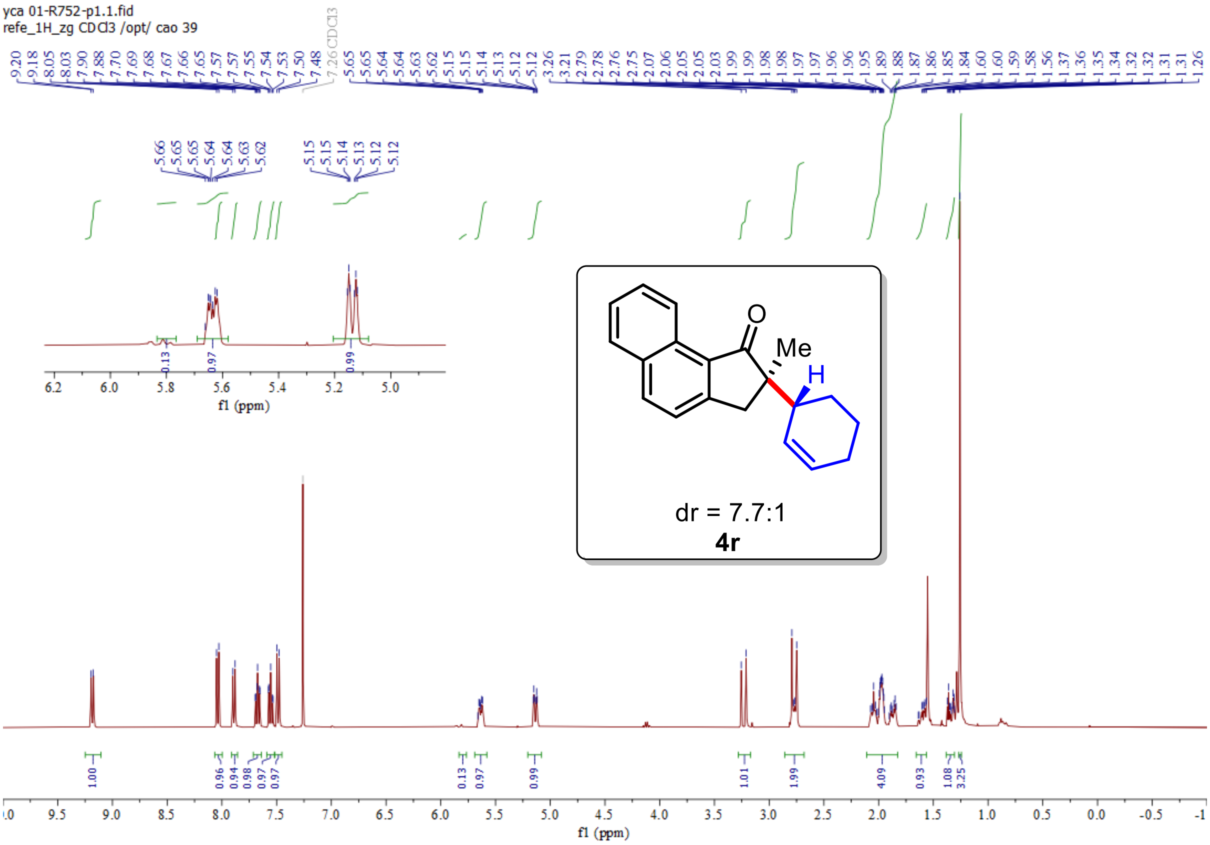
yca 01-R752-p1.1.fid
refe_1H_zg CDCl3 /opt/ cao 39
O
Me
H
dr = 7.7:1
4r
f1 (ppm)

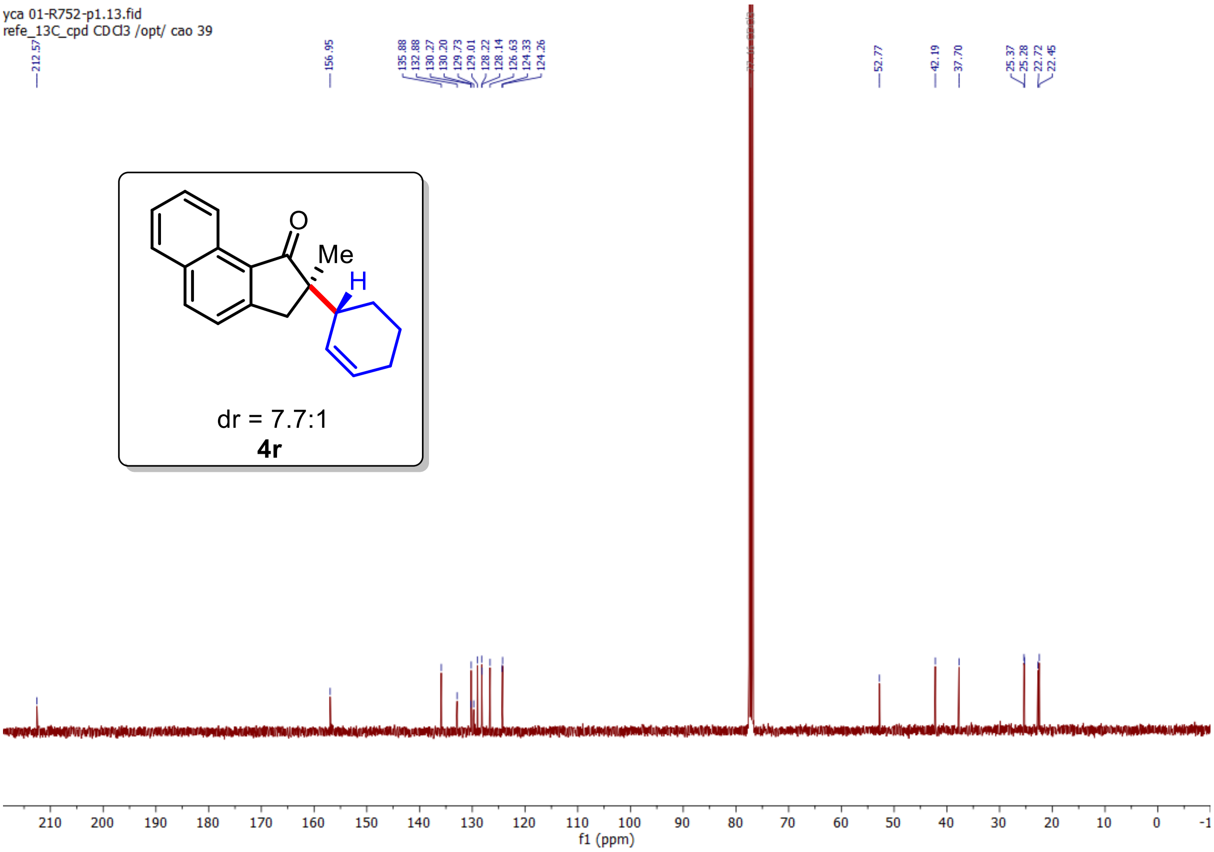
yca 01-R752-p1.13.fid
refe_13C_cpd CDCl3 /opt/ cao 39
212.57
156.95
135.88
132.88
130.27
130.20
129.73
129.01
128.22
128.14
126.63
124.33
124.26
52.77
42.19
37.70
25.37
25.28
22.72
22.45
O
Me
H
dr = 7.7:1
4r
210 200 190 180 170 160 150 140 130 120 110 100 90 80 70 60 50 40 30 20 10 0 -1
f1 (ppm)

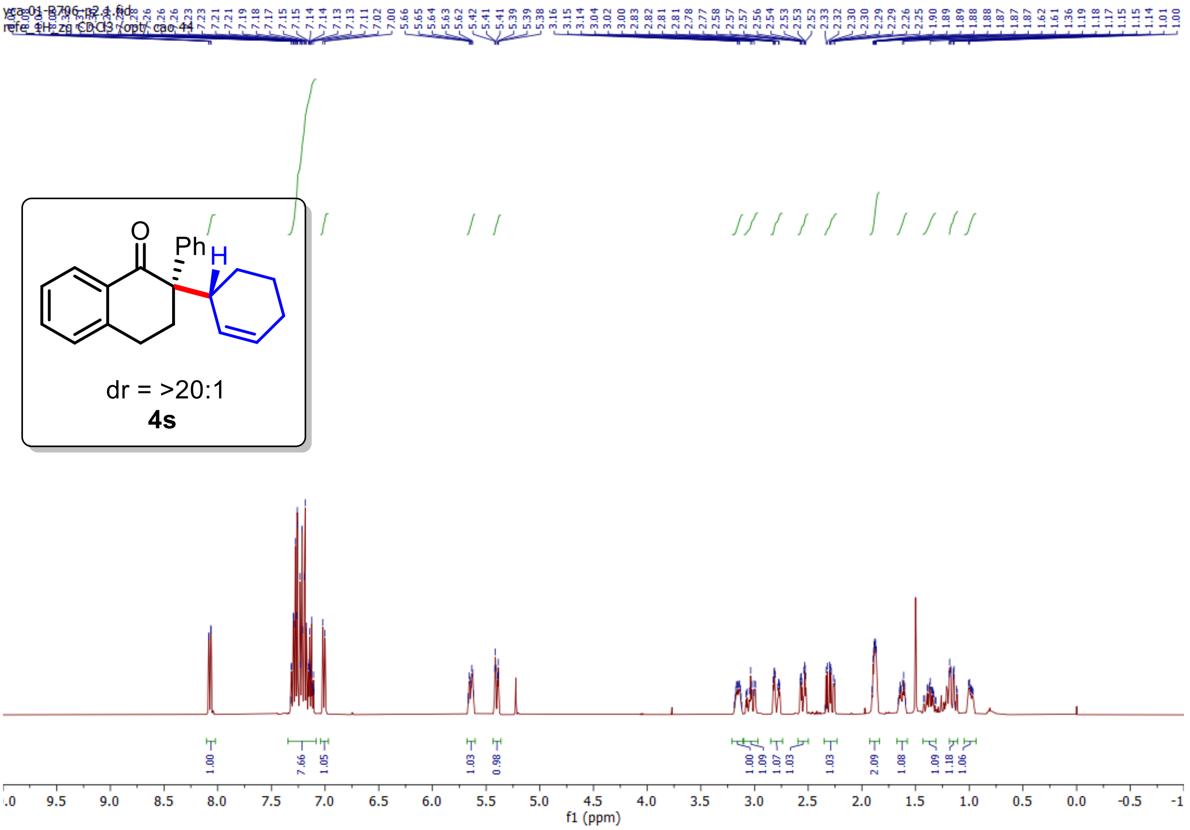

yca 01-R706-p2.1.fid
refe_1H_zg CDCl3 /opt/ cao 44
O
Ph
H
dr = >20:1
4s
f1 (ppm)

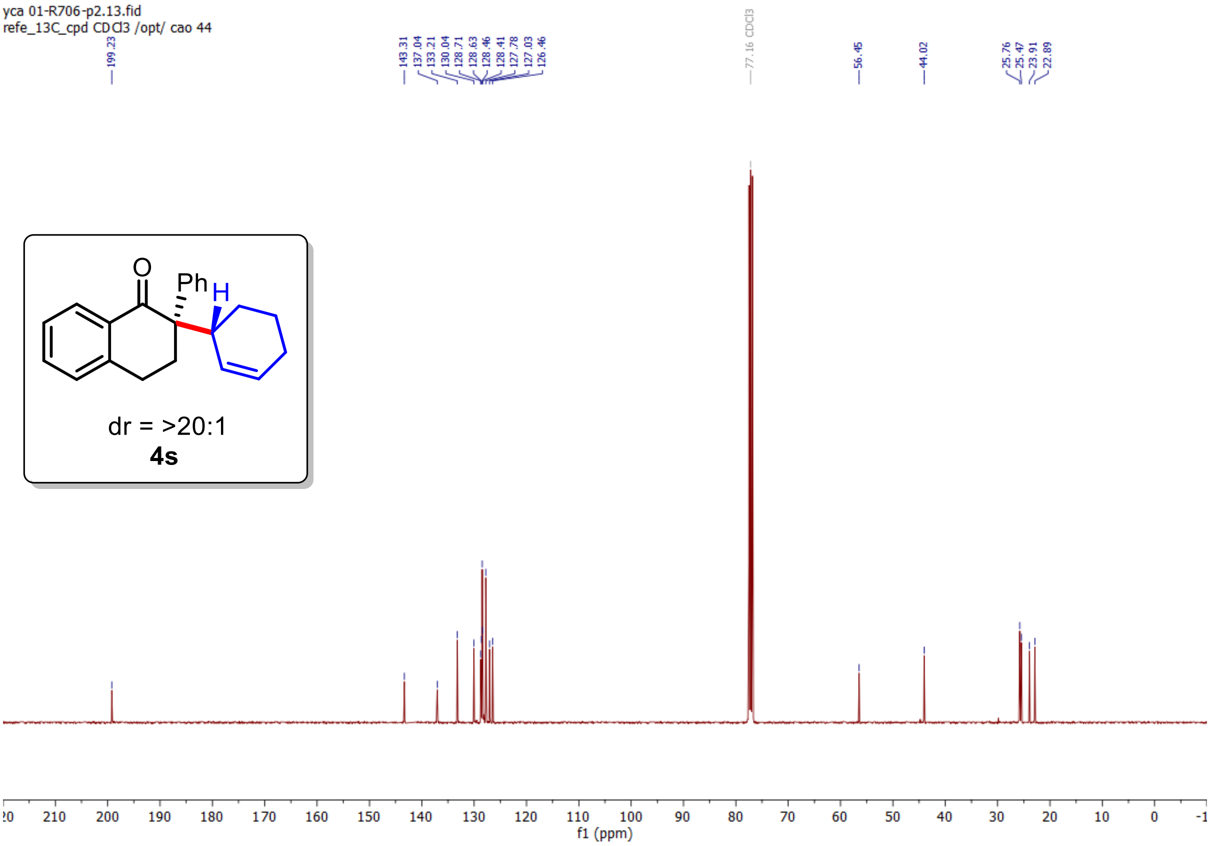

yca 01-R706-p2.13.fid
refe_13C_cpd CDCl3 /opt/ cao 44
199.23
143.31
137.04
133.21
130.04
128.71
128.63
128.46
128.41
127.78
127.03
126.46
77.16 CDCl3
56.45
44.02
25.76
25.47
23.91
22.89
O
Ph
H
dr = >20:1
4s
f1 (ppm)

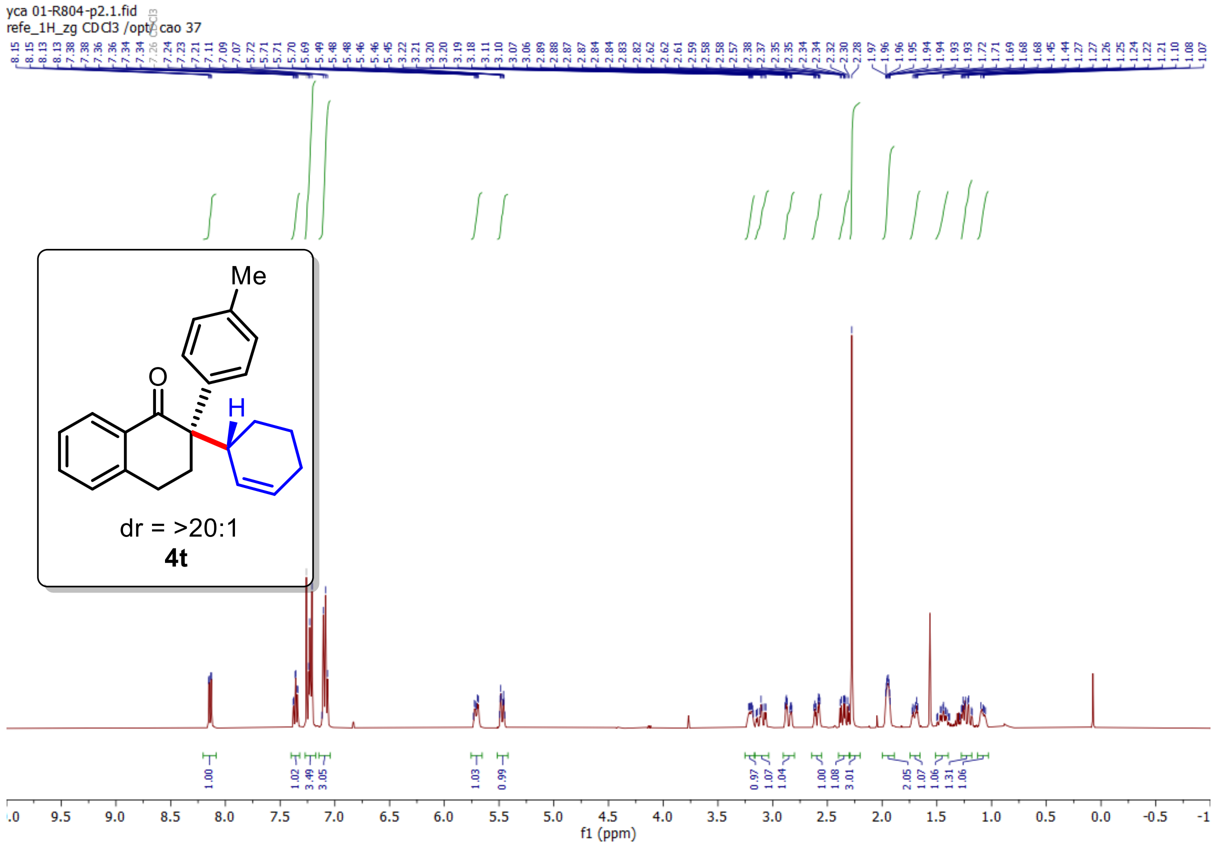
yca 01-R804-p2.1.fid
refe_1H_zg CDCl3 /opt/ cao 37
8.15 8.15 8.13 8.13 7.38 7.38 7.36 7.36 7.34 7.34 7.26 CDCl3 7.24 7.23 7.21 7.11 7.09 7.07 5.72 5.71 5.71 5.70 5.69 5.49 5.48 5.48 5.46 5.46 5.45 3.22 3.21 3.20 3.20 3.19 3.18 3.11 3.10 3.07 3.06 2.89 2.88 2.87 2.84 2.83 2.82 2.62 2.61 2.59 2.58 2.58 2.57 2.38 2.37 2.35 2.35 2.34 2.34 2.32 2.30 2.28 1.97 1.96 1.96 1.95 1.94 1.94 1.93 1.93 1.72 1.71 1.69 1.68 1.68 1.45 1.44 1.27 1.27 1.26 1.25 1.24 1.22 1.21 1.10 1.08 1.07
Me
O
H
dr = >20:1
4t
1.00 1.02 3.49 3.05 1.03 0.99 0.97 1.07 1.04 1.00 1.08 3.01 2.05 1.07 1.06 1.31 1.06
f1 (ppm)

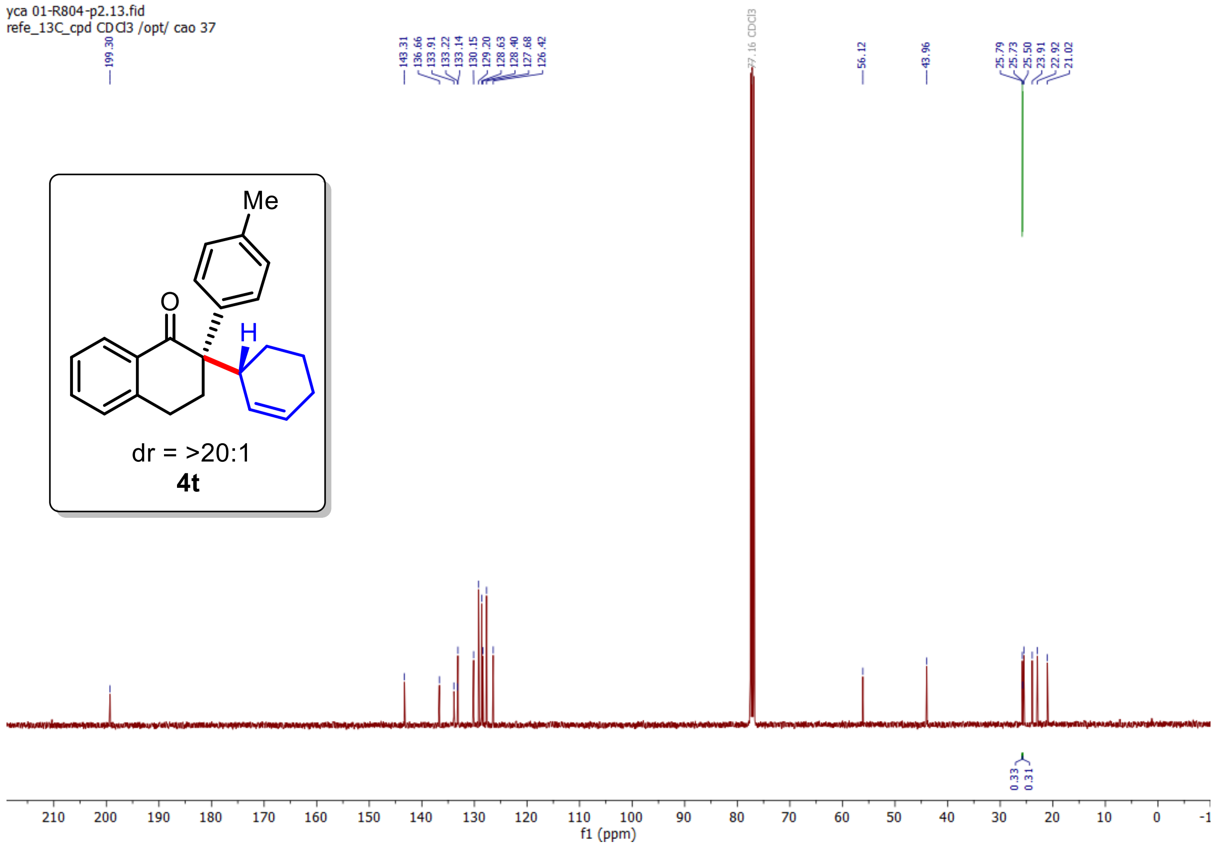
yca 01-R804-p2.13.fid
refe_13C_cpd CDCl3 /opt/ cao 37
199.30
143.31 136.66 133.91 133.22 133.14 130.15 129.20 128.63 128.40 127.68 126.42
77.16 CDCl3
56.12
43.96
25.79 25.73 25.50 23.91 22.92 21.02
Me
O
H
dr = >20:1
4t
0.33 0.31
f1 (ppm)

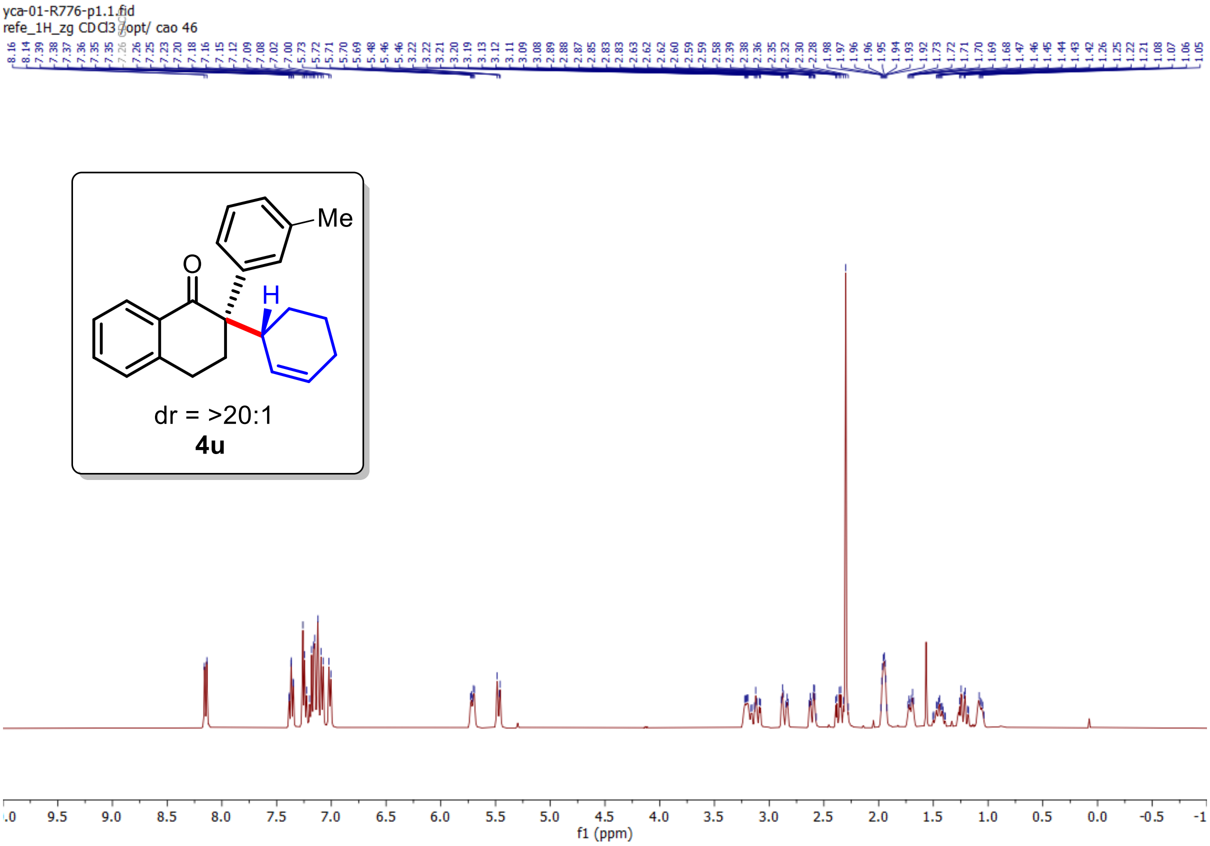
yca-01-R776-p1.1.fid
refe_1H_zg CDCl3 /opt/ cao 46
8.16 8.14 7.39 7.38 7.37 7.36 7.35 7.35 7.26 7.26 7.25 7.23 7.20 7.18 7.16 7.15 7.12 7.09 7.08 7.02 7.00 5.73 5.72 5.71 5.70 5.69 5.48 5.46 5.46 3.22 3.22 3.21 3.20 3.19 3.13 3.12 3.11 3.09 3.08 2.89 2.88 2.87 2.85 2.83 2.83 2.63 2.62 2.62 2.60 2.59 2.59 2.58 2.39 2.38 2.36 2.35 2.32 2.30 2.28 1.98 1.97 1.96 1.95 1.94 1.93 1.92 1.73 1.72 1.71 1.70 1.69 1.68 1.47 1.46 1.45 1.44 1.43 1.42 1.26 1.25 1.22 1.21 1.08 1.07 1.06 1.05
Me
O
H
dr = >20:1
4u
f1 (ppm)

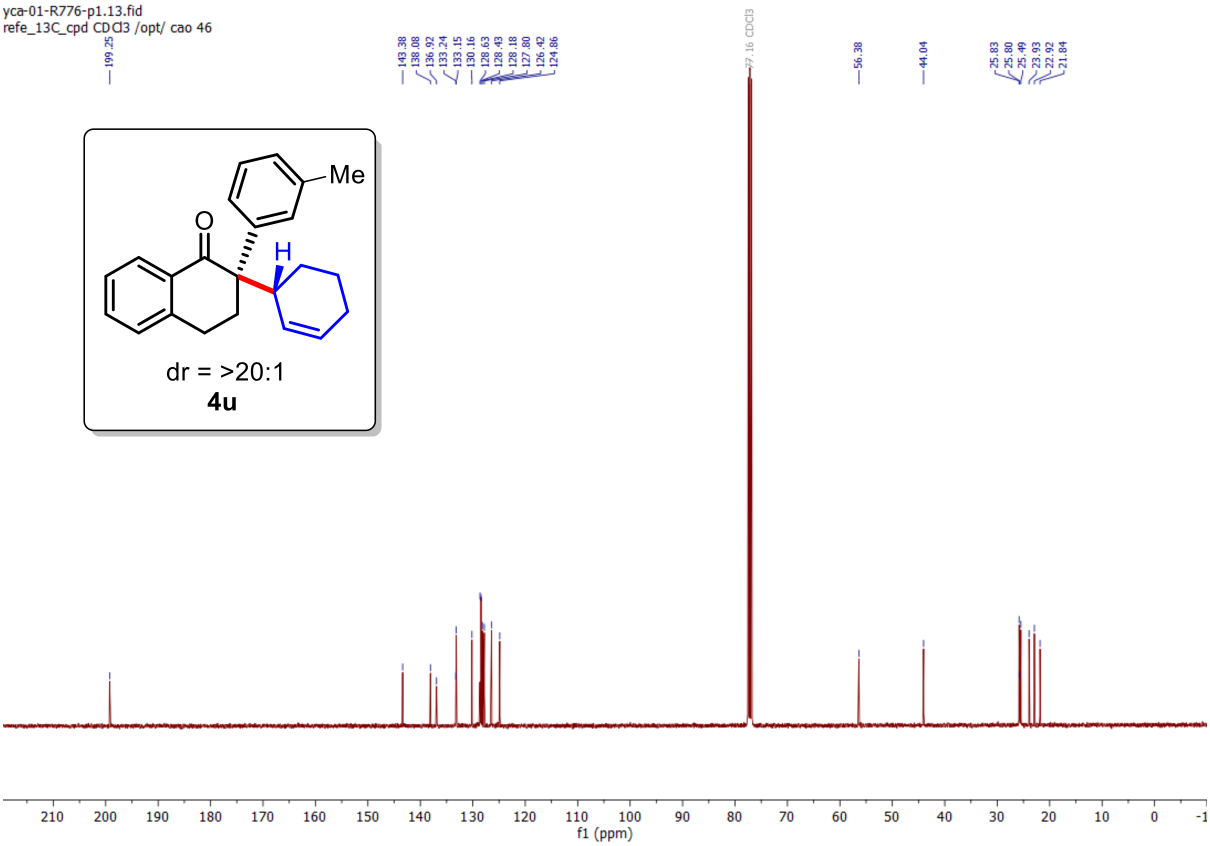
yca-01-R776-p1.13.fid
refe_13C_cpd CDCl3 /opt/ cao 46
199.25
143.38 138.08 136.92 133.24 133.15 130.16 128.63 128.43 128.18 127.80 126.42 124.86
77.16 CDCl3
56.38
44.04
25.83 25.80 25.49 23.93 22.92 21.84
Me
O
H
dr = >20:1
4u
f1 (ppm)

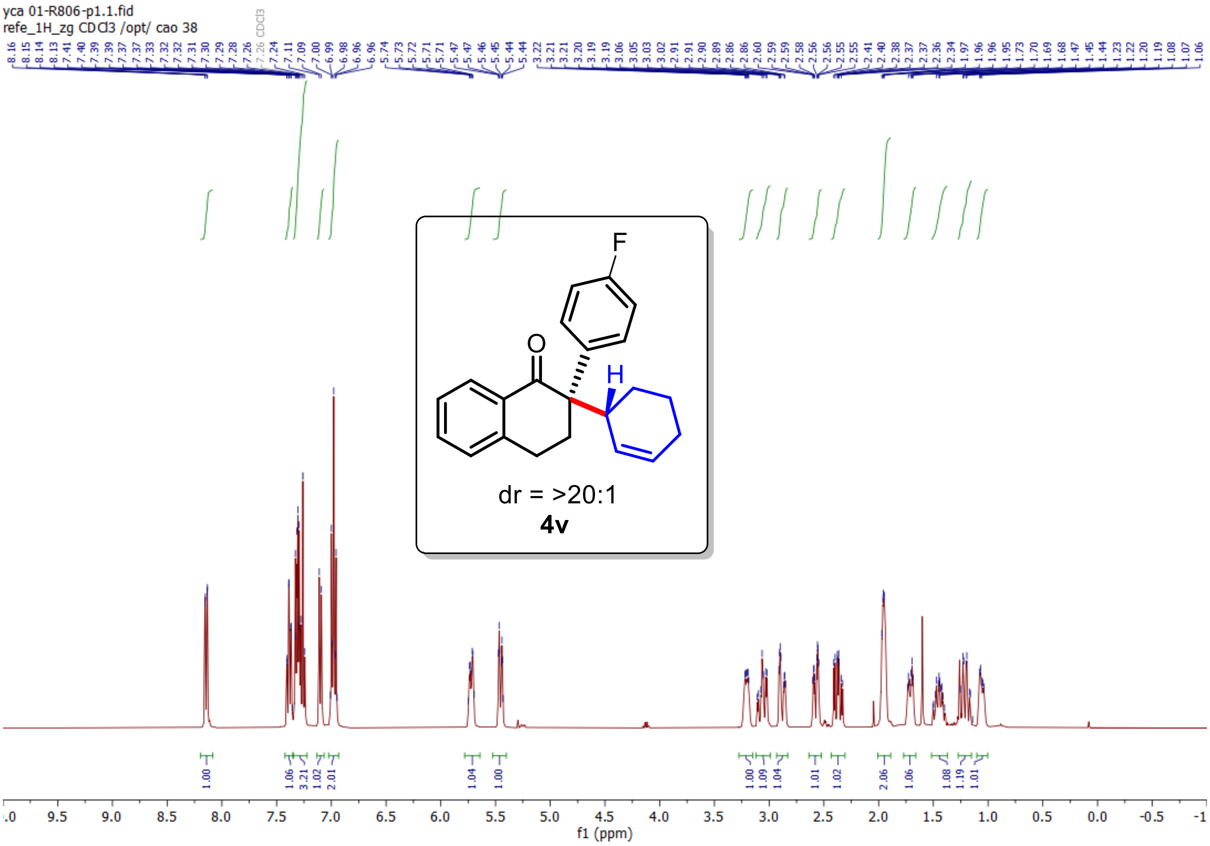
yca 01-R806-p1.1.fid
refe_1H_zg CDCl3 /opt/ cao 38
CDCl3
F
O
H
dr = >20:1
4v
f1 (ppm)

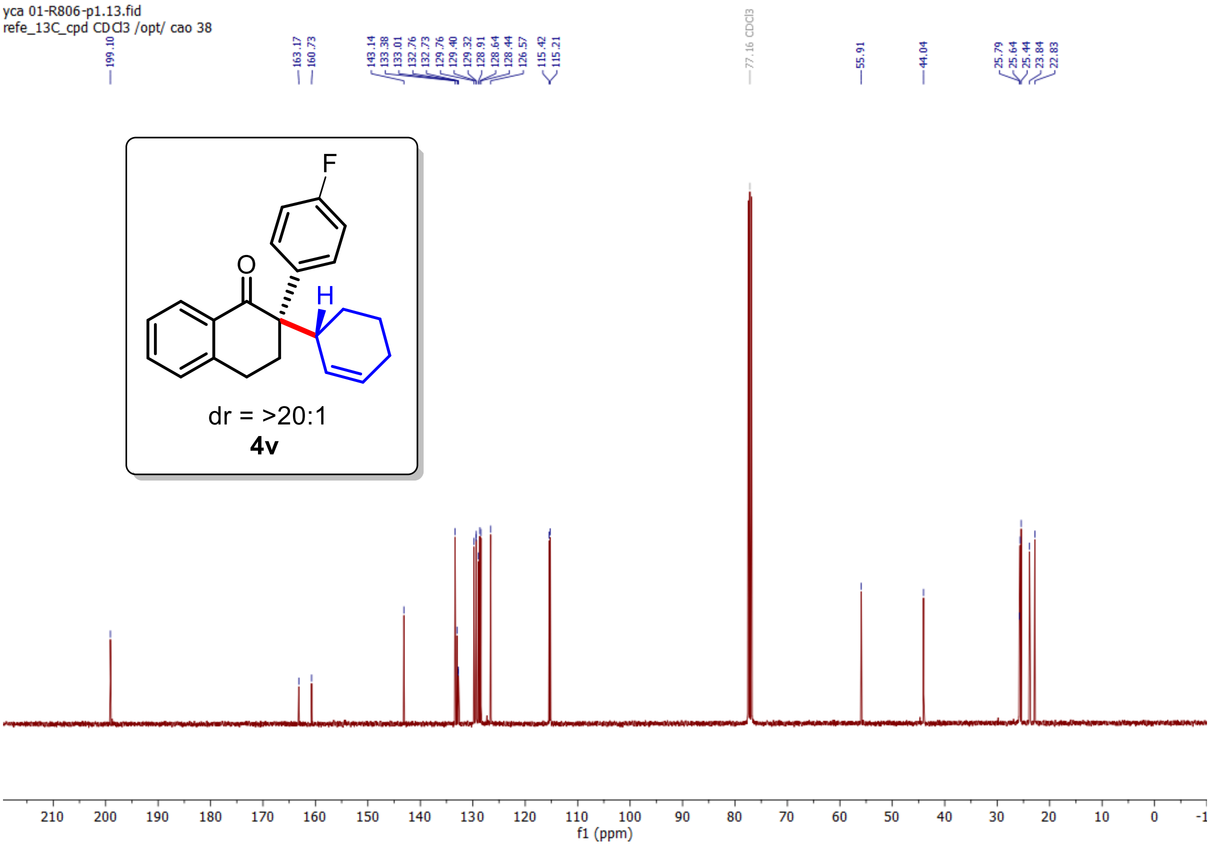
yca 01-R806-p1.13.fid
refe_13C_cpd CDCl3 /opt/ cao 38
199.10
163.17
160.73
143.14
133.38
133.01
132.76
132.73
129.76
129.40
129.32
128.91
128.64
128.44
126.57
115.42
115.21
77.16 CDCl3
55.91
44.04
25.79
25.64
25.44
23.84
22.83
F
O
H
dr = >20:1
4v
f1 (ppm)

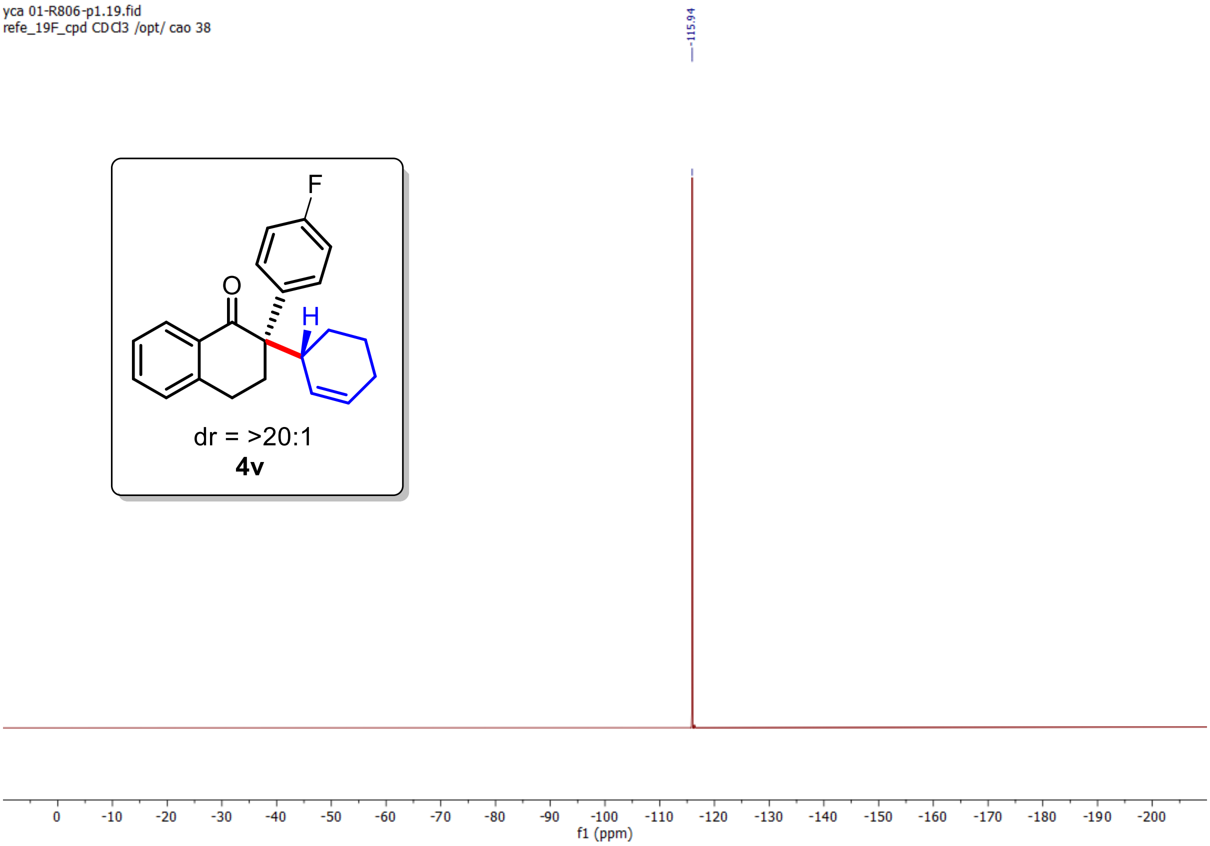
yca 01-R806-p1.19.fid
refe_19F_cpd CDCl3 /opt/ cao 38
-115.94
F
O
H
dr = >20:1
4v
f1 (ppm)

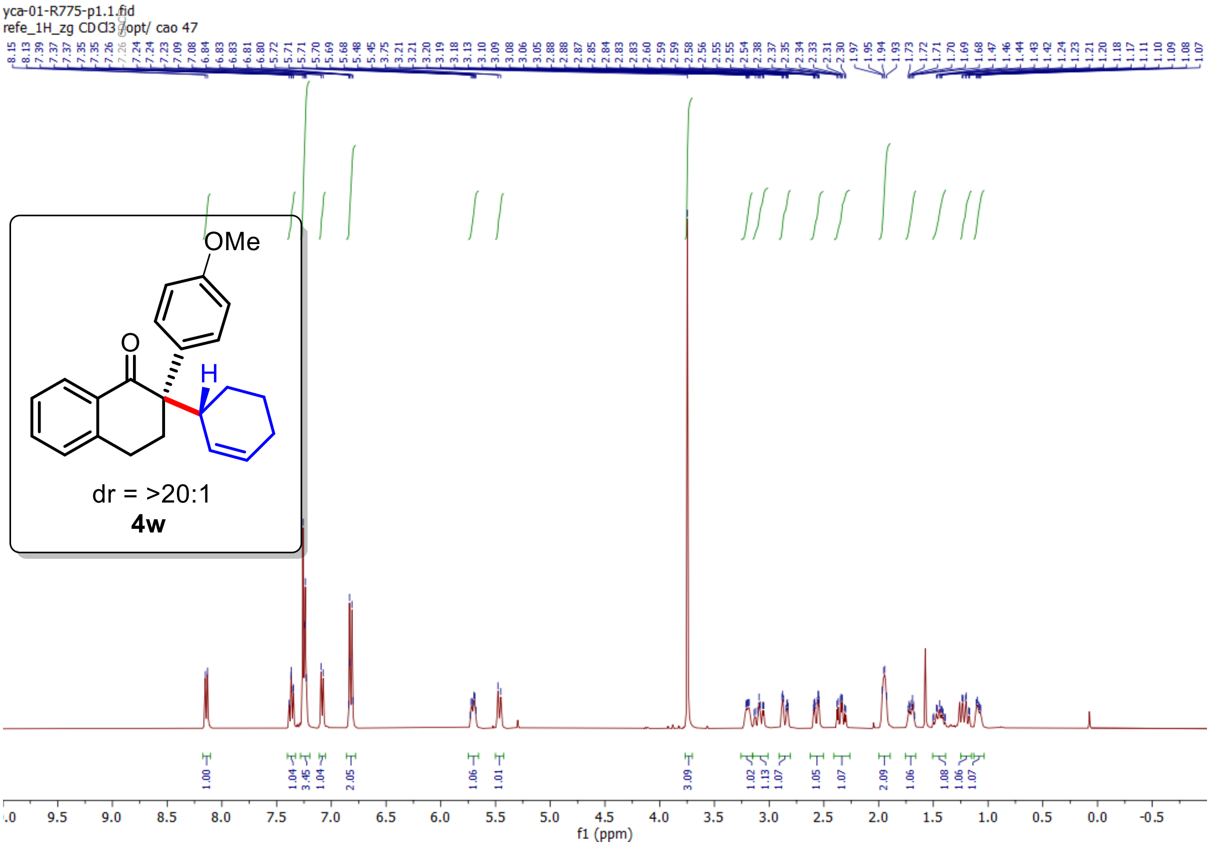
yca-01-R775-p1.1.fid
refe_1H_zg CDCl3 /opt/ cao 47
OMe
O
H
dr = >20:1
4w
f1 (ppm)

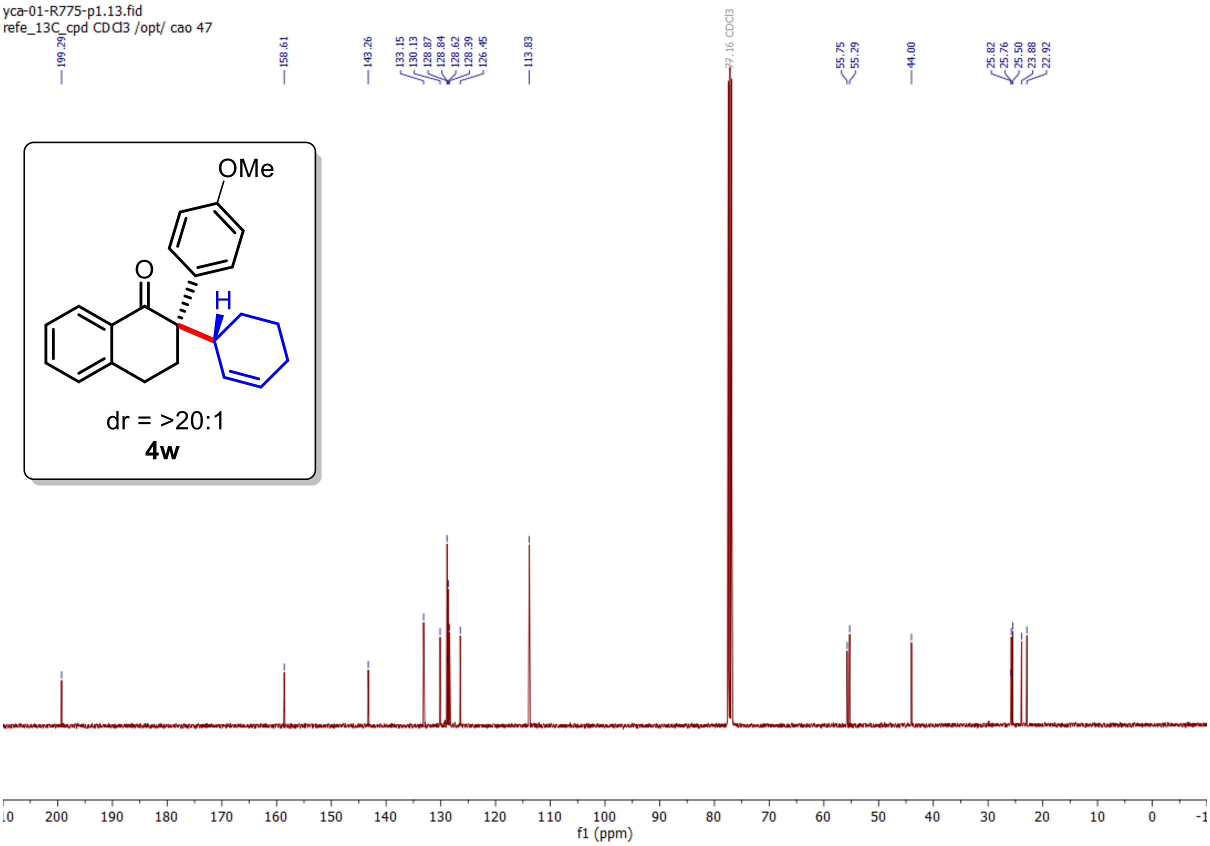
yca-01-R775-p1.13.fid
refe_13C_cpd CDCl3 /opt/ cao 47
199.29
158.61
143.26
133.15
130.13
128.87
128.84
128.62
128.39
126.45
113.83
77.16 CDCl3
55.75
55.29
44.00
25.82
25.76
25.50
23.88
22.92
OMe
O
H
dr = >20:1
4w
f1 (ppm)

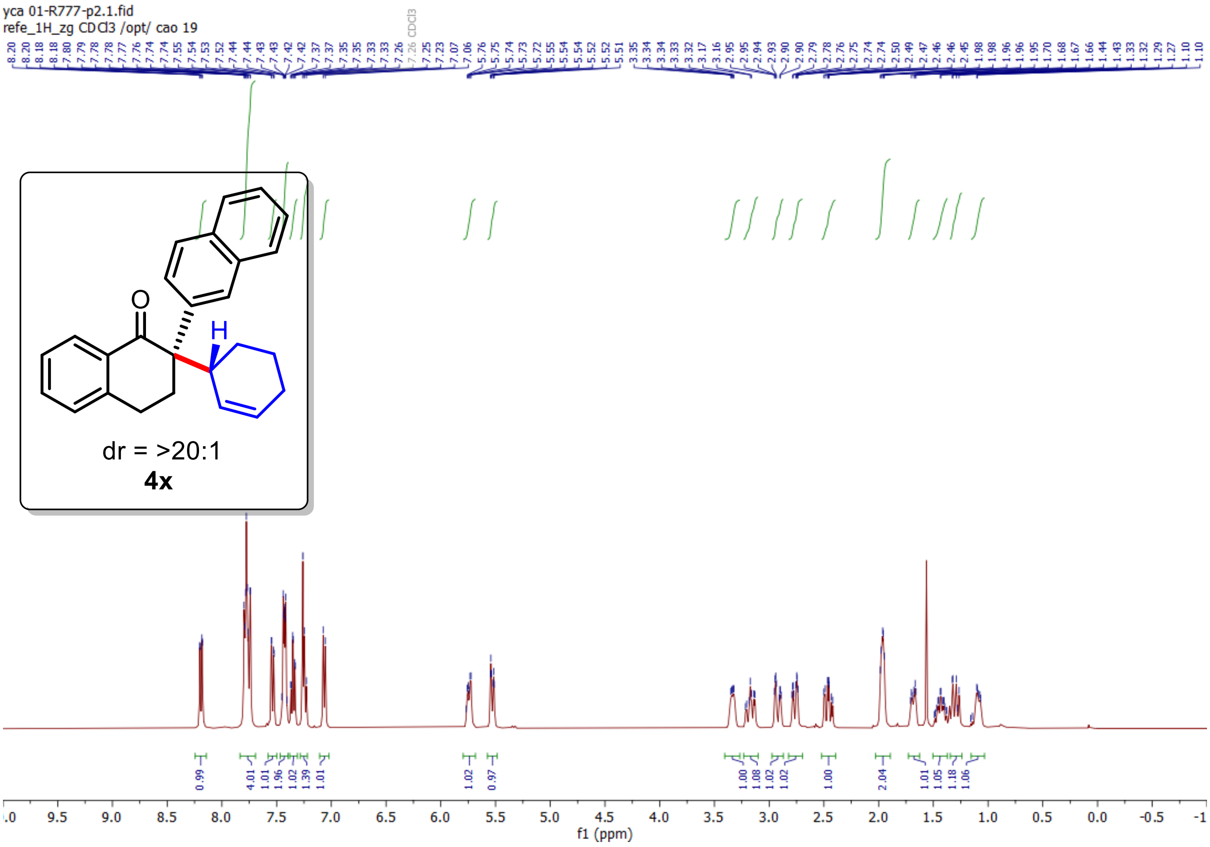
yca 01-R777-p2.1.fid
refe_1H_zg CDCl3 /opt/ cao 19
O
H
dr = >20:1
4x
0.99
4.01
1.01
1.96
1.02
1.39
1.01
1.02
0.97
1.00
1.08
1.02
1.02
1.00
2.04
1.01
1.05
1.18
1.06
f1 (ppm)

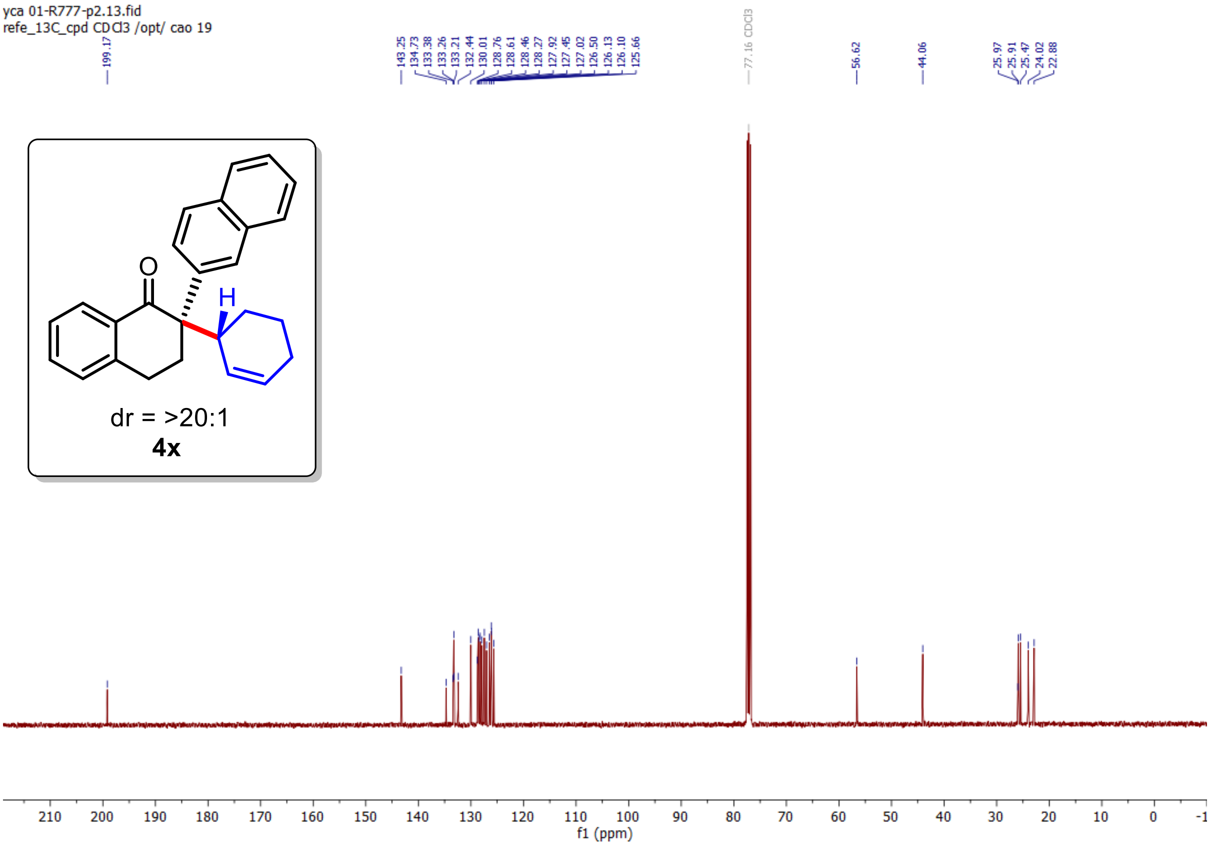
yca 01-R777-p2.13.fid
refe_13C_cpd CDCl3 /opt/ cao 19
199.17
143.25 134.73 133.38 133.26 133.21 132.44 130.01 128.76 128.61 128.46 128.27 127.92 127.45 127.02 126.50 126.13 126.10 125.66
77.16 CDCl3
56.62
44.06
25.97 25.91 25.47 24.02 22.88
O
H
dr = >20:1
4x
f1 (ppm)

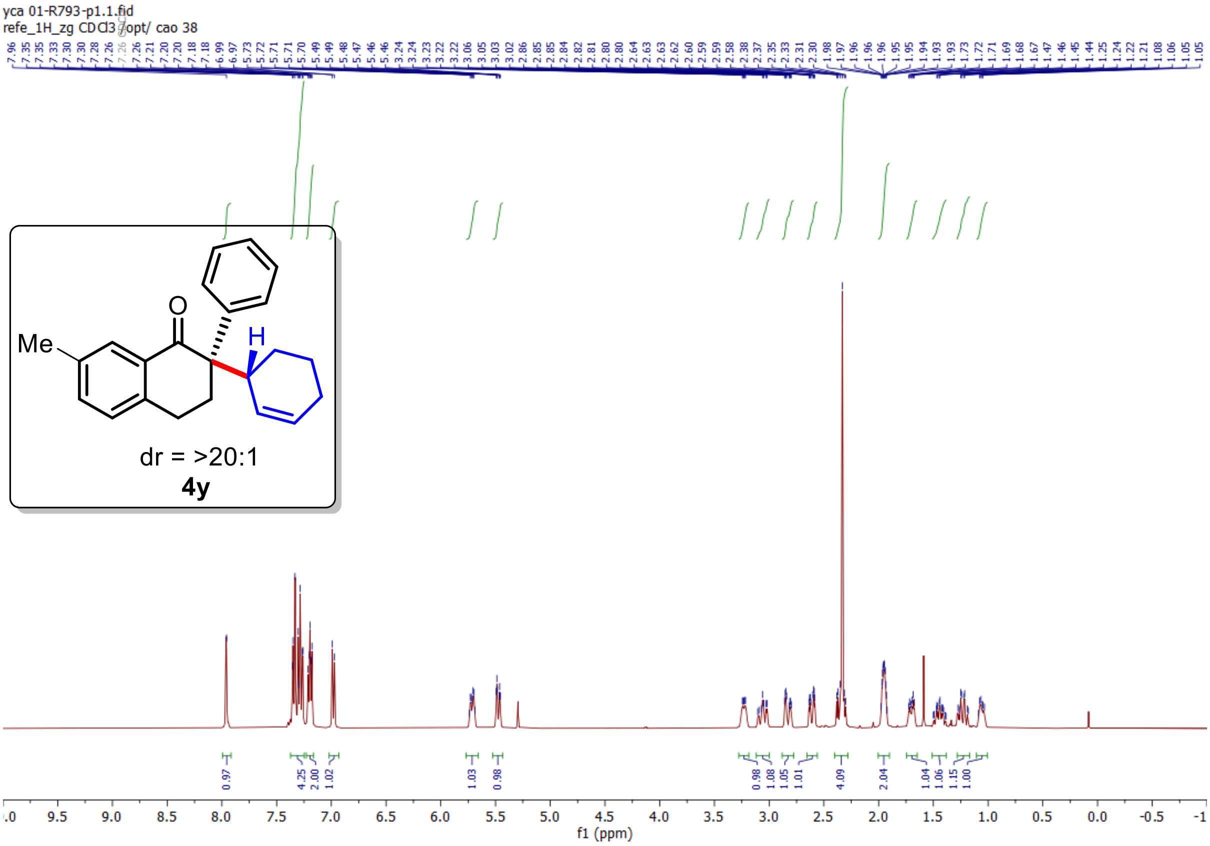
yca 01-R793-p1.1.fid
refe_1H_zg CDCl3 /opt/ cao 38
Me
O
H
dr = >20:1
4y
0.97
4.25
2.00
1.02
1.03
0.98
0.98
1.08
1.05
1.01
4.09
2.04
1.04
1.06
1.15
1.00
f1 (ppm)

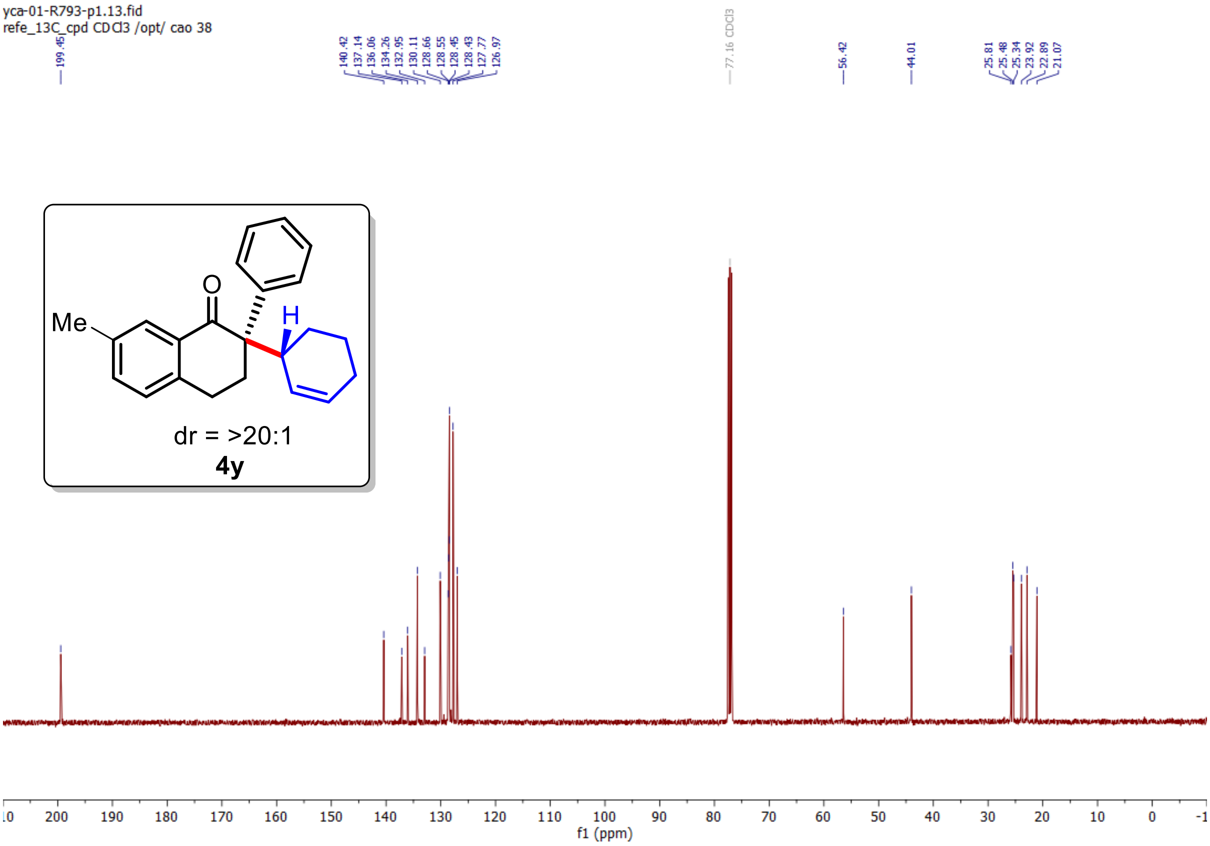
yca-01-R793-p1.13.fid
refe_13C_cpd CDCl3 /opt/ cao 38
199.45
140.42
137.14
136.06
134.26
132.95
130.11
128.66
128.55
128.45
128.43
127.77
126.97
77.16 CDCl3
56.42
44.01
25.81
25.48
25.34
23.92
22.89
21.07
Me
O
H
dr = >20:1
4y
f1 (ppm)

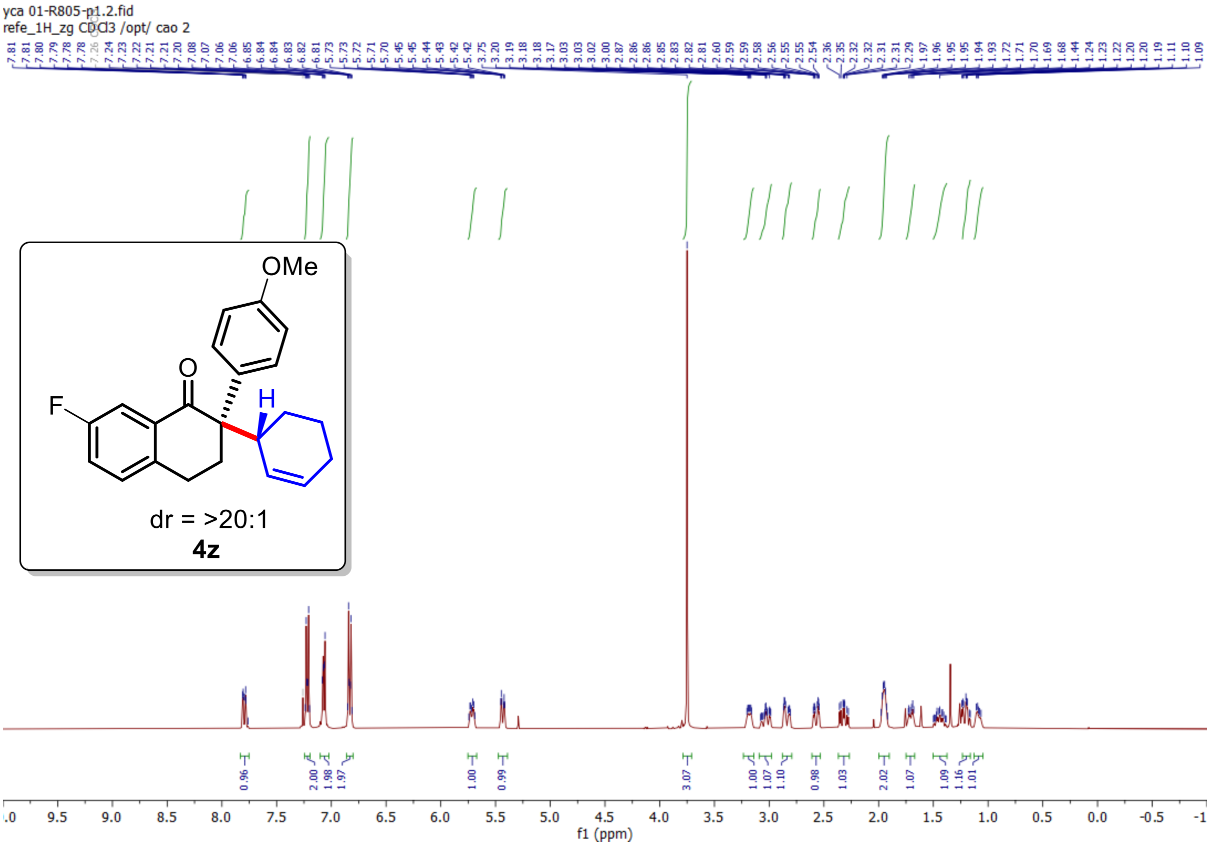
yca 01-R805-p1.2.fid
refe_1H_zg CDCl3 /opt/ cao 2
OMe
F
O
H
dr = >20:1
4z
0.96
2.00
1.98
1.97
1.00
0.99
3.07
1.00
1.07
1.10
0.98
1.03
2.02
1.07
1.09
1.16
1.01
f1 (ppm)

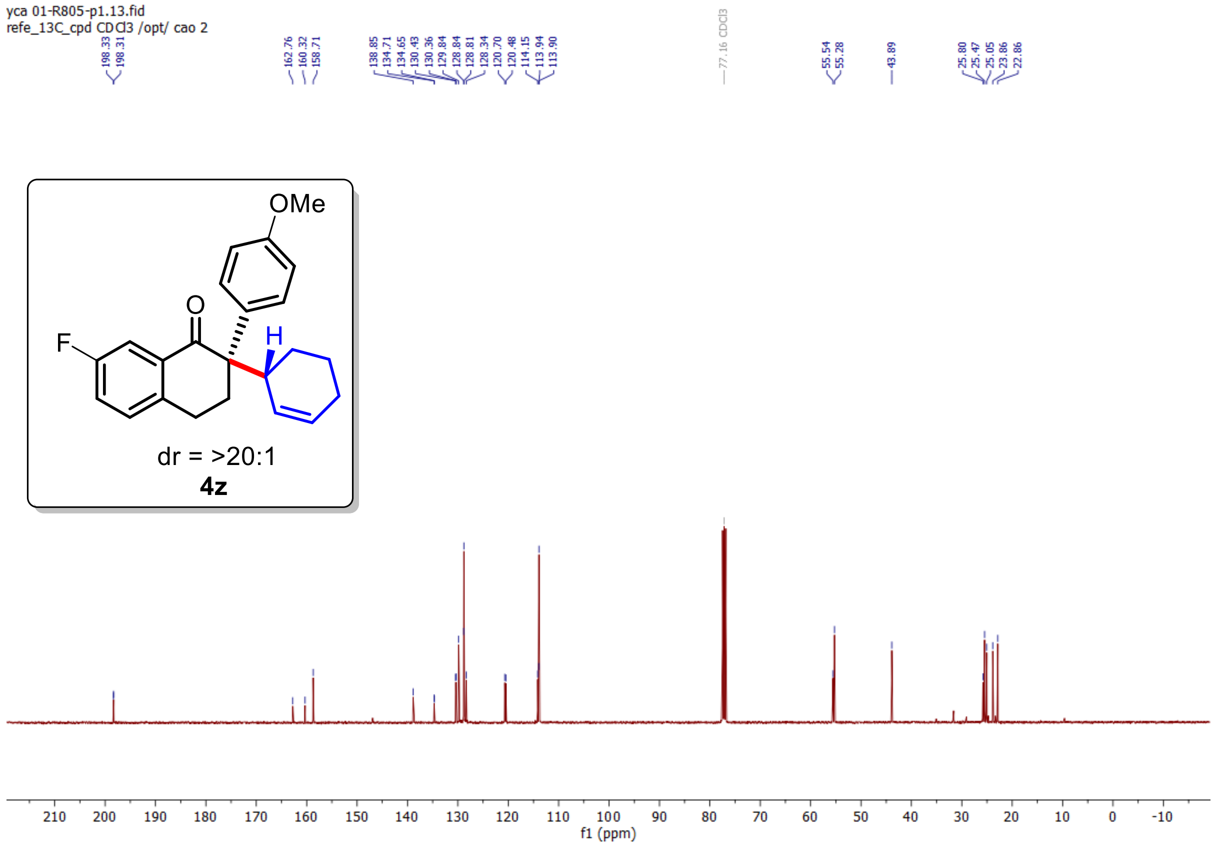
yca 01-R805-p1.13.fid
refe_13C_cpd CDCl3 /opt/ cao 2
198.33
198.31
162.76
160.32
158.71
138.85
134.71
134.65
130.43
130.36
129.84
128.84
128.81
128.34
120.70
120.48
114.15
113.94
113.90
77.16 CDCl3
55.54
55.28
43.89
25.80
25.47
25.05
23.86
22.86
OMe
O
F
H
dr = >20:1
4z
f1 (ppm)

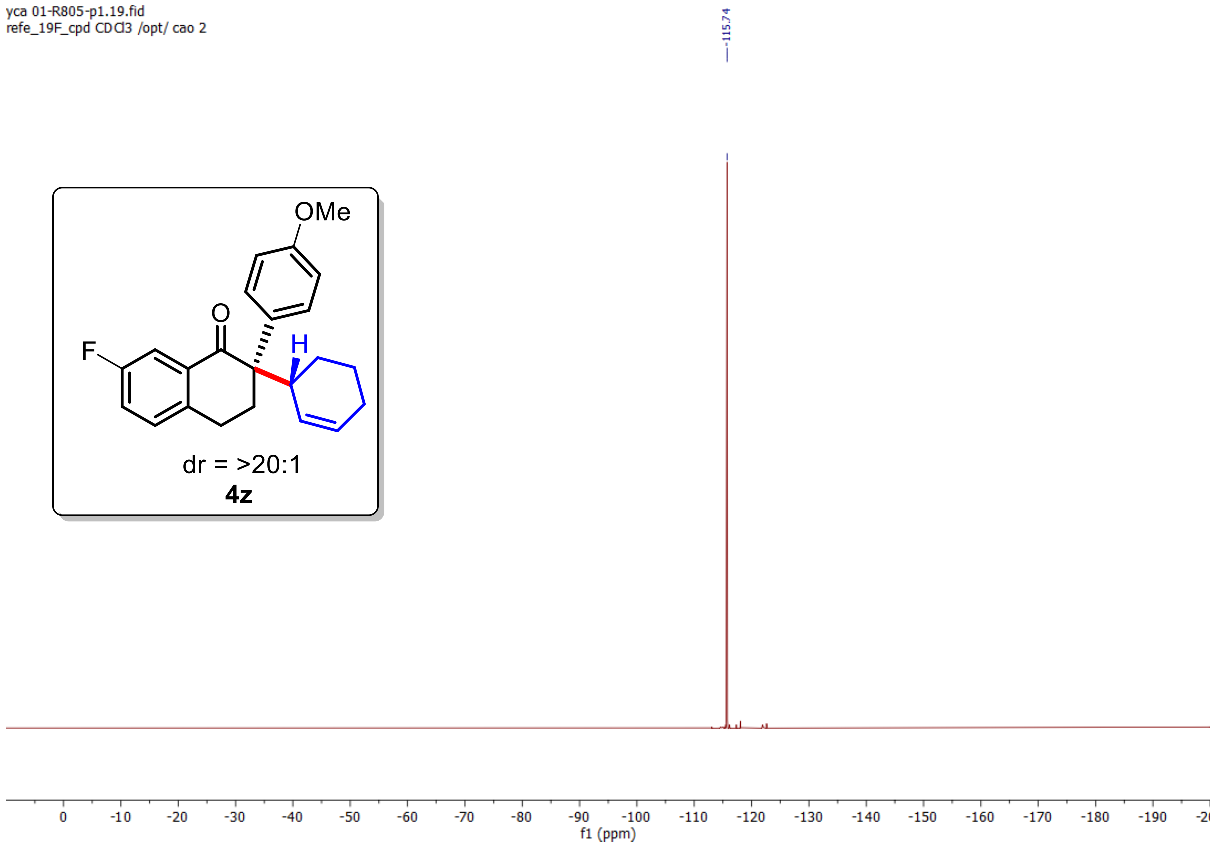
yca 01-R805-p1.19.fid
refe_19F_cpd CDCl3 /opt/ cao 2
-115.74
OMe
O
F
H
dr = >20:1
4z
f1 (ppm)

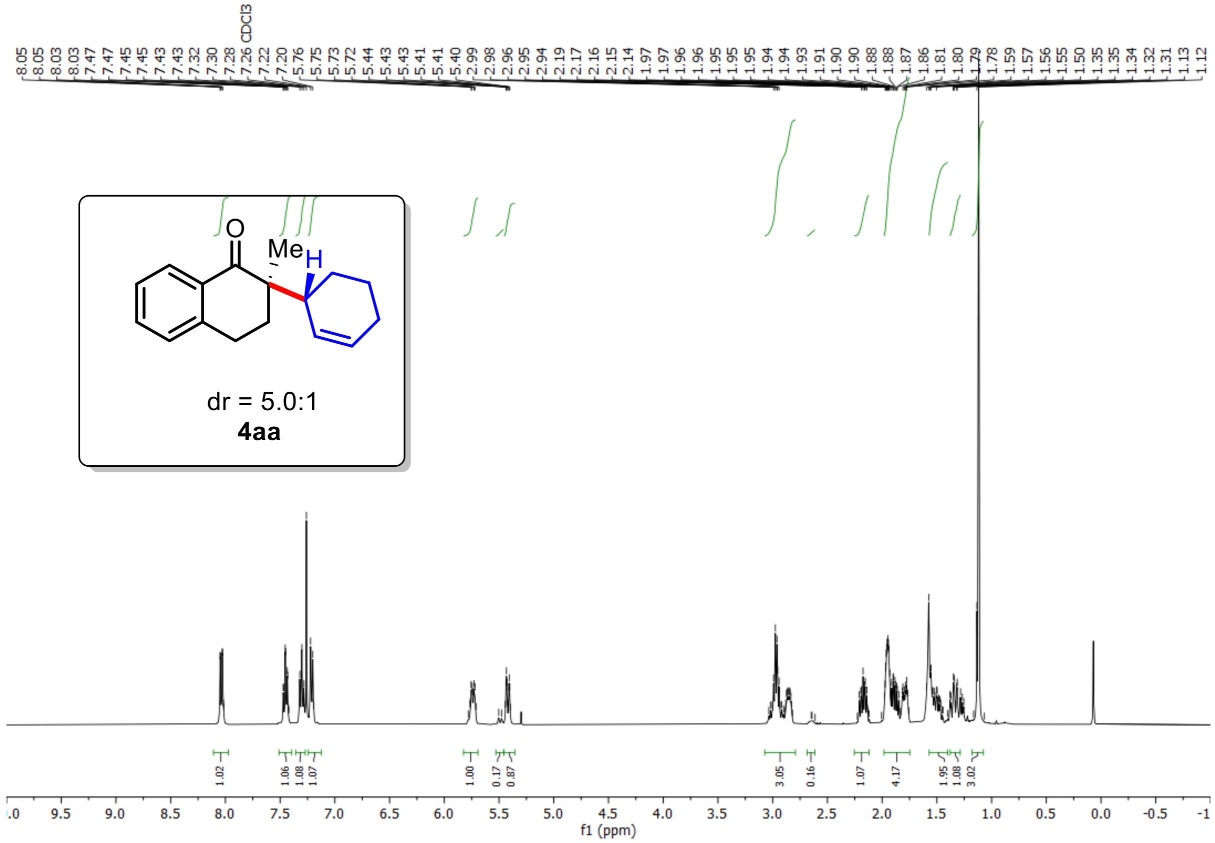
8.05 8.05 8.03 8.03 7.47 7.47 7.45 7.45 7.43 7.43 7.32 7.30 7.28 7.26 CDCl3 7.22 7.20 5.76 5.75 5.73 5.72 5.44 5.43 5.43 5.41 5.41 5.40 2.99 2.98 2.96 2.95 2.94 2.19 2.17 2.16 2.15 2.14 1.97 1.97 1.96 1.96 1.95 1.95 1.95 1.94 1.94 1.93 1.91 1.90 1.90 1.88 1.88 1.87 1.86 1.81 1.80 1.79 1.78 1.59 1.57 1.56 1.55 1.50 1.35 1.35 1.34 1.32 1.31 1.13 1.12
O
Me
H
dr = 5.0:1
4aa
1.02 1.06 1.08 1.07 1.00 0.17 0.87 3.05 0.16 1.07 4.17 1.95 1.08 3.02
f1 (ppm)

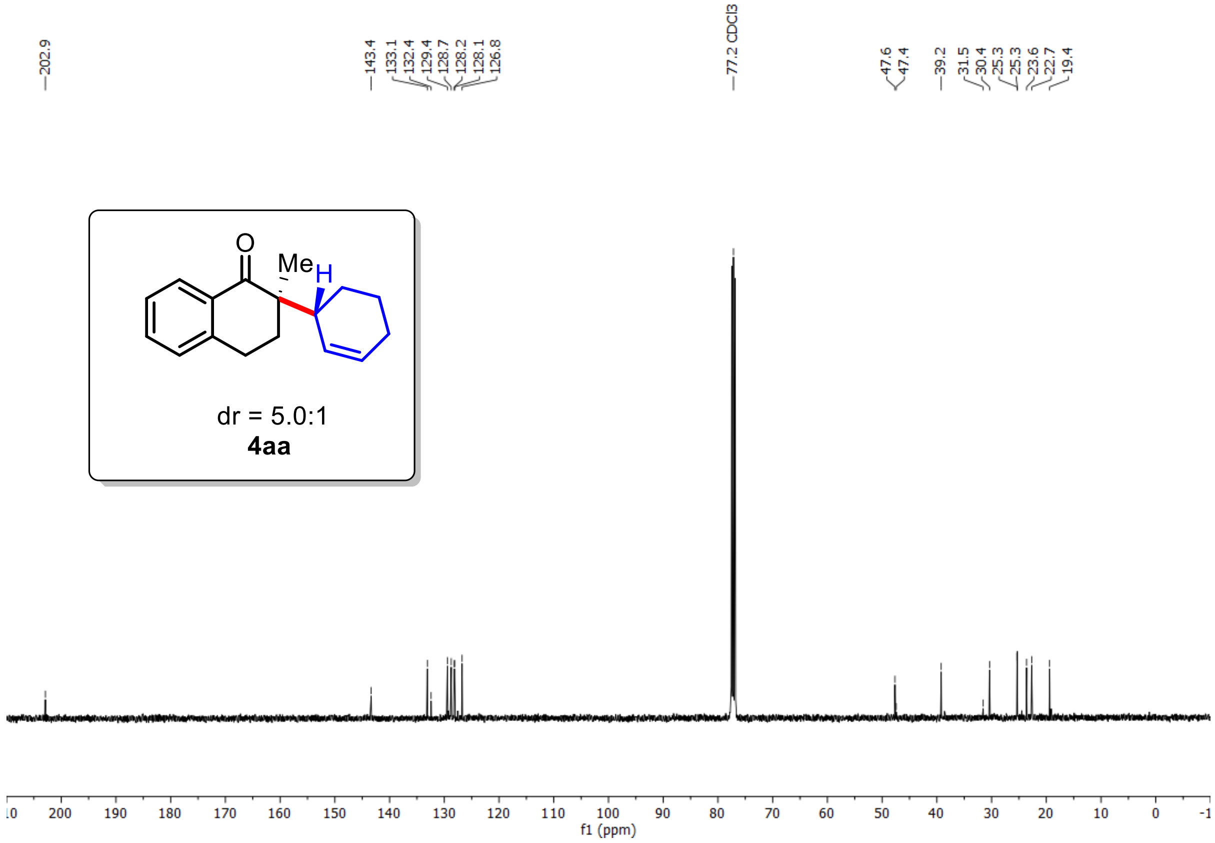
202.9 143.4 133.1 132.4 129.4 128.7 128.2 128.1 126.8 77.2 CDCl3 47.6 47.4 39.2 31.5 30.4 25.3 25.3 23.6 22.7 19.4
O
Me
H
dr = 5.0:1
4aa
f1 (ppm)

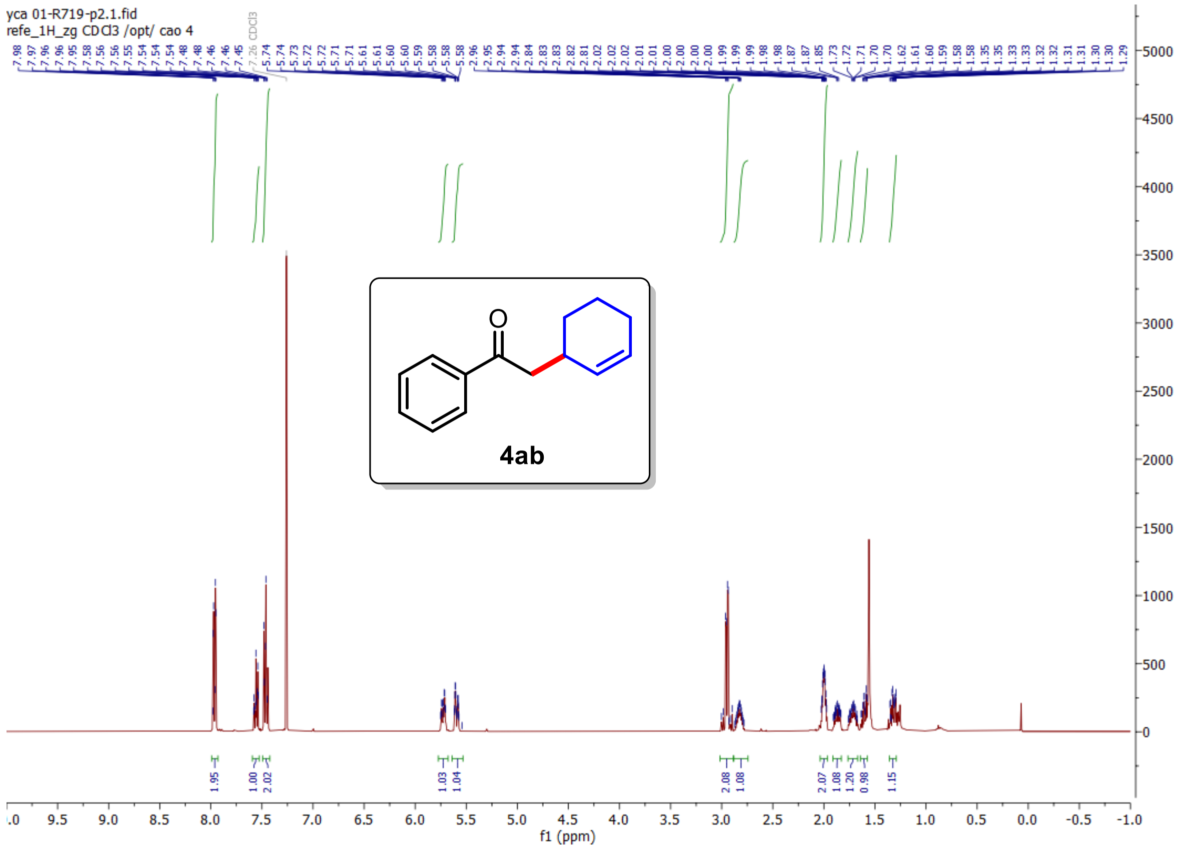
yca 01-R719-p2.1.fid
refe_1H_zg CDCl3 /opt/ cao 4
4ab
1.95
1.00
2.02
1.03
1.04
2.08
1.08
2.07
1.08
1.20
0.98
1.15
f1 (ppm)

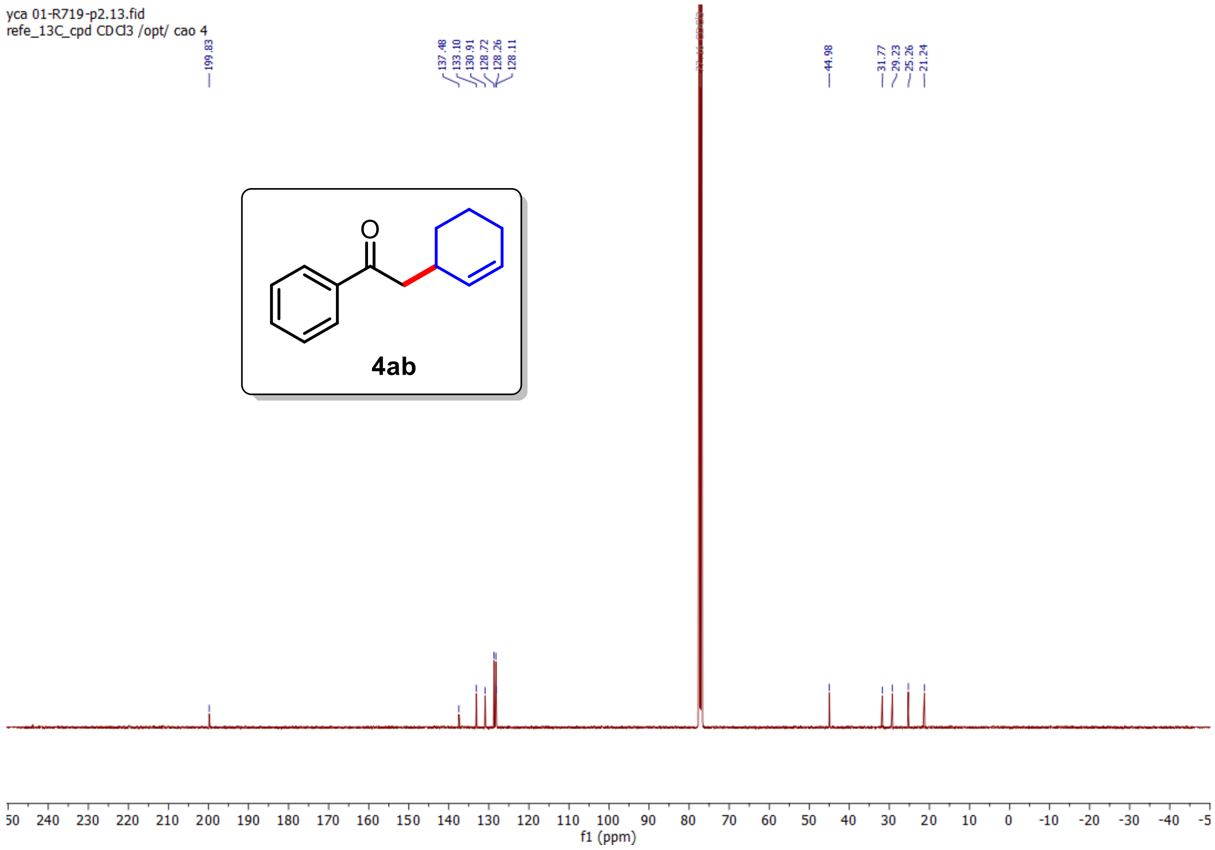
yca 01-R719-p2.13.fid
refe_13C_cpd CDCl3 /opt/ cao 4
199.83
137.48
133.10
130.91
128.72
128.26
128.11
44.98
31.77
29.23
25.26
21.24
4ab
f1 (ppm)

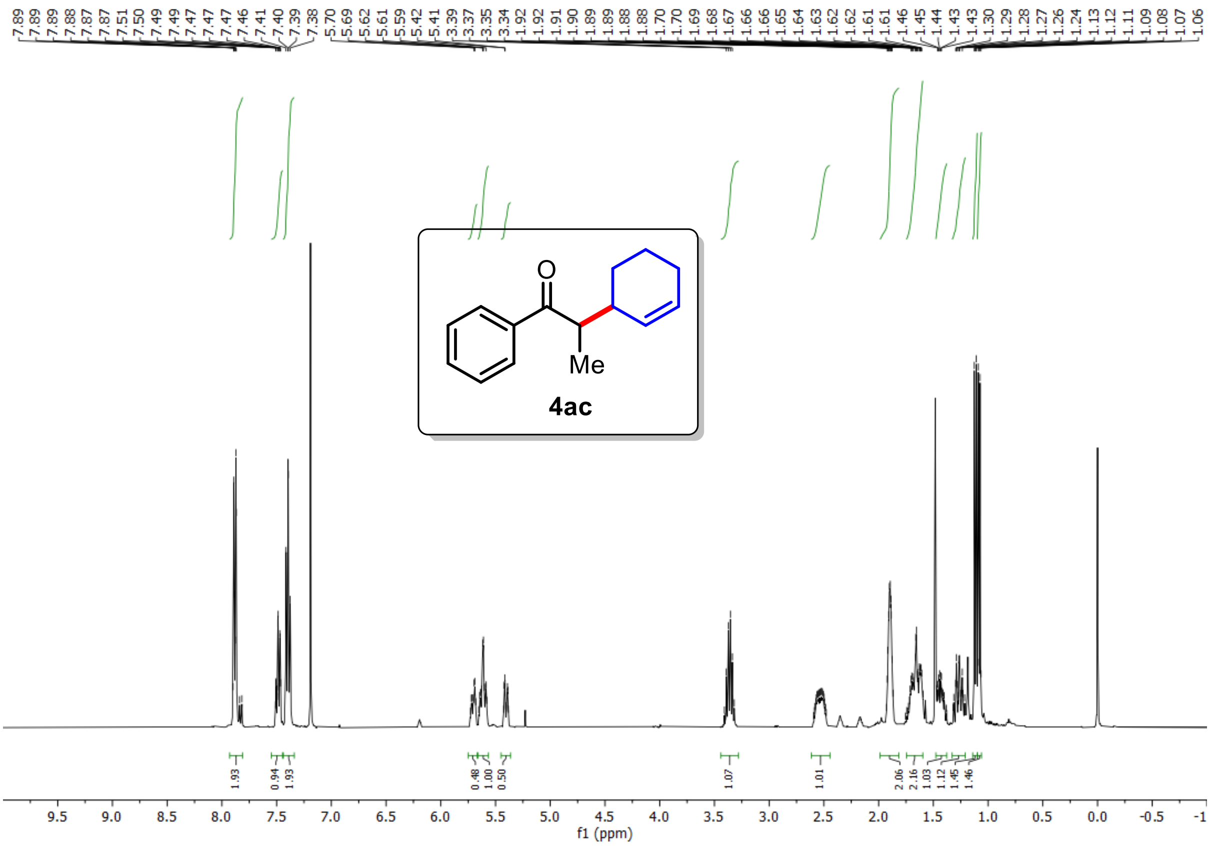
7.89
7.89
7.89
7.88
7.87
7.87
7.51
7.50
7.49
7.49
7.47
7.47
7.47
7.46
7.41
7.40
7.39
7.38
5.70
5.69
5.62
5.61
5.59
5.42
5.41
3.39
3.37
3.35
3.34
1.92
1.92
1.91
1.90
1.89
1.89
1.88
1.88
1.70
1.70
1.69
1.68
1.67
1.66
1.66
1.65
1.64
1.63
1.62
1.62
1.61
1.61
1.46
1.45
1.44
1.43
1.43
1.30
1.29
1.28
1.27
1.26
1.24
1.13
1.12
1.11
1.09
1.08
1.07
1.06
O
Me
4ac
1.93
0.94
1.93
0.48
1.00
0.50
1.07
1.01
2.06
2.16
1.03
1.12
1.45
1.46
f1 (ppm)

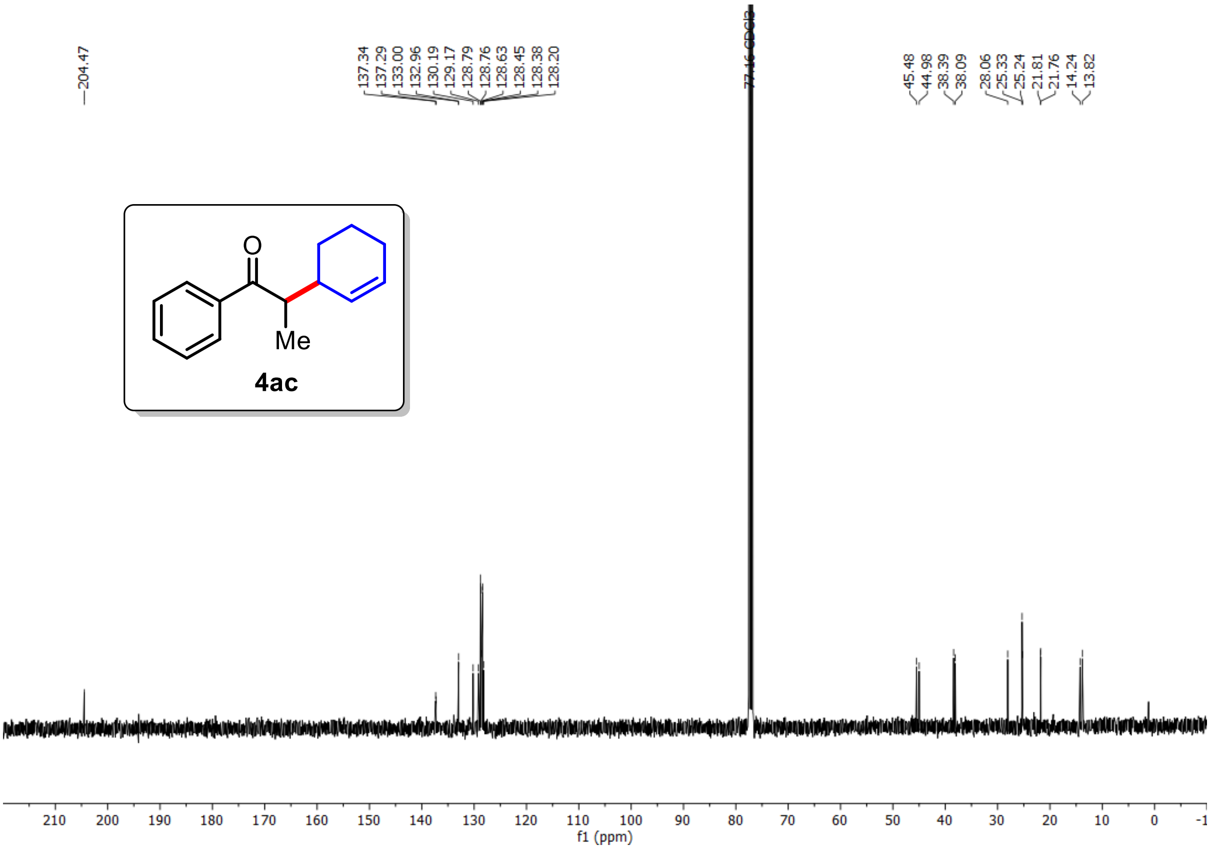
204.47
137.34
137.29
133.00
132.96
130.19
129.17
128.79
128.76
128.63
128.45
128.38
128.20
77.16 CDCl3
45.48
44.98
38.39
38.09
28.06
25.33
25.24
21.81
21.76
14.24
13.82
O
Me
4ac
f1 (ppm)

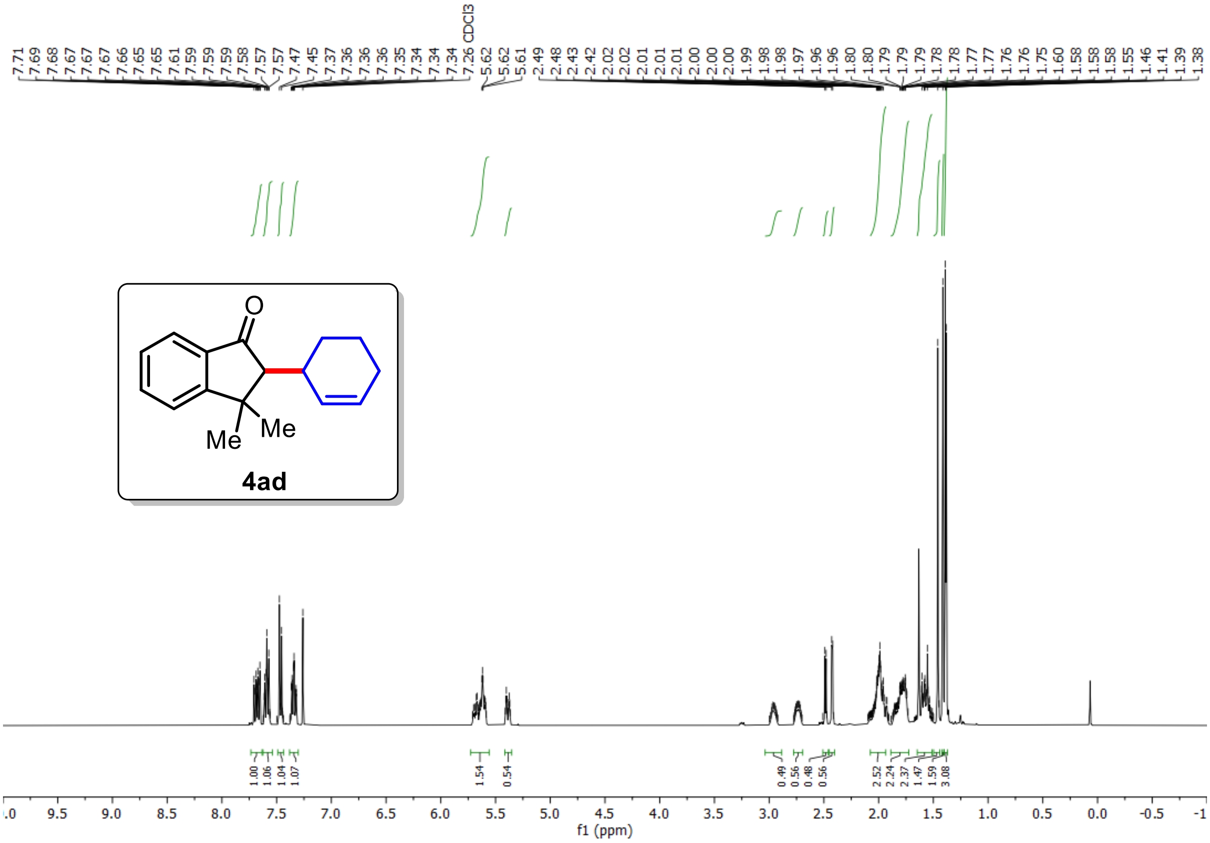
CDCl3
O
Me
Me
4ad
f1 (ppm)

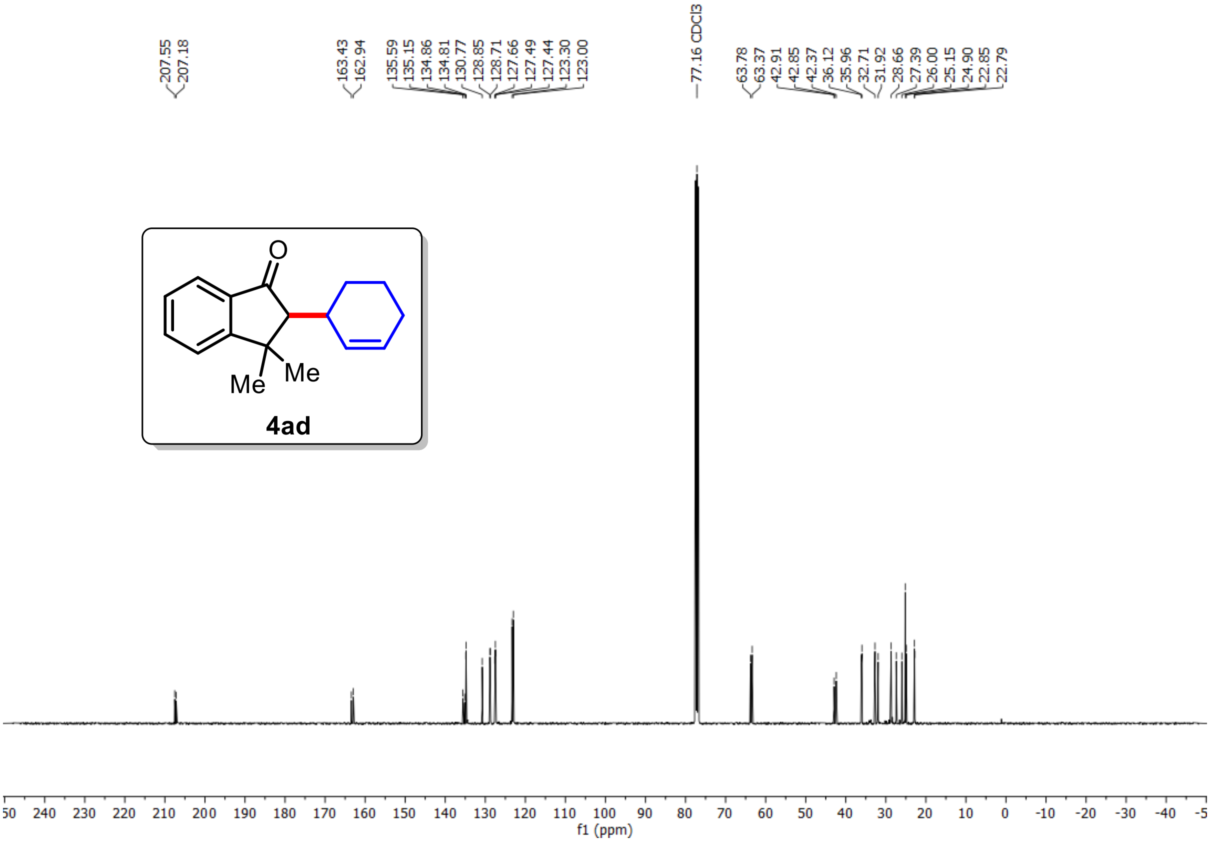
CDCl3
O
Me
Me
4ad
f1 (ppm)

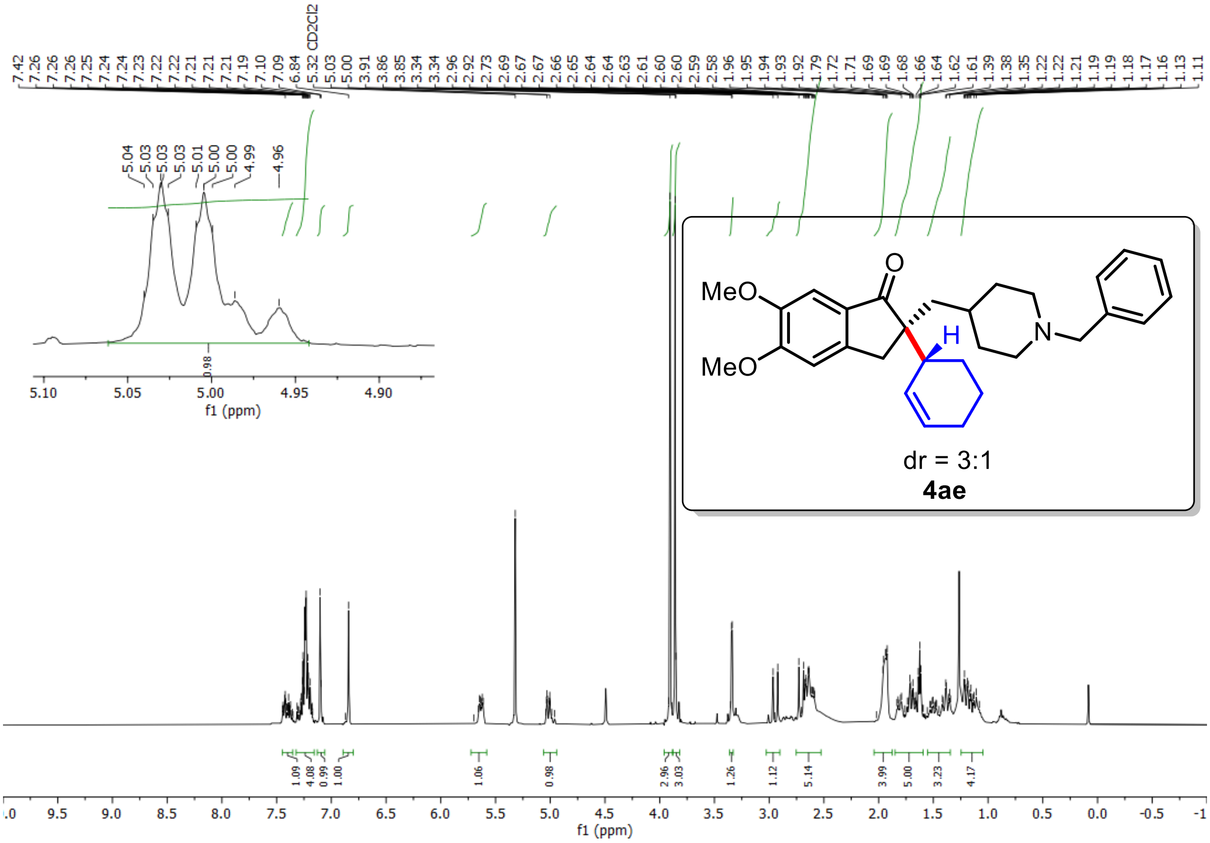
MeO
MeO
O
H
N
dr = 3:1
4ae
f1 (ppm)

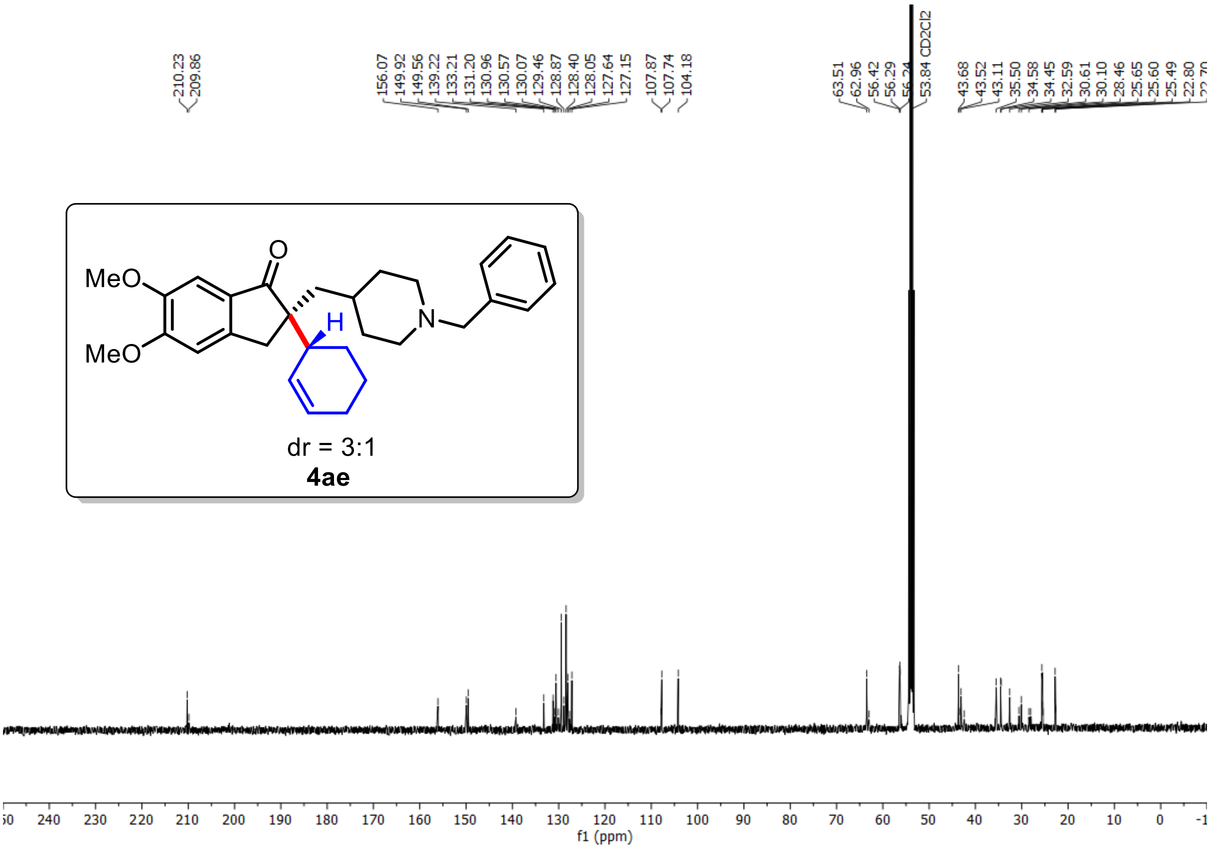
MeO
MeO
O
H
N
dr = 3:1
4ae
f1 (ppm)

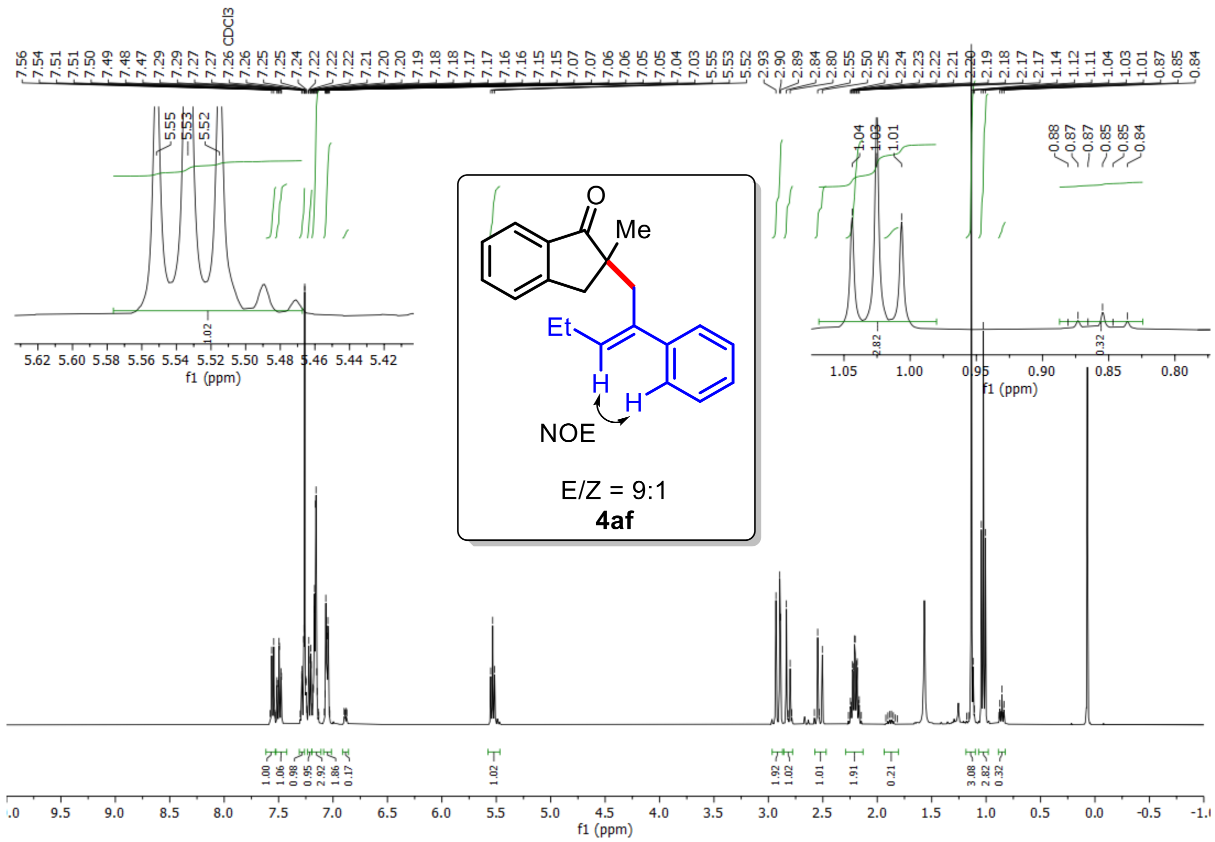

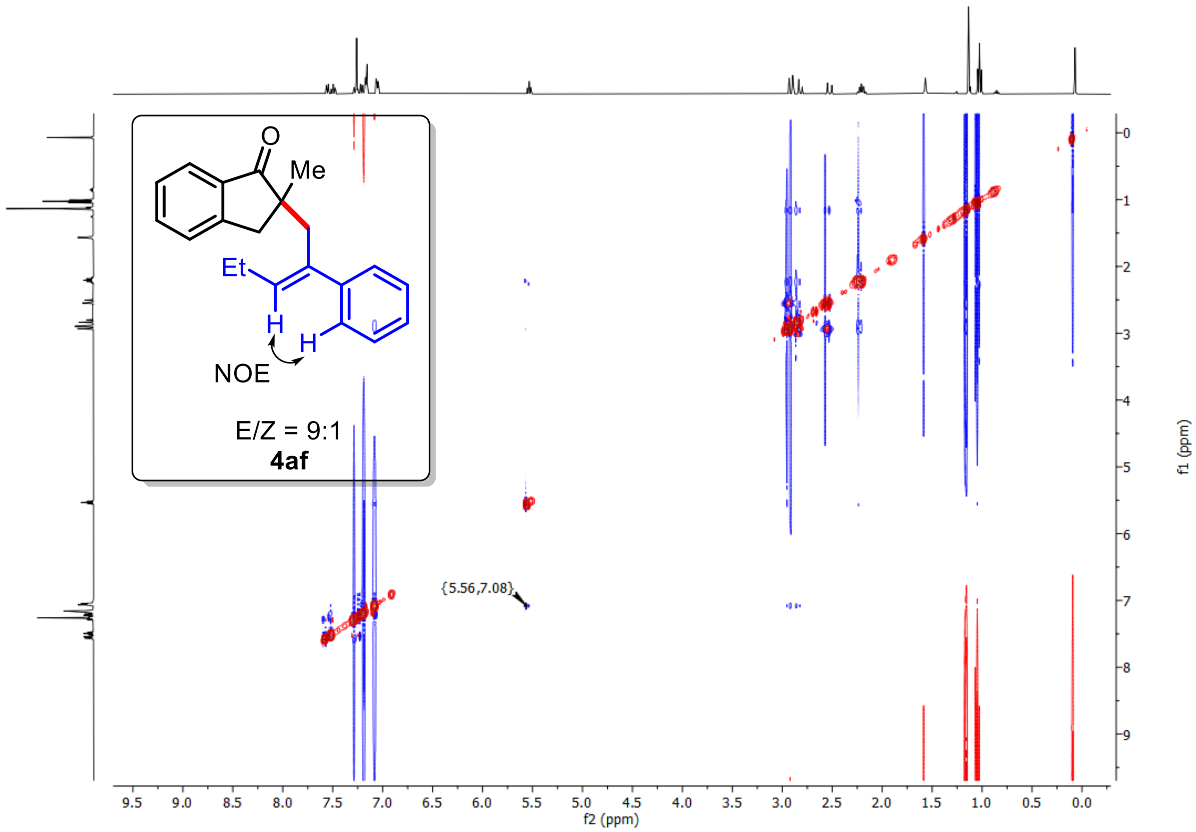

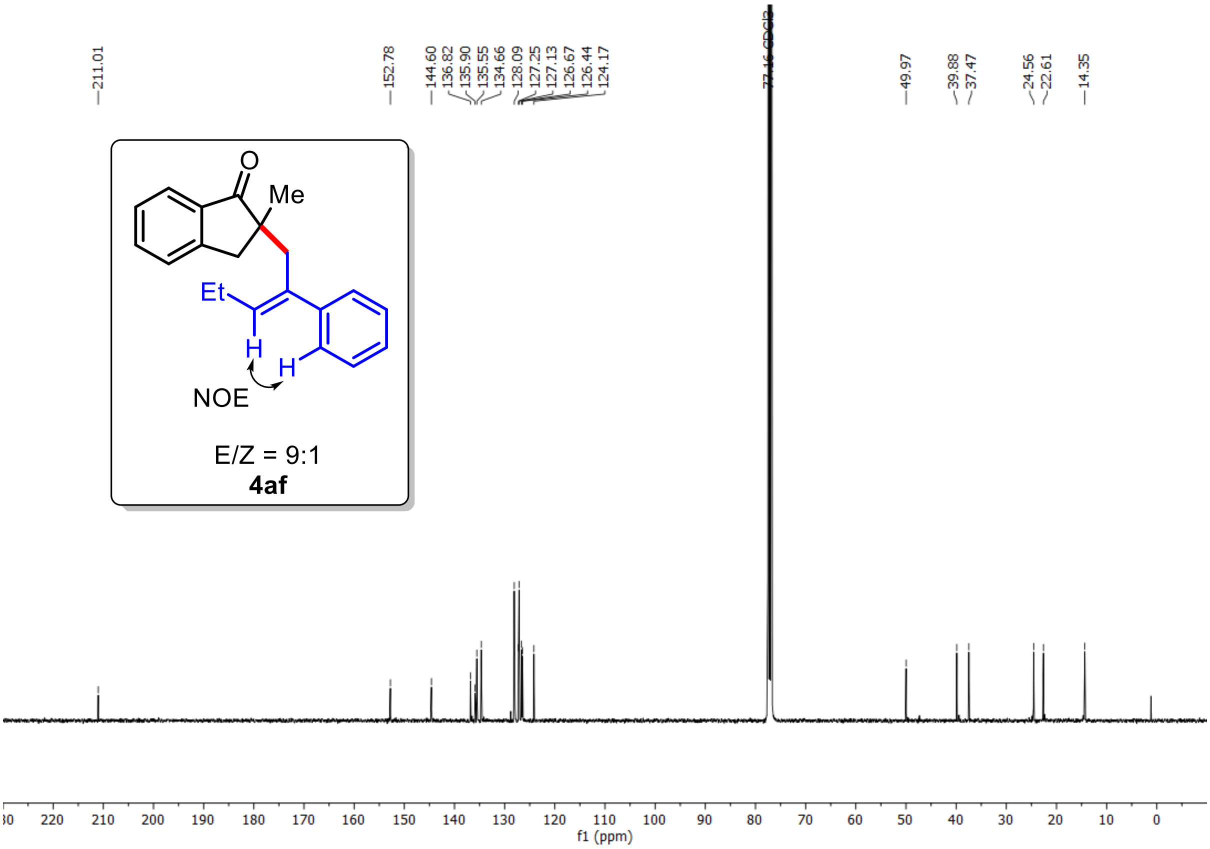
211.01
152.78
144.60
136.82
135.90
135.55
134.66
128.09
127.25
127.13
126.67
126.44
124.17
77.16 CDCl3
49.97
39.88
37.47
24.56
22.61
14.35
O
Me
Et
H
H
NOE
E/Z = 9:1
4af
f1 (ppm)

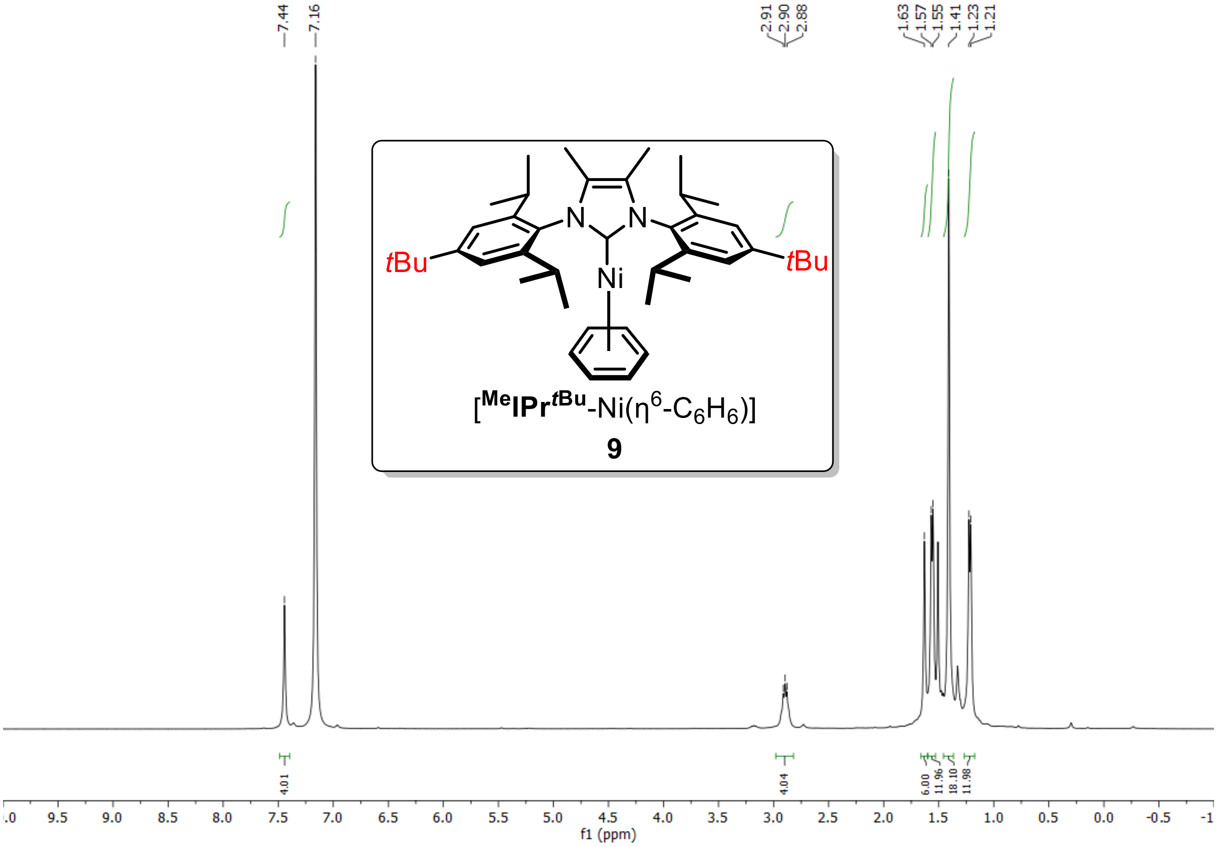
7.44
7.16
2.91
2.90
2.88
1.63
1.57
1.55
1.41
1.23
1.21
tBu
N
N
Ni
tBu
[MeIPrtBu-Ni(η6-C6H6)]
9
4.01
4.04
6.00
11.96
18.10
11.98
f1 (ppm)

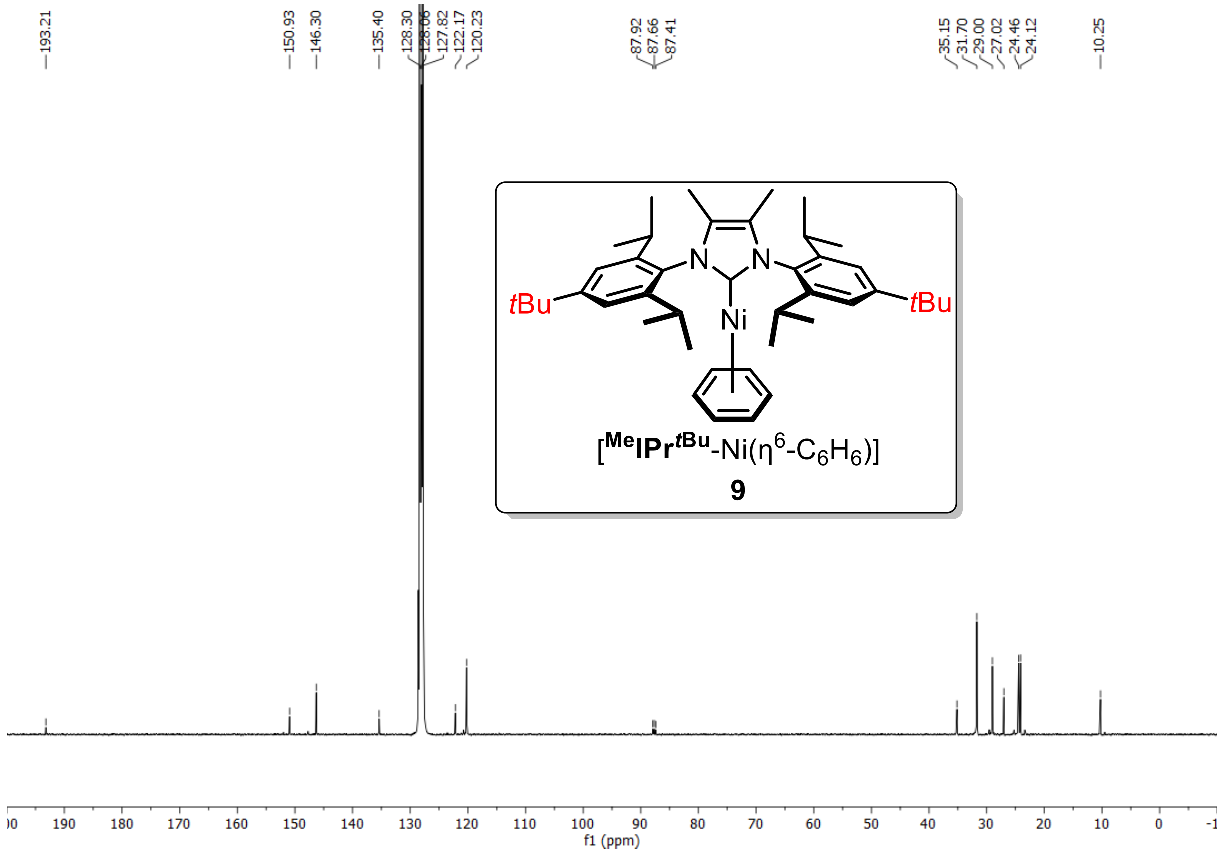
193.21
150.93
146.30
135.40
128.30
127.82
122.17
120.23
87.92
87.66
87.41
35.15
31.70
29.00
27.02
24.46
24.12
10.25
tBu
N
N
Ni
tBu
[MeIPrtBu-Ni(η6-C6H6)]
9
f1 (ppm)

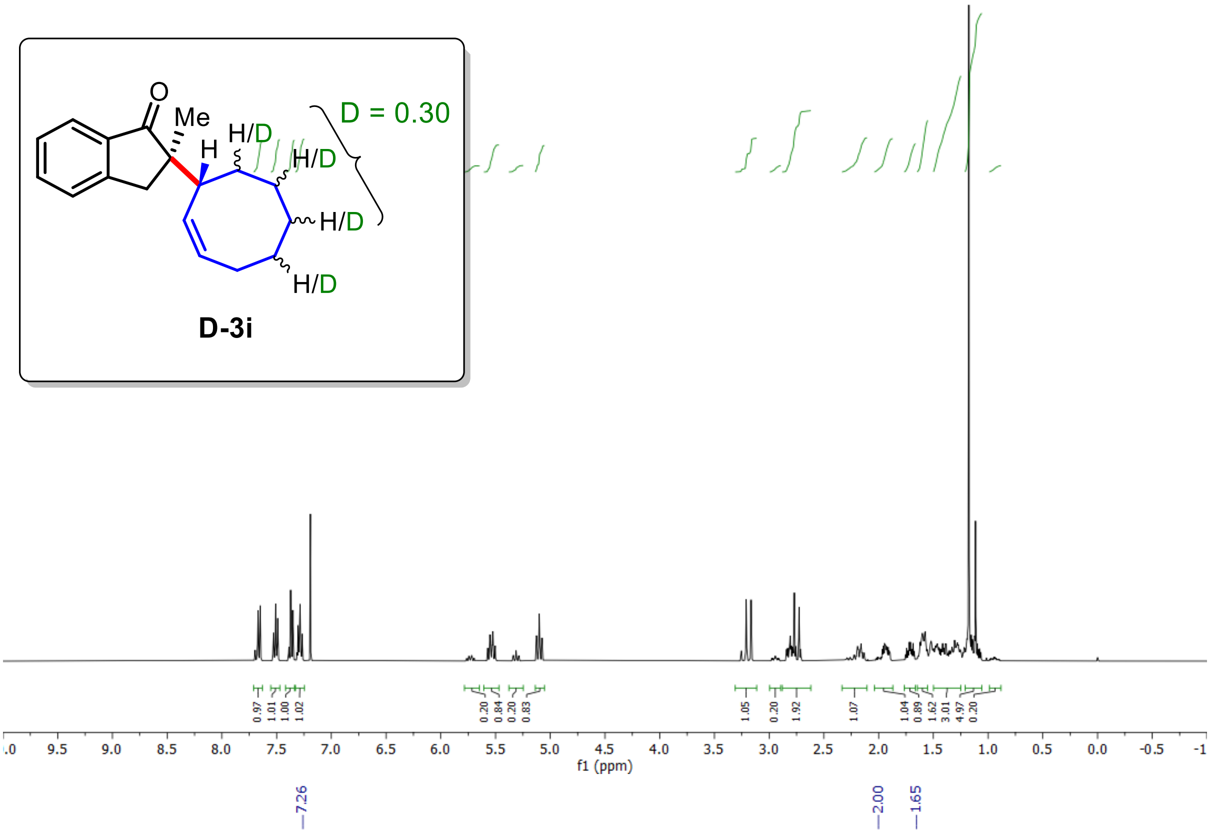
D = 0.30
Me
H
H/D
D-3i
0.97
1.01
1.00
1.02
0.20
0.84
0.20
0.83
1.05
0.20
1.92
1.07
1.04
0.89
1.62
3.01
4.97
0.20
f1 (ppm)

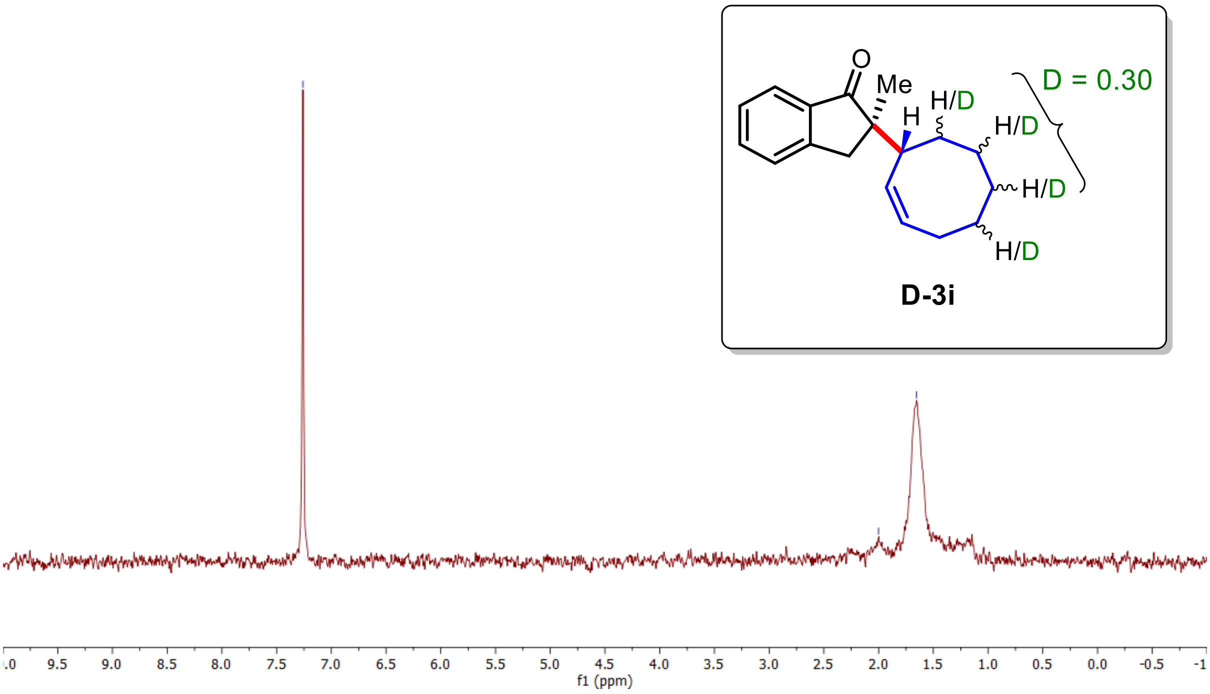
7.26
2.00
1.65
D = 0.30
Me
H
H/D
D-3i
f1 (ppm)

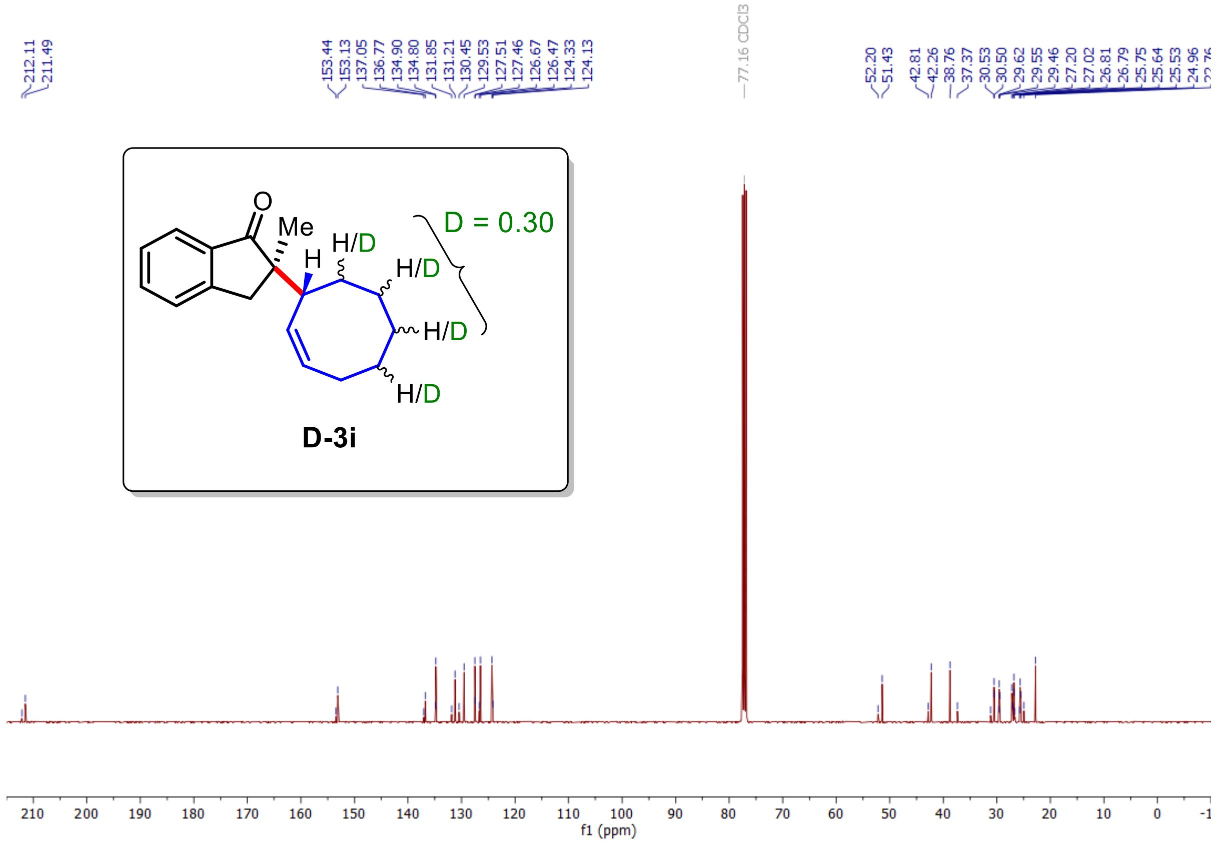
212.11
211.49
153.44
153.13
137.05
136.77
134.90
134.80
131.85
131.21
130.45
129.53
127.51
127.46
126.67
126.47
124.33
124.13
77.16 CDCl3
52.20
51.43
42.81
42.26
38.76
37.37
30.53
30.50
29.62
29.55
29.46
27.20
27.02
26.81
26.79
25.75
25.64
25.53
24.96
O
Me
H
H/D
D = 0.30
D-3i
f1 (ppm)

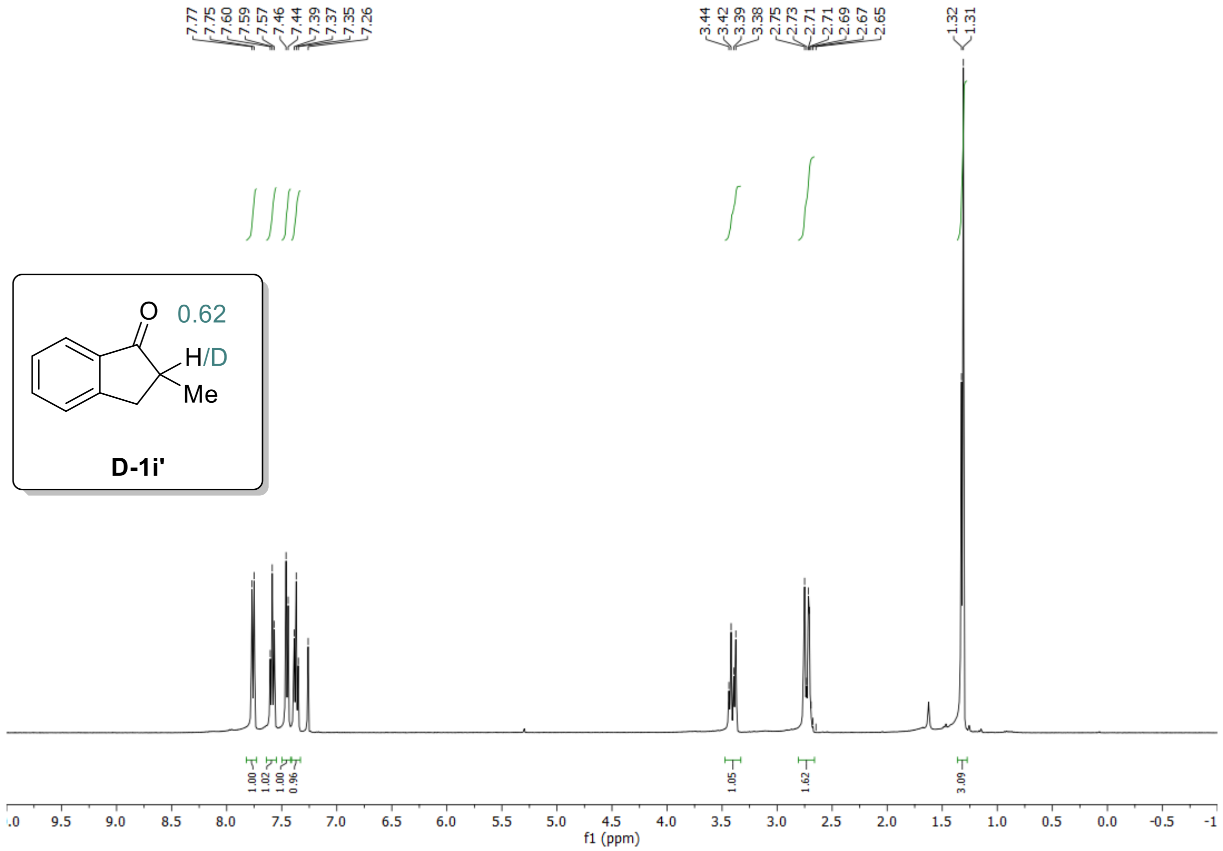
7.77
7.75
7.60
7.59
7.57
7.46
7.44
7.39
7.37
7.35
7.26
3.44
3.42
3.39
3.38
2.75
2.73
2.71
2.71
2.69
2.67
2.65
1.32
1.31
O
0.62
H/D
Me
D-1i'
1.00
1.02
1.00
0.96
1.05
1.62
3.09
f1 (ppm)

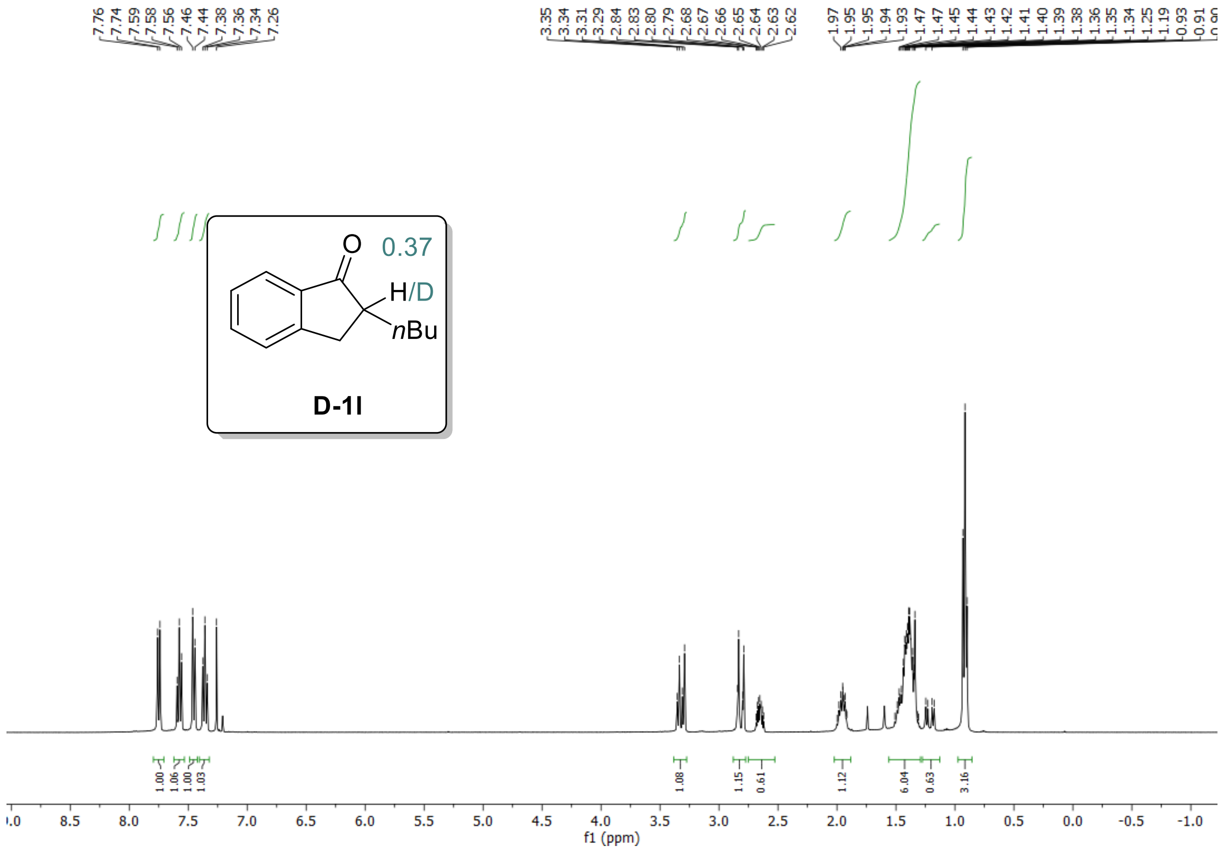
7.76 7.74 7.59 7.58 7.56 7.46 7.44 7.38 7.36 7.34 7.26
3.35 3.34 3.31 3.29 2.84 2.83 2.80 2.79 2.68 2.67 2.66 2.65 2.64 2.63 2.62
1.97 1.95 1.95 1.94 1.93 1.47 1.47 1.45 1.44 1.43 1.42 1.41 1.40 1.39 1.38 1.36 1.35 1.34 1.25 1.19 0.93 0.91
O
0.37
H/D
nBu
D-1l
1.00 1.06 1.00 1.03 1.08 1.15 0.61 1.12 6.04 0.63 3.16
f1 (ppm)

8. Reference:

(1) Freitag, B., Stegner, P., Thum, K., Fischer, C. A. & Harder, S. *Eur. J. Inorg. Chem.* 1938–1944 **(**2018).

(2) Bakhoda, A., Jiang, Q., Badiei, Y. M., Bertke, J. A., Cundari, T. R. & Warren, T. H. *Angew. Chem. Int. Ed.*, **58**, 3421–3425 (2019**)**.

(3) Ausdall, B. R. V., Glass, J. L., Wiggins, K. M., Aarif, A. M. & Louie J. *J. Org. Chem.* **74**, 7935–7942 (2009).

(4) Dai, S., Sui, X. & Chen, C. *Angew. Chem. Int. Ed.* **54**, 9948–9953 (2015).

(5) Beillard, A., Métro, T.-X., Bantreil, X., Martinez, J. & Lamaty, F. *Chem. Sci.* **8**, 1086-1089 (2017**)**.

(6) Tate, B. K., Wyss, C. M., Bacsa. J., Kluge, K., Gelbaum, L. & Sadighi, J. P. *Chem. Sci.* **4**, 3068-3074 (2013).

(7) Meiries, S.; Nolan, S. P. *Synlett*. **25**, 393–398 (2014**)**.

(8) Nelson, D. J., Collado, A., Manzini, S., Meiries, S., Slawin, A. M. Z., Cordes, D. B. & Nolan, S. P. *Organometallics*. **33**, 2048−2058 (2014**)**.

(9) Diesel, J., Finogenova, A. M. & Cramer, N. *J. Am. Chem. Soc.* **140**, 4489− 4493 (2018).

(10) Nelson, D. J., Collado, A., Manzini, S., Meiries, S., Slawin, A. M. Z., Cordes, D. B. & Nolan, S. P. *Organometallics.*, **33**, 2048−2058 (2014).

(11) Diesel, J., Finogenova, A. & Cramer, N. *J. Am. Chem. Soc.* **140**, 4489-4493 (2018).

(12) Diesel, J., Grosheva, D., Kodama, S. & Cramer, N. *Angew. Chem. Int. Ed.* **58**, 11044-11048 (2019**)**.

(13) Braconi, E. & Cramer, N., *Angew. Chem. Int. Ed.* **59**, 16425-16429 (2020).

(14) Moreno, I., Tellitu, I., Domínguez, E. & SanMartín, R. *Eur. J. Org. Chem.* 2126–2135 (2002).

(15) Li, C., Cao, Y.-X., Jin, R.- X., Bian, K.-J., Qin, Z.-Y., Lan, Q. & Wang, X.-S. *Chem. Sci.*, **10**, 9285−9291 (2019**)**.

(16) Li, C., Cao, Y.-X., Wang, R., Wang, Y.-N., Lan, Q. & Wang, X.-S.; *Nat Commun.*, **9**, 4951 (2018).

(17) Yoon, I. C., Kim, T. G. & Cho, C. S. *Organometallics*. **33**, 1890 (2014).

(18) Hussain, Z., Hopf, H., Pohl, L., Oeser, T., Fischer, A. K. & Jones, P. G. *Eur. J. Org. Chem.* **24**, 5555-5569 (2006).

(19) He, T., Wang, G., Bonetti, V., Klare, H. F. T. & Oestreich, M. Silylium-Ion-Promoted (5 + 1) Cycloaddition of Aryl-Substituted Vinylcyclopropanes and Hydrosilanes Involving Aryl Migration. *Angew. Chem., Int. Ed.* **59**, 12186−12191 (2020).

(20) Yang, C.-F., Wang, J.-Y. & Tian, S.-K. *Chem. Commun.* **47**, 8343 (2011).

(21) Hoshimoto, Y., Hayashi, Y., Suzuki, H., Ohashi, M. & Ogoshi, S. *Organometallics.* **33**, 1276–1282 (2014).

(22) Saper, N. I. & Hartwig, J. F. *J. Am. Chem. Soc.* **139**, 17667–17676 (2017).

(23) Saper, N., Ohgi, A., Small, D., Semba, K., Nakao, Y. & Hartwig, J. *Nat. Chem.* **12**, 276 (2020).

(24) Yu, X., Zhao, H., Li, P. & Koh, M. J. *J. Am. Chem. Soc.* **142**, 18223–18230 (2020).

(25) Meng, Q.-Y., Schirmer, T. E., Katou, K. & König, B. *Angew. Chem. Int. Ed.* **58**, 5723–5728 (2019).

(26) Poater, A., Ragone, F., Giudice, S., Costabile, C., Dorta, R., Nolan, S. P. & Cavallo, L. *Organometallics.* **27**, 2679−2681 (2008).

(27) Poater, A., Ragone, F., Mariz, R., Dorta, R. & Cavallo, L. *Chem. Eur. J.* **16**, 14348−14353 (2010).

(28) Laplaza, R., Sobez, J.-G., Wodrich, M. D., Reiher, M. & Corminboeuf, C. The (Not so) Simple Prediction of Enantioselectivty - A Pipeline for High-Fidelity Computations. *Chem. Sci.* **13**, 6858–6864. https://doi.org/10.1039/D2SC01714H (2022).

(29) Wodrich, M. D., Laplaza, R., Cramer, N., Reiher, M. & Corminboeuf, C. Toward in Silico Catalyst Optimization. *Chimia* **77**, 139–143 (2023).

(30) Chai, J.-D. & Head-Gordon, M. Long-Range Corrected Hybrid Density Functionals with Damped Atom–Atom Dispersion Corrections. *Phys. Chem. Chem. Phys.* **10**, 6615–6620 (2008).

(31) Weigend, F. & Ahlrichs, R. Balanced Basis Sets of Split Valence, Triple Zeta Valence and Quadruple Zeta Valence Quality for H to Rn: Design and Assessment of Accuracy. *Phys. Chem. Chem. Phys.* **7**, 3297–3305. https://doi.org/10.1039/B508541A (2005).

(32) Gaussian 16, Revision C.01, Frisch, M. J., Trucks, G. W., Schlegel, H. B., Scuseria, G. E., Robb, M. A., Cheeseman, J. R., Scalmani, G., Barone, V., Petersson, G. A., Nakatsuji, H., Li, X., Caricato, M., Marenich, A. V., Bloino, J., Janesko, B. G., Gomperts, R., Mennucci, B., Hratchian, H. P., Ortiz, J. V., Izmaylov, A. F., Sonnenberg, J. L., Williams-Young, D., Ding, F., Lipparini, F., Egidi, F., Goings, J., Peng, B., Petrone, A., Henderson, T., Ranasinghe, D., Zakrzewski, V. G., Gao, J., Rega, N., Zheng, G., Liang, W., Hada, M., Ehara, M., Toyota, K., Fukuda, R., Hasegawa, J., Ishida, M., Nakajima, T., Honda, Y., Kitao, O., Nakai, H., Vreven, T., Throssell, K., Montgomery, J. A., Jr., Peralta, J. E., Ogliaro, F., Bearpark, M. J., Heyd, J. J., Brothers, E. N., Kudin, K. N., Staroverov, V. N., Keith, T. A., Kobayashi, R., Normand, J., Raghavachari, K., Rendell, A. P., Burant, J. C., Iyengar, S. S., Tomasi, J., Cossi, M., Millam, J. M., Klene, M., Adamo, C., Cammi, R., Ochterski, J. W., Martin, R. L., Morokuma, K., Farkas, O., Foresman, J. B., Fox, D. J. Gaussian, Inc., Wallingford CT, (2016). .

(33) Marenich, A. V., Cramer, C. J. & Truhlar, D. G. Universal Solvation Model Based on Solute Electron Density and on a Continuum Model of the Solvent Defined by the Bulk Dielectric Constant and Atomic Surface Tensions. *J. Phys. Chem. B* **113**, 6378–6396. https://doi.org/10.1021/jp810292n (2009).

(34) Grimme, S. Supramolecular Binding Thermodynamics by Dispersion-Corrected Density Functional Theory. *Chem. Eur. J.* **18**, 9955–9964 (2012).

(35) Martin, R. L., Hay, P. J. & Pratt, L. R. Hydrolysis of Ferric Ion in Water and Conformational Equilibrium. *J. Phys. Chem. A* **102**, 3565–3573 (1998).

(36) Luchini, G., Alegre-Requena, J. V., Guan, Y., Funes-Ardoiz, I., Paton, R. S. GoodVibes v3.0.1, http://doi.org/10.5281/zenodo.595246 (2019).

(37) Luchini, G., Alegre-Requena, J. V., Funes-Ardoiz, I. & Paton, R. S. GoodVibes: Automated Thermochemistry for Heterogeneous Computational Chemistry Data. *F1000Research* **9**, 291, (2020).
